# Nickel‐Catalyzed Suzuki–Miyaura Cross‐Coupling Reaction of Aliphatic Alcohol Derivatives

**DOI:** 10.1002/anie.202509657

**Published:** 2025-06-17

**Authors:** Chloe D. Wong, Lauren C. Bradford, Nadia Hirbawi, Elizabeth R. Jarvo

**Affiliations:** ^1^ Department of Chemistry University of California Irvine California 92617 USA

**Keywords:** Aliphatic alcohol, Aryl glutarimide, Cross‐coupling reactions, Nickel catalysis, Suzuki–Miyaura

## Abstract

Reactions leveraging readily available starting materials are valuable for synthetic utility, especially for late‐stage functionalization. Herein, we report a nickel‐catalyzed Suzuki–Miyaura cross‐coupling (XC) reaction of aliphatic sulfonates with aryl boronic acids. This reaction provides straightforward access to an array of arylated products in good yield. We have established a one‐pot protocol allowing for the arylation of alcohols in a single reaction vessel. Additionally, an asymmetric reaction was developed to provide enantioenriched α‐aryl and heteroaryl glutarimides in good yield and enantioselectivity.

## Graphical abstract

A nickel‐catalyzed Suzuki–Miyaura coupling reaction of aliphatic mesylates is reported. This reaction provides a strategy for late‐stage functionalization of bioactive compounds that have high F(sp^3^). Preliminary mechanistic experiments confirm a stereoablative reaction outcome, consistent with alkyl radical intermediates. Application in an enantioselective transformation is demonstrated for the synthesis of enantioenriched α‐aryl glutaramides.

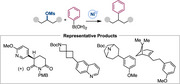

## Introduction

**Scheme 1:**
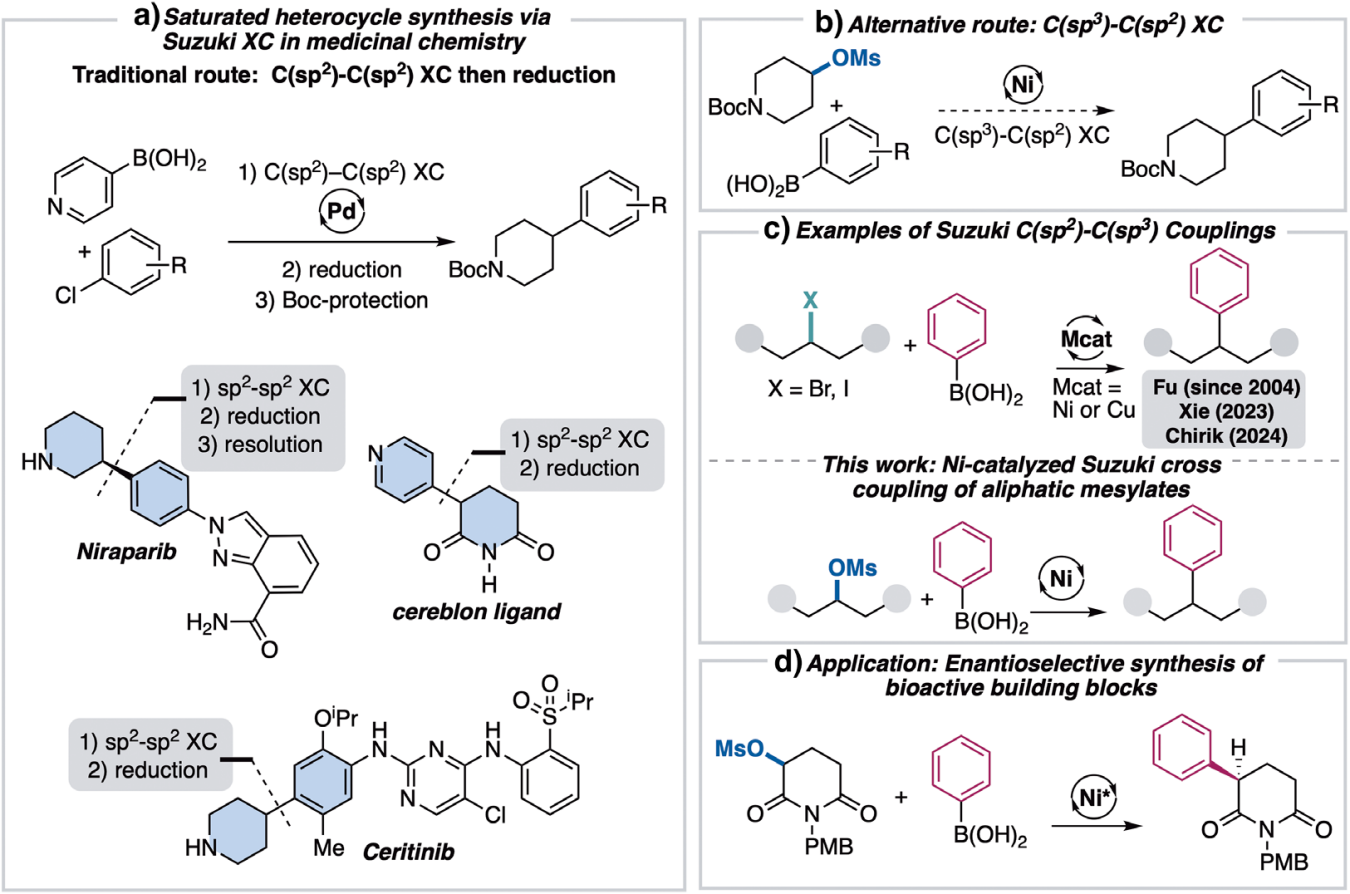
Suzuki–Miyaura reaction progress toward development of C(sp^3^) coupling.

Carbon–carbon bond forming reactions are essential for the construction of medicinal agents, natural products, and agrochemicals. Of these reactions, transition‐metal‐catalyzed cross‐coupling (XC) and cross‐electrophile coupling (XEC) reactions have had a significant impact on synthetic chemistry.^[^ ^1^, ^2^, ^3^ ^]^ The Suzuki–Miyaura XC reaction has served as a powerful workhorse transformation, facilitating C─C bond formation between halides or pseudohalides and boronic acid starting materials.^[^ ^4^, ^5^, ^6^ ^]^ The Suzuki–Miyaura XC reaction holds many advantages in comparison to other transition‐metal‐catalyzed XC systems due to the wide availability of boronic acids, ease of handling of reagents, and mild reaction conditions. For these reasons, it is extensively used in the pharmaceutical industry and is responsible for forging C─C bonds in a number of medicinal agents. Most strategies rely on traditional biaryl coupling, although coupling of aliphatic electrophiles would provide a more direct route. For example, approaches to nitrogen‐containing medicinal agents, including ALK inhibitor Ceritinib, PARP inhibitor Niraparib, and cereblon ligand, rely on C(sp^2^)─C(sp^2^) coupling followed by reduction of the moiety to the saturated heterocycle (Scheme 1a).^[^ ^2^, ^7^, ^8^ ^]^ A more direct strategy would be coupling a substituted piperdine; however, such couplings are less well developed (Scheme 1b).^[^ ^9^ ^]^

Current approaches for C(sp^2^)─C(sp^3^) Suzuki–Miyaura reactions are limited to halide electrophiles (Scheme 1b).^[^ ^10^, ^11^, ^12^, ^13^, ^14^, ^15^, ^16^, ^17^ ^]^ Our lab envisioned that the development of a Suzuki–Miyaura XC reaction that transforms C─O bonds of aliphatic alcohol derivatives to C─C bonds would be of value, particularly for late‐stage functionalization transformations. Specifically, reactions employing aliphatic alcohol derivatives as coupling partners are desirable considering the prevalence of alcohols in medicinal agents and natural products.^[^ ^18^, ^19^, ^20^ ^]^ Alcohols are also more commercially available in comparison to other coupling partners such as halides and amines. However, XC and XEC reactions of aliphatic alcohol derivatites remain underdeveloped.^[^ ^21^, ^22^, ^23^, ^24^, ^25^, ^26^, ^27^, ^28^, ^29^, ^30^ ^]^

In this manuscript we describe the development of a nickel‐catalyzed Suzuki–Miyaura XC of aliphatic mesylates with aryl boronic acids (Scheme 1c). The XC system features a diverse substrate pool and broad scope of boronic acids. As part of this strategy, we also developed a one‐pot protocol, allowing for arylation of alcohols in an efficient manner. Additionally, mechanistic studies are consistent with a stereoablative reaction outcome. Based on this mechanistic understanding, we developed enantioselective stereoconvergent reaction conditions employing a chiral nickel catalyst for the synthesis of enantioenriched α‐aryl substituted glutarimides (Scheme 1d).

## Results and Discussion

Reaction optimization began with biaryl mesylate **1** and reagents that are commonly utilized in Suzuki–Miyaura reactions, including a broad range of bases and alcohol solvents (Table 1). We also employed a halide salt to facilitate displacement of the alkyl sulfonate. We identified a combination of NiBr_2_(dme) and Dtbbpy in the presence of K_3_PO_4_, NaI, water, and *t*‐BuOH that provided a high yield of product **2** with minimal recovered iodide **3** (Table 1, entry 1). Ligand evaluation demonstrated that utilization of a phenanthroline ligand and a more electron‐rich bipyridine ligand did not increase yield (entries 2 and 3). Alternative solvent conditions to include different alcohols or an ethereal cosolvent had no positive effect on the reaction outcome (entries 4 and 5). Alternative precatalysts, lowering reaction temperature, and sodium iodide equivalents all resulted in decreased yields of **2** (entries 6–8). Omitting water drastically lowered the yield, and alkoxide bases shut down reactivity and resulted in recovery of iodide **3** (entries 9 and 10). We observed no reaction in the absence of the ligand (entry 11). Finally, reaction with no nickel catalyst or ligand resulted in no arylated product and is consistent with the nickel catalyst and ligand playing a critical role in the XC reaction (entry 12).

**Table 1:** Optimization of the reaction.

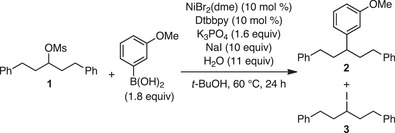			
Entry	Deviation from standard conditions	Yield 2 (%)^a)^	Yield 3 (%)^a)^
1	None	89 (92)^b)^	7
2	BPhen instead of Dtbbpy	72	19
3	4,4′‐(MeO)_2_‐bipy instead of Dtbbpy	51	28
4	*s*‐BuOH instead of *t*‐BuOH	<2	93
5	*t*‐BuOH:Dioxane (1:1) instead of *t*‐BuOH	66	17
6	Ni(cod)_2_ instead of NiBr_2_(dme)	65	<2
7	40 °C instead of 60 °C	44	65
8	5 equiv NaI	49	23
9	No H_2_O	58	12
10	KO*t*‐Bu instead of K_3_PO_4_	<2	94
11	No Dtbbpy	<2	87
12	No NiBr_2_(dme) or Dtbbpy	<2	93
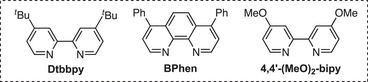			

^a)^ Yield determined by ^1^H NMR based on PhTMS as an internal standard.

^b)^ Isolated yield.

**Scheme 2:**
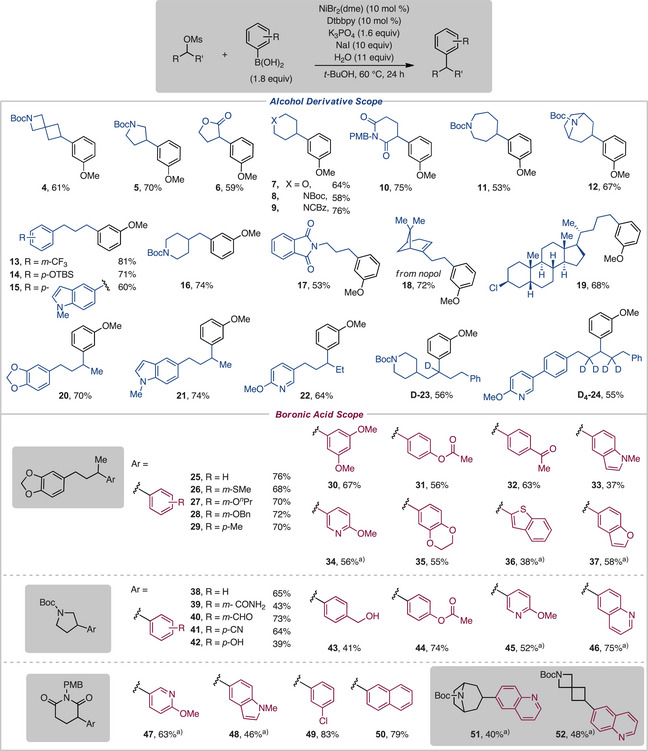
Aliphatic mesylate and boronic acid scope. ^a)^Reaction performed with 2.1 equiv of boronic acid and 15 equiv of NaI.

With the optimized reaction conditions established, we evaluated a variety of aliphatic mesylates to explore substrate scope (Scheme 2). We were pleased to observe a series of primary and secondary aliphatic mesylates were well tolerated in our reaction. Cyclic aliphatic coupling partners tested include an array of medicinally valuable saturated heterocycles^[^ ^31^ ^]^ of different ring sizes, including four‐, five‐, six‐, and seven‐membered rings, generating the arylated product in good to high yield (**4–12**). Primary mesylates containing pendant aryl groups substituted with electron‐donating and withdrawing groups performed well (**13** and **14**). Other primary substrates containing heterocycles, including indole (**15**), piperidine (**16**), and phthalimide (**17**), formed the desired product. Excitingly, the terpenoid, nopol (**18**), and lithocholic acid derivative containing an alkyl chloride (**19**) were well tolerated. Secondary acyclic substrates containing pendant aryl groups, including benzodioxole (**20**), indole (**21**), and pyridine (**22**), afforded the corresponding arylated product in good yield. Additionally, mesylates bearing deuterium labels coupled successfully (**D‐23** and **D_4_‐24**), which could have applications for pharmacokinetic studies in medicinal chemistry campaigns.^[^ ^32^, ^33^, ^34^ ^]^

Next, we evaluated a series of aryl boronic acids. To fully challenge the scope and versatility of this XC strategy, we examined a number of different substrate and aryl boronic acid combinations. A secondary acyclic mesylate featuring a benzodioxole was one of our model substrates for aryl boronic acid evaluation. For this compound, arenes substituted with electron‐donating groups were all compatible with our reaction conditions (**25–30**). Boronic acids featuring carbonyl moieties, including an ester (**31**) and ketone (**32**), in addition to an array of heterocyclic boronic acids, were found to furnish the desired arylated product in fair yield (**33–37**).

Additionally, we evaluated model substrates in the boronic acid scope that included a variety of nitrogen‐containing saturated heterocycles. With these substrates, we observed tolerance of arenes with electron‐donating groups (**38**, **42–44**, and **50**). Aryl boronic acids featuring electron‐withdrawing groups also afforded arylated products in good yields, including amide (**39**), aldehyde (**40**), nitrile (**41**), and chloride (**49**). A select few examples of heteroaryl boronic acids were also tolerated with the saturated heterocyclic mesylates in modest to good yield (**45–48** and **51–52**).

**Scheme 3:**
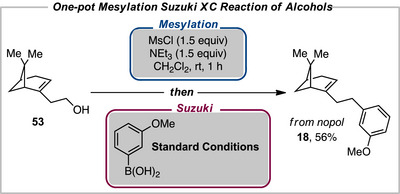
One‐pot arylation of alcohols.

We sought to develop a one‐pot procedure where the alcohol could be mesylated and then converted to the iodide to undergo the XC reaction. As proof of concept, we were delighted to find that arylated nopol could be furnished in good yield in a one‐pot manner through a sequential mesylation and XC reaction (Scheme 3). Therefore, this reaction provides an efficient approach to access a number of arylated products directly from simple and abundant starting materials in a single reaction vessel.

**Scheme 4:**
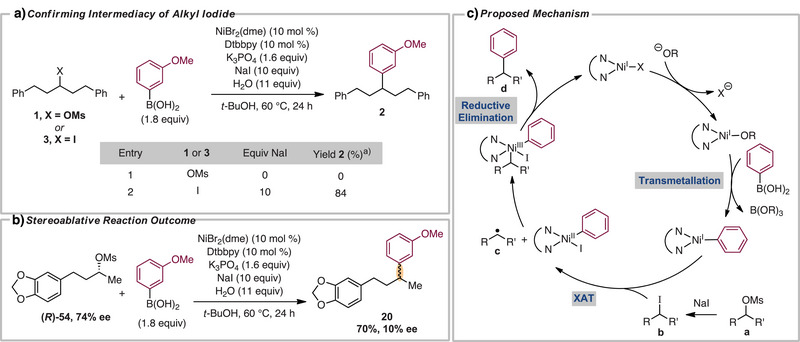
Mechanistic studies. ^a)^Yield determined by ^1^H NMR based on PhTMS as an internal standard.

We performed a series of experiments to provide insight into the reaction mechanism (Scheme 4). Based on prior work in our lab, we proposed that in situ displacement of sulfonates by iodide salts provided alkyl iodides that are key intermediates.^[^ ^23^, ^35^, ^36^, ^37^, ^38^, ^39^ ^]^ Omitting halide salt with the reaction of mesylate **1** resulted in exclusive recovery of starting material (Scheme 4a). Furthermore, subjecting iodide **3** to standard reaction conditions resulted in an 84% yield of the arylated product. Therefore, we propose that the reaction proceeds through the formation of alkyl iodides in situ. We next turned our attention to the stereochemical outcome of the reaction (Scheme 4b). Alkyl iodides in the presence of nickel catalysts typically undergo oxidative addition steps proceeding through halogen atom transfer (XAT) to yield radical intermediates, resulting in a stereoablative outcome at the secondary center.^[^ ^35^, ^40^, ^41^, ^42^, ^43^, ^44^ ^]^ Enantioenriched mesylate **(*R*)‐54** was synthesized and subjected to the XC reaction. The results showed a near‐racemic mixture of the arylated product, consistent with the presence of an alkyl radical intermediate. Based on these experiments, a plausible mechanism of the reaction is outlined in Scheme 4c. Transmetallation of the boronic acid with the nickel‐oxo intermediate provides an aryl Ni(I) intermediate. Displacement of mesylate **a** results in generation of the alkyl iodide **b**. Iodide **b** will then undergo oxidative addition through XAT with the Ni(I) catalyst to provide alkyl radical **c**. Recombination of **c** with Ni(II) species provides an organonickel(III) intermediate; subsequent reductive elimination affords the desired product **d**. The mechanism is analogous to those proposed for nickel‐catalyzed Suzuki–Miyaura coupling of alkyl bromides in which nickel(I)–(III) cycles are commonly invoked.^[^ ^11^, ^14^, ^45^ ^]^ Mechanisms involving radical chains or initiation by oxidative addition of a nickel(0) catalyst are also reasonable.^[^ ^46^, ^47^, ^48^ ^]^

**Scheme 5:**
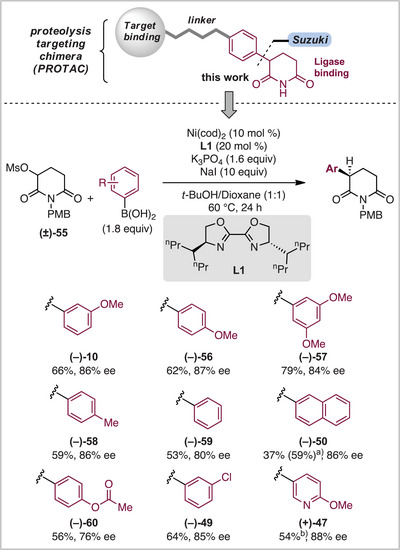
Enantioselective reaction scope of α‐aryl glutarimides. ^a)^Yield determined by ^1^H NMR based on PhTMS as an internal standard. ^b)^Reaction performed with 2.1 equiv of boronic acid and 15 equiv of NaI.

Based on confirmation of the stereoablative reaction outcome, we investigated whether the reaction could be performed in a stereoconvergent and enantioselective manner when a chiral ligand is employed. Surprisingly, a limited number of enantioselective nickel‐catalyzed Suzuki–Miyaura XC reactions for C(sp^2^)─C(sp^3^) have been reported.^[^ ^15^, ^16^, ^17^ ^]^ We envisioned the development of a Suzuki–Miyaura reaction to provide enantioenriched α‐aryl and heteroaryl glutarimides, compounds of interest to the field of targeted protein degradation (Scheme 5.^[^ ^8^, ^49^, ^50^, ^51^, ^52^ ^]^ This reaction would be complementary to the electrochemical and asymmetric reductive XEC reactions recently reported for this moiety.^[^ ^53^, ^54^ ^]^ We employed mesylate **55** and evaluated a broad range of chiral ligands commonly utilized in asymmetric nickel catalysis. 4‐HeptyBiOx (**L1**) provided the desired arylated product in high yield and enantioselectivity.^[^ ^55^ ^]^ Interestingly, this is the same ligand employed by Reisman and co‐workers,^[^ ^53^ ^]^ consistent with a similar reductive elimination step for both the XC and XEC reactions. With enantioselective reaction conditions established, we explored an array of aryl boronic acids. Simple arenes reacted smoothly in the XC reaction, providing the desired product in good yield and ee (**(−)‐10**, **(−)‐50**, and **(−)**‐**56**–**(−)**‐**59**). Arenes substituted with functionalities such as an ester (**(−)‐60**) and chloride (**(−)‐49**), in addition to a heteroaryl pyridine boronic acid (**(+)‐47**), were all compatible in our reaction scheme. We anticipate this reaction will have applications in medicinal chemistry as well as be informative for the development of future enantioselective nickel‐catalyzed XC and XEC reactions.

## Conclusion

In conclusion, we report a nickel‐catalyzed Suzuki–Miyaura XC reaction of aliphatic sulfonates with aryl boronic acids for the installation of aryl groups. The reaction features a broad scope of aliphatic mesylates and boronic acids, including heteroaryl boronic acids. The reaction can be performed in a one‐pot manner, allowing for the direct arylation of alcohols. Mechanistic studies confirmed the stereoablative nature of the reaction and the intermediacy of alkyl radicals. Finally, we rendered the transformation to be enantioselective with a chiral bisoxazoline ligand for the synthesis of enantioenriched bioactive building blocks.

## Conflict of Interests

The authors declare no conflict of interest.

## Supporting information



Supporting Information

## Data Availability

The data that support the findings of this study are available in the supplementary material of this article.
