# Supplementary material for: Nickel‐Catalyzed Suzuki–Miyaura Cross‐Coupling Reaction of Aliphatic Alcohol Derivatives
Source: Angew Chem Int Ed Engl. 2025 Jun 17;64(31):e202509657. doi: 10.1002/anie.202509657 (PMC12304806; doi:10.1002/anie.202509657)
Supplement: Supplementary file 1 — Supporting Information [file ANIE-64-e202509657-s001.pdf]

Supporting Information

Nickel-Catalyzed Suzuki-Miyaura Cross-Coupling Reaction of Aliphatic Alcohol Derivatives

Chloe D. Wong, Lauren C. Bradford, Nadia Hirbawi, Elizabeth R. Jarvo*

Department of Chemistry, University of California, Irvine, CA 92697-2025

*Corresponding author: erjarvo@uci.edu

Table of Contents

I. **General Procedures** SI-2
II. **Experimental** SI-3
A. General Suzuki Cross-Coupling (XC) Reaction Procedures SI-3
1. Method A: XC for Synthesis of Racemic Arylated Products SI-3
2. Method B: Modified Racemic Conditions XC Procedure for Heteroaryl Boronic Acids SI-3
3. Method C: XC for Synthesis of Enantioenriched Arylated Products SI-4
4. Method D: Modified Enantioselective XC Procedure for Heteroaryl Boronic Acids SI-4
5. Method E: One-pot Mesylation Suzuki Reaction Procedure SI-5
6. Scheme SI-1: Chiral Ligand Evaluation SI-6
B. Experimental Data for Mechanistic Studies SI-7
1. Confirming Intermediacy of Alkyl Iodides SI-7
2. Stereochemical Outcome of the Reaction SI-7
C. Characterization Data for Arylated Products SI-8
1. Racemic Products SI-8
2. Enantioenriched Products SI-33
D. General Procedures for Starting Material Synthesis SI-37
1. Method F: Mesylation of Alcohol SI-37
E. Synthesis and Characterization Data of Alcohols, Mesylates, and Iodide SI-38
1. Substrate Scope SI-38
2. Mechanistic Studies SI-49
III. **References for Supporting Information** SI-51
IV. **SFC Traces** SI-52
V. **NMR Spectra** SI-63

I. GENERAL PROCEDURES

All reactions were carried out under an atmosphere of N_2 when noted. All glassware was oven- or flame-dried prior to use. Dichloromethane (CH_2Cl_2) was degassed with Ar and then passed through two 4 x 36-inch columns of anhydrous neutral A-2 alumina (8 x 14 mesh; LaRoche Chemicals; activated under a flow of argon at 350 °C for 12 h) to remove H_2O. All other solvents utilized were purchased "anhydrous" commercially or purified as described. ^{1}H NMR spectra were recorded on AVANCE-400 (400 MHz ^{1}H, 100.6 MHz ^{13}C), NEO-400 (400 MHz ^{1}H), GN-500 (500 MHz ^{1}H, 125.7 MHz ^{13}C) or AVANCE-600 (600 MHz ^{1}H, 150.9 MHz ^{13}C, 564.7 MHz ^{19}F) spectrometers. Proton chemical shifts are reported in ppm (δ) relative to internal tetramethylsilane (TMS, δ 0.00). Data are reported as follows: chemical shift (multiplicity [singlet (s), broad singlet (br s), doublet (d), doublet of doublets (dd), doublet of doublet of doublets (ddd), triplet (t), doublet of triplets (dt), doublet of quartets (dq), triplet of doublets (td), quartet (q), multiplet (m), apparent singlet (ap s), apparent doublet (ad), apparent triplet (at), apparent quartet (aq), apparent quintet (aquint)], coupling constants [Hz], integration). Carbon chemical shifts are reported in ppm (δ) relative to TMS with the respective solvent resonance as the internal standard ($CDCl_3$, δ 77.16 ppm). NMR data were collected at 25 °C. Analytical thin-layer chromatography (TLC) was performed using Silica Gel 60 F254 precoated plates (0.25 mm thickness). Visualization was accomplished by irradiation with a UV lamp or stain if noted. Flash chromatography was performed using SilicaFlash F60 (40-63 μm, 60 Å) from SiliCycle or Teledyne Isco Combiflash® Rf+ automated flash chromatography system. Optical rotations were measured on a Rudolph Research Analytical Autopol III Automatic Polarimeter. Determination of enantiomeric ratios were performed by chiral SFC analysis and performed on an Agilent Technologies 1260 Infinity Analytical SFC system using OB-H, OD-H, OJ-H, and AD-H Chiralpak columns (100 bar, 50 ºC, 254 nm). High resolution mass spectrometry was performed by the University of California, Irvine Mass Spectrometry Center.

Bis(1,5-cyclooctadiene)nickel and nickel(II) bromide ethylene glycol dimethyl ether complex was purchased from Strem, stored in a glovebox under an atmosphere of N_2 and used as received. All ligands were purchased from Strem, Sigma Aldrich or Ambeed and were stored under N_2 atmosphere and used as received. All other chemicals were purchased commercially and used as received, unless otherwise noted.

II. EXPERIMENTAL

A. General Suzuki Cross-Coupling (XC) Reaction Procedures

1. Method A: XC for Synthesis of Racemic Arylated Products

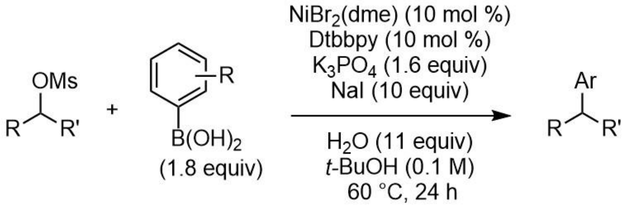

In a glovebox, an oven-dried 7-mL vial equipped with a stir bar was charged with mesylate (1.0 equiv), boronic acid (1.8 equiv), nickel(II) bromide ethylene glycol dimethyl ether complex ($NiBr_2$(dme), 10. mol %), 4,4′-Di-tert-butyl-2,2′-dipyridyl (Dtbbpy; 10. mol %), potassium phosphate (K_3PO_4; 1.6 equiv), and sodium iodide (NaI; 10. equiv). The vial was sealed with an open-top cap with septa before removing from the glovebox. Water (H_2O; 11 equiv) and anhydrous *tert*-butanol (*t*-BuOH) (0.10 M in substrate) were added outside the glovebox the vial was placed in an oil bath heated to 60 ºC. After 24 h, the reaction was allowed to cool to rt before filtering through a plug of silica gel (eluting with 100% Et_2O or EtOAc for polar substrates) and concentrated in vacuo. Phenyltrimethylsilane (PhTMS; 8.6 μL, 50. μmol) was added to determine the yield by ^{1}H NMR (aryl multiplets ~7 ppm) based on comparison to PhTMS as internal standard.

2. Method B: Modified Racemic Conditions XC Procedure for Heteroaryl Boronic Acids

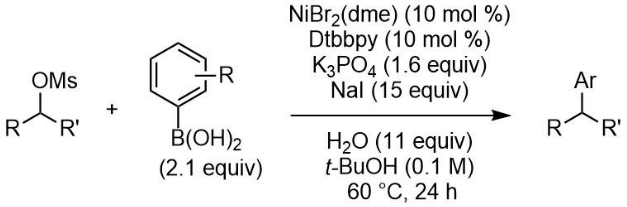

In a glovebox, an oven-dried 7-mL vial equipped with a stir bar was charged with mesylate (1.0 equiv), boronic acid (2.1 equiv), $NiBr_2$(dme) (10. mol %), Dtbbpy (10. mol %), K_3PO_4 (1.6 equiv), and NaI (15 equiv). The vial was sealed with an open-top cap with septa before removing from the glovebox. H_2O (11 equiv) and anhydrous *t*-BuOH (0.10 M in substrate) were added outside the glovebox the vial was placed in an oil bath heated to 60 ºC. After 24 h, the reaction was allowed to cool to rt before filtering through a plug of silica gel (eluting with 100% Et_2O or EtOAc for polar substrates) and concentrated in vacuo. Phenyltrimethylsilane (PhTMS; 8.6 μL, 50. μmol) was added to determine the yield by ^{1}H NMR (aryl multiplets ~7 ppm) based on comparison to PhTMS as internal standard.

3. Method C: XC for Synthesis of Enantioenriched Arylated Products

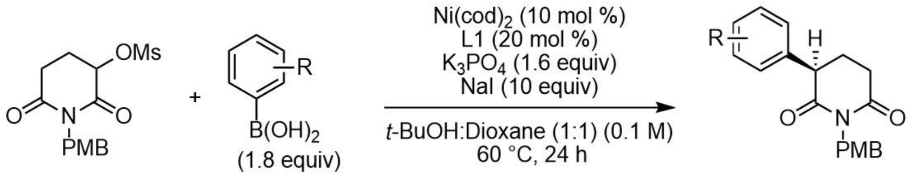

In a glovebox, an oven-dried 7-mL vial equipped with a stir bar was charged with mesylate (1.0 equiv), boronic acid (1.8 equiv), bis(1,5-cyclooctadiene)nickel (Ni(cod)$_2$; 10. mol %), (4S,4'S)-4,4'-Di(heptan-4-yl)-4,4',5,5'-tetrahydro-2,2'-bioxazole ((*S*)-4-heptylBiOX; (20. mol %),) K_3PO_4 (1.6 equiv), and NaI (10. equiv). The vial was sealed with an open-top cap with septa before removing from the glovebox. Anhydrous 1,4-Dioxane and *t*-BuOH (1:1, 0.10 M in substrate) were added outside the glovebox the vial was placed in an oil bath heated to 60 ºC. After 24 h, the reaction was allowed to cool to rt before filtering through a plug of silica gel (eluting with 100% Et_2O or EtOAc for polar substrates) and concentrated in vacuo. Absolute configuration of products was determined from analogy to work by Reisman and coworkers.[1] Phenyltrimethylsilane (PhTMS; 8.6 μL, 50. μmol) was added to determine the yield by ^{1}H NMR (aryl multiplets ~7 ppm) based on comparison to PhTMS as internal standard.

4. Method D: Modified Enantioselective XC Procedure for Heteroaryl Boronic Acids

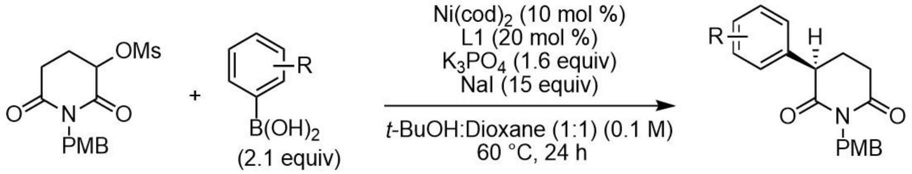

In a glovebox, an oven-dried 7-mL vial equipped with a stir bar was charged with mesylate (1.0 equiv), boronic acid (2.1 equiv), Ni(cod)$_2$ (10. mol %), (*S*)-4-heptylBiOX (20. mol %), K_3PO_4 (1.6 equiv), and NaI (15 equiv). The vial was sealed with an open-top cap with septa before removing from the glovebox. Anhydrous 1,4-Dioxane and *t*-BuOH (1:1, 0.10 M in substrate) were added outside the glovebox the vial was placed in an oil bath heated to 60 ºC. After 24 h, the reaction was allowed to cool to rt before filtering through a plug of silica gel (eluting with 100% Et_2O or EtOAc for polar substrates) and concentrated in vacuo. Absolute configuration of products was determined from analogy to work by Reisman and coworkers.[1] Phenyltrimethylsilane (PhTMS; 8.6 μL, 50. μmol) was added to determine the yield by ^{1}H NMR (aryl multiplets ~7 ppm) based on comparison to PhTMS as internal standard.

5. Method E: One-pot Mesylation Suzuki Reaction Procedure

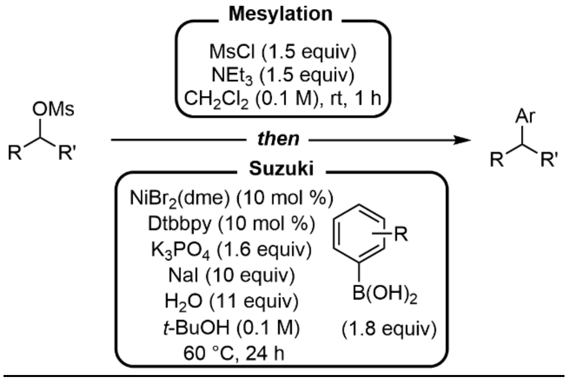

To a flame-dried 7-mL vial equipped with a stir bar was added alcohol (1.0 equiv) and CH_2Cl_2 (0.20 M in substrate) under N_2. Then, Et_3N (1.5 equiv) and MsCl (1.5 equiv) were added, and the reaction mixture was allowed to stir at rt 1 h. The reaction was concentrated in vacuo and pumped into glovebox. In a glovebox, an oven-dried 7-mL vial equipped with a stir bar was charged with boronic acid (1.8 equiv), $NiBr_2$(dme) (10. mol %), Dtbbpy (10. mol %), K_3PO_4 (1.6 equiv), and NaI (10. equiv). The vial was sealed with an open-top cap with septa before removing from the glovebox. H_2O (11 equiv) and anhydrous *t*-BuOH (0.10 M in substrate) were added outside the glovebox the vial was placed in an oil bath heated to 60 ºC. After 24 h, the reaction was allowed to cool to rt before filtering through a plug of silica gel (eluting with 100% Et_2O or EtOAc for polar substrates) and concentrated in vacuo. Phenyltrimethylsilane (PhTMS; 8.6 μL, 50. μmol) was added to determine the yield by ^{1}H NMR (aryl multiplets ~7 ppm) based on comparison to PhTMS as internal standard.

6. Optimization Data

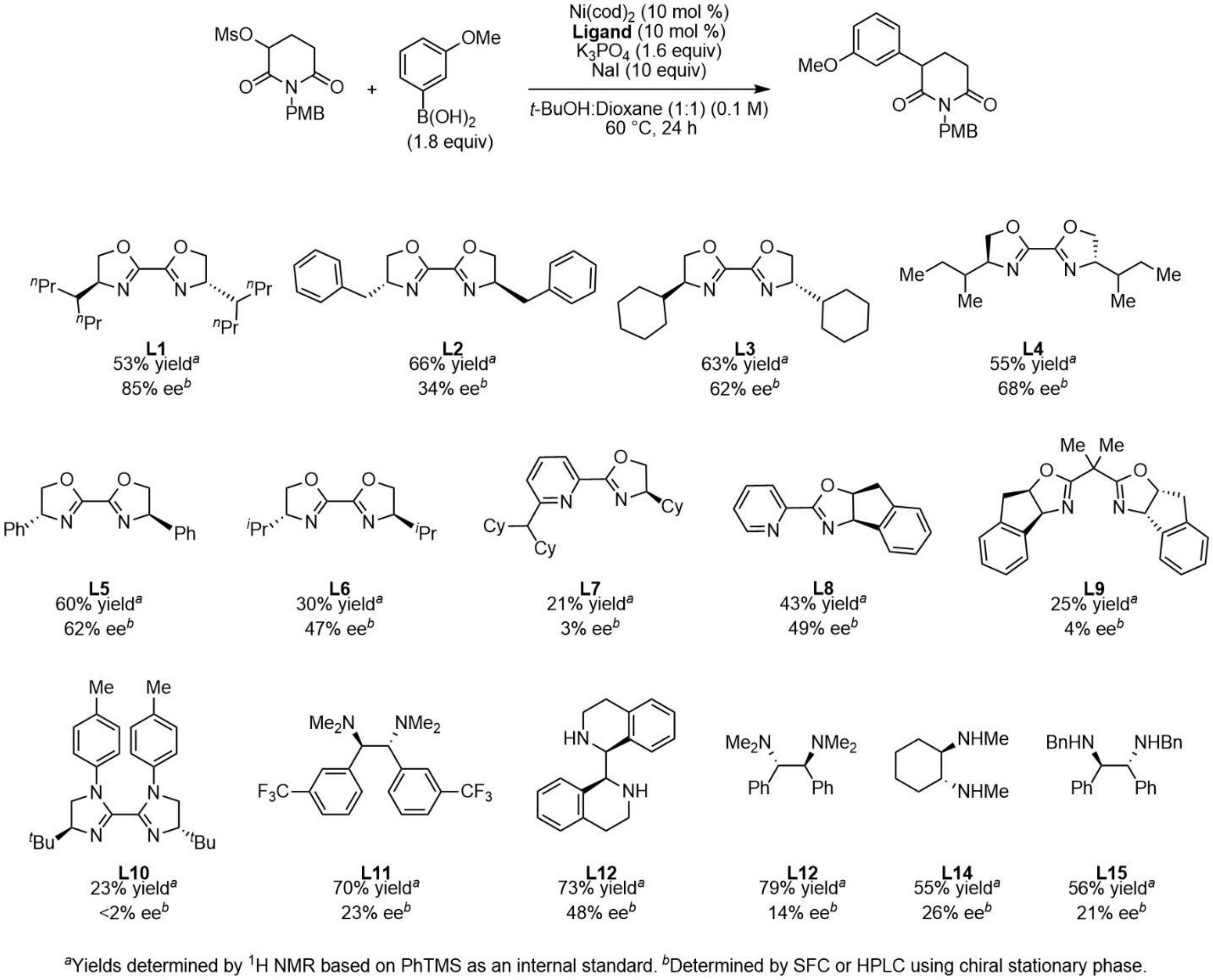

Scheme SI-1: Chiral Ligand Evaluation

B. Experimental Data for Mechanistic Studies

1. Confirming Intermediacy of Alkyl Iodide

To confirm the intermediacy of the secondary alkyl iodide, alkyl iodide **3** was synthesized and subjected to Method A.

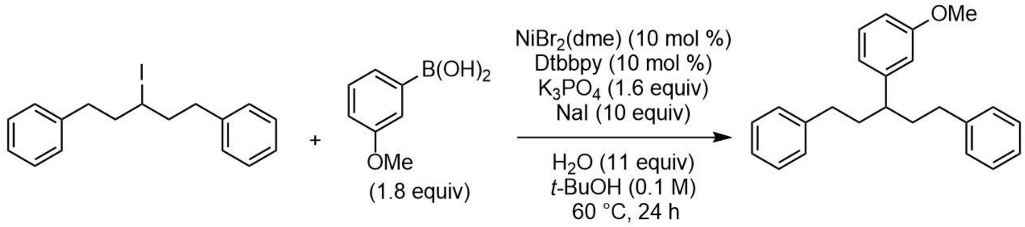

Scheme SI-2: Confirming Intermediacy of Alkyl Iodide

Arylated product **2** was synthesized from iodide **3** following Method A. The following amounts of reagents were used: iodide **3** (35 mg, 0.10 mmol, 1.0 equiv), boronic acid (28 mg, 1.8 mmol, 1.8 equiv), $NiBr_2$(dme) (3.1 mg, 10. μmol, 10. mol %), Dtbbpy (2.7 mg, 10. μmol, 10. mol %), K_3PO_4 (34 mg, 0.16 mmol, 1.6 equiv), NaI (150 mg, 1.0 mmol, 10. equiv), H_2O (20. μL, 1.1 mmol, 11 equiv), and anhydrous *t*-BuOH (1.0 mL, 0.10 M in substrate). A ^{1}H NMR yield of 84% was obtained based on comparison to PhTMS as internal standard.

2. Stereochemical Outcome of the Reaction

The stereochemical outcome of the reaction was determined by subjecting enantioenriched mesylate **(*S*)-54** to Method A (Scheme SI-3).

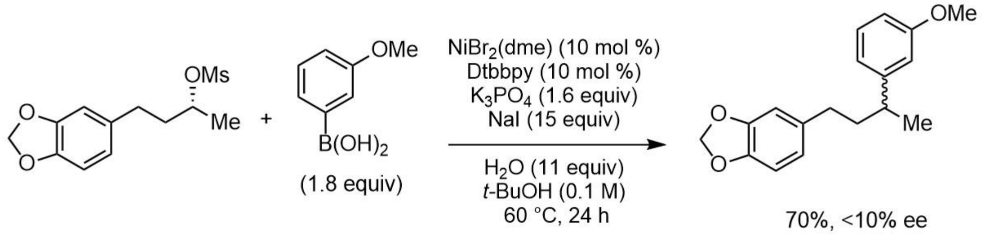

Scheme SI-3: Proof of Stereochemical Outcome for arylated product **20**

We conclude that the XC reaction proceeds with epimerization at the mesylate center through a stereoablative oxidative addition pathway because the arylated product is generated a racemic mixture determined by chiral SFC analysis of product **20**.

C. Characterization Data for Arylated Products

1. Racemic Products

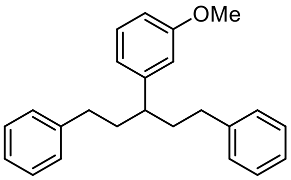

Arylated product **2** was prepared according to Method A. The following amounts of reagents were used: mesylate **1** (32 mg, 0.10 mmol, 1.0 equiv), boronic acid (27 mg, 0.18 mmol, 1.8 equiv), $NiBr_2$(dme) (3.1 mg, 10 µmol, 10. mol %), Dtbbpy (2.7 mg, 10 µmol, 10. mol %), K_3PO_4 (34 mg, 0.16 mmol, 1.6 equiv), NaI (150 mg, 0.10 mmol, 10. equiv), H_2O (20. µL, 1.0 mmol, 11 equiv), and anhydrous *t*-BuOH (1.0 mL, 0.10 M in substrate). The residue was then purified by flash column chromatography (0–20% benzene/hexanes) to afford the title compound as a clear oil (30. mg, 92 µmol, 92%). **TLC R_f** = 0.3 (20% benzene/hexanes, CAM stain); **^{1}H NMR** (500 MHz, $CDCl_3$) δ 7.27–7.22 (m, 5H), 7.16–7.13 (m, 2H), 7.09–7.08 (m, 4H), 6.81–6.74 (m, 3H), 3.82 (s, 3H), 2.58–2.52 (m, 1H), 2.46 (at, *J* = 8.0 Hz, 4H), 2.00–1.86 (m, 4H); **^{13}C NMR** (150.9 MHz, $CDCl_3$) δ 159.9, 147.0, 142.6 (2C), 129.5, 128.5 (4C), 128.4 (4C), 125.8 (2C), 120.5, 113.9, 111.2, 55.3, 45.4, 38.8 (2C), 33.9 (2C); **HRMS** (TOF MS CI+) *m/z*: $[M]^+$ calculated for $C_{24}H_{26}O$, 330.1984; found 330.1981.

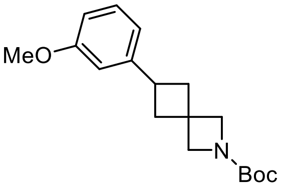

Arylated product **4** was prepared according to Method A. The following amounts of reagents were used: mesylate **SI-1** (29 mg, 0.10 mmol, 1.0 equiv), boronic acid (27 mg, 0.18 mmol, 1.8 equiv), $NiBr_2$(dme) (3.1 mg, 10. µmol, 10. mol %), Dtbbpy (2.7 mg, 10. µmol, 10. mol %), K_3PO_4 (34 mg, 0.16 mmol, 1.6 equiv), NaI (150 mg, 0.10 mmol, 10. equiv), H_2O (20. µL, 1.1 mmol, 11 equiv), and anhydrous *t*-BuOH (1.0 mL, 0.10 M in substrate). The residue was then purified by flash column chromatography (0–15% EtOAc/hexanes) to afford the title compound as a white solid (18 mg, 61 µmol, 61%). **TLC R_f** = 0.4 (15% EtOAc/hexanes, $KMnO_4$ stain); **^{1}H NMR** (500 MHz, $CDCl_3$) δ 7.23 (t, *J* = 7.8 Hz, 1H), 6.77–6.70 (m, 3H), 4.05 (s, 2H), 3.83 (s, 2H). 3.79 (s, 3H), 3.40 (quint, *J* = 9.0 Hz, 1H), 2.58 (t, *J* = 11.2 Hz, 2H), 2.29 (t, *J* = 10.2 Hz, 2H), 1.44 (s, 9H); **^{13}C NMR** (150.9 MHz, $CDCl_3$) δ 159.8, 156.4, 146.5, 129.5, 119.7, 112.4, 111.3, 79.4, 55.3 (2C), 40.4 (2C), 34.2, 34.0, 29.8, 28.6 (3C); **HRMS** (TOF MS ES+) *m/z*: $[M + Na]^+$ calculated for $C_{18}H_{25}NO_3Na$, 326.1732; found 326.1736.

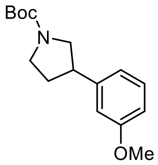

Arylated product **5** was prepared according to Method A. The following amounts of reagents were used: mesylate **SI-2** (27 mg, 0.10 mmol, 1.0 equiv), boronic acid (28 mg, 0.18 mmol, 1.8 equiv), $NiBr_2$(dme) (3.2 mg, 10. µmol, 10. mol %), Dtbbpy (2.8 mg, 10. µmol, 10. mol %), K_3PO_4 (35 mg, 0.16 mmol, 1.6 equiv), NaI (150 mg, 0.10 mmol, 10. equiv), H_2O (20. µL, 1.1 mmol, 11 equiv), and anhydrous *t*-BuOH (1.0 mL, 0.10 M in substrate). The residue was then purified by flash column chromatography (0–25% EtOAc/hexanes) to afford the title compound as a clear oil (20. mg, 72 µmol, 70%). **TLC R_f** = 0.6 (25% EtOAc/hexanes, $KMnO_4$ stain); **^{1}H NMR** (600 MHz, $CDCl_3$) δ (mixture of rotamers) 7.25–7.23 (m, 1H), 6.84 (d, *J* = 7.6 Hz, 1H), 6.78 (br s, 2H), 3.85–3.75 (m, 4H), 3.67 (t, *J* = 8.9 Hz, 0.5H), 3.57 (t, *J* = 9.4 Hz, 0.5H), 3.43–3.25 (m, 3H), 2.27–2.24 (m, 1H), 2.01 (quint, *J* = 9.5 Hz, 1H), 1.48 (d, *J* = 8.8 Hz, 9H). Analytical data is consistent with literature values.[2]

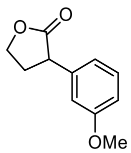

Arylated product **6** was prepared according to Method A. The following amounts of reagents were used: mesylate **SI-3** (20. mg, 0.11 mmol, 1.0 equiv), boronic acid (31 mg, 0.20 mmol, 1.8 equiv), $NiBr_2$(dme) (3.5 mg, 11 µmol, 10. mol %), Dtbbpy (3.0 mg, 11 µmol, 10. mol %), K_3PO_4 (38 mg, 0.18 mmol, 1.6 equiv), NaI (170 mg, 0.11 mmol, 10. equiv), H_2O (23. µL, 1.3 mmol, 11 equiv), and anhydrous *t*-BuOH (1.1 mL, 0.10 M in substrate). The residue was then purified by flash column chromatography (0–25% EtOAc/hexanes) to afford the title compound as a clear oil (13 mg, 67 µmol, 59%). **TLC R_f** = 0.2 (25% EtOAc/hexanes, $KMnO_4$ stain); **^{1}H NMR** (600 MHz, $CDCl_3$) δ 7.28 (ad, *J* = 14.7 Hz, 1H), 6.84 (br s, 3H), 4.48 (br s, 1H), 4.35 (br s, 1H), 3.81 (br s, 4H), 2.71 (br s, 1H), 2.44 (br s, 1H). Analytical data is consistent with literature values.[3]

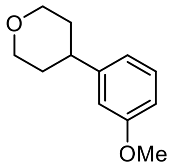

Arylated product **7** was prepared according to Method A. The following amounts of reagents were used: mesylate **SI-4** (21 mg, 0.11 mmol, 1.0 equiv), boronic acid (31 mg, 0.20 mmol, 1.8 equiv), $NiBr_2$(dme) (3.5 mg, 11 µmol, 10. mol %), Dtbbpy (3.0 mg, 11 µmol, 10. mol %), K_3PO_4 (39 mg, 0.18 mmol, 1.6 equiv), NaI (170 mg, 0.10 mmol, 10. equiv), H_2O (23. µL, 1.3 mmol, 11 equiv),

and anhydrous *t*-BuOH (1.1 mL, 0.10 M in substrate). The residue was then purified by flash column chromatography (0–25% EtOAc/hexanes) to afford the title compound as a clear oil (14 mg, 72 μmol, 64%). **TLC R_f** = 0.5 (25% EtOAc/hexanes, $KMnO_4$ stain); **^{1}H NMR** (500 MHz, $CDCl_3$) δ 7.24–7.22 (m, 1H), 6.83 (d, *J* = 7.7 Hz, 1H), 6.77–6.75 (m, 2H), 4.10–4.06 (m, 2H), 3.81 (s, 3H), 3.54 (t, *J* = 11.8 Hz, 2H), 2.76–2.71 (m, 1H), 1.86–1.75 (m, 4H). Analytical data is consistent with literature values.[4]

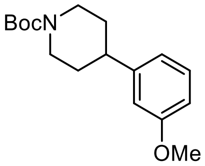

Arylated product **8** was prepared according to Method A. The following amounts of reagents were used: mesylate **SI-5** (28 mg, 0.10 mmol, 1.0 equiv), boronic acid (27 mg, 0.18 mmol, 1.8 equiv), $NiBr_2$(dme) (3.1 mg, 10. μmol, 10. mol %), Dtbbpy (2.7 mg, 10. μmol, 10. mol %), K_3PO_4 (34 mg, 0.16 mmol, 1.6 equiv), NaI (150 mg, 0.10 mmol, 10. equiv), H_2O (20. μL, 1.0 mmol, 11 equiv), and anhydrous *t*-BuOH (1.0 mL, 0.10 M in substrate). The residue was then purified by flash column chromatography (0–25% EtOAc/hexanes) to afford the title compound as a clear oil (16 mg, 58 μmol, 58%). **TLC R_f** = 0.6 (25% EtOAc/hexanes, PMA stain); **^{1}H NMR** (600 MHz, $CDCl_3$) δ 7.24–7.21 (m, 1H), 6.81 (d, *J* = 7.6 Hz, 1H), 6.76–6.75 (m, 2H), 4.24 (br s, 2H), 3.80 (s, 3H), 2.79 (br s, 2H), 2.64−2.59 (m, 1H), 1.83−1.81 (m, 2H), 1.60−1.59 (m, 2H), 1.48 (s, 9H). Analytical data is consistent with literature values.[5]

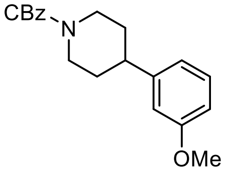

Arylated product **9** was prepared according to Method A. The following amounts of reagents were used: mesylate **SI-6** (35 mg, 0.10 mmol, 1.0 equiv), boronic acid (30. mg, 0.18 mmol, 1.8 equiv), $NiBr_2$(dme) (3.4 mg, 11 μmol, 10. mol %), Dtbbpy (2.9 mg, 11 μmol, 10. mol %), K_3PO_4 (38 mg, 0.16 mmol, 1.6 equiv), NaI (170 mg, 0.10 mmol, 10. equiv), H_2O (22. μL, 1.0 mmol, 11 equiv), and anhydrous *t*-BuOH (1.1 mL, 0.10 M in substrate). The residue was then purified by flash column chromatography (0–25% EtOAc/hexanes) to afford the title compound as a yellow oil (27 mg, 84 μmol, 76%). **TLC R_f** = 0.5 (25% EtOAc/hexanes, $KMnO_4$ stain); **^{1}H NMR** (500 MHz, $CDCl_3$) δ 7.37–7.31 (m, 5H), 7.23 (at, *J* = 7.9 Hz, 1H), 6.79–6.74 (m, 3H), 5.15 (br s, 2H), 4.35 (br s, 2H), 3.79 (s, 3H), 2.92–2.85 (m, 2H), 2.67–2.62 (m, 1H), 1.85–1.83 (m, 2H), 1.64–1.61 (m, 2H); **^{13}C NMR** (150.9 MHz, $CDCl_3$) δ 159.8, 155.4, 147.4, 136.9, 129.6, 128.6 (2C), 128.1, 128.0 (2C), 119.3, 112.9, 111.6, 67.2, 55.3, 44.7 (2C), 42.8 (2C), 30.5; **HRMS** (TOF MS ES+) *m/z*: $[M + Na]^+$ calculated for $C_{20}H_{23}NO_3Na$, 326.1756; found 326.1751.

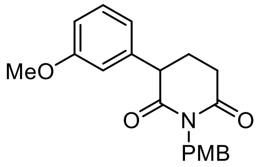

Arylated product **10** was prepared according to Method A. The following amounts of reagents were used: mesylate **55** (32 mg, 0.10 mmol, 1.0 equiv), boronic acid (27 mg, 0.18 mmol, 1.8 equiv), $NiBr_2$(dme) (3.0 mg, 10. µmol, 10. mol %), Dtbbpy (2.6 mg, 10. µmol, 10. mol %), K_3PO_4 (33 mg, 0.16 mmol, 1.6 equiv), NaI (150 mg, 0.10 mmol, 10. equiv), H_2O (20. µL, 1.1 mmol, 11 equiv), and anhydrous *t*-BuOH (1.0 mL, 0.10 M in substrate). The residue was then purified by flash column chromatography (0–25% EtOAc/hexanes) to afford the title compound as a clear oil (25 mg, 73 µmol, 75%). **TLC** $\mathbf{R_f}$ = 0.3 (25% EtOAc/hexanes, $KMnO_4$ stain); ^{1}**H NMR** (500 MHz, $CDCl_3$) δ 7.39 (d, *J* = 11.5 Hz, 2H), 7.25 (t, *J* = 8.1 Hz, 1H), 6.82 (d, *J* = 8.6 Hz, 3H), 6.69 (d, *J* = 7.6 Hz, 1H), 6.61 (br s, 1H), 4.99–4.92 (m, 2H), 3.81–3.78 (m, 1H), 3.78 (s, 3H), 3.72 (s, 3H), 2.78–2.64 (m, 2H), 2.24–2.12 (m, 2H); ^{13}**C NMR** (150.9 MHz, $CDCl_3$) δ 173.3, 172.3, 159.9, 159.1, 139.6, 130.9 (2C), 129.9, 129.7, 120.3, 114.0, 113.8 (2C), 112.9, 55.3, 55.3, 48.7, 42.7, 31.5, 25.3; **HRMS** (TOF MS ES+) *m/z*: $[M + Na]^+$ calculated for $C_{20}H_{21}NO_4Na$, 362.1368; found 362.1369.

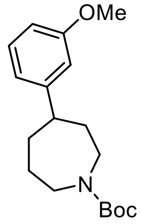

Arylated product **11** was prepared according to Method A. The following amounts of reagents were used: mesylate **SI-7** (29 mg, 0.10 mmol, 1.0 equiv), boronic acid (27 mg, 0.18 mmol, 1.8 equiv), $NiBr_2$(dme) (3.1 mg, 10. µmol, 10. mol %), Dtbbpy (2.7 mg, 10. µmol, 10. mol %), K_3PO_4 (34 mg, 0.16 mmol, 1.6 equiv), NaI (150 mg, 0.10 mmol, 10. equiv), H_2O (20. µL, 1.1 mmol, 11 equiv), and anhydrous *t*-BuOH (1.0 mL, 0.10 M in substrate). The residue was then purified by flash column chromatography (0–25% EtOAc/hexanes) to afford the title compound as a clear oil (16 mg, 53 µmol, 53%). **TLC** $\mathbf{R_f}$ = 0.7 (25% EtOAc/hexanes, $KMnO_4$ stain); ^{1}**H NMR** (500 MHz, $CDCl_3$) δ (mixture of rotamers) 7.21 (q, *J* = 7.2 Hz, 1H), 6.77 (d, *J* = 7.7 Hz, 1H), 6.72 (br s, 2H), 3.79 (s, 3H), 3.73–3.61 (m, 1H), 3.54–3.46 (m, 2H), 3.37–3.19 (m, 1H), 2.59 (br s, 1H), 1.99–1.92 (m, 3H), 1.85–1.63 (m, 3H), 1.49 (s, 9H); ^{13}**C NMR** (150.9 MHz, $CDCl_3$) δ (mixture of rotamers) 159.8, 155.9, 150.2, 150.1, 129.6, 129.5, 112.8, 112.6, 111.2, 111.0, 79.3, 55.3, 46.8, 46.7, 46.3, 45.9, 45.6, 45.3, 37.3, 36.9, 35.7, 34.6, 30.4, 28.7 (3C), 27.8, 27.6; **HRMS** (TOF MS ES+) *m/z*: $[M + Na]^+$ calculated for $C_{18}H_{27}NO_3Na$, 328.1889; found 328.1884.

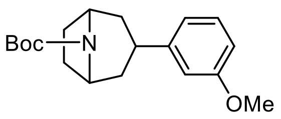

Arylated product **12** was prepared according to Method A. The following amounts of reagents were used: mesylate **SI-8** (31 mg, 0.10 mmol, 1.0 equiv), boronic acid (28 mg, 0.18 mmol, 1.8 equiv), $NiBr_2$(dme) (3.1 mg, 10. μmol, 10. mol %), Dtbbpy (2.7 mg, 10. μmol, 10. mol %), K_3PO_4 (35 mg, 0.18 mmol, 1.6 equiv), NaI (150 mg, 0.10 mmol, 10. equiv), H_2O (20. μL, 1.1 mmol, 11 equiv), and anhydrous *t*-BuOH (1.0 mL, 0.10 M in substrate). The residue was then purified by flash column chromatography (0–25% EtOAc/hexanes) to afford the title compound as a clear oil (22 mg, 67 μmol, 67%). **TLC R_f** = 0.7 (15% EtOAc/hexanes, $KMnO_4$ stain); **^{1}H NMR** (500 MHz, $CDCl_3$) δ (mixture of rotamers) 7.22 (t, *J* = 8.3 Hz, 1H), 6.79 (d, *J* = 7.6 Hz, 1H), 6.73 (br s, 2H), 4.35–4.24 (m, 2H), 3.79 (s, 3H), 3.08–3.04 (m, 1H), 2.02–1.66 (m, 8H), 1.49 (s, 9H); **^{13}C NMR** (150.9 MHz, $CDCl_3$) δ (mixture of rotamers) 159.8, 153.7.7, 147.1, 129.6, 119.6, 113.2, 111.6, 79.3, 55.2, 54.3, 53.6, 39.2, 37.8, 35.4, 30.4, 29.8 28.7 (3C); **HRMS** (TOF MS ES+) *m/z*: [M + Na]$^+$ calculated for $C_{19}H_{27}NO_3Na$, 340.1889; found 340.1884.

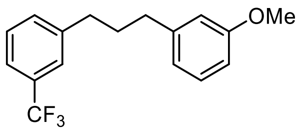

Arylated product **13** was prepared according to Method A. The following amounts of reagents were used: mesylate **SI-9** (28 mg, 0.10 mmol, 1.0 equiv), boronic acid (27 mg, 0.18 mmol, 1.8 equiv), $NiBr_2$(dme) (3.1 mg, 10. μmol, 10. mol %), Dtbbpy (2.7 mg, 10. μmol, 10. mol %), K_3PO_4 (34 mg, 0.16 mmol, 1.6 equiv), NaI (150 mg, 0.10 mmol, 10. equiv), H_2O (20. μL, 1.1 mmol, 11 equiv), and anhydrous *t*-BuOH (1.0 mL, 0.10 M in substrate). The residue was then purified by flash column chromatography (0–15% benzene/hexanes) to afford the title compound as a clear oil (25 mg, 81 μmol, 81%). **TLC R_f** = 0.4 (20% benzene/hexanes, CAM stain); **^{1}H NMR** (500 MHz, $CDCl_3$) δ 7.46–7.31 (m, 4H), 7.20 (t, *J* = 7.6 Hz, 1H), 6.80–6.70 (m, 3H), 3.79 (s, 3H), 2.70 (t, *J* = 7.6 Hz, 2H), 2.63 (t, *J* = 7.5 Hz, 2H), 1.97 (quint, *J* = 7.5 Hz, 2H). Analytical data is consistent with literature values.[6]

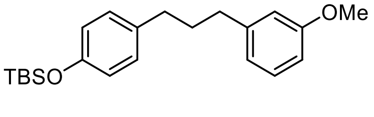

Arylated product **14** was prepared according to Method A. The following amounts of reagents were used: mesylate **SI-10** (35 mg, 0.10 mmol, 1.0 equiv), boronic acid (27 mg, 0.18 mmol, 1.8 equiv), $NiBr_2$(dme) (3.1 mg, 10. μmol, 10. mol %), Dtbbpy (2.7 mg, 10. μmol, 10. mol %), K_3PO_4 (34 mg, 0.16 mmol, 1.6 equiv), NaI (150 mg, 0.10 mmol, 10. equiv), H_2O (20. μL, 1.1 mmol, 11

equiv), and anhydrous *t*-BuOH (1.0 mL, 0.10 M in substrate). The residue was then purified by flash column chromatography (0–20% benzene/hexanes) to afford the title compound as a clear oil (25 mg, 71 μmol, 71%). **TLC R$_f$** = 0.4 (20% benzene/hexanes, CAM stain); **^{1}H NMR** (600 MHz, $CDCl_3$) δ 7.19 (at, *J* = 8.1 Hz, 1H), 7.02 (d, *J* = 8.4 Hz, 2H), 6.80–6.70 (m, 5H), 3.79 (s, 3H), 2.63–2.55 (m, 4H), 1.91 (quint, *J* = 8.1 Hz, 2H), 0.98 (s, 9H), 0.18 (s, 6H); **^{13}C NMR** (150.9 MHz, $CDCl_3$) δ 159.7, 153.6, 144.1, 135.0, 129.29 (2C), 129.27, 120.9, 119.9 (2C), 114.3, 111.0, 55.2, 35.5, 34.7, 33.1, 25.8 (3C), 18.3, –4.4 (2C); **HRMS** (TOF MS ES+) *m/z*: $[M + Na]^+$ calculated for $C_{22}H_{32}O_2SiNa$, 379.2069; found 379.2067.

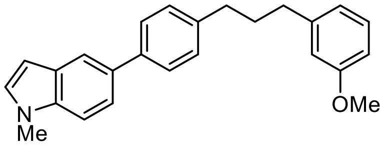

Arylated product **15** was prepared according to Method A. The following amounts of reagents were used: mesylate **SI-11** (36 mg, 0.10 mmol, 1.0 equiv), boronic acid (28 mg, 0.18 mmol, 1.8 equiv), $NiBr_2$(dme) (3.1 mg, 10. μmol, 10. mol %), Dtbbpy (2.7 mg, 10. μmol, 10. mol %), K_3PO_4 (35 mg, 0.16 mmol, 1.6 equiv), NaI (150 mg, 0.10 mmol, 10. equiv), H_2O (20. μL, 1.0 mmol, 11 equiv), and anhydrous *t*-BuOH (0.98 mL, 0.10 M in substrate). The residue was then purified by flash column chromatography (0–25% EtOAc/hexanes) to afford the title compound as a clear oil (36 mg, 60. μmol, 60%). **TLC R$_f$** = 0.5 (25% EtOAc/hexanes, CAM stain); **^{1}H NMR** (400 MHz, $CDCl_3$) δ 7.82 (br s, 1H), 7.58 (d, *J* = 9.9 Hz, 2H), 7.48 (d, *J* = 8.5 Hz, 1H), 7.37 (d, *J* = 8.5 Hz, 1H), 7.26–7.18 (m, 2H), 7.20–7.18 (m, 1H), 7.06 (d, *J* = 3.0 Hz, 1H), 6.81 (d, *J* = 7.6 Hz, 1H), 6.76–6.73 (m, 2H) 6.52 (d, *J* = 3.0 Hz, 1H), 3.79 (s, 6H), 2.71 (q, *J* = 8.4 Hz, 4H), 2.04–1.96 (m, 2H); **^{13}C NMR** (150.9 MHz, $CDCl_3$) δ 159.8, 144.2, 140.4, 140.2, 136.3, 132.9, 129.5, 129.4, 129.1, 128.9 (2C), 127.4 (2C), 121.4, 121.1, 119.3, 114.3, 111.2, 109.5, 101.4, 55.3, 35.7, 35.2, 33.1, 32.9; **HRMS** (TOF MS ES+) *m/z*: $[M + H]^+$ calculated for $C_{25}H_{26}NO$, 356.2014; found 356.2004.

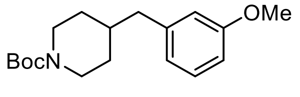

Arylated product **16** was prepared according to Method A. The following amounts of reagents were used: mesylate **SI-12** (30. mg, 0.10 mmol, 1.0 equiv), boronic acid (27 mg, 0.18 mmol, 1.8 equiv), $NiBr_2$(dme) (3.1 mg, 10. μmol, 10. mol %), Dtbbpy (2.7 mg, 10. μmol, 10. mol %), K_3PO_4 (34 mg, 0.16 mmol, 1.6 equiv), NaI (150 mg, 0.10 mmol, 10. equiv), H_2O (20. μL, 1.1 mmol, 11 equiv), and anhydrous *t*-BuOH (1.0 mL, 0.10 M in substrate). The residue was then purified by flash column chromatography (0–20% EtOAc/hexanes) to afford the title compound as a clear oil (23 mg, 74 μmol, 74%). **TLC R$_f$** = 0.6 (20% EtOAc/hexanes, PMA stain); **^{1}H NMR** (500 MHz,

$CDCl_3$) δ 7.19 (t, *J* = 7.8 Hz, 1H), 6.73 (at, *J* = 6.8 Hz, 2H), 6.68 (s, 1H), 4.06 (br s, 2H), 3.80 (s, 3H), 2.63 (at, *J* = 11.1 Hz, 2H), 2.50 (d, *J* = 7.0 Hz, 2H), 1.72–1.55 (m, 3H), 1.45 (s, 9H), 1.20–1.07 (m, 2H). Analytical data is consistent with literature values.[7]

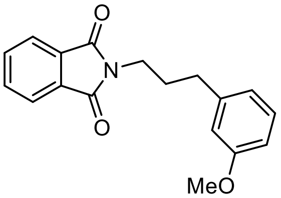

Arylated product **17** was prepared according to Method A. The following amounts of reagents were used: mesylate **SI-13** (29 mg, 0.10 mmol, 1.0 equiv), boronic acid (28 mg, 0.18 mmol, 1.8 equiv), $NiBr_2$(dme) (3.1 mg, 10. µmol, 10. mol %), Dtbbpy (2.7 mg, 10. µmol, 10. mol %), K_3PO_4 (35 mg, 0.16 mmol, 1.6 equiv), NaI (150 mg, 1.0 mmol, 10. equiv), H_2O (20. µL, 1.1 mmol, 11 equiv), and anhydrous *t*-BuOH (1.0 mL, 0.10 M in substrate). The residue was then purified by flash column chromatography (0–25% EtOAc/hexanes) to afford the title compound as a clear oil (30. mg, 54 µmol, 53%). **TLC R_f** = 0.4 (25% EtOAc/hexanes, CAM stain); **^{1}H NMR** (500 MHz, $CDCl_3$) δ 7.83–7.81 (m, 2H), 7.70–7.68 (m, 2H), 7.16 (t, *J* = 15.8 Hz, 1H), 6.78 (d, *J* = 7.6 Hz, 1H), 6.74 (s, 1H), 6.79 (dd, *J* = 8.3, 1.9 Hz, 1H), 3.78 (s, 3H), 3.76 (t, *J* = 7.2 Hz, 2H), 2.68 (t, *J* = 7.6 Hz, 2H), 2.07–2.00 (m, 2H). Analytical data is consistent with literature values.[8]

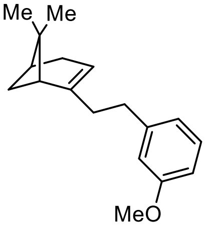

Arylated product **18** was prepared according to Method A. The following amounts of reagents were used: mesylate **SI-14** (26 mg, 0.10 mmol, 1.0 equiv), boronic acid (28 mg, 0.18 mmol, 1.8 equiv), $NiBr_2$(dme) (3.1 mg, 10. µmol, 10. mol %), Dtbbpy (2.7 mg, 10. µmol, 10. mol %), K_3PO_4 (35 mg, 0.16 mmol, 1.6 equiv), NaI (150 mg, 1.0 mmol, 10. equiv), H_2O (20. µL, 1.0 mmol, 11 equiv), and anhydrous *t*-BuOH (1.0 mL, 0.10 M in substrate). The residue was then purified by flash column chromatography (0–20% benzene/hexanes) to afford the title compound as a clear oil (26 mg, 73 µmol, 72%). **TLC R_f** = 0.5 (20% benzene/hexanes, CAM stain); **^{1}H NMR** (500 MHz, $CDCl_3$) δ 7.19 (t, *J* = 8.0 Hz, 1H), 6.78 (d, *J* = 7.1 Hz, 1H), 6.74–6.71 (m, 2H), 5.24–5.23 (m, 1H) 3.79 (s, 3H), 2.65 (t, *J* = 7.9 Hz, 2H), 2.39–2.25 (m, 1H), 2.26–2.17 (m, 4H), 2.08–2.07 (m, 2H), 1.29 (s, 3H), 1.17 (d, *J* = 8.3 Hz, 1H), 0.85 (s, 3H); **^{13}C NMR** (125.4 MHz, $CDCl_3$) δ 159.7, 148.0, 144.4, 129.3, 120.9, 116.3, 114.2, 111.1, 55.3, 46.1, 41.0, 38.8, 38.1, 34.1, 31.8, 31.4, 26.5, 21.4**; HRMS** (TOF MS ES+) *m/z*: $[M]^+$ calculated for $C_{18}H_{24}O$, 244.1313; found 256.1832.

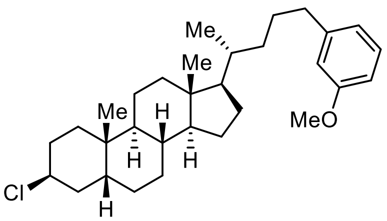

Arylated product **19** was prepared according to Method A. The following amounts of reagents were used: mesylate **SI-15** (46 mg, 0.10 mmol, 1.0 equiv), boronic acid (28 mg, 0.18 mmol, 1.8 equiv), $NiBr_2$(dme) (3.1 mg, 10. µmol, 10. mol %), Dtbbpy (2.7 mg, 10. µmol, 10. mol %), K_3PO_4 (34 mg, 0.16 mmol, 1.6 equiv), NaI (150 mg, 0.10 mmol, 10. equiv), H_2O (20. µL, 1.1 mmol, 11 equiv), and anhydrous *t*-BuOH (1.0 mL, 0.10 M in substrate). The residue was then purified by flash column chromatography (0–20% benzene/hexanes) to afford the title compound as a clear oil (32 mg, 68 µmol, 68%). **TLC R_f** = 0.6 (20% benzene/hexanes, CAM stain); **^{1}H NMR** (600 MHz, $CDCl_3$) δ 7.18 (t, *J* = 7.7 Hz, 1H), 6.77 (d, *J* = 7.5 Hz, 1H), 6.75–6.70 (m, 2H), 4.59–4.55 (m, 1H), 3.79 (s, 3H), 2.58 (ddd, *J* = 15.0, 9.6, 6.0 Hz, 1H), 2.49 (ddd, *J* = 14.0, 9.7, 6.3 Hz, 1H), 2.22 (ddd, *J* = 15.3, 12.8, 3.3 Hz, 1H), 1.97 (ad, *J* = 12.6 Hz, 1H), 1.94–1.74 (m, 4H), 1.73–1.61 (m, 2H), 1.61–1.32 (m, 10H), 1.31–0.86 (m, 16H), 0.64 (s, 3H); **^{13}C NMR** (150.9 MHz, $CDCl_3$) δ 159.6, 144.7, 129.2, 120.9, 114.2, 110.8, 61.7, 56.7, 56.3, 55.2, 42.8, 40.5, 40.3, 36.7, 36.6, 35.8, 35.74, 35.67, 35.2, 34.6, 30.1, 29.0, 28.3, 28.0, 26.6, 26.4, 24.2, 23.8, 21.0, 18.7, 12.1; **HRMS** (TOF MS EI+) *m/z*: $[M]^+$ calculated for $C_{31}H_{47}ClO$, 470.33154; found 470.33124.

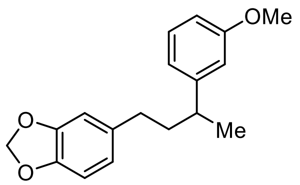

Arylated product **20** was prepared according to Method A. The following amounts of reagents were used: mesylate **(±)54** (30. mg, 0.11 mmol, 1.0 equiv), boronic acid (30. mg, 0.19 mmol, 1.8 equiv), $NiBr_2$(dme) (3.4 mg, 11 µmol, 10. mol %), Dtbbpy (2.9 mg, 11 µmol, 10. mol %), K_3PO_4 (37 mg, 0.18 mmol, 1.6 equiv), NaI (160 mg, 1.1 mmol, 10. equiv), H_2O (22 µL, 1.2 mmol, 11 equiv), and anhydrous *t*-BuOH (1.1 mL, 0.10 M in substrate). The residue was then purified by flash column chromatography (0–25% benzene/hexanes) to afford the title compound as a clear oil (20. mg, 70. µmol, 70%). **TLC R_f** = 0.3 (25% benzene/hexanes, CAM stain); **^{1}H NMR** (500 MHz, $CDCl_3$) δ 7.25–7.20 (m, 1H), 6.80 (ad, *J* = 7.5 Hz, 1H), 6.75–6.74 (m, 2H), 6.71–6.69 (m, 1H), 6.62 (s, 1H), 6.57 (ad, *J* = 7.9 Hz, 1H), 5.90 (ad, *J* = 1.3 Hz, 2H), 3.81 (s, 3H), 2.70–2.26 (m, 1H), 2.44 (t, *J* = 7.2 Hz, 2H), 1.90–1.80 (m, 2H), 1.26 (d, *J* = 8.1 Hz, 3H). Analytical data is consistent with literature values.[9]

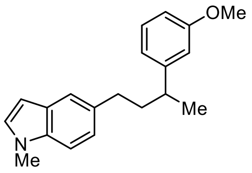

Arylated product **21** was prepared according to Method A. The following amounts of reagents were used: mesylate **SI-16** (28 mg, 0.10 mmol, 1.0 equiv), boronic acid (27 mg, 0.18 mmol, 1.8 equiv), $NiBr_2$(dme) (3.1 mg, 10. μmol, 10. mol %), Dtbbpy (2.7 mg, 10. μmol, 10. mol %), K_3PO_4 (34 mg, 0.16 mmol, 1.6 equiv), NaI (150 mg, 1.0 mmol, 10. equiv), H_2O (20. μL, 1.1 mmol, 11 equiv), and anhydrous *t*-BuOH (1.0 mL, 0.10 M in substrate). The residue was then purified by flash column chromatography (0–30% benzene/hexanes) to afford the title compound as a yellow oil (22 mg, 74 μmol, 74%). **TLC** $\mathbf{R_f}$ = 0.3 (20% benzene/hexanes, CAM stain); ^{1}**H NMR** (600 MHz, $CDCl_3$) δ 7.37 (s, 1H), 7.23 (d, *J* = 7.6 Hz, 1H), 7.22 (d, *J* = 7.0 Hz, 1H), 7.01 (d, *J* = 10.6 Hz, 1H), 7.00 (s, 1H), 6.83 (d, *J* = 7.2 Hz, 1H), 6.78 (s, 1H), 6.75 (d, *J* = 7.9 Hz, 1H), 6.39 (s, 1H), 3.81 (s, 3H), 3.75 (s, 3H), 2.72 (sextet, *J* = 6.5 Hz, 1H), 2.62 (at, *J* = 7.1 Hz, 2H), 2.01–1.91 (m, 2H), 1.26 (d, *J* = 6.5 Hz, 3H); ^{13}**C NMR** (150.9 MHz, $CDCl_3$) δ 159.6, 149.4, 135.3, 133.3, 129.3, 128.8, 128.6, 122.5, 119.9, 119.6, 113.1, 110.9, 108.9, 100.4, 55.1, 40.7, 39.5, 33.9, 32.8, 22.5; **HRMS** (TOF MS ES+) *m/z*: $[M + H]^+$ calculated for $C_{20}H_{24}NO$, 294.1858; found 294.1867.

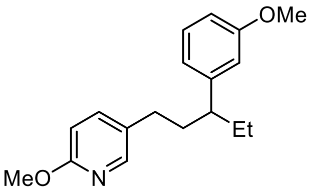

Arylated product **22** was prepared according to Method A. The following amounts of reagents were used: mesylate **SI-17** (28 mg, 0.10 mmol, 1.0 equiv), boronic acid (28 mg, 0.18 mmol, 1.8 equiv), $NiBr_2$(dme) (3.1 mg, 10. μmol, 10. mol %), Dtbbpy (2.7 mg, 10. μmol, 10. mol %), K_3PO_4 (35 mg, 0.16 mmol, 1.6 equiv), NaI (150 mg, 0.10 mmol, 10. equiv), H_2O (20. μL, 1.0 mmol, 11 equiv), and anhydrous *t*-BuOH (1.0 mL, 0.10 M in substrate). The residue was then purified by flash column chromatography (10% EtOAc/hexanes) to afford the title compound as a clear oil (19 mg, 65 μmol, 64%). **TLC** $\mathbf{R_f}$ = 0.5 (25% EtOAc/hexanes, CAM stain); ^{1}**H NMR** (500 MHz, $CDCl_3$) δ 7.89 (br s, 1H), 7.33 (dd, *J* = 8.6, 3.2 Hz, 1H), 7.25 (t, *J* = 7.9 Hz, 1H), 6.76 (d, *J* = 8.0 Hz, 2H), 6.70 (br s, 1H), 6.66 (d, *J* = 13.4 Hz, 1H), 3.85 (s, 3H), 3.81 (s, 3H), 2.42–2.32 (m, 3H), 1.89–1.79 (m, 2H), 1.69–1.53 (m, 2H), 0.79 (t, *J* = 7.4 Hz, 3H); ^{13}**C NMR** (150.9 MHz, $CDCl_3$) δ 162.7, 159.8, 147.0, 146.1, 139.0, 130.5, 129.4, 120.5, 113.9, 111.0, 110.4, 55.3, 53.4, 47.4, 38.1, 30.0, 29.9 12.2; **HRMS** (TOF MS ES+) *m/z*: $[M + H]^+$ calculated for $C_{18}H_{24}NO_2$, 286.1807; found 286.1808.

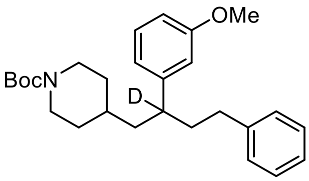

Arylated product **D-23** was prepared according to Method A. The following amounts of reagents were used: mesylate **D-SI-18** (40. mg, 0.10 mmol, 1.0 equiv), boronic acid (27 mg, 0.18 mmol, 1.8 equiv), $NiBr_2$(dme) (3.0 mg, 10. µmol, 10. mol %), Dtbbpy (2.6 mg, 10. µmol, 10. mol %), K_3PO_4 (33 mg, 0.16 mmol, 1.6 equiv), NaI (150 mg, 0.10 mmol, 10. equiv), H_2O (20. µL, 1.0 mmol, 11 equiv), and anhydrous *t*-BuOH (0.97 mL, 0.10 M in substrate). The residue was then purified by flash column chromatography (0–10% EtOAc/hexanes) to afford the title compound as a yellow oil (23 mg, 56 µmol, 56%). **TLC R_f** = 0.7 (10% EtOAc/hexanes, $KMnO_4$ stain; **^{1}H NMR** (400 MHz, $CDCl_3$) δ 7.26–7.21 (m, 3H), 7.18–7.13 (m, 1H), 7.10 (ad, *J* = 7.2 Hz, 2H), 6.77 (d, *J* = 8.0 Hz, 2H), 6.71 (s, 1H), 3.98 (br s, 2H), 3.81 (s, 3H), 2.53 (br s, 2H), 2.45 (t, *J* = 8.0 Hz, 2H), 1.90–1.81 (m, 2H), 1.69–1.56 (m, 3H), 1.43 (br s, 9H), 1.23–1.16 (m, 2H), 1.09–0.96 (m, 2H); **^{13}C NMR** (150.9 MHz, $CDCl_3$) δ 159.8, 154.9, 147.1, 142.5, 129.5, 129.4, 128.5 (2C), 128. 4 (2C), 125.8, 125.6, 120.3, 113.9, 113.8, 110.9, 79.3, 71.6, 55.3, 43.8, 39.1, 33.8, 33.4, 30.4, 28.6 (3C); **HRMS** (TOF MS ES+) *m/z*: $[M + Na]^+$ calculated for $C_{27}H_{36}DNO_3Na$, 447.2735; found 447.2749.

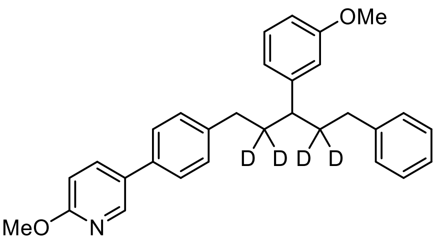

Arylated product **D_4-24** was prepared according to Method A. The following amounts of reagents were used: mesylate **D_4-SI-19** (42 mg, 0.10 mmol, 1.0 equiv), boronic acid (27 mg, 0.18 mmol, 1.8 equiv), $NiBr_2$(dme) (3.1 mg, 10. µmol, 10. mol %), Dtbbpy (2.7 mg, 10. µmol, 10. mol %), K_3PO_4 (34 mg, 0.16 mmol, 1.6 equiv), NaI (150 mg, 0.10 mmol, 10. equiv), H_2O (20. µL, 1.0 mmol, 11 equiv), and anhydrous *t*-BuOH (0.98 mL, 0.10 M in substrate). The residue was then purified by flash column chromatography (0–10% EtOAc/hexanes) to afford the title compound as a yellow oil (24 mg, 55 µmol, 55%). **TLC R_f** = 0.5 (10% EtOAc/hexanes, CAM stain); **^{1}H NMR** (500 MHz, $CDCl_3$) δ 8.36 (br s, 1H), 7.78 (ad, *J* = 8.6 Hz, 1H), 7.41 (d, *J* = 7.5 Hz, 2H), 7.28–7.26 (m, 3H), 7.17 (d, *J* = 7.4 Hz, 3H), 7.10 (d, *J* = 7.4 Hz, 2H), 6.82–6.76 (m, 4H), 3.97 (s, 3H), 3.82 (br s, 3H), 2.55 (s, 1H), 2.48 (d, *J* = 11.6 Hz, 4H); **^{13}C NMR** (150.9 MHz, $CDCl_3$) δ 163.5, 159.8, 146.9, 144.8, 142.5, 141.8, 137.4, 135.3, 130.1, 129.5, 129.1 (2C), 128.4 (2C), 128.3 (2C), 126.6 (2C), 125.7, 120.4, 113.9, 111.1, 110.8, 55.2, 53.6, 44.9, 33.6, 33.3, carbons bearing

deuterium labels are not visible; **HRMS** (TOF MS ES+) *m/z*: $[M + H]^+$ calculated for $C_{30}H_{27}D_4NO_2H$, 442.2688; found 442.2680.

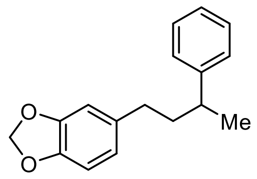

Arylated product **25** was prepared according to Method A. The following amounts of reagents were used: mesylate **(±)54** (31 mg, 0.11 mmol, 1.0 equiv), boronic acid (25 mg, 0.21 mmol, 1.8 equiv), $NiBr_2$(dme) (3.5 mg, 11 μmol, 10. mol %), Dtbbpy (3.1 mg, 11 μmol, 10. mol %), K_3PO_4 (39 mg, 0.18 mmol, 1.6 equiv), NaI (170 mg, 1.1 mmol, 10. equiv), H_2O (20. μL, 1.0 mmol, 11 equiv) and anhydrous *t*-BuOH (1.1 mL, 0.10 M in substrate). The residue was then purified by flash column chromatography (0–20% benzene/hexanes) to afford the title compound as a clear oil (22 mg, 55 μmol, 76%). **TLC** $\mathbf{R_f}$ = 0.6 (20% benzene/hexanes, CAM stain); ^{1}**H NMR** (500 MHz, $CDCl_3$) δ 7.31–7.29 (m, 2H), 7.20–7.18 (m, 3H), 6.71 (d, *J* = 7.9 Hz, 1H), 6.62 (s, 1H), 6.57 (d, *J* = 7.8 Hz, 1H), 5.89 (s, 2H), 2.71 (sextet, *J* = 6.9 Hz, 1H), 2.44–2.38 (m, 2H), 1.94–1.79 (m, 2H), 1.26 (d, *J* = 6.9 Hz, 3H). Analytical data is consistent with literature values.[10]

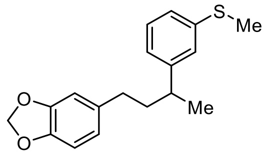

Arylated product **26** was prepared according to Method A. The following amounts of reagents were used: mesylate **(±)54** (27 mg, 98 μmol, 1.0 equiv), boronic acid (30. mg, 0.18 mmol, 1.8 equiv), $NiBr_2$(dme) (3.0 mg, 10. μmol, 10. mol %), Dtbbpy (2.6 mg, 10. μmol, 10. mol %), K_3PO_4 (33 mg, 0.16 mmol, 1.6 equiv), NaI (150 mg, 0.98 mmol, 10. equiv), H_2O (20. μL, 1.1 mmol, 11 equiv), and anhydrous *t*-BuOH (0.98 mL, 0.10 M in substrate). The residue was then purified by flash column chromatography (0–25% benzene/hexanes) to afford the title compound as a clear oil (20. mg, 66 μmol, 68%). **TLC** $\mathbf{R_f}$ = 0.4 (20% benzene/hexanes, CAM stain); ^{1}**H NMR** (500 MHz, $CDCl_3$) δ 7.23 (t, *J* = 7.8 Hz, 1H), 7.10 (d, *J* = 6.5 Hz, 1H), 7.09 (s, 1H), 6.97 (d, *J* = 7.8 Hz, 1H), 6.70 (d, *J* = 7.8 Hz, 1H), 6.62 (s, 1H), 6.56 (d, *J* = 7.8 Hz, 1H), 5.90 (s, 2H), 2.66 (sextet, *J* = 7.0 Hz, 1H), 2.48 (s, 3H), 2.43 (t, *J* = 8.0 Hz, 2H), 1.91–1.79 (m, 2H), 1.25 (d, *J* = 6.9 Hz, 3H); ^{13}**C NMR** (150.9 MHz, $CDCl_3$) δ 146.9, 146.5, 144.5, 137.2, 135.2, 127.9, 124.5, 123.1, 122.9, 120.0, 107.8, 107.0, 99.7, 39.0, 38.3, 32.6, 21.4, 14.9; **HRMS** (TOF MS EI+) *m/z*: $[M]^+$ calculated for $C_{18}H_{20}O_2S$, 300.1184; found 300.1191.

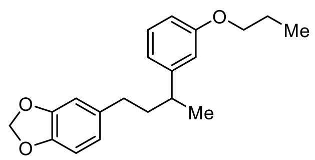

Arylated product **27** was prepared according to Method A. The following amounts of reagents were used: mesylate **(±)54** (27 mg, 0.10 mmol, 1.0 equiv), boronic acid (32 mg, 0.18 mmol, 1.8 equiv), $NiBr_2$(dme) (3.1 mg, 10. µmol, 10. mol %), Dtbbpy (2.7 mg, 10. µmol, 10. mol %), K_3PO_4 (35 mg, 0.16 mmol, 1.6 equiv), NaI (150 mg, 1.0 mmol, 10. equiv), H_2O (20. µL, 1.1 mmol, 11 equiv), and anhydrous *t*-BuOH (1.0 mL, 0.10 M in substrate). The residue was then purified by flash column chromatography (0–1% EtOAc/hexanes) to afford the title compound as a clear oil (22 mg, 70. µmol, 70%). **TLC R_f** = 0.5 (2% EtOAc/hexanes, CAM stain); **^{1}H NMR** (500 MHz, $CDCl_3$) δ 7.20 (t, *J* = 7.3 Hz, 1H), 6.77 (d, *J* = 7.6 Hz, 1H), 6.74 (s, 1H), 6.73 (d, *J* = 8.9 Hz, 1H), 6.70 (d, *J* = 7.8 Hz, 1H), 6.63 (s, 1H), 6.57 (d, *J* = 8.0 Hz, 1H), 5.90 (s, 2H), 3.92 (t, *J* = 6.5 Hz, 2H), 2.67 (sextet, *J* = 7.0 Hz, 1H), 2.43 (t, *J* = 7.1 Hz, 2H), 1.91–1.78 (m, 4H), 1.25 (d, *J* = 6.8 Hz, 3H), 1.04 (t, *J* = 7.4 Hz, 3H); **^{13}C NMR** (150.9 MHz, $CDCl_3$) δ 159.3, 148.9, 147.5, 145.4, 136.4, 129.3, 121.0, 119.4, 113.6, 111.5, 108.8, 108.0, 100.7, 69.4, 40.1, 39.4, 33.6, 22.7, 22.5, 10.6; **HRMS** (TOF MS EI+) *m/z*: $[M]^+$ calculated for $C_{20}H_{24}O_3$, 312.1725; found 312.1716.

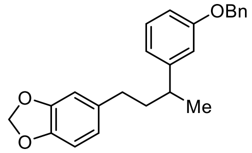

Arylated product **28** was prepared according to Method A. The following amounts of reagents were used: mesylate **(±)54** (28 mg, 0.10 mmol, 1.0 equiv), boronic acid (42 mg, 0.18 mmol, 1.8 equiv), $NiBr_2$(dme) (3.2 mg, 10. µmol, 10. mol %), Dtbbpy (2.8 mg, 10. µmol, 10. mol %), K_3PO_4 (35 mg, 0.16 mmol, 1.6 equiv), NaI (150 mg, 1.0 mmol, 10. equiv), H_2O (20. µL, 1.1 mmol, 11 equiv), and anhydrous *t*-BuOH (1.0 mL, 0.10 M in substrate). The residue was then purified by flash column chromatography (0–5% EtOAc/hexanes) to afford the title compound as a clear oil (26 mg, 73 µmol, 72%). **TLC R_f** = 0.3 (10% benzene/hexanes, CAM stain); **^{1}H NMR** (500 MHz, $CDCl_3$) δ 7.44 (d, *J* = 7.2 Hz, 2H), 7.38 (t, *J* = 7.0 Hz, 2H), 7.32 (d, *J* = 7.1 Hz, 1H), 7.23 (d, *J* = 9.8 Hz, 1H), 6.82 (s, 1H), 6.80 (t, *J* = 7.0 Hz, 2H), 6.69 (d, *J* = 7.8 Hz, 1H), 6.61 (s, 1H), 6.55 (d, *J* = 7.8 Hz, 1H), 5.89 (s, 2H), 5.05 (s, 2H), 2.67 (sextet, *J* = 7.0, 1H), 2.41 (t, *J* = 7.1 Hz, 2H), 1.90–1.77 (m, 2H), 1.24 (d, *J* = 6.9 Hz, 3H); **^{13}C NMR** (125.3 MHz, $CDCl_3$) δ 157.9, 148.0, 146.4, 144.4, 136.1, 135.3, 128.3, 127.5 (2C), 126.9, 126.5 (2C), 120.0, 118.8, 113.0, 110.9, 107.8, 107.0, 99.6, 68.9, 38.1, 38.4, 32.6, 21.4; **HRMS** (TOF MS EI+) *m/z*: $[M]^+$ calculated for $C_{24}H_{24}O_3$, 360.1725; found 360.1718.

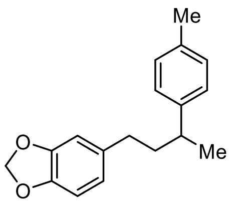

Arylated product **29** was prepared according to Method A. The following amounts of reagents were used: mesylate **(±)54** (28 mg, 0.10 mmol, 1.0 equiv), boronic acid (25 mg, 0.18 mmol, 1.8 equiv), $NiBr_2$(dme) (3.1 mg, 10. μmol, 10. mol %), Dtbbpy (2.7 mg, 10. μmol, 10. mol %), K_3PO_4 (34 mg, 0.18 mmol, 1.6 equiv), NaI (150 mg, 1.0 mmol, 10. equiv), H_2O (20. μL, 1.1 mmol, 11 equiv) and anhydrous *t*-BuOH (1.0 mL, 0.10 M in substrate). The residue was then purified by flash column chromatography (0–20% benzene/hexanes) to afford the title compound as a clear oil (19 mg, 71 μmol, 70%). **TLC R_f** = 0.4 (20% benzene/hexanes, CAM stain); **^{1}H NMR** (500 MHz, $CDCl_3$) δ 7.12 (q, *J* = 7.9 Hz, 4H), 6.71 (d, *J* = 7.9 Hz, 1H), 6.62 (s, 1H), 6.57 (d, *J* = 7.8 Hz, 1H), 5.89 (s, 2H), 2.71 (sextet, *J* = 6.9 Hz, 1H), 2.44 (t, *J* = 7.7 Hz, 2H), 2.32 (s, 3H), 1.90–1.78 (m, 2H), 1.25 (d, *J* = 6.9 Hz, 3H); **^{13}C NMR** (125.3 MHz, $CDCl_3$) δ 147.6, 145.6, 144.3, 136.6, 135.5, 129.2 (2C), 127.1 (2C), 121.2, 108.9, 108.2, 100.8, 40.4, 39.1, 33.8, 22.8, 21.1; **HRMS** (TOF MS EI+) *m/z*: $[M]^+$ calculated for $C_{18}H_{20}O_2$, 268.1463; found 268.1465.

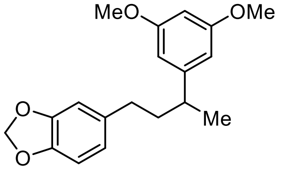

Arylated product **30** was prepared according to Method A. The following amounts of reagents were used: mesylate **(±)54** (27 mg, 0.10 mmol, 1.0 equiv), boronic acid (33 mg, 0.18 mmol, 1.8 equiv), $NiBr_2$(dme) (3.1 mg, 10. μmol, 10. mol %), Dtbbpy (2.7 mg, 10. μmol, 10. mol %), K_3PO_4 (34 mg, 0.18 mmol, 1.6 equiv), NaI (150 mg, 1.0 mmol, 10. equiv), H_2O (20. μL, 1.1 mmol, 11 equiv) and anhydrous *t*-BuOH (1.0 mL, 0.10 M in substrate). The residue was then purified by flash column chromatography (0–5% EtOAc/hexanes) to afford the title compound as a clear oil (21 mg, 67 μmol, 67%). **TLC R_f** = 0.4 (10% EtOAc/hexanes, CAM stain); **^{1}H NMR** (500 MHz, $CDCl_3$) δ 6.71 (d, *J* = 7.9 Hz, 1H), 6.63 (s, 1H), 6.57 (d, *J* = 7.8 Hz, 1H), 6.36 (s, 2H), 6.31 (s, 1H), 5.90 (s, 2H), 3.79 (s, 6H), 2.64–2.61 (m, 1H), 2.45 (t, *J* = 7.7 Hz, 2H), 1.86–1.81 (m, 2H), 1.24 (d, *J* = 6.9 Hz, 3H); **^{13}C NMR** (150.9 MHz, $CDCl_3$) δ 160.9 (2C), 149.9, 147.6, 145.6, 136.5, 121.2, 108.9, 108.2, 105.4 (2C), 108.8, 97.7, 55.4 (2C), 40.1, 39.8, 33.7, 22.5; **HRMS** (TOF MS ES+) *m/z*: $[M + H]^+$ calculated for $C_{19}H_{23}O_4$, 315.1596; found 315.1600.

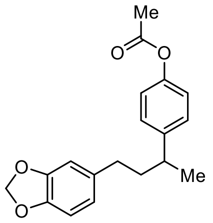

Arylated product **31** was prepared according to Method A. The following amounts of reagents were used: mesylate **(±)54** (27 mg, 0.10 mmol, 1.0 equiv), boronic acid (32 mg, 0.18 mmol, 1.8 equiv), $NiBr_2$(dme) (3.1 mg, 10. µmol, 10. mol %), Dtbbpy (2.7 mg, 10. µmol, 10. mol %), K_3PO_4 (34 mg, 0.16 mmol, 1.6 equiv), NaI (150 mg, 1.0 mmol, 10. equiv), H_2O (20. µL, 1.1 mmol, 11 equiv), and anhydrous *t*-BuOH (1.0 mL, 0.10 M in substrate). The residue was then purified by flash column chromatography (0–5% EtOAc/hexanes) to afford the title compound as a clear oil (17 mg, 56 µmol, 56%). **TLC R_f** = 0.3 (5% EtOAc/hexanes, CAM stain); **^{1}H NMR** (500 MHz, $CDCl_3$) δ 7.18 (d, *J* = 7.7 Hz, 2H), 7.02 (d, *J* = 7.7 Hz, 2H), 6.69 (d, *J* = 7.9 Hz, 1H), 6.62 (s, 1H), 6.56 (d, *J* = 7.8 Hz, 1H), 5.90 (s, 2H), 2.70 (sextet, *J* = 6.8 Hz, 1H), 2.43 (at, *J* = 7.1 Hz, 2H), 2.29 (s, 3H), 1.90–1.79 (m, 2H), 1.25 (d, *J* = 7.0 Hz, 3H); **^{13}C NMR** (125.3 MHz, $CDCl_3$) δ 169.7, 148.8, 147.6, 145.5, 144.8, 136.3, 128.0, 121.4, 121.1, 108.9, 108.1, 100.8, 40.3, 38.8, 33.6, 30.4, 29.8, 22.5, 21.2; **HRMS** (TOF MS ES+) *m/z*: $[M + Na]^+$ calculated for $C_{19}H_{20}O_4Na$, 335.1259; found 335.1267.

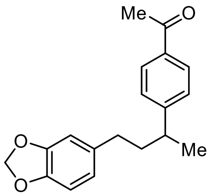

Arylated product **32** was prepared according to Method A. The following amounts of reagents were used: mesylate **(±)54** (28 mg, 0.10 mmol, 1.0 equiv), boronic acid (30. mg, 0.19 mmol, 1.8 equiv), $NiBr_2$(dme) (3.2 mg, 10. µmol, 10. mol %), Dtbbpy (2.8 mg, 10. µmol, 10. mol %), K_3PO_4 (35 mg, 0.18 mmol, 1.6 equiv), NaI (150 mg, 1.0 mmol, 10. equiv), H_2O (20. µL, 1.1 mmol, 11 equiv) and anhydrous *t*-BuOH (1.0 mL, 0.10 M in substrate). The residue was then purified by flash column chromatography (0–10% EtOAc/hexanes) to afford the title compound as a clear oil (19 mg, 64 µmol, 63%). **TLC R_f** = 0.3 (5% EtOAc/hexanes, CAM stain); **^{1}H NMR** (500 MHz, $CDCl_3$) δ 7.92 (d, *J* = 8.2 Hz, 2H), 7.29 (d, *J* = 8.3 Hz, 2H), 6.71 (d, *J* = 7.9 Hz, 1H), 6.60 (s, 1H), 6.55 (d, *J* = 7.8 Hz, 1H), 5.90 (s, 2H), 2.81 (sextet, *J* = 6.9 Hz, 1H), 2.59 (s, 3H), 2.44–2.40 (m, 2H), 1.90–1.86 (m, 2H), 1.28 (d, *J* = 6.9 Hz, 3H); **^{13}C NMR** (150.9 MHz, $CDCl_3$) δ 197.9, 153.1, 147.6, 145.6, 135.9, 135.4, 128.7 (2C), 127.3 (2C), 121.1, 108.8, 108.2, 100.8, 39.9, 39.4, 33.6, 26.6, 22.3; **HRMS** (TOF MS EI+) *m/z*: $[M]^+$ calculated for $C_{19}H_{20}O_3$, 296.1412; found 296.1417.

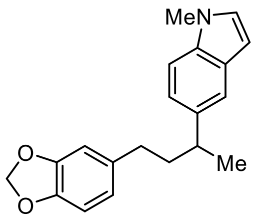

Arylated product **33** was prepared according to Method A. The following amounts of reagents were used: mesylate **(±)54** (29 mg, 0.11 mmol, 1.0 equiv), boronic acid (33 mg, 0.19 mmol, 1.8 equiv), $NiBr_2$(dme) (3.3 mg, 11 μmol, 10. mol %), Dtbbpy (2.8 mg, 11 μmol, 10. mol %), K_3PO_4 (36 mg, 0.17 mmol, 1.6 equiv), NaI (160 mg, 1.1 mmol, 10. equiv), H_2O (20. μL, 1.1 mmol, 11 equiv) and anhydrous *t*-BuOH (1.1 mL, 0.10 M in substrate). The residue was then purified by flash column chromatography (0–50% benzene/hexanes) to afford the title compound as a clear oil (12 mg, 39 μmol, 37%). **TLC R_f** = 0.2 (25% benzene/hexanes, CAM stain); **^{1}H NMR** (400 MHz, $CDCl_3$) δ 7.44 (d, *J* = 1.6 Hz, 1H), 7.28 (d, *J* = 8.4 Hz, 1H), 7.09 (dd, *J* = 8.5, 1.6 Hz, 1H), 7.02 (d, *J* = 3.0 Hz, 1H), 6.71 (d, *J* = 7.9 Hz, 1H), 6.63 (d, *J* = 1.6 Hz, 1H), 6.57–6.56 (m, 1H), 6.44 (dd, J = 3.1, 0.8 Hz, 1H), 5.90 (s, 2H), 3.78 (s, 3H), 2.84 (sextet, *J* = 6.9 Hz, 1H), 2.45–2.42 (m, 2H), 1.96–1.84 (m, 2H), 1.31 (d, *J* = 6.9 Hz, 3H); **^{13}C NMR** (150.9 MHz, $CDCl_3$) δ 147.5, 145.5, 138.2, 136.9, 135.6, 129.1, 128.7, 121.19, 121.17, 118.9, 109.2, 109.3, 108.1, 100.8, 100.7, 40.9, 39.6, 33.9, 32.9, 23.5; **HRMS** (TOF MS ES+) *m/z*: $[M + H]^+$ calculated for $C_{20}H_{22}NO_2$, 308.1659; found 308.1653.

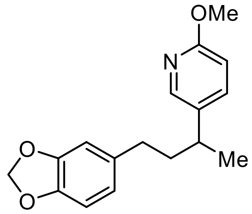

Arylated product **34** was prepared according to Method B. The following amounts of reagents were used: mesylate **(±)54** (30. mg, 0.11 mmol, 1.0 equiv), boronic acid (35 mg, 0.23 mmol, 2.1 equiv), $NiBr_2$(dme) (3.3 mg, 11 μmol, 10. mol %), Dtbbpy (2.9 mg, 11 μmol, 10. mol %), K_3PO_4 (37 mg, 0.18 mmol, 1.6 equiv), NaI (240 mg, 1.6 mmol, 15 equiv), H_2O (20. μL, 1.1 mmol, 11 equiv) and anhydrous *t*-BuOH (1.1 mL, 0.10 M in substrate). The residue was then purified by flash column chromatography (0–15% EtOAc/hexanes) to afford the title compound as a clear oil (17 mg, 61 μmol, 56%). TLC R_r = 0.7 (25% EtOAc/hexanes, CAM stain); **^{1}H NMR** (600 MHz, $CDCl_3$) δ 7.97 (d, *J* = 2.4 Hz, 1H), 7.43 (dd, *J* = 8.5, 2.5 Hz, 1H), 6.72–6.69 (m, 2H), 6.61 (d, *J* = 1.4 Hz, 1H), 6.55–6.54 (m, 1H), 5.91 (s, 2H), 3.93 (s, 3H), 2.68 (sextet, *J* = 6.8 Hz, 1H), 2.44 (t, *J* = 7.8 Hz, 2H), 1.87–1.79 (m, 2H), 1.24 (d, *J* = 6.9 Hz, 3H); **^{13}C NMR** (150.9 MHz, $CDCl_3$) δ 162.9, 147.7, 145.7, 145.5, 137.3, 136.1, 134.9, 121.2, 110.8, 108.9, 108.2, 100.9, 53.4, 40.1, 36.0, 33.6, 22.6; **HRMS** (TOF MS ES+) *m/z*: $[M + Na]^+$ calculated for $C_{17}H_{19}NO_3Na$, 308.1263; found 308.1256.

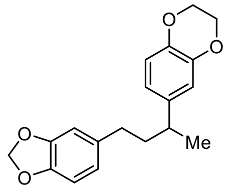

Arylated product **35** was prepared according to Method A. The following amounts of reagents were used: mesylate **(±)54** (29 mg, 0.11 mmol, 1.0 equiv), boronic acid (34 mg, 0.19 mmol, 1.8 equiv), $NiBr_2$(dme) (3.3 mg, 11 μmol, 10. mol %), Dtbbpy (2.8 mg, 11 μmol, 10. mol %), K_3PO_4 (36 mg, 0.17 mmol, 1.6 equiv), NaI (160 mg, 1.1 mmol, 10. equiv), H_2O (20. μL, 1.1 mmol, 11 equiv) and anhydrous *t*-BuOH (1.1 mL, 0.10 M in substrate). The residue was then purified by flash column chromatography (0–10% EtOAc/hexanes) to afford the title compound as a clear oil (18 mg, 58 μmol, 55%). **TLC R_f** = 0.2 (5% EtOAc/hexanes, CAM stain); **^{1}H NMR** (600 MHz, $CDCl_3$) δ 6.79 (ad, *J* = 8.2 Hz, 1H), 6.99–6.68 (m, 2H), 6.66 (dd, *J* = 8.2, 1.9 Hz, 1H), 6.62 (br s, 1H), 6.55 (d, *J* = 7.9 Hz, 1H), 5.89 (s, 2H), 4.24–4.40 (m, 4H), 2.61 (sextet, *J* = 6.8 Hz, 1H), 2.43 (t, *J* = 7.9 Hz, 2H) 1.84–1.74 (m, 2H), 1.21 (d, *J* = 6.9 Hz, 3H); **^{13}C NMR** (150.9 MHz, $CDCl_3$) δ 147.6, 145.5, 143.4, 141.8, 140.8, 136.6, 121.2, 120.1, 117.1, 115.6, 108.9, 108.2, 100.8, 64.6, 64.5, 40.4, 38.8, 33.7, 22.7; **HRMS** (TOF MS EI+) *m/z*: $[M]^+$ calculated for $C_{19}H_{20}O_4$, 312.1362; found 312.1353.

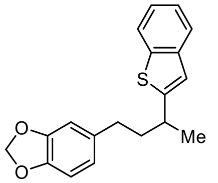

Arylated product **36** was prepared according to Method B. The following amounts of reagents were used: mesylate **(±)54** (29 mg, 0.11 mmol, 1.0 equiv), boronic acid (40. mg, 0.22 mmol, 2.1 equiv), $NiBr_2$(dme) (3.3 mg, 11 μmol, 10. mol %), Dtbbpy (2.9 mg, 11 μmol, 10. mol %), K_3PO_4 (36 mg, 0.17 mmol, 1.6 equiv), NaI (240 mg, 1.6 mmol, 15 equiv), H_2O (21 μL, 1.2 mmol, 11 equiv), and anhydrous *t*-BuOH (1.0 mL, 0.10 M in substrate). The residue was then purified by flash column chromatography (0–5% EtOAc/hexanes) to afford the title compound as a yellow oil (13 mg, 42 μmol, 38%). **TLC R_f** = 0.4 (5% benzene/hexanes, CAM stain); **^{1}H NMR** (500 MHz, $CDCl_3$) δ 7.78 (d, *J* = 7.9 Hz, 1H), 7.67 (d, *J* = 7.9 Hz, 1H), 7.31 (t, *J* = 7.3 Hz, 1H), 7.26 (t, *J* = 7.7 Hz, 1H), 7.03 (s, 1H), 6.72 (d, *J* = 7.8 Hz, 1H), 6.66 (s, 1H), 6.60 (d, *J* = 7.8 Hz, 1H), 5.91 (s, 2H), 3.10 (sextet, *J* = 6.9 Hz, 1H), 2.59–2.49 (m, 2H), 2.01–1.90 (m, 2H), 1.40 (d, *J* = 6.9 Hz, 3H); **^{13}C NMR** (150.9 MHz, $CDCl_3$) δ 152.4, 147.5, 145.6, 140.0, 138.9, 135.9, 124.1, 123.4, 122.8, 122.3, 121.1, 119.5, 108.9, 108.1, 100.7, 40.7, 35.6, 33.3, 23.1; **HRMS** (TOF MS EI+) *m/z*: $[M]^+$ calculated for $C_{19}H_{18}O_2S$, 310.1028; found 310.1035.

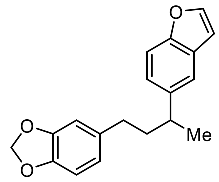

Arylated product **37** was prepared according to Method B. The following amounts of reagents were used: mesylate **(±)54** (30. mg, 0.11 mmol, 1.0 equiv), boronic acid (38 mg, 0.23 mmol, 2.1 equiv), $NiBr_2$(dme) (3.4 mg, 11 µmol, 10. mol %), Dtbbpy (3.0 mg, 11 µmol, 10. mol %), K_3PO_4 (38 mg, 0.18 mmol, 1.6 equiv), NaI (250 mg, 1.6 mmol, 15 equiv), H_2O (22 µL, 1.2 mmol, 11 equiv), and anhydrous *t*-BuOH (1.1 mL, 0.10 M in substrate). The residue was then purified by flash column chromatography (0–25% benzene/hexanes) to afford the title compound as a yellow oil (19 mg, 65 µmol, 58%). **TLC R$_f$** = 0.5 (10% benzene/hexanes, CAM stain); **^{1}H NMR** (500 MHz, $CDCl_3$) δ 7.60 (d, *J* = 2.0 Hz, 1H), 7.43 (d, *J* = 8.4 Hz, 1H), 7.40 (ad, *J* = 1.1 Hz, 1H), 7.13 (dd, *J* = 8.5, 1.4 Hz, 1H), 6.72 (ad, *J* = 1.2 Hz, 1H), 6.70 (d, *J* = 7.9 Hz, 1H), 6.62 (ap s, 1H), 6.56 (d, *J* = 2.9 Hz, 1H), 5.90 (s, 2H), 2.80 (sextet, *J* = 7.1 Hz, 1H), 2.48–2.38 (m, 2H), 1.96–1.84 (m, 2H), 1.30 (d, *J* = 7.0 Hz, 3H); **^{13}C NMR** (150.9 MHz, $CDCl_3$) δ 153.6, 147.4, 145.4, 145.1, 141.8, 136.4, 127.5, 123.5, 121.0, 119.1, 111.1, 108.8, 108.0, 106.5, 100.7, 40.7, 39.3, 33.7, 23.1; **HRMS** (TOF MS EI+) *m/z*: $[M]^+$ calculated for $C_{19}H_{18}O_3$, 294.1256; found 294.1259.

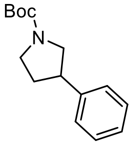

Arylated product **38** was prepared according to Method A. The following amounts of reagents were used: mesylate **SI-2** (27 mg, 0.10 mmol, 1.0 equiv), boronic acid (22 mg, 0.18 mmol, 1.8 equiv), $NiBr_2$(dme) (3.1 mg, 10. µmol, 10. mol %), Dtbbpy (2.7 mg, 10. µmol, 10. mol %), K_3PO_4 (34 mg, 0.16 mmol, 1.6 equiv), NaI (150 mg, 1.0 mmol, 10 equiv), H_2O (20. µL, 1.1 mmol, 11 equiv), and anhydrous *t*-BuOH (1.0 mL, 0.10 M in substrate). The residue was then purified by flash column chromatography (0–10%/10% EtOAc/benzene/hexanes) to afford the title compound as a clear oil (16 mg, 65 µmol, 65%). **TLC R$_f$** = 0.5 (5%/10% EtOAc/benzene/hexanes, $KMnO_4$ stain); **^{1}H NMR** (500 MHz, $CDCl_3$) δ (mixture of rotamers) 7.32 (t, *J* = 7.3 Hz, 2H), 7.24 (ad, *J* = 7.3 Hz, 3H), 3.88–3.76 (m, 1H), 3.67–3.54 (m, 1H), 3.44–3.25 (m, 3H), 2.27–2.25 (m, 1H), 1.98 (aquint, *J* = 9.8 Hz, 1H), 1.49 & 1.47 (s, 9H). Analytical data is consistent with literature values.[2]

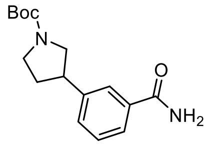

Arylated product **39** was prepared according to Method A. The following amounts of reagents were used: mesylate **SI-2** (30. mg, 0.11 mmol, 1.0 equiv), boronic acid (33 mg, 0.20 mmol, 1.8 equiv), $NiBr_2$(dme) (3.5 mg, 11 μmol, 10. mol %), Dtbbpy (3.0 mg, 11 μmol, 10. mol %), K_3PO_4 (38 mg, 0.18 mmol, 1.6 equiv), NaI (170 mg, 1.1 mmol, 10. equiv), H_2O (22 μL, 1.2 mmol, 11 equiv), and anhydrous *t*-BuOH (1.1 mL, 0.10 M in substrate). The residue was then purified by flash column chromatography (0–50% EtOAc/Et_2O) to afford the title compound as a clear oil (14 mg, 49 μmol, 43%). **TLC R_f** = 0.4 (50% EtOAc/Et_2O, $KMnO_4$ stain); **^{1}H NMR** (600 MHz, $CDCl_3$) δ (mixture of rotamers) 7.75 (ad, *J* = 10.2 Hz, 1H), 7.65 (br s, 1H), 7.40 (br s, 2H), 6.25 (br s, 1H), 5.97 (br s, 1H), 3.90–3.79 (m, 1H), 3.67–3.55 (m, 1H), 3.44–3.28 (m, 3H), 2.31–2.25 (m, 1H), 2.01 (aquint, *J* = 9.5 Hz, 1H), 1.49 & 1.47 (s, 9H); **^{13}C NMR** (150.9 MHz, $CDCl_3$) δ (mixture of rotamers) 169.4, 154.5, 142.2, 133.7, 130.7, 128.9, 126.5, 126.4, 125.6, 125.52, 125.46, 79.4, 52.4, 51.7, 45.9, 45.6, 44.1, 43.2, 30.3, 28.5 (3C); **HRMS** (TOF MS ES+) *m/z*: $[M + Na]^+$ calculated for $C_{16}H_{22}N_2O_3Na$, 313.1528; found 313.1530.

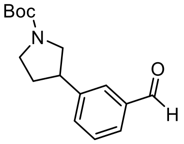

Arylated product **40** was prepared according to Method A. The following amounts of reagents were used: mesylate **SI-2** (27 mg, 0.10 mmol, 1.0 equiv), boronic acid (27 mg, 0.18 mmol, 1.8 equiv), $NiBr_2$(dme) (3.1 mg, 10. μmol, 10. mol %), Dtbbpy (2.7 mg, 10. μmol, 10. mol %), K_3PO_4 (35 mg, 0.16 mmol, 1.6 equiv), NaI (150 mg, 1.0 mmol, 10. equiv), H_2O (20. μL, 1.1 mmol, 11 equiv), and anhydrous *t*-BuOH (1.0 mL, 0.10 M in substrate). The residue was then purified by flash column chromatography (0–20% EtOAc/Et_2O) to afford the title compound as a clear oil (20. mg, 74 μmol, 73%). **TLC R_f** = 0.5 (30% EtOAc/Et_2O, $KMnO_4$ stain); **^{1}H NMR** (500 MHz, $CDCl_3$) δ 10.01 (s, 1H), 7.77 (ap s, 2H), 7.51 (ap s, 2H), 3.90–3.82 (m, 1H), 3.68–3.59 (m, 1H), 3.44–3.29 (m, 3H), 2.36–2.26 (m, 1H), 2.02 (aquint, *J* = 10.2 Hz, 1H), 1.49 (s, 9H); **^{13}C NMR** (150.9 MHz, $CDCl_3$) δ (mixture of rotamers) 192.3, 154.5, 136.7, 133.3, 129.3, 128.8, 128.6, 128.0, 127.8, 79.61, 79.57, 52.3, 51.7, 45.9, 45.6, 43.9, 43.0, 33.2, 32.4, 28.5 (3C); **HRMS** (TOF MS ES+) *m/z*: $[M + Na]^+$ calculated for $C_{16}H_{21}NO_3Na$, 298.1419; found 298.1417.

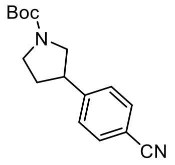

Arylated product **41** was prepared according to Method A. The following amounts of reagents were used: mesylate **SI-2** (29 mg, 0.11 mmol, 1.0 equiv), boronic acid (34 mg, 0.23 mmol, 1.8 equiv), $NiBr_2$(dme) (3.3 mg, 11 μmol, 10. mol %), Dtbbpy (2.9 mg, 11 μmol, 10. mol %), K_3PO_4 (37 mg, 0.17 mmol, 1.6 equiv), NaI (160 mg, 1.1 mmol, 10. equiv), H_2O (21 μL, 1.2 mmol, 11 equiv), and anhydrous *t*-BuOH (1.1 mL, 0.10 M in substrate). The residue was then purified by flash column chromatography (0–20% EtOAc/hexanes) to afford the title compound as a clear oil (19 mg, 70. μmol, 64%). **TLC R_f** = 0.2 (10% EtOAc/hexanes, $KMnO_4$ stain); **^{1}H NMR** (500 MHz, $CDCl_3$) δ 7.62 (d, *J* = 7.8 Hz, 2H), 7.34 (d, *J* = 7.2 Hz, 2H), 3.86–3.79 (m, 1H), 3.65–3.56 (m, 1H), 3.42–3.27 (m, 3H), 2.30 (ap s, 1H), 1.97 (aquint, *J* = 9.2 Hz, 1H), 1.48 (s, 9H); **^{13}C NMR** (150.9 MHz, $CDCl_3$) δ (mixture of rotamers) 154.5, 154.4, 147.2, 147.1, 132.5 (2C), 127.9 (2C), 118.8, 116.4, 79.7, 52.1, 51.4, 45.7, 45.5, 44.3, 43.3, 33.1, 32.2, 30.3, 29.7, 28.5 (3C); **HRMS** (TOF MS ES+) *m/z*: $[M + Na]^+$ calculated for $C_{16}H_{20}N_2O_2Na$, 295.1422; found 295.1422.

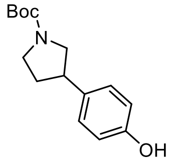

Arylated product **42** was prepared according to Method A. The following amounts of reagents were used: mesylate **SI-2** (28 mg, 0.10 mmol, 1.0 equiv), boronic acid (26 mg, 0.19 mmol, 1.8 equiv), $NiBr_2$(dme) (3.2 mg, 10. μmol, 10. mol %), Dtbbpy (2.8 mg, 10. μmol, 10. mol %), K_3PO_4 (35 mg, 0.17 mmol, 1.6 equiv), NaI (160 mg, 1.0 mmol, 10. equiv), H_2O (20. μL, 1.2 mmol, 11 equiv), and anhydrous *t*-BuOH (1.0 mL, 0.10 M in substrate). The residue was then purified by flash column chromatography (0–20% EtOAc/hexanes) to afford the title compound as a clear oil (11 mg, 41 μmol, 39%). **TLC R_f** = 0.3 (15% EtOAc/hexanes, $KMnO_4$ stain); **^{1}H NMR** (500 MHz, $CDCl_3$) δ (mixture of rotamers) 7.08 (d, *J* = 8.0 Hz, 1H), 7.05 (d, *J* = 8.0 Hz, 1H), 6.81 (t, *J* = 7.7 Hz, 2H), 3.88–3.72 (m, 1H), 3.63–3.53 (m, 1H), 3.43–3.33 (m, 1H), 3.26–3.20 (m, 2H), 2.24–2.18 (m, 1H), 1.97–1.91 (m, 1H), 1.74 (br s, 1H), 1.49 & 1.48 (s, 9H); **^{13}C NMR** (150.9 MHz, $CDCl_3$) δ (mixture of rotamers) 154.9, 154.8, 133.0, 132.9, 128.1, 128.0, 125.5, 115.50, 115.46, 79.6, 52.8, 52.2, 46.1, 45.6, 43.4, 42.6, 33.4, 32.6, 30.3, 29.7, 28.6 (3C); **HRMS** (TOF MS ES+) *m/z*: $[M + Na]^+$ calculated for $C_{15}H_{21}NO_3Na$, 286.1419; found 286.1422.

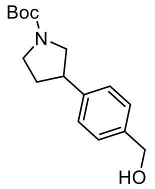

Arylated product **43** was prepared according to Method A. The following amounts of reagents were used: mesylate **SI-2** (28 mg, 0.11 mmol, 1.0 equiv), boronic acid (29 mg, 0.19 mmol, 1.8 equiv), $NiBr_2$(dme) (3.3 mg, 11 µmol, 10. mol %), Dtbbpy (2.8 mg, 11 µmol, 10. mol %), K_3PO_4 (36 mg, 0.16 mmol, 1.6 equiv), NaI (160 mg, 1.1 mmol, 10. equiv), H_2O (21 µL, 1.2 mmol, 11 equiv), and anhydrous *t*-BuOH (1.0 mL, 0.10 M in substrate). The residue was then purified by flash column chromatography (0–30% EtOAc/hexanes) to afford the title compound as a clear oil (12 mg, 44 µmol, 41%). **TLC R_f** = 0.2 (20% EtOAc/hexanes, $KMnO_4$ stain); **^{1}H NMR** (500 MHz, $CDCl_3$) δ (mixture of rotamers) 7.33 (d, *J* = 7.6 Hz, 2H), 7.23 (d, *J* = 7.3 Hz, 2H), 4.67 (s, 2H), 3.86–3.75 (m, 1H), 3.65–3.53 (m, 1H), 3.43–3.24 (m, 3H), 2.27–2.23 (m, 1H), 1.97 (aquint, *J* = 9.3 Hz, 1H), 1.84 (br s, 1H), 1.48 & 1.47 (s, 9H); **^{13}C NMR** (150.9 MHz, $CDCl_3$) δ (mixture of rotamers) 154.6, 140.9, 139.5, 139.4, 127.33 (2C), 127.27 (2C), 79.3, 65.0, 52.6, 51.8, 45.9, 45.6, 44.0, 43.1, 33.4, 32.5, 30.3, 29.7, 29.4, 28.6 (3C); **HRMS** (TOF MS ES+) *m/z*: $[M + Na]^+$ calculated for $C_{16}H_{23}NO_3Na$, 300.1576; found 300.1577.

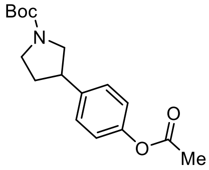

Arylated product **44** was prepared according to Method A. The following amounts of reagents were used: mesylate **SI-2** (30. mg, 0.11 mmol, 1.0 equiv), boronic acid (37 mg, 0.20 mmol, 1.8 equiv), $NiBr_2$(dme) (3.5 mg, 11 µmol, 10. mol %), Dtbbpy (3.0 mg, 11 µmol, 10. mol %), K_3PO_4 (38 mg, 0.18 mmol, 1.6 equiv), NaI (170 mg, 1.1 mmol, 10. equiv), H_2O (22 µL, 1.3 mmol, 11 equiv), and anhydrous *t*-BuOH (1.1 mL, 0.10 M in substrate). The residue was then purified by flash column chromatography (0–20% EtOAc/hexanes) to afford the title compound as a clear oil (26 mg, 84 µmol, 74%). **TLC R_f** = 0.2 (10% EtOAc/hexanes, CAM stain); **^{1}H NMR** (500 MHz, $CDCl_3$) δ 7.24 (d, *J* = 7.9 Hz, 2H), 7.03 (d, *J* = 8.4 Hz, 2H), 3.87–3.75 (m, 1H), 3.66–3.53 (m, 1H), 3.42–3.23 (m, 3H), 2.29 (s, 3H), 2.27–2.23 (m, 1H), 1.95 (aquint, *J* = 9.6 Hz, 1H), 1.47 (s, 9H); **^{13}C NMR** (150.9 MHz, $CDCl_3$) δ (mixture of rotamers) 169.7, 154.6, 149.40, 149.37, 139.1, 128.1 (2C), 121.7 (2C), 79.40, 79.36, 52.7, 51.8, 45.9, 45.6, 43.8, 42.9, 33.5, 32.5, 30.4, 29.7, 28.6 (3C); **HRMS** (TOF MS ES+) *m/z*: $[M + Na]^+$ calculated for $C_{17}H_{23}NO_4Na$, 328.1525; found 328.1539.

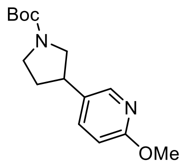

Arylated product **45** was prepared according to Method B. The following amounts of reagents were used: mesylate **SI-2** (27 mg, 0.10 mmol, 1.0 equiv), boronic acid (33 mg, 0.22 mmol, 2.1 equiv), $NiBr_2$(dme) (3.2 mg, 10. µmol, 10. mol %), Dtbbpy (2.8 mg, 10. µmol, 10. mol %), K_3PO_4 (35 mg, 0.17 mmol, 1.6 equiv), NaI (230 mg, 1.5 mmol, 15 equiv), H_2O (20. µL, 1.1 mmol, 11 equiv), and anhydrous *t*-BuOH (1.0 mL, 0.10 M in substrate). The residue was then purified by flash column chromatography (0–20% EtOAc/hexanes) to afford the title compound as a yellow oil (15 mg, 54 µmol, 52%). **TLC R_f** = 0.4 (20% EtOAc/hexanes, $KMnO_4$ stain); **^{1}H NMR** (500 MHz, $CDCl_3$) δ 8.04 (d, *J* = 1.7 Hz, 1H), 7.45 (d, *J* = 8.3 Hz, 1H), 6.72 (d, *J* = 8.6 Hz, 1H), 3.92 (s, 3H), 3.87–3.74 (m, 1H), 3.65–3.54 (m, 1H), 3.44–3.19 (m, 3H), 2.23 (ap s, 1H), 1.93 (aquint, *J* = 9.7 Hz, 1H), 1.47 (s, 9H); **^{13}C NMR** (150.9 MHz, $CDCl_3$) δ (mixture of rotamers) 163.3, 154.5, 145.3, 145.2, 137.3, 137.2, 129.5, 129.4, 125.5, 110.9, 79.4, 53.4, 52.4, 51.8, 45.8, 45.5, 41.1, 40.2, 33.2, 32.4, 30.3, 29.7, 28.5 (3C); **HRMS** (TOF MS ES+) *m/z*: $[M + Na]^+$ calculated for $C_{15}H_{22}N_2O_3Na$, 301.1528; found 301.1532.

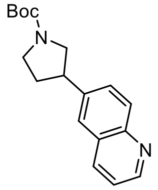

Arylated product **46** was prepared according to Method B. The following amounts of reagents were used: mesylate **SI-2** (25 mg, 0.10 mmol, 1.0 equiv), boronic acid (35 mg, 0.20 mmol, 2.1 equiv), $NiBr_2$(dme) (2.9 mg, 10. µmol, 10. mol %), Dtbbpy (2.6 mg, 10. µmol, 10. mol %), K_3PO_4 (32 mg, 0.15 mmol, 1.6 equiv), NaI (210 mg, 1.4 mmol, 15 equiv), H_2O (19 µL, 1.1 mmol, 11 equiv), and anhydrous *t*-BuOH (0.95 mL, 0.10 M in substrate). The residue was then purified by flash column chromatography (0–40% EtOAc/hexanes) to afford the title compound as a yellow oil (21 mg, 72 µmol, 75%). **TLC R_f** = 0.2 (30% EtOAc/hexanes, $KMnO_4$ stain); **^{1}H NMR** (500 MHz, $CDCl_3$) δ (mixture of rotamers) 8.89 (d, *J* = 3.1 Hz, 1H), 8.12 (d, *J* = 8.1 Hz, 1H), 8.07 (d, *J* = 8.6 Hz, 1H), 7.64 (s, 1H), 7.62 (d, *J* = 9.0 Hz, 1H), 7.40 (dd, *J* = 8.0, 4.0 Hz, 1H), 3.95–3.85 (m, 1H), 3.72–3.35 (m, 4H), 2.35 (ap s, 1H), 2.09 (tt, J = 11.9, 9.5 Hz, 1H), 1.50 & 1.49 (s, 9H); **^{13}C NMR** (150.9 MHz, $CDCl_3$) δ (mixture of rotamers) 154.6, 154.5, 150.2, 147.4, 139.93, 139.89, 135.8, 129.7, 129.3, 128.2, 125.1, 121.4, 79.4, 52.4, 51.7, 45.9, 45.6, 44.2, 43.3, 33.4, 32.4, 30.3, 29.7, 28.6 (3C); **HRMS** (TOF MS ES+) *m/z*: $[M + Na]^+$ calculated for $C_{18}H_{22}N_2O_2Na$, 299.1760; found 299.1756.

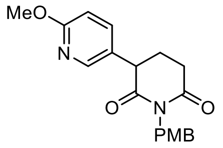

Arylated product **47** was prepared according to Method B The following amounts of reagents were used: mesylate **55** (36 mg, 0.11 mmol, 1.0 equiv), boronic acid (35 mg, 0.23 mmol, 2.1 equiv), $NiBr_2$(dme) (3.4 mg, 11 µmol, 10. mol %), Dtbbpy (2.9 mg, 11 µmol, 10. mol %), K_3PO_4 (37 mg, 0.17 mmol, 1.6 equiv), NaI (240 mg, 1.6 mmol, 15 equiv), H_2O (21 µL, 1.2 mmol, 11 equiv), and anhydrous *t*-BuOH (1.0 mL, 0.10 M in substrate). The residue was then purified by flash column chromatography (0–45% EtOAc/hexanes) to afford the title compound as a dark yellow oil (23 mg, 68 µmol, 63%). **TLC** $\mathbf{R_f}$ = 0.2 (35% EtOAc/hexanes, $KMnO_4$ stain); **^{1}H NMR** (500 MHz, $CDCl_3$) δ 7.97 (d, *J* = 1.8 Hz, 1H), 7.36 (d, *J* = 8.5 Hz, 2H), 7.33 (dd, *J* = 8.8, 2.4 Hz, 1H), 6.82 (d, *J* = 8.5 Hz, 2H), 6.73 (d, *J* = 8.6 Hz, 1H), 4.93 (q, *J* = 13.6 Hz, 2H), 3.92 (s, 3H), 3.78 (s, 3H), 3.71 (dd, *J* = 10.8, 5.2 Hz, 1H), 2.87–2.82 (m, 1H), 2.75–2.63 (m, 1H), 2.27–2.09 (m, 2H); **^{13}C NMR** (150.9 MHz, $CDCl_3$) δ 172.9, 171.8, 163.6, 159.0, 146.3, 138.3, 130.7 (2C), 129.4, 126.5, 113.7 (2C), 111.1, 55.2, 53.5, 45.9, 42.8, 32.2, 25.2; **HRMS** (TOF MS ES+) *m/z*: $[M + Na]^+$ calculated for $C_{19}H_{20}N_2O_4Na$, 363.1321; found 363.1320.

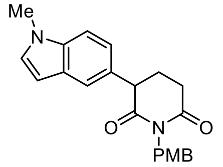

Arylated product **48** was prepared according to Method B. The following amounts of reagents were used: mesylate **55** (32 mg, 0.10 mmol, 1.0 equiv), boronic acid (36 mg, 0.21 mmol, 2.1 equiv), $NiBr_2$(dme) (3.1 mg, 10. µmol, 10. mol %), Dtbbpy (2.7 mg, 10. µmol, 10. mol %), K_3PO_4 (34 mg, 0.16 mmol, 1.6 equiv), NaI (220 mg, 1.5 mmol, 15 equiv), H_2O (19 µL, 1.1 mmol, 11 equiv), and anhydrous *t*-BuOH (0.99 mL, 0.10 M in substrate). The residue was then purified by flash column chromatography (0–50% EtOAc/hexanes) to afford the title compound as a pink oil (17 mg, 46 µmol, 46%). **TLC** $\mathbf{R_f}$ = 0.3 (35% EtOAc/hexanes, $KMnO_4$ stain); **^{1}H NMR** (500 MHz, $CDCl_3$) δ 7.40 (d, *J* = 8.7 Hz, 2H), 7.28 (ad, *J* = 3.2 Hz, 1H), 7.26 (ad, *J* = 5.1 Hz, 1H), 7.04 (d, *J* = 2.9 Hz, 1H), 6.95 (dd, *J* = 8.3, 1.1 Hz, 1H), 6.83 (d, *J* = 8.6 Hz, 2H), 6.38 (d, *J* = 2.8 Hz, 1H), 4.99 (d, *J* = 5.5 Hz, 2H), 3.79 (s, 3H), 3.77 (s, 3H), 2.80–2.74 (m, 1H), 2.70–2.63 (m, 1H), 2.29–2.18 (m, 3H); **^{13}C NMR** (150.9 MHz, $CDCl_3$) δ 174.1, 172.5, 158.9, 135.9, 130.8 (2C), 129.8, 129.5, 128.8, 128.6, 121.4, 120.0, 113.7 (2C), 109.6, 100.9, 55.2, 48.6, 42.5, 32.9, 31.4, 25.7; **HRMS** (TOF MS ES+) *m/z*: $[M + Na]^+$ calculated for $C_{22}H_{22}N_2O_3$, 385.1528; found 385.1534.

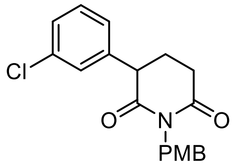

Arylated product **49** was prepared according to Method A. The following amounts of reagents were used: mesylate **55** (32 mg, 0.10 mmol, 1.0 equiv), boronic acid (28 mg, 0.18 mmol, 1.8 equiv), $NiBr_2$(dme) (3.0 mg, 10. µmol, 10. mol %), Dtbbpy (2.6 mg, 10. µmol, 10. mol %), K_3PO_4 (33 mg, 0.18 mmol, 1.6 equiv), NaI (150 mg, 0.10 mmol, 10. equiv), H_2O (20. µL, 1.1 mmol, 11 equiv), and anhydrous *t*-BuOH (1.0 mL, 0.10 M in substrate). The residue was then purified by flash column chromatography (0–30% EtOAc/hexanes) to afford the title compound as a clear oil (30. mg, 86 µmol, 83%). **TLC R_f** = 0.3 (25% EtOAc/hexanes, $KMnO_4$ stain); **^{1}H NMR** (600 MHz, $CDCl_3$) δ 7.37 (d, *J* = 8.6 Hz, 2H), 7.27–7.26 (m, 2H), 7.10 (s, 1H), 7.00–6.98 (m, 1H), 6.83 (d, *J* = 8.7 Hz, 2H), 4.97–4.92 (m, 2H), 3.78 (s, 3H), 3.78–3.75 (m, 1H), 2.79–2.65 (m, 2H), 2.24–2.11 (m, 2H); **^{13}C NMR** (150.9 MHz, $CDCl_3$) δ 172.8, 171.9, 159.1, 140.0, 134.7, 130.8 (2C), 130.2, 129.5, 128.4, 127.9, 126.4, 113.9 (2C), 55.3, 48.5, 42.8, 31.8, 25.3; **HRMS** (TOF MS ES+) *m/z*: $[M + Na]^+$ calculated for $C_{19}H_{18}ClNO_3Na$, 366.0873; found 366.0871.

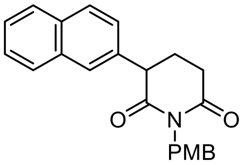

Arylated product **50** was prepared according to Method A. The following amounts of reagents were used: mesylate **55** (35 mg, 0.10 mmol, 1.0 equiv), boronic acid (29 mg, 0.19 mmol, 1.8 equiv), $NiBr_2$(dme) (3.3 mg, 11 µmol, 10. mol %), Dtbbpy (2.8 mg, 11 µmol, 10. mol %), K_3PO_4 (36 mg, 0.17 mmol, 1.6 equiv), NaI (160 mg, 1.1 mmol, 10. equiv), H_2O (21 µL, 1.1 mmol, 11 equiv), and anhydrous *t*-BuOH (1.1 mL, 0.10 M in substrate). The residue was then purified by flash column chromatography (0–30% EtOAc/hexanes) to afford the title compound as a clear oil (31 mg, 84 µmol, 79%). **TLC R_f** = 0.3 (25% EtOAc/hexanes, $KMnO_4$ stain); **^{1}H NMR** (600 MHz, $CDCl_3$) δ 7.82–7.79 (m, 2H), 7.67–7.65 (m, 1H), 7.46–7.45 (m, 3H), 7.43 (d, *J* = 8.6 Hz, 2H), 7.25–7.22 (m, 1H), 6.86 (d, *J* = 8.5 Hz, 2H), 5.03–4.98 (m, 2H), 3.99–3.97 (m, 1H), 3.79 (s, 3H), 2.78–2.67 (m, 2H), 2.29–2.25 (m, 2H); **^{13}C NMR** (150.9 MHz, $CDCl_3$) δ 173.4, 172.3, 159.2, 135.5, 133.4, 132.7, 130.9 (2C), 129.7, 128.8, 127.9, 127.7, 126.9, 126.5, 126.3, 125.9, 133.9 (2C). 55.4, 48.8, 42.7, 31.5 25.2; **HRMS** (TOF MS ES+) *m/z*: $[M + Na]^+$ calculated for $C_{23}H_{21}NO_3Na$, 382.1419; found 382.1419.

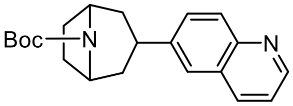

Arylated product **51** was prepared according to Method B. The following amounts of reagents were used: mesylate **SI-8** (32 mg, 0.11 mmol, 1.0 equiv), boronic acid (38 mg, 0.22 mmol, 2.1 equiv), $NiBr_2$(dme) (3.3 mg, 11 μmol, 10. mol %), Dtbbpy (2.8 mg, 11 μmol, 10. mol %), K_3PO_4 (36 mg, 0.17 mmol, 1.6 equiv), NaI (240 mg, 1.6 mmol, 15 equiv), H_2O (21 mL, 1.2 mmol, 11 equiv), and anhydrous *t*-BuOH (1.0 mL, 0.10 M in substrate). The residue was then purified by flash column chromatography (0–50% EtOAc/hexanes) to afford the title compound as a yellow oil (14 mg, 42 μmol, 40%). **TLC** $\mathbf{R_f}$ = 0.3 (30% EtOAc/hexanes, $KMnO_4$ stain); ^{1}**H NMR** (600 MHz, $CDCl_3$) δ 8.87 (d, *J* = 2.9 Hz, 1H), 8.11 (d, *J* = 8.0 Hz, 1H), 8.06 (d, *J* = 8.7 Hz, 1H), 7.61 (ad, *J* = 1.6 Hz, 1H), 7.58 (dd, *J* = 8.7, 1.8 Hz, 1H), 7.39 (dd, *J* = 8.2, 4.2 Hz, 1H), 4.42 (br s, 1H), 4.31 (br s, 1H), 3.35–3.27 (m, 1H), 2.15–2.04 (m, 3H), 1.93–1.80 (m, 5H), 1.52 (s, 9H); ^{13}**C NMR** (150.9 MHz, $CDCl_3$) δ (mixture of rotamers) 153.6, 149.8, 147.1, 143.6, 136.0, 129.7, 129.4, 128.4, 125.0, 121.2, 79.3 (2C), 65.9, 65.3, 54.1, 53.4, 39.0, 37.6, 35.2 (2C), 28.6 (3C), 27.9, 15.3; **HRMS** (TOF MS ES+) *m/z*: $[M + H]^+$ calculated for $C_{21}H_{27}N_2O_2$, 339.2072; found 339.2059.

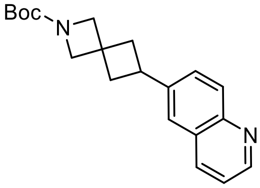

Arylated product **52** was prepared according to Method B. The following amounts of reagents were used: mesylate **SI-1** (30 mg, 0.10 mmol, 1.0 equiv), boronic acid (38 mg, 0.21 mmol, 2.1 equiv), $NiBr_2$(dme) (3.2 mg, 10. μmol, 10. mol %), Dtbbpy (2.8 mg, 10. μmol, 10. mol %), K_3PO_4 (35 mg, 0.17 mmol, 1.6 equiv), NaI (230 mg, 1.6 mmol, 15 equiv), H_2O (20. mL, 1.2 mmol, 11 equiv), and anhydrous *t*-BuOH (1.0 mL, 0.10 M in substrate). The residue was then purified by flash column chromatography (0–50% EtOAc/hexanes) to afford the title compound as a yellow oil (16 mg, 49 μmol, 48%) with a small amount of mesylate **SI-1** (2.0 mg, 8.0 μmol, 7%). **TLC** $\mathbf{R_f}$ = 0.2 (30% EtOAc/hexanes, $KMnO_4$ stain); ^{1}**H NMR** (500 MHz, $CDCl_3$) δ 8.87 (s, 1H), 8.11 (d, *J* = 8.2 Hz, 1H), 8.05 (d, *J* = 9.1 Hz, 1H), 7.55 (at, *J* = 4.0 Hz, 2H), 7.39 (dd, *J* = 8.1, 4.0 Hz, 1H), 4.11 (s, 2H), 3.88 (s, 2H), 3.59 (aquint, *J* = 8.7 Hz, 1H), 2.67 (td, *J* = 9.3, 2.2 Hz, 2H), 2.39 (td, *J* = 9.7, 2.2 Hz, 2H), 1.45 (s, 9H); ^{13}**C NMR** (150.9 MHz, $CDCl_3$) δ 156.3, 149.8, 147.1, 143.0, 135.8, 129.4, 128.8, 124.0, 121.3, 79.4, 41.4, 40.1 (2C), 34.1, 34.0, 29.7, 28.4, 28.3 (3C); **HRMS** (TOF MS ES+) *m/z*: $[M + H]^+$ calculated for $C_{20}H_{25}N_2O_2$, 325.1916; found 325.1905.

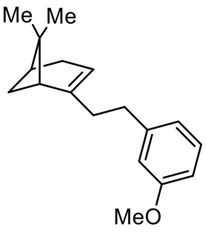

Arylated product **18** was prepared according to Method E. The following amounts of reagents were used: alcohol **53** (24 mg, 0.15 mmol, 1.0 equiv), MsCl (17 µL, 0.22 mmol, 1.5 equiv), Et_3N (30. µL, 0.22 mmol, 1.5 equiv), CH_2Cl_2 (0.70 mL, 0.20 M in substrate). The reaction mixture was allowed to stir at rt for 1 hour. The reaction was concentrated in vacuo and the following amounts of reagents were used: boronic acid (40. mg, 0.26 mmol, 1.8 equiv), $NiBr_2$(dme) (4.5 mg, 15 µmol, 10. mol %), Dtbbpy (3.9 mg, 15 µmol, 10. mol %), K_3PO_4 (50. mg, 0.23 mmol, 1.6 equiv), NaI (220 mg, 1.5 mmol, 10. equiv), H_2O (29 µL, 1.6 mmol, 11 equiv), and anhydrous *t*-BuOH (1.5 mL, 0.10 M in substrate). The residue was then purified by flash column chromatography (0–20% benzene/hexanes) to afford the title compound as a clear oil (21 mg, 82 µmol, 56%). Analytical data is consistent with **18**.

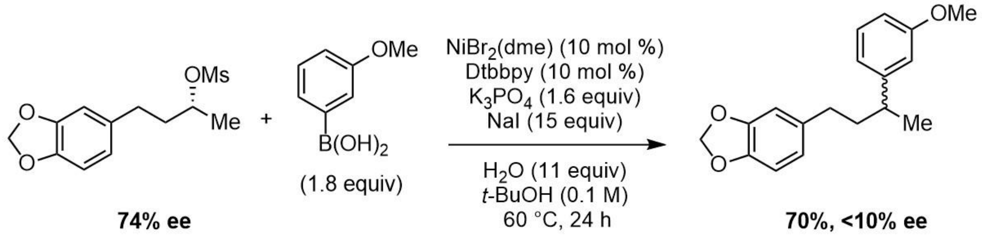

Arylated product **20** was prepared according to Method A. The following amounts of reagents were used: mesylate **(*R*)-54** (29 mg, 0.11 mmol, 1.0 equiv), boronic acid (29 mg, 0.19 mmol, 1.8 equiv), $NiBr_2$(dme) (3.4 mg, 11 µmol, 10. mol %), Dtbbpy (2.9 mg, 11 µmol, 10. mol %), K_3PO_4 (36 mg, 0.17 mmol, 1.6 equiv), NaI (160 mg, 1.1 mmol, 10. equiv), H_2O (21 µL, 1.2 mmol, 11 equiv), and anhydrous *t*-BuOH (1.1 mL, 0.10 M in substrate). The residue was then purified by flash column chromatography (0–25% benzene/hexanes) to afford the title compound as a clear oil (21 mg, 70. µmol, 70%). **TLC R_f** = 0.3 (25% benzene/hexanes, CAM stain); **^{1}H NMR** (500 MHz, $CDCl_3$) δ 7.25–7.20 (m, 1H), 6.80 (ad, *J* = 7.5 Hz, 1H), 6.75–6.74 (m, 2H), 6.71–6.69 (m, 1H), 6.62 (s, 1H), 6.57 (ad, *J* = 7.9 Hz, 1H), 5.90 (ad, *J* = 1.3 Hz, 2H), 3.81 (s, 3H), 2.70–2.26 (m, 1H), 2.44 (t, *J* = 7.2 Hz, 2H), 1.90–1.80 (m, 2H), 1.26 (d, *J* = 8.1 Hz, 3H); **Chiral SFC:** (Chiralcel OJ-H, 3% IPA in CO_2, 2.0 mL/min, λ = 210 nm) indicated 9% ee: t_R (major) = 29.8 min, t_R (minor) = 27.2 min. Analytical data is consistent with literature values.[9]

2. Enantioenriched Products

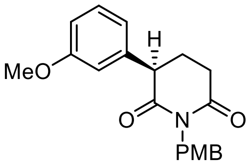

Arylated product **(–)-10** was prepared according to Method C. The following amounts of reagents were used: mesylate **55** (33 mg, 0.10 mmol, 1.0 equiv), boronic acid (28 mg, 0.18 mmol, 1.8 equiv), $Ni(cod)_2$ (2.8 mg, 10. µmol, 10. mol %), (*S*)-4-heptylBiOX (6.9 mg, 20. µmol, 20. mol %), K_3PO_4 (35 mg, 0.16 mmol, 1.6 equiv), NaI (150 mg, 0.10 mmol, 10. equiv), and anhydrous 1,4-Dioxane and *t*-BuOH (1:1, 0.10 M in substrate). The residue was then purified by flash column chromatography (0–25% EtOAc/hexanes) to afford the title compound as a clear oil (23 mg, 67 µmol, 66%). **TLC R_f** = 0.3 (35% EtOAc/hexanes, $KMnO_4$ stain); **^{1}H NMR** (500 MHz, $CDCl_3$) δ 7.39 (d, *J* = 8.6 Hz, 2H), 7.25 (t, *J* = 7.9 Hz, 1H), 6.82 (d, *J* = 8.6 Hz, 3H), 6.69 (d, *J* = 7.6 Hz, 1H), 6.61 (s, 1H), 4.96 (d, *J* = 5.5 Hz, 2H), 3.80–3.79 (m, 1H), 3.78 (s, 3H), 3.71 (s, 3H), 2.78–2.62 (m, 2H), 2.24–2.12 (m, 2H); **$[\alpha]_D^{23}$** = –16.6° (c 7.1 mg/1.5 mL, $CHCl_3$); **Chiral SFC** (Chiralcel AD-H, 20% IPA in CO_2, 1.5 mL/min, λ = 210 nm) indicated 86% ee: t_R (major) = 4.2 min, t_R (minor) = 5.9 min. Analytical data consistent with **10**.

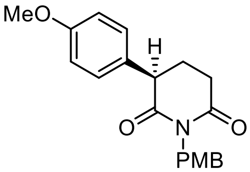

Arylated product **(–)-56** was prepared according to Method C. The following amounts of reagents were used: mesylate **55** (32 mg, 0.10 mmol, 1.0 equiv), boronic acid (26 mg, 0.17 mmol, 1.8 equiv), $Ni(cod)_2$ (2.6 mg, 10. µmol, 10. mol %), (*S*)-4-heptylBiOX (6.4 mg, 20. µmol, 20. mol %), K_3PO_4 (32 mg, 0.19 mmol, 1.6 equiv), NaI (140 mg, 0.10 mmol, 10. equiv), and anhydrous 1,4-Dioxane and *t*-BuOH (1:1, 0.10 M in substrate). The residue was then purified by flash column chromatography (0–35% EtOAc/hexanes) to afford the title compound as a clear oil (20. mg, 59 µmol, 62%). **TLC R_f** = 0.3 (35% EtOAc/hexanes, $KMnO_4$ stain); **^{1}H NMR** (500 MHz, $CDCl_3$) δ 7.38 (d, *J* = 8.6 Hz, 2H), 7.03 (d, *J* = 8.7 Hz, 2H), 6.89 (d, *J* = 8.6 Hz, 2H), 6.82 (d, *J* = 8.6 Hz, 2H), 4.98 (d, *J* = 10.3 Hz, 2H), 3.79 (s, 3H), 3.78 (s, 3H), 3.87–3.73 (m, 1H), 2.80–2.63 (m, 2H), 2.21–2.11 (m, 2H); **^{13}C NMR** (150.9 MHz, $CDCl_3$) δ 173.7, 172.4, 159.1, 159.0, 130.9 (2C), 130.2, 129.8, 129.1 (2C), 114.4 (2C), 113.8 (2C), 55.4, 55.3, 48.0, 42.8, 31.8, 25.4; **HRMS** (TOF MS ES+) *m/z*: $[M + Na]^+$ calculated for $C_{20}H_{21}NO_4Na$, 362.1368; found 362.1367; **$[\alpha]_D^{23}$** = –7.6° (c 11 mg/1.5 mL, $CHCl_3$); **Chiral SFC:** (Chiralcel OJ-H, 20% IPA in CO_2,1.5 mL/min, λ = 210 nm) indicated 87% ee: t_R (major) = 15.6 min, t_R (minor) = 17.0 min.

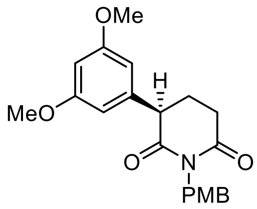

Arylated product **(–)-57** was prepared according to Method C. The following amounts of reagents were used: mesylate **55** (32 mg, 0.10 mmol, 1.0 equiv), boronic acid (32 mg, 0.18 mmol, 1.8 equiv), Ni(cod)$_2$ (2.7 mg, 10. µmol, 10. mol %), (*S*)-4-heptylBiOX (6.7 mg, 20. µmol, 20. mol %), K_3PO_4 (34 mg, 0.16 mmol, 1.6 equiv), NaI (150 mg, 0.10 mmol, 10. equiv), and anhydrous 1,4-Dioxane and *t*-BuOH (1:1, 0.10 M in substrate). The residue was then purified by flash column chromatography (0–35% EtOAc/hexanes) to afford the title compound as a clear oil (29 mg, 78 µmol, 79%). **TLC R$_f$** = 0.2 (35% EtOAc/hexanes, $KMnO_4$ stain); **^{1}H NMR** (500 MHz, $CDCl_3$) δ 7.40 (d, *J* = 8.6 Hz, 2H), 6.81 (d, *J* = 8.7 Hz, 2H), 6.36 (s, 1H), 6.21 (d, *J* = 2.0 Hz, 2H), 4.98 (s, 2H), 3.79–3.74 (m, 1H), 3.78 (s, 3H), 3.68 (s, 6H), 2.73–2.61 (m, 2H), 2.27–2.10 (m, 2H); **^{13}C NMR** (150.9 MHz, $CDCl_3$) δ 173.1, 172.3, 161.1, 159.1, 140.3, 130.9 (2C), 129.7, 113.8 (2C), 106.2 (2C), 99.5, 55.4 (2C), 55.3 (2C), 48.7, 42.7, 31.3, 25.4; **HRMS** (TOF MS ES+) *m/z*: [M + Na]$^+$ calculated for $C_{21}H_{23}NO_5Na$, 392.1474; found 392.1481; **[α]** $^{23}_{D}$ = –22.9° (c 8.6 mg/1.5 mL, $CHCl_3$); **Chiral SFC** (Chiralcel OD-H, 20% IPA in CO_2, 1.5 mL/min, λ = 210 nm) indicated 84% ee: t_R (major) = 6.4 min, t_R (minor) = 4.4 min.

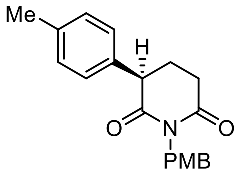

Arylated product **(–)-58** was prepared according to Method C. The following amounts of reagents were used: mesylate **55** (31 mg, 99 µmol, 1.0 equiv), boronic acid (23 mg, 0.17 mmol, 1.8 equiv), Ni(cod)$_2$ (2.6 mg, 9.0 µmol, 10. mol %), (*S*)-4-heptylBiOX (6.3 mg, 19 µmol, 20. mol %), K_3PO_4 (32 mg, 0.19 mmol, 1.6 equiv), NaI (140 mg, 0.90 mmol, 10. equiv), and anhydrous 1,4-Dioxane and *t*-BuOH (1:1, 0.10 M in substrate). The residue was then purified by flash column chromatography (0–25% EtOAc/hexanes) to afford the title compound as a clear oil (30. mg, 55 µmol, 59%). **TLC R$_f$** = 0.4 (35% EtOAc/hexanes, $KMnO_4$ stain); **^{1}H NMR** (500 MHz, $CDCl_3$) δ 7.38 (d, *J* = 8.9 Hz, 2H), 7.13 (d, *J* = 7.9 Hz, 2H), 6.99 (d, *J* = 7.9 Hz, 2H), 6.82 (d, *J* = 8.6 Hz, 2H), 4.98 (d, *J* = 10.3 Hz, 2H), 3.78 (s, 3H), 3.78–3.76 (m, 1H), 2.79–2.63 (m, 2H), 2.32 (s, 3H), 2.22–2.12 (m, 2H); **^{13}C NMR** (150.9 MHz, $CDCl_3$) δ 173.6, 172.4, 159.1, 137.4, 135.1, 130.1 (2C), 129.7, 129.6 (2C), 127.9 (2C), 113.8 (2C), 55.4, 48.4, 42.7, 31.7, 25.3, 21.2; **HRMS** (TOF MS ES+) *m/z*: [M + Na]$^+$ calculated for $C_{20}H_{21}NO_3Na$, 346.1419; found 346.1419; **[α]** $^{23}_{D}$ = –11.4° (c 5.7 mg/1.5 mL, $CHCl_3$); **Chiral SFC** (Chiralcel OB-H, 20% IPA in CO_2, 1.5 mL/min, λ = 210 nm) indicated 86% ee: t_R (major) = 13.6 min, t_R (minor) = 11.2 min.

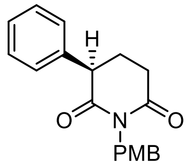

Arylated product **(–)-59** was prepared according to Method C. The following amounts of reagents were used: mesylate **55** (30. mg, 99 µmol, 1.0 equiv), boronic acid (20. mg, 0.16 mmol, 1.8 equiv), Ni(cod)$_2$ (2.5 mg, 9.0 µmol, 10. mol %), (*S*)-4-heptylBiOX (6.1 mg, 18 µmol, 20. mol %), K_3PO_4 (31 mg, 0.15 mmol, 1.6 equiv), NaI (140 mg, 0.90 mmol, 10. equiv), and anhydrous 1,4-Dioxane and *t*-BuOH (1:1, 0.10 M in substrate). The residue was then purified by flash column chromatography (0–25% EtOAc/hexanes) to afford the title compound as a clear oil (15 mg, 48 µmol, 53%). **TLC R$_f$** = 0.4 (35% EtOAc/hexanes, $KMnO_4$ stain); **^{1}H NMR** (500 MHz, $CDCl_3$) δ 7.38 (d, *J* = 8.1 Hz, 2H), 7.32–7.28 (m, 3H), 7.11 (d, *J* = 7.2 Hz, 2H), 6.83 (d, *J* = 8.7 Hz, 2H), 4.96 (d, *J* = 10.0 Hz, 2H), 3.82–3.80 (m, 1H), 3.78 (s, 3H), 2.79–2.64 (m, 2H), 2.25–2.15 (m, 2H); **^{13}C NMR** (150.9 MHz, $CDCl_3$) δ 173.4, 172.3, 159.1, 138.1, 130.9 (2C), 129.7, 128.9 (2C), 128.1 (2C), 127.7, 113.8 (2C), 55.4, 48.8, 42.8, 31.7, 25.4; **HRMS** (TOF MS ES+) *m/z*: $[M + Na]^+$ calculated for $C_{19}H_{19}NO_3Na$, 332.1263; found 332.1264; **$[\alpha]_D^{23}$** = –170.° (c 0.7 mg/1.5 mL, $CHCl_3$); **Chiral SFC** (Chiralcel OB-H, 20% IPA in CO_2, 1.5 mL/min, λ = 210 nm) indicated 80% ee: t_R (major) = 12.2 min, t_R (minor) = 13.4 min.

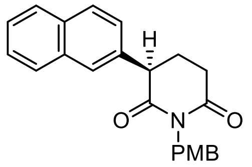

Arylated product **(–)-50** was prepared according to Method C. The following amounts of reagents were used: mesylate **55** (35 mg, 0.11 mmol, 1.0 equiv), boronic acid (33 mg, 0.19 mmol, 1.8 equiv), Ni(cod)$_2$ (2.9 mg, 11 µmol, 10. mol %), (*S*)-4-heptylBiOX (7.2 mg, 21 µmol, 20. mol %), K_3PO_4 (36 mg, 0.17 mmol, 1.6 equiv), NaI (150 mg, 0.11 mmol, 10. equiv), and anhydrous 1,4-Dioxane and *t*-BuOH (1:1, 0.10 M in substrate). Before purification, a ^{1}H NMR yield of 59% was obtained based on comparison to PhTMS as internal standard. The residue was then purified by preparative thin-layer chromatography (1:3:6, EtOAc/benzene/hexanes) to afford the title compound as a clear oil (14 mg, 39 µmol, 37%). **TLC R$_f$** = 0.4 (35% EtOAc/hexanes, $KMnO_4$ stain); **^{1}H NMR** (400 MHz, $CDCl_3$) δ 7.82–7.79 (m, 2H), 7.67–7.65 (m, 1H), 7.46–7.45 (m, 3H), 7.43 (d, *J* = 8.6 Hz, 2H), 7.25–7.22 (m, 1H), 6.86 (d, *J* = 8.5 Hz, 2H), 5.03–4.98 (m, 2H), 3.99–3.97 (m, 1H), 3.79 (s, 3H), 2.78–2.67 (m, 2H), 2.29–2.25 (m, 2H); **$[\alpha]_D^{23}$** = –18.1° (c 3.7 mg/1.5 mL, $CHCl_3$); **Chiral SFC** (Chiralcel OB-H, 40% IPA in CO_2, 1.5 mL/min, λ = 210 nm) indicated 86% ee: t_R (major) = 20.1 min, t_R (minor) = 17.7 min. Analytical data consistent with **50**.

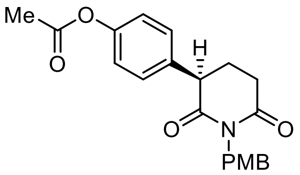

Arylated product **(–)-60** was prepared according to Method C. The following amounts of reagents were used: mesylate **55** (30 mg, 92 µmol, 1.0 equiv), boronic acid (30 mg, 0.17 mmol, 1.8 equiv), Ni(cod)$_2$ (2.5 mg, 9.0 µmol, 10. mol %), (*S*)-4-heptylBiOX (6.2 mg, 18 µmol, 20. mol %), K_3PO_4 (31 mg, 0.15 mmol, 1.6 equiv), NaI (140 mg, 0.92 mmol, 10. equiv), and anhydrous 1,4-Dioxane and *t*-BuOH (1:1, 0.10 M in substrate). The residue was then purified by flash column chromatography (0–30% EtOAc/hexanes) to afford the title compound as a clear oil (19 mg, 52 µmol, 56%). **TLC R$_f$** = 0.3 (35% EtOAc/hexanes, $KMnO_4$ stain); **^{1}H NMR** (500 MHz, $CDCl_3$) δ 7.37 (d, *J* = 8.6 Hz, 2H), 7.12 (d, *J* = 8.6 Hz, 2H), 7.06 (d, *J* = 8.5 Hz, 2H), 6.8 (d, *J* = 8.6 Hz, 2H), 4.98 (d, *J* = 7.1 Hz, 2H), 3.81–3.79 (m, 1H), 3.78 (s, 3H), 2.81–2.63 (m, 2H), 2.28 (s, 3H), 2.22–2.10 (m, 2H); **^{13}C NMR** (150.9 MHz, $CDCl_3$) δ 173.2, 172.1, 169.5, 159.1, 150.1, 135.6, 130.9 (2C), 129.6, 129.2 (2C), 122.1 (2C), 113.8 (2C), 55.3, 48.3, 42.8, 31.8, 25.4, 21.2; **HRMS** (TOF MS ES+) *m/z*: $[M + Na]^+$ calculated for $C_{21}H_{21}NO_5Na$, 390.1317; found 390.1321; **$[\alpha]^{23}_{D}$** = –8.5° (c 5.3 mg/1.5 mL, $CHCl_3$); **Chiral SFC** (Chiralcel OB-H, 20% IPA in CO_2, 1.5 mL/min, λ = 210 nm) indicated 76% ee: t_R (major) = 22.2 min, t_R (minor) = 18.5 min.

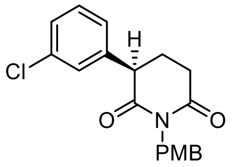

Arylated product **(–)-49** was prepared according to Method C. The following amounts of reagents were used: mesylate **55** (34 mg, 0.11 mmol, 1.0 equiv), boronic acid (30. mg, 0.19 mmol, 1.8 equiv), Ni(cod)$_2$ (2.9 mg, 11 µmol, 10. mol %), (*S*)-4-heptylBiOX (7.1 mg, 21 µmol, 20. mol %), K_3PO_4 (36 mg, 0.17 mmol, 1.6 equiv), NaI (160 mg, 0.11 mmol, 10. equiv), and anhydrous 1,4-Dioxane and *t*-BuOH (1:1, 0.10 M in substrate). The residue was then purified by flash column chromatography (0–20% EtOAc/hexanes) to afford the title compound as a clear oil (23 mg, 67 µmol, 64%). **TLC R$_f$** = 0.4 (35% EtOAc/hexanes, $KMnO_4$ stain); **^{1}H NMR** (400 MHz, $CDCl_3$) δ 7.37 (d, *J* = 8.6 Hz, 2H), 7.27–7.26 (m, 2H), 7.10 (s, 1H), 7.00–6.98 (m, 1H), 6.83 (d, *J* = 8.7 Hz, 2H), 4.97–4.92 (m, 2H), 3.78 (s, 3H), 3.78–3.75 (m, 1H), 2.79–2.65 (m, 2H), 2.24–2.11 (m, 2H); **$[\alpha]^{23}_{D}$** = –13.6° (c 14.5 mg/1.5 mL, $CHCl_3$); **Chiral SFC** (Chiracel OB-H, 12% IPA in CO_2, 1.5 mL/min, λ = 210 nm) indicated 85% ee: t_R (major) = 21.9 min, t_R (minor) = 23.9 min. Analytical data consistent with **49**.

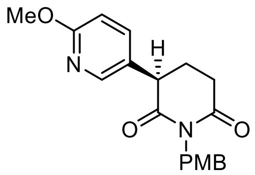

Arylated product **(+)-47** was prepared according to Method D. The following amounts of reagents were used: mesylate **55** (33 mg, 0.10 mmol, 1.0 equiv), boronic acid (32 mg, 0.21 mmol, 2.1 equiv), Ni(cod)$_2$ (2.8 mg, 10. µmol, 10. mol %), (*S*)-4-heptylBiOX; (6.7 mg, 20. µmol, 20. mol %), K_3PO_4 (34 mg, 0.16 mmol, 1.6 equiv), NaI (230 mg, 1.5 mmol, 15 equiv), and anhydrous 1,4-Dioxane and *t*-BuOH (1:1, 0.10 M in substrate) residue was then purified by flash column chromatography (0–45% EtOAc/hexanes) to afford the title compound as a clear oil (18 mg, 54 µmol, 54%). **TLC R$_f$** = 0.3 (35% EtOAc/hexanes, $KMnO_4$ stain); **^{1}H NMR** (600 MHz, $CDCl_3$) δ 7.97 (d, *J* = 2.1 Hz, 1H), 7.36 (d, *J* = 8.6 Hz, 2H), 7.33 (dd, *J* = 8.6, 2.5 Hz, 1H), 6.82 (d, *J* = 8.7 Hz, 2H), 6.73 (d, *J* = 8.6, 1H), 4.93 (q, *J* = 13.6 Hz, 2H), 3.92 (s, 3H), 3.78 (s, 3H), 3.72 (dd, *J* = 11.0, 5.2 Hz, 1H), 2.87–2.83 (m, 1H), 2.75–2.69 (m, 1H), 2.21–2.10 (m, 2H); $[\alpha]_D^{23}$ = +11.0° (c 6.6 mg/2 mL, $CHCl_3$); **Chiral SFC** (Chiracel OB-H col, 20% IPA in CO_2, 1.5 mL/min, 210 nm) 88% ee t_R (major enantiomer) = 10. min, t_R (minor enantiomer) = 12 min. Analytical data consistent with **47**.

D. General Procedures for Starting Material Synthesis

1. Method F: Mesylation of Alcohol

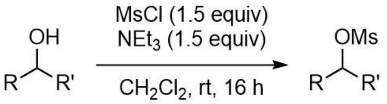

To a flame-dried round-bottom flask equipped with a stir bar was added alcohol (1.0 equiv) and CH_2Cl_2 (0.20 M in substrate) under N_2. Then, Et_3N (1.5 equiv) and MsCl (1.5 equiv) were added, and the reaction mixture was allowed to stir at rt for at least 2 h. Saturated $NaHCO_3$ (aq) was added to quench, and the reaction mixture was extracted with CH_2Cl_2 (3 x 20 mL). The combined organic layers were washed with brine, dried over Na_2SO_4, filtered, and concentrated in vacuo.

E. Synthesis and Characterization Data of Alcohols, Mesylates, and Iodides

1. Substrate Scope

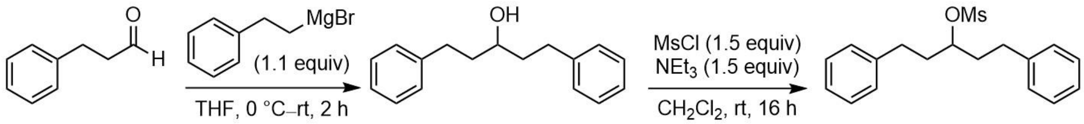

Scheme SI-4: Synthesis of Mesylate **1**

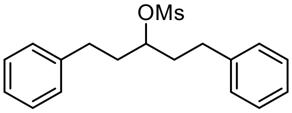

Mesylate **1** was prepared according to Method F. The following amounts of reagents were used: alcohol (1.6 g, 6.5 mmol, 1.0 equiv), Et_3N (1.4 mL, 9.8 mmol, 1.5 equiv), MsCl (1.8 mL, 9.8 mmol, 1.5 equiv), and CH_2Cl_2 (33 mL, 0.20 M in substrate). The compound was purified by flash column chromatography (0–30% EtOAc/hexanes) to afford the title compound as a white solid (1.8 g, 5.8 mmol, 89%). **^{1}H NMR** (400 MHz, $CDCl_3$) δ 7.31–7.27 (m, 4H), 7.21–7.16 (m, 6H), 4.84–4.81 (m, 1H), 2.98 (s, 3H), 2.77–2.68 (m, 4H), 2.11–2.03 (m, 4H). Analytical data is consistent with literature values.[11]

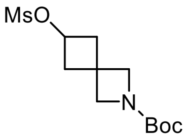

Mesylate **SI-1** was prepared according to Method F. The following amounts of reagents were used: alcohol (210 mg, 1.0 mmol, 1.0 equiv), Et_3N (0.21 mL, 1.5 mmol, 1.5 equiv), MsCl (0.12 mL, 1.5 mmol, 1.5 equiv), and CH_2Cl_2 (5.0 mL, 0.20 M in substrate). The compound was purified by flash column chromatography (0–50% EtOAc/hexanes) to afford the title compound as a white solid (270 mg, 0.92 mmol, 92%). **TLC R_f** = 0.5 (50% EtOAc/hexanes, $KMnO_4$ stain); **^{1}H NMR** (600 MHz, $CDCl_3$) δ 4.91 (quint, *J* = 7.2 Hz, 1H), 3.93 (ad, *J* = 2.8 Hz, 4H), 2.99 (s, 3H), 2.71–2.67 (m, 2H), 2.45–2.45 (m, 2H), 1.43 (s, 9H); **^{13}C NMR** (150.9 MHz, $CDCl_3$) δ 156.1, 79.8, 69.3, 41.6 (4C), 38.4, 30.8, 28.5 (3C); **HRMS** (TOF MS ES+) *m/z*: $[M + Na]^+$ calculated for $C_{12}H_{21}NO_5SNa$, 314.1038; found 314.1035.

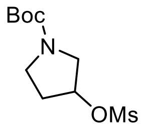

Mesylate **SI-2** was prepared according to Method F. The following amounts of reagents were used: alcohol (190 mg, 1.0 mmol, 1.0 equiv), Et_3N (0.21 mL, 1.5 mmol, 1.5 equiv), MsCl (0.21 mL, 1.5 mmol, 1.5 equiv), and CH_2Cl_2 (5.0 mL, 0.20 M in substrate). The compound was purified by flash column chromatography (0–50% EtOAc/hexanes) to afford the title compound as a clear oil (240 mg, 0.92 mmol, 92%). **TLC R_f** = 0.5 (50% EtOAc/hexanes, $KMnO_4$ stain); **^{1}H NMR** (600 MHz, $CDCl_3$) δ (mixture of rotamers) 5.23 (br s, 1H), 3.71–3.63 (m, 1H), 3.61–3.47 (m, 3H), 3.06 (s, 3H), 2.29–2.25 (m, 1H), 2.14 (br s, 1H), 1.47 (s, 9H); **^{13}C NMR** (150.9 MHz, $CDCl_3$) δ (mixture of rotamers) 154.3, 154.2, 80.0, 79.99, 79.94, 79.6, 52.3, 51.8, 43.7, 43.3, 38.8, 28.5 (3C); **HRMS** (TOF MS ES+) *m/z*: $[M + Na]^+$ calculated for $C_{10}H_{19}NO_5SNa$, 288.0882; found 288.0869.

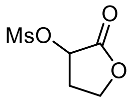

Mesylate **SI-3** was prepared according to Method F. The following amounts of reagents were used: alcohol (210 mg, 2.0 mmol, 1.0 equiv), Et_3N (0.42 mL, 3.0 mmol, 1.5 equiv), MsCl (0.23 mL, 3.0 mmol, 1.5 equiv), and CH_2Cl_2 (10. mL, 0.20 M in substrate). The compound was purified by flash column chromatography (0–50% EtOAc/hexanes) to afford the title compound as a clear oil (220 mg, 1.2 mmol, 61%). **TLC R_f** = 0.5 (50% EtOAc/hexanes, $KMnO_4$ stain); **^{1}H NMR** (500 MHz, $CDCl_3$) δ 5.34 (t, *J* = 8.9 Hz, 1H), 4.54–4.49 (m, 1H), 4.36–4.31 (m, 1H), 3.27 (s, 3H), 2.79–2.75 (m, 1H), 2.59–2.51 (m, 1H); **^{13}C NMR** (100.6 MHz, $CDCl_3$) δ 171.6, 73.4, 65.4, 39.9, 29.5; **HRMS** (TOF MS ES+) *m/z*: $[M + Na]^+$ calculated for $C_5H_8O_5SNa$, 202.9990; found 202.9989.

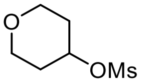

Mesylate **SI-4** was prepared according to Method F. The following amounts of reagents were used: alcohol (130 mg, 1.3 mmol, 1.0 equiv), Et_3N (0.25 mL, 1.9 mmol, 1.5 equiv), MsCl (0.15 mL, 1.9 mmol, 1.5 equiv), and CH_2Cl_2 (6.2 mL, 0.20 M in substrate). The compound was purified by flash column chromatography (0–25% EtOAc/hexanes) to afford the title compound as a white solid (190 mg, 1.1 mmol, 86%). **TLC R_f** = 0.2 (25% EtOAc/hexanes); **^{1}H NMR** (500 MHz, $CDCl_3$) δ 4.90–4.89 (m, 1H), 3.96–3.94 (m, 2H), 3.57–3.53 (m, 2H), 3.04 (s, 3H), 2.06–2.03 (m, 2H), 1.89–1.87 (m, 2H), Analytical data is consistent with literature values.[12]

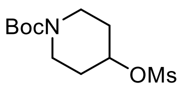

Mesylate **SI-5** was prepared according to Method F. The following amounts of reagents were used: alcohol (0.40 g, 2.0 mmol, 1.0 equiv), Et_3N (0.42 mL, 3.0 mmol, 1.5 equiv), MsCl (0.23 mL, 3.0 mmol, 1.5 equiv), and CH_2Cl_2 (10. mL, 0.20 M in substrate). The compound was purified by flash column chromatography (0–50% EtOAc/hexanes) to afford the title compound as a yellow oil (0.52 g, 1.9 mmol, 92%). **TLC R_f** = 0.4 (50% EtOAc/hexanes, $KMnO_4$ stain); **^{1}H NMR** (500 MHz, $CDCl_3$) δ 4.94–4.86 (m, 1H), 3.72–3.69 (m, 2H), 3.32–3.27 (m, 2H), 3.04 (s, 3H), 2.00–1.94 (m, 2H), 1.85–1.79 (m, 2H), 1.46 (s, 9H). Analytical data is consistent with literature values.[5]

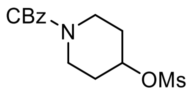

Mesylate **SI-6** was prepared according to Method F. The following amounts of reagents were used: alcohol (240 mg, 1.0 mmol, 1.0 equiv), Et_3N (0.21 mL, 1.5 mmol, 1.5 equiv), MsCl (0.12 mL, 1.5 mmol, 1.5 equiv), and CH_2Cl_2 (5.0 mL, 0.20 M in substrate). The compound was purified by flash column chromatography (0–50% EtOAc/hexanes) to afford the title compound as a yellow oil (0.31 g, 0.85 mmol, 85%). **TLC R_f** = 0.4 (50% EtOAc/hexanes); **^{1}H NMR** (400 MHz, $CDCl_3$) δ 7.38–7.31 (m, 5H), 5.13 (s, 2H), 4.93–4.88 (m, 1H), 3.78–3.73 (m, 2H), 3.45–3.40 (m, 2H), 3.04 (s, 3H), 1.98–1.95 (m, 2H), 1.86–1.82 (m, 2H). Analytical data is consistent with literature values.[13]

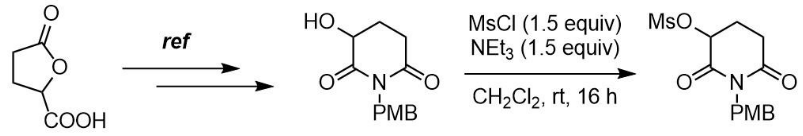

Scheme SI-5: Synthesis of Mesylate **55**[14]

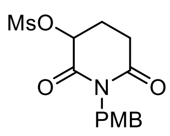

Mesylate **55** was prepared according to Method F. The following amounts of reagents were used: alcohol (0.52 mg, 2.1 mmol, 1.0 equiv), Et_3N (0.43 mL, 3.1 mmol, 1.5 equiv), MsCl (0.24 mL, 3.1 mmol, 1.5 equiv), and CH_2Cl_2 (11 mL, 0.20 M in substrate). The compound was purified by flash column chromatography (0–50% EtOAc/hexanes) to afford the title compound as a pale yellow solid (0.60 g, 1.9 mmol, 89%). **TLC R_f** = 0.4 (50% EtOAc/hexanes, $KMnO_4$ stain); **^{1}H NMR** (500 MHz, $CDCl_3$) δ 7.32 (d, *J* = 8.8 Hz, 2H), 6.83 (d, *J* = 8.7 Hz, 2H), 5.22–5.19 (m, 1H), 4.91–4.85

(m, 2H), 3.78 (s, 3H), 3.27 (s, 3H), 2.98 (dt, J = 17.8, 4.8 Hz, 1H), 2.74–2.67 (m, 1H), 2.38–2.32 (m, 1H), 2.29–2.21 (m, 1H). Analytical data is consistent with literature values.[1]

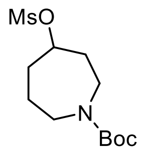

Mesylate **SI-7** was prepared according to Method F. The following amounts of reagents were used: alcohol (220 mg, 1.0 mmol, 1.0 equiv), Et_3N (0.21 mL, 1.5 mmol, 1.5 equiv), MsCl (0.12 mL, 1.5 mmol, 1.5 equiv), and CH_2Cl_2 (5.0 mL, 0.20 M in substrate). The compound was purified by flash column chromatography (0–50% EtOAc/hexanes) to afford the title compound as a clear oil (270 mg, 0.93 mmol, 93%). **TLC R$_f$** = 0.2 (25% EtOAc/hexanes, $KMnO_4$ stain); **^{1}H NMR** (500 MHz, $CDCl_3$) δ (mixture of rotamers) 4.91–4.89 (m, 1H), 3.47–3.36 (m, 4H), 3.01 (s, 3H), 2.07–1.90 (m, 5H), 1.72–1.67 (m, 1H), 1.46 (s, 9H); **^{13}C NMR** (150.9 MHz, $CDCl_3$) δ (mixture of rotamers) 155.54, 155.48, 81.3, 81.2, 79.8, 79.8, 46.4, 45.4, 40.9, 40.5, 38.8, 35.7, 35.2, 32.9, 32.4, 28.6, 21.9, 21.6; **HRMS** (TOF MS ES+) m/z: $[M + Na]^+$ calculated for $C_{12}H_{23}NO_5SNa$, 316.1195; found 316.1183.

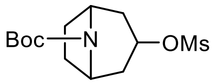

Mesylate **SI-8** was prepared according to Method F. The following amounts of reagents were used: alcohol (210 mg, 1.0 mmol, 1.0 equiv), Et_3N (0.21 mL, 1.5 mmol, 1.5 equiv), MsCl (0.12 mL, 1.5 mmol, 1.5 equiv), and CH_2Cl_2 (5.0 mL, 0.20 M in substrate). The compound was purified by flash column chromatography (0–50% EtOAc/hexanes) to afford the title compound as a yellow oil (270 mg, 0.87 mmol, 87%). **TLC R$_f$** = 0.6 (50% EtOAc/hexanes, $KMnO_4$ stain); **^{1}H NMR** (600 MHz, $CDCl_3$) δ (mixture of rotamers) 5.06–5.02 (m, 1H), 4.32–4.11 (m, 3H), 3.01 (s, 3H), 2.07–1.94 (m, 7H), 1.47 (ad, J = 10.8 Hz, 9H); **^{13}C NMR** (150.9 MHz, $CDCl_3$) δ (mixture of rotamers) 153.3, 153.2, 79.9, 79.7, 76.9 (2C), 75.2 (2C), 60.5, 39.1 (2C), 38.7, 28.5 (3C); **HRMS** (TOF MS ES+) m/z: $[M + Na]^+$ calculated for $C_{13}H_{23}NO_5SNa$, 328.1195; found 328.1194.

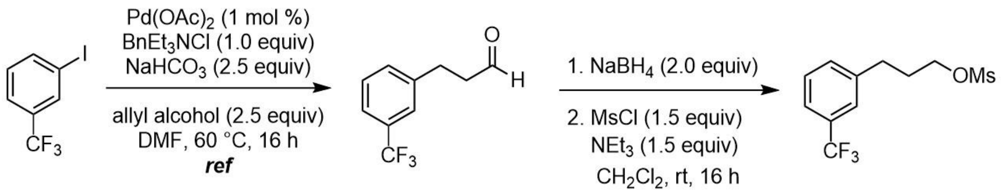

Scheme SI-6: Synthesis of Mesylate **SI-9**[15]

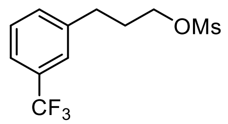

Mesylate **SI-9** was prepared according to the following two-step procedure. In a round-bottom flask with a stir bar open to air was added aldehyde (250 mg, 1.2 mmol, 1.0 equiv) and MeOH (6.2 mL, 0.20 M in substrate). Then, $NaBH_4$ (94 mg, 2.5 mmol, 2.0 equiv) was added in one portion and the reaction was allowed to stir open to air for 1 h. Upon completion, the solution was concentrated in vacuo, followed by dilution with CH_2Cl_2. H_2O was added and the mixture was extracted with CH_2Cl_2 (3 x 10 mL). The combined organic layers were dried with Na_2SO_4, filtered, and concentrated in vacuo. Then the flask was capped with a stopper, evacuated and backfilled with N_2. Under an N_2 atmosphere, CH_2Cl_2 (6.2 mL, 0.20 M in substrate) and Et_3N (260 μL, 1.9 mmol, 1.5 equiv) were added. Then MsCl (140 μL, 1.9 mmol, 1.5 equiv) was added dropwise. The reaction was allowed to stir overnight (16 h). Then, saturated $NaHCO_3$(aq) was added, and the reaction mixture was extracted with CH_2Cl_2 (3 x 10 mL). The combined organic layers were washed with brine, dried over Na_2SO_4, filtered, and concentrated in vacuo. The compound was purified by flash column chromatography (0–30% EtOAc/hexanes) to afford the title compound as a pale-yellow oil (190 mg, 0.67 mmol, 54% yield over 2 steps). **TLC R$_f$** = 0.2 (20% EtOAc/hexanes, CAM stain); **^{1}H NMR** (500 MHz, $CDCl_3$) δ 7.52–7.35 (m, 4H), 4.24 (t, J = 6.2 Hz, 2H), 3.01 (s, 3H), 2.83 (t, J = 7.6 Hz, 2H), 2.10 (quint, J = 6.9 Hz, 2H). Analytical data is consistent with literature values.[16]

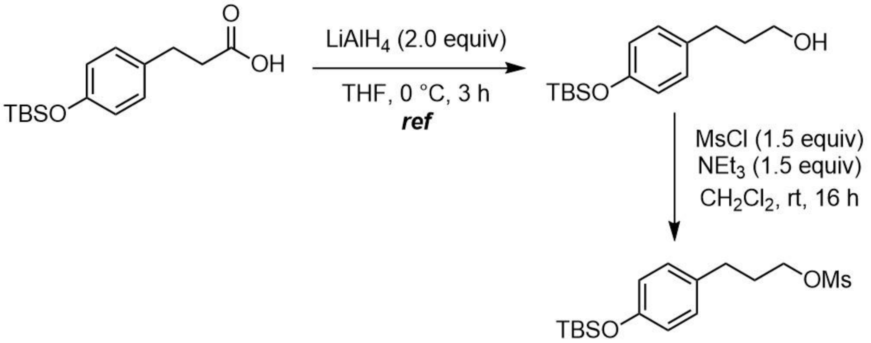

Scheme SI-7: Synthesis of Mesylate **SI-10**[17]

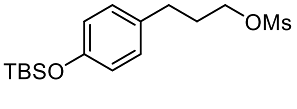

Mesylate **SI-10** was prepared according to Method F. The following amounts of reagents were used: alcohol (110 mg, 0.41 mmol, 1.0 equiv), Et_3N (90. μL, 0.62 mmol, 1.5 equiv), MsCl (50. μL, 0.62 mmol, 1.5 equiv), and CH_2Cl_2 (2.0 mL, 0.20 M in substrate). The compound was purified by flash column chromatography (0–20% EtOAc/hexanes) to afford the title compound as a colorless oil (94 mg, 0.27 mmol, 66%). **TLC R$_f$** = 0.3 (20% EtOAc/hexanes, CAM stain); **^{1}H**

NMR (500 MHz, $CDCl_3$) δ 7.03 (d, *J* = 8.1 Hz, 2H), 6.77 (d, *J* = 8.1 Hz, 2H), 4.21 (t, *J* = 6.3 Hz, 2H), 2.98 (s, 3H), 2.68 (t, *J* = 7.5 Hz, 2H), 2.04 (quint, *J* = 7.0 Hz, 2H), 0.98 (s, 9H), 0.18 (s, 6H). Analytical data is consistent with literature values.[18]

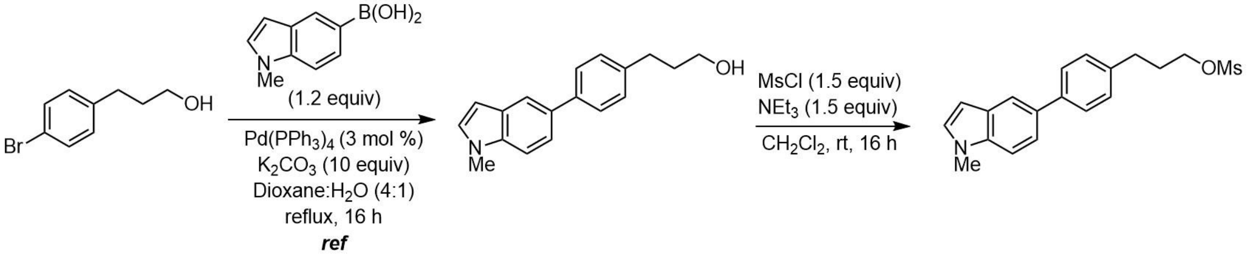

Scheme SI-8: Synthesis of Mesylate **SI-11**[19]

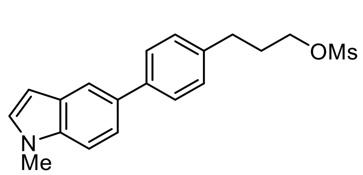

Mesylate **SI-11** was prepared according to Method F. The following amounts of reagents were used: alcohol (190 mg, 0.73 mmol, 1.0 equiv), Et_3N (0.15 mL, 1.1 mmol, 1.5 equiv), MsCl (85 µL, 1.1 mmol, 1.5 equiv), and CH_2Cl_2 (4.0 mL, 0.20 M in substrate). The compound was purified by flash column chromatography (0–50% EtOAc/hexanes) to afford the title compound as a yellow oil (200 mg, 0.59 mmol, 80%). **TLC R_f** = 0.6 (50% EtOAc/hexanes, CAM stain); **^{1}H NMR** (500 MHz, $CDCl_3$) δ 7.82 (d, *J* = 1.4 Hz, 1H), 7.59 (d, *J* = 3.1 Hz, 2H), 7.47 (dd, *J* = 8.4, 1.8 Hz, 1H), 7.39–7.36 (m, 1H), 7.26–7.24 (m, 2H), 7.08 (d, *J* = 3.0 Hz, 1H), 6.53 (d, *J* = 2.8 Hz, 1H), 4.29 (t, *J* = 6.4 Hz, 2H), 3.83 (s, 3H), 3.01 (s, 3H), 2.81 (t, *J* = 7.4 Hz, 2H), 2.15–2.10 (m, 2H); **^{13}C NMR** (100.6 MHz, $CDCl_3$) δ 140.8, 138.3, 136.3, 132.6, 129.6, 129.0, 128.9 (2C), 127.6 (2C), 121.4, 119.3, 109.6, 101.4, 69.4, 37.5, 33.1, 31.3, 30.8; **HRMS** (TOF MS ES+) *m/z*: $[M + Na]^+$ calculated for $C_{19}H_{21}NO_3SNa$, 336.0882; found 366.1151.

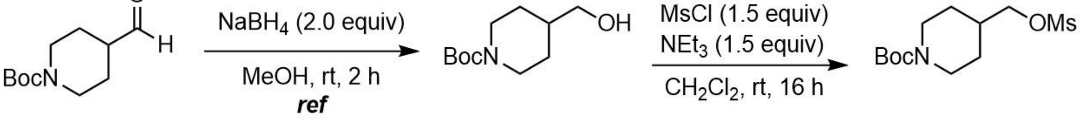

Scheme SI-9: Synthesis of Mesylate **SI-12**[17]

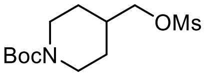

Mesylate **SI-12** was prepared according to Method F. The following amounts of reagents were used: alcohol (140 mg, 0.67 mmol, 1.0 equiv), Et_3N (0.14 mL, 1.0 mmol, 1.5 equiv), MsCl (77 µL, 1.0 mmol, 1.5 equiv), and CH_2Cl_2 (3.4 mL, 0.20 M in substrate). The compound was purified by flash column chromatography (0–50% EtOAc/hexanes) to afford the title compound as a yellow oil (93 mg, 0.32 mmol, 47%). **TLC R_f** = 0.4 (50% EtOAc/hexanes, $KMnO_4$ stain); **^{1}H NMR** (500 MHz, $CDCl_3$) δ 4.27–4.10 (br s, 2H), 4.07 (d, *J* = 4.1 Hz, 2H), 3.01 (s, 3H), 2.71 (at, *J* = 11.0 Hz, 2H), 1.98–1.85 (m, 1H), 1.74 (d, *J* = 13.0 Hz, 2H), 1.46 (s, 9H), 1.22 (qd, *J* = 12.3, 3.5 Hz, 2H). Analytical data is consistent with literature values.[20]

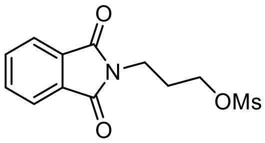

Mesylate **SI-13** was prepared according to Method F. The following amounts of reagents were used: alcohol (210 mg, 1.0 mmol, 1.0 equiv), Et_3N (0.20 mL, 1.5 mmol, 1.5 equiv), MsCl (0.12 mL, 1.5 mmol, 1.5 equiv), and CH_2Cl_2 (5.0 mL, 0.20 M in substrate). The compound was purified by flash column chromatography (0–50% EtOAc/hexanes) to afford the title compound as a yellow oil (250 mg, 0.89 mmol, 89%). **TLC R_f** = 0.6 (50% EtOAc/hexanes, CAM stain); **^{1}H NMR** (500 MHz, $CDCl_3$) δ 7.87–7.85 (m, 2H), 7.75–7.73 (m, 2H), 4.29 (t, *J* = 6.0 Hz, 2H), 3.86 (t, *J* = 6.6 Hz, 2H), 3.05 (s, 3H), 2.17–2.15 (m, 2H). Analytical data is consistent with literature values.[21]

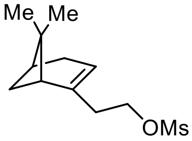

Mesylate **SI-14** was prepared according to Method F. The following amounts of reagents were used: alcohol (170 mg, 1.0 mmol, 1.0 equiv), Et_3N (0.21 mL, 1.5 mmol, 1.5 equiv), MsCl (0.12 mL, 1.5 mmol, 1.5 equiv), and CH_2Cl_2 (5.0 mL, 0.20 M in substrate). The compound was purified by flash column chromatography (0–25% EtOAc/hexanes) to afford the title compound as a yellow oil (240 mg, 0.91 mmol, 91%). **TLC R_f** = 0.5 (25% EtOAc/hexanes, $KMnO_4$ stain); **^{1}H NMR** (500 MHz, $CDCl_3$) δ 5.36–5.36 (m, 1H), 4.23–4.20 (m, 2H), 2.99 (s, 3H), 2.43−2.36 (m, 3H), 2.30−2.18 (m, 2H), 2.10−2.04 (m, 2H), 1.28 (s, 3H), 1.16 (d, *J* = 8.6 Hz, 1H), 0.83 (s, 3H). Analytical data is consistent with literature values.[11]

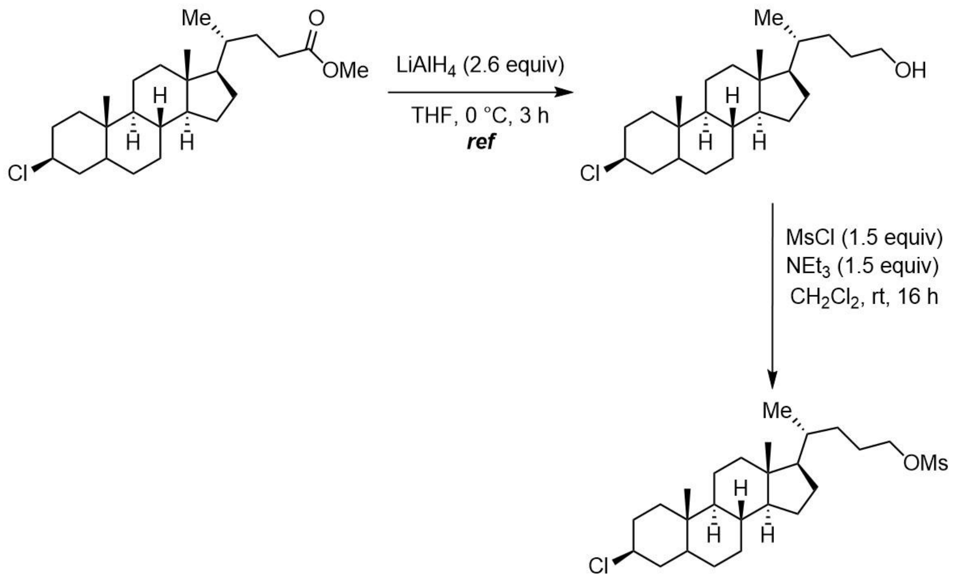

Scheme SI-10: Synthesis of Mesylate **SI-15**[17]

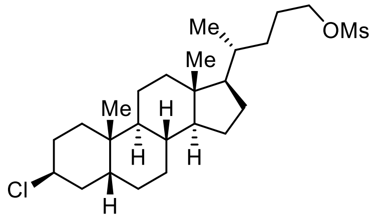

Mesylate **SI-15** was prepared according to Method F. The following amounts of reagents were used: alcohol (140 mg, 0.38 mmol, 1.0 equiv), Et_3N (44 µL, 0.57 mmol, 1.5 equiv), MsCl (80. µL, 0.57 mmol, 1.5 equiv), and CH_2Cl_2 (1.9 mL, 0.20 M in substrate). The compound was purified by flash column chromatography (0–15% EtOAc/hexanes) to afford the title compound as a white solid (99 mg, 0.22 mmol, 57%). **TLC R_f** = 0.6 (25% EtOAc/hexanes, CAM stain); **^{1}H NMR** (600 MHz, $CDCl_3$) δ 4.60–4.56 (m, 1H), 4.20 (ddt, *J* = 16.6, 10.0, 6.9 Hz, 2H), 3.00 (s, 3H), 2.22 (ddd, *J* = 15.3, 12.8, 3.3 Hz, 1H), 2.01–1.75 (m, 6H), 1.75–1.32 (m, 12H), 1.32–0.95 (m, 12H), 0.93 (d, *J* = 6.5 Hz, 3H), 0.65 (s, 3H); **^{13}C NMR** (150.9 MHz, $CDCl_3$) δ 70.7, 61.7, 56.7, 56.1, 42.8, 40.4, 40.2, 37.5, 36.6, 35.7, 35.4, 35.2, 34.5, 31.6, 30.0, 29.0, 28.3, 26.6, 26.4, 25.9, 24.2, 23.8, 21.0, 18.6, 12.1; **HRMS** (TOF MS EI+) *m/z*: $[M]^+$ calculated for $C_{25}H_{43}ClO_3S$, 458.21160; found 458.26145.

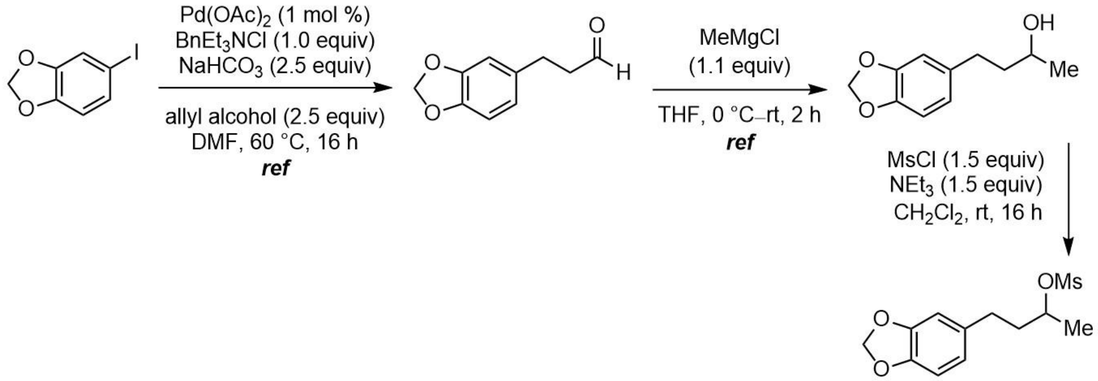

Scheme SI-11: Synthesis of Mesylate **(±)54**[15,13]

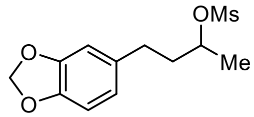

Mesylate **(±)54** was prepared according to Method F. The following amounts of reagents were used: alcohol (0.62 g, 3.2 mmol, 1.0 equiv), Et_3N (0.66 mL, 4.8 mmol, 1.5 equiv), MsCl (0.37 mL, 4.8 mmol, 1.5 equiv), and CH_2Cl_2 (16 mL, 0.20 M in substrate). The compound was purified by flash column chromatography (0–30% EtOAc/hexanes) to afford the title compound as a yellow oil (0.79 g, 2.9 mmol, 92%). **TLC R_f** = 0.4 (25% EtOAc/hexanes, CAM stain); **^{1}H NMR** (500 MHz, $CDCl_3$) δ 6.71 (d, *J* = 7.8 Hz, 1H), 6.67 (s, 1H), 6.63 (d, *J* = 7.8 Hz, 1H), 5.89 (s, 2H), 4.83−4.77 (m, 1H), 2.98 (s, 3H), 2.69−2.56 (m, 2H), 2.02−1.94 (m, 1H), 1.90−1.83 (m, 1H), 1.43 (d, *J* = 6.2 Hz, 3H). Analytical data is consistent with literature values.[11]

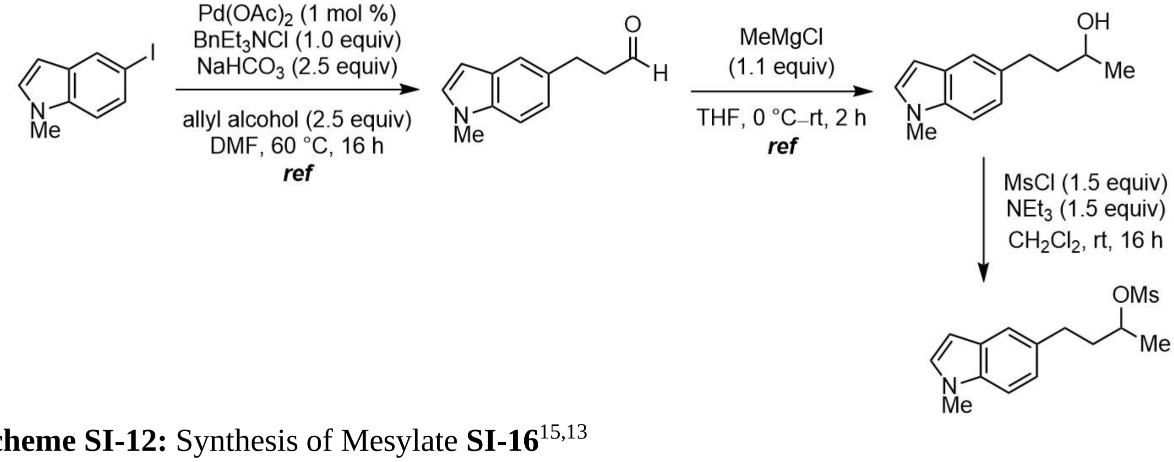

Scheme SI-12: Synthesis of Mesylate **SI-16**[15,13]

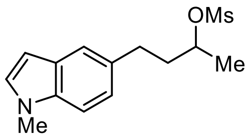

Mesylate **SI-16** was prepared according to Method F. The following amounts of reagents were used: alcohol (0.34 g, 1.70 mmol, 1.0 equiv), Et_3N (0.35 mL, 1.5 mmol, 1.5 equiv), MsCl (0.20 mL, 2.5 mmol, 1.5 equiv), and CH_2Cl_2 (8.4 mL, 0.20 M in substrate). The compound was purified by flash column chromatography (0–60% EtOAc/hexanes) to afford the title compound as a white solid (0.33 g, 1.2 mmol, 70%). **TLC R_f** = 0.5 (20% EtOAc/hexanes, CAM stain); **^{1}H NMR** (600 MHz, $CDCl_3$) δ 7.43 (s, 1H), 7.25 (d, *J* = 5.8 Hz, 1H), 7.06 (d, *J* = 8.3 Hz, 1H), 7.03 (d, *J* = 2.6 Hz, 1H), 6.42 (d, *J* = 2.8 Hz, 1H), 4.86 (sextet, *J* = 6.1 Hz, 1H), 3.77 (s, 3H), 2.99 (s, 3H), 2.88–2.77 (m, 2H), 2.14–2.08 (m, 1H), 2.00–1.94 (m, 1H), 1.47 (d, *J* = 6.2 Hz, 3H); **^{13}C NMR** (150.9 MHz, $CDCl_3$) δ 135.5, 131.4, 129.1, 128.7, 122.3, 120.0, 109.2, 100.5, 79.9, 39.1, 38.7, 32.9, 31.5, 21.3; **HRMS** (TOF MS ES+) *m/z*: $[M + Na]^+$ calculated for $C_{14}H_{19}NO_3SNa$, 304.0983; found 304.0983.

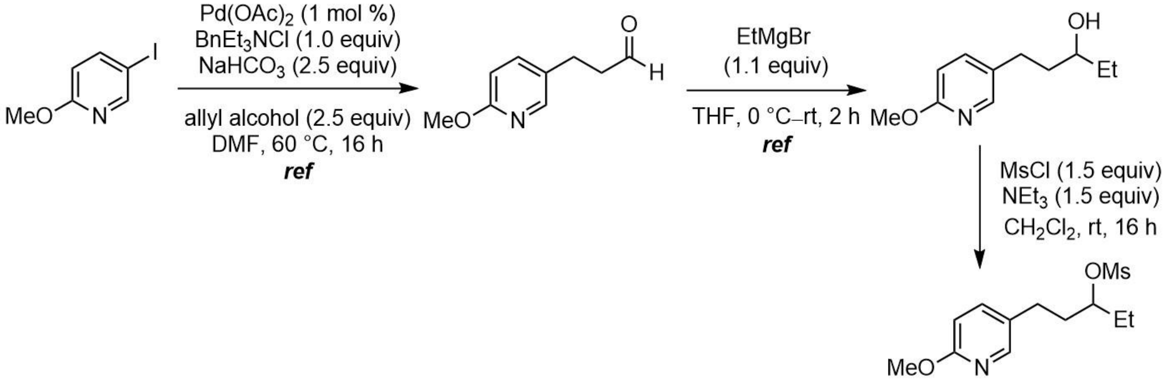

Scheme SI-13: Synthesis of Mesylate **SI-17**[15,13]

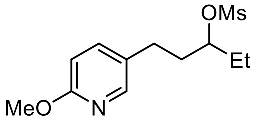

Mesylate **SI-17** was prepared according to Method F. The following amounts of reagents were used: alcohol (130 mg, 0.64 mmol, 1.0 equiv), Et_3N (0.130 mL, 0.96 mmol, 1.5 equiv), MsCl (75 µL, 0.96 mmol, 1.5 equiv), and CH_2Cl_2 (3.2 mL, 0.20 M in substrate). The compound was purified by flash column chromatography (0–50% EtOAc/hexanes) to afford the title compound as a yellow oil (120 mg, 0.44 mmol, 68%). **TLC R_f** = 0.8 (50% EtOAc/hexanes, PMA stain); **^{1}H NMR** (500 MHz, $CDCl_3$) δ 7.99 (d, *J* = 2.0 Hz, 1H), 7.44 (dd, *J* = 8.5, 2.5 Hz, 1H), 6.70 (d, *J* = 8.5 Hz, 1H), 4.74–4.69 (m, 1H), 3.91 (s, 3H), 3.02 (s, 3H), 2.73–2.59 (m, 2H), 2.02–1.91 (m, 2H), 1.81 (quintet,

J = 7.1 Hz, 2H), 1.00 (t, *J* = 7.5 Hz, 3H); **HRMS** (TOF MS ES+) *m/z*: $[M + H]^+$ calculated for $C_{12}H_{19}NO_4SH$, 274.1113; found 274.1112.

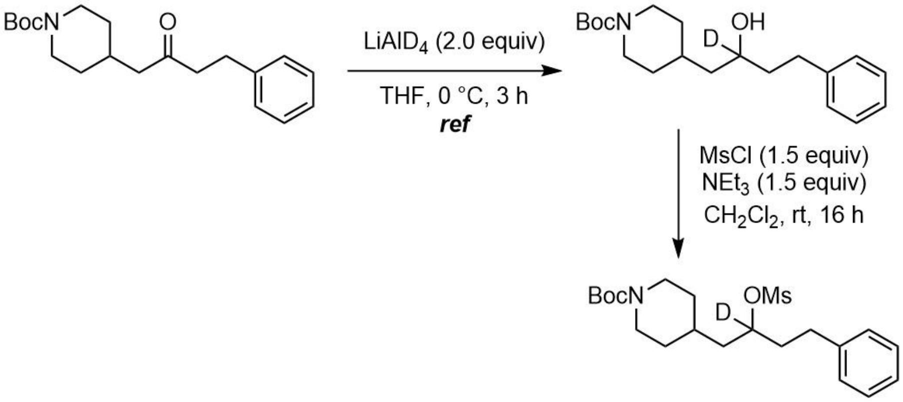

Scheme SI-14: Synthesis of Mesylate **D-SI-18**[13]

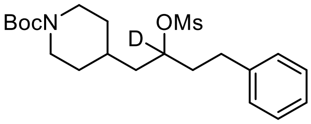

Mesylate **D-SI-18** was prepared according to Method F. The following amounts of reagents were used: alcohol (190 mg, 0.58 mmol, 1.0 equiv), Et_3N (0.12 mL, 0.87 mmol, 1.5 equiv), MsCl (68 μL, 0.87 mmol, 1.5 equiv), and CH_2Cl_2 (3.0 mL, 0.20 M in substrate). The compound was purified by flash column chromatography (0–30% EtOAc/hexanes) to afford the title compound as a cloudy white oil (0.21 g, 0.51 mmol, 87%). **TLC R$_f$** = 0.3 (30% EtOAc/hexanes, CAM stain); **^{1}H NMR** (400 MHz, $CDCl_3$) δ 7.32–7.27 (m, 2H), 7.22–7.18 (m, 3H), 4.07 (br s, 2H), 2.99 (s, 3H), 2.76–2.68 (m, 4H), 2.05–2.00 (m, 2H), 1.79–1.73 (m, 2H), 1.59–2.56 (m, 3H), 1.45 (br s, 9H), 1.26–1.06 (m, 2H). Analytical data is consistent with literature values.[13]

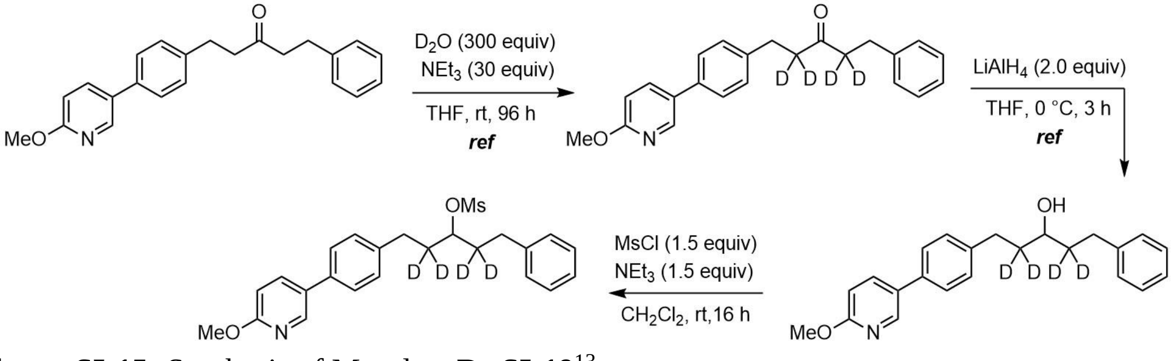

Scheme SI-15: Synthesis of Mesylate **D$_4$-SI-19**[13]

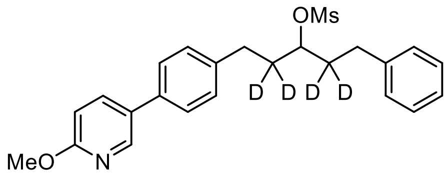

Mesylate **D_4-SI-19** was prepared according to Method F. The following amounts of reagents were used: alcohol (0.46 g, 1.3 mmol, 1.0 equiv), Et_3N (0.27 mL, 1.9 mmol, 1.5 equiv), MsCl (0.15 mL, 1.9 mmol, 1.5 equiv), and CH_2Cl_2 (6.5 mL, 0.20 M in substrate). The compound was purified by flash column chromatography (0–40% EtOAc/hexanes) to afford the title compound as a pale-yellow oil (0.53 mg, 1.2 mmol, 95%). **TLC R_f** = 0.3 (25% EtOAc/hexanes); **^{1}H NMR** (400 MHz, $CDCl_3$) δ 8.38 (br s, 1H), 7.78 (dd, J = 8.6, 1.6 Hz, 1H), 7.46 (d, J = 5.1 Hz, 2H), 7.30–7.22 (m, 4H), 7.22–7.18 (m, 3H), 6.83 (dd, J = 13.0, 1.1 Hz, 1H), 4.83 (s, 1H), 3.99 (s, 3H), 3.00 (s, 3H), 2.84–2.67 (m, 4H); **^{13}C NMR** (150.9 MHz, $CDCl_3$) δ 163.7, 144.9, 140.8, 140.1, 137.5, 136.0, 129.9, 129.2 (2C), 128.7 (2C), 128.5 (2C), 126.9 (2C), 126.4, 110.9, 82.3, 53.7, 38.9, 31.2, 30.7, carbons bearing deuterium labels are not visible; **HRMS** (TOF MS ES+) *m/z*: $[M + Na]^+$ calculated for $C_{24}H_{23}D_4NO_4SNa$, 452.1813; found 452.1812.

2. Mechanistic Studies

- Confirming Intermediacy of Alkyl Iodide

To confirm the intermediacy of alkyl iodide, iodide **3** was synthesized from mesylate **1**.

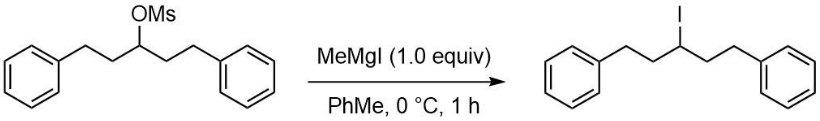

Iodide **3** was synthesized following a procedure reported by Jarvo.[11] To a flame-dried round bottom flask containing mesylate **1** (0.73 g, 2.3 mmol, 1.0 equiv) and a stir bar was added PhMe (23 mL, 0.10 M in substrate). The flask was cooled to 0 ºC and allowed to stir for 5 min before adding MeMgI (0.77 mL, 2.3 mmol, 1.0 equiv, 3.0 M in Et_2O) via syringe. The reaction was allowed to stir at 0 ºC for 1 h before quenching with NH_4Cl (aq). The aqueous layer was extracted with Et_2O (3 x 5 mL), washed with brine, and dried over Na_2SO_4 before filtering and concentrated in vacuo. The residue was purified by flash column chromatography (0–10% EtOAc/hexanes) to afford the title compound as a clear oil (0.60 g, 1.7 mmol, 75%). **TLC R_f** = 0.7 (10% EtOAc/hexanes, CAM stain); **^{1}H NMR** (500 MHz, $CDCl_3$) δ 7.29–7.25 (m, 5H), 7.21–7.17 (m, 5H), 4.05 (septet, J = 4.0 Hz, 1H), 2.91–2.85 (m, 2H), 2.73–2.67 (m, 2H), 2.24–2.17 (m, 2H), 2.04–1.97 (m, 2H).

- Stereochemical Outcome

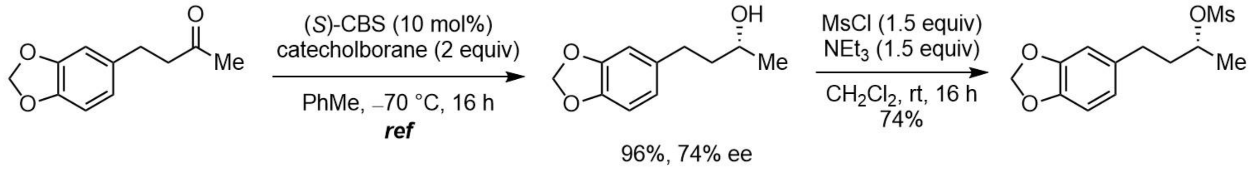

Scheme SI-16: Synthesis of mesylate ***(R)*-54**

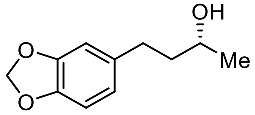

Alcohol **SI-(*R*)-20** was prepared according to a procedure reported by Jarvo.[22] Absolute configuration was assigned based on the accepted model for selectivity in CBS reductions.[23] In a glovebox, (*S*)-Me-CBS (23 mg, 82 µmol, 0.10 equiv) was added to a flame-dried round bottom flask equipped with a stir bar. The flask was capped with a septum and removed from the box. Ketone (0.16 g, 0.82 mmol, 1.0 equiv) was added to the flask as a solution in PhMe (4.0 mL). The reaction was then cooled to –70 °C and catecholborane (0.17 mL, 1.6 mmol, 2.0 equiv) was added dropwise via syringe. After stirring for 24 h at –70 °C, the reaction was warmed to ambient temperature and quenched with water. Sat. NH_4Cl (aq) was added to the reaction flask and the mixture was extracted with EtOAc (3 x 30 mL). The combined organics were washed with brine, dried over Na_2SO_4 and concentrated in vacuo. The compound was purified by flash column chromatography (0–30% EtOAc/hexanes) to afford the title compound as a pale-yellow oil (94 mg, 0.48 mmol, 58%). **TLC R_f** = 0.4 (25% EtOAc/hexanes, CAM stain); **^{1}H NMR** (600 MHz, $CDCl_3$) δ 6.72 (d, J = 7.9 Hz, 1H), 6.69 (s, 1H), 6.65 (d, J = 7.9 Hz, 1H), 5.91 (s, 2H), 3.81 (br s, 1H), 2.71–2.56 (m, 2H), 1.75–1.69 (m, 2H), 1.32 (br s, 1H), 1.22 (d, J = 6.2 Hz, 3H); $[\alpha]_D^{25}$ = + 9.6º (c 6.2 mg/1.5 mL $CHCl_3$); **SFC Analysis** (Chiralcel AD-H, 3% IPA, 2.0 mL/min, 230 nm) indicated 74% ee: t_R (major enantiomer) = 65.6 minutes, t_R (minor enantiomer) = 63.7 minutes. Analytical data is consistent with literature values.[11]

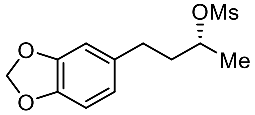

Mesylate ***(R)*-54** was prepared according to Method F. The following amounts of reagents were used: alcohol **SI-(*R*)-20** (94 mg, 0.48 mmol, 1.0 equiv), Et_3N (0.10 mL, 0.72 mmol, 1.5 equiv), MsCl (56 µL, 0.72 mmol, 1.5 equiv), and CH_2Cl_2 (2.4 mL, 0.20 M in substrate). The compound was purified by flash column chromatography (0–30% EtOAc/hexanes) to afford the title compound as a yellow oil (97 mg, 0.35 mmol, 75%). **TLC R_f** = 0.3 (25% EtOAc/hexanes, CAM stain); **^{1}H NMR** (500 MHz, $CDCl_3$) δ 6.71 (d, J = 7.8 Hz, 1H), 6.67 (s, 1H), 6.63 (d, J = 7.8 Hz, 1H), 5.89 (s, 2H), 4.83−4.77 (m, 1H), 2.98 (s, 3H), 2.69−2.56 (m, 2H), 2.02−1.94 (m, 1H), 1.90−1.83 (m, 1H), 1.43 (d, J = 6.2 Hz, 3H). Analytical data is consistent with literature values.

III. REFERENCES

[1] L. M. Chen, C. Shin, T. DeLano, A. Carretero-Cerdán, G. Gheibi, S. E. Reisman, *J. Am. Chem. Soc.*, **2024**, *146*, 29523–29530.
[2] B. Barré, L. Gonnard, R. Campagne, S. Reymond, J. Marin, P. Ciapetti, M. Brellier, A. Guérinot, J. Cossy, *Org. Lett.* **2014**, *16*, 6160– 6163.
[3] F. Liu, Z. Cheng, Y. Fang, X. Wang, L. Zhao, Z. Rong, *Org. Lett.* **2023**, *25*, 3618–3622.
[4] V. R. Bhonde, B. T. O'Neill, S. L. Buchwald, *Angew. Chem., Int. Ed.* **2016**, *55*, 1849– 1853
[5] G. A. Molander, K. M. Traister, B. T. O'Neill, *J. Org. Chem.* **2014**, *79*, 5771– 5780.
[6] J.-H. Liu, C.-T. Yang, X.-Y. Lu, Z.-Q. Zhang, L. Xu, M. Cui, X. Lu, B. Xiao, Y. Fu, L. Lu, *Chem. Eur. J.* **2014**, *20*, 15334–15338.
[7] J. Zhang, X. Wang, Y. Zhang, R. Lin, Y. Yu, L. Chen, J. Lin, International Patent WO2015090232A1, June 25, 2015.
[8] G. C. Tsui, F. Menard, M. Lautens, *Org. Lett.* **2010**, *12*, 2456– 2459.
[9] C.-F. Liu, X. Luo, H. Wang, M. J. Koh, *J. Am. Chem. Soc.* **2021**, *143*, 9498– 9506.
[10] B. C. Figula, D. L. Kane, K. Balaraman, C. Wolf, *Org. Lett.* **2022**, *24*, 8719–8723.
[11] N. Hirbawi, P. C. Lin, E. R. Jarvo, *J. Org. Chem.* **2022**, *87*, 12352–12369.
[12] C. E. Mowbray, S. Braillard, P. A. Glossop, G. A. Whitlock, R. T. Jacobs, J. Speake, B. Pandi, B. Nare, L. Maes, V. Yardley, Y. Freund, R. J. Wall, S. Carvalho, D. Bello, M. Van den Kerkhof, G. Caljon, I. H. Gilbert, V. Corpas-Lopez, I. Lukac, S. Patterson, F. Zuccotto, S. Wyllie, *J. Med. Chem.* **2021**, *64*, 16159– 16176.
[13] P. C. Lin, C. D. Wong, E. R. Jarvo, *Angew. Chem. Int. Ed.* **2024**, 63, e202403119.
[14] P.-Q. Huang, L.-X. Liu, B.-G. Wei, Y.-P. Ruan, *Org. Lett.* **2003**, *5*, 1927–1929.
[15] Y. Shang, X. Jie, K. Jonnada, S. N. Zafar, W. Su, *Nature Commun.* **2017**, *8*, 2273.
[16] H. Dirk, K. A. Newlander, US Patent US6559172B1, May 6, 2003.
[17] A. B. Sanford, T. A. Thane, T. M. McGinnis, P.-P. Chen, X. Hong, E. R. Jarvo, *J. Am. Chem. Soc.* **2020**, *142*, 5017–5023.
[18] H. Liang, M. A. Ciufolini, *Org. Lett.* **2010**, *12*, 1760–1763.
[19] T. Terai, M. Kohno, G. Boncompain, S. Sugiyama, N. Saito, R. Fujikake, T. Ueno, T. Komatsu, K. Hanaoka, T. Okabe, Y. Urano, F. Perez, T. Nagano, *J. Am. Chem. Soc.* **2015**, *137*, 10464–10467.
[20] K. Berg, P. Hegde, V. Pujari, M. Brinkmann, D. Z. Wilkins, T. Parish, D. C. Crick, C. C. Aldrich, *Eur. J. Med. Chem.* **2023**, *249*, 115125.
[21] A. Xia, P. Lv, X. Xie, Y. Liu, *Org. Lett.* **2020**, *22*, 7842– 7847.
[22] M. R. Harris, M. O. Konev, E. R. Jarvo, *J. Am. Chem. Soc.* **2014**, *136*, 7825– 7828.
[23] E. J. Corey, C. J. Helal, *Angew. Chem., Int. Ed.* **1998**, *37*, 1986–2012.

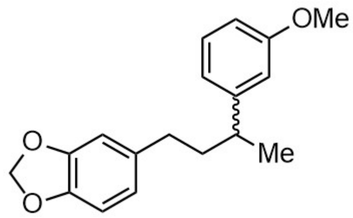

20
from (*R*)-54

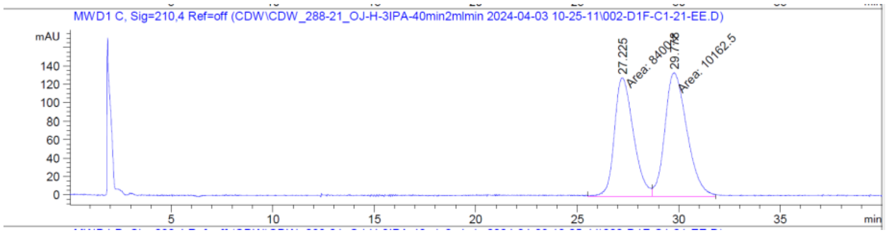

Peak #	RetTime [min]	Type	Width [min]	Area [mAU*s]	Height [mAU]	Area %
1	27.225	MF	1.0876	8400.40039	128.73239	45.2536
2	29.778	FM	1.2617	1.01625e4	134.24649	54.7464

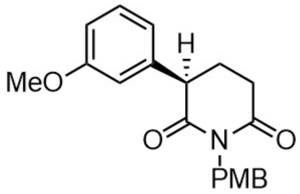

(–)-10

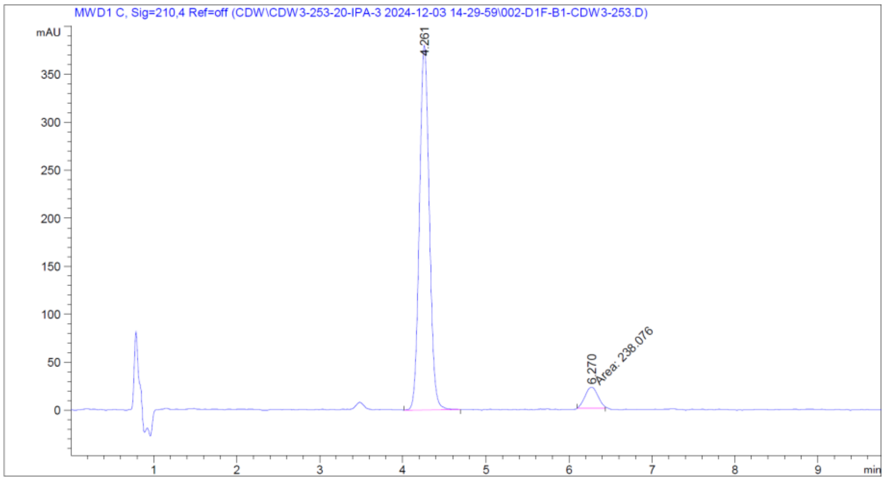

Peak #	RetTime [min]	Type	Width [min]	Area [mAU*s]	Height [mAU]	Area %
1	4.261	BV R	0.1281	3128.35425	379.96124	92.9280
2	6.270	MM	0.1811	238.07559	21.90901	7.0720

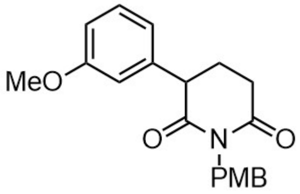

(±)-10

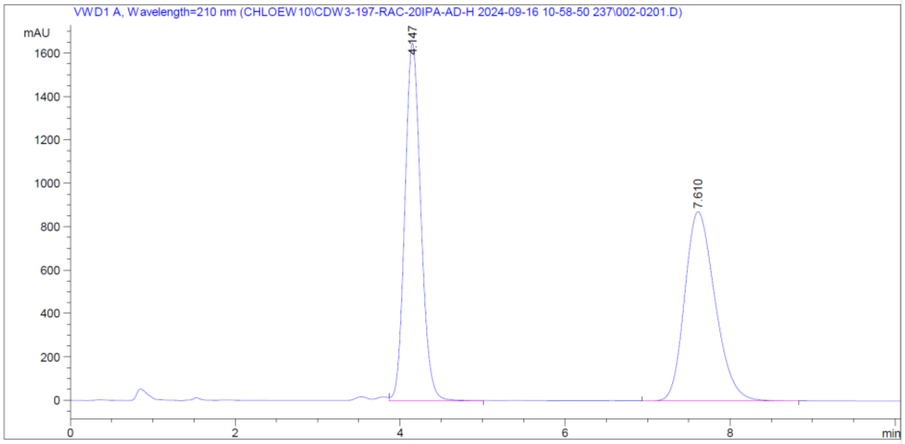

Peak #	RetTime [min]	Type	Width [min]	Area mAU *s	Height [mAU]	Area %
1	4.147	VB	0.2079	2.21567e4	1647.24023	49.9437
2	7.610	VB	0.3950	2.22066e4	871.08893	50.0563

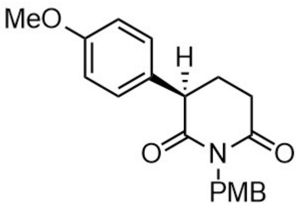

(–)-56

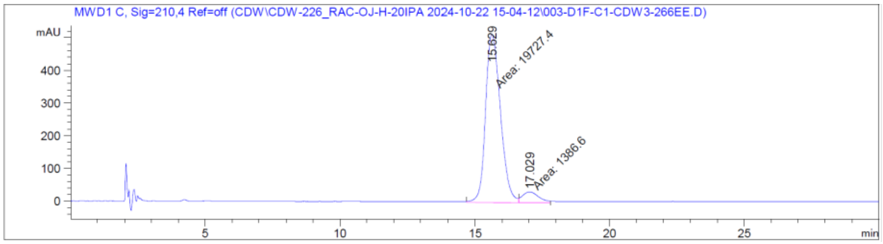

Peak #	RetTime [min]	Type	Width [min]	Area [mAU*s]	Height [mAU]	Area %
1	15.629	MF	0.6389	1.97274e4	514.58557	93.4328
2	17.029	FM	0.7007	1386.60022	32.98302	6.5672

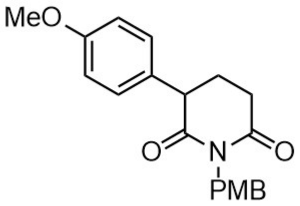

(±)-56

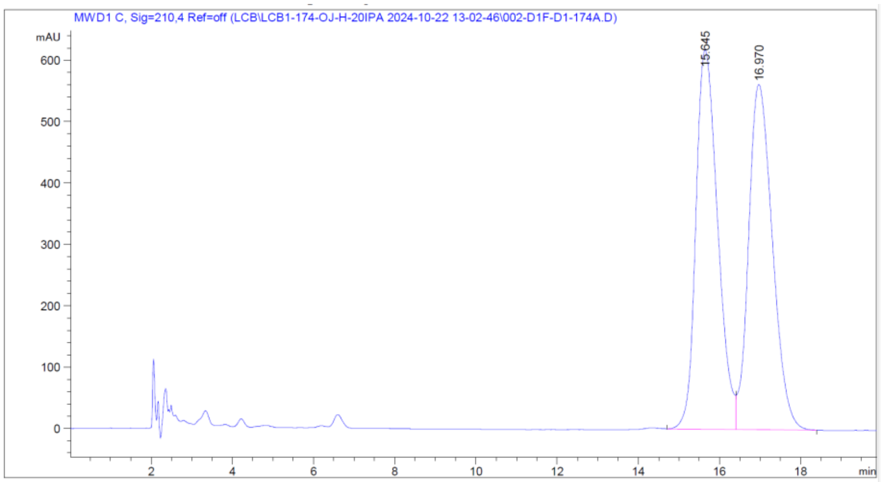

Peak #	RetTime [min]	Type	Width [min]	Area [mAU*s]	Height [mAU]	Area %
1	15.645	BV	0.5856	2.30646e4	617.75275	50.1072
2	16.970	VB	0.6345	2.29660e4	562.35004	49.8928

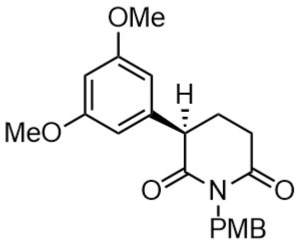

(–)-57

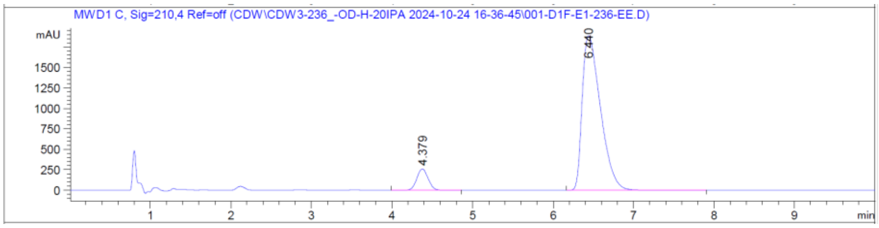

Peak #	RetTime [min]	Type	Width [min]	Area [mAU*s]	Height [mAU]	Area %
1	4.379	VV R	0.1516	2530.77051	260.00204	7.8557
2	6.440	BV R	0.2437	2.96848e4	1885.72852	92.1443

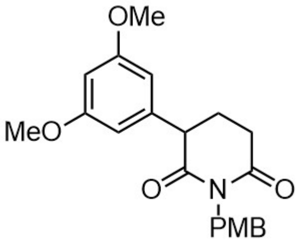

(±)-57

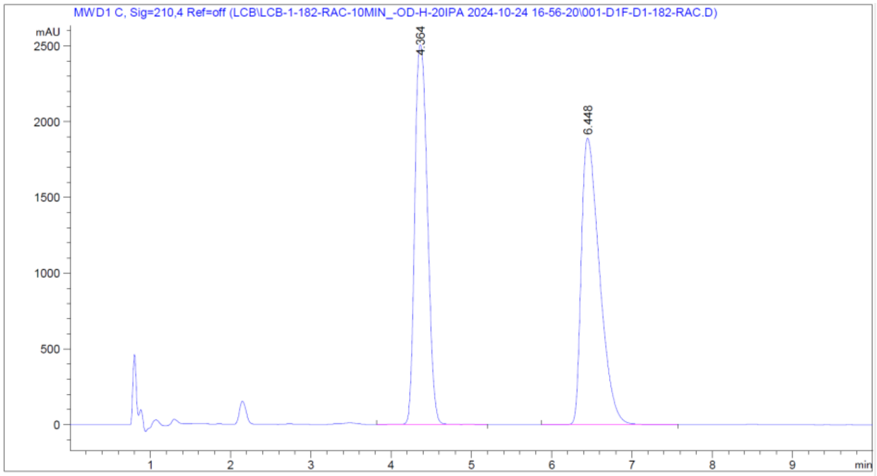

Peak #	RetTime [min]	Type	Width [min]	Area [mAU*s]	Height [mAU]	Area %
1	4.364	VV R	0.1755	2.74623e4	2508.64941	47.9520
2	6.448	VV R	0.2437	2.98081e4	1892.58704	52.0480

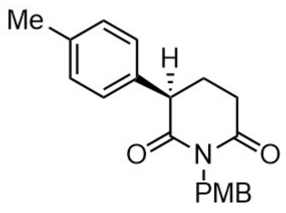

(–)-58

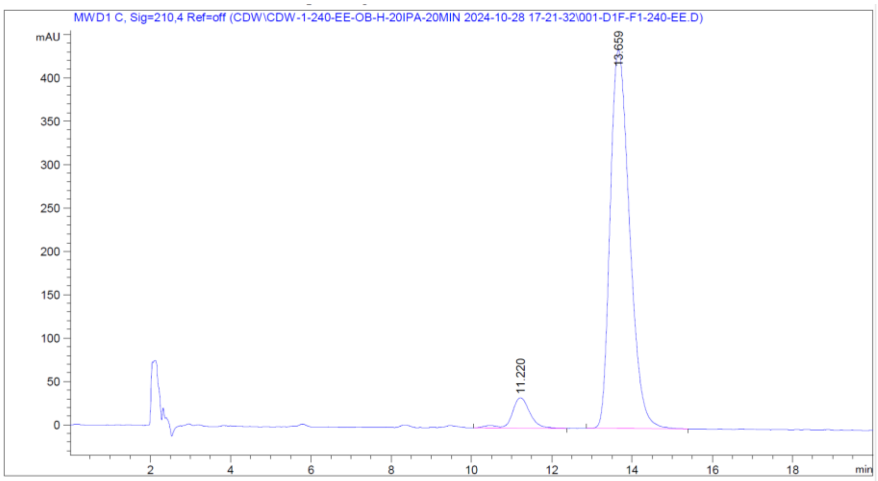

Peak #	RetTime [min]	Type	Width [min]	Area [mAU*s]	Height [mAU]	Area %
1	11.220	VB R	0.4534	1080.76123	34.87494	6.8648
2	13.659	BB	0.5279	1.46628e4	436.45773	93.1352

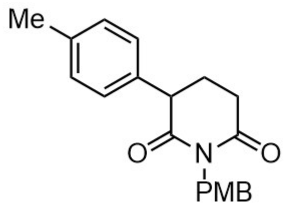

(±)-58

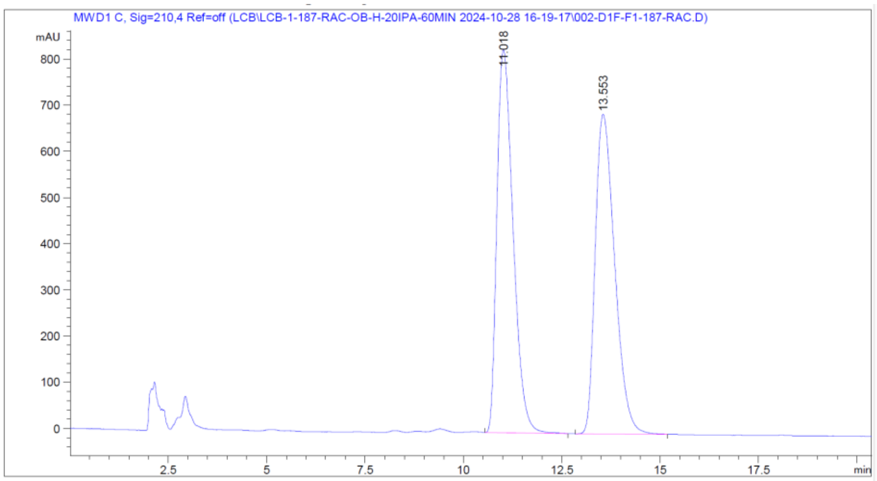

Peak #	RetTime [min]	Type	Width [min]	Area [mAU*s]	Height [mAU]	Area %
1	11.018	BV R	0.4452	2.36837e4	829.34839	49.9019
2	13.553	BB	0.5347	2.37768e4	692.20264	50.0981

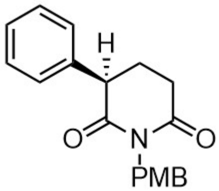

(–)-59

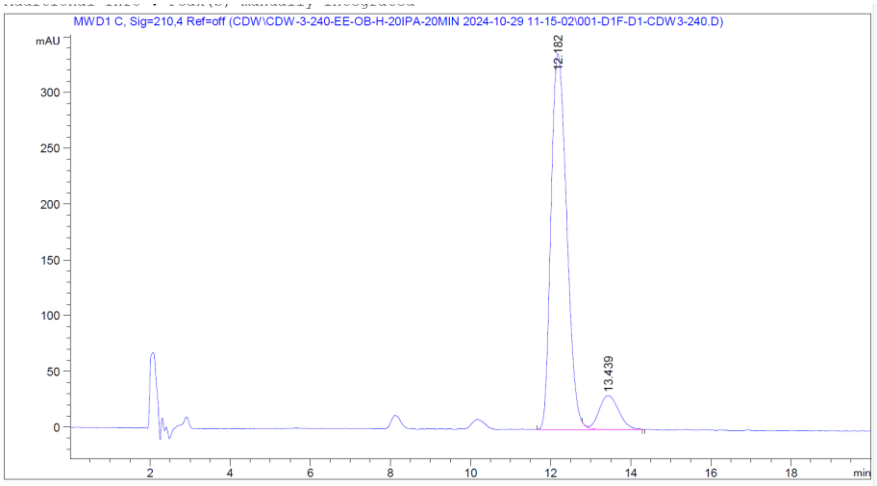

Peak #	RetTime [min]	Type	Width [min]	Area [mAU*s]	Height [mAU]	Area %
1	12.182	BV R	0.4362	9184.43262	336.73438	89.9186
2	13.439	VV E	0.4405	1029.72778	30.45454	10.0814

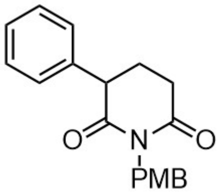

(±)-59

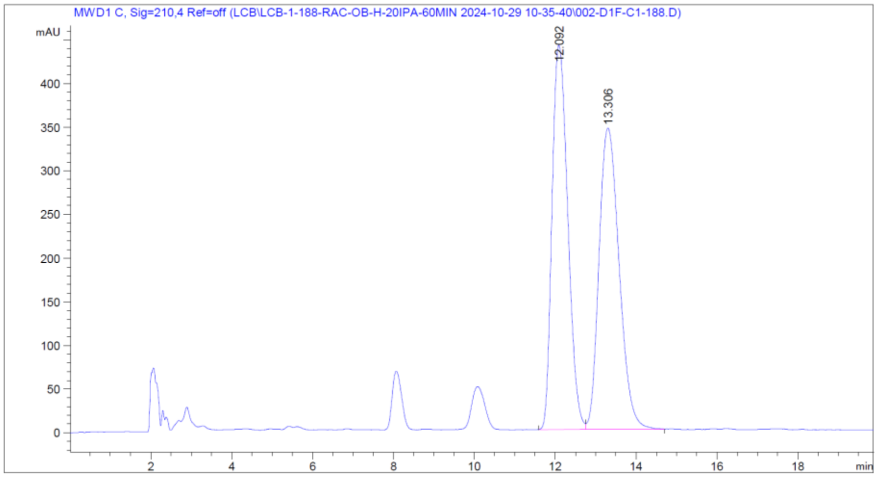

Peak #	RetTime [min]	Type	Width [min]	Area [mAU*s]	Height [mAU]	Area %
1	12.092	BV	0.4287	1.18864e4	440.69794	49.8864
2	13.306	VV R	0.5434	1.19405e4	345.34482	50.1136

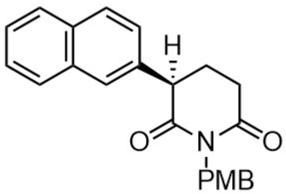

(–)-50

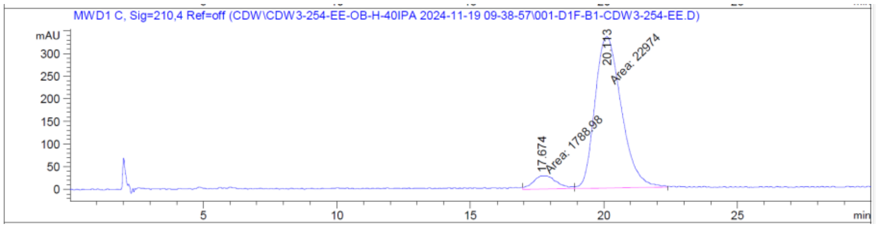

Peak #	RetTime [min]	Type	Width [min]	Area [mAU*s]	Height [mAU]	Area %
1	17.674	MF	1.0036	1788.98108	29.71016	7.2244
2	20.113	FM	1.1461	2.29740e4	334.09427	92.7756

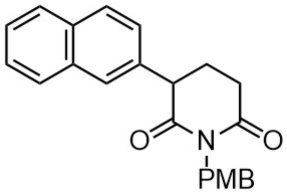

(±)-50

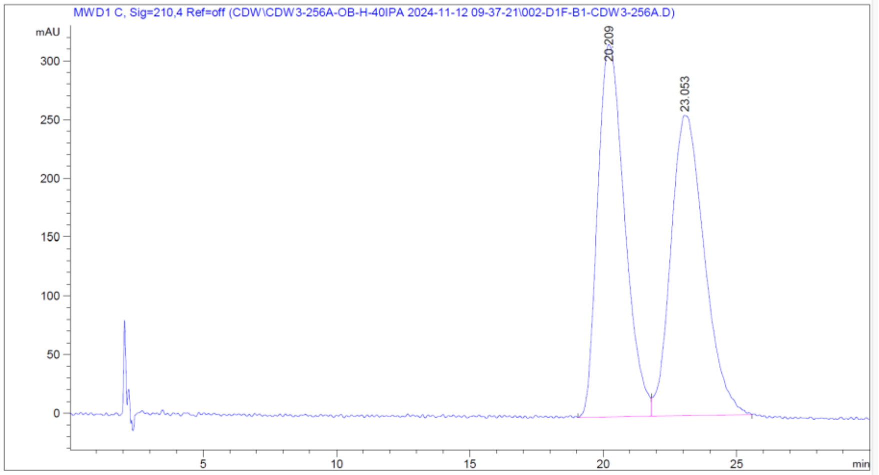

Peak #	RetTime [min]	Type	Width [min]	Area [mAU*s]	Height [mAU]	Area %
1	20.209	BV	1.0279	2.22683e4	316.84656	49.8999
2	23.053	VV R	1.0649	2.23576e4	255.50079	50.1001

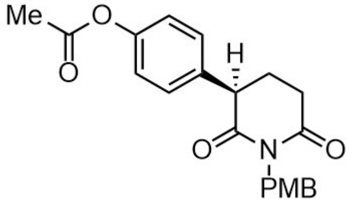

(–)-60

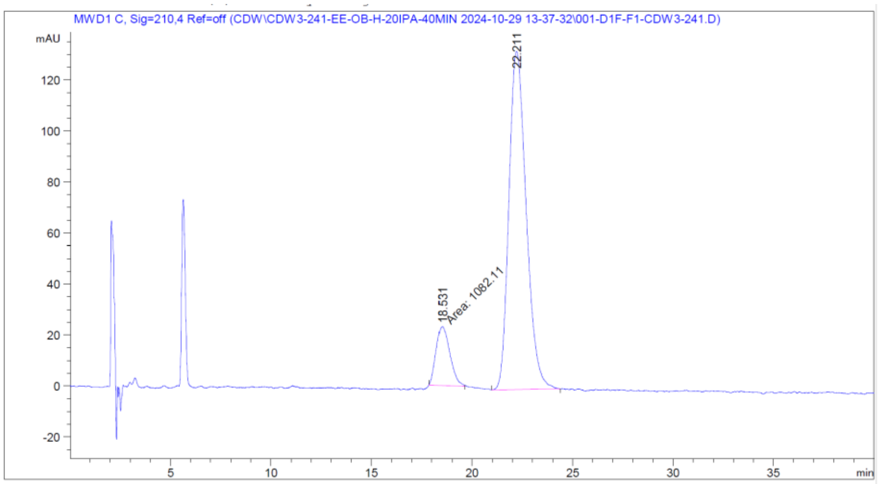

Peak #	RetTime [min]	Type	Width [min]	Area [mAU*s]	Height [mAU]	Area %
1	18.531	MM	0.7788	1082.11316	23.15890	11.9926
2	22.211	BV R	0.8669	7941.05859	132.55463	88.0074

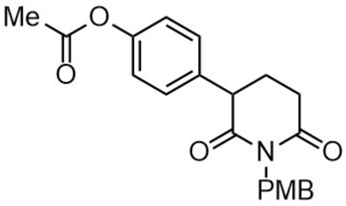

(±)-60

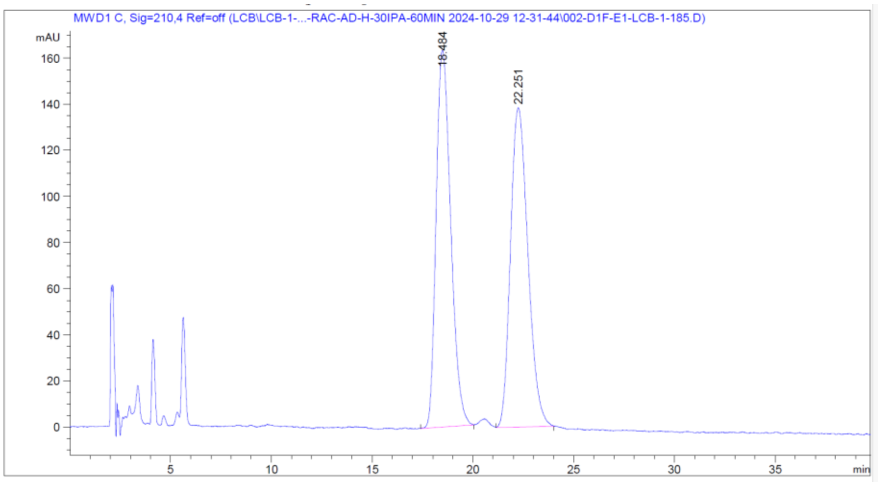

Peak #	RetTime [min]	Type	Width [min]	Area [mAU*s]	Height [mAU]	Area %
1	18.484	VB R	0.7570	8223.50977	163.43263	49.6887
2	22.251	BB	0.7515	8326.56055	138.48320	50.3113

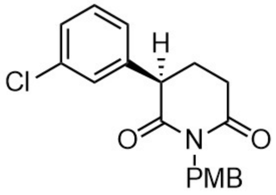

(–)-49

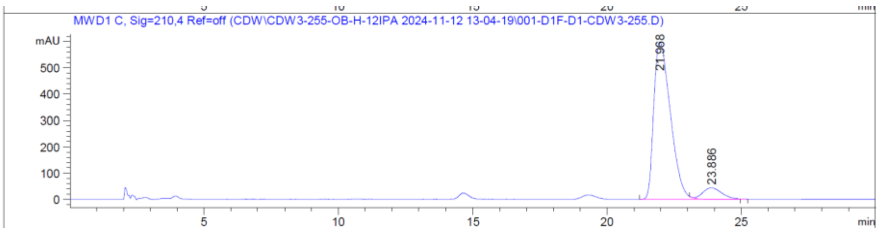

Peak #	RetTime [min]	Type	Width [min]	Area [mAU*s]	Height [mAU]	Area %
1	21.968	BV R	0.6603	2.57909e4	599.02216	92.3457
2	23.886	VV E	0.5894	2137.75122	43.19136	7.6543

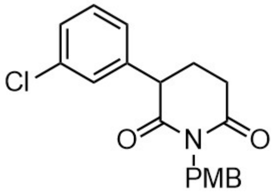

(±)-49

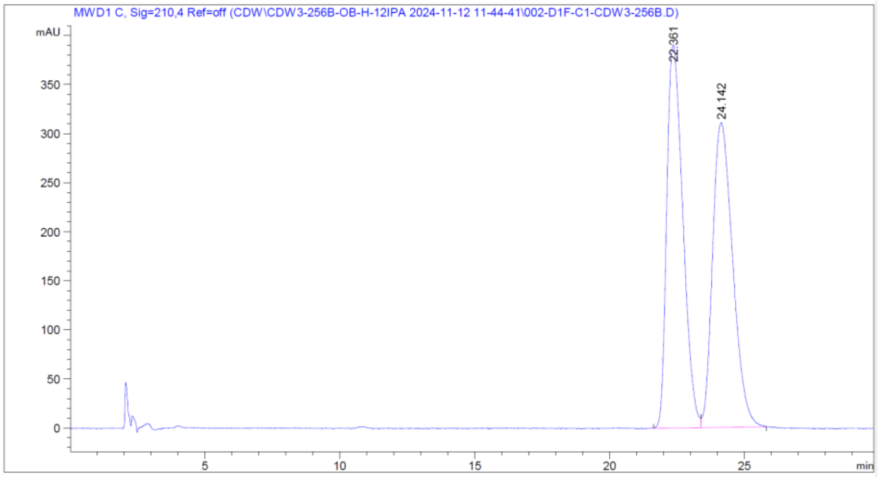

Peak #	RetTime [min]	Type	Width [min]	Area [mAU*s]	Height [mAU]	Area %
1	22.361	BV	0.6114	1.61778e4	390.42783	50.0967
2	24.142	VV R	0.7833	1.61153e4	310.61026	49.9033

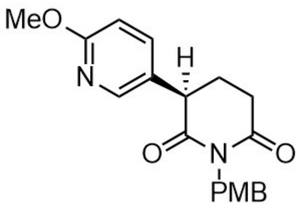

(+)-47

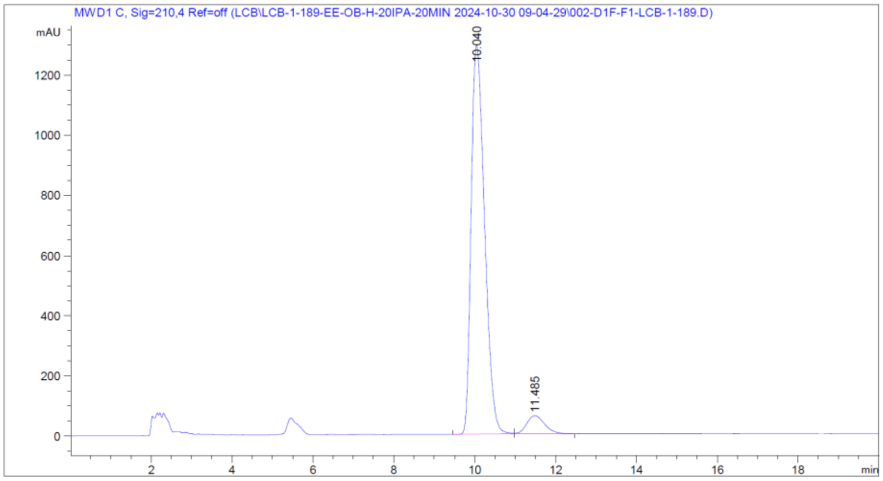

Peak #	RetTime [min]	Type	Width [min]	Area [mAU*s]	Height [mAU]	Area %
1	10.040	VV R	0.3653	2.98587e4	1293.60156	94.0683
2	11.485	VV R	0.4918	1882.81152	60.72000	5.9317

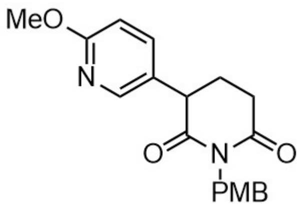

(±)-47

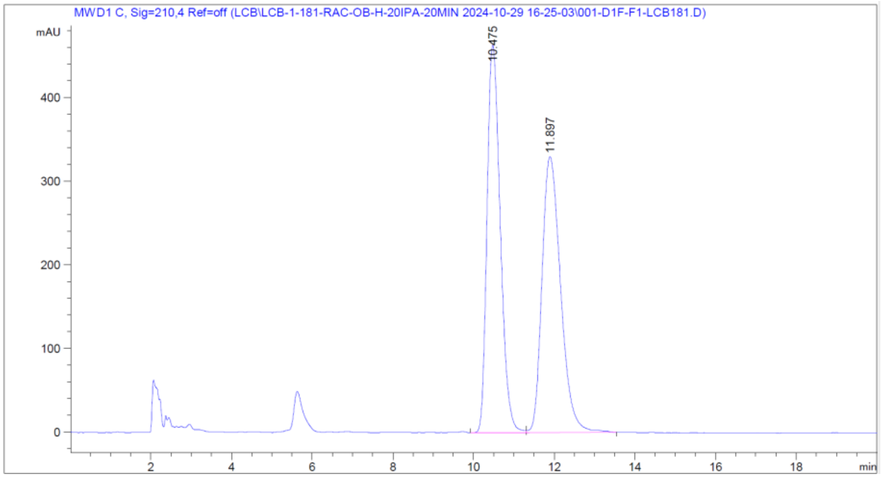

Peak #	RetTime [min]	Type	Width [min]	Area [mAU*s]	Height [mAU]	Area %
1	10.475	BV	0.3584	1.05772e4	463.22501	50.0313
2	11.897	VV R	0.4979	1.05640e4	329.66547	49.9687

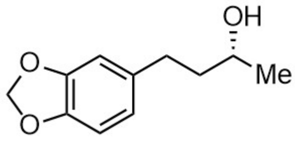

SI-(*R*)-20

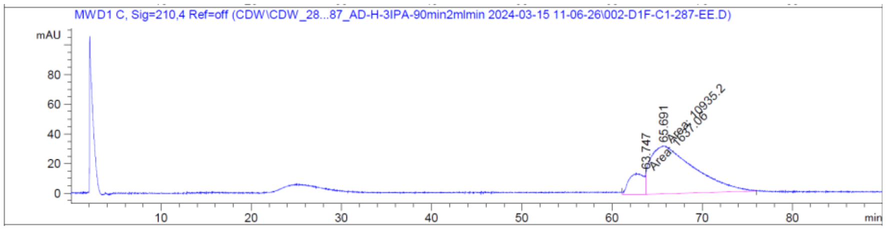

Peak #	RetTime [min]	Type	Width [min]	Area [mAU*s]	Height [mAU]	Area %
1	63.747	MM	1.8780	1637.05505	14.52871	13.0211
2	65.691	MM	5.6115	1.09352e4	32.47836	86.9789

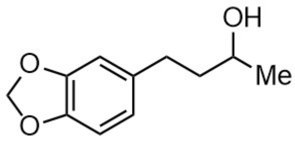

SI-(±)-20

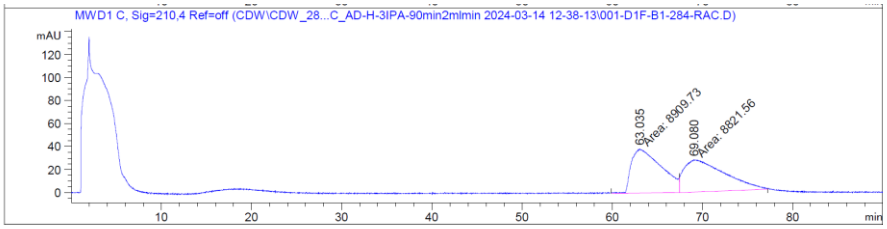

Peak #	RetTime [min]	Type	Width [min]	Area [mAU*s]	Height [mAU]	Area %
1	63.035	MM	3.8534	8909.72754	38.53618	50.2486
2	69.080	MM	5.2036	8821.56445	28.25489	49.7514

1H spectrum

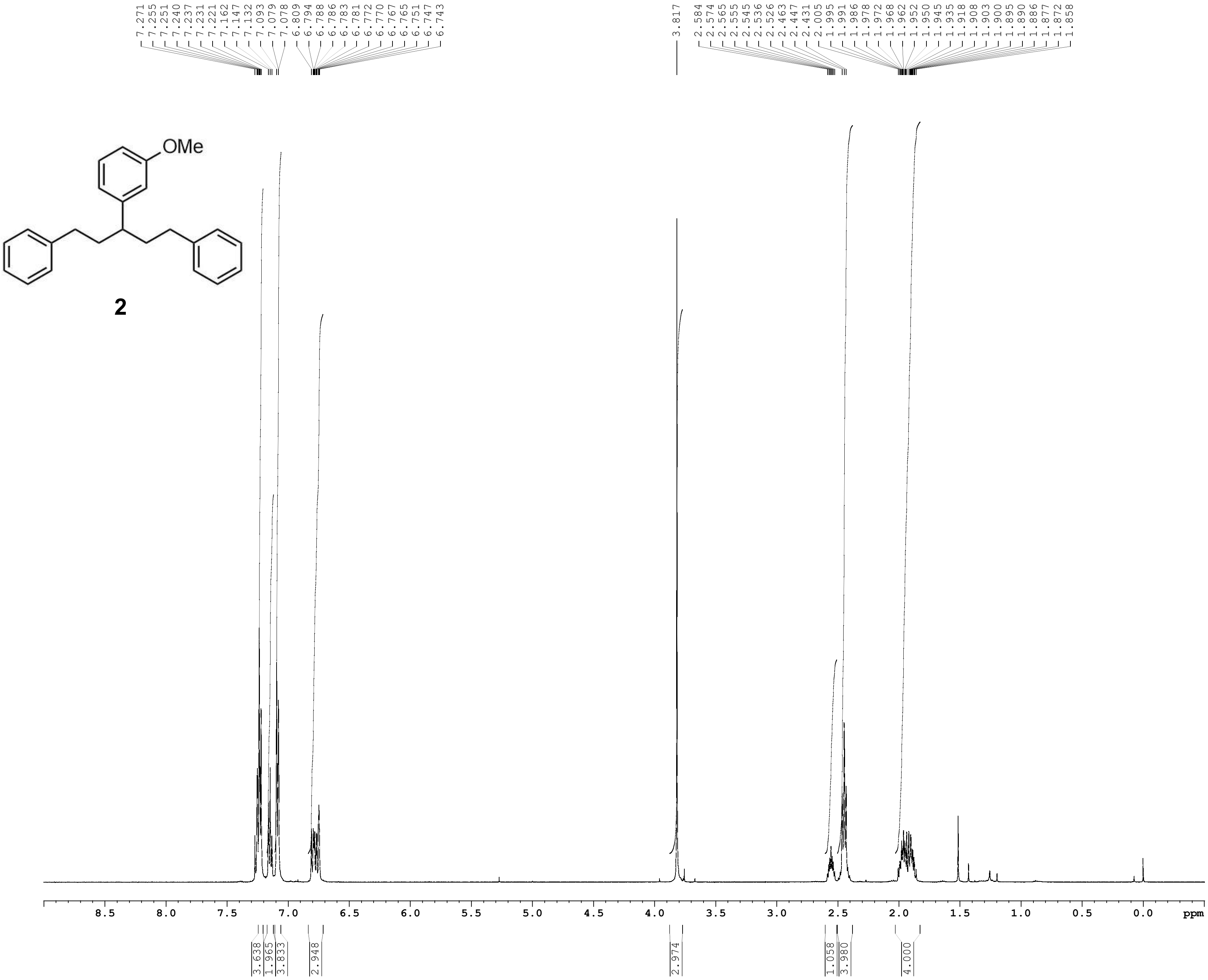

2

```
Current Data Parameters
NAME        cdw-2-suz-m
EXPNO                 1
PROCNO                1

F2 - Acquisition Parameters
Date_          20231113
Time              16.00
INSTRUM           gn500
PROBHD   5 mm broadband
PULPROG            zg30
TD                81728
SOLVENT           CDCl3
NS                    8
DS                    2
SWH            8012.820 Hz
FIDRES         0.098043 Hz
AQ            5.0998273 sec
RG                143.7
DW               62.400 usec
DE                 6.00 usec
TE                298.4 K
D1           0.10000000 sec
MCREST                0 sec
MCWRK        0.01500000 sec

======== CHANNEL f1 ========
NUC1                 1H
P1                12.00 usec
PL1               -6.00 dB
SFO1        498.5534899 MHz

F2 - Processing parameters
SI                65536
SF          498.5500410 MHz
WDW                  no
SSB                   0
LB                    0 Hz
GB                    0
PC                 1.00
```

13C spectrum with 1H decoupling

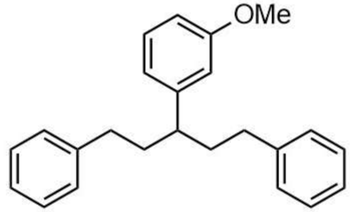

2

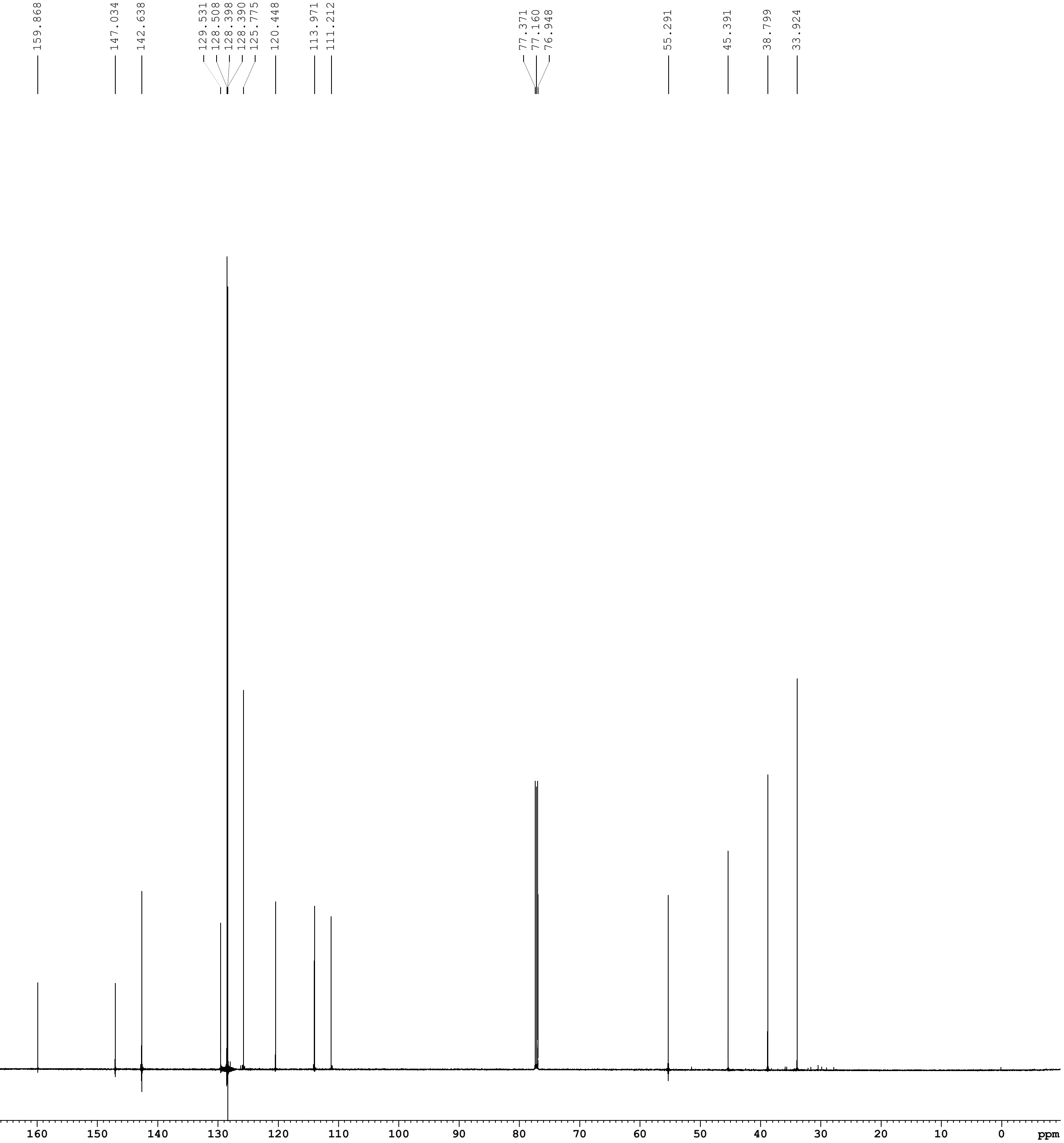


```
Current Data Parameters
NAME        cdw-suz-c13
EXPNO                 1
PROCNO                1

F2 - Acquisition Parameters
Date_          20231114
Time               8.14
INSTRUM           av600
PROBHD   5 mm CPBBO BB-
PULPROG          zgdc30
TD                65536
SOLVENT           CDCl3
NS                  520
DS                    4
SWH           36231.883 Hz
FIDRES         0.552855 Hz
AQ            0.9043968 sec
RG                 2050
DW               13.800 usec
DE                19.65 usec
TE                298.0 K
D1           0.40000001 sec
D11          0.03000000 sec
TD0                   1

======== CHANNEL f1 ========
SFO1        150.9194080 MHz
NUC1                13C
P1                10.00 usec
PLW1        68.40000153 W

======== CHANNEL f2 ========
SFO2        600.1330010 MHz
NUC2                 1H
CPDPRG[2        waltz16
PCPD2             80.00 usec
PLW2        30.00000000 W
PLW12        0.39811000 W

F2 - Processing parameters
SI                65536
SF          150.9027986 MHz
WDW                  no
SSB                   0
LB                    0 Hz
GB                    0
PC                 1.00
```

1H spectrum

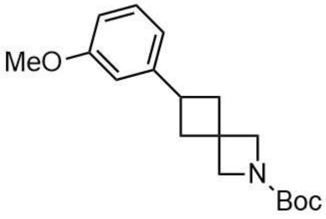

4

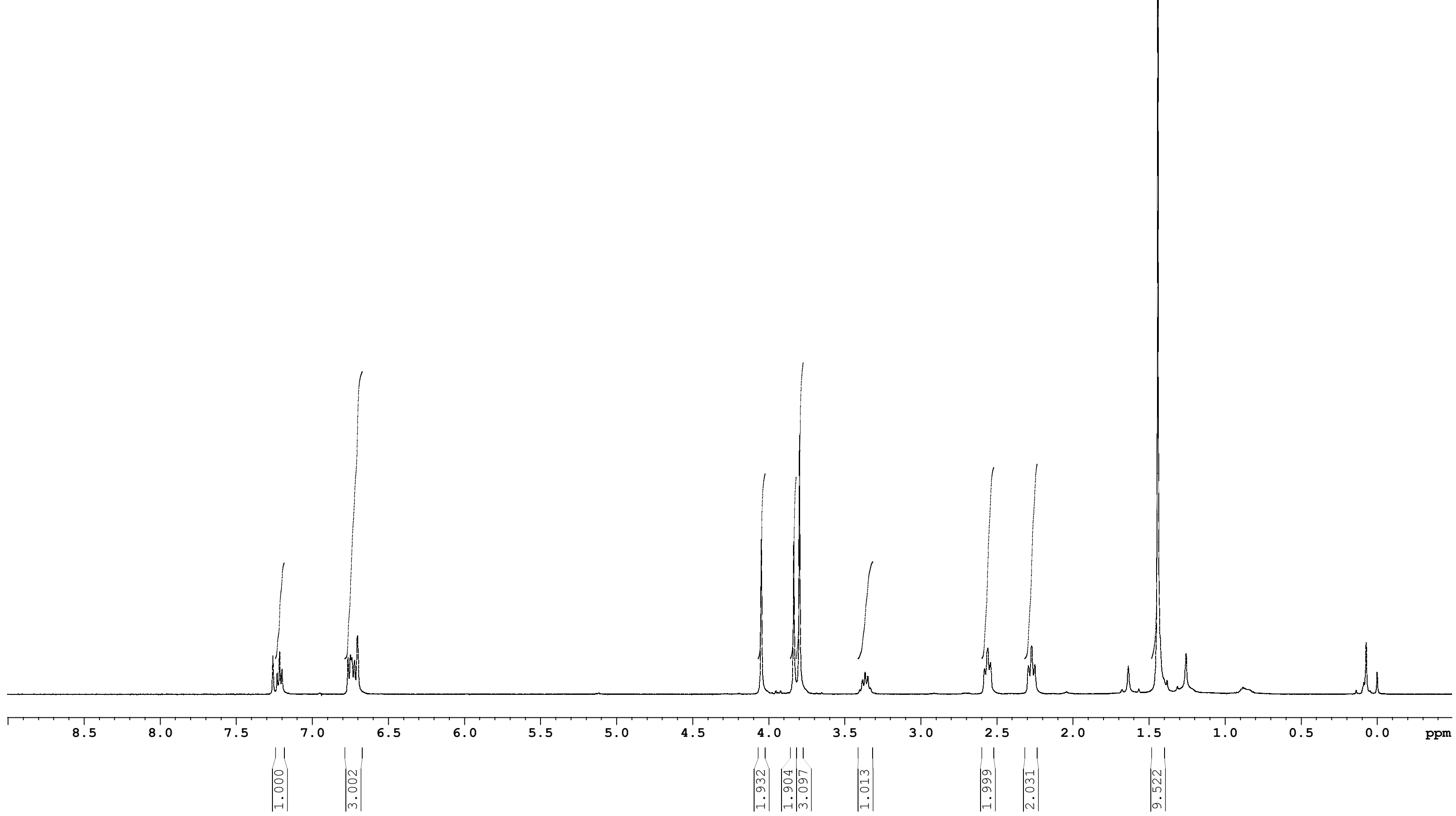


```
Current Data Parameters
NAME          cdw3-162-p
EXPNO                  1
PROCNO                 1

F2 - Acquisition Parameters
Date_           20240802
Time                7.42
INSTRUM            gn500
PROBHD   5 mm broadband
PULPROG             zg30
TD                 81728
SOLVENT            CDC13
NS                     8
DS                     2
SWH             8012.820 Hz
FIDRES          0.098043 Hz
AQ             5.0998273 sec
RG                   181
DW                62.400 usec
DE                  6.00 usec
TE                 298.0 K
D1            0.10000000 sec
MCREST                 0 sec
MCWRK         0.01500000 sec

======== CHANNEL f1 ========
NUC1                  1H
P1                 12.00 usec
PL1                -6.00 dB
SFO1        498.4534891 MHz

F2 - Processing parameters
SI                 65536
SF           498.4500310 MHz
WDW                   no
SSB                    0
LB                     0 Hz
GB                     0
PC                  1.00
```

13C spectrum with 1H decoupling

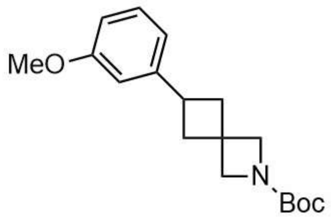

4

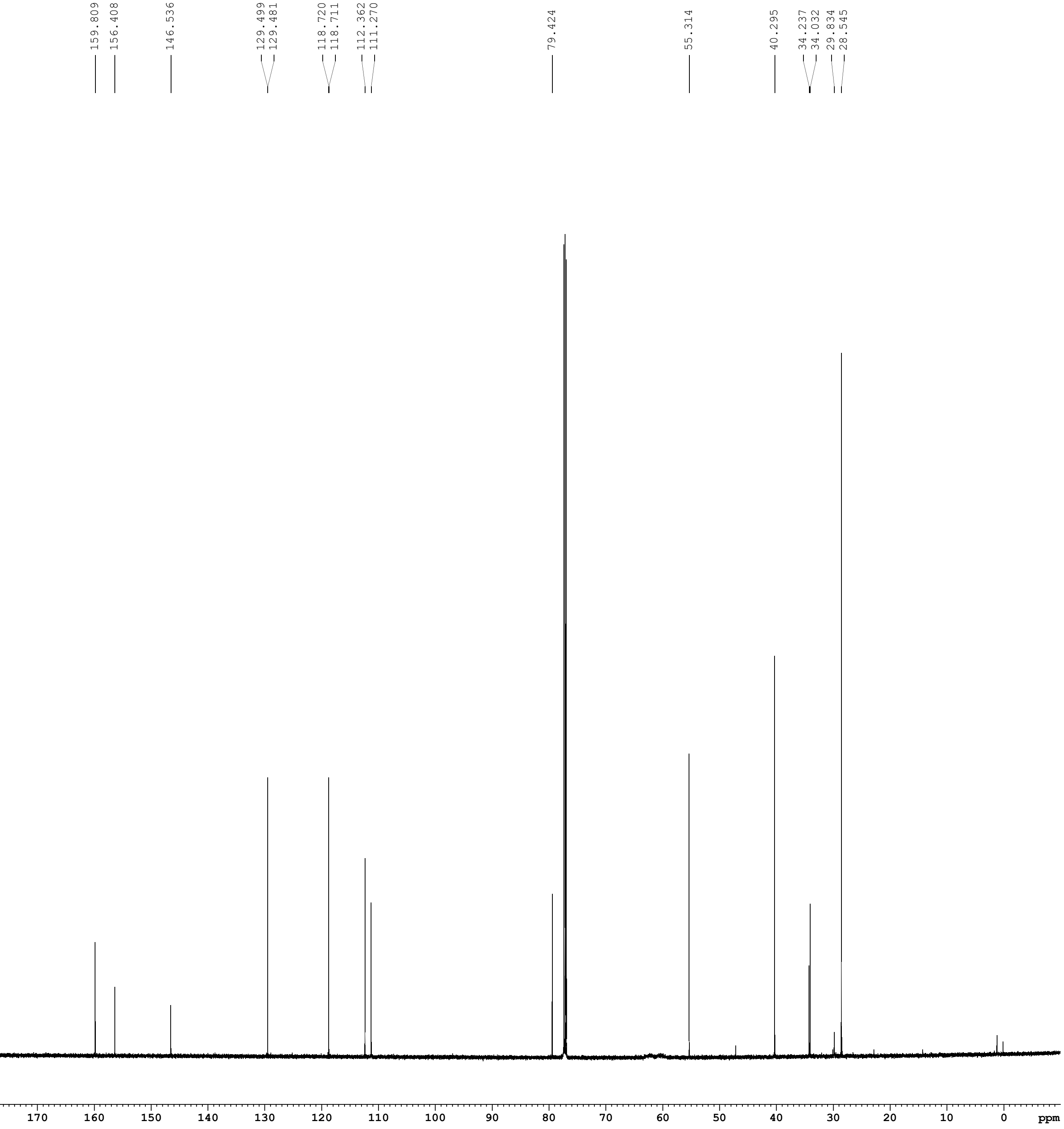

Current Data Parameters
NAME cdw3-162-c13
EXPNO 1
PROCNO 1

F2 - Acquisition Parameters
Date_ 20240803
Time 11.01
INSTRUM av600
PROBHD 5 mm CPBBO BB-
PULPROG zgdc30
TD 65536
SOLVENT CDCl3
NS 580
DS 4
SWH 36231.883 Hz
FIDRES 0.552855 Hz
AQ 0.9043968 sec
RG 2050
DW 13.800 usec
DE 19.65 usec
TE 298.0 K
D1 0.40000001 sec
D11 0.03000000 sec
TD0 1

======== CHANNEL f1 ========
SFO1 150.9194080 MHz
NUC1 13C
P1 10.00 usec
PLW1 68.40000153 W

======== CHANNEL f2 ========
SFO2 600.1330010 MHz
NUC2 1H
CPDPRG[2 waltz16
PCPD2 80.00 usec
PLW2 30.00000000 W
PLW12 0.39811000 W

F2 - Processing parameters
SI 65536
SF 150.9027955 MHz
WDW no
SSB 0
LB 0 Hz
GB 0
PC 1.00

1H spectrum

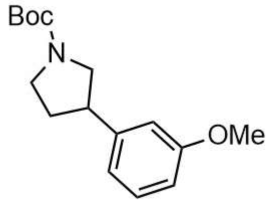

5

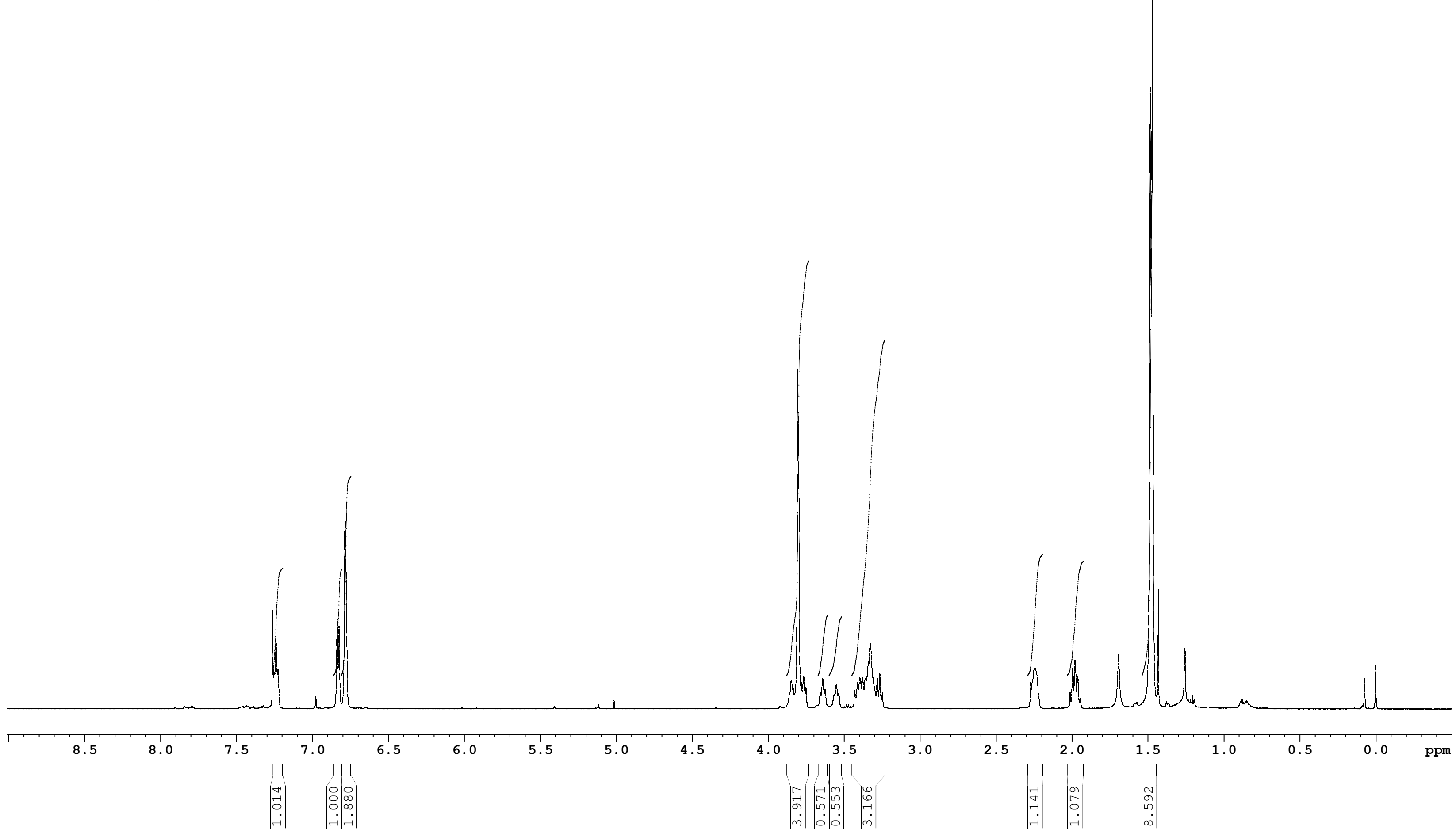

Current Data Parameters
NAME cdw-3-109-p
EXPNO 1
PROCNO 1

F2 - Acquisition Parameters
Date_ 20240612
Time 7.54
INSTRUM av600
PROBHD 5 mm CPBBO BB-
PULPROG zg30
TD 98074
SOLVENT CDCl3
NS 8
DS 2
SWH 9615.385 Hz
FIDRES 0.098042 Hz
AQ 5.0998478 sec
RG 10
DW 52.000 usec
DE 14.12 usec
TE 298.2 K
D1 0.10000000 sec
TD0 1

======== CHANNEL f1 ========
SFO1 600.1342009 MHz
NUC1 1H
P1 10.00 usec
PLW1 30.00000000 W

F2 - Processing parameters
SI 65536
SF 600.1300338 MHz
WDW no
SSB 0
LB 0 Hz
GB 0
PC 1.00

1H spectrum

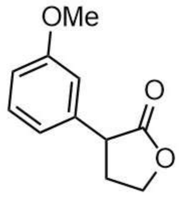

6

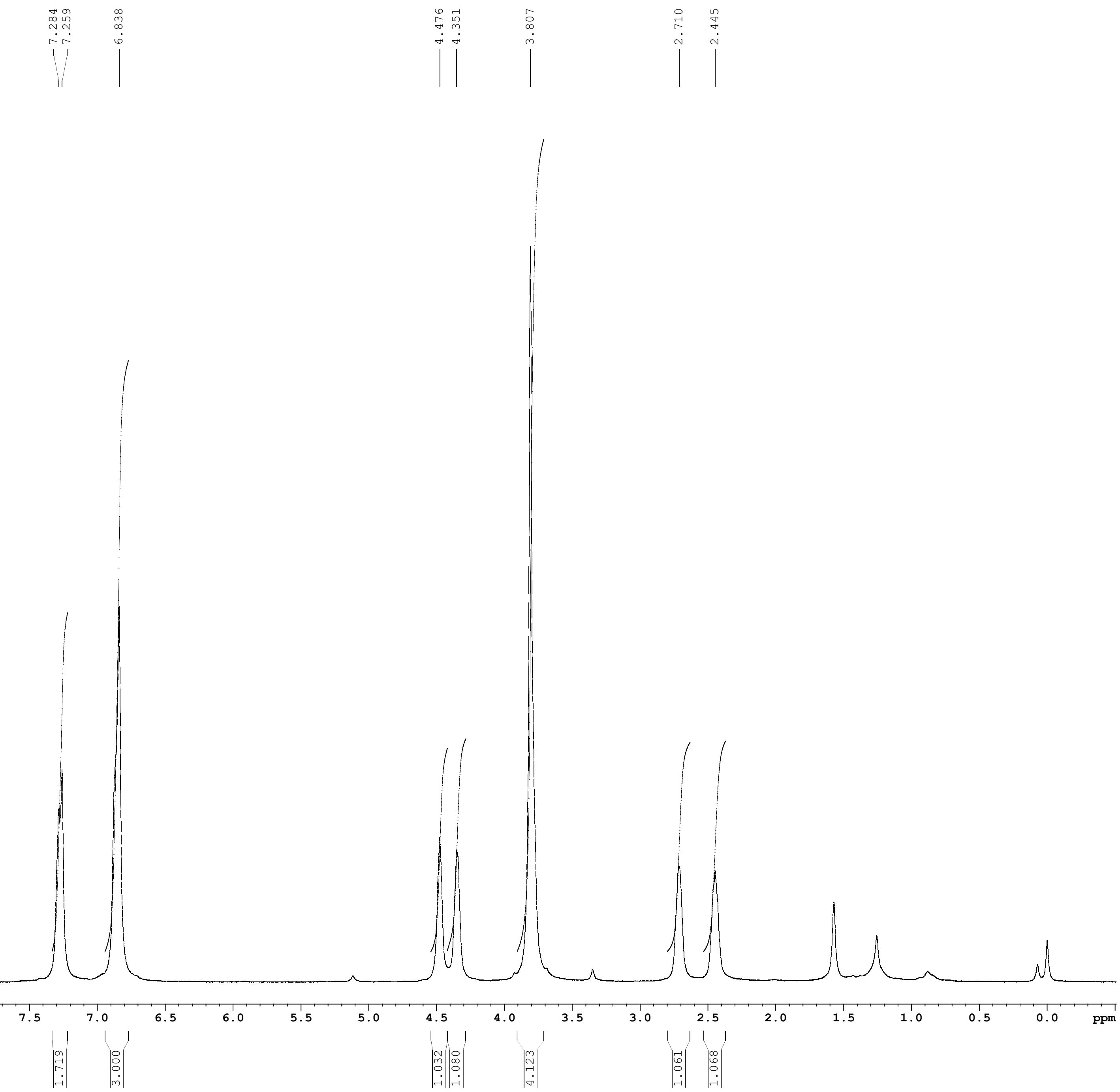


```
Current Data Parameters
NAME        cdw3-175-ge4
EXPNO                  1
PROCNO                 1

F2 - Acquisition Parameters
Date_           20240823
Time                8.26
INSTRUM            av600
PROBHD   5 mm CPBBO BB-
PULPROG             zg30
TD                 98074
SOLVENT            CDCl3
NS                     8
DS                     2
SWH             9615.385 Hz
FIDRES          0.098042 Hz
AQ             5.0998478 sec
RG                   256
DW                52.000 usec
DE                 53.12 usec
TE                 298.0 K
D1            0.10000000 sec
TD0                    1

======== CHANNEL f1 ========
SFO1         600.1342009 MHz
NUC1                  1H
P1                 10.00 usec
PLW1         30.00000000 W

F2 - Processing parameters
SI                 65536
SF           600.1300359 MHz
WDW                   no
SSB                    0
LB                     0 Hz
GB                     0
PC                  1.00
```

1H spectrum

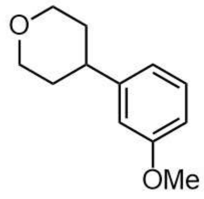

7

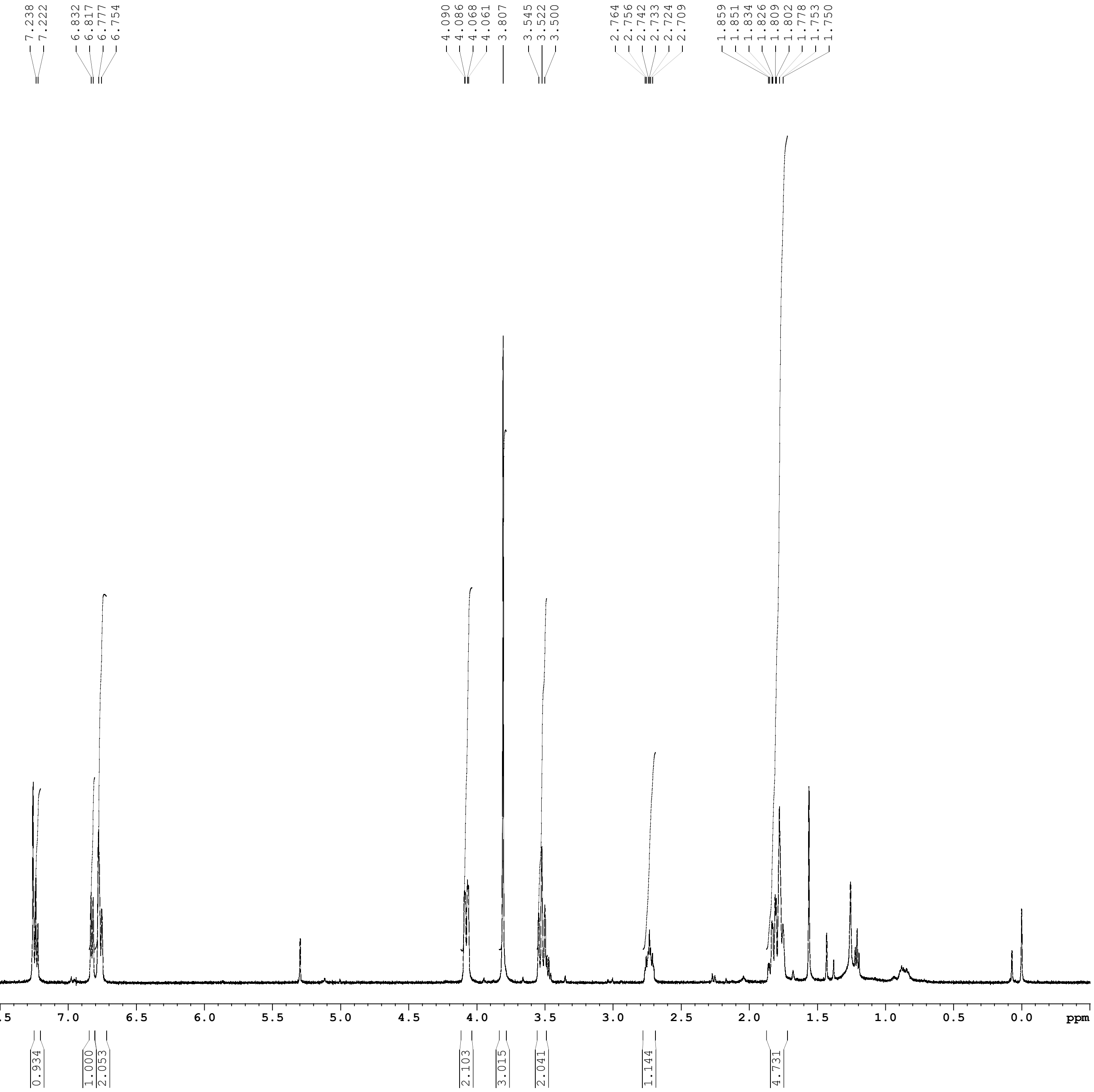


```
Current Data Parameters
NAME         cdw-3-32-p2
EXPNO                  1
PROCNO                 1

F2 - Acquisition Parameters
Date_           20240408
Time               12.47
INSTRUM            gn500
PROBHD   5 mm broadband
PULPROG             zg30
TD                 81728
SOLVENT            CDCl3
NS                     8
DS                     2
SWH             8012.820 Hz
FIDRES          0.098043 Hz
AQ             5.0998273 sec
RG                1149.4
DW                62.400 usec
DE                  6.00 usec
TE                 298.0 K
D1            0.10000000 sec
MCREST                 0 sec
MCWRK         0.01500000 sec

======== CHANNEL f1 ========
NUC1                  1H
P1                 12.00 usec
PL1                -6.00 dB
SFO1         498.4534891 MHz

F2 - Processing parameters
SI                 65536
SF           498.4500297 MHz
WDW                   no
SSB                    0
LB                     0 Hz
GB                     0
PC                  1.00
```

1H spectrum

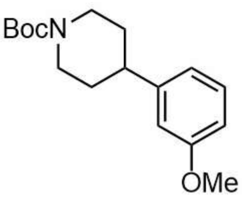

8

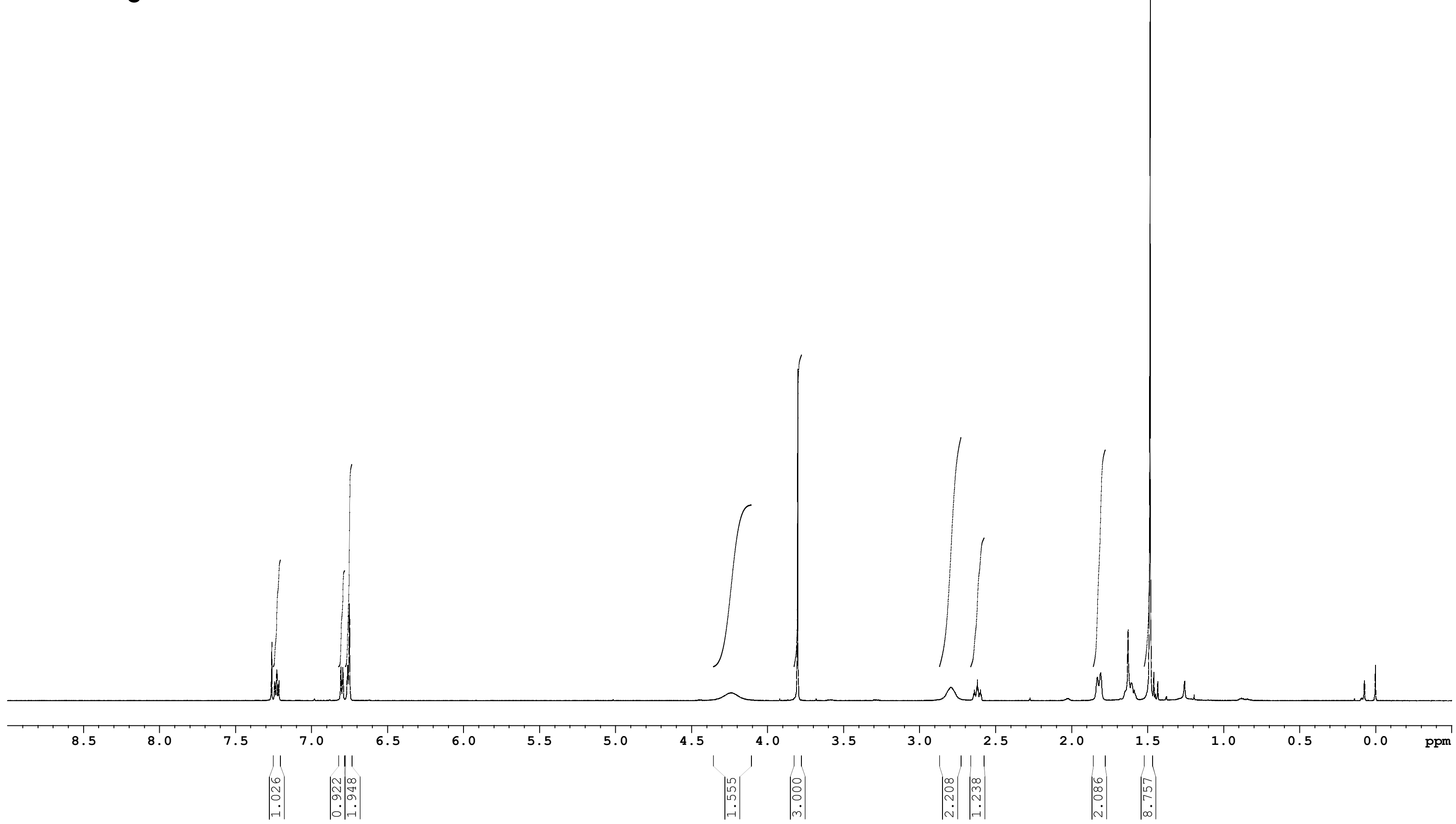


```
Current Data Parameters
NAME      cdw-2-262-p3-3
EXPNO                  1
PROCNO                 1

F2 - Acquisition Parameters
Date_           20240219
Time                9.00
INSTRUM            av600
PROBHD   5 mm CPBBO BB-
PULPROG             zg30
TD                 98074
SOLVENT            CDCl3
NS                     8
DS                     2
SWH             9615.385 Hz
FIDRES          0.098042 Hz
AQ             5.0998478 sec
RG                    10
DW                52.000 usec
DE                 14.12 usec
TE                 298.0 K
D1            0.10000000 sec
TD0                    1

======== CHANNEL f1 ========
SFO1        600.1342009 MHz
NUC1                  1H
P1                 10.00 usec
PLW1         30.00000000 W

F2 - Processing parameters
SI                 65536
SF           600.1300344 MHz
WDW                   no
SSB                    0
LB                     0 Hz
GB                     0
PC                  1.00
```

1H spectrum

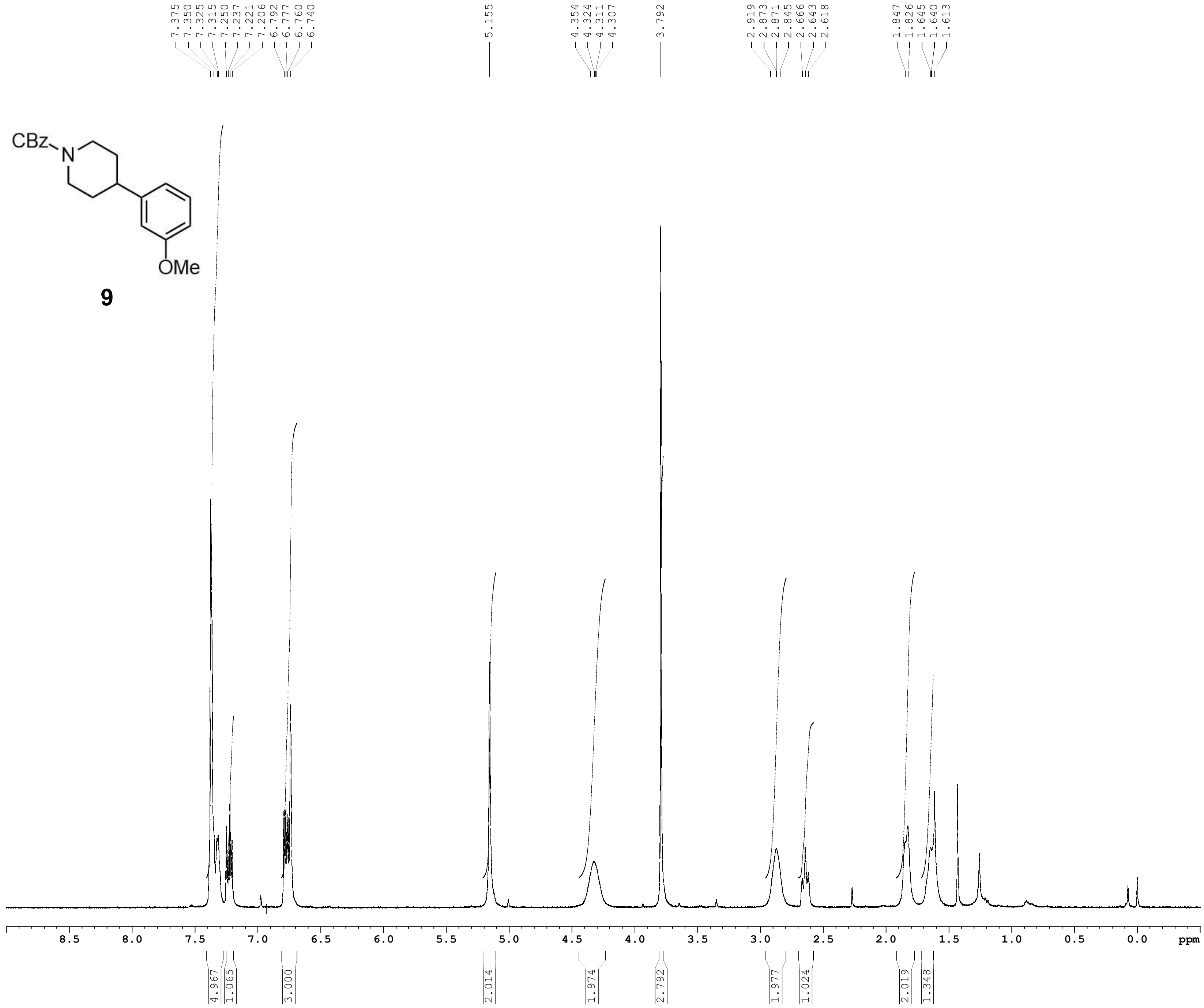


```
Current Data Parameters
NAME         cdw-3-35-p
EXPNO                 1
PROCNO                1

F2 - Acquisition Parameters
Date_          20240409
Time               9.05
INSTRUM           gn500
PROBHD   5 mm broadband
PULPROG            zg30
TD                81728
SOLVENT           CDCl3
NS                    8
DS                    2
SWH            8012.820 Hz
FIDRES         0.098043 Hz
AQ            5.0998273 sec
RG                  181
DW               62.400 usec
DE                 6.00 usec
TE                298.0 K
D1           0.10000000 sec
MCREST                0 sec
MCWRK        0.01500000 sec

======== CHANNEL f1 ========
NUC1                 1H
P1                12.00 usec
PL1               -6.00 dB
SFO1        498.4534891 MHz

F2 - Processing parameters
SI                65536
SF          498.4500336 MHz
WDW                  no
SSB                   0
LB                    0 Hz
GB                    0
PC                 1.00
```

13C spectrum with 1H decoupling

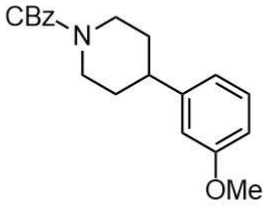

9

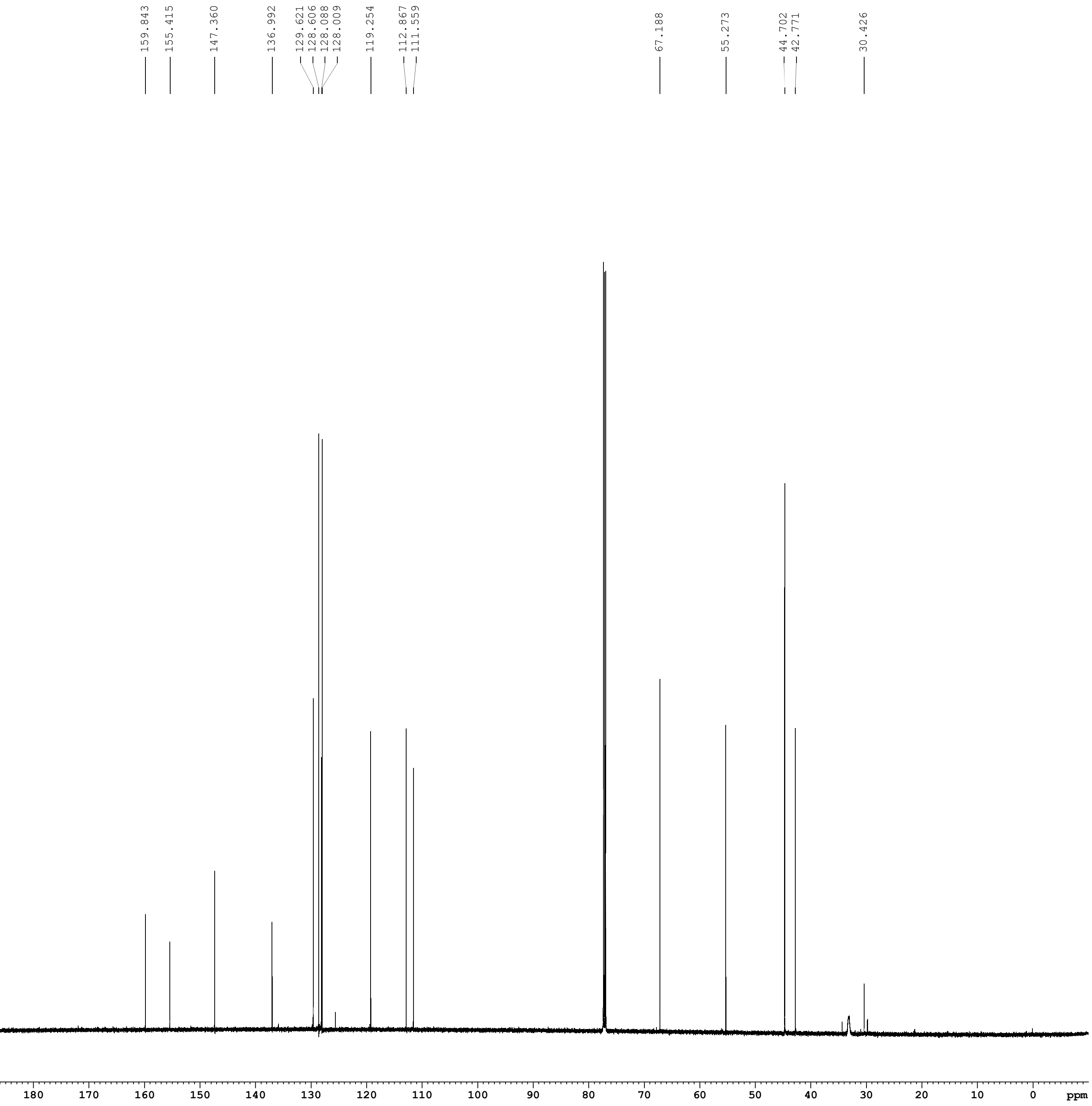


```
Current Data Parameters
NAME       cdw-3-35-c13
EXPNO                 1
PROCNO                1

F2 - Acquisition Parameters
Date_          20240410
Time               9.20
INSTRUM           av600
PROBHD   5 mm CPBBO BB-
PULPROG          zgdc30
TD                65536
SOLVENT           CDCl3
NS                  400
DS                    4
SWH           36231.883 Hz
FIDRES         0.552855 Hz
AQ            0.9043968 sec
RG                 2050
DW               13.800 usec
DE                19.65 usec
TE                298.1 K
D1           0.40000001 sec
D11          0.03000000 sec
TD0                   1

======== CHANNEL f1 ========
SFO1        150.9194080 MHz
NUC1                13C
P1                10.00 usec
PLW1        68.40000153 W

======== CHANNEL f2 ========
SFO2        600.1330010 MHz
NUC2                 1H
CPDPRG[2           waltz16
PCPD2             80.00 usec
PLW2        30.00000000 W
PLW12        0.39811000 W

F2 - Processing parameters
SI                65536
SF          150.9028009 MHz
WDW                  no
SSB                   0
LB                    0 Hz
GB                    0
PC                 1.00
```

1H spectrum

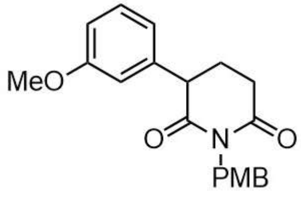

10

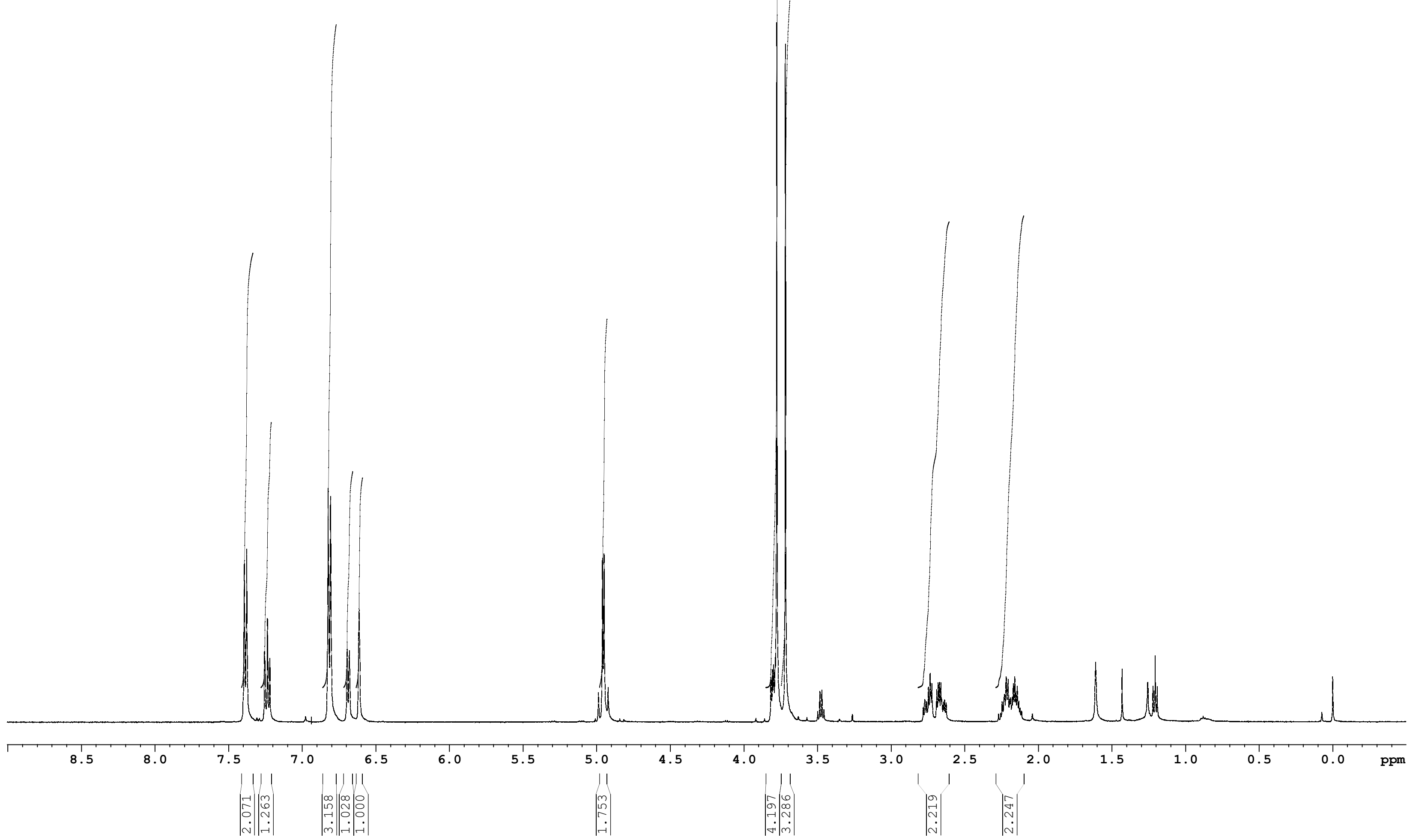

Current Data Parameters
NAME cdw3-197-p
EXPNO 1
PROCNO 1

F2 - Acquisition Parameters
Date_ 20240911
Time 11.09
INSTRUM gn500
PROBHD 5 mm broadband
PULPROG zg30
TD 81728
SOLVENT CDCl3
NS 8
DS 2
SWH 8012.820 Hz
FIDRES 0.098043 Hz
AQ 5.0998273 sec
RG 181
DW 62.400 usec
DE 6.00 usec
TE 298.0 K
D1 0.10000000 sec
MCREST 0 sec
MCWRK 0.01500000 sec

======== CHANNEL f1 ========
NUC1 1H
P1 12.00 usec
PL1 -6.00 dB
SFO1 498.4534891 MHz

F2 - Processing parameters
SI 65536
SF 498.4500317 MHz
WDW no
SSB 0
LB 0 Hz
GB 0
PC 1.00

13C spectrum with 1H decoupling

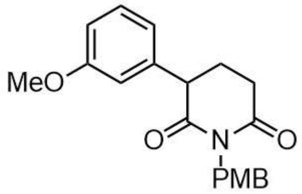

10

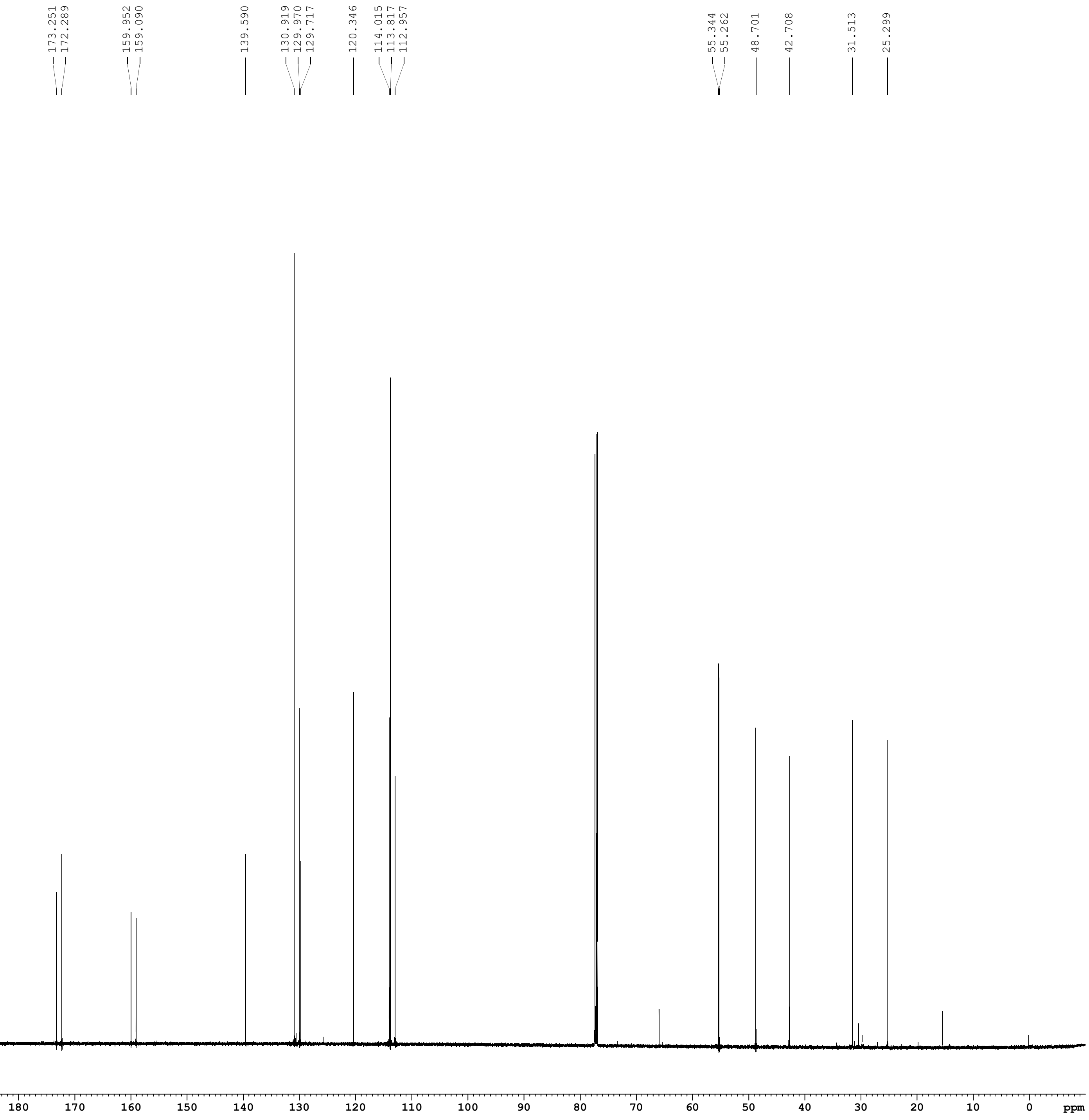


```
Current Data Parameters
NAME        cdw3-197-c13
EXPNO                  1
PROCNO                 1

F2 - Acquisition Parameters
Date_           20240912
Time               11.22
INSTRUM            av600
PROBHD   5 mm CPBBO BB-
PULPROG          zgdc30
TD                 65536
SOLVENT            CDCl3
NS                   450
DS                     4
SWH            36231.883 Hz
FIDRES          0.552855 Hz
AQ             0.9043968 sec
RG                  2050
DW                13.800 usec
DE                 19.65 usec
TE                 298.1 K
D1            0.40000001 sec
D11           0.03000000 sec
TD0                    1

======== CHANNEL f1 ========
SFO1         150.9194080 MHz
NUC1                 13C
P1                 10.00 usec
PLW1         68.40000153 W

======== CHANNEL f2 ========
SFO2         600.1330010 MHz
NUC2                  1H
CPDPRG[2          waltz16
PCPD2              80.00 usec
PLW2         30.00000000 W
PLW12         0.39811000 W

F2 - Processing parameters
SI                 65536
SF           150.9027986 MHz
WDW                   no
SSB                    0
LB                     0 Hz
GB                     0
PC                  1.00
```

1H spectrum

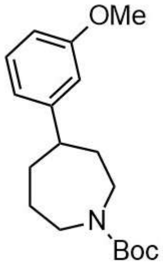

11

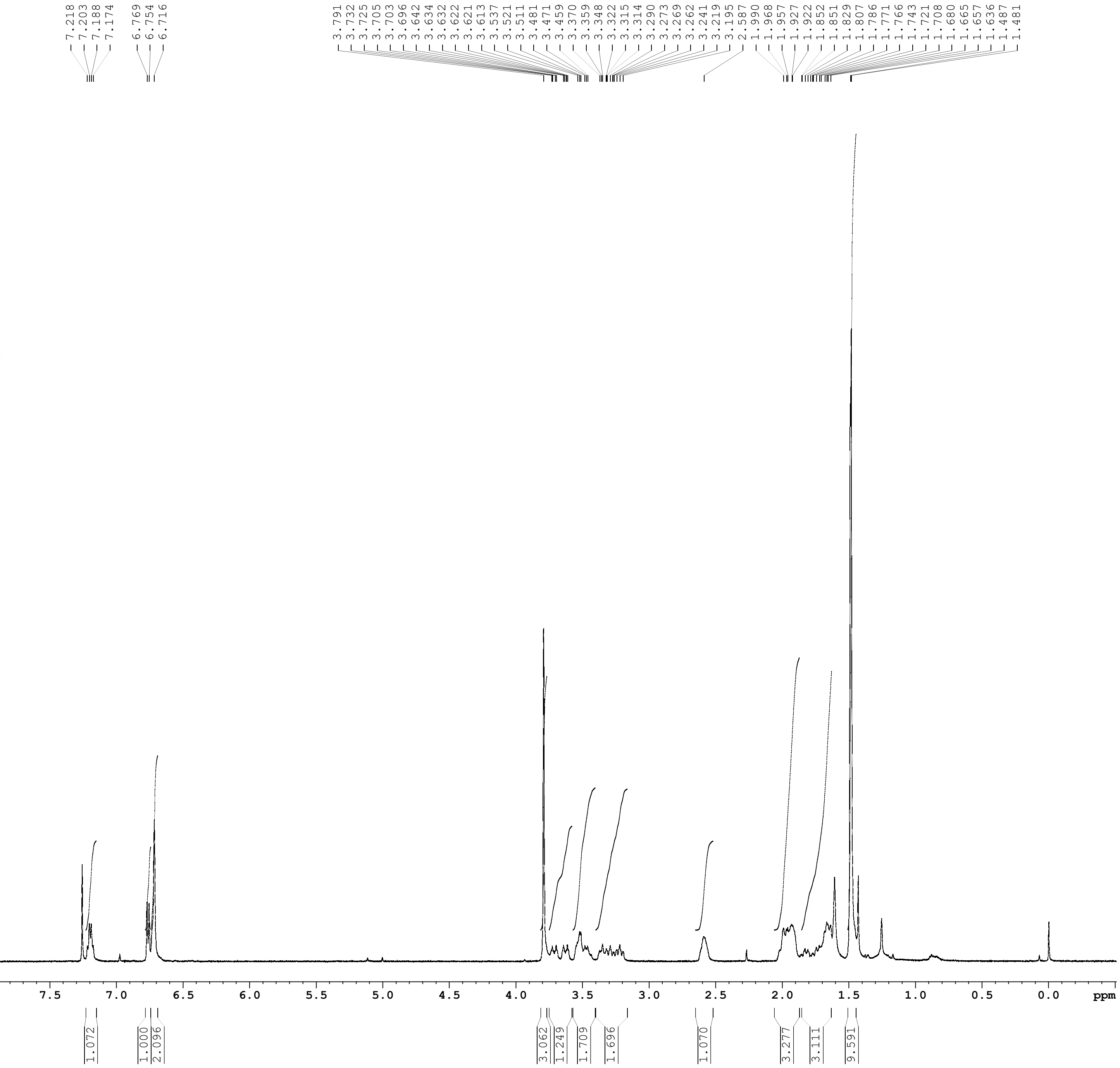


```
Current Data Parameters
NAME          cdw-3-140-p
EXPNO                   1
PROCNO                  1

F2 - Acquisition Parameters
Date_            20240709
Time                 8.47
INSTRUM             gn500
PROBHD   5 mm broadband
PULPROG              zg30
TD                  81728
SOLVENT             CDCl3
NS                      8
DS                      2
SWH              8012.820 Hz
FIDRES           0.098043 Hz
AQ              5.0998273 sec
RG                  812.7
DW                 62.400 usec
DE                   6.00 usec
TE                  298.0 K
D1             0.10000000 sec
MCREST                  0 sec
MCWRK          0.01500000 sec

======== CHANNEL f1 ========
NUC1                   1H
P1                  12.00 usec
PL1                 -6.00 dB
SFO1         498.4534891 MHz

F2 - Processing parameters
SI                  65536
SF           498.4500314 MHz
WDW                    no
SSB                     0
LB                      0 Hz
GB                      0
PC                   1.00
```

13C spectrum with 1H decoupling

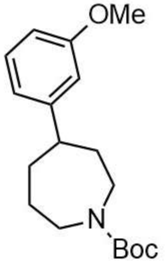

11

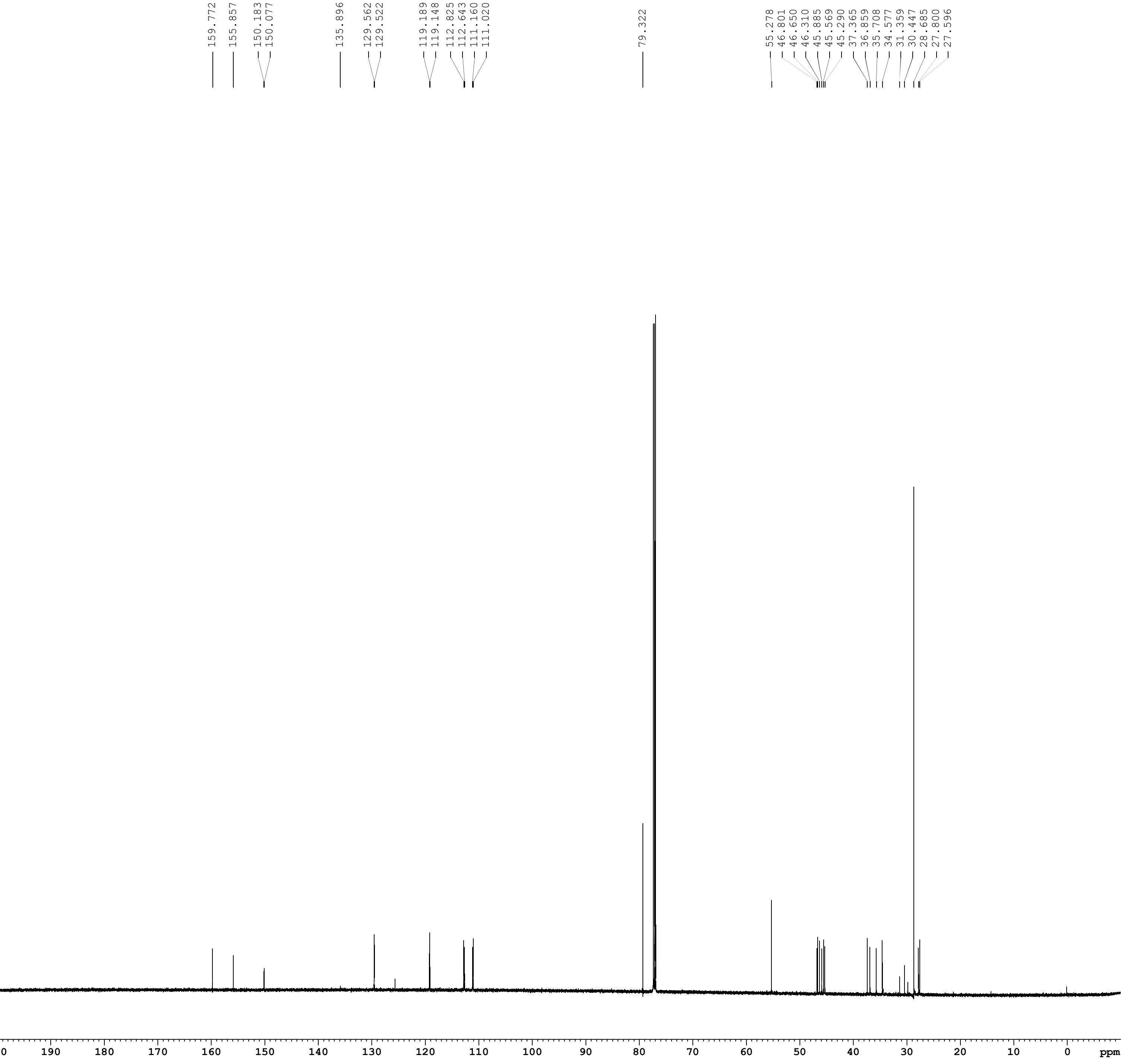


```
Current Data Parameters
NAME        cdw3-140-c13
EXPNO                  1
PROCNO                 1

F2 - Acquisition Parameters
Date_           20240712
Time               11.14
INSTRUM            av600
PROBHD   5 mm CPBBO BB-
PULPROG          zgdc30
TD                 65536
SOLVENT            CDCl3
NS                   710
DS                     4
SWH           36231.883 Hz
FIDRES         0.552855 Hz
AQ            0.9043968 sec
RG                  2050
DW                13.800 usec
DE                 19.65 usec
TE                 297.9 K
D1            0.40000001 sec
D11           0.03000000 sec
TD0                    1

======== CHANNEL f1 ========
SFO1        150.9194080 MHz
NUC1                 13C
P1                 10.00 usec
PLW1         68.40000153 W

======== CHANNEL f2 ========
SFO2        600.1330010 MHz
NUC2                  1H
CPDPRG[2         waltz16
PCPD2              80.00 usec
PLW2         30.00000000 W
PLW12         0.39811000 W

F2 - Processing parameters
SI                 65536
SF          150.9027960 MHz
WDW                   no
SSB                    0
LB                     0 Hz
GB                     0
PC                  1.00
```

1H spectrum

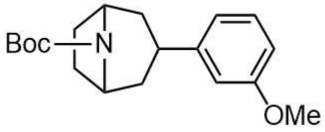

12

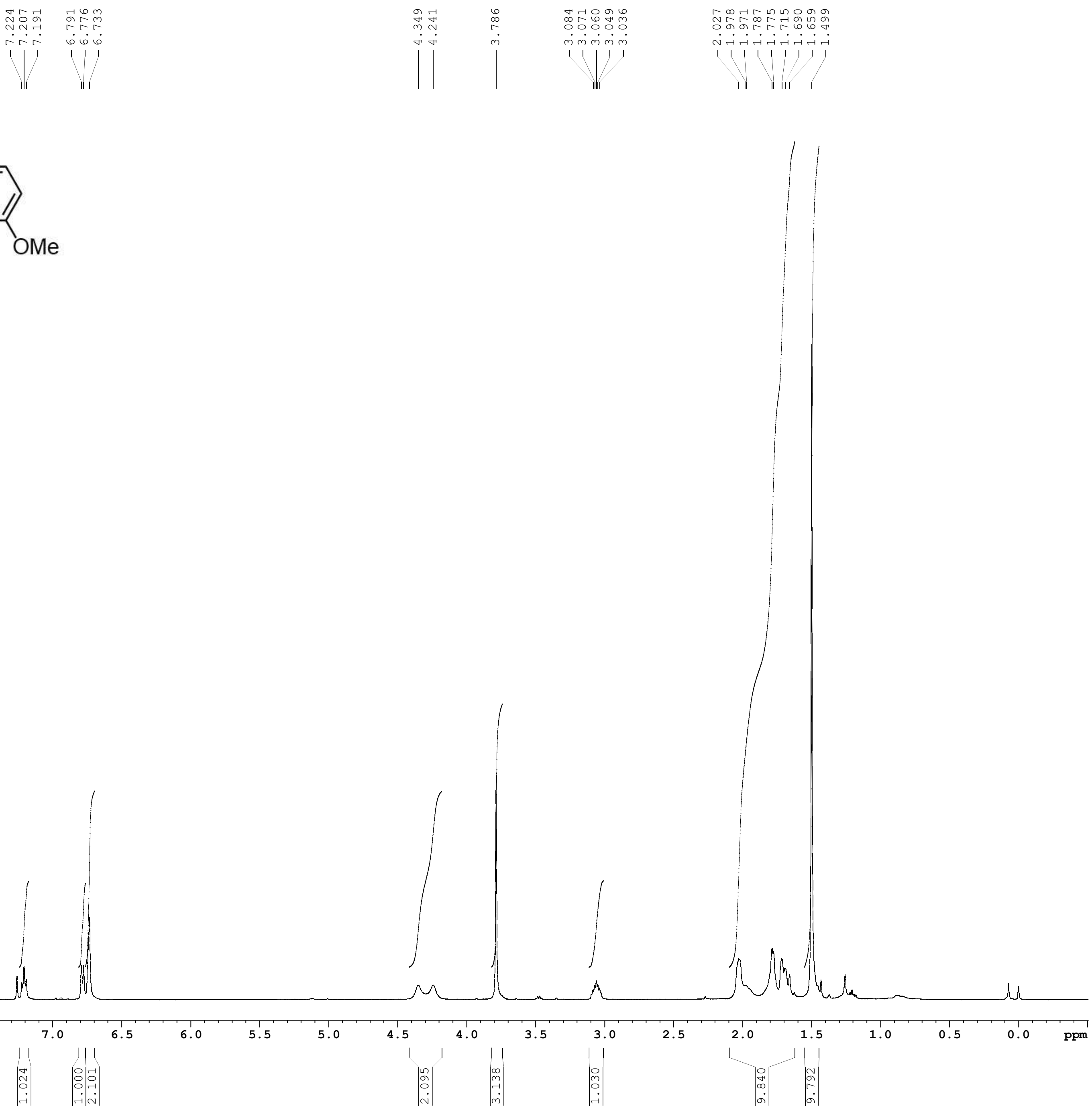


```
Current Data Parameters
NAME     cdw3-161-p-good
EXPNO                  1
PROCNO                 1

F2 - Acquisition Parameters
Date_           20240801
Time                7.56
INSTRUM            gn500
PROBHD   5 mm broadband
PULPROG             zg30
TD                 81728
SOLVENT            CDCl3
NS                     8
DS                     2
SWH             8012.820 Hz
FIDRES          0.098043 Hz
AQ             5.0998273 sec
RG                 143.7
DW                62.400 usec
DE                  6.00 usec
TE                 298.0 K
D1            0.10000000 sec
MCREST                 0 sec
MCWRK         0.01500000 sec

======== CHANNEL f1 ========
NUC1                  1H
P1                 12.00 usec
PL1                -6.00 dB
SFO1         498.4534891 MHz

F2 - Processing parameters
SI                 65536
SF           498.4500304 MHz
WDW                   no
SSB                    0
LB                     0 Hz
GB                     0
PC                  1.00
```

13C spectrum with 1H decoupling

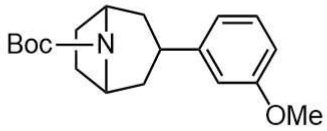

12

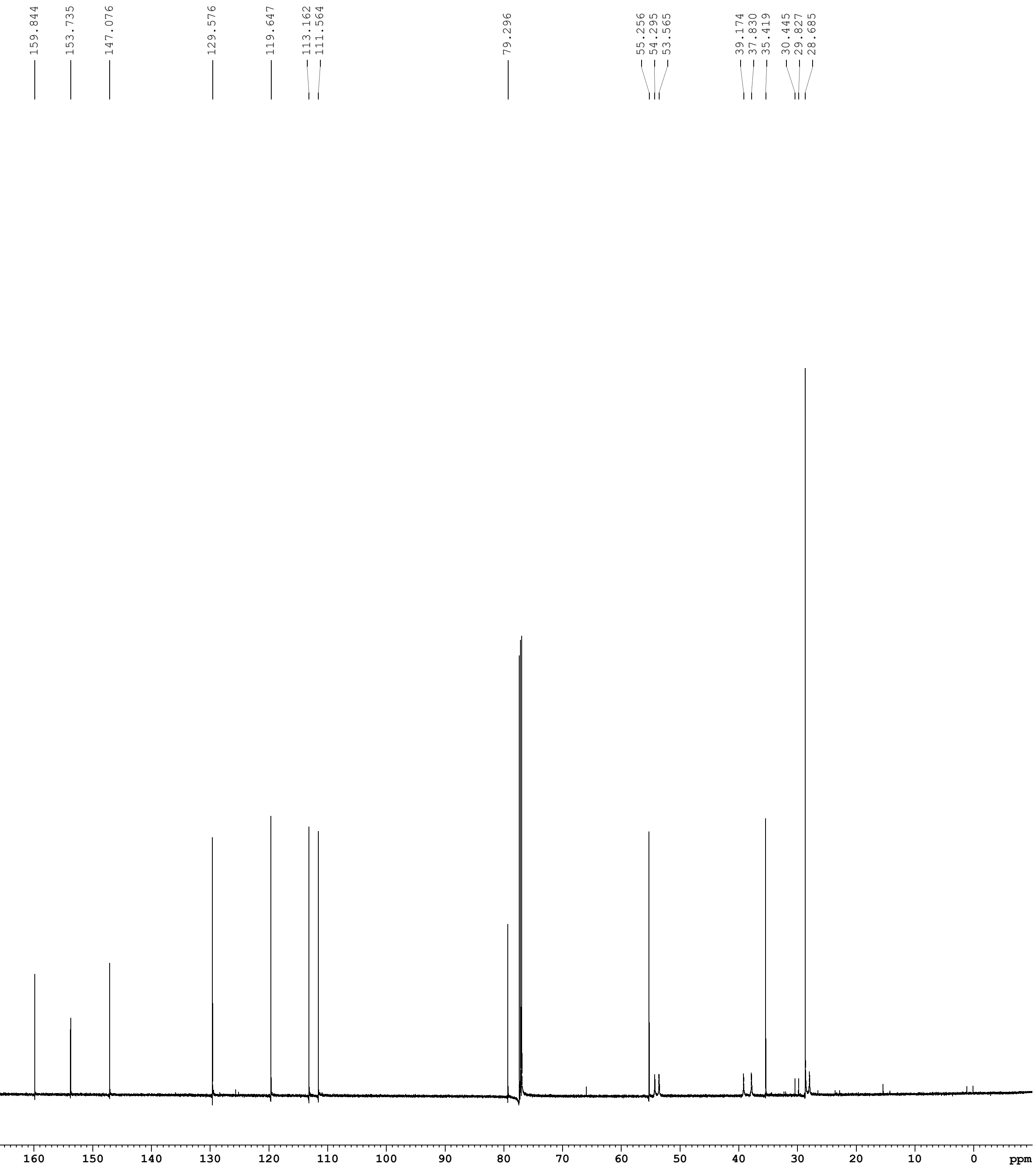


```
Current Data Parameters
NAME       cdw3-161-c13
EXPNO                 1
PROCNO                1

F2 - Acquisition Parameters
Date_          20240803
Time              10.50
INSTRUM           av600
PROBHD   5 mm CPBBO BB-
PULPROG          zgdc30
TD                65536
SOLVENT           CDCl3
NS                  600
DS                    4
SWH           36231.883 Hz
FIDRES         0.552855 Hz
AQ            0.9043968 sec
RG                 2050
DW               13.800 usec
DE                19.65 usec
TE                297.9 K
D1           0.40000001 sec
D11          0.03000000 sec
TD0                   1

======== CHANNEL f1 ========
SFO1        150.9194080 MHz
NUC1                13C
P1                10.00 usec
PLW1        68.40000153 W

======== CHANNEL f2 ========
SFO2        600.1330010 MHz
NUC2                 1H
CPDPRG[2        waltz16
PCPD2             80.00 usec
PLW2        30.00000000 W
PLW12        0.39811000 W

F2 - Processing parameters
SI                65536
SF          150.9027967 MHz
WDW                  no
SSB                   0
LB                    0 Hz
GB                    0
PC                 1.00
```

1H spectrum

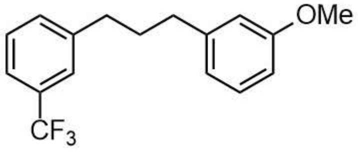

13

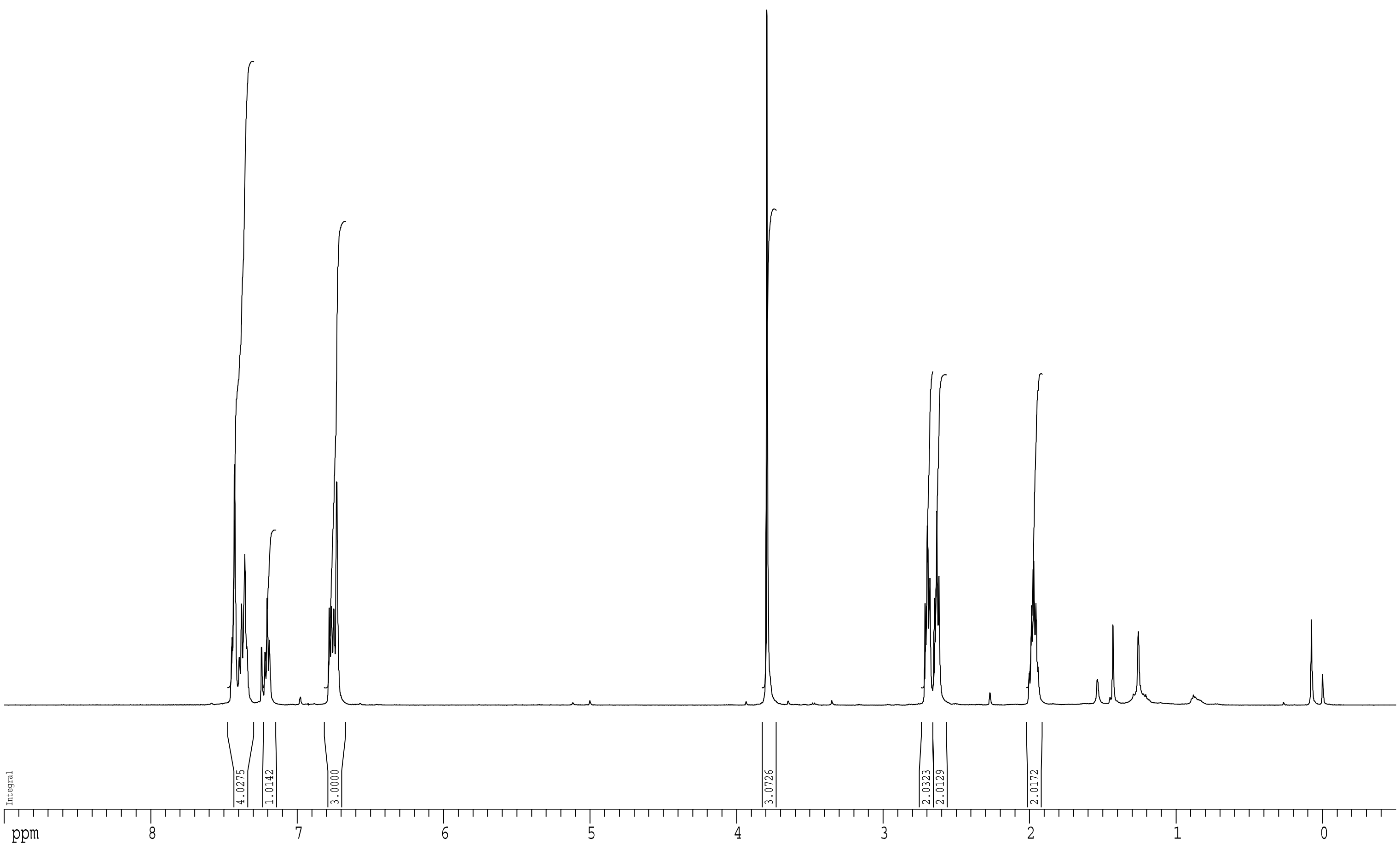

Current Data Parameters
USER nhirbawi
NAME NH-4-189-column
EXPNO 1
PROCNO 1

F2 - Acquisition Parameters
Date_ 20240419
Time 14.58
INSTRUM gn500
PROBHD 5 mm broadband
PULPROG zg30
TD 48074
SOLVENT CDCl3T
NS 8
DS 2
SWH 8012.820 Hz
FIDRES 0.166677 Hz
AQ 2.9998677 sec
RG 161.3
DW 62.400 usec
DE 6.00 usec
TE 298.0 K
D1 0.10000000 sec
MCREST 0.00000000 sec
MCWRK 0.01500000 sec

======== CHANNEL f1 ========
NUC1 1H
P1 12.00 usec
PL1 -6.00 dB
SFO1 498.4534891 MHz

F2 - Processing parameters
SI 65536
SF 498.4500390 MHz
WDW EM
SSB 0
LB 0.30 Hz
GB 0
PC 1.00

1D NMR plot parameters
CX 20.00 cm
CY 10.00 cm
F1P 9.000 ppm
F1 4486.05 Hz
F2P -0.500 ppm
F2 -249.23 Hz
PPMCM 0.47500 ppm/cm
HZCM 236.76576 Hz/cm

1H spectrum

14

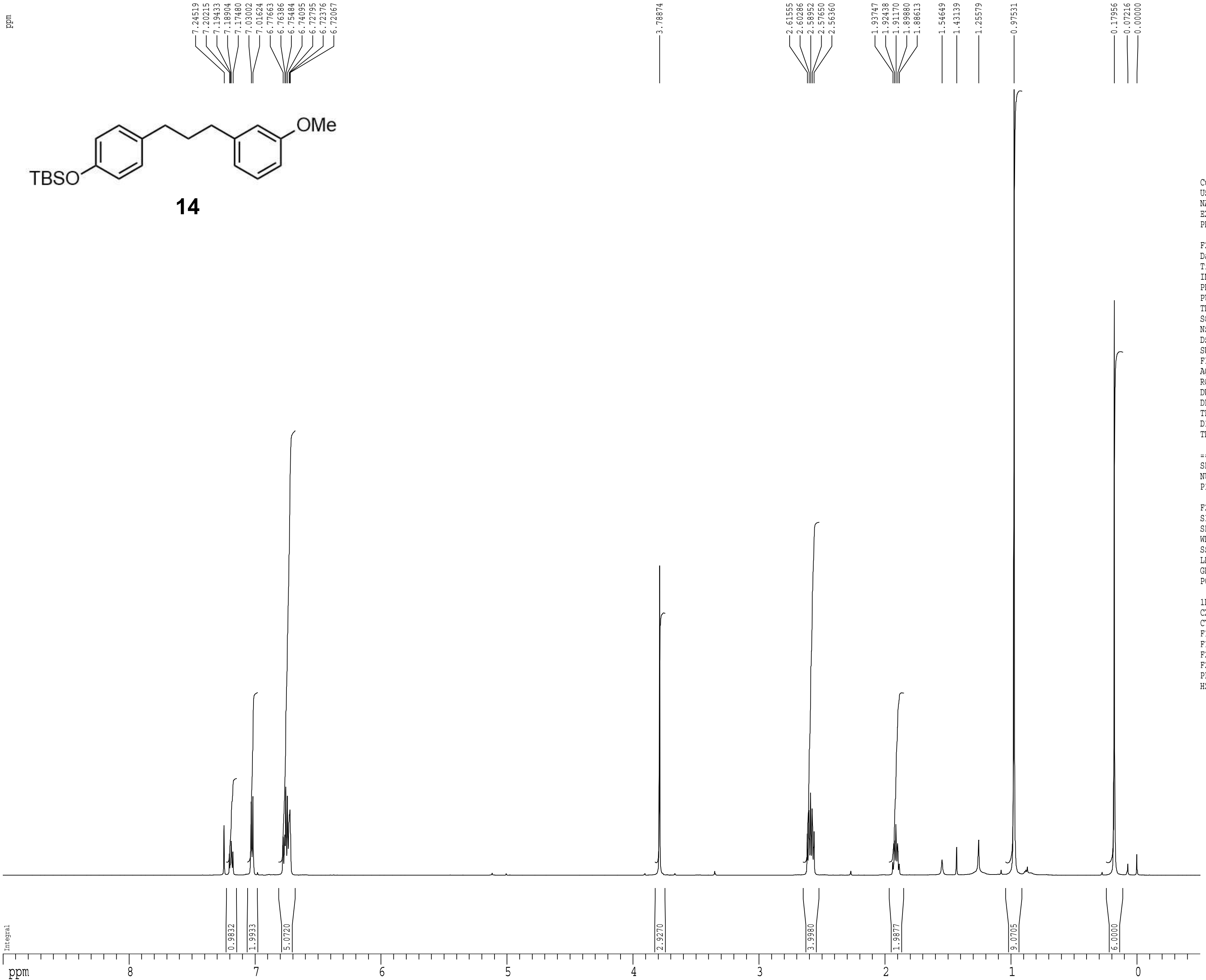

Current Data Parameters
USER nhirbawi
NAME NH-4-190
EXPNO 2
PROCNO 1

F2 - Acquisition Parameters
Date_ 20240528
Time 18.15
INSTRUM av600
PROBHD 5 mm CPBBO BB-
PULPROG zg30
TD 98074
SOLVENT CDCl3T
NS 8
DS 2
SWH 9615.385 Hz
FIDRES 0.098042 Hz
AQ 5.0998979 sec
RG 28.5
DW 52.000 usec
DE 14.12 usec
TE 297.9 K
D1 0.10000000 sec
TD0 1

======== CHANNEL f1 ========
SFO1 600.1342009 MHz
NUC1 1H
P1 10.00 usec

F2 - Processing parameters
SI 65536
SF 600.1300435 MHz
WDW EM
SSB 0
LB 0.30 Hz
GB 0
PC 1.00

1D NMR plot parameters
CX 22.80 cm
CY 15.00 cm
F1P 9.000 ppm
F1 5401.17 Hz
F2P -0.500 ppm
F2 -300.06 Hz
PPMCM 0.41667 ppm/cm
HZCM 250.05420 Hz/cm

13C spectrum with 1H decoupling

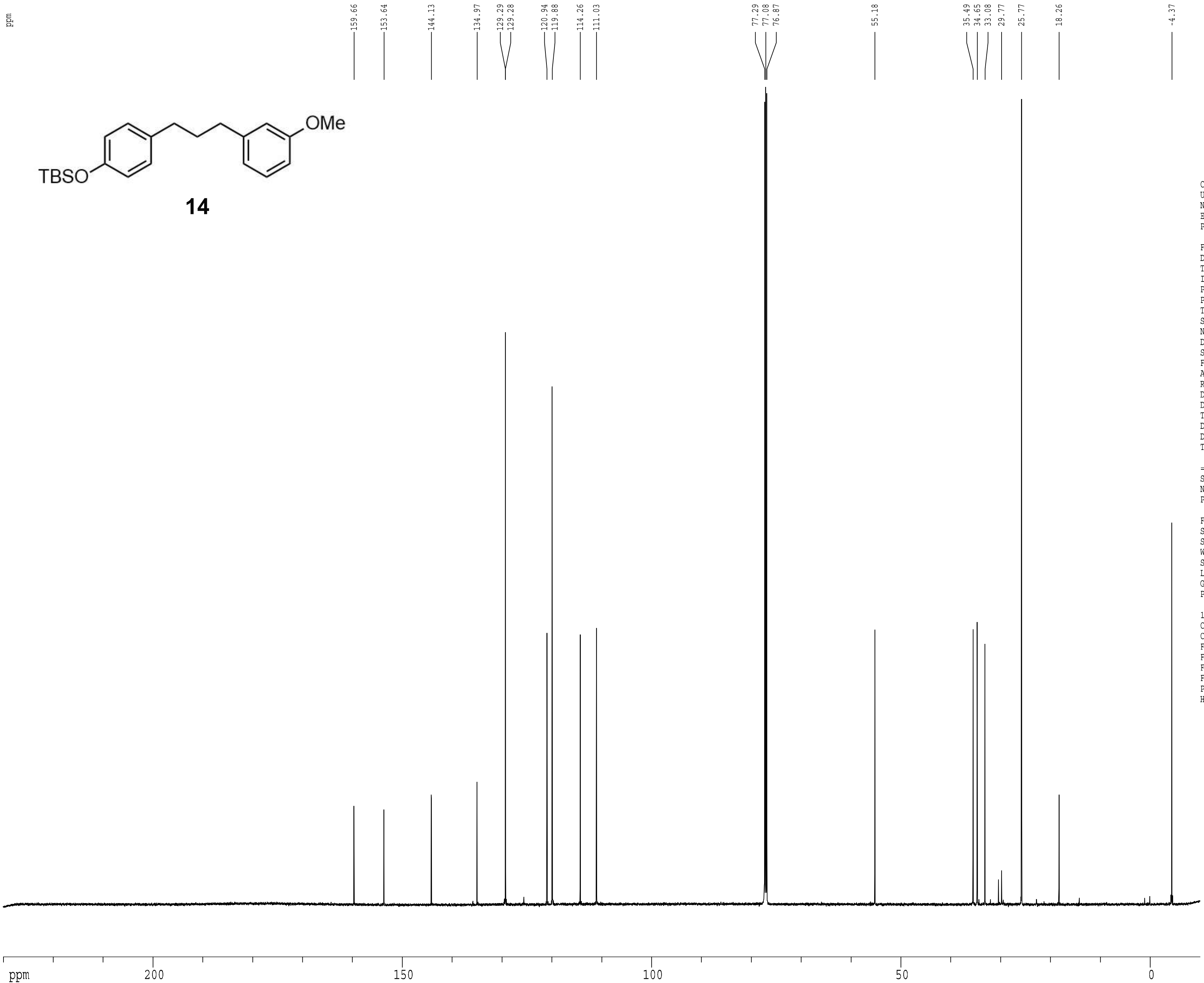


```
Current Data Parameters
USER            nhirbawi
NAME            NH-4-190
EXPNO                  1
PROCNO                 1

F2 - Acquisition Parameters
Date_           20240528
Time               18.10
INSTRUM            av600
PROBHD   5 mm CPBBO BB-
PULPROG           zgdc30
TD                 65536
SOLVENT           CDCl3T
NS                   377
DS                     4
SWH            36231.883 Hz
FIDRES          0.552855 Hz
AQ             0.9044468 sec
RG                  2050
DW                13.800 usec
DE                 19.65 usec
TE                 298.0 K
D1            0.40000001 sec
D11           0.03000000 sec
TD0                    1

======== CHANNEL f1 ========
SFO1         150.9194080 MHz
NUC1                 13C
P1                 10.00 usec

F2 - Processing parameters
SI                 65536
SF           150.9028085 MHz
WDW                   EM
SSB                    0
LB                  1.00 Hz
GB                     0
PC                  1.00

1D NMR plot parameters
CX                 22.80 cm
CY                 15.65 cm
F1P              230.000 ppm
F1              34707.64 Hz
F2P              -10.000 ppm
F2              -1509.03 Hz
PPMCM           10.52632 ppm/cm
HZCM          1588.45056 Hz/cm
```

1H spectrum

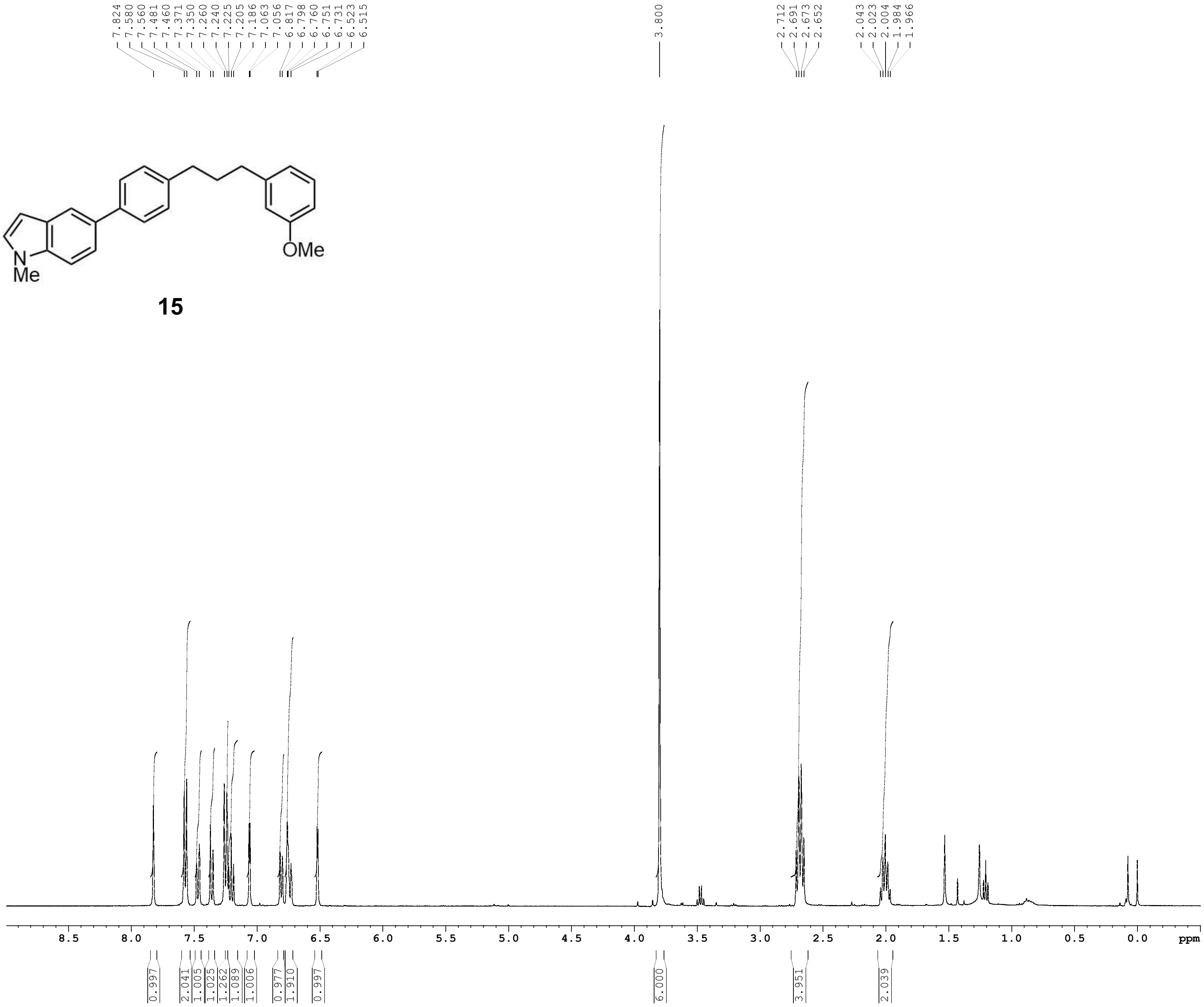

15

```
Current Data Parameters
NAME          cdw-2-273-p
EXPNO                   1
PROCNO                  1

F2 - Acquisition Parameters
Date_            20240219
Time                17.07 h
INSTRUM           NEO 400
PROBHD   Z163739_0122 (
PULPROG              zg30
TD                  65536
SOLVENT             CDCl3
NS                      8
DS                      2
SWH              6250.000 Hz
FIDRES           0.190735 Hz
AQ              5.2428799 sec
RG                    101
DW                 80.000 usec
DE                   8.64 usec
TE                  298.3 K
D1             0.10000000 sec
TD0                     1
SFO1          400.1328009 MHz
NUC1                   1H
P0                   2.67 usec
P1                   8.00 usec
PLW1          23.01399994 W

F2 - Processing parameters
SI                  65536
SF            400.1300316 MHz
WDW                    no
SSB                     0
LB                      0 Hz
GB                      0
PC                   1.00
```

13C spectrum with 1H decoupling

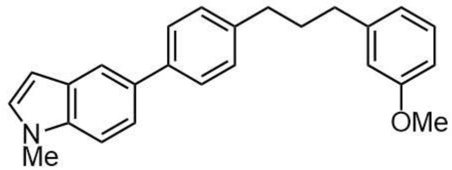

15

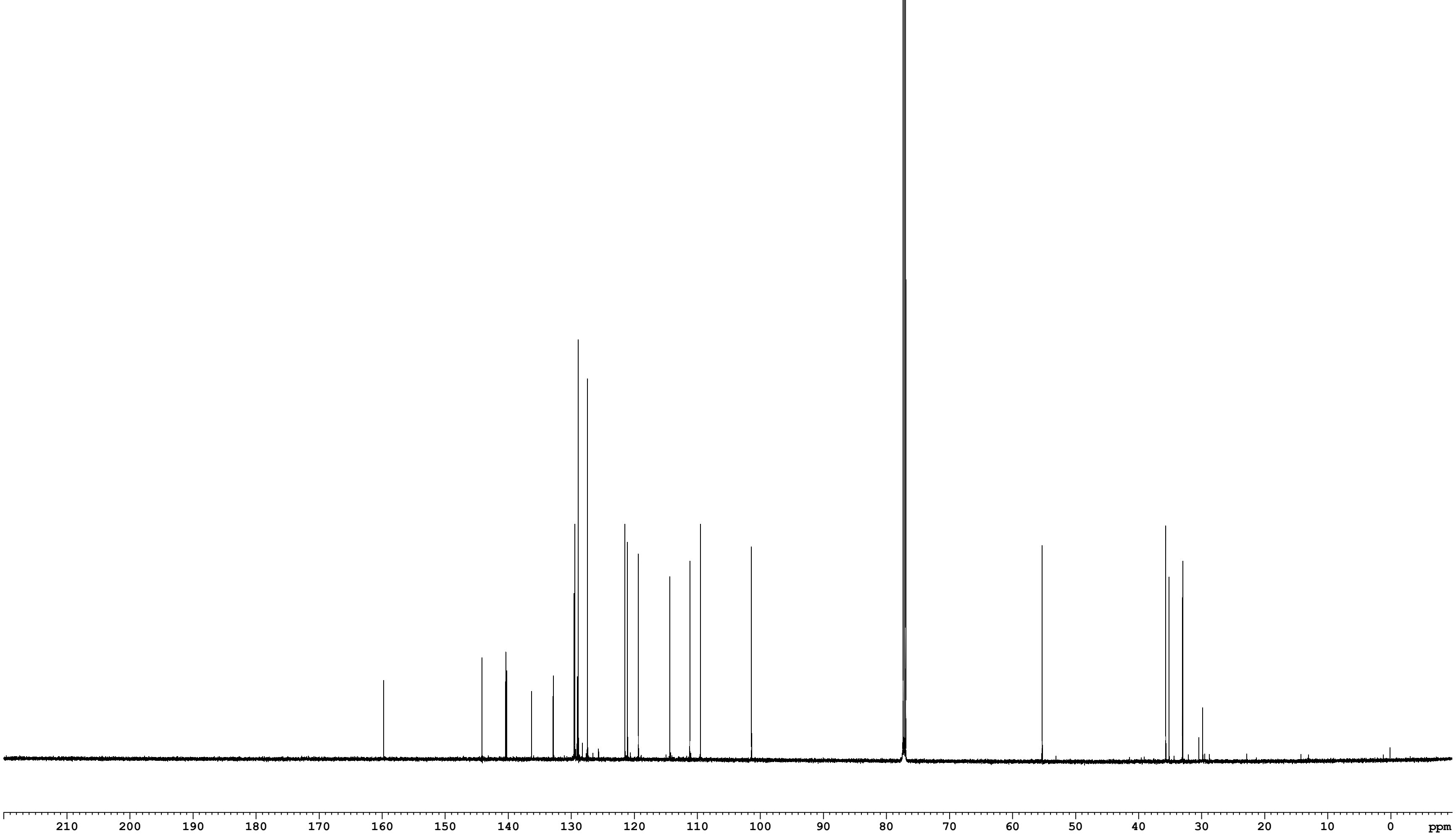


```
Current Data Parameters
NAME        cdw2-273-c13
EXPNO                  1
PROCNO                 1

F2 - Acquisition Parameters
Date_           20240801
Time                8.09
INSTRUM            av600
PROBHD   5 mm CPBBO BB-
PULPROG          zgdc30
TD                65536
SOLVENT           CDCl3
NS                  800
DS                    4
SWH           36231.883 Hz
FIDRES         0.552855 Hz
AQ            0.9043968 sec
RG                 2050
DW               13.800 usec
DE                19.65 usec
TE                298.0 K
D1           0.40000001 sec
D11          0.03000000 sec
TD0                   1

======== CHANNEL f1 ========
SFO1        150.9194080 MHz
NUC1                13C
P1                10.00 usec
PLW1        68.40000153 W

======== CHANNEL f2 ========
SFO2        600.1330010 MHz
NUC2                 1H
CPDPRG[2          waltz16
PCPD2             80.00 usec
PLW2        30.00000000 W
PLW12        0.39811000 W

F2 - Processing parameters
SI                65536
SF          150.9027971 MHz
WDW                  no
SSB                   0
LB                    0 Hz
GB                    0
PC                 1.00
```

1H spectrum

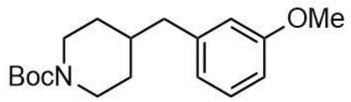

16

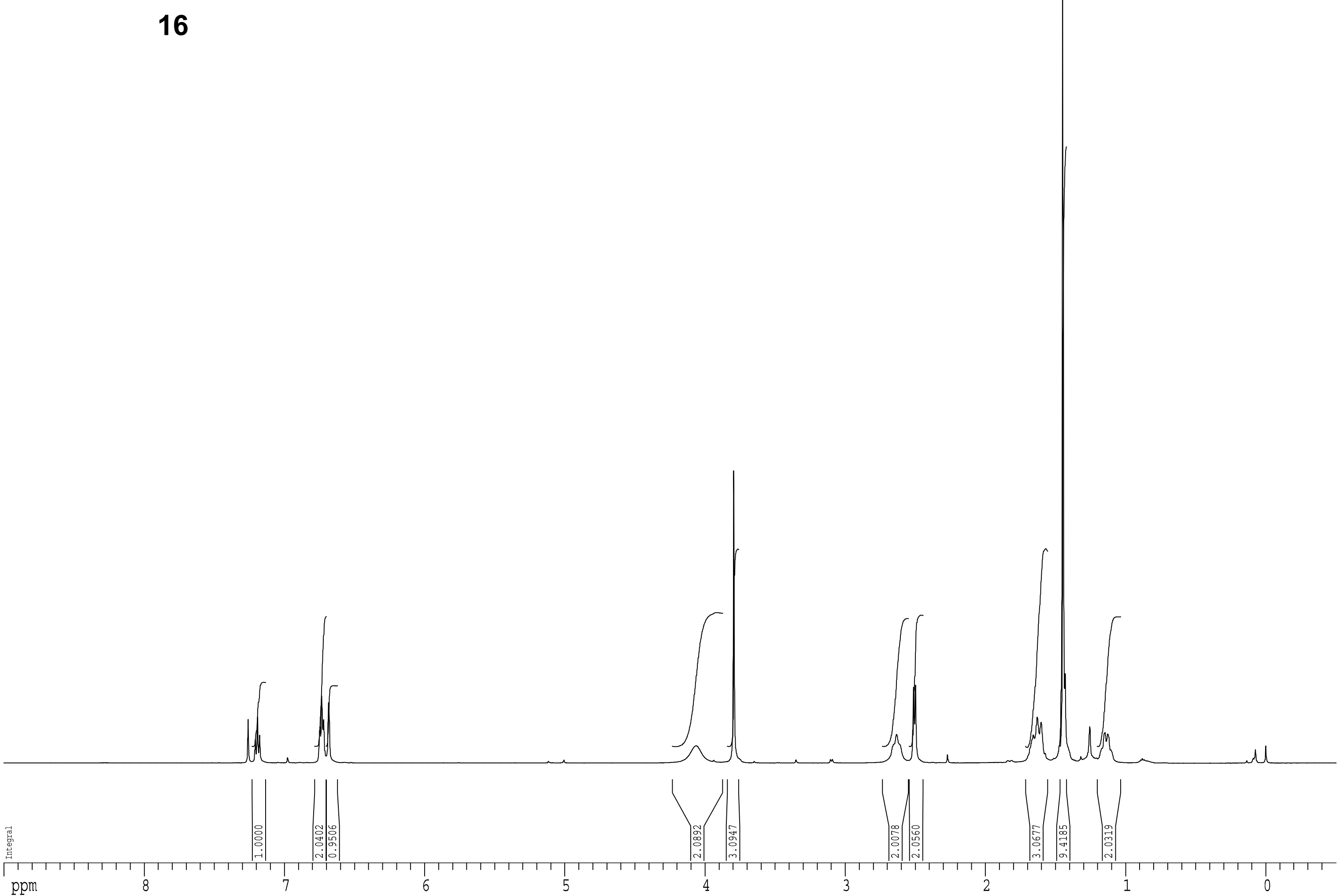

Current Data Parameters
USER nhirbawi
NAME NH-4-194-frac73-83
EXPNO 1
PROCNO 1

F2 - Acquisition Parameters
Date_ 20240603
Time 16.11
INSTRUM gn500
PROBHD 5 mm broadband
PULPROG zg30
TD 81728
SOLVENT CDCl3T
NS 8
DS 2
SWH 8012.820 Hz
FIDRES 0.098043 Hz
AQ 5.0998774 sec
RG 143.7
DW 62.400 usec
DE 6.00 usec
TE 298.1 K
D1 0.10000000 sec
MCREST 0.00000000 sec
MCWRK 0.01500000 sec

======== CHANNEL f1 ========
NUC1 1H
P1 12.00 usec
PL1 -6.00 dB
SFO1 498.4534891 MHz

F2 - Processing parameters
SI 65536
SF 498.4500304 MHz
WDW EM
SSB 0
LB 0.30 Hz
GB 0
PC 1.00

1D NMR plot parameters
CX 20.00 cm
CY 12.50 cm
F1P 9.000 ppm
F1 4486.05 Hz
F2P -0.500 ppm
F2 -249.23 Hz
PPMCM 0.47500 ppm/cm
HZCM 236.76376 Hz/cm

1H spectrum

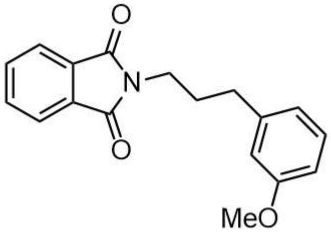

17

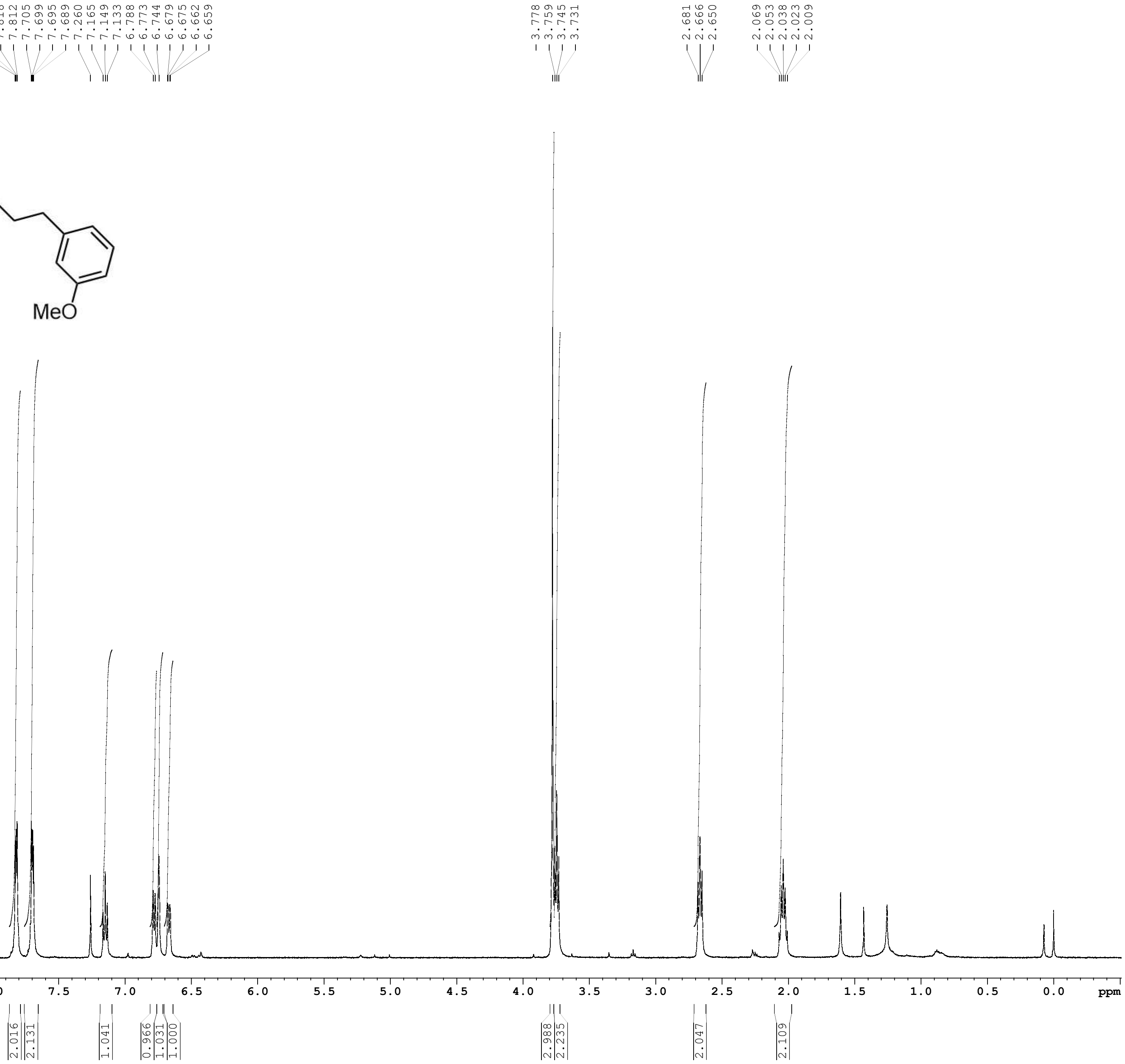


```
Current Data Parameters
NAME          cdw-2-292-p
EXPNO                   1
PROCNO                  1

F2 - Acquisition Parameters
Date_            20240319
Time                10.10
INSTRUM             gn500
PROBHD   5 mm broadband
PULPROG              zg30
TD                  81728
SOLVENT             CDCl3
NS                      8
DS                      2
SWH              8012.820 Hz
FIDRES           0.098043 Hz
AQ              5.0998273 sec
RG                  645.1
DW                 62.400 usec
DE                   6.00 usec
TE                  298.0 K
D1             0.10000000 sec
MCREST                  0 sec
MCWRK          0.01500000 sec

======== CHANNEL f1 ========
NUC1                   1H
P1                  12.00 usec
PL1                 -6.00 dB
SFO1          498.4534891 MHz

F2 - Processing parameters
SI                  65536
SF            498.4500303 MHz
WDW                    no
SSB                     0
LB                      0 Hz
GB                      0
PC                   1.00
```

13C spectrum with 1H decoupling

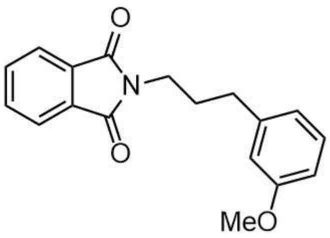

17

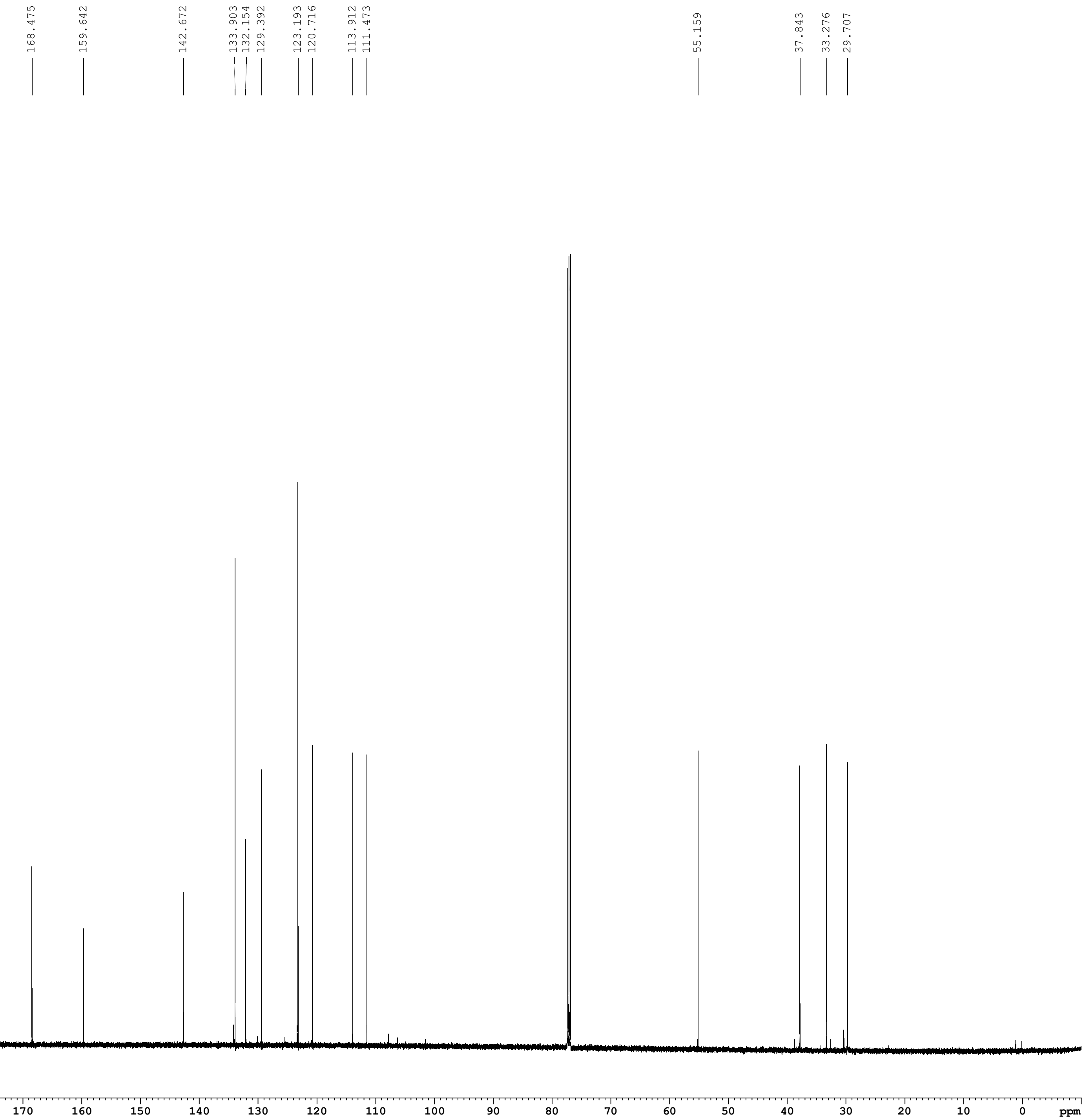


```
Current Data Parameters
NAME     cdw-2-292-c13
EXPNO               1
PROCNO              1

F2 - Acquisition Parameters
Date_        20240321
Time             9.37
INSTRUM         av600
PROBHD   5 mm CPBBO BB-
PULPROG        zgdc30
TD              65536
SOLVENT         CDCl3
NS                250
DS                  4
SWH         36231.883 Hz
FIDRES       0.552855 Hz
AQ          0.9043968 sec
RG               2050
DW             13.800 usec
DE              19.65 usec
TE              298.1 K
D1         0.40000001 sec
D11        0.03000000 sec
TD0                 1

======== CHANNEL f1 ========
SFO1      150.9194080 MHz
NUC1              13C
P1              10.00 usec
PLW1      68.40000153 W

======== CHANNEL f2 ========
SFO2      600.1330010 MHz
NUC2               1H
CPDPRG[2      waltz16
PCPD2           80.00 usec
PLW2      30.00000000 W
PLW12      0.39811000 W

F2 - Processing parameters
SI              65536
SF        150.9028085 MHz
WDW                no
SSB                 0
LB                  0 Hz
GB                  0
PC               1.00
```

1H spectrum

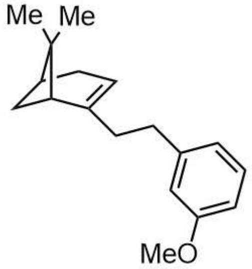

18

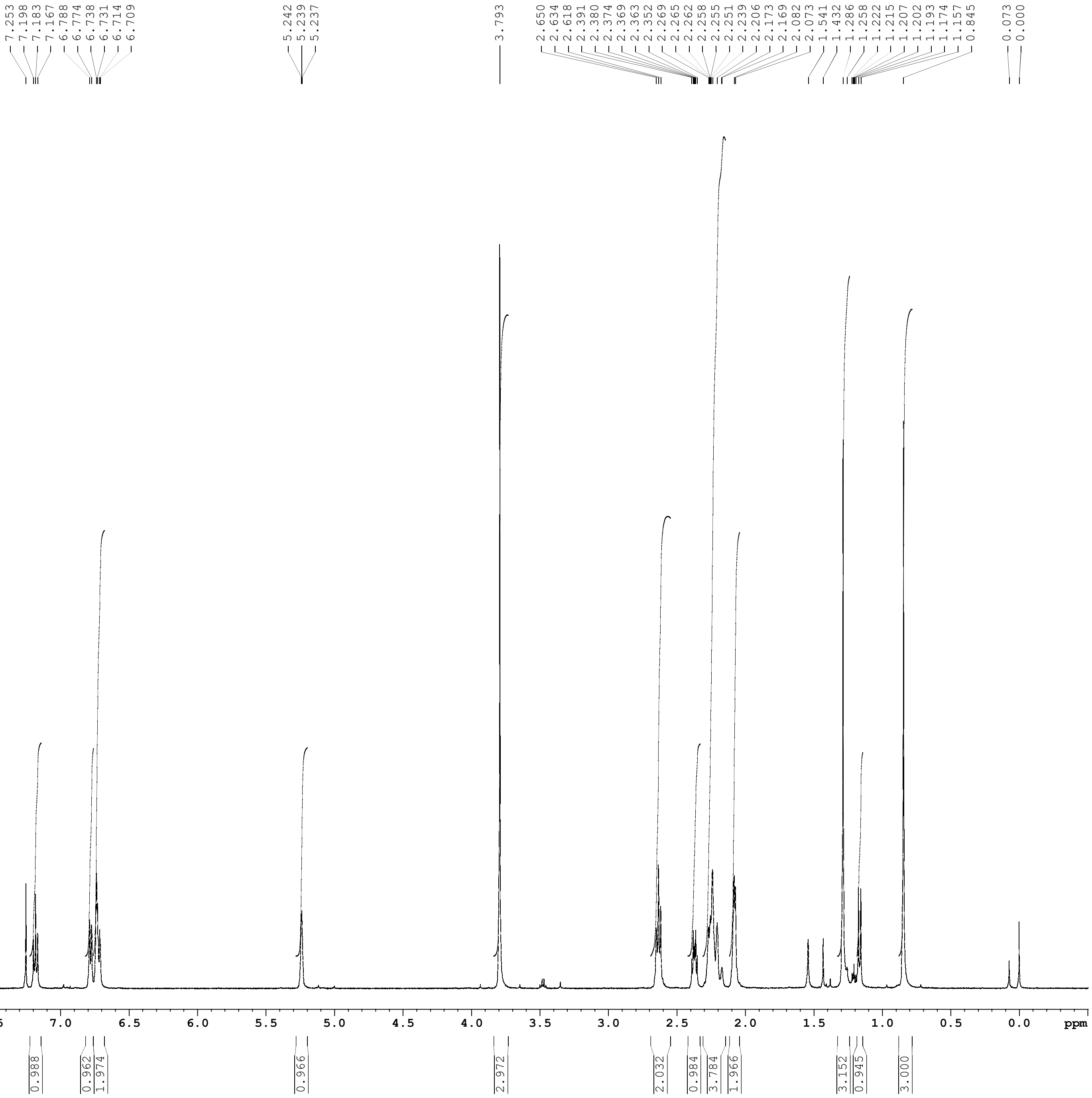


```
Current Data Parameters
NAME        cdw-2-289-ge
EXPNO                  1
PROCNO                 1

F2 - Acquisition Parameters
Date_           20240313
Time                9.02
INSTRUM            gn500
PROBHD   5 mm broadband
PULPROG             zg30
TD                 81728
SOLVENT            CDCl3
NS                     8
DS                     2
SWH             8012.820 Hz
FIDRES          0.098043 Hz
AQ             5.0998273 sec
RG                 574.7
DW                62.400 usec
DE                  6.00 usec
TE                 298.0 K
D1            0.10000000 sec
MCREST                 0 sec
MCWRK         0.01500000 sec

======== CHANNEL f1 ========
NUC1                  1H
P1                 12.00 usec
PL1                -6.00 dB
SFO1         498.4534891 MHz

F2 - Processing parameters
SI                 65536
SF           498.4500339 MHz
WDW                   no
SSB                    0
LB                     0 Hz
GB                     0
PC                  1.00
```

13C spectrum with 1H decoupling

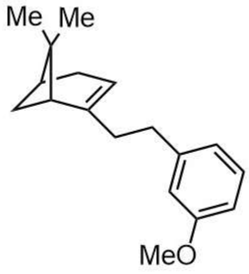

18

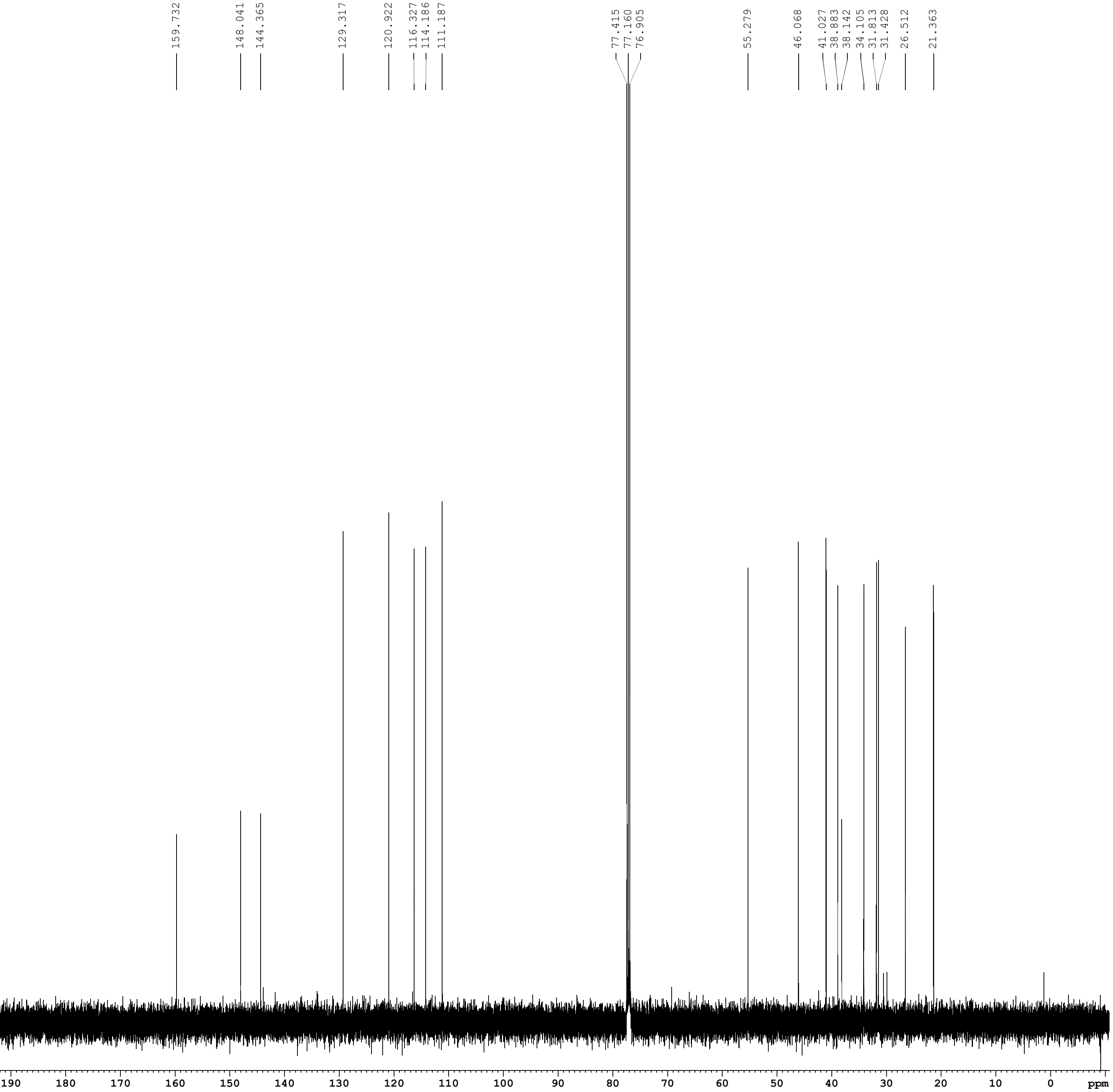


```
Current Data Parameters
NAME      cdw-2-289-c13
EXPNO                 1
PROCNO                1

F2 - Acquisition Parameters
Date_          20240309
Time               9.52
INSTRUM           gn500
PROBHD   5 mm broadband
PULPROG          zgdc30
TD                65536
SOLVENT           CDCl3
NS                  890
DS                    4
SWH           30303.031 Hz
FIDRES         0.462388 Hz
AQ            1.0813440 sec
RG              20642.5
DW               16.500 usec
DE                 6.00 usec
TE                298.0 K
D1           0.25000000 sec
d11          0.03000000 sec
MCREST                0 sec
MCWRK        0.01500000 sec

======== CHANNEL f1 ========
NUC1                13C
P1                14.20 usec
PL1               -6.00 dB
SFO1        125.3491398 MHz

======== CHANNEL f2 ========
CPDPRG[2        waltz16
NUC2                 1H
PCPD2            100.00 usec
PL2               -6.00 dB
PL12              12.30 dB
SFO2        498.4524922 MHz

F2 - Processing parameters
SI                65536
SF          125.3353398 MHz
WDW                  no
SSB                   0
LB                    0 Hz
GB                    0
PC                 2.00
```

1H spectrum

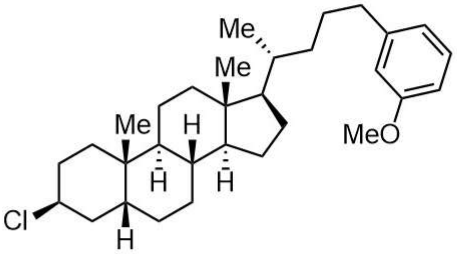

19

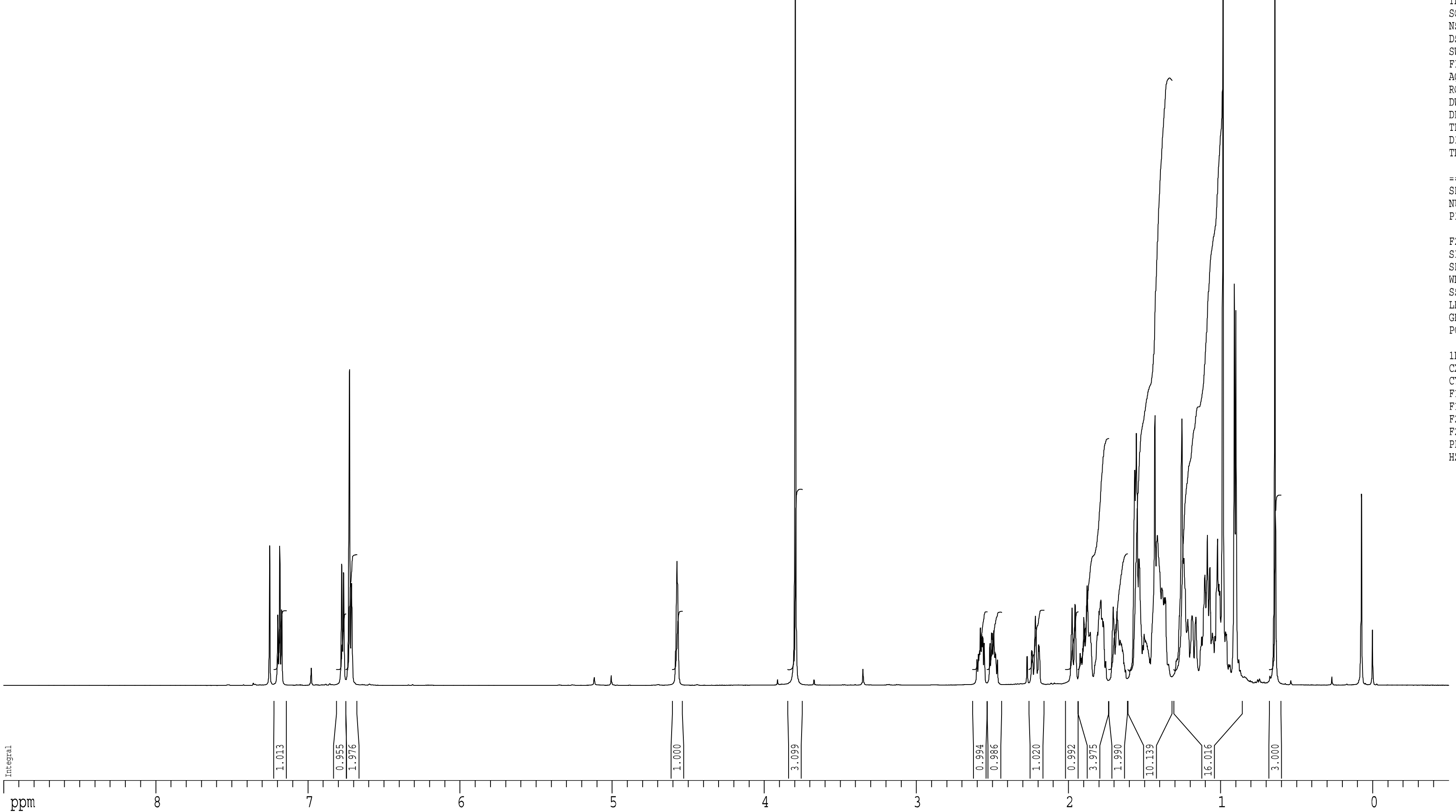

Current Data Parameters
USER nhirbawi
NAME NH-4-195
EXPNO 2
PROCNO 1

F2 - Acquisition Parameters
Date_ 20240528
Time 18.48
INSTRUM av600
PROBHD 5 mm CPBBO BB-
PULPROG zg30
TD 98074
SOLVENT CDCl3T
NS 8
DS 2
SWH 9615.385 Hz
FIDRES 0.098042 Hz
AQ 5.0998979 sec
RG 22.6
DW 52.000 usec
DE 14.12 usec
TE 298.0 K
D1 0.10000000 sec
TD0 1

======== CHANNEL f1 ========
SFO1 600.1342009 MHz
NUC1 1H
P1 10.00 usec

F2 - Processing parameters
SI 65536
SF 600.1300405 MHz
WDW EM
SSB 0
LB 0.30 Hz
GB 0
PC 1.00

1D NMR plot parameters
CX 22.80 cm
CY 15.00 cm
F1P 9.000 ppm
F1 5401.17 Hz
F2P -0.500 ppm
F2 -300.06 Hz
PPMCM 0.41667 ppm/cm
HZCM 250.05420 Hz/cm

13C spectrum with 1H decoupling

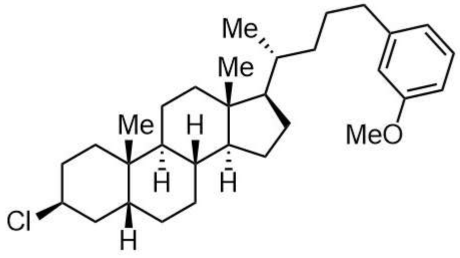

19

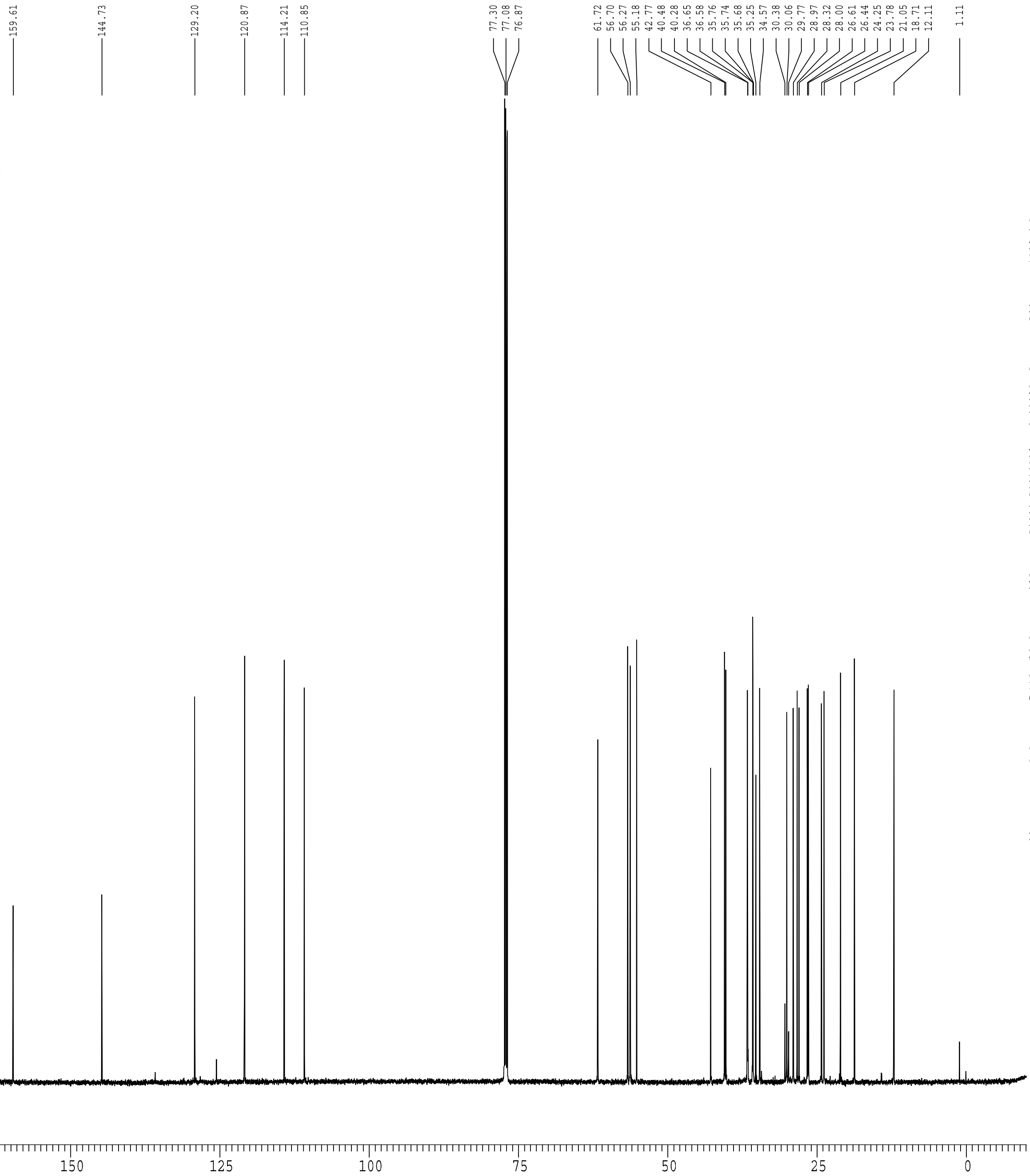

Current Data Parameters
USER nhirbawi
NAME NH-4-195
EXPNO 1
PROCNO 1

F2 - Acquisition Parameters
Date_ 20240528
Time 18.44
INSTRUM av600
PROBHD 5 mm CPBBO BB-
PULPROG zgdc30
TD 65536
SOLVENT CDCl3T
NS 216
DS 4
SWH 36231.883 Hz
FIDRES 0.552855 Hz
AQ 0.9044468 sec
RG 2050
DW 13.800 usec
DE 19.65 usec
TE 297.9 K
D1 0.40000001 sec
D11 0.03000000 sec
TD0 1

======== CHANNEL f1 ========
SFO1 150.9194080 MHz
NUC1 13C
P1 10.00 usec

F2 - Processing parameters
SI 65536
SF 150.9028085 MHz
WDW EM
SSB 0
LB 1.00 Hz
GB 0
PC 1.00

1D NMR plot parameters
CX 22.80 cm
CY 15.65 cm
F1P 229.520 ppm
F1 34635.16 Hz
F2P -10.507 ppm
F2 -1585.47 Hz
PPMCM 10.52747 ppm/cm
HZCM 1588.62451 Hz/cm

1H spectrum

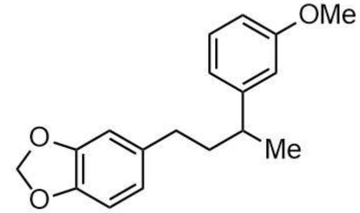

20

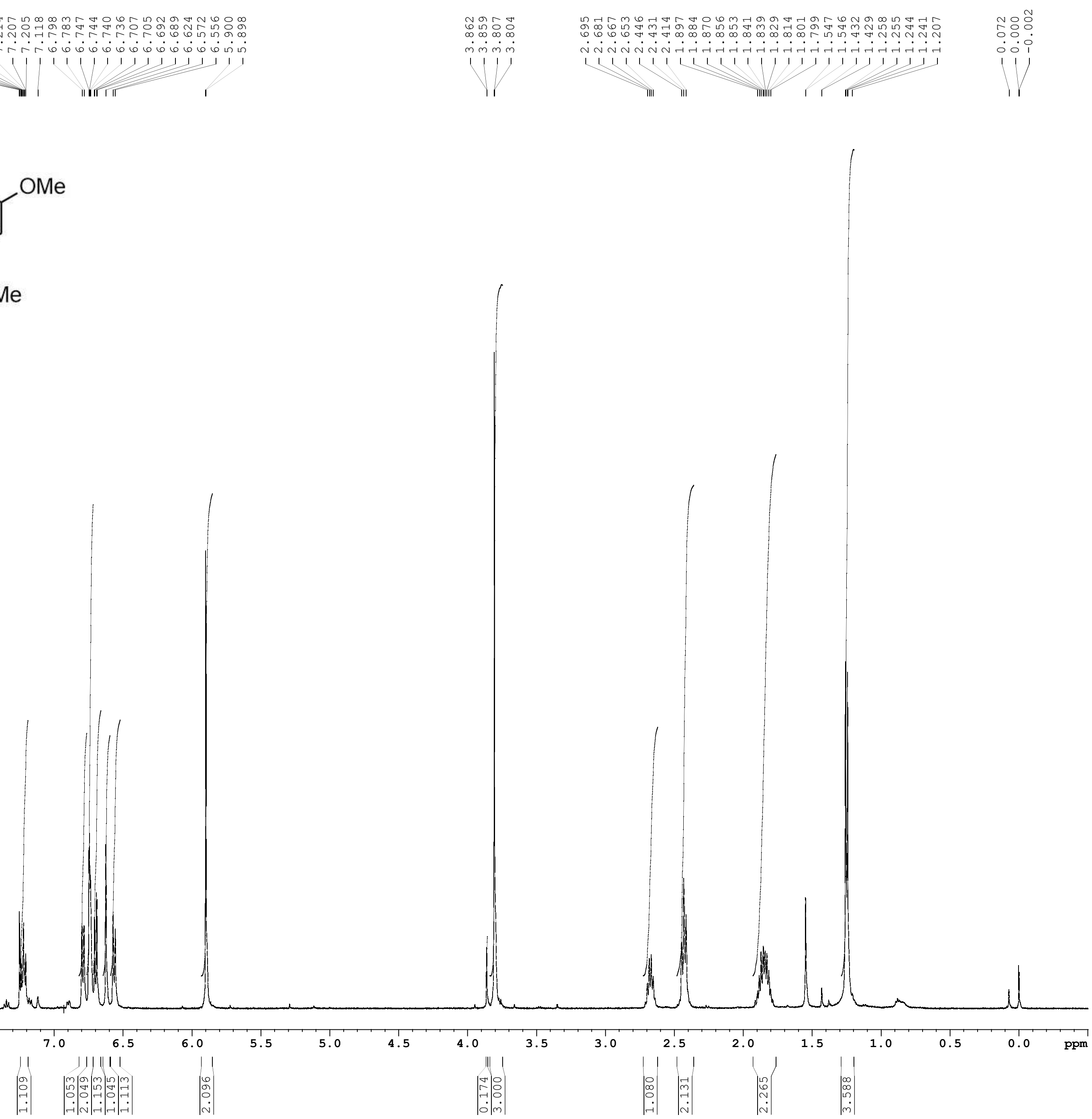

Current Data Parameters
NAME cdw-2-288-p2
EXPNO 1
PROCNO 1

F2 - Acquisition Parameters
Date_ 20240308
Time 8.10
INSTRUM gn500
PROBHD 5 mm broadband
PULPROG zg30
TD 81728
SOLVENT CDCl3
NS 8
DS 2
SWH 8012.820 Hz
FIDRES 0.098043 Hz
AQ 5.0998273 sec
RG 645.1
DW 62.400 usec
DE 6.00 usec
TE 298.0 K
D1 0.10000000 sec
MCREST 0 sec
MCWRK 0.01500000 sec

======== CHANNEL f1 ========
NUC1 1H
P1 12.00 usec
PL1 -6.00 dB
SFO1 498.4534891 MHz

F2 - Processing parameters
SI 65536
SF 498.4500349 MHz
WDW no
SSB 0
LB 0 Hz
GB 0
PC 1.00

1H spectrum

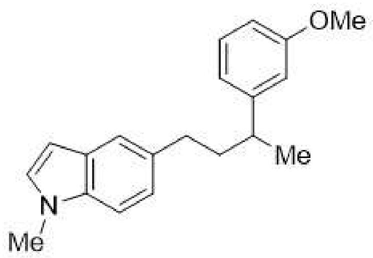

21

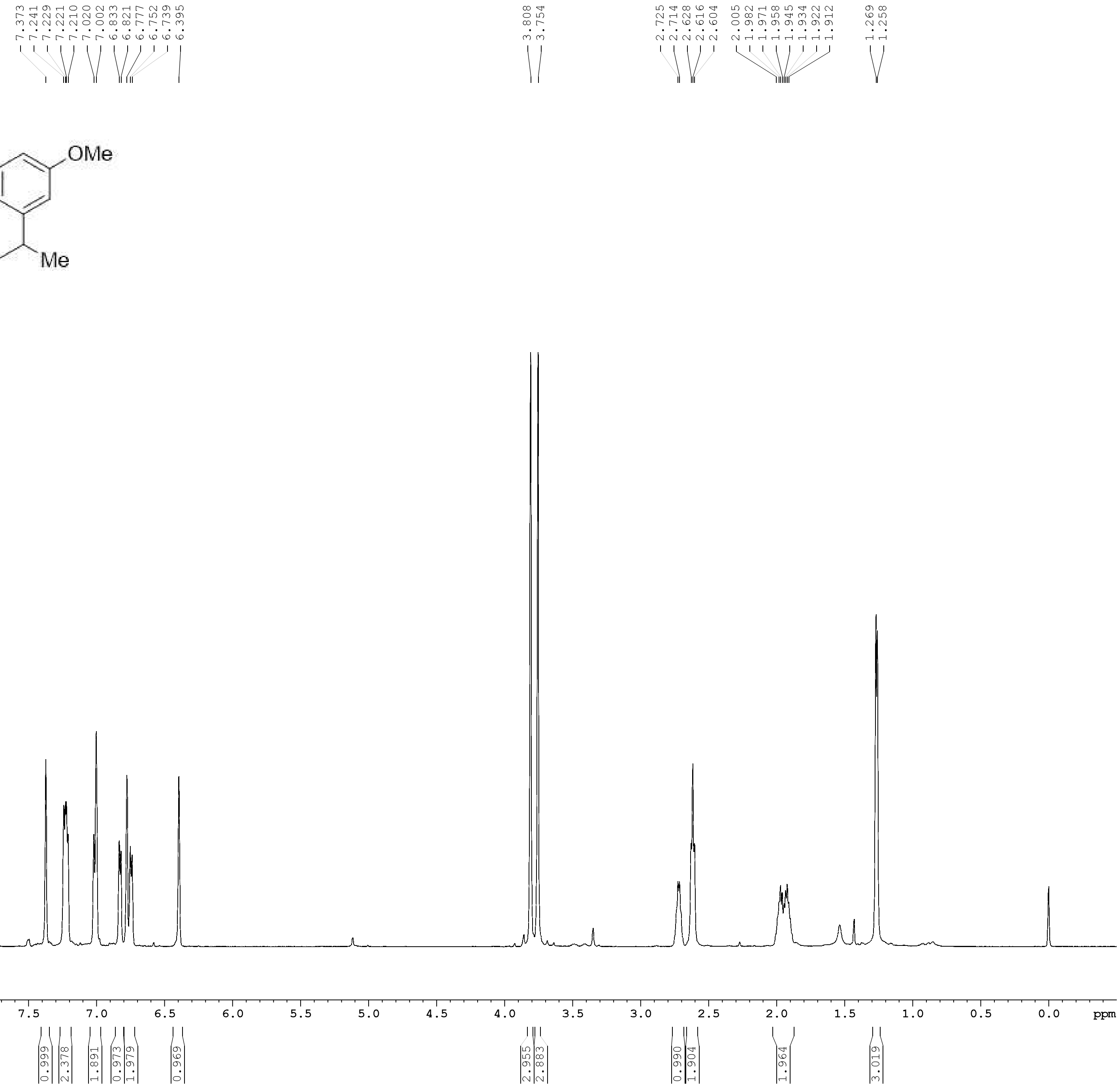


```
Current Data Parameters
NAME     LCB-1-32-colfr46-80-600
EXPNO                  1
PROCNO                 1

F2 - Acquisition Parameters
Date_           20240429
Time               14.47
INSTRUM            av600
PROBHD   5 mm CPBBO BB-
PULPROG             zg30
TD                 98074
SOLVENT           CDCl3T
NS                     8
DS                     2
SWH             9615.385 Hz
FIDRES          0.098042 Hz
AQ             5.0998478 sec
RG                  25.4
DW                52.000 usec
DE                 14.12 usec
TE                 298.0 K
D1            0.10000000 sec
TD0                    1

======== CHANNEL f1 ========
SFO1         600.1342009 MHz
NUC1                  1H
P1                 10.00 usec
PLW1         30.00000000 W

F2 - Processing parameters
SI                 65536
SF           600.1300471 MHz
WDW                   EM
SSB                    0
LB                  0.30 Hz
GB                     0
PC                  1.00
```

13C spectrum with 1H decoupling

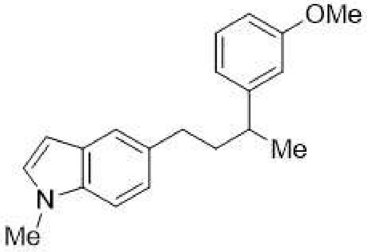

21

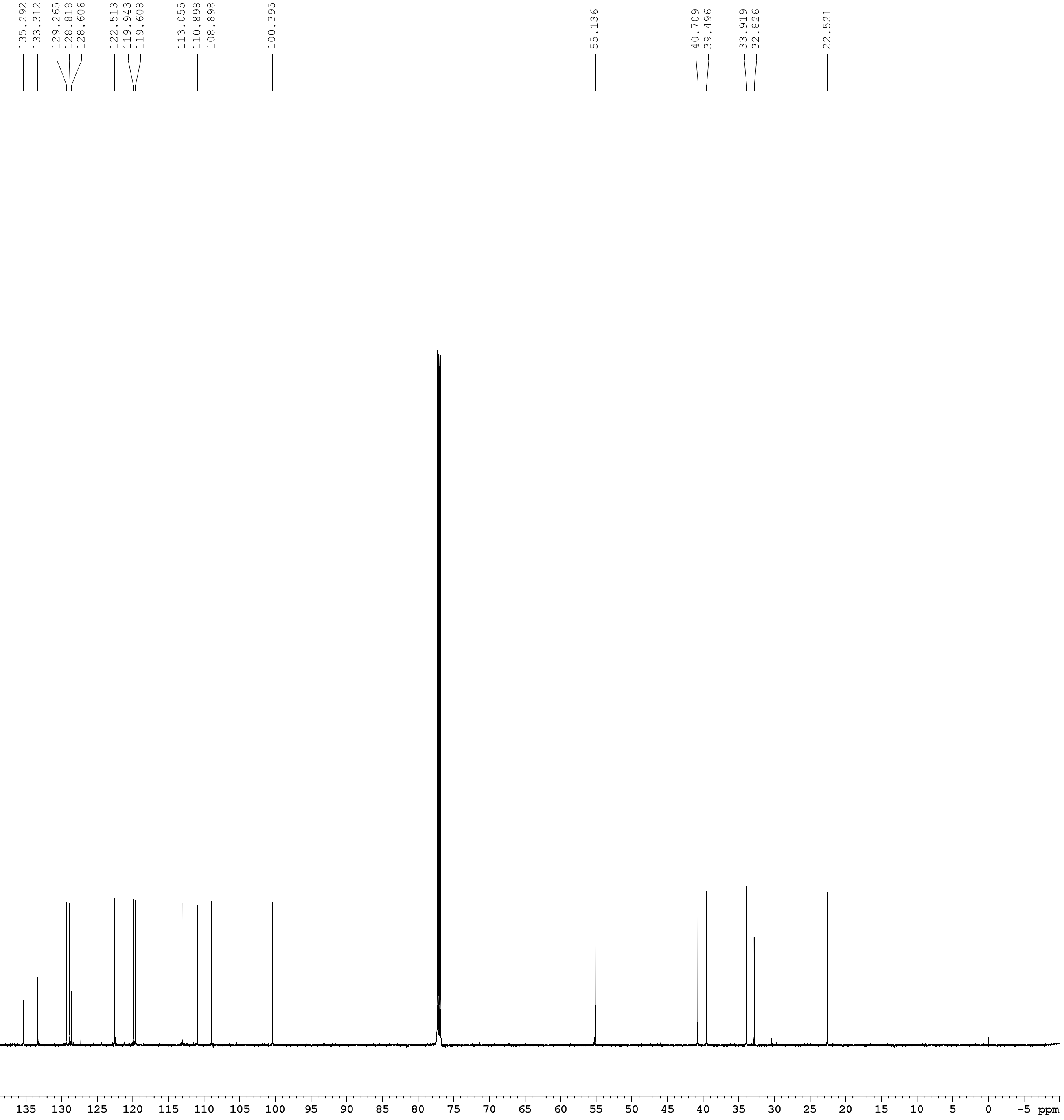

Current Data Parameters
NAME LCB-1-32-colfr46-80
EXPNO 1
PROCNO 1

F2 - Acquisition Parameters
Date_ 20240429
Time 14.41
INSTRUM av600
PROBHD 5 mm CPBBO BB-
PULPROG zgdc30
TD 65536
SOLVENT CDCl3T
NS 303
DS 4
SWH 36231.883 Hz
FIDRES 0.552855 Hz
AQ 0.9043968 sec
RG 2050
DW 13.800 usec
DE 19.65 usec
TE 297.9 K
D1 0.40000001 sec
D11 0.03000000 sec
TD0 1

======== CHANNEL f1 ========
SFO1 150.9194080 MHz
NUC1 13C
P1 10.00 usec
PLW1 68.40000153 W

======== CHANNEL f2 ========
SFO2 600.1330010 MHz
NUC2 1H
CPDPRG[2 waltz16
PCPD2 80.00 usec
PLW2 30.00000000 W
PLW12 0.39811000 W

F2 - Processing parameters
SI 65536
SF 150.9028185 MHz
WDW EM
SSB 0
LB 1.00 Hz
GB 0
PC 1.00

1H spectrum

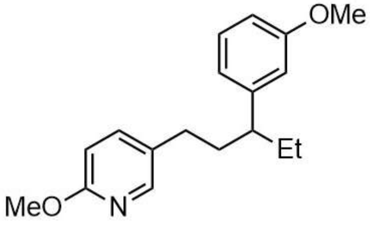

22

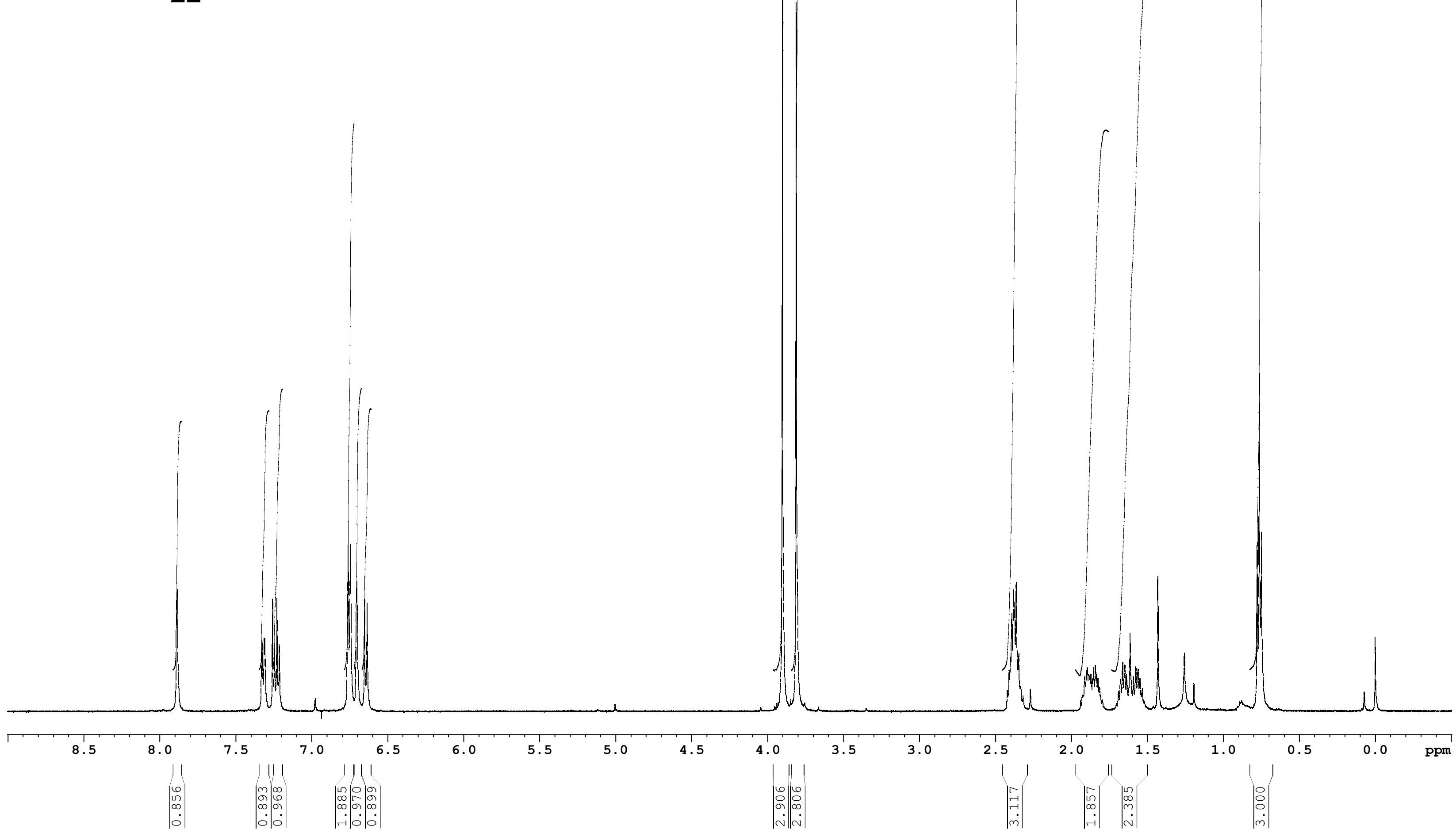


```
Current Data Parameters
NAME          cdw-2-279-p
EXPNO                   1
PROCNO                  1

F2 - Acquisition Parameters
Date_            20240308
Time                 8.03
INSTRUM             gn500
PROBHD   5 mm broadband
PULPROG              zg30
TD                  81728
SOLVENT             CDCl3
NS                      8
DS                      2
SWH            8012.820 Hz
FIDRES         0.098043 Hz
AQ            5.0998273 sec
RG                  645.1
DW               62.400 usec
DE                 6.00 usec
TE                298.0 K
D1           0.10000000 sec
MCREST                0 sec
MCWRK        0.01500000 sec

======== CHANNEL f1 ========
NUC1                   1H
P1                12.00 usec
PL1               -6.00 dB
SFO1        498.4534891 MHz

F2 - Processing parameters
SI                  65536
SF          498.4500316 MHz
WDW                    no
SSB                     0
LB                      0 Hz
GB                      0
PC                   1.00
```

13C spectrum with 1H decoupling

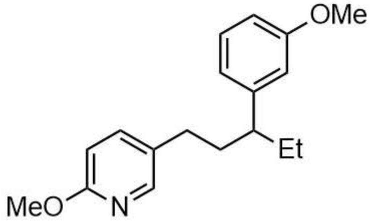

22

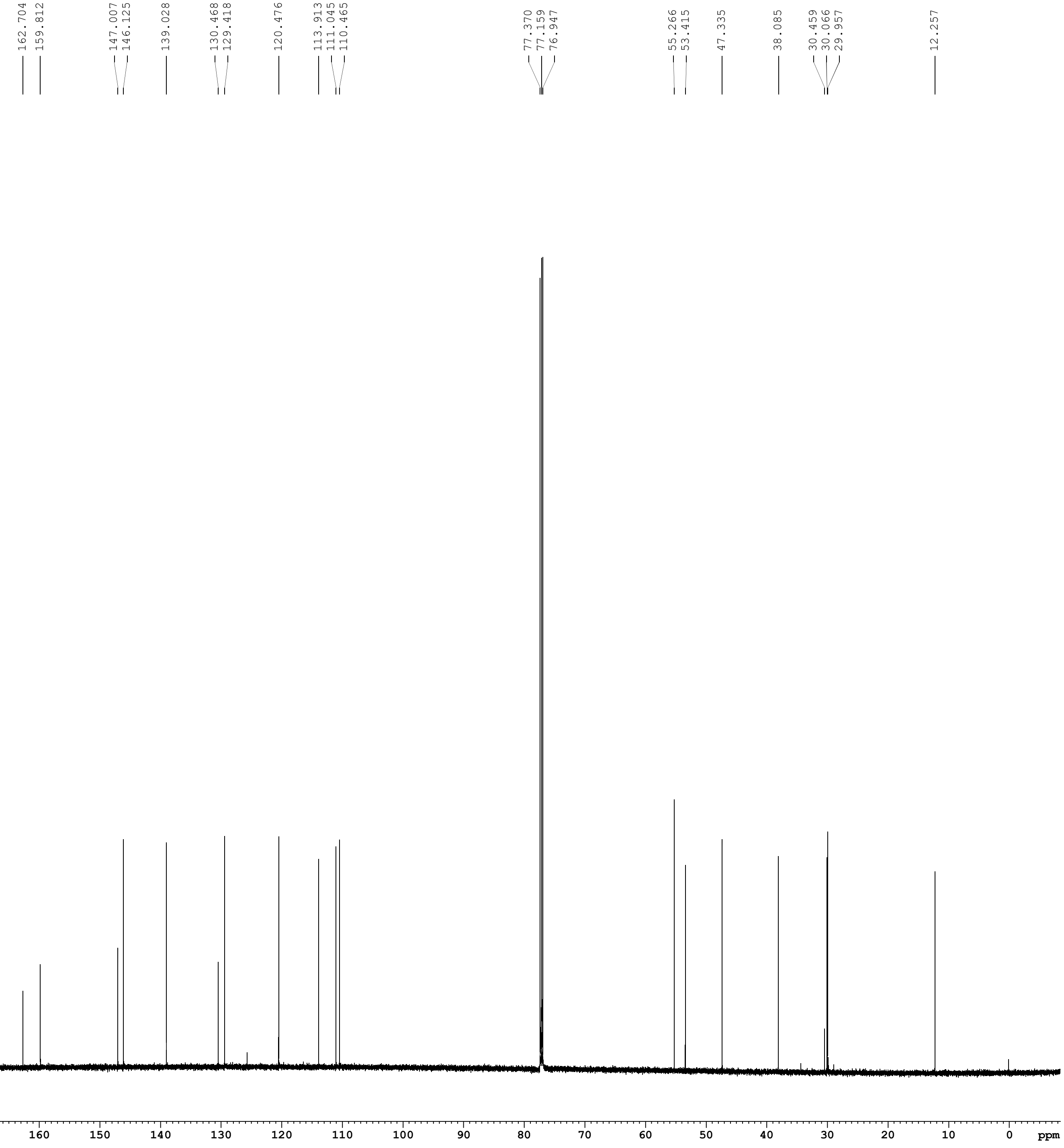


```
Current Data Parameters
NAME      cdw-2-279-c13
EXPNO                 1
PROCNO                1

F2 - Acquisition Parameters
Date_          20240308
Time               8.25
INSTRUM           av600
PROBHD   5 mm CPBBO BB-
PULPROG          zgdc30
TD                65536
SOLVENT           CDCl3
NS                  215
DS                    4
SWH           36231.883 Hz
FIDRES         0.552855 Hz
AQ            0.9043968 sec
RG                 2050
DW               13.800 usec
DE                19.65 usec
TE                298.1 K
D1           0.40000001 sec
D11          0.03000000 sec
TD0                   1

======== CHANNEL f1 ========
SFO1        150.9194080 MHz
NUC1                13C
P1                10.00 usec
PLW1        68.40000153 W

======== CHANNEL f2 ========
SFO2        600.1330010 MHz
NUC2                 1H
CPDPRG[2        waltz16
PCPD2             80.00 usec
PLW2        30.00000000 W
PLW12        0.39811000 W

F2 - Processing parameters
SI                65536
SF          150.9027947 MHz
WDW                  no
SSB                   0
LB                    0 Hz
GB                    0
PC                 1.00
```

1H spectrum

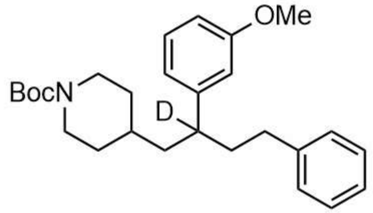

D-23

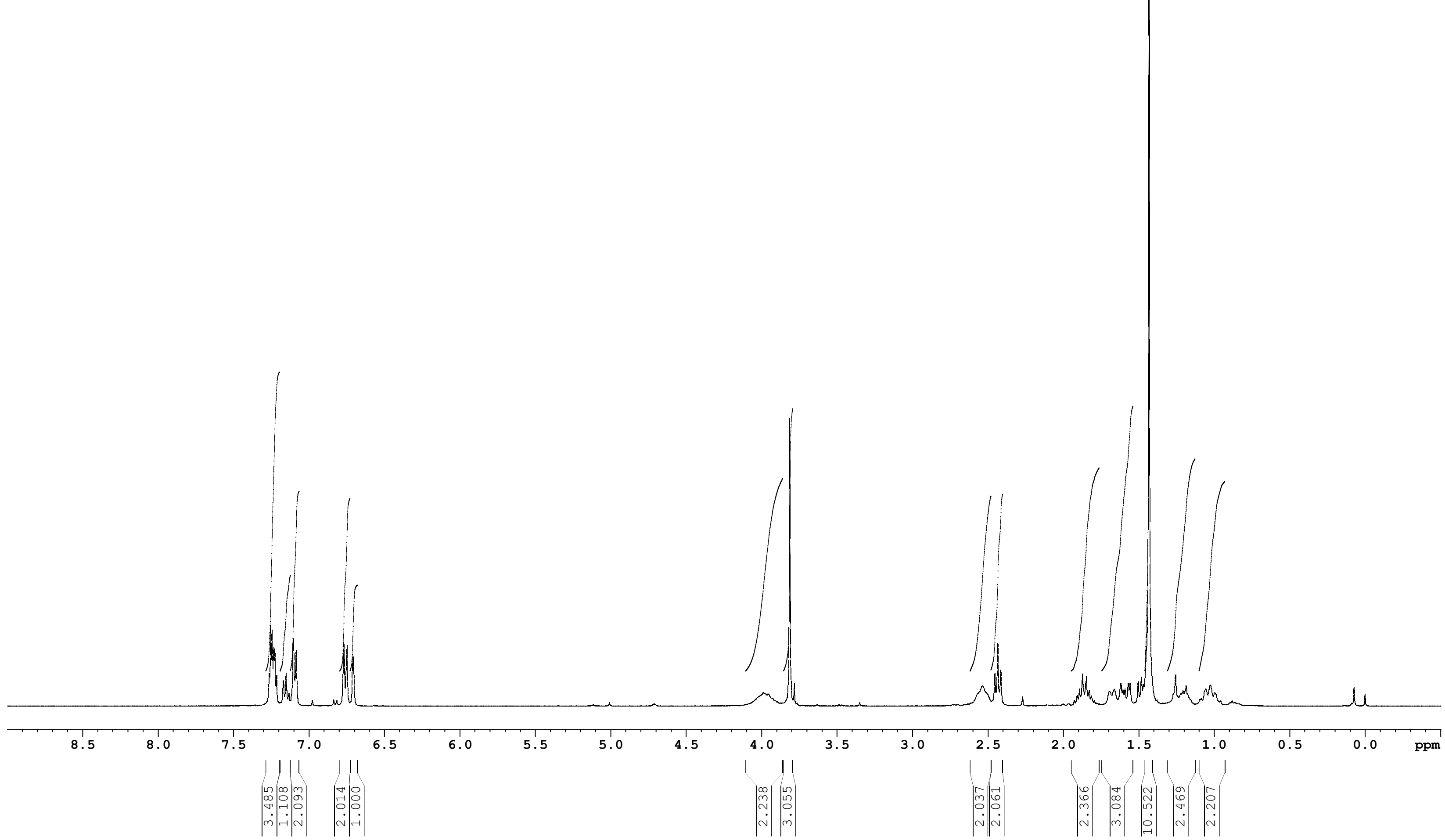


```
Current Data Parameters
NAME      cdw-3-45-4c-5
EXPNO                 1
PROCNO                1

F2 - Acquisition Parameters
Date_          20240415
Time              14.27 h
INSTRUM         NEO 400
PROBHD   Z163739_0122 (
PULPROG            zg30
TD                65536
SOLVENT           CDCl3
NS                    8
DS                    2
SWH            6250.000 Hz
FIDRES         0.190735 Hz
AQ            5.2428799 sec
RG                  101
DW               80.000 usec
DE                 8.64 usec
TE                298.3 K
D1           0.10000000 sec
TD0                   1
SFO1        400.1328009 MHz
NUC1                 1H
P0                 2.67 usec
P1                 8.00 usec
PLW1        23.01399994 W

F2 - Processing parameters
SI                65536
SF          400.1300243 MHz
WDW                  no
SSB                   0
LB                    0 Hz
GB                    0
PC                 1.00
```

13C spectrum with 1H decoupling

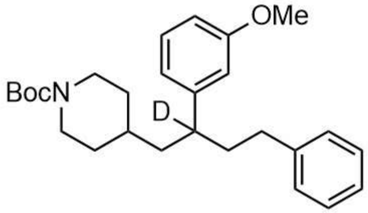

D-26

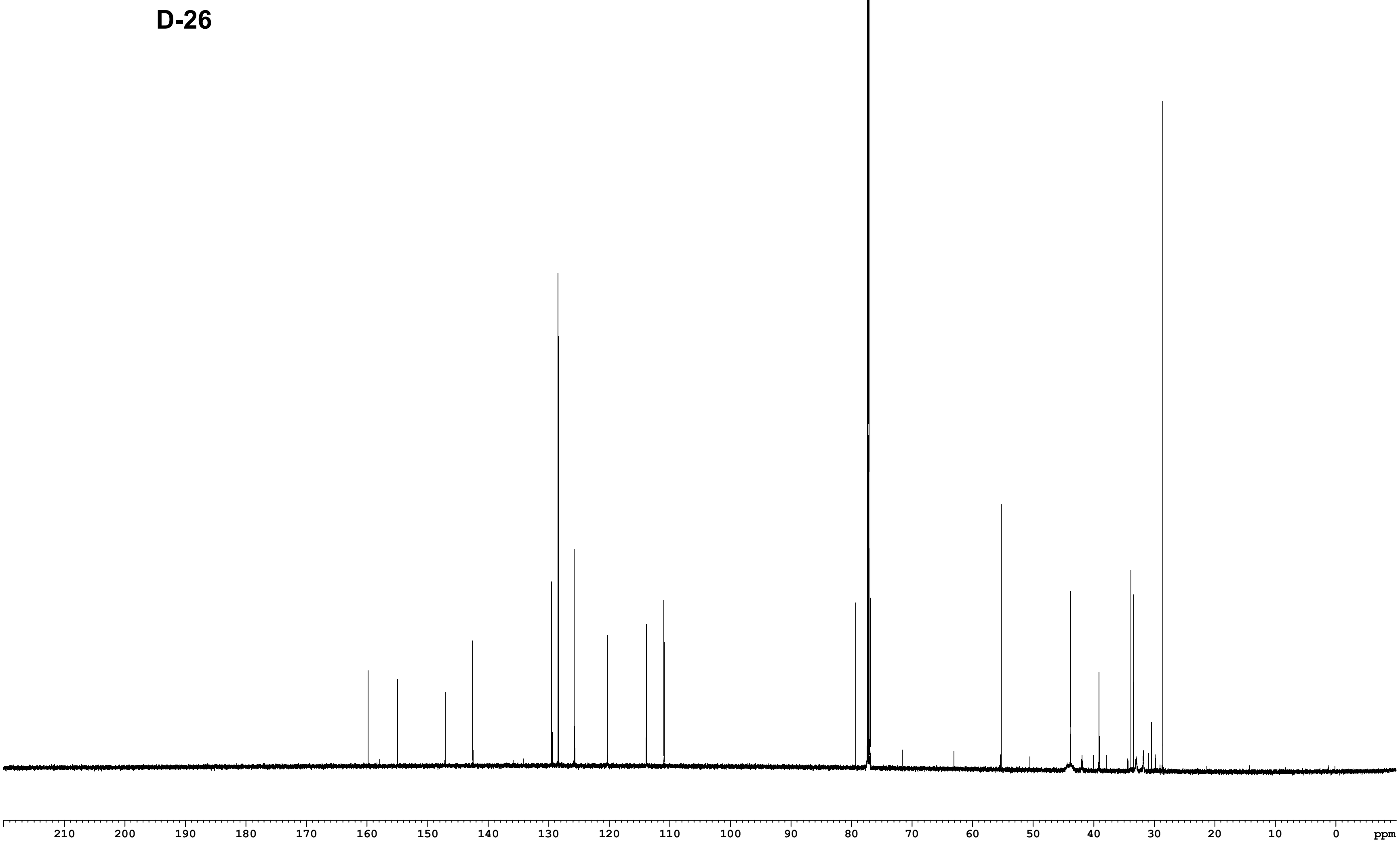

Current Data Parameters
NAME cdw-3-45-c13
EXPNO 1
PROCNO 1

F2 - Acquisition Parameters
Date_ 20240416
Time 10.55
INSTRUM av600
PROBHD 5 mm CPBBO BB-
PULPROG zgdc30
TD 65536
SOLVENT CDCl3
NS 400
DS 4
SWH 36231.883 Hz
FIDRES 0.552855 Hz
AQ 0.9043968 sec
RG 2050
DW 13.800 usec
DE 19.65 usec
TE 298.1 K
D1 0.40000001 sec
D11 0.03000000 sec
TD0 1

======== CHANNEL f1 ========
SFO1 150.9194080 MHz
NUC1 13C
P1 10.00 usec
PLW1 68.40000153 W

======== CHANNEL f2 ========
SFO2 600.1330010 MHz
NUC2 1H
CPDPRG[2 waltz16
PCPD2 80.00 usec
PLW2 30.00000000 W
PLW12 0.39811000 W

F2 - Processing parameters
SI 65536
SF 150.9027984 MHz
WDW no
SSB 0
LB 0 Hz
GB 0
PC 1.00

1H spectrum

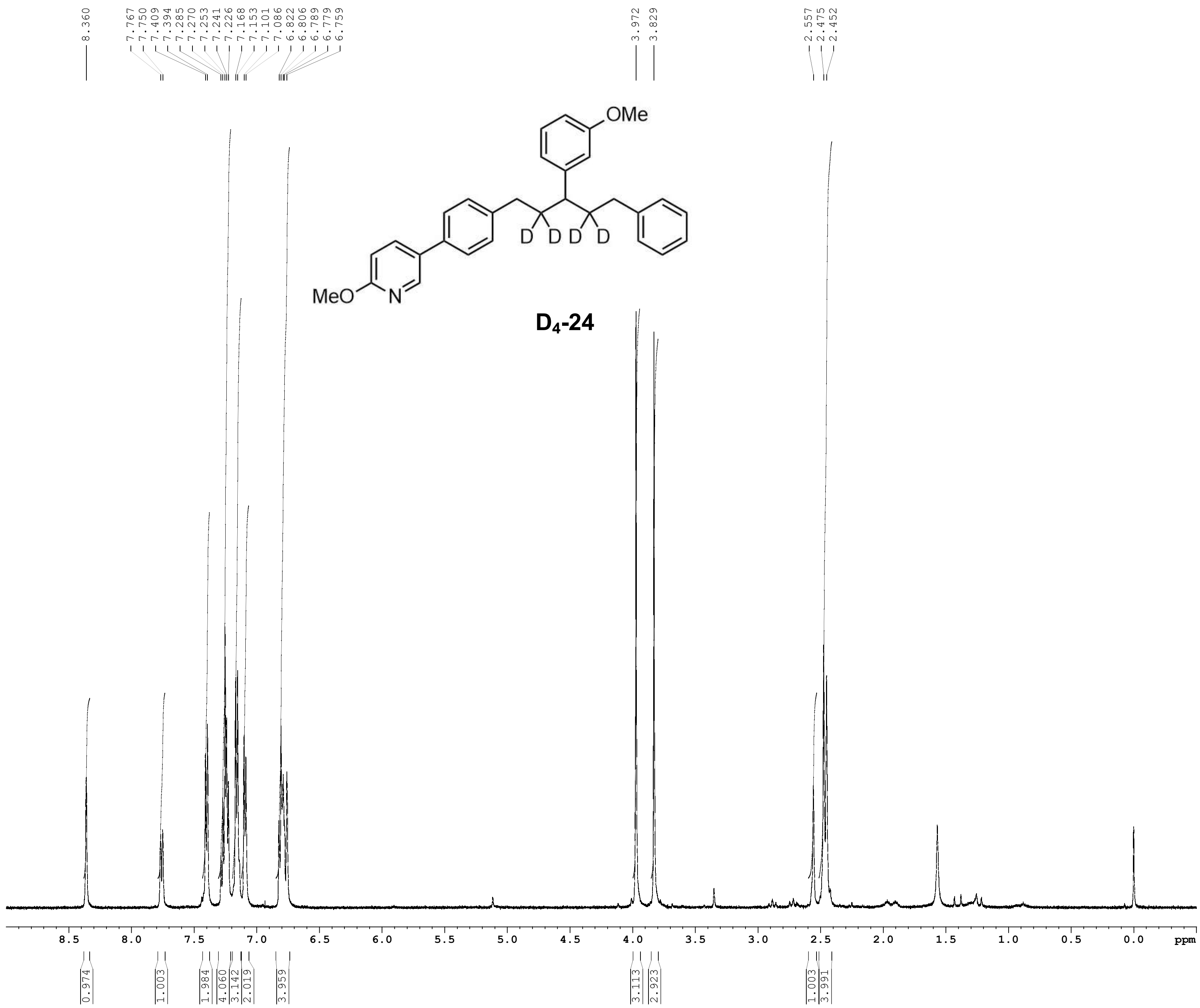


```
Current Data Parameters
NAME       cdw-2-295-p2
EXPNO                 1
PROCNO                1

F2 - Acquisition Parameters
Date_          20240403
Time               8.40
INSTRUM           gn500
PROBHD   5 mm broadband
PULPROG            zg30
TD                81728
SOLVENT           CDCl3
NS                    8
DS                    2
SWH            8012.820 Hz
FIDRES         0.098043 Hz
AQ            5.0998273 sec
RG                912.3
DW               62.400 usec
DE                 6.00 usec
TE                298.0 K
D1           0.10000000 sec
MCREST                0 sec
MCWRK        0.01500000 sec

======== CHANNEL f1 ========
NUC1                 1H
P1                12.00 usec
PL1               -6.00 dB
SFO1        498.4534891 MHz

F2 - Processing parameters
SI                65536
SF          498.4500326 MHz
WDW                  no
SSB                   0
LB                    0 Hz
GB                    0
PC                 1.00
```

13C spectrum with 1H decoupling

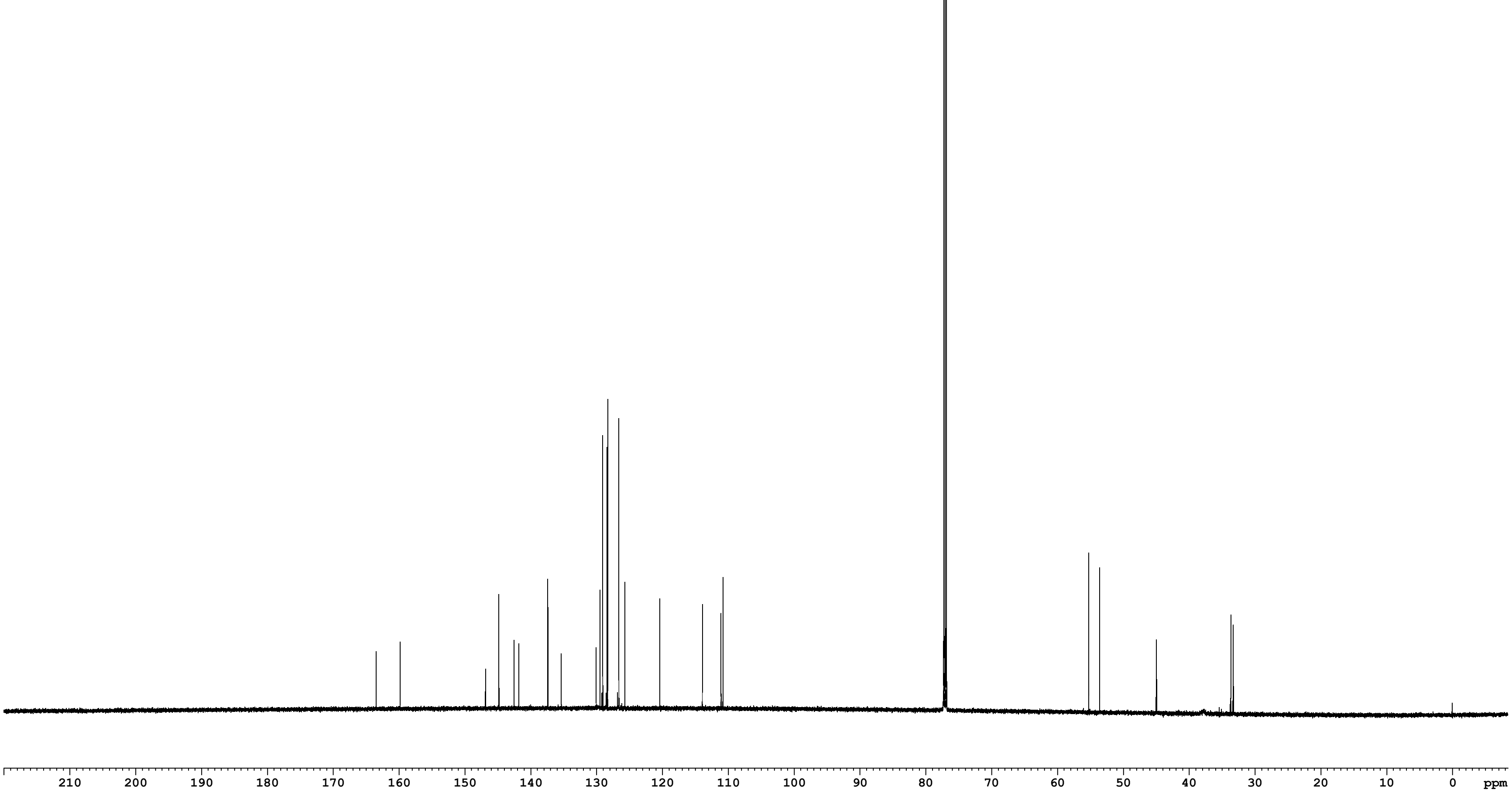

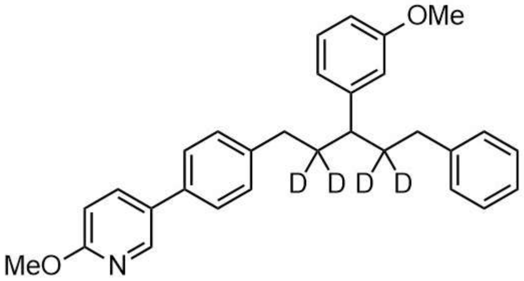

D_4-24

```
Current Data Parameters
NAME        cdw-2-295-c13
EXPNO                   1
PROCNO                  1

F2 - Acquisition Parameters
Date_            20240403
Time                 9.02
INSTRUM             av600
PROBHD   5 mm CPBBO BB-
PULPROG            zgdc30
TD                  65536
SOLVENT             CDCl3
NS                    570
DS                      4
SWH             36231.883 Hz
FIDRES           0.552855 Hz
AQ              0.9043968 sec
RG                   2050
DW                 13.800 usec
DE                  19.65 usec
TE                  298.1 K
D1             0.40000001 sec
D11            0.03000000 sec
TD0                     1

======== CHANNEL f1 ========
SFO1          150.9194080 MHz
NUC1                  13C
P1                  10.00 usec
PLW1          68.40000153 W

======== CHANNEL f2 ========
SFO2          600.1330010 MHz
NUC2                   1H
CPDPRG[2          waltz16
PCPD2               80.00 usec
PLW2          30.00000000 W
PLW12          0.39811000 W

F2 - Processing parameters
SI                  65536
SF            150.9028085 MHz
WDW                    no
SSB                     0
LB                      0 Hz
GB                      0
PC                   1.00
```

1H spectrum

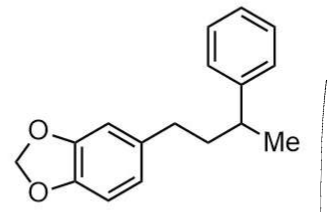

25

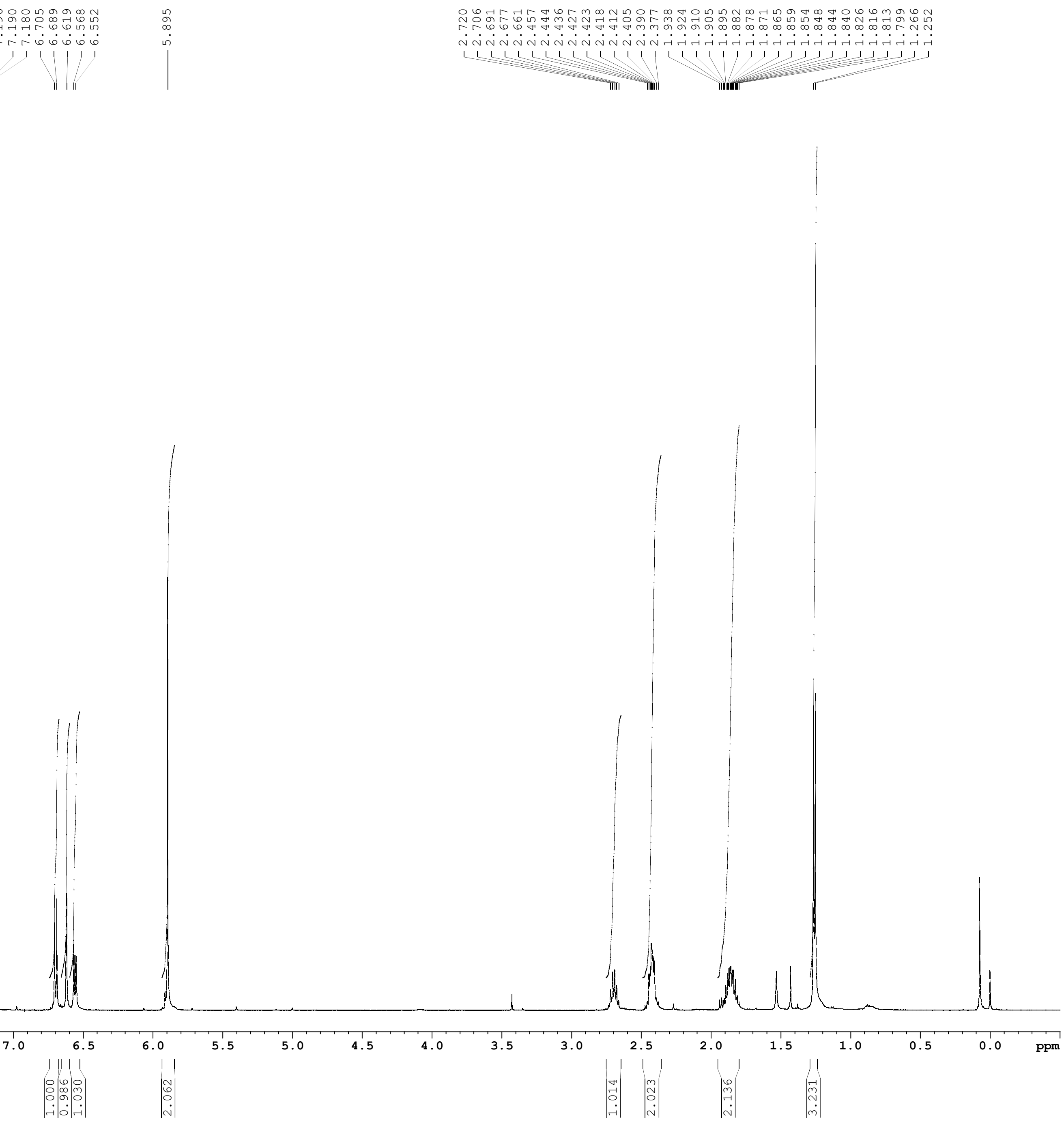


```
Current Data Parameters
NAME        cdw-3-105-p
EXPNO                 1
PROCNO                1

F2 - Acquisition Parameters
Date_          20240610
Time               8.22
INSTRUM           gn500
PROBHD   5 mm broadband
PULPROG            zg30
TD                81728
SOLVENT           CDCl3
NS                    8
DS                    2
SWH            8012.820 Hz
FIDRES         0.098043 Hz
AQ            5.0998273 sec
RG                161.3
DW               62.400 usec
DE                 6.00 usec
TE                298.1 K
D1           0.10000000 sec
MCREST                0 sec
MCWRK        0.01500000 sec

======== CHANNEL f1 ========
NUC1                 1H
P1                12.00 usec
PL1               -6.00 dB
SFO1        498.4534891 MHz

F2 - Processing parameters
SI                65536
SF          498.4500391 MHz
WDW                  no
SSB                   0
LB                    0 Hz
GB                    0
PC                 1.00
```

1H spectrum

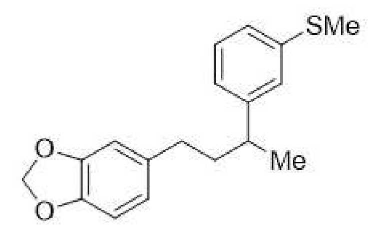

26

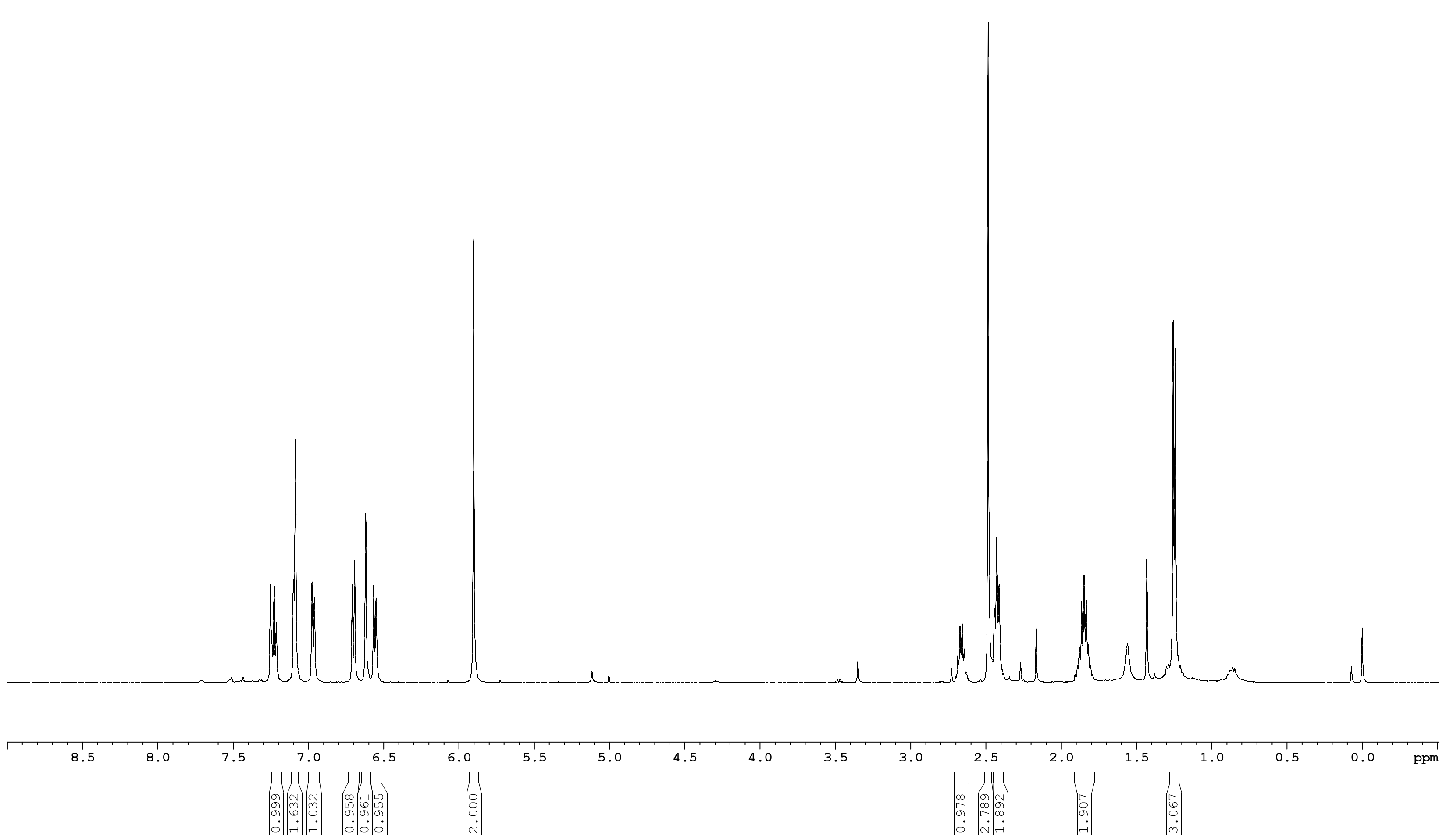


```
Current Data Parameters
NAME     LCB-1-64-ge-dry
EXPNO                  1
PROCNO                 1

F2 - Acquisition Parameters
Date_           20240716
Time                9.13
INSTRUM            gn500
PROBHD   5 mm broadband
PULPROG             zg30
TD                 81728
SOLVENT           CDCl3T
NS                     8
DS                     2
SWH             8012.820 Hz
FIDRES          0.098043 Hz
AQ             5.0998273 sec
RG                 574.7
DW                62.400 usec
DE                  6.00 usec
TE                 298.0 K
D1            0.10000000 sec
MCREST                 0 sec
MCWRK         0.01500000 sec

======== CHANNEL f1 ========
NUC1                  1H
P1                 12.00 usec
PL1                -6.00 dB
SFO1         498.4534891 MHz

F2 - Processing parameters
SI                 65536
SF           498.4500337 MHz
WDW                   EM
SSB                    0
LB                  0.30 Hz
GB                     0
PC                  1.00
```

13C spectrum with 1H decoupling

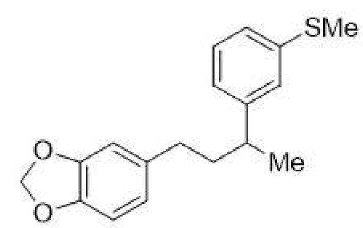

26

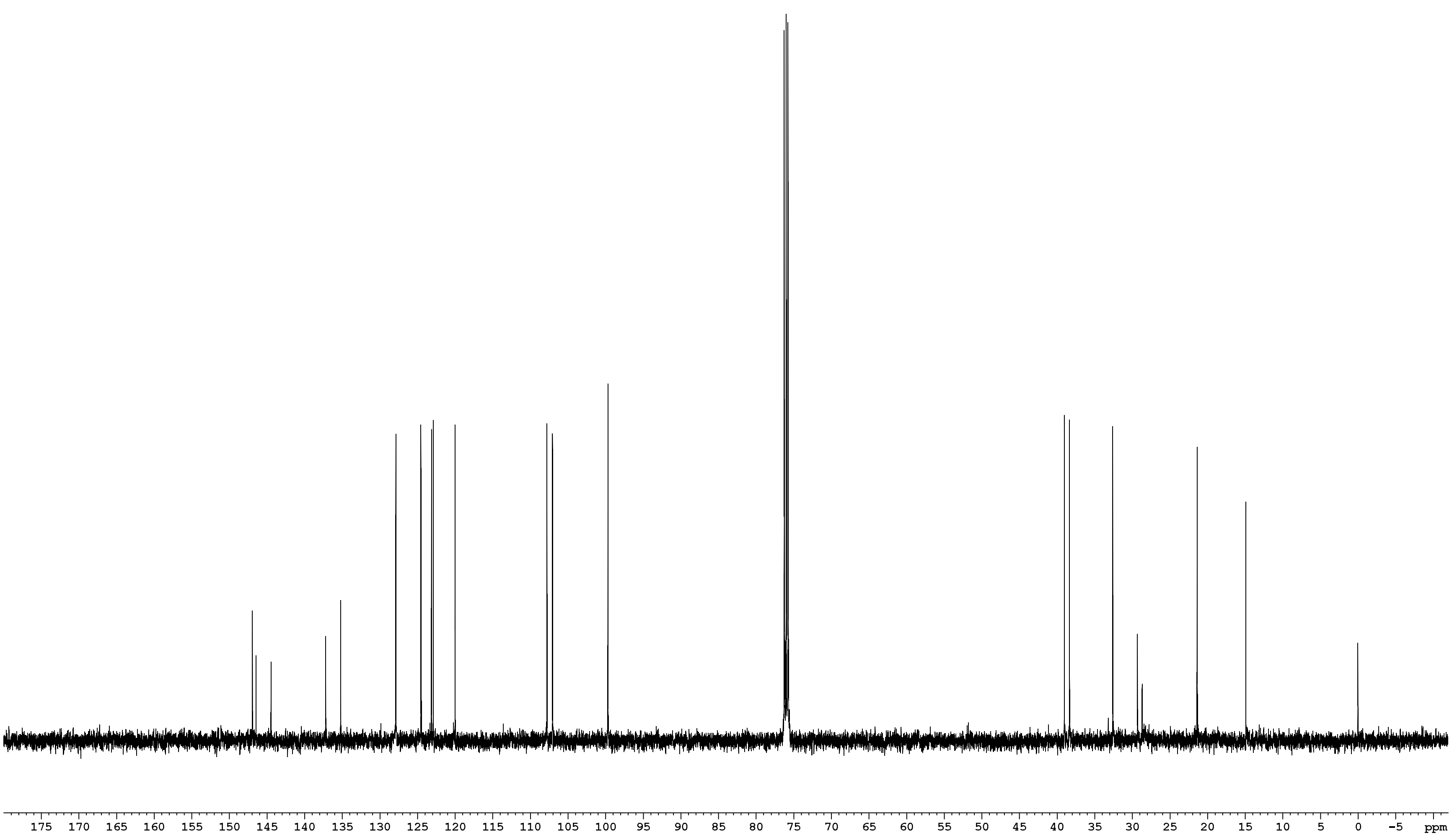


```
Current Data Parameters
NAME     LCB-1-64-500C-2
EXPNO                  1
PROCNO                 1

F2 - Acquisition Parameters
Date_           20240709
Time                9.34
INSTRUM            gn500
PROBHD   5 mm broadband
PULPROG           zgdc30
TD                 65536
SOLVENT           CDCl3T
NS                   505
DS                     4
SWH            30303.031 Hz
FIDRES          0.462388 Hz
AQ             1.0813440 sec
RG                5792.6
DW                16.500 usec
DE                  6.00 usec
TE                 298.0 K
D1            0.25000000 sec
d11           0.03000000 sec
MCREST                 0 sec
MCWRK         0.01500000 sec

======== CHANNEL f1 ========
NUC1                 13C
P1                 14.20 usec
PL1                -6.00 dB
SFO1         125.3491398 MHz

======== CHANNEL f2 ========
CPDPRG[2         waltz16
NUC2                  1H
PCPD2             100.00 usec
PL2                -6.00 dB
PL12               12.30 dB
SFO2         498.4524922 MHz

F2 - Processing parameters
SI                 65536
SF           125.3354873 MHz
WDW                   EM
SSB                    0
LB                  1.00 Hz
GB                     0
PC                  2.00
```

1H spectrum

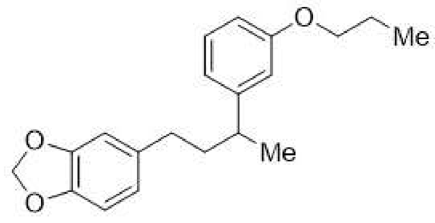

27

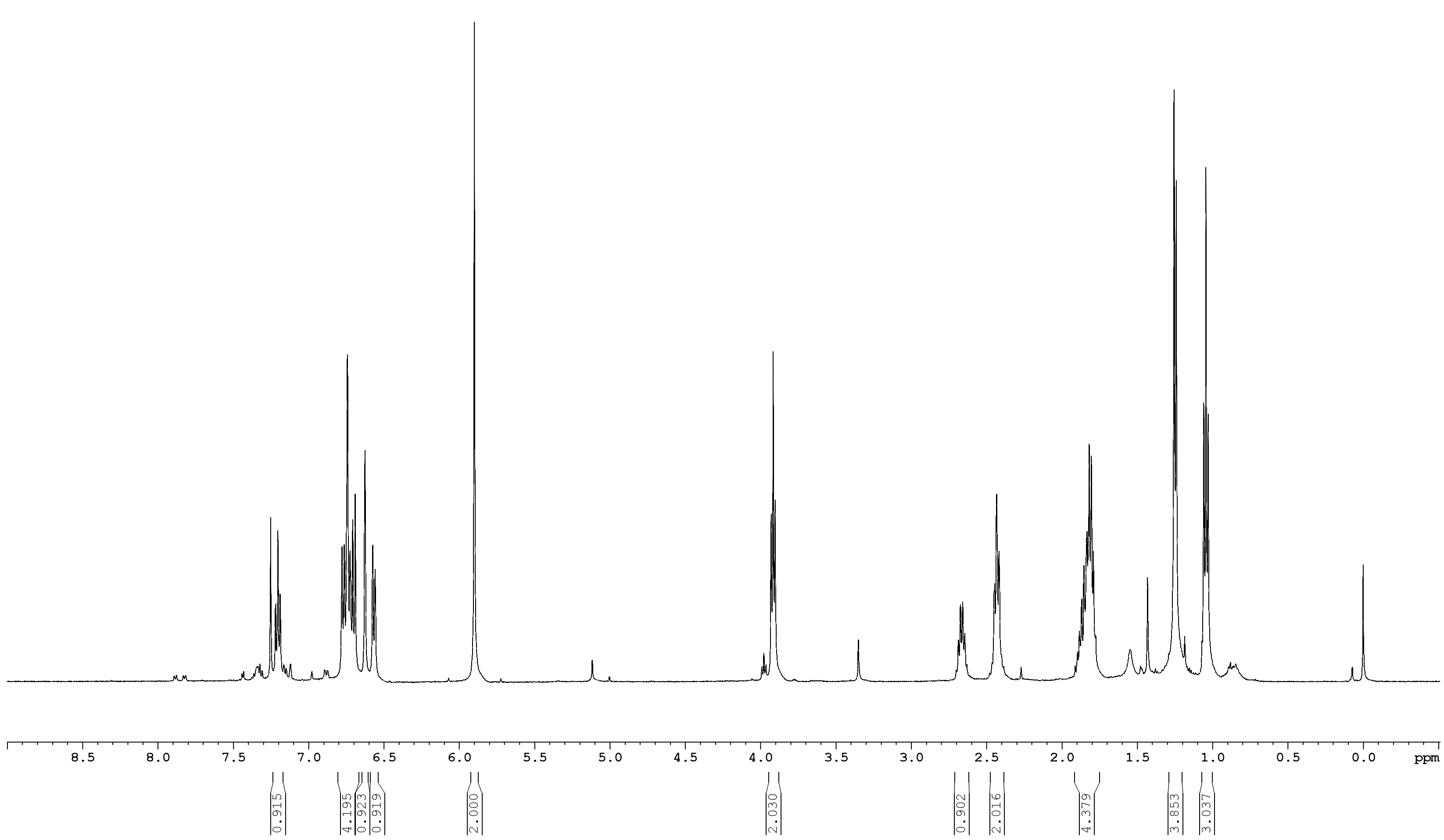

Current Data Parameters
NAME LCB-1-65-ge-dry4
EXPNO 1
PROCNO 1

F2 - Acquisition Parameters
Date_ 20240815
Time 15.36
INSTRUM gn500
PROBHD 5 mm broadband
PULPROG zg30
TD 81728
SOLVENT CDCl3T
NS 8
DS 2
SWH 8012.820 Hz
FIDRES 0.098043 Hz
AQ 5.0998273 sec
RG 574.7
DW 62.400 usec
DE 6.00 usec
TE 298.0 K
D1 0.10000000 sec
MCREST 0 sec
MCWRK 0.01500000 sec

======== CHANNEL f1 ========
NUC1 1H
P1 12.00 usec
PL1 -6.00 dB
SFO1 498.4534891 MHz

F2 - Processing parameters
SI 65536
SF 498.4500339 MHz
WDW EM
SSB 0
LB 0.30 Hz
GB 0
PC 1.00

13C spectrum with 1H decoupling

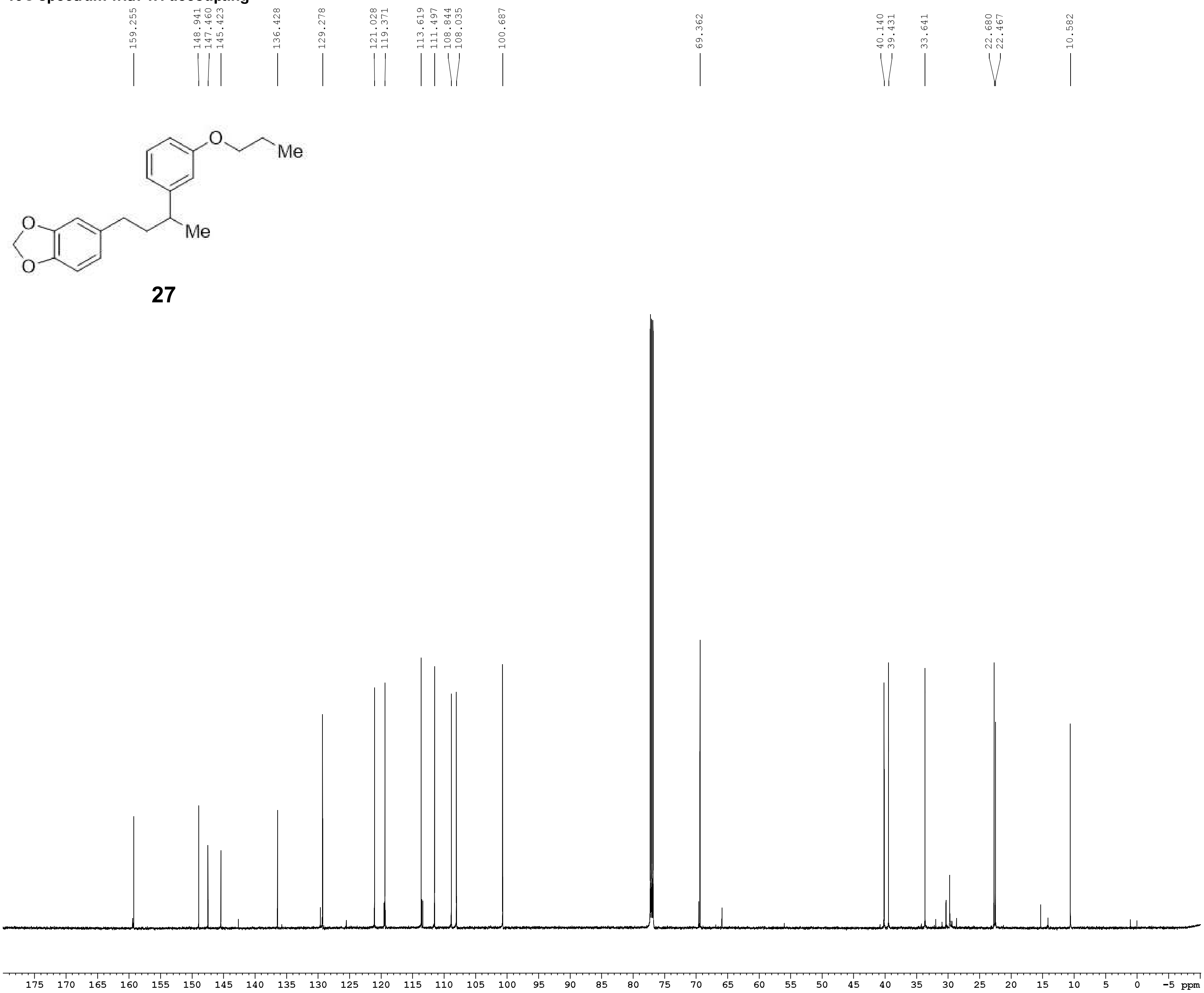

Current Data Parameters
NAME LCB-1-600C
EXPNO 1
PROCNO 1

F2 - Acquisition Parameters
Date_ 20240712
Time 14.49
INSTRUM av600
PROBHD 5 mm CPBBO BB-
PULPROG zgdc30
TD 65536
SOLVENT CDCl3T
NS 397
DS 4
SWH 36231.883 Hz
FIDRES 0.552855 Hz
AQ 0.9043968 sec
RG 2050
DW 13.800 usec
DE 19.65 usec
TE 298.0 K
D1 0.40000001 sec
D11 0.03000000 sec
TD0 1

======== CHANNEL f1 ========
SFO1 150.9194080 MHz
NUC1 13C
P1 10.00 usec
PLW1 68.40000153 W

======== CHANNEL f2 ========
SFO2 600.1330010 MHz
NUC2 1H
CPDPRG[2 waltz16
PCPD2 80.00 usec
PLW2 30.00000000 W
PLW12 0.39811000 W

F2 - Processing parameters
SI 65536
SF 150.9028167 MHz
WDW EM
SSB 0
LB 1.00 Hz
GB 0
PC 1.00

1H spectrum

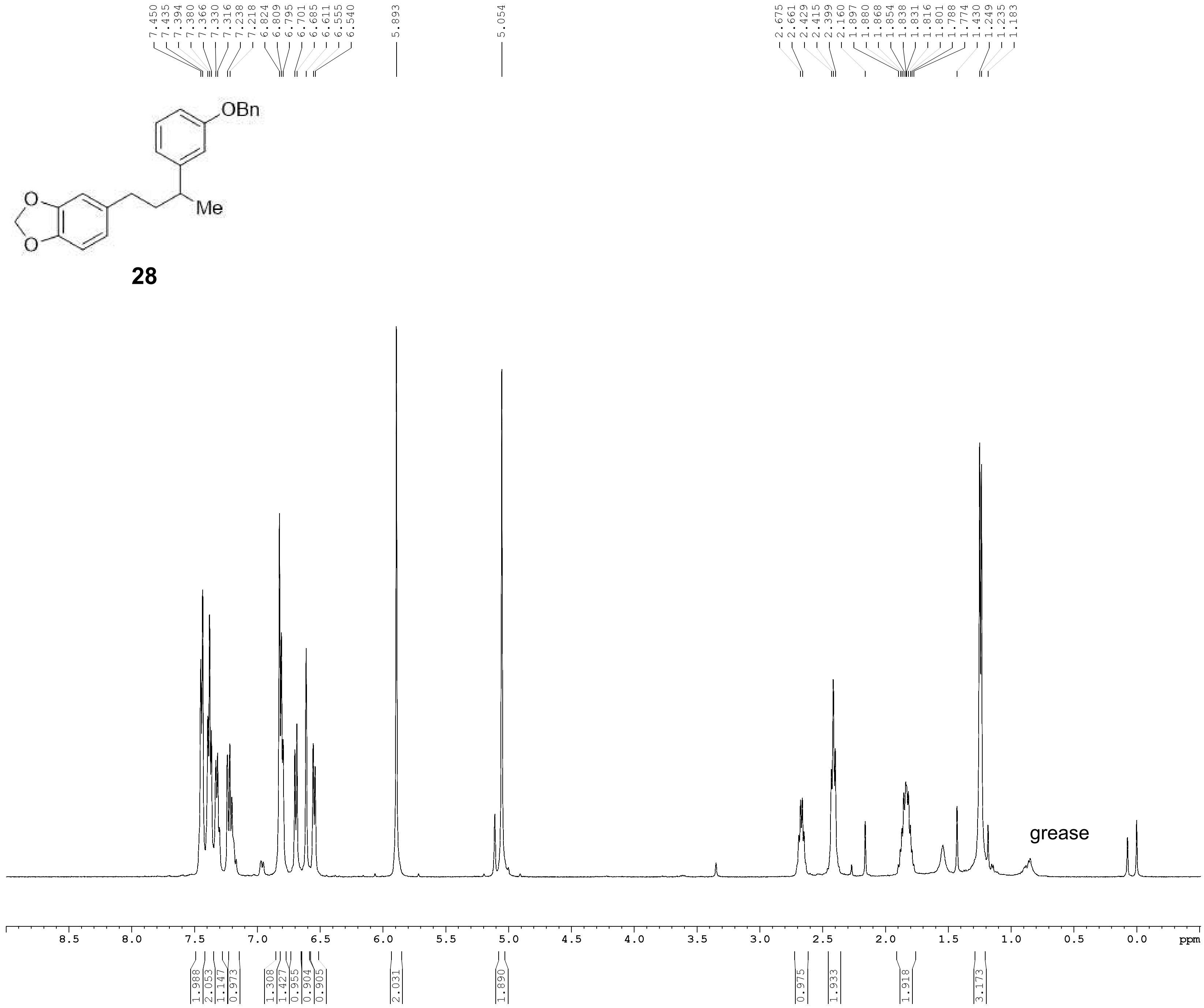


```
Current Data Parameters
NAME        LCB-1-83-ge
EXPNO                 1
PROCNO                1

F2 - Acquisition Parameters
Date_          20240718
Time              10.04
INSTRUM           gn500
PROBHD   5 mm broadband
PULPROG            zg30
TD                81728
SOLVENT          CDCl3T
NS                    8
DS                    2
SWH            8012.820 Hz
FIDRES         0.098043 Hz
AQ            5.0998273 sec
RG                161.3
DW               62.400 usec
DE                 6.00 usec
TE                298.0 K
D1           0.10000000 sec
MCREST                0 sec
MCWRK        0.01500000 sec

======== CHANNEL f1 ========
NUC1                 1H
P1                12.00 usec
PL1               -6.00 dB
SFO1        498.4534891 MHz

F2 - Processing parameters
SI                65536
SF          498.4500401 MHz
WDW                  EM
SSB                   0
LB                 0.30 Hz
GB                    0
PC                 1.00
```

13C spectrum with 1H decoupling

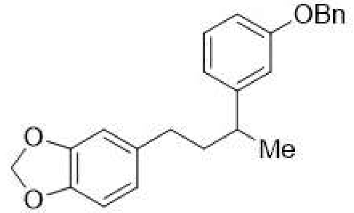

28

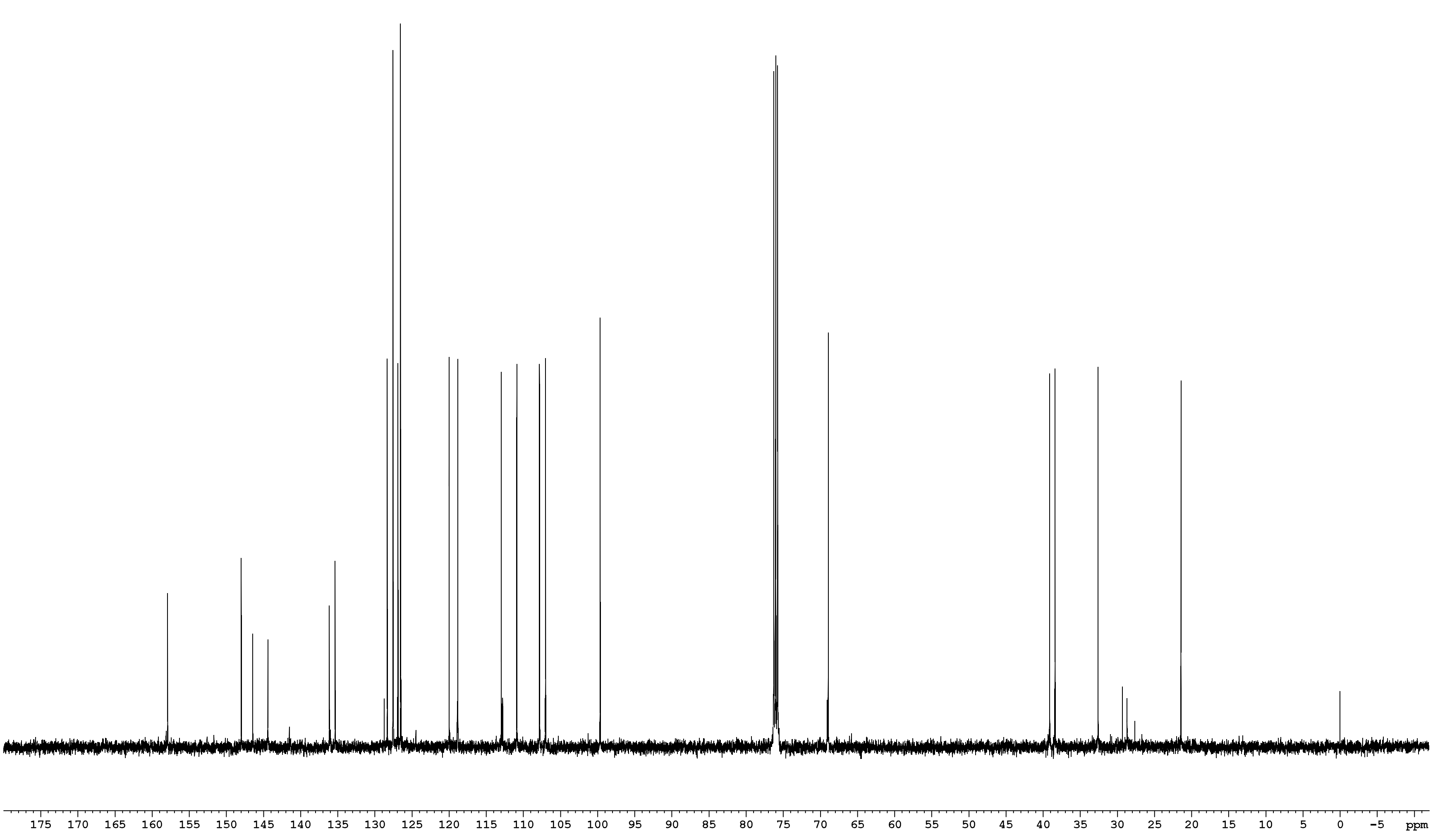


```
Current Data Parameters
NAME      LCB-1-83-500C
EXPNO                 1
PROCNO                1

F2 - Acquisition Parameters
Date_          20240716
Time               9.05
INSTRUM           gn500
PROBHD   5 mm broadband
PULPROG          zgdc30
TD                65536
SOLVENT          CDCl3T
NS                  719
DS                    4
SWH           30303.031 Hz
FIDRES         0.462388 Hz
AQ            1.0813440 sec
RG              23170.5
DW               16.500 usec
DE                 6.00 usec
TE                298.0 K
D1           0.25000000 sec
d11          0.03000000 sec
MCREST                0 sec
MCWRK        0.01500000 sec

======== CHANNEL f1 ========
NUC1                13C
P1                14.20 usec
PL1               -6.00 dB
SFO1        125.3491398 MHz

======== CHANNEL f2 ========
CPDPRG[2        waltz16
NUC2                 1H
PCPD2            100.00 usec
PL2               -6.00 dB
PL12              12.30 dB
SFO2        498.4524922 MHz

F2 - Processing parameters
SI                65536
SF          125.3354907 MHz
WDW                  EM
SSB                   0
LB                 1.00 Hz
GB                    0
PC                 2.00
```

1H spectrum

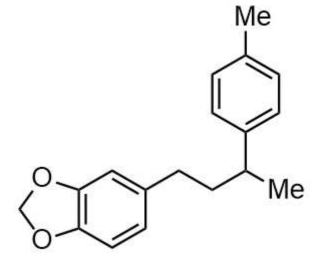

29

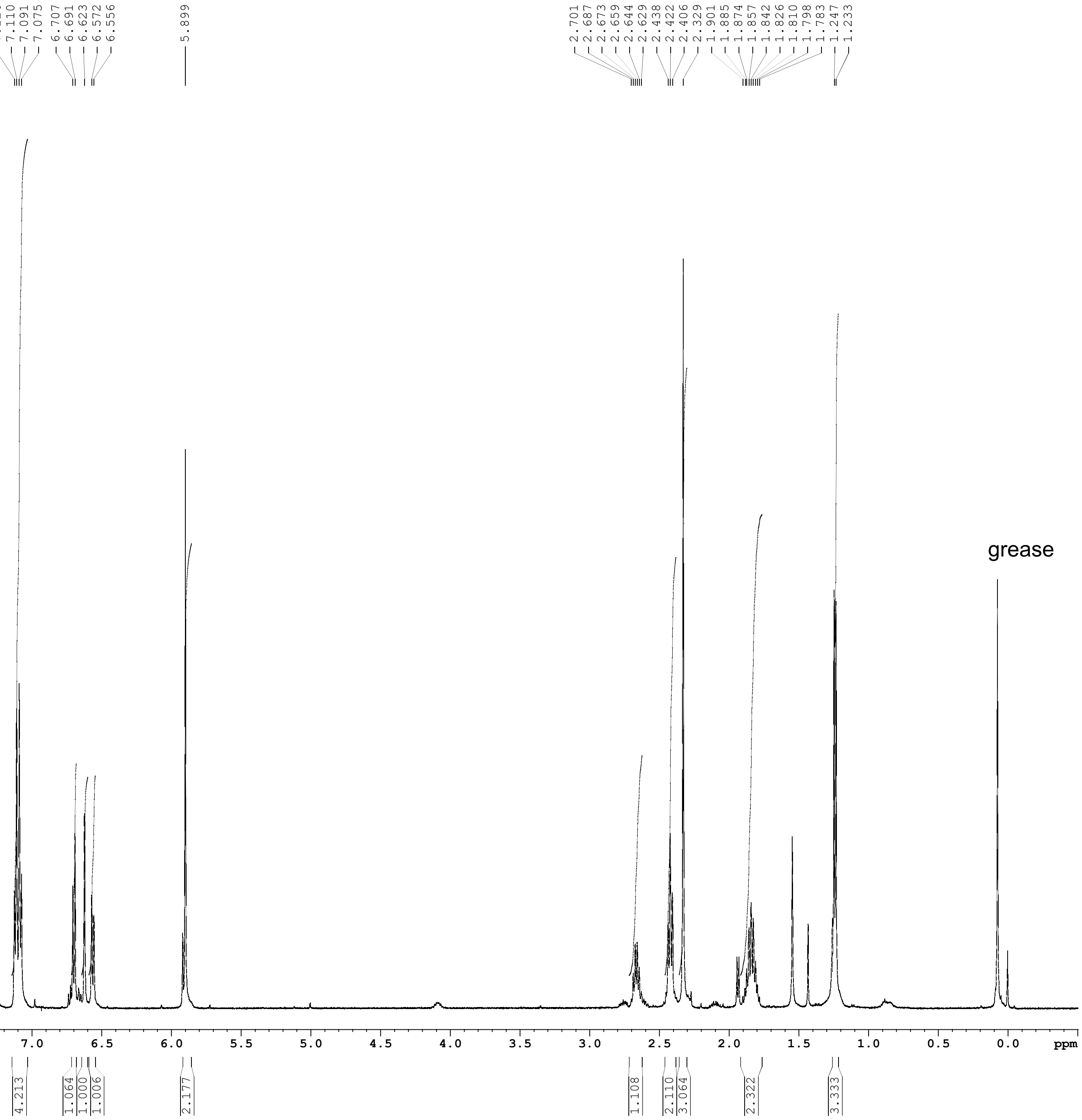


```
Current Data Parameters
NAME         cdw-3-133-p
EXPNO                  1
PROCNO                 1

F2 - Acquisition Parameters
Date_           20240629
Time               11.10
INSTRUM            gn500
PROBHD   5 mm broadband
PULPROG             zg30
TD                 81728
SOLVENT            CDCl3
NS                     8
DS                     2
SWH             8012.820 Hz
FIDRES          0.098043 Hz
AQ             5.0998273 sec
RG                 645.1
DW                62.400 usec
DE                  6.00 usec
TE                 298.0 K
D1            0.10000000 sec
MCREST                 0 sec
MCWRK         0.01500000 sec

======== CHANNEL f1 ========
NUC1                  1H
P1                 12.00 usec
PL1                -6.00 dB
SFO1         498.4534891 MHz

F2 - Processing parameters
SI                 65536
SF           498.4500350 MHz
WDW                   no
SSB                    0
LB                     0 Hz
GB                     0
PC                  1.00
```

13C spectrum with 1H decoupling

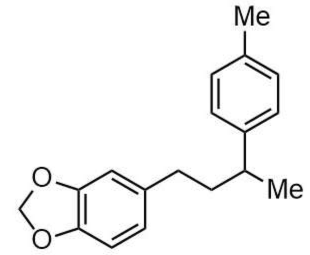

29

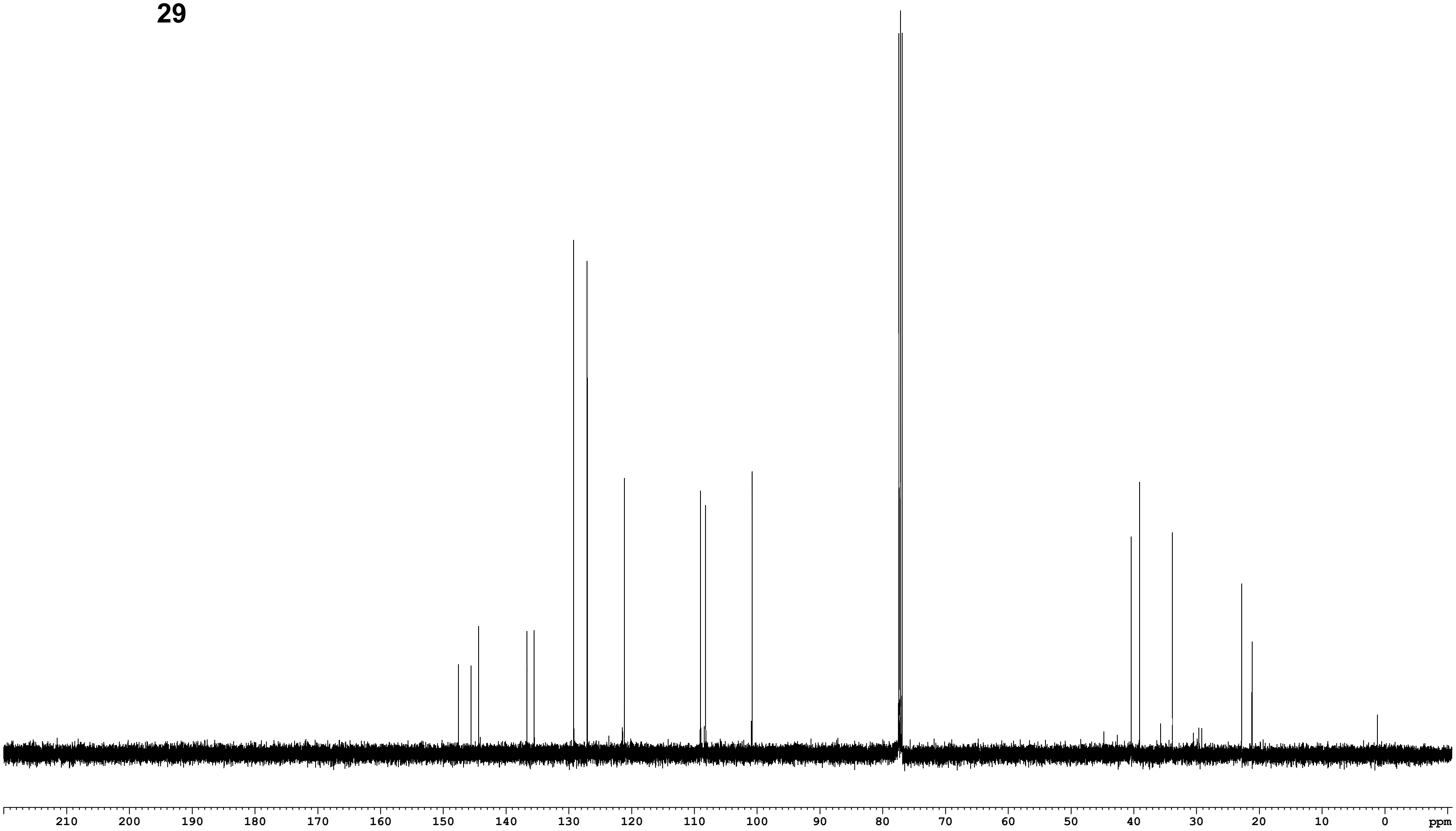


```
Current Data Parameters
NAME      cdw-3-133-c13
EXPNO                 1
PROCNO                1

F2 - Acquisition Parameters
Date_          20240629
Time              11.18
INSTRUM           gn500
PROBHD   5 mm broadband
PULPROG          zgdc30
TD                65536
SOLVENT           CDCl3
NS                  700
DS                    4
SWH           30303.031 Hz
FIDRES         0.462388 Hz
AQ            1.0813440 sec
RG                 6502
DW               16.500 usec
DE                 6.00 usec
TE                298.0 K
D1           0.25000000 sec
d11          0.03000000 sec
MCREST                0 sec
MCWRK        0.01500000 sec

======== CHANNEL f1 ========
NUC1                13C
P1                14.20 usec
PL1               -6.00 dB
SFO1        125.3491398 MHz

======== CHANNEL f2 ========
CPDPRG[2        waltz16
NUC2                 1H
PCPD2            100.00 usec
PL2               -6.00 dB
PL12              12.30 dB
SFO2        498.4524922 MHz

F2 - Processing parameters
SI                65536
SF          125.3353409 MHz
WDW                  no
SSB                   0
LB                    0 Hz
GB                    0
PC                 2.00
```

1H spectrum

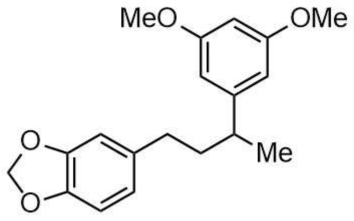

30

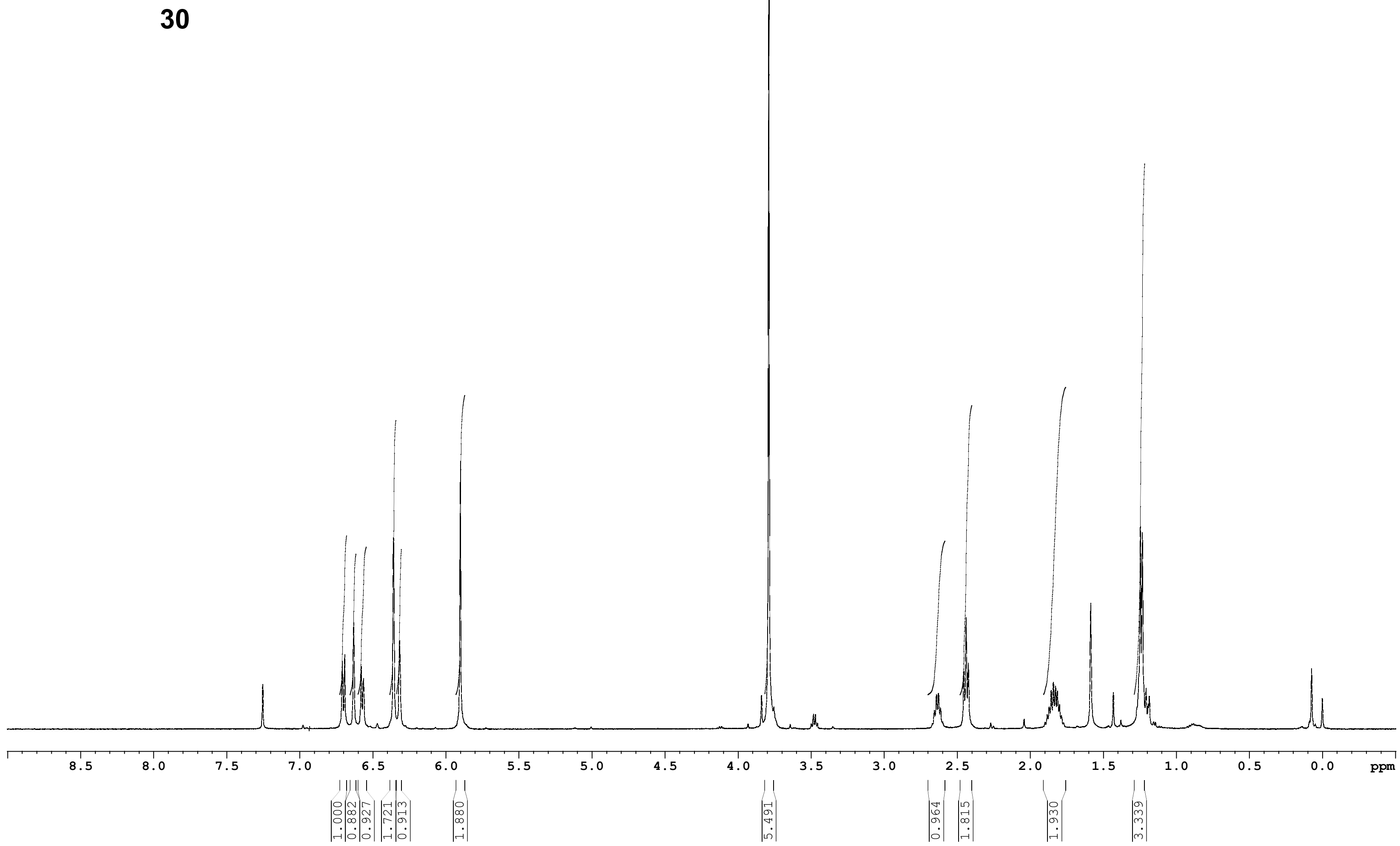


```
Current Data Parameters
NAME          cdw3-147-p
EXPNO                  1
PROCNO                 1

F2 - Acquisition Parameters
Date_           20240723
Time               10.34
INSTRUM            gn500
PROBHD   5 mm broadband
PULPROG             zg30
TD                 81728
SOLVENT            CDCl3
NS                     8
DS                     2
SWH            8012.820 Hz
FIDRES         0.098043 Hz
AQ            5.0998273 sec
RG                   181
DW               62.400 usec
DE                 6.00 usec
TE                298.0 K
D1           0.10000000 sec
MCREST                0 sec
MCWRK        0.01500000 sec

======== CHANNEL f1 ========
NUC1                  1H
P1                 12.00 usec
PL1                -6.00 dB
SFO1         498.4534891 MHz

F2 - Processing parameters
SI                 65536
SF           498.4500331 MHz
WDW                   no
SSB                    0
LB                     0 Hz
GB                     0
PC                  1.00
```

13C spectrum with 1H decoupling

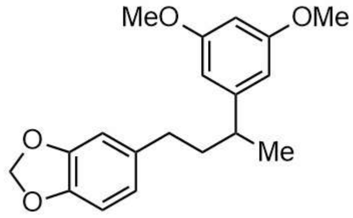

30

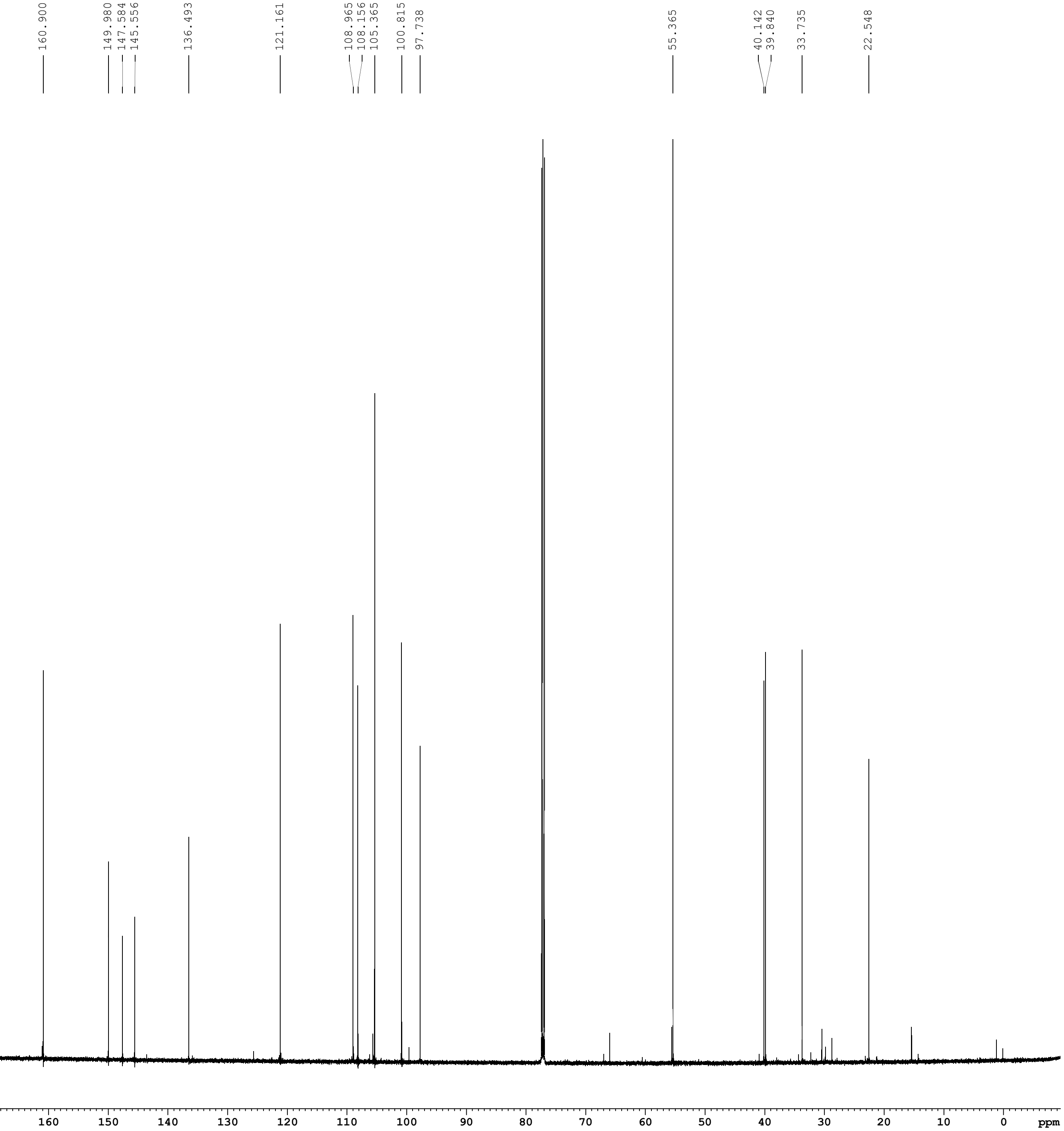


```
Current Data Parameters
NAME      cdw-3-147-c13
EXPNO                 1
PROCNO                1

F2 - Acquisition Parameters
Date_          20240725
Time               9.23
INSTRUM           av600
PROBHD   5 mm CPBBO BB-
PULPROG         zgdc30
TD               65536
SOLVENT          CDCl3
NS                 575
DS                   4
SWH          36231.883 Hz
FIDRES        0.552855 Hz
AQ           0.9043968 sec
RG                2050
DW              13.800 usec
DE               19.65 usec
TE               298.0 K
D1          0.40000001 sec
D11         0.03000000 sec
TD0                  1

======== CHANNEL f1 ========
SFO1       150.9194080 MHz
NUC1               13C
P1               10.00 usec
PLW1       68.40000153 W

======== CHANNEL f2 ========
SFO2       600.1330010 MHz
NUC2                1H
CPDPRG[2        waltz16
PCPD2            80.00 usec
PLW2       30.00000000 W
PLW12       0.39811000 W

F2 - Processing parameters
SI               65536
SF         150.9027983 MHz
WDW                 no
SSB                  0
LB                   0 Hz
GB                   0
PC                1.00
```

1H spectrum

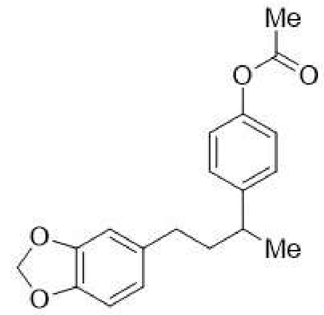

31

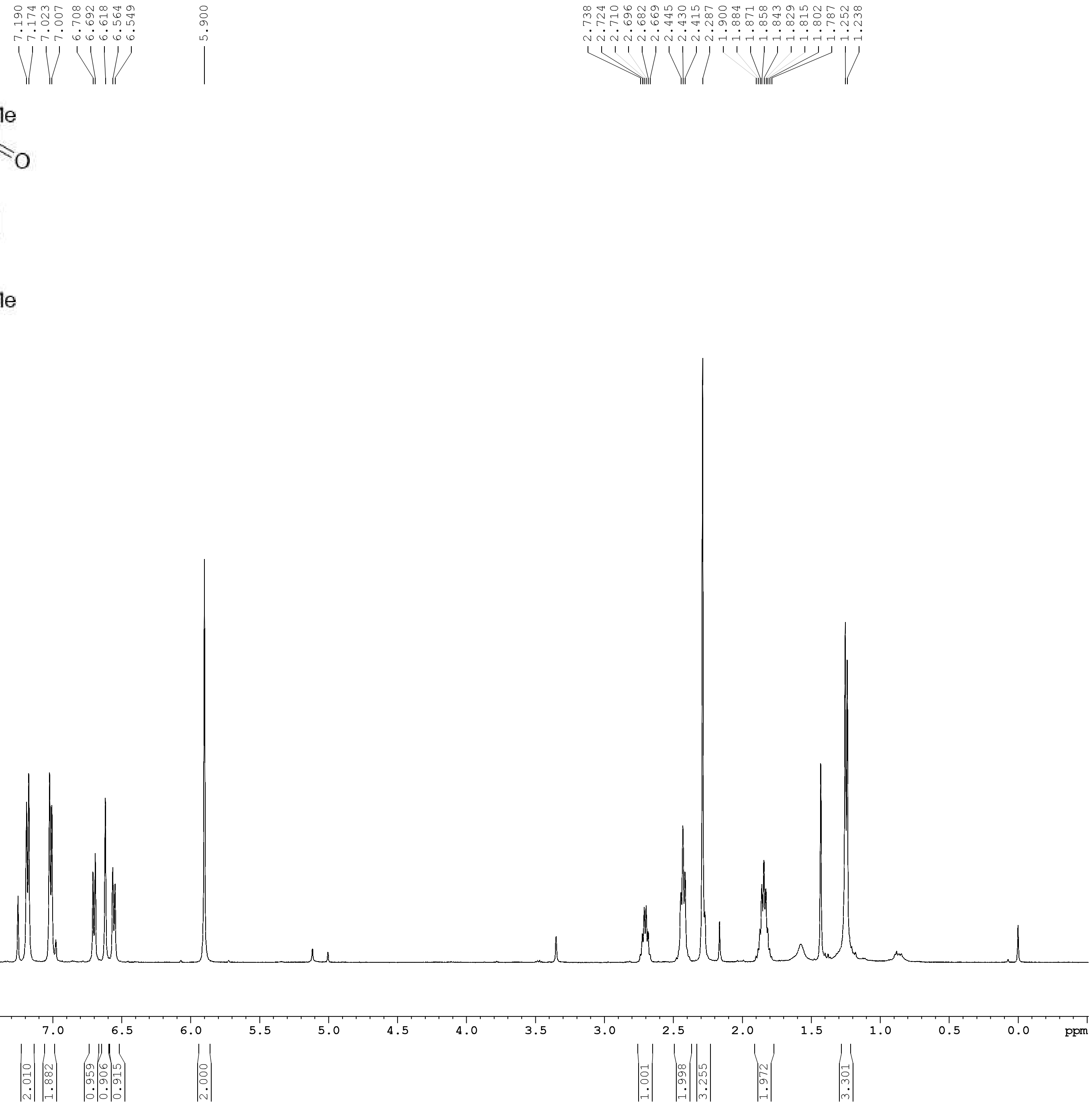

Current Data Parameters
NAME LCB-1-75-ge
EXPNO 1
PROCNO 1

F2 - Acquisition Parameters
Date_ 20240716
Time 9.20
INSTRUM gn500
PROBHD 5 mm broadband
PULPROG zg30
TD 81728
SOLVENT CDCl3T
NS 8
DS 2
SWH 8012.820 Hz
FIDRES 0.098043 Hz
AQ 5.0998273 sec
RG 181
DW 62.400 usec
DE 6.00 usec
TE 298.0 K
D1 0.10000000 sec
MCREST 0 sec
MCWRK 0.01500000 sec

======== CHANNEL f1 ========
NUC1 1H
P1 12.00 usec
PL1 -6.00 dB
SFO1 498.4534891 MHz

F2 - Processing parameters
SI 65536
SF 498.4500337 MHz
WDW EM
SSB 0
LB 0.30 Hz
GB 0
PC 1.00

13C spectrum with 1H decoupling

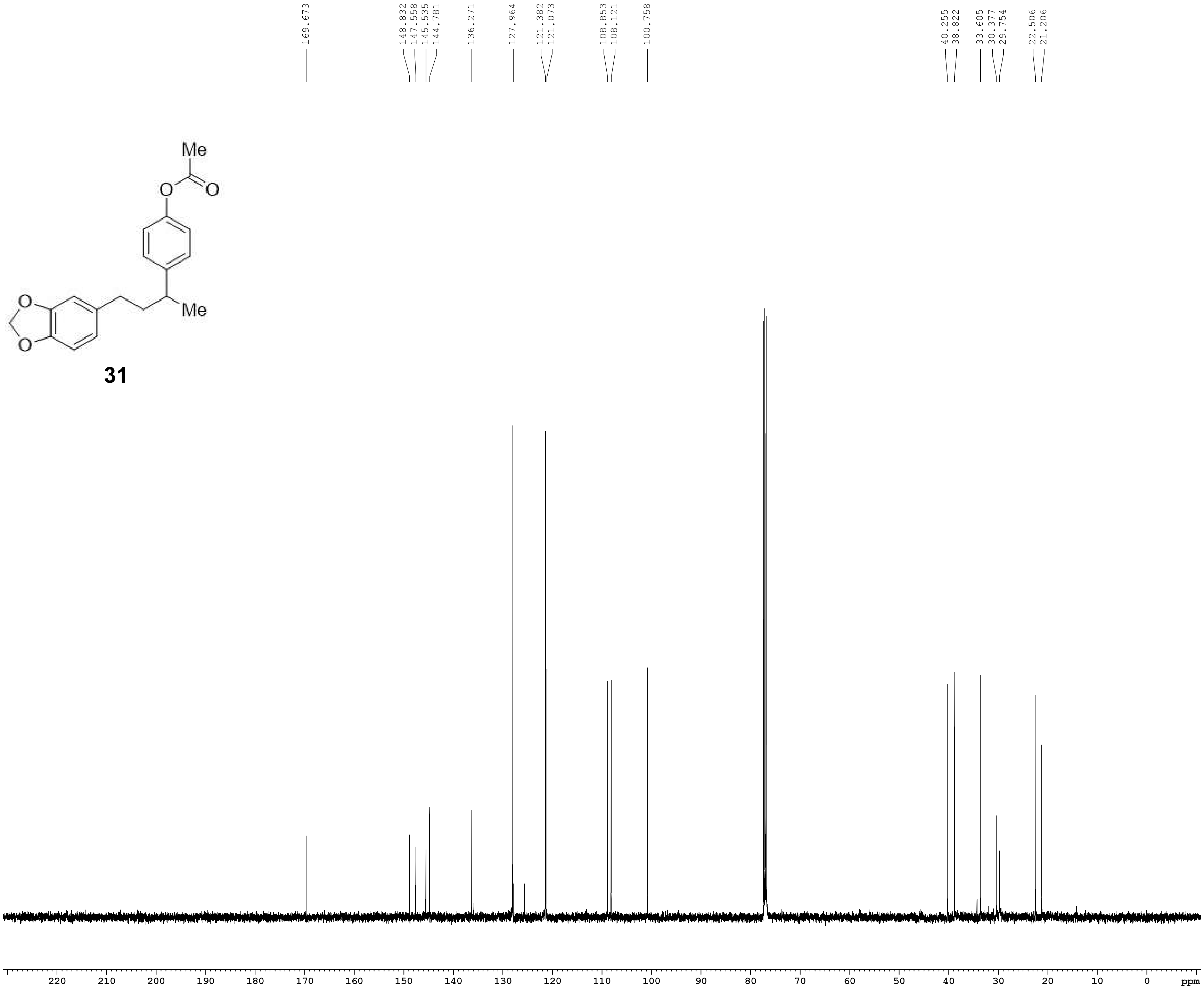

31

```
Current Data Parameters
NAME      LCB-1-75-500C
EXPNO                 1
PROCNO                1

F2 - Acquisition Parameters
Date_          20240711
Time               9.40
INSTRUM           gn500
PROBHD   5 mm broadband
PULPROG          zgdc30
TD                65536
SOLVENT          CDCl3T
NS                  833
DS                    4
SWH           30303.031 Hz
FIDRES         0.462388 Hz
AQ            1.0813440 sec
RG                 3251
DW               16.500 usec
DE                 6.00 usec
TE                298.0 K
D1           0.25000000 sec
d11          0.03000000 sec
MCREST                0 sec
MCWRK        0.01500000 sec

======== CHANNEL f1 ========
NUC1                13C
P1                14.20 usec
PL1               -6.00 dB
SFO1        125.3491398 MHz

======== CHANNEL f2 ========
CPDPRG[2        waltz16
NUC2                 1H
PCPD2            100.00 usec
PL2               -6.00 dB
PL12              12.30 dB
SFO2        498.4524922 MHz

F2 - Processing parameters
SI                65536
SF          125.3353520 MHz
WDW                  EM
SSB                   0
LB                 1.00 Hz
GB                    0
PC                 2.00
```

1H spectrum

32

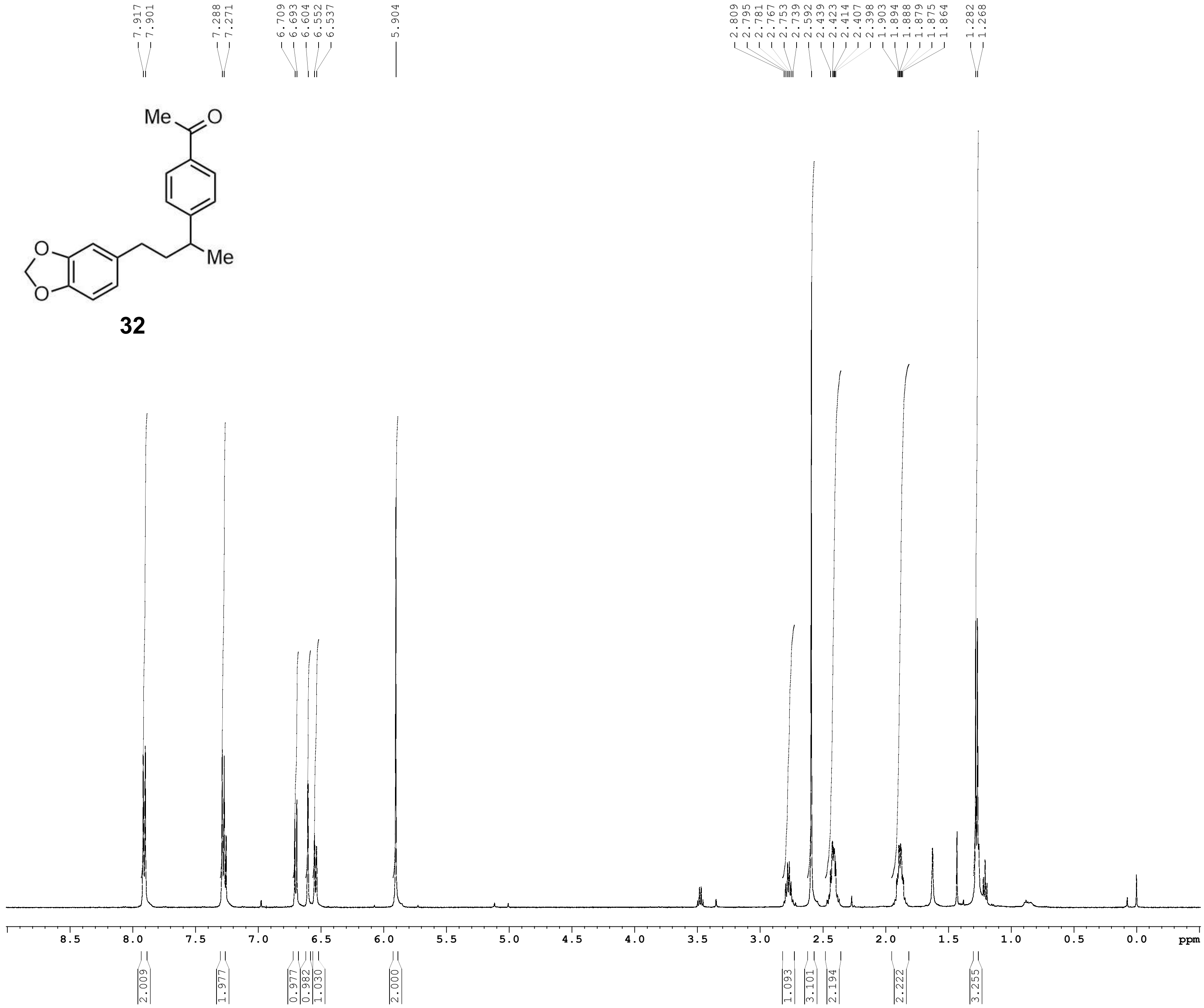

Current Data Parameters
NAME cdw-3-132-p
EXPNO 1
PROCNO 1

F2 - Acquisition Parameters
Date_ 20240626
Time 8.49
INSTRUM gn500
PROBHD 5 mm broadband
PULPROG zg30
TD 81728
SOLVENT CDCl3
NS 8
DS 2
SWH 8012.820 Hz
FIDRES 0.098043 Hz
AQ 5.0998273 sec
RG 574.7
DW 62.400 usec
DE 6.00 usec
TE 298.0 K
D1 0.10000000 sec
MCREST 0 sec
MCWRK 0.01500000 sec

======== CHANNEL f1 ========
NUC1 1H
P1 12.00 usec
PL1 -6.00 dB
SFO1 498.4534891 MHz

F2 - Processing parameters
SI 65536
SF 498.4500314 MHz
WDW no
SSB 0
LB 0 Hz
GB 0
PC 1.00

13C spectrum with 1H decoupling

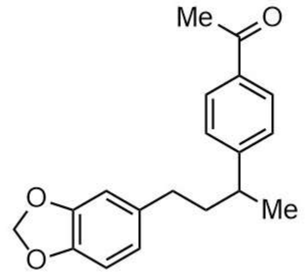

32

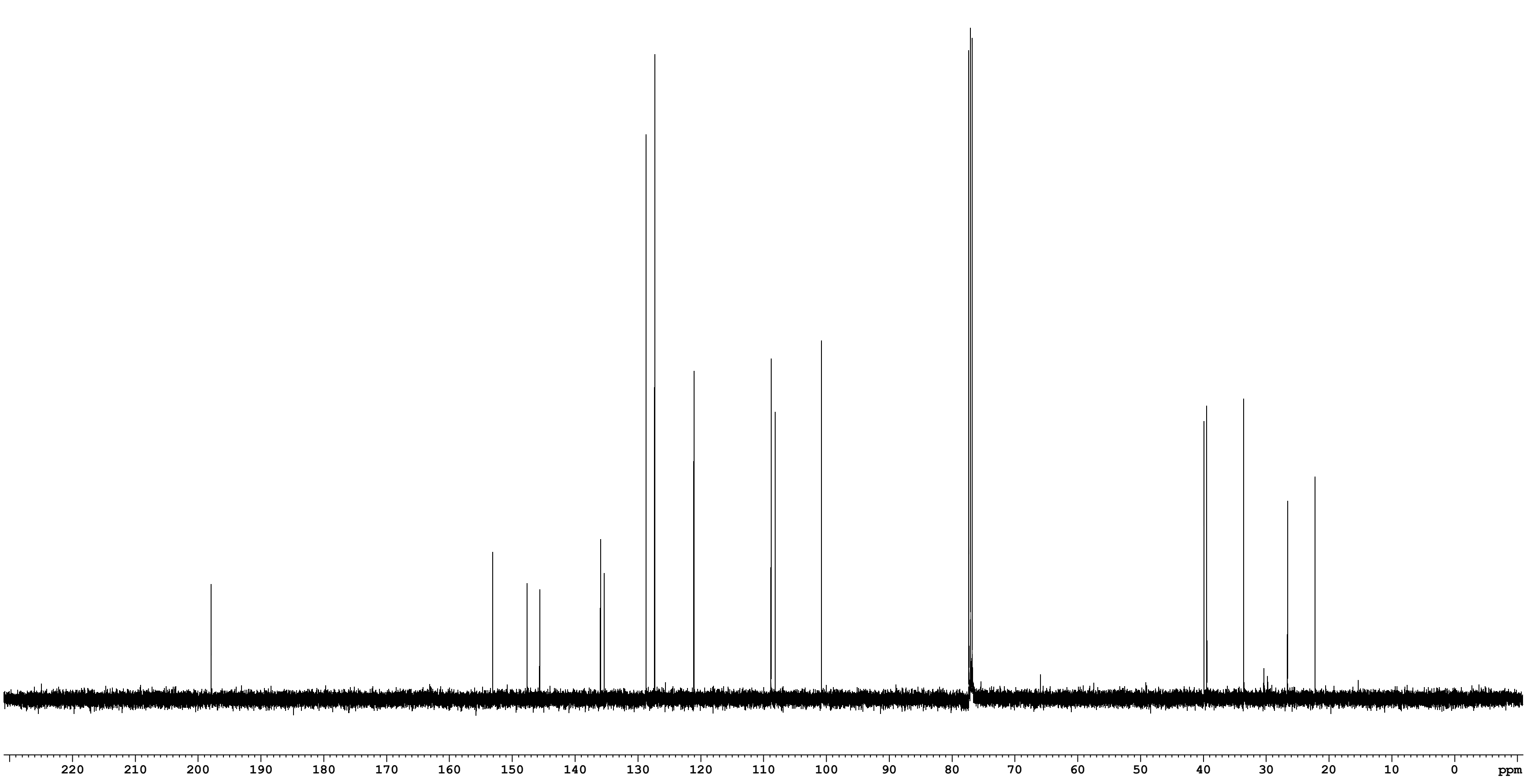


```
Current Data Parameters
NAME      cdw-3-132-c13
EXPNO                 1
PROCNO                1

F2 - Acquisition Parameters
Date_          20240626
Time               8.57
INSTRUM           gn500
PROBHD   5 mm broadband
PULPROG          zgdc30
TD                65536
SOLVENT           CDCl3
NS                  600
DS                    4
SWH           30303.031 Hz
FIDRES         0.462388 Hz
AQ            1.0813440 sec
RG               5792.6
DW               16.500 usec
DE                 6.00 usec
TE                298.0 K
D1           0.25000000 sec
d11          0.03000000 sec
MCREST                0 sec
MCWRK        0.01500000 sec

======== CHANNEL f1 ========
NUC1                13C
P1                14.20 usec
PL1               -6.00 dB
SFO1        125.3491398 MHz

======== CHANNEL f2 ========
CPDPRG[2        waltz16
NUC2                 1H
PCPD2            100.00 usec
PL2               -6.00 dB
PL12              12.30 dB
SFO2        498.4524922 MHz

F2 - Processing parameters
SI                65536
SF          125.3353520 MHz
WDW                  no
SSB                   0
LB                    0 Hz
GB                    0
PC                 2.00
```

1H spectrum

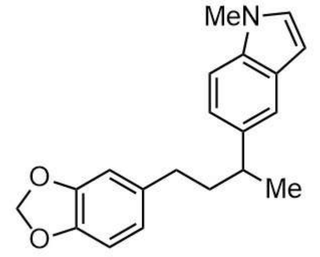

33

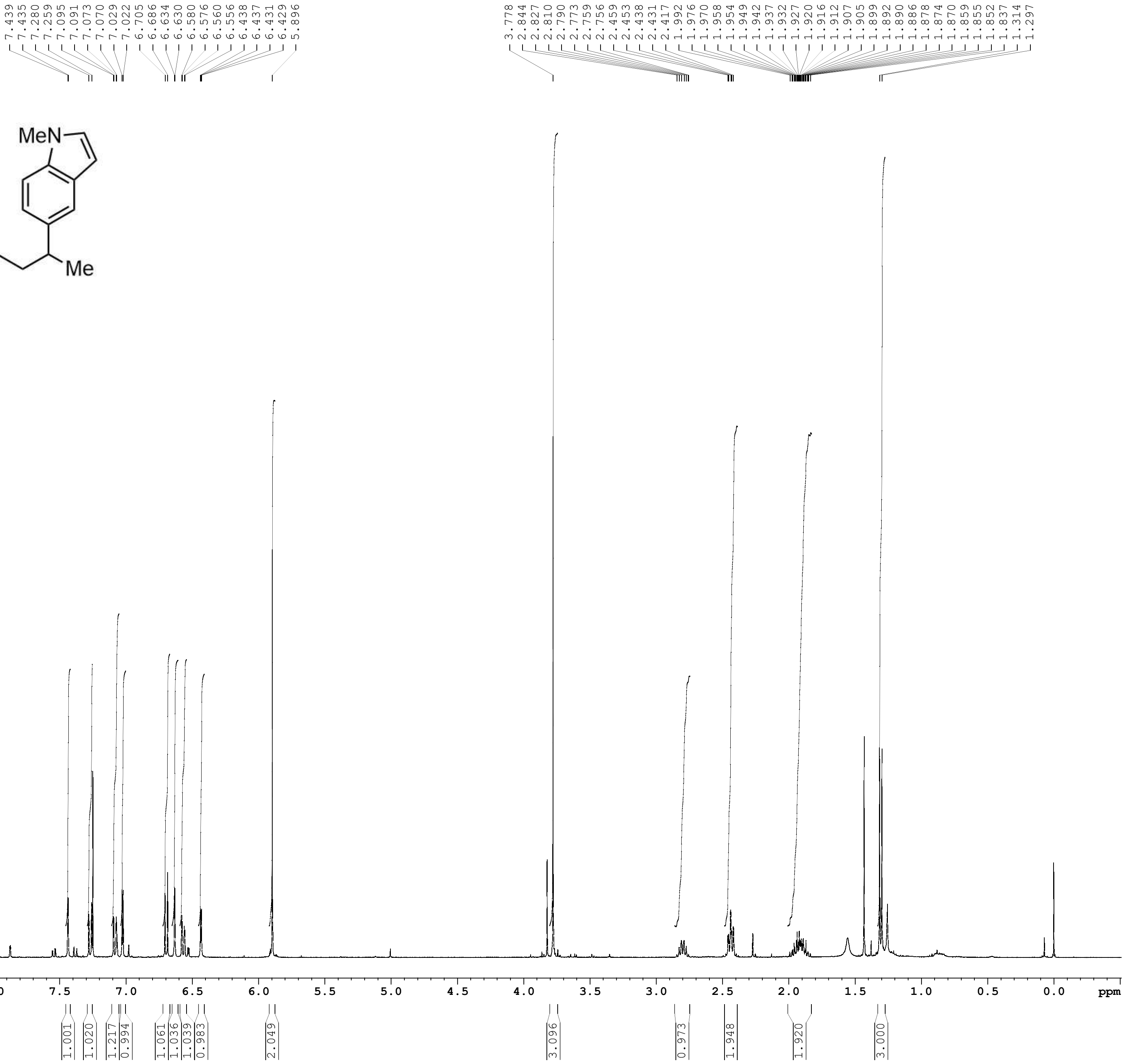

Current Data Parameters
NAME cdw-2-283-p
EXPNO 1
PROCNO 1

F2 - Acquisition Parameters
Date_ 20240229
Time 15.32 h
INSTRUM NEO 400
PROBHD Z163739_0122 (
PULPROG zg30
TD 65536
SOLVENT CDCl3
NS 8
DS 2
SWH 6250.000 Hz
FIDRES 0.190735 Hz
AQ 5.2428799 sec
RG 101
DW 80.000 usec
DE 8.64 usec
TE 298.3 K
D1 0.10000000 sec
TD0 1
SFO1 400.1328009 MHz
NUC1 1H
P0 2.67 usec
P1 8.00 usec
PLW1 23.01399994 W

F2 - Processing parameters
SI 65536
SF 400.1300258 MHz
WDW no
SSB 0
LB 0 Hz
GB 0
PC 1.00

13C spectrum with 1H decoupling

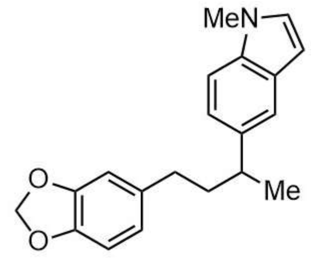

33

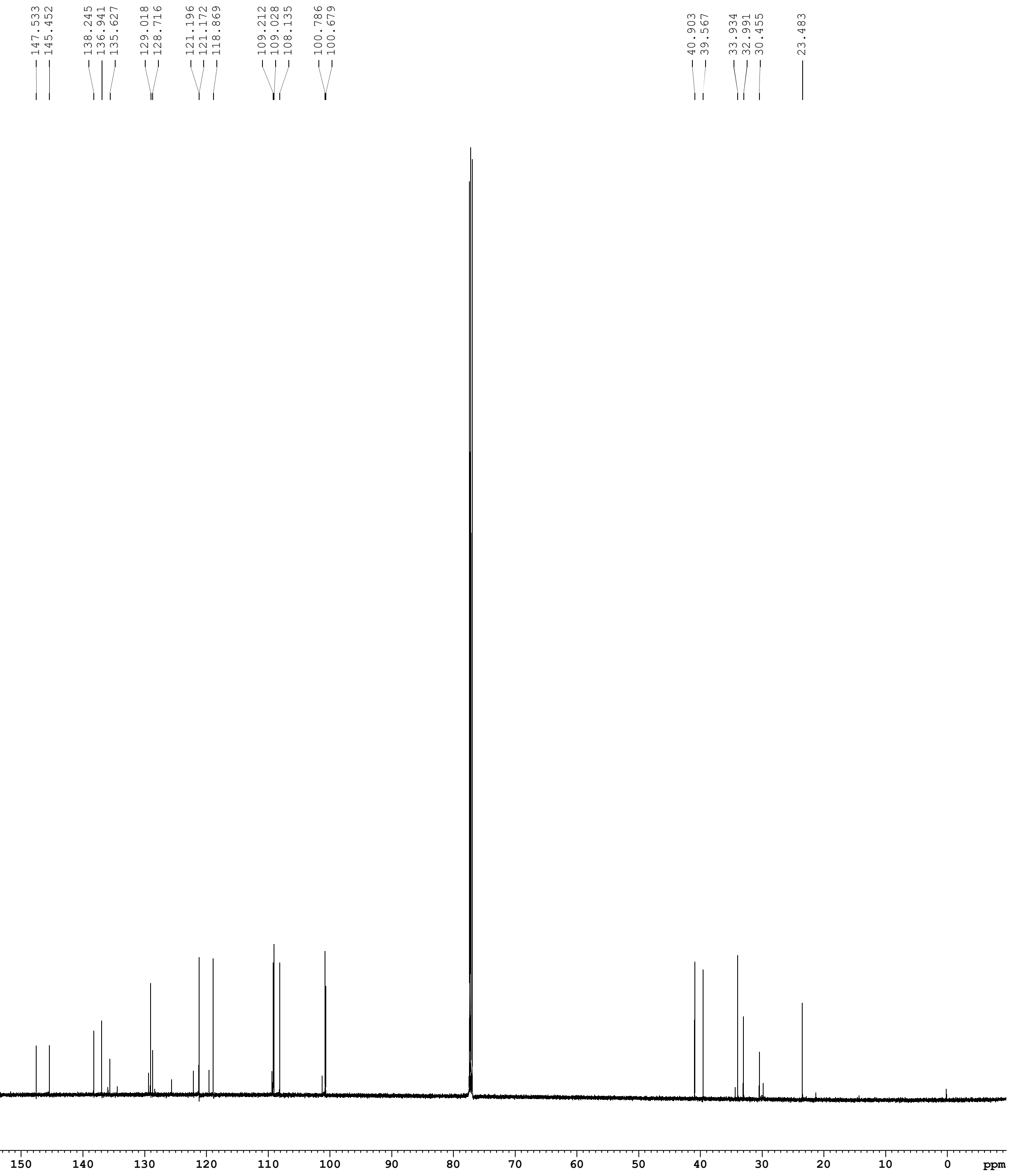


```
Current Data Parameters
NAME       cdw-2-283-c13
EXPNO                  1
PROCNO                 1

F2 - Acquisition Parameters
Date_           20240301
Time                7.54
INSTRUM            av600
PROBHD   5 mm CPBBO BB-
PULPROG           zgdc30
TD                 65536
SOLVENT            CDCl3
NS                   530
DS                     4
SWH           36231.883 Hz
FIDRES         0.552855 Hz
AQ            0.9043968 sec
RG                  2050
DW                13.800 usec
DE                 19.65 usec
TE                 298.0 K
D1            0.40000001 sec
D11           0.03000000 sec
TD0                    1

======== CHANNEL f1 ========
SFO1        150.9194080 MHz
NUC1                 13C
P1                 10.00 usec
PLW1         68.40000153 W

======== CHANNEL f2 ========
SFO2        600.1330010 MHz
NUC2                  1H
CPDPRG[2         waltz16
PCPD2              80.00 usec
PLW2         30.00000000 W
PLW12         0.39811000 W

F2 - Processing parameters
SI                 65536
SF          150.9027964 MHz
WDW                   no
SSB                    0
LB                     0 Hz
GB                     0
PC                  1.00
```

1H spectrum

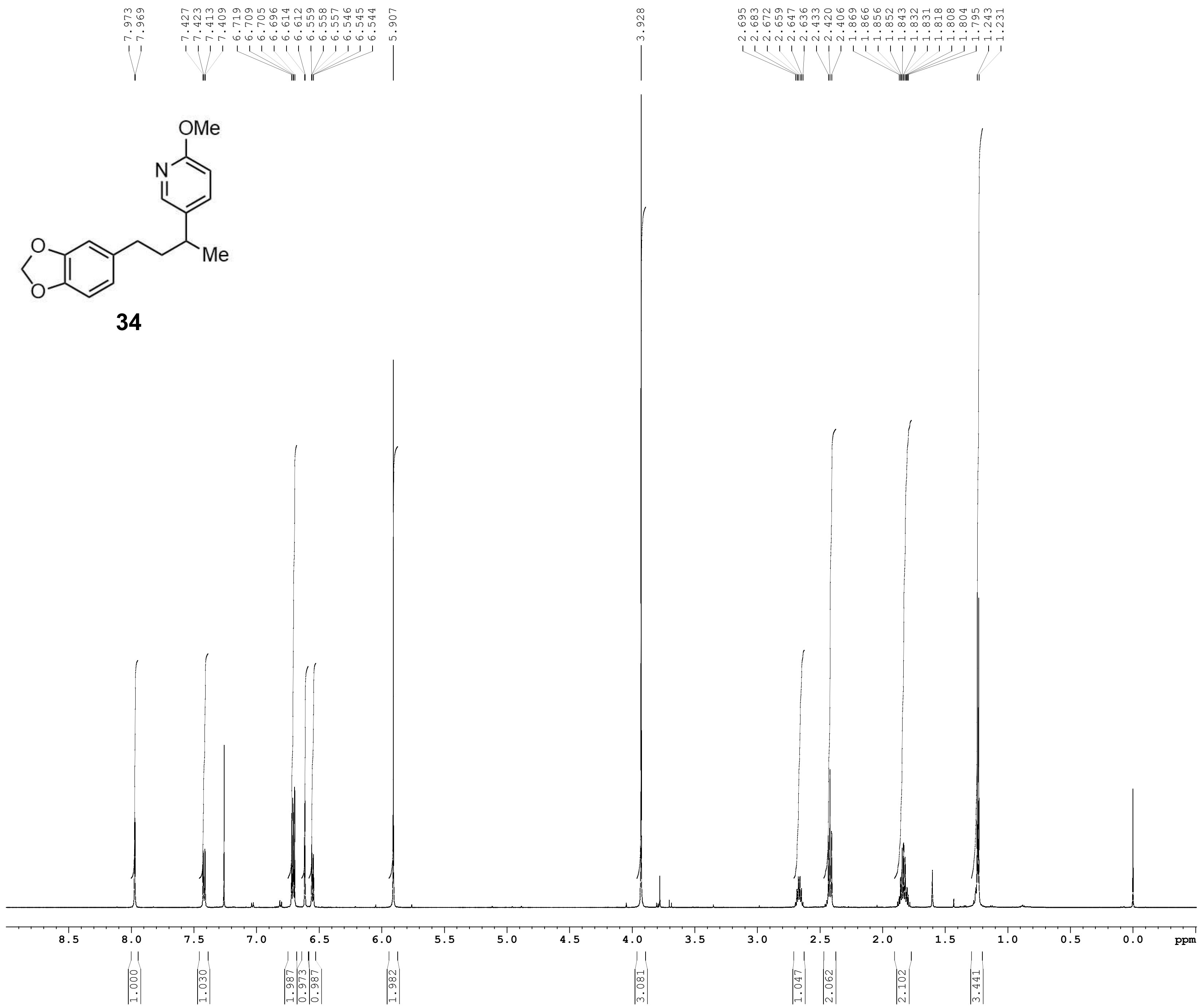

Current Data Parameters
NAME cdw3-250-p
EXPNO 1
PROCNO 1

F2 - Acquisition Parameters
Date_ 20241106
Time 7.53
INSTRUM av600
PROBHD 5 mm CPBBO BB-
PULPROG zg30
TD 98074
SOLVENT CDCl3
NS 8
DS 2
SWH 9615.385 Hz
FIDRES 0.098042 Hz
AQ 5.0998478 sec
RG 228
DW 52.000 usec
DE 53.12 usec
TE 298.0 K
D1 0.10000000 sec
TD0 1

======== CHANNEL f1 ========
SFO1 600.1342009 MHz
NUC1 1H
P1 10.00 usec
PLW1 30.00000000 W

F2 - Processing parameters
SI 65536
SF 600.1300348 MHz
WDW no
SSB 0
LB 0 Hz
GB 0
PC 1.00

13C spectrum with 1H decoupling

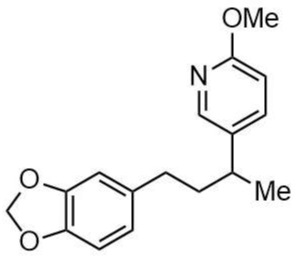

34

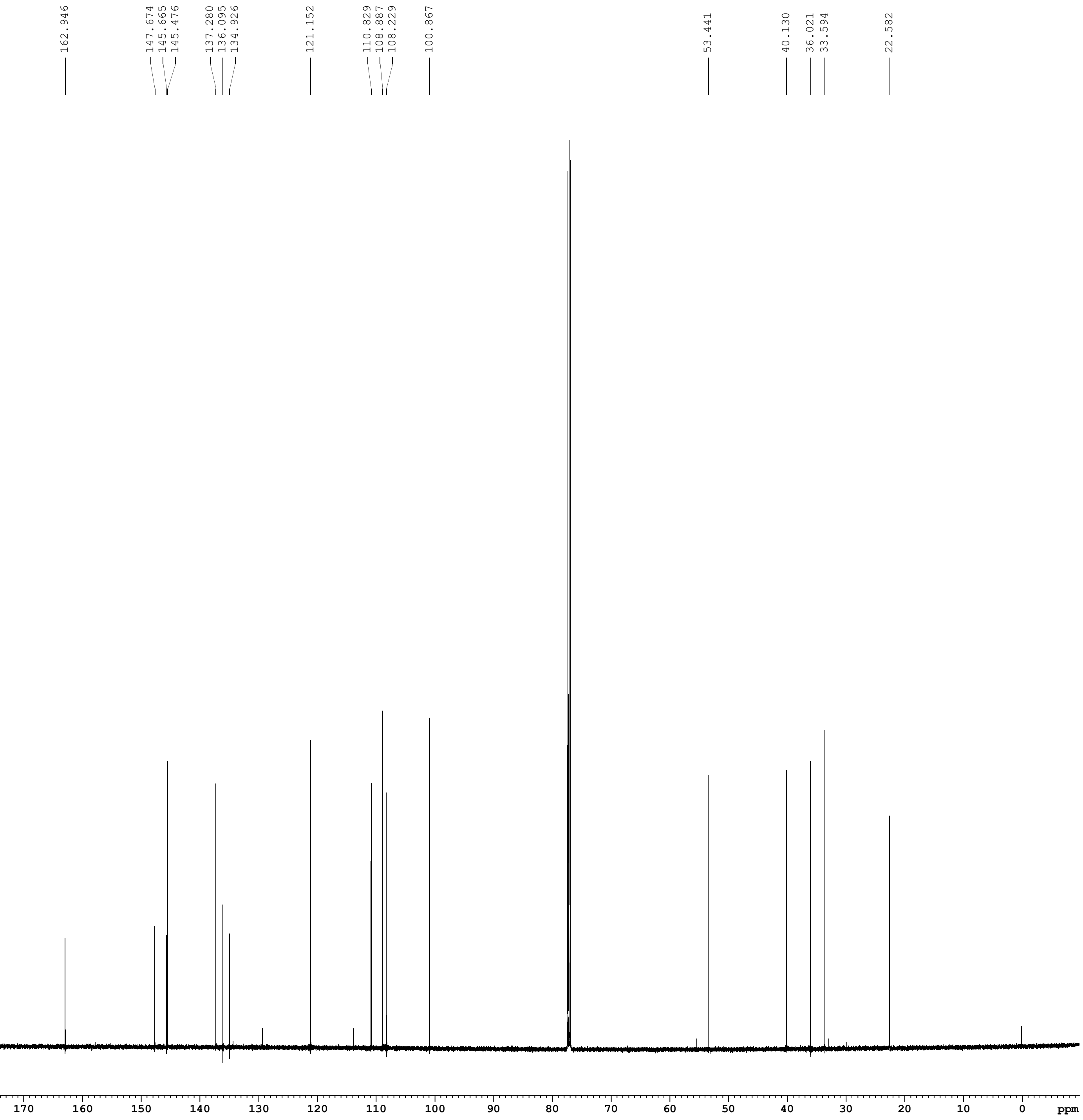


```
Current Data Parameters
NAME       cdw3-250-c13
EXPNO                 1
PROCNO                1

F2 - Acquisition Parameters
Date_          20241106
Time               8.04
INSTRUM           av600
PROBHD   5 mm CPBBO BB-
PULPROG          zgdc30
TD                65536
SOLVENT           CDCl3
NS                  560
DS                    4
SWH           36231.883 Hz
FIDRES         0.552855 Hz
AQ            0.9043968 sec
RG                 2050
DW               13.800 usec
DE                19.65 usec
TE                298.0 K
D1           0.40000001 sec
D11          0.03000000 sec
TD0                   1

======== CHANNEL f1 ========
SFO1        150.9194080 MHz
NUC1                13C
P1                10.00 usec
PLW1        68.40000153 W

======== CHANNEL f2 ========
SFO2        600.1330010 MHz
NUC2                 1H
CPDPRG[2         waltz16
PCPD2             80.00 usec
PLW2        30.00000000 W
PLW12        0.39811000 W

F2 - Processing parameters
SI                65536
SF          150.9027957 MHz
WDW                  no
SSB                   0
LB                    0 Hz
GB                    0
PC                 1.00
```

1H spectrum

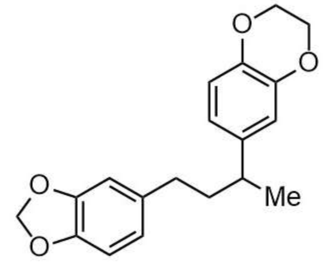

35

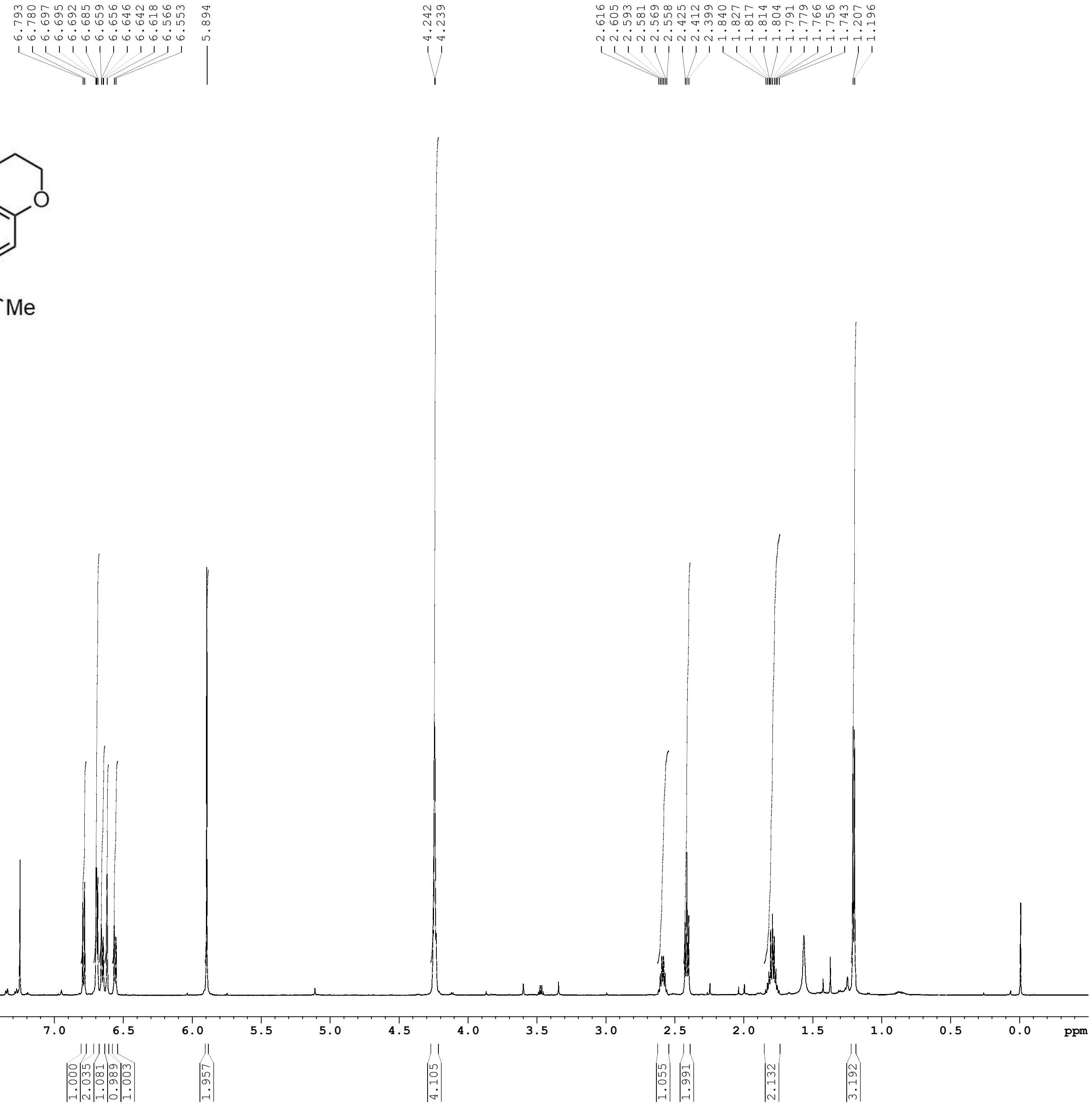

Current Data Parameters
NAME cdw-3-107-ge-pure
EXPNO 1
PROCNO 1

F2 - Acquisition Parameters
Date_ 20240725
Time 9.08
INSTRUM av600
PROBHD 5 mm CPBBO BB-
PULPROG zg30
TD 98074
SOLVENT CDCl3
NS 8
DS 2
SWH 9615.385 Hz
FIDRES 0.098042 Hz
AQ 5.0998478 sec
RG 181
DW 52.000 usec
DE 53.12 usec
TE 298.0 K
D1 0.10000000 sec
TD0 1

======== CHANNEL f1 ========
SFO1 600.1342009 MHz
NUC1 1H
P1 10.00 usec
PLW1 30.00000000 W

F2 - Processing parameters
SI 65536
SF 600.1300411 MHz
WDW no
SSB 0
LB 0 Hz
GB 0
PC 1.00

13C spectrum with 1H decoupling

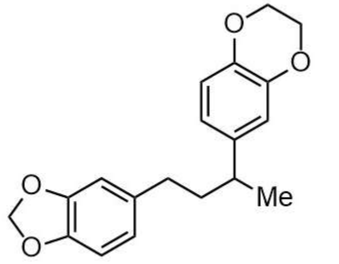

35

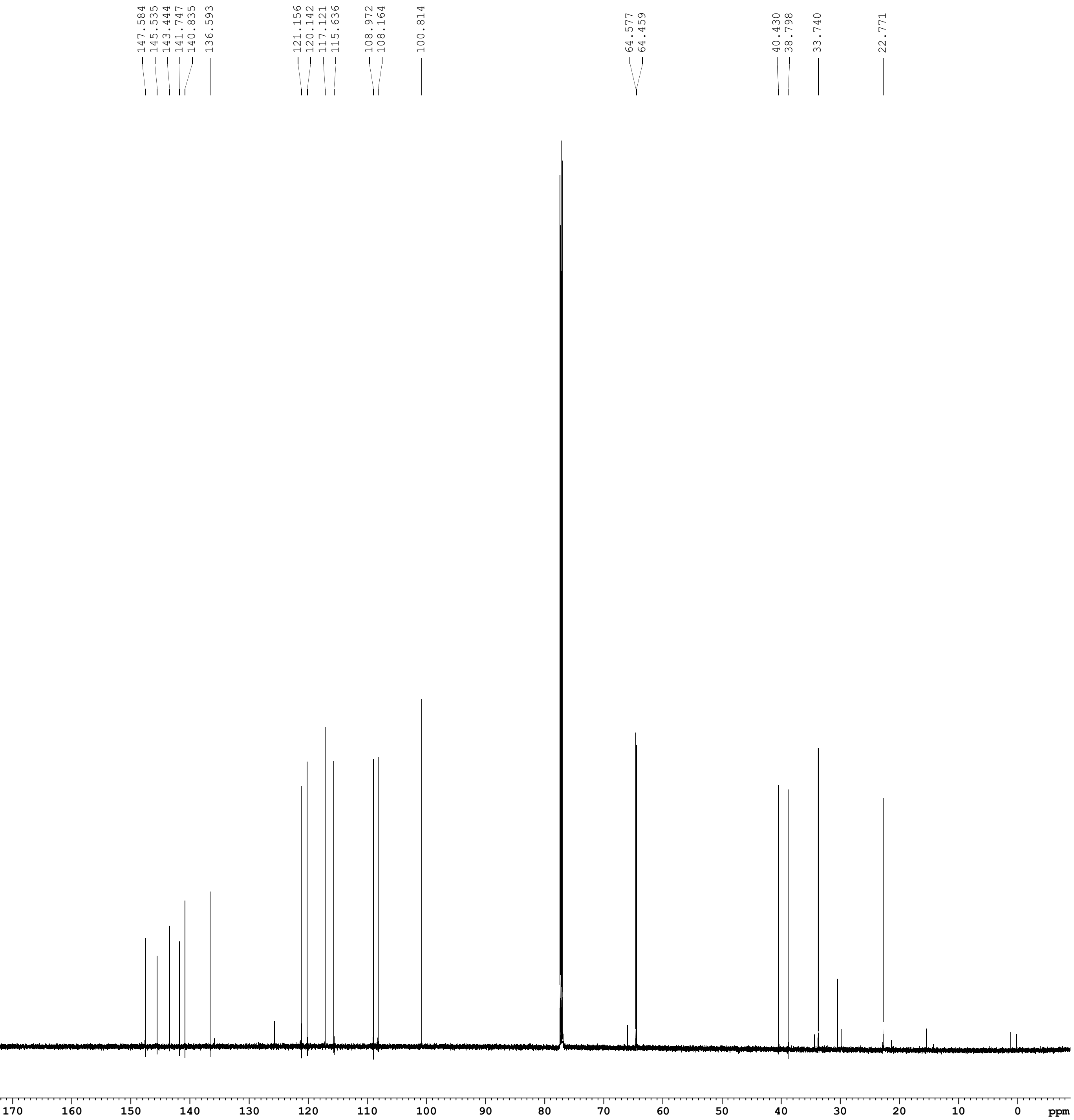


```
Current Data Parameters
NAME        cdw-3-107-c13
EXPNO                   1
PROCNO                  1

F2 - Acquisition Parameters
Date_            20240612
Time                 8.12
INSTRUM             av600
PROBHD   5 mm CPBBO BB-
PULPROG            zgdc30
TD                  65536
SOLVENT             CDCl3
NS                    300
DS                      4
SWH             36231.883 Hz
FIDRES           0.552855 Hz
AQ              0.9043968 sec
RG                   2050
DW                 13.800 usec
DE                  19.65 usec
TE                  298.1 K
D1             0.40000001 sec
D11            0.03000000 sec
TD0                     1

======== CHANNEL f1 ========
SFO1          150.9194080 MHz
NUC1                  13C
P1                  10.00 usec
PLW1          68.40000153 W

======== CHANNEL f2 ========
SFO2          600.1330010 MHz
NUC2                   1H
CPDPRG[2           waltz16
PCPD2               80.00 usec
PLW2          30.00000000 W
PLW12          0.39811000 W

F2 - Processing parameters
SI                  65536
SF            150.9027959 MHz
WDW                    no
SSB                     0
LB                      0 Hz
GB                      0
PC                   1.00
```

1H spectrum

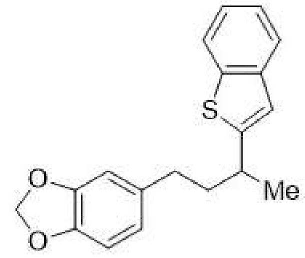

36

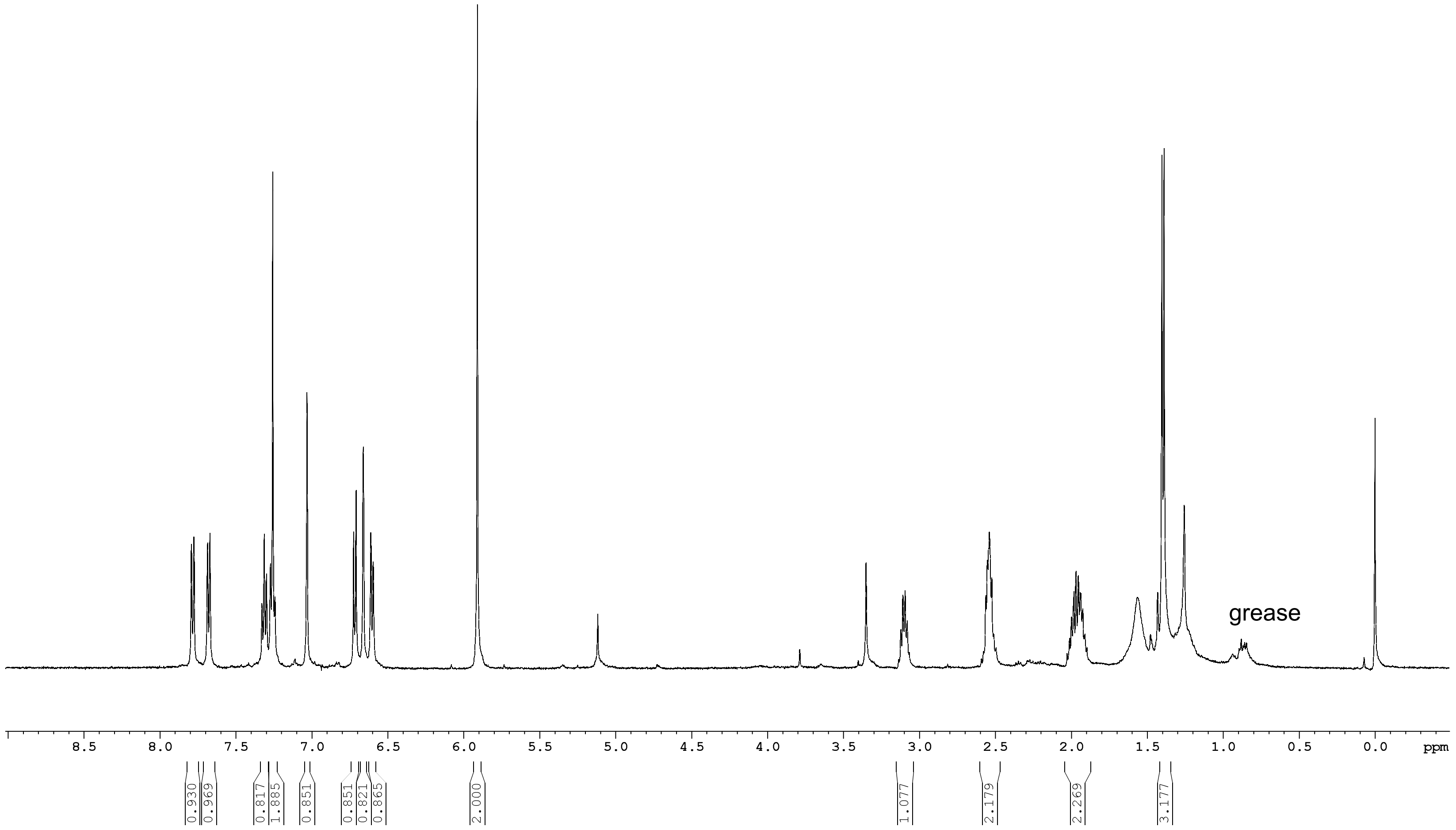


```
Current Data Parameters
NAME      LCB-1-140-ge4
EXPNO                 1
PROCNO                1

F2 - Acquisition Parameters
Date_          20241008
Time               9.52
INSTRUM           gn500
PROBHD   5 mm broadband
PULPROG            zg30
TD                48074
SOLVENT          CDCl3T
NS                    8
DS                    2
SWH            8012.820 Hz
FIDRES         0.166677 Hz
AQ            2.9998176 sec
RG               1149.4
DW               62.400 usec
DE                 6.00 usec
TE                298.0 K
D1           0.10000000 sec
MCREST                0 sec
MCWRK        0.01500000 sec

======== CHANNEL f1 ========
NUC1                 1H
P1                12.00 usec
PL1               -6.00 dB
SFO1        498.4534891 MHz

F2 - Processing parameters
SI                65536
SF          498.4500317 MHz
WDW                  EM
SSB                   0
LB                 0.30 Hz
GB                    0
PC                 1.00
```

13C spectrum with 1H decoupling

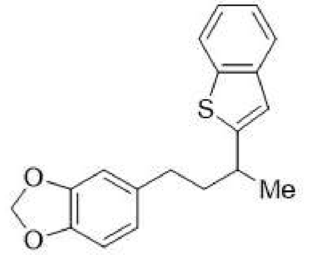

36

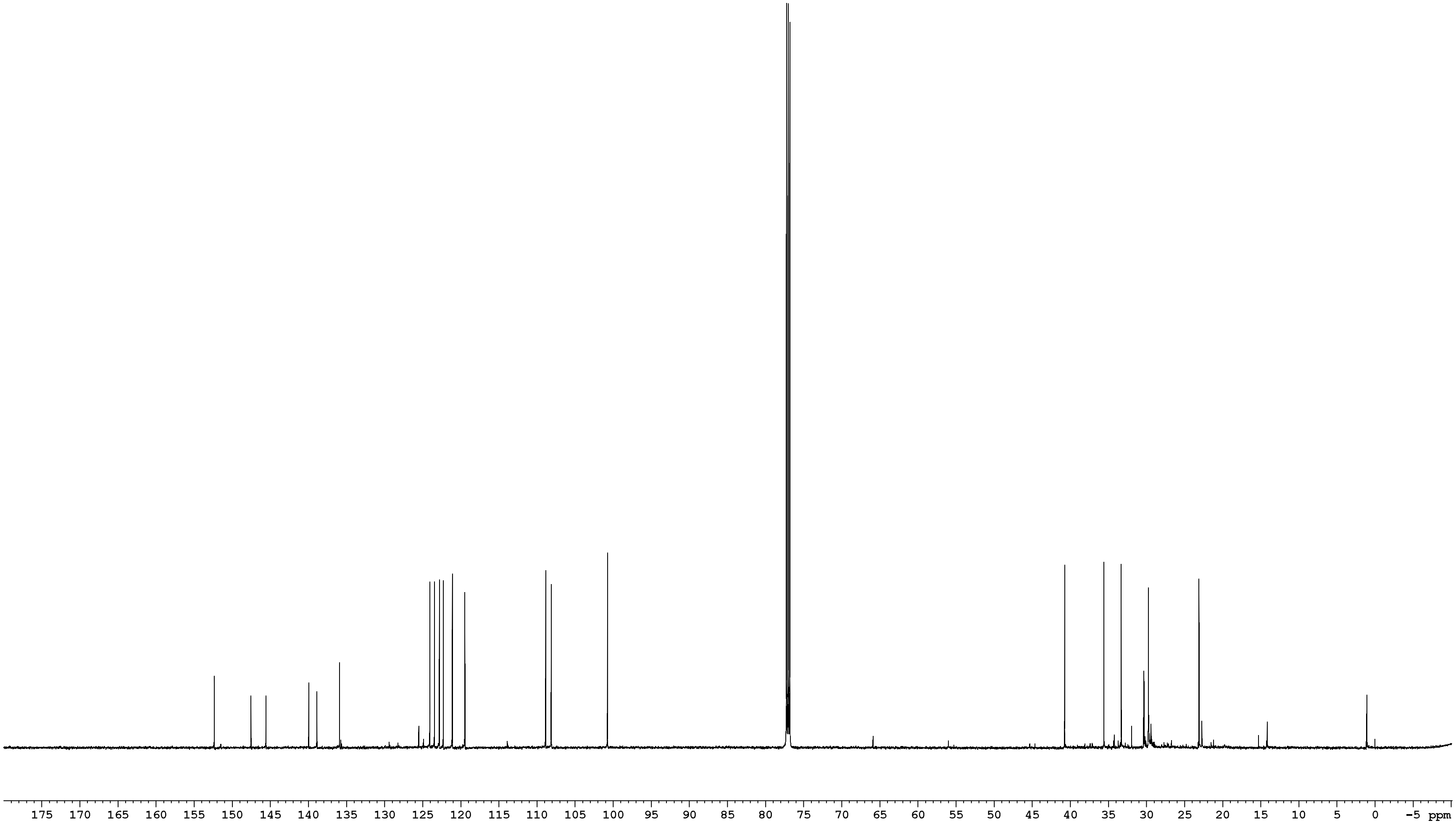


```
Current Data Parameters
NAME     LCB-1-140-600C-2
EXPNO                   1
PROCNO                  1

F2 - Acquisition Parameters
Date_            20240925
Time                 9.47
INSTRUM             av600
PROBHD   5 mm CPBBO BB-
PULPROG           zgdc30
TD                 65536
SOLVENT           CDCl3T
NS                   721
DS                     4
SWH            36231.883 Hz
FIDRES          0.552855 Hz
AQ             0.9043968 sec
RG                  2050
DW                13.800 usec
DE                 19.65 usec
TE                 298.1 K
D1            0.40000001 sec
D11           0.03000000 sec
TD0                    1

======== CHANNEL f1 ========
SFO1         150.9194080 MHz
NUC1                 13C
P1                 10.00 usec
PLW1         68.40000153 W

======== CHANNEL f2 ========
SFO2         600.1330010 MHz
NUC2                  1H
CPDPRG[2         waltz16
PCPD2              80.00 usec
PLW2         30.00000000 W
PLW12         0.39811000 W

F2 - Processing parameters
SI                 65536
SF           150.9028173 MHz
WDW                   EM
SSB                    0
LB                  1.00 Hz
GB                     0
PC                  1.00
```

1H spectrum

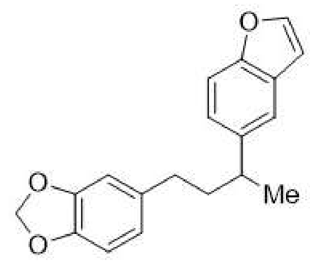

37

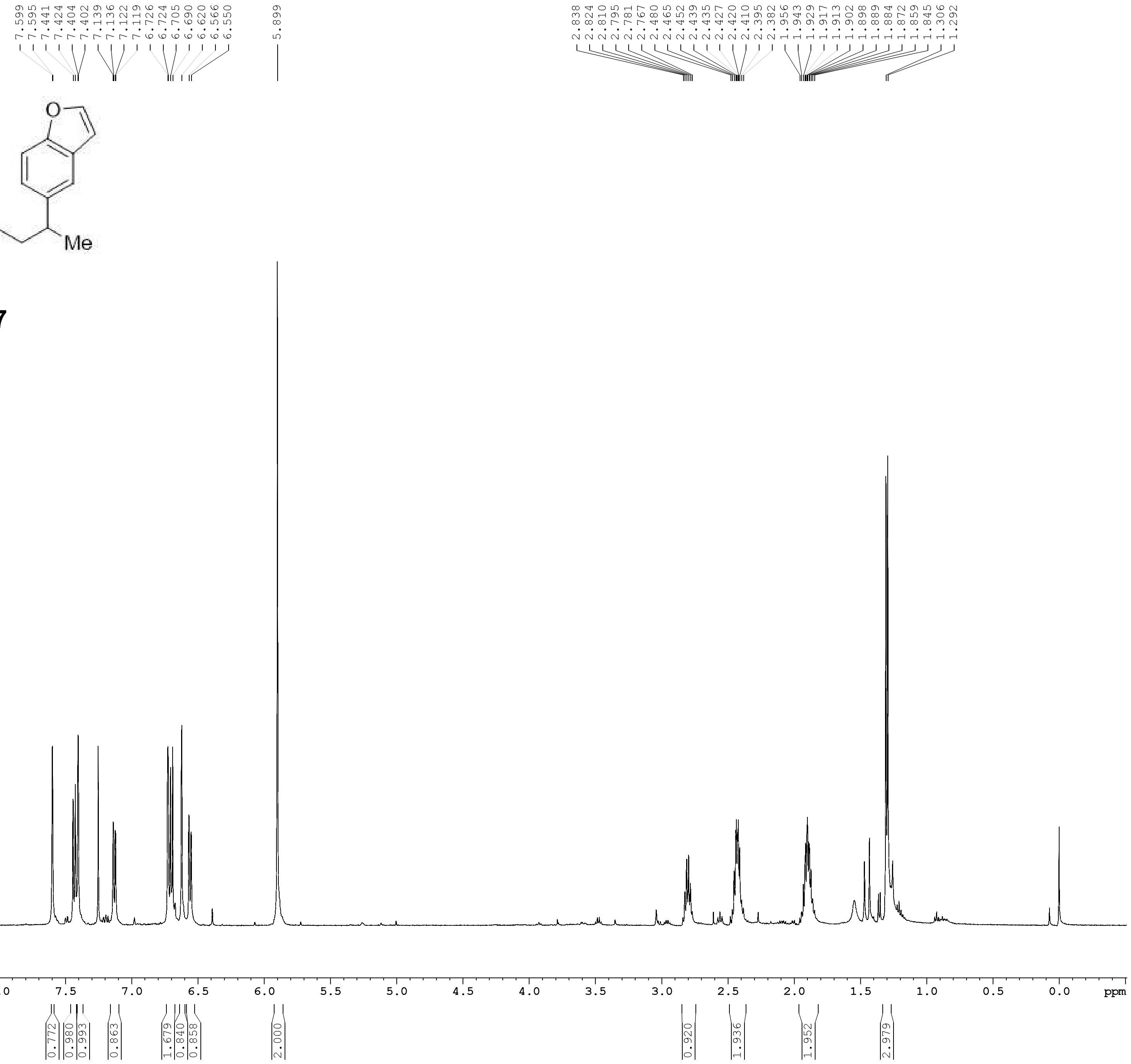


```
Current Data Parameters
NAME       LCB-1-138-ge
EXPNO                 1
PROCNO                1

F2 - Acquisition Parameters
Date_          20240925
Time               9.44
INSTRUM           gn500
PROBHD   5 mm broadband
PULPROG            zg30
TD                81728
SOLVENT          CDCl3T
NS                    8
DS                    2
SWH            8012.820 Hz
FIDRES         0.098043 Hz
AQ            5.0998273 sec
RG                724.1
DW               62.400 usec
DE                 6.00 usec
TE                298.0 K
D1           0.10000000 sec
MCREST                0 sec
MCWRK        0.01500000 sec

======== CHANNEL f1 ========
NUC1                 1H
P1                12.00 usec
PL1               -6.00 dB
SFO1        498.4534891 MHz

F2 - Processing parameters
SI                65536
SF          498.4500341 MHz
WDW                  EM
SSB                   0
LB                 0.30 Hz
GB                    0
PC                 1.00
```

13C spectrum with 1H decoupling

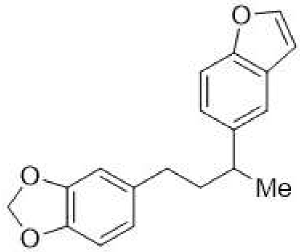

37

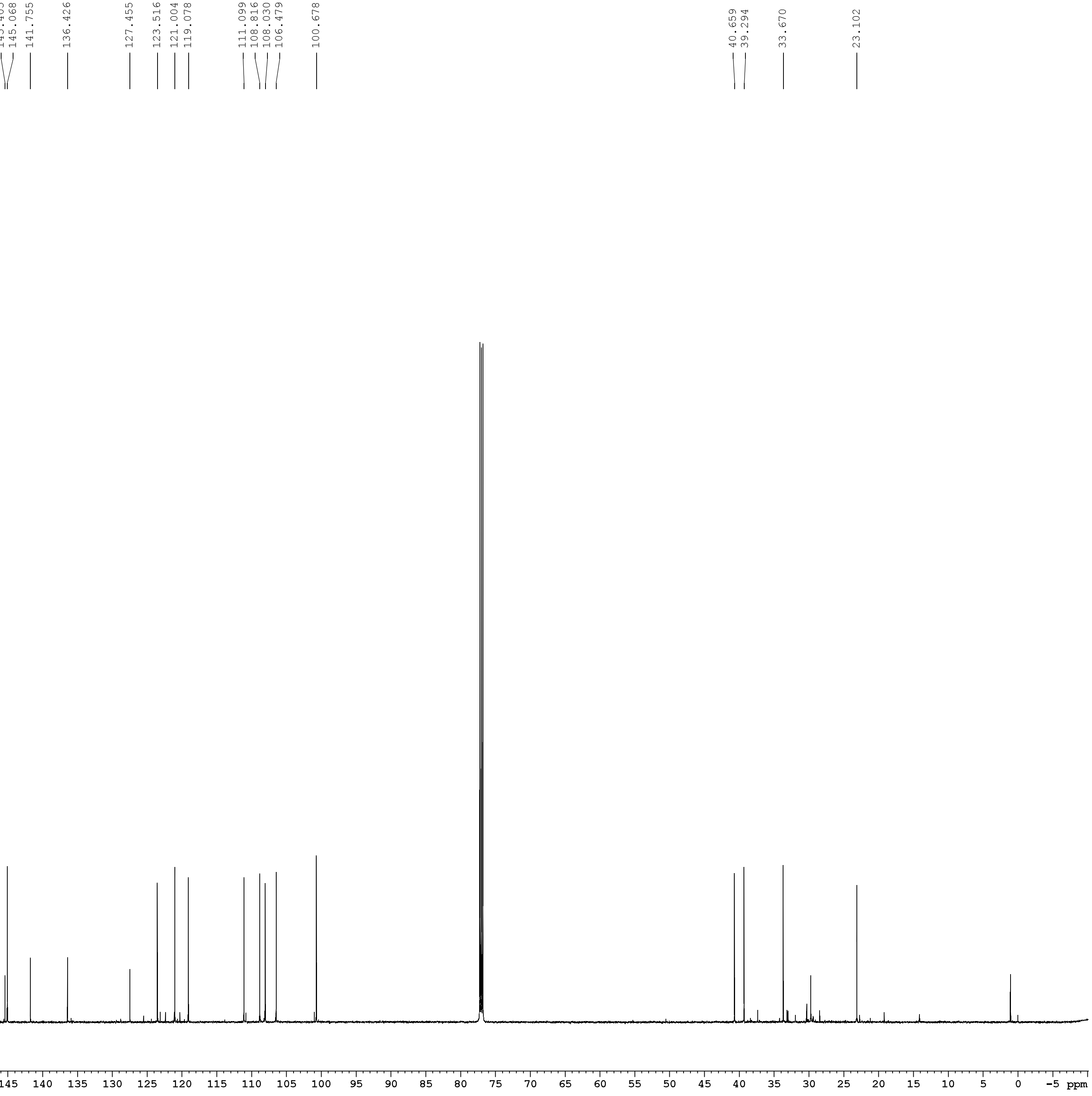


```
Current Data Parameters
NAME     LCB-1-138-600C-3
EXPNO                   1
PROCNO                  1

F2 - Acquisition Parameters
Date_          20240924
Time              13.50
INSTRUM           av600
PROBHD   5 mm CPBBO BB-
PULPROG          zgdc30
TD                65536
SOLVENT          CDCl3T
NS                  619
DS                    4
SWH           36231.883 Hz
FIDRES         0.552855 Hz
AQ            0.9043968 sec
RG                 2050
DW               13.800 usec
DE                19.65 usec
TE                298.1 K
D1           0.40000001 sec
D11          0.03000000 sec
TD0                   1

======== CHANNEL f1 ========
SFO1        150.9194080 MHz
NUC1                13C
P1                10.00 usec
PLW1        68.40000153 W

======== CHANNEL f2 ========
SFO2        600.1330010 MHz
NUC2                 1H
CPDPRG[2          waltz16
PCPD2             80.00 usec
PLW2        30.00000000 W
PLW12        0.39811000 W

F2 - Processing parameters
SI                65536
SF          150.9028185 MHz
WDW                  EM
SSB                   0
LB                 1.00 Hz
GB                    0
PC                 1.00
```

1H spectrum

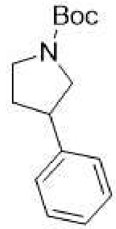

38

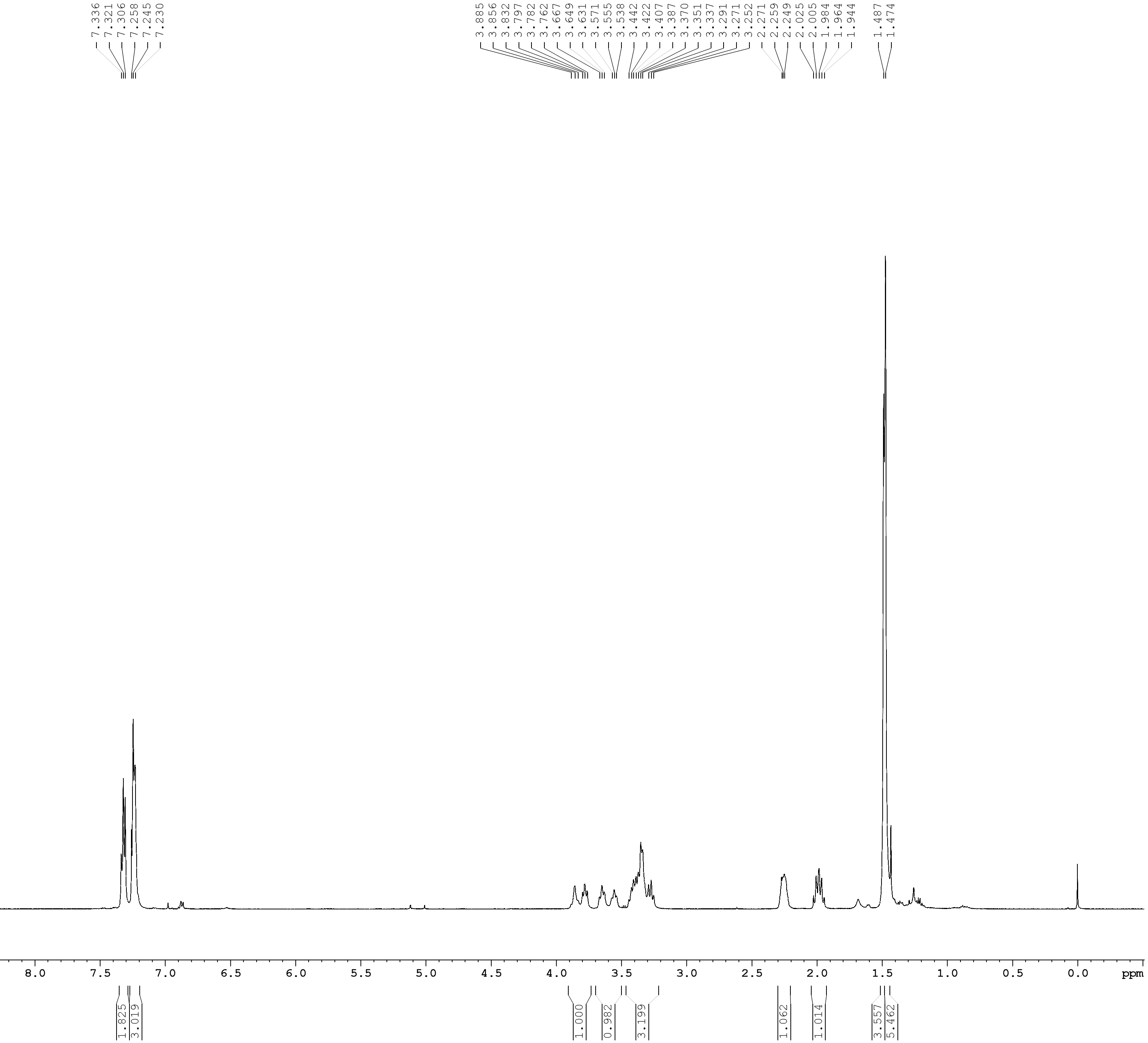


```
Current Data Parameters
NAME       LCB-1-148-ge
EXPNO                 1
PROCNO                1

F2 - Acquisition Parameters
Date_          20241009
Time              14.14
INSTRUM           gn500
PROBHD   5 mm broadband
PULPROG            zg30
TD                48074
SOLVENT          CDCl3T
NS                    8
DS                    2
SWH            8012.820 Hz
FIDRES         0.166677 Hz
AQ            2.9998176 sec
RG                161.3
DW               62.400 usec
DE                 6.00 usec
TE                298.0 K
D1           0.10000000 sec
MCREST                0 sec
MCWRK        0.01500000 sec

======== CHANNEL f1 ========
NUC1                 1H
P1                12.00 usec
PL1               -6.00 dB
SFO1        498.4534891 MHz

F2 - Processing parameters
SI                65536
SF          498.4500309 MHz
WDW                  EM
SSB                   0
LB                 0.30 Hz
GB                    0
PC                 1.00
```

1H spectrum

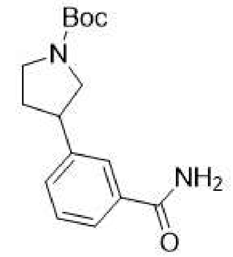

39

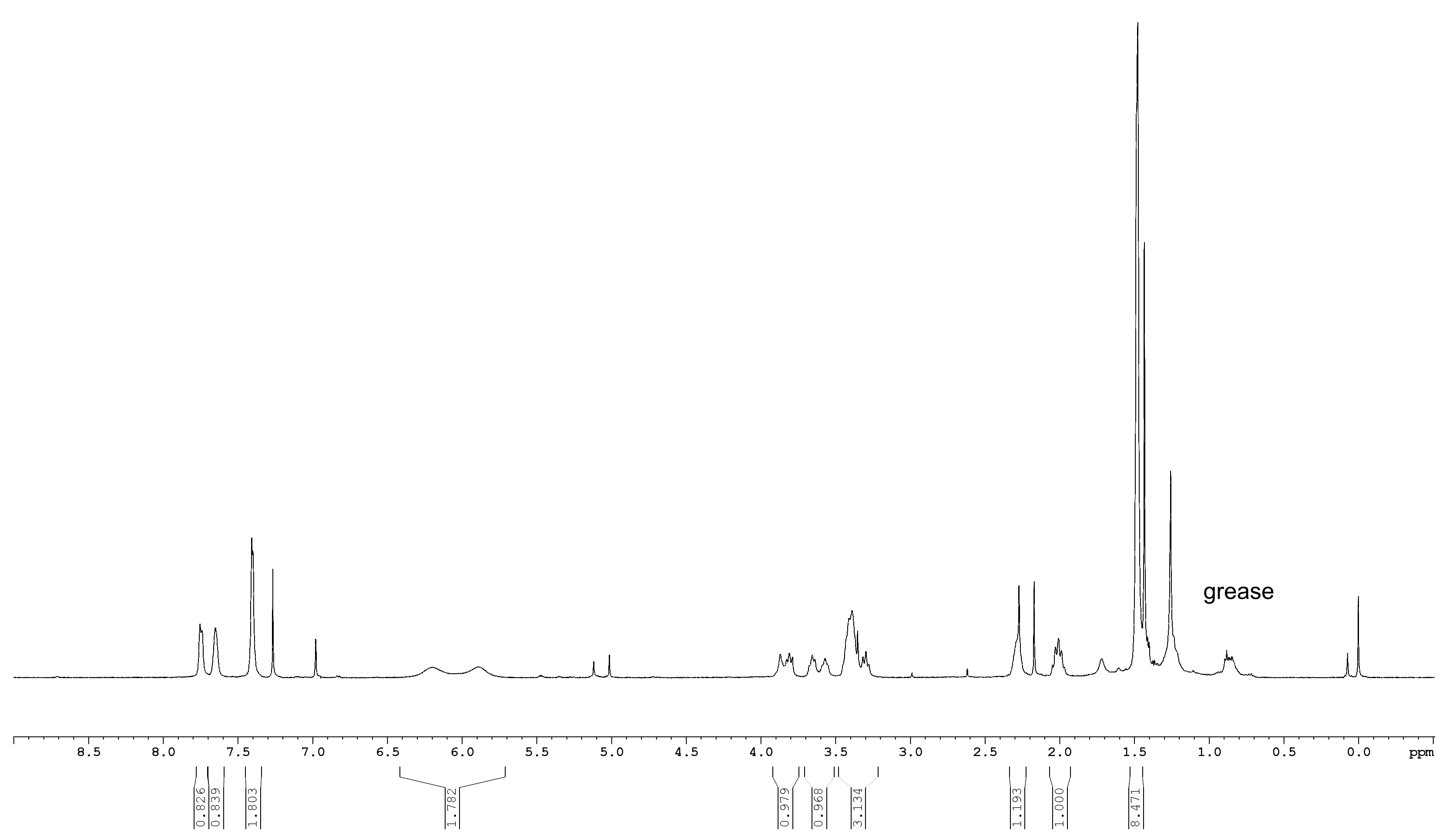


```
Current Data Parameters
NAME      LCB-1-149-dry
EXPNO                 1
PROCNO                1

F2 - Acquisition Parameters
Date_          20240919
Time              12.06
INSTRUM           gn500
PROBHD   5 mm broadband
PULPROG            zg30
TD                48074
SOLVENT          CDCl3T
NS                    8
DS                    2
SWH            8012.820 Hz
FIDRES         0.166677 Hz
AQ            2.9998176 sec
RG                645.1
DW               62.400 usec
DE                 6.00 usec
TE                298.0 K
D1           0.10000000 sec
MCREST                0 sec
MCWRK        0.01500000 sec

======== CHANNEL f1 ========
NUC1                 1H
P1                12.00 usec
PL1               -6.00 dB
SFO1        498.4534891 MHz

F2 - Processing parameters
SI                65536
SF          498.4500273 MHz
WDW                  EM
SSB                   0
LB                 0.30 Hz
GB                    0
PC                 1.00
```

13C spectrum with 1H decoupling

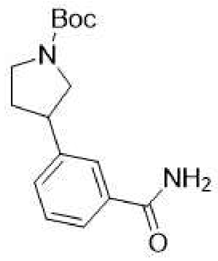

39

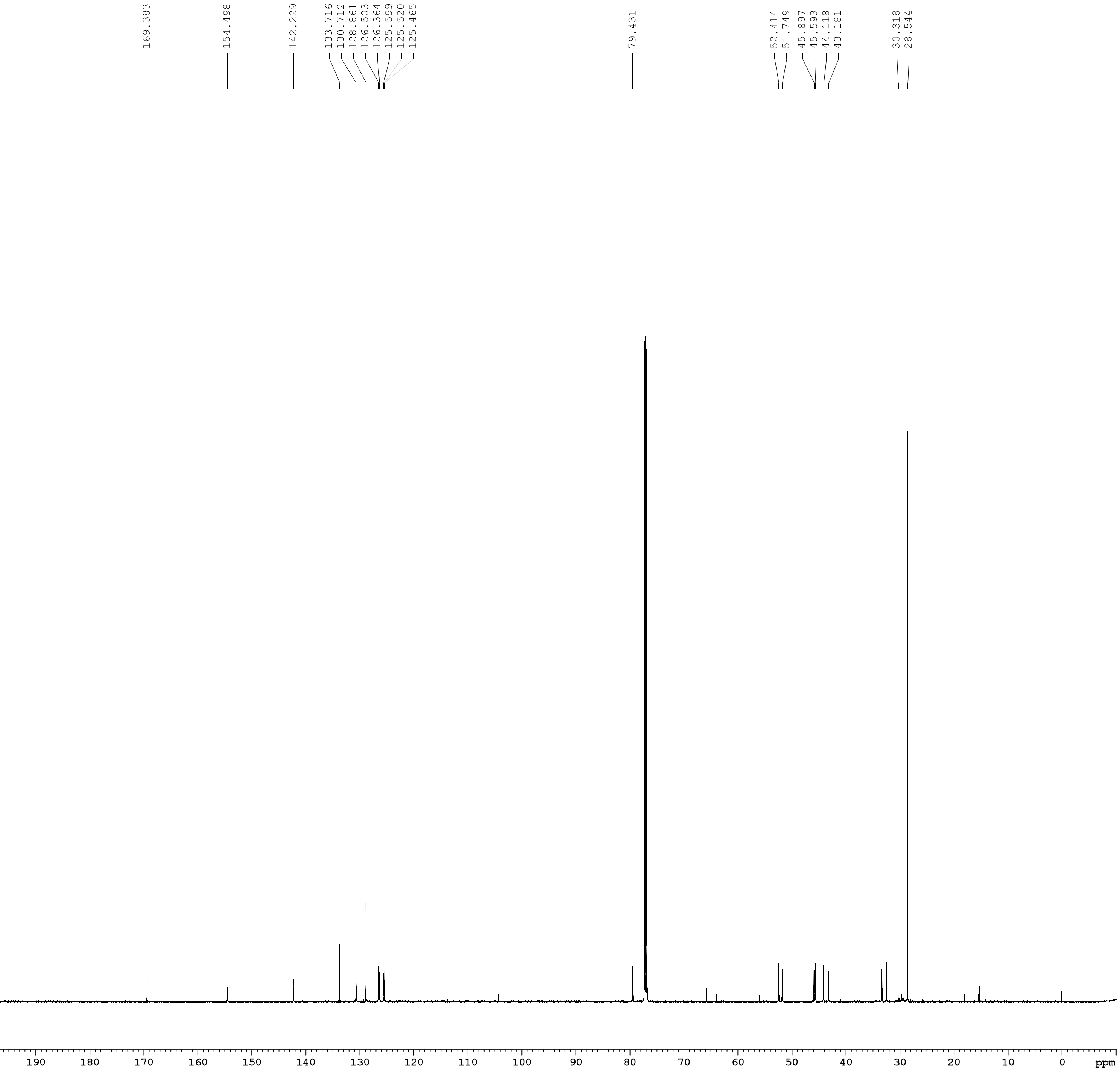


```
Current Data Parameters
NAME     LCB-1-149-600C
EXPNO                 1
PROCNO                1

F2 - Acquisition Parameters
Date_          20241015
Time              16.21
INSTRUM           av600
PROBHD   5 mm CPBBO BB-
PULPROG          zgdc30
TD                65536
SOLVENT          CDCl3T
NS                  918
DS                    4
SWH           36231.883 Hz
FIDRES         0.552855 Hz
AQ            0.9043968 sec
RG                 2050
DW               13.800 usec
DE                19.65 usec
TE                297.9 K
D1           0.40000001 sec
D11          0.03000000 sec
TD0                   1

======== CHANNEL f1 ========
SFO1        150.9194080 MHz
NUC1                13C
P1                10.00 usec
PLW1        68.40000153 W

======== CHANNEL f2 ========
SFO2        600.1330010 MHz
NUC2                 1H
CPDPRG[2        waltz16
PCPD2             80.00 usec
PLW2        30.00000000 W
PLW12        0.39811000 W

F2 - Processing parameters
SI                65536
SF          150.9028148 MHz
WDW                  EM
SSB                   0
LB                 1.00 Hz
GB                    0
PC                 1.00
```

1H spectrum

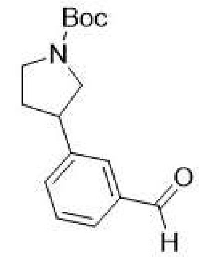

40

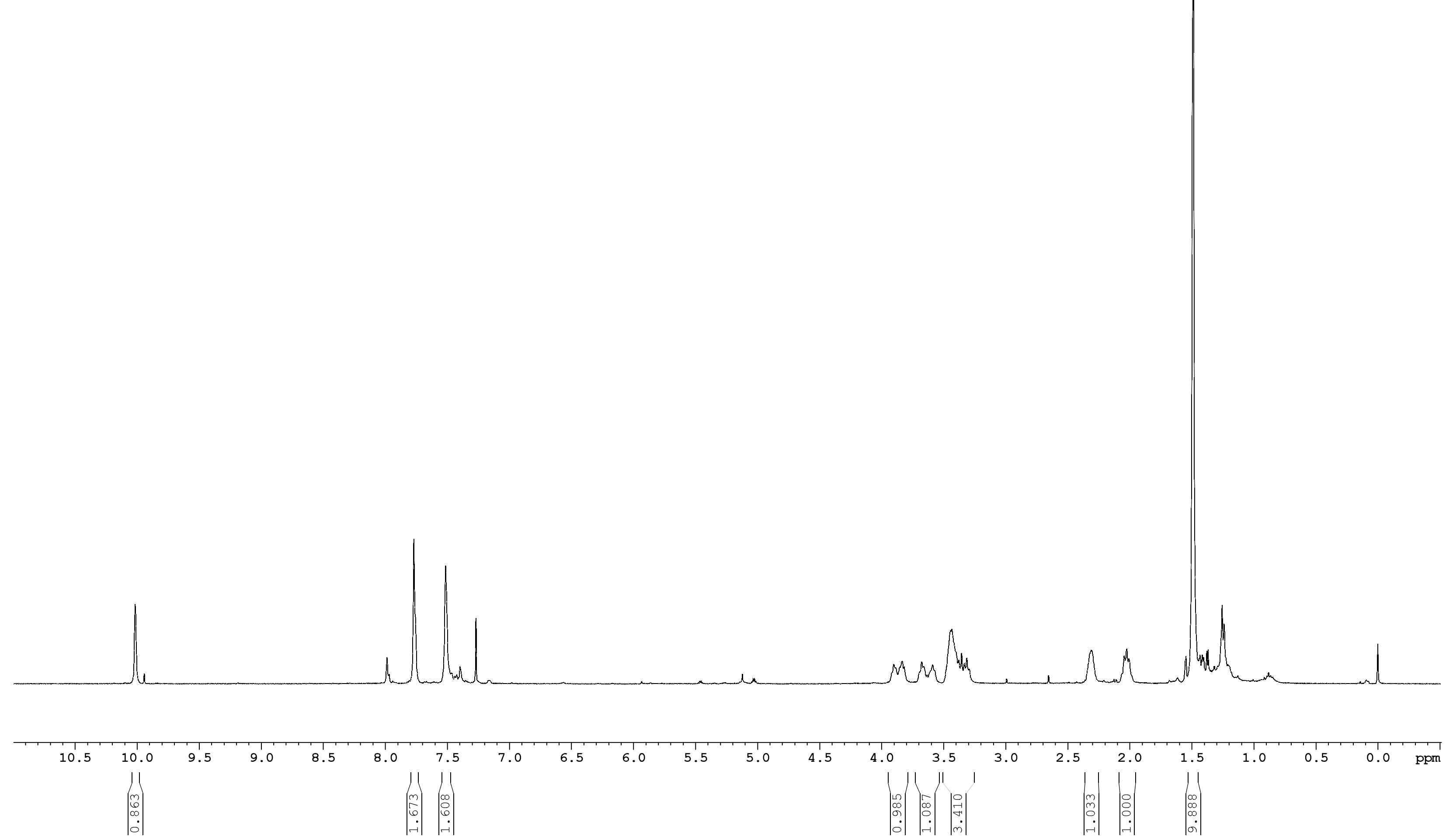


```
Current Data Parameters
NAME      LCB-1-171-ge-2
EXPNO                  1
PROCNO                 1

F2 - Acquisition Parameters
Date_           20241029
Time               14.09
INSTRUM            gn500
PROBHD   5 mm broadband
PULPROG             zg30
TD                 81728
SOLVENT           CDCl3T
NS                     8
DS                     2
SWH          8012.820 Hz
FIDRES       0.098043 Hz
AQ          5.0998273 sec
RG                   181
DW             62.400 usec
DE               6.00 usec
TE              298.0 K
D1         0.10000000 sec
MCREST              0 sec
MCWRK      0.01500000 sec

======== CHANNEL f1 ========
NUC1                  1H
P1              12.00 usec
PL1             -6.00 dB
SFO1       498.4534891 MHz

F2 - Processing parameters
SI                 65536
SF         498.4500258 MHz
WDW                   EM
SSB                    0
LB                0.30 Hz
GB                     0
PC                  1.00
```

13C spectrum with 1H decoupling

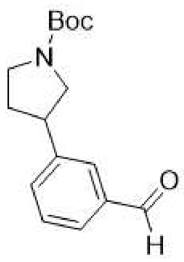

40

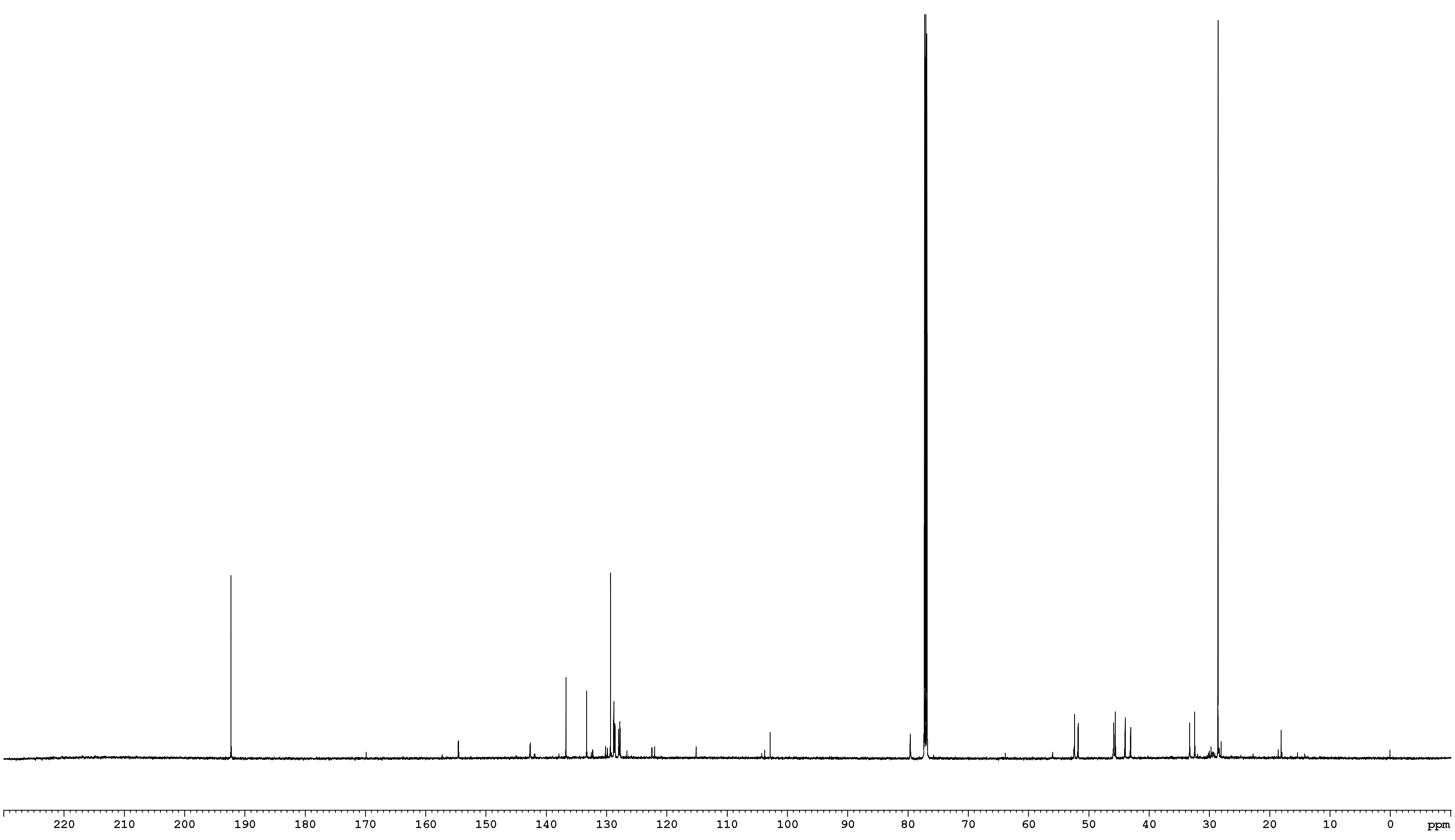

Current Data Parameters
NAME LCB-1-171-600C-3
EXPNO 1
PROCNO 1

F2 - Acquisition Parameters
Date_ 20241108
Time 9.18
INSTRUM av600
PROBHD 5 mm CPBBO BB-
PULPROG zgdc30
TD 65536
SOLVENT CDCl3T
NS 842
DS 4
SWH 36231.883 Hz
FIDRES 0.552855 Hz
AQ 0.9043968 sec
RG 2050
DW 13.800 usec
DE 19.65 usec
TE 298.0 K
D1 0.40000001 sec
D11 0.03000000 sec
TD0 1

======== CHANNEL f1 ========
SFO1 150.9194080 MHz
NUC1 13C
P1 10.00 usec
PLW1 68.40000153 W

======== CHANNEL f2 ========
SFO2 600.1330010 MHz
NUC2 1H
CPDPRG[2 waltz16
PCPD2 80.00 usec
PLW2 30.00000000 W
PLW12 0.39811000 W

F2 - Processing parameters
SI 65536
SF 150.9028147 MHz
WDW EM
SSB 0
LB 1.00 Hz
GB 0
PC 1.00

1H spectrum

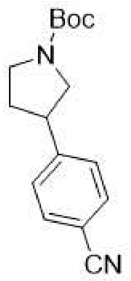

41

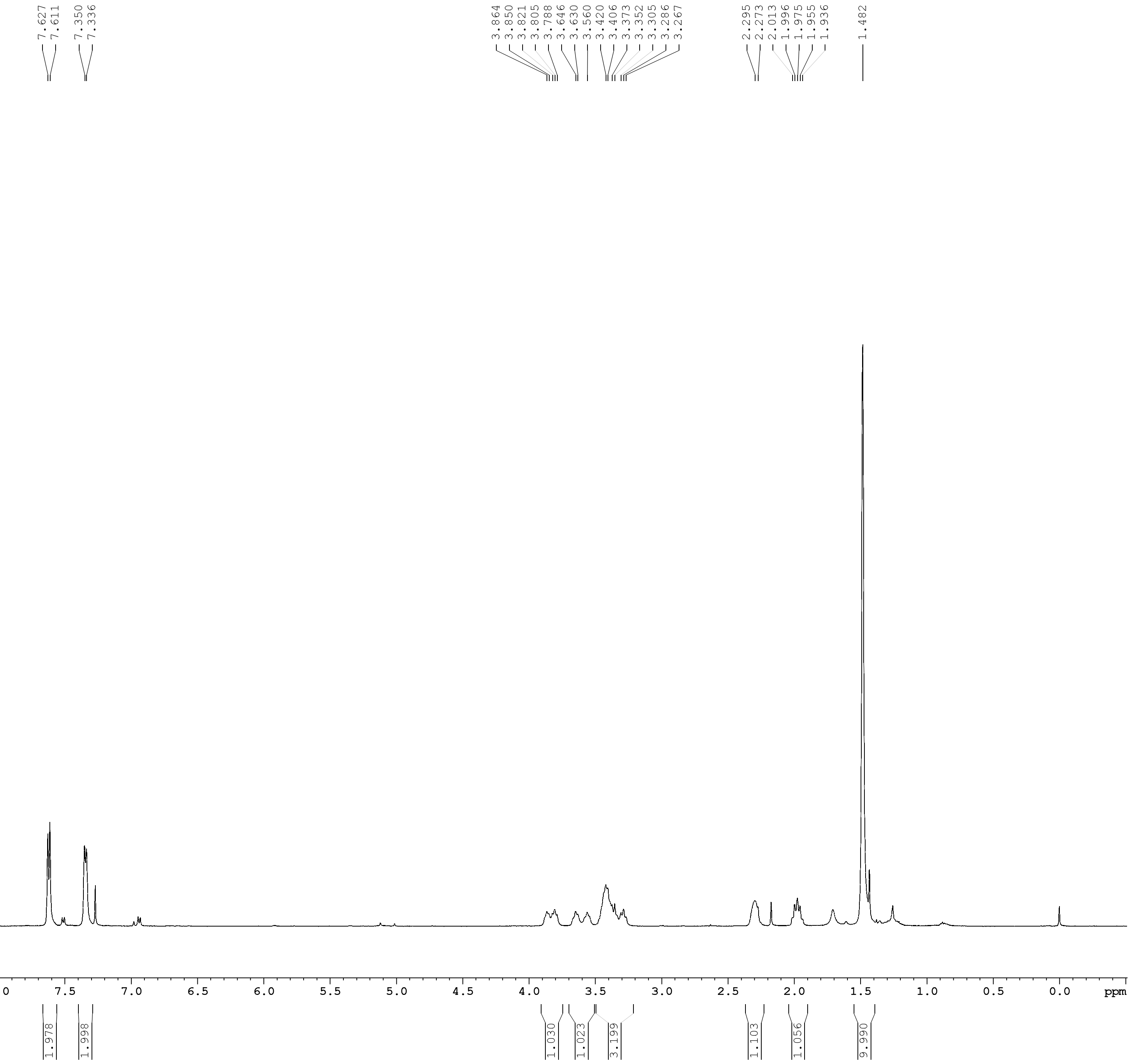

Current Data Parameters
NAME LCB-1-101-ge
EXPNO 1
PROCNO 1

F2 - Acquisition Parameters
Date_ 20240802
Time 13.26
INSTRUM gn500
PROBHD 5 mm broadband
PULPROG zg30
TD 48074
SOLVENT CDCl3T
NS 8
DS 2
SWH 8012.820 Hz
FIDRES 0.166677 Hz
AQ 2.9998176 sec
RG 161.3
DW 62.400 usec
DE 6.00 usec
TE 298.0 K
D1 0.10000000 sec
MCREST 0 sec
MCWRK 0.01500000 sec

======== CHANNEL f1 ========
NUC1 1H
P1 12.00 usec
PL1 -6.00 dB
SFO1 498.4534891 MHz

F2 - Processing parameters
SI 65536
SF 498.4500250 MHz
WDW EM
SSB 0
LB 0.30 Hz
GB 0
PC 1.00

13C spectrum with 1H decoupling

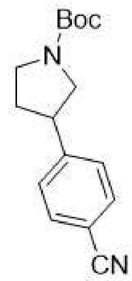

41

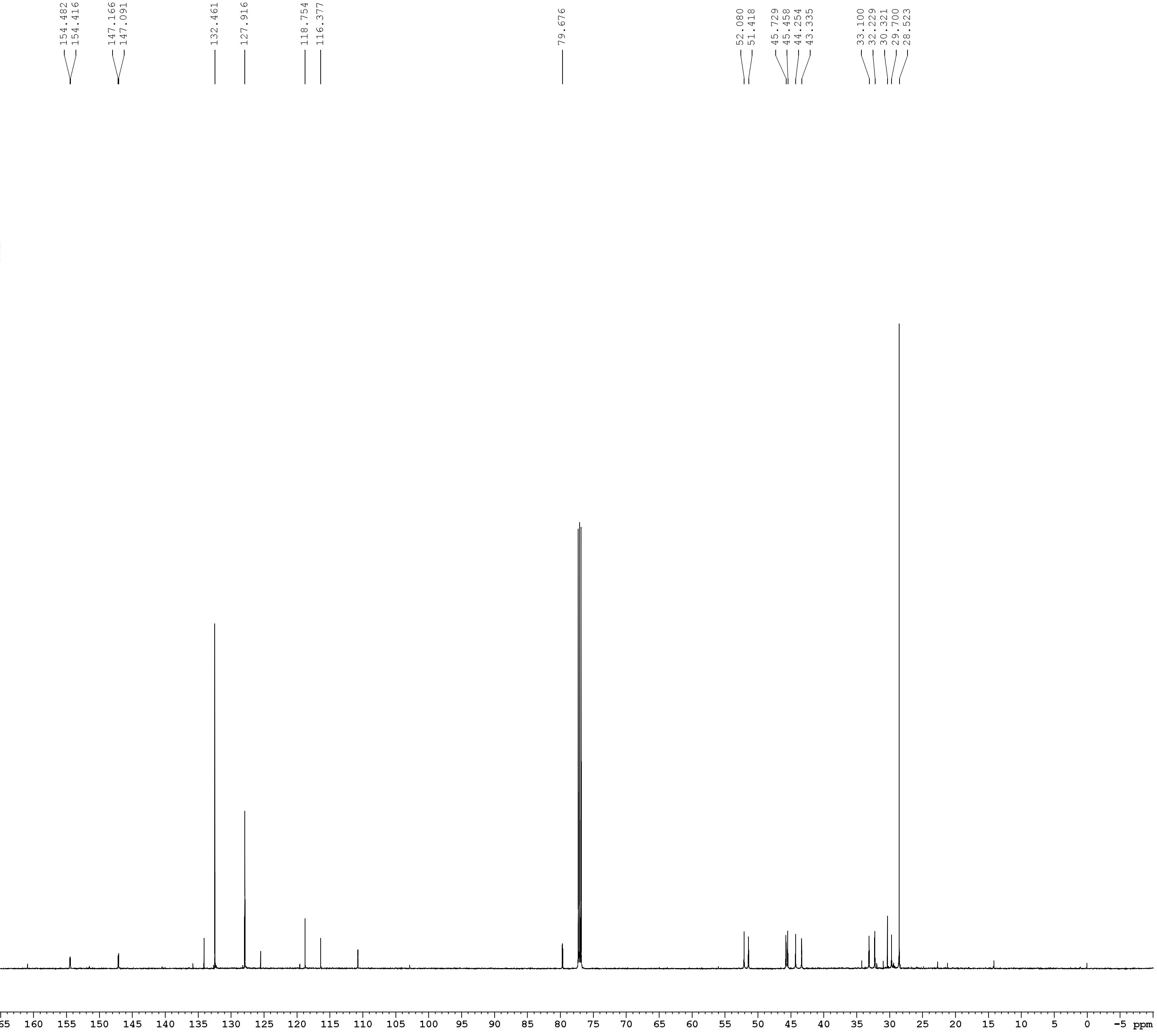


```
Current Data Parameters
NAME     LCB-1-101-600C
EXPNO                 1
PROCNO                1

F2 - Acquisition Parameters
Date_          20240801
Time               9.40
INSTRUM           av600
PROBHD   5 mm CPBBO BB-
PULPROG          zgdc30
TD                65536
SOLVENT          CDCl3T
NS                  874
DS                    4
SWH           36231.883 Hz
FIDRES         0.552855 Hz
AQ            0.9043968 sec
RG                 2050
DW               13.800 usec
DE                19.65 usec
TE                298.0 K
D1           0.40000001 sec
D11          0.03000000 sec
TD0                   1

======== CHANNEL f1 ========
SFO1        150.9194080 MHz
NUC1                13C
P1                10.00 usec
PLW1        68.40000153 W

======== CHANNEL f2 ========
SFO2        600.1330010 MHz
NUC2                 1H
CPDPRG[2        waltz16
PCPD2             80.00 usec
PLW2        30.00000000 W
PLW12        0.39811000 W

F2 - Processing parameters
SI                65536
SF          150.9028143 MHz
WDW                  EM
SSB                   0
LB                 1.00 Hz
GB                    0
PC                 1.00
```

1H spectrum

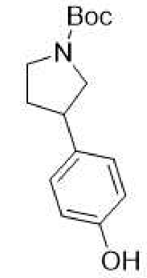

42

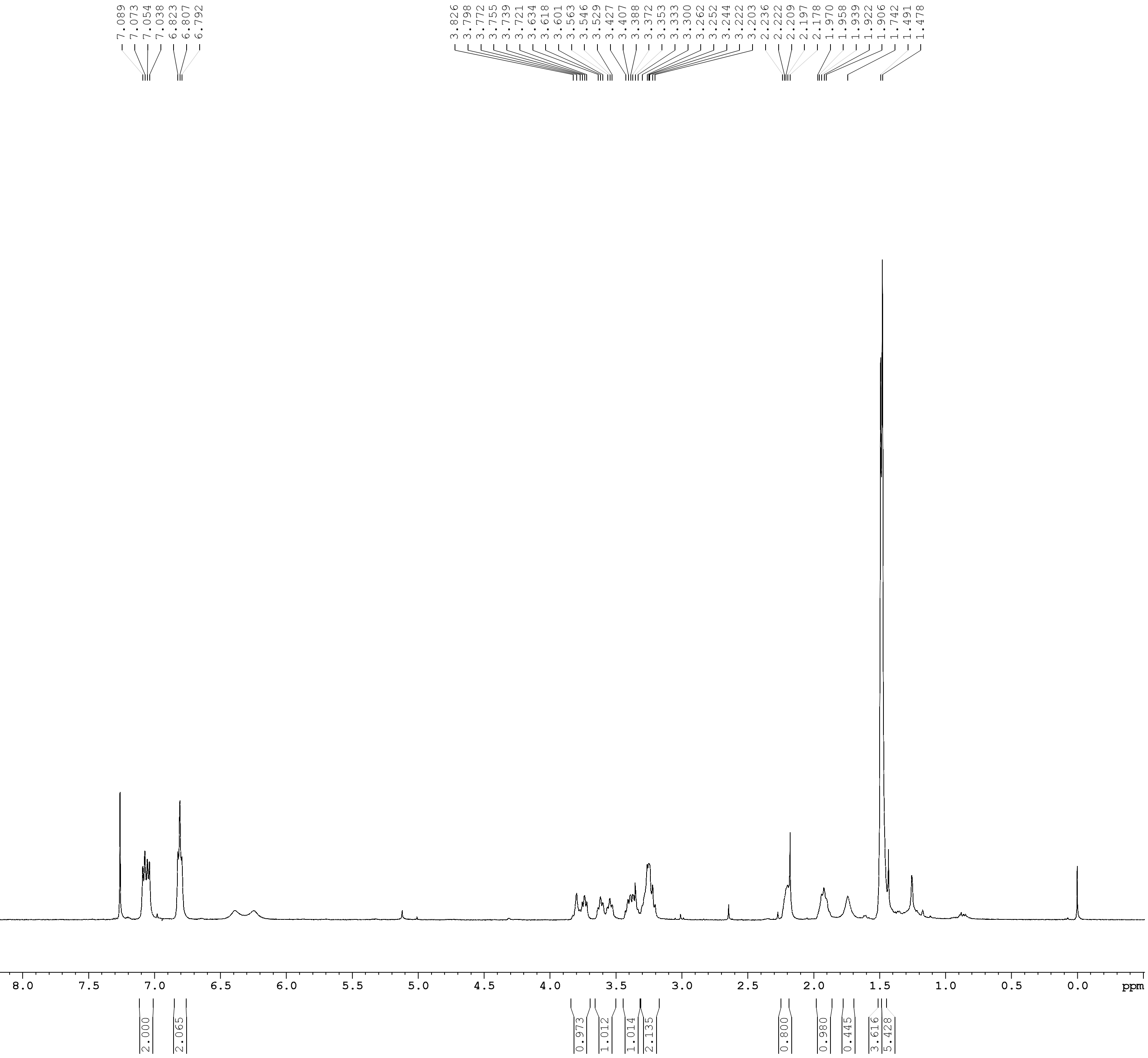


```
Current Data Parameters
NAME       LCB-1-107-ge
EXPNO                 1
PROCNO                1

F2 - Acquisition Parameters
Date_          20240806
Time              12.04
INSTRUM           gn500
PROBHD   5 mm broadband
PULPROG            zg30
TD                81728
SOLVENT          CDCl3T
NS                    8
DS                    2
SWH            8012.820 Hz
FIDRES         0.098043 Hz
AQ            5.0998273 sec
RG                574.7
DW               62.400 usec
DE                 6.00 usec
TE                298.0 K
D1           0.10000000 sec
MCREST                0 sec
MCWRK        0.01500000 sec

======== CHANNEL f1 ========
NUC1                 1H
P1                12.00 usec
PL1               -6.00 dB
SFO1        498.4534891 MHz

F2 - Processing parameters
SI                65536
SF          498.4500293 MHz
WDW                  EM
SSB                   0
LB                 0.30 Hz
GB                    0
PC                 1.00
```

13C spectrum with 1H decoupling

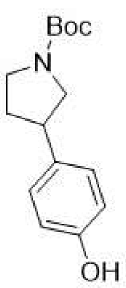

42

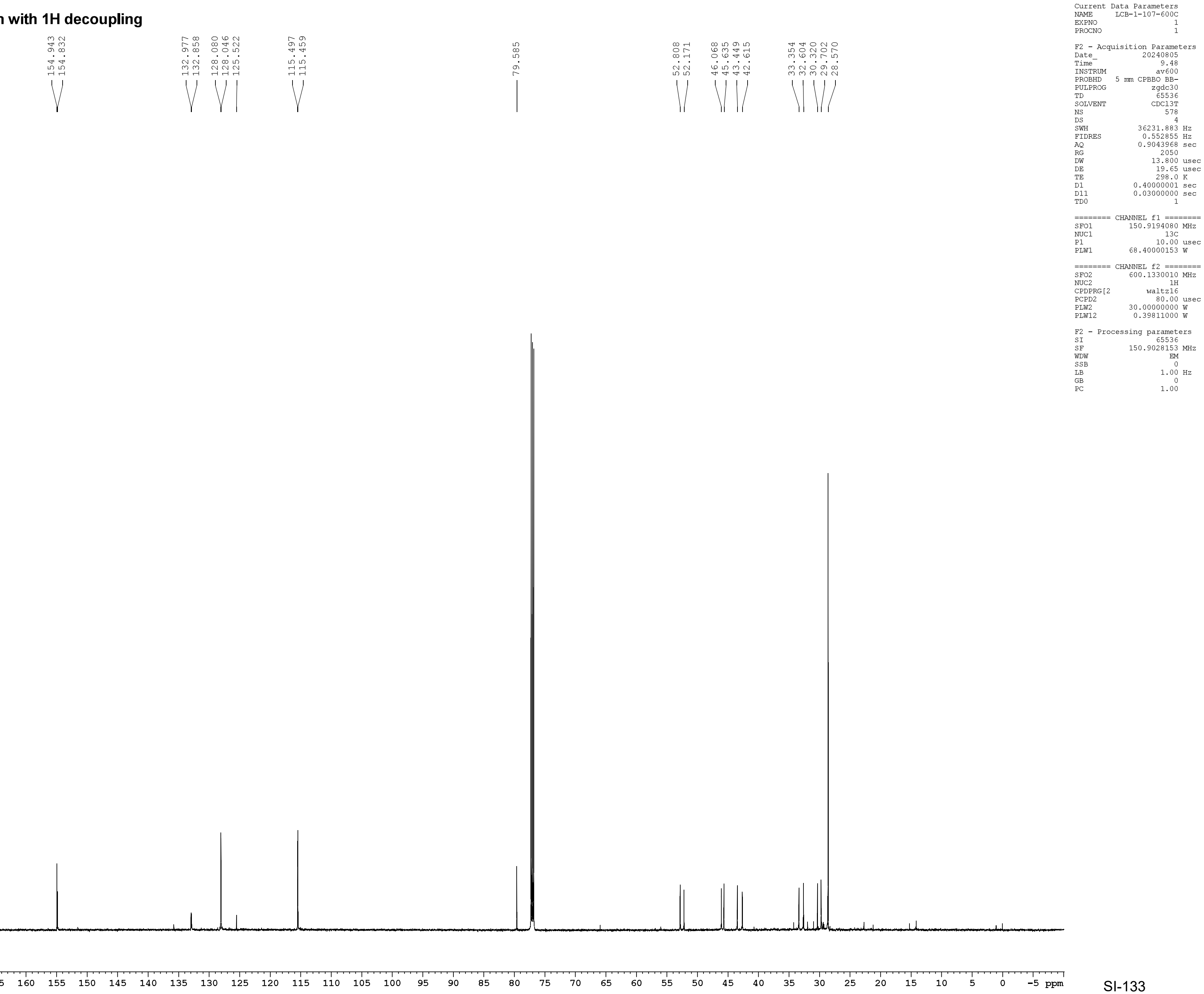

1H spectrum

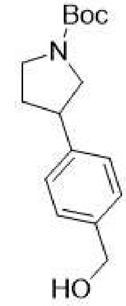

43

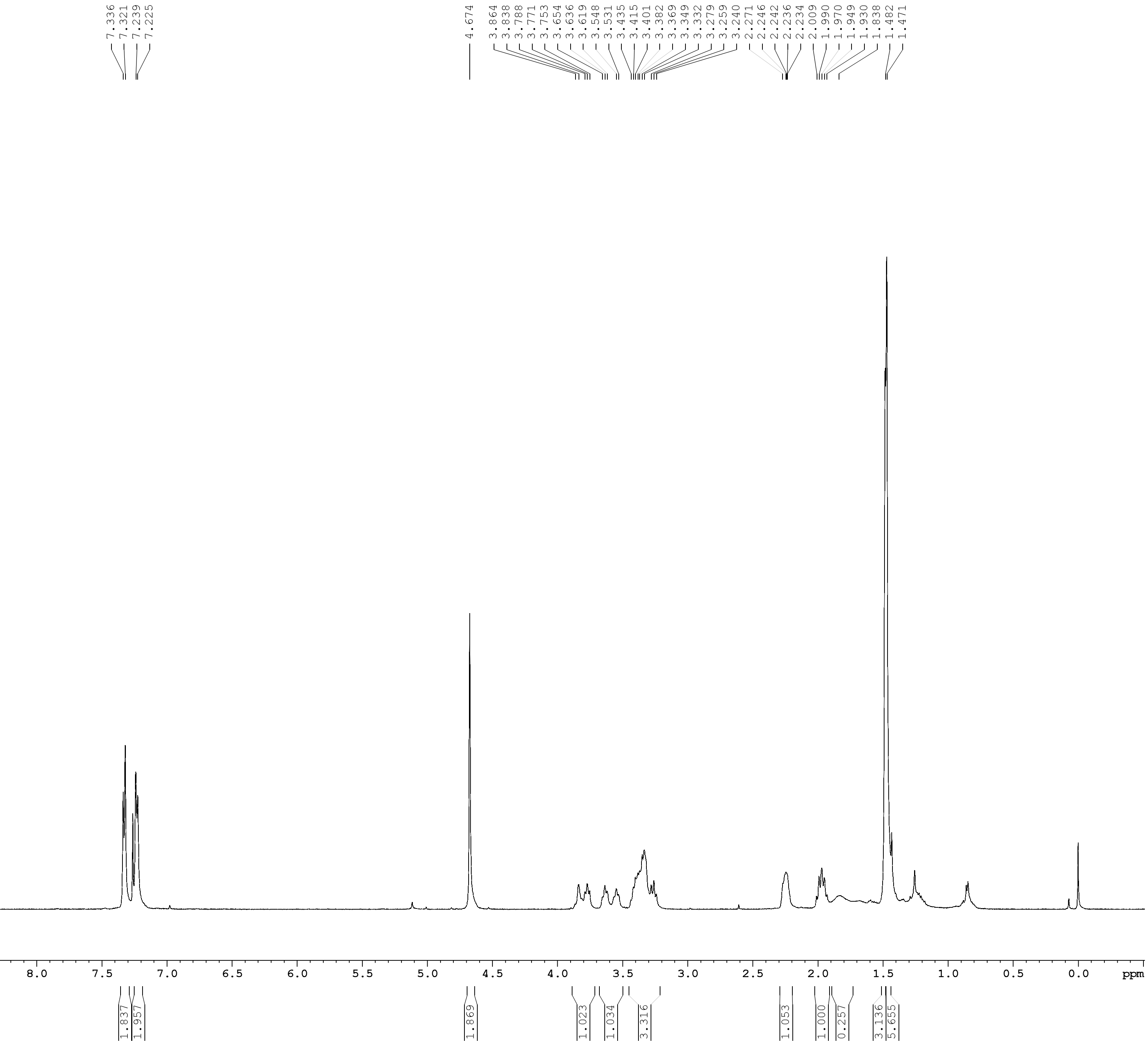


```
Current Data Parameters
NAME       LCB-1-112-ge
EXPNO                 1
PROCNO                1

F2 - Acquisition Parameters
Date_          20240815
Time              15.40
INSTRUM           gn500
PROBHD   5 mm broadband
PULPROG            zg30
TD                81728
SOLVENT          CDCl3T
NS                    8
DS                    2
SWH            8012.820 Hz
FIDRES         0.098043 Hz
AQ            5.0998273 sec
RG                574.7
DW               62.400 usec
DE                 6.00 usec
TE                298.0 K
D1           0.10000000 sec
MCREST                0 sec
MCWRK        0.01500000 sec

======== CHANNEL f1 ========
NUC1                 1H
P1                12.00 usec
PL1               -6.00 dB
SFO1        498.4534891 MHz

F2 - Processing parameters
SI                65536
SF          498.4500288 MHz
WDW                  EM
SSB                   0
LB                 0.30 Hz
GB                    0
PC                 1.00
```

13C spectrum with 1H decoupling

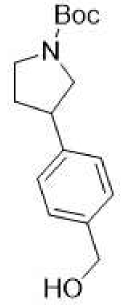

43

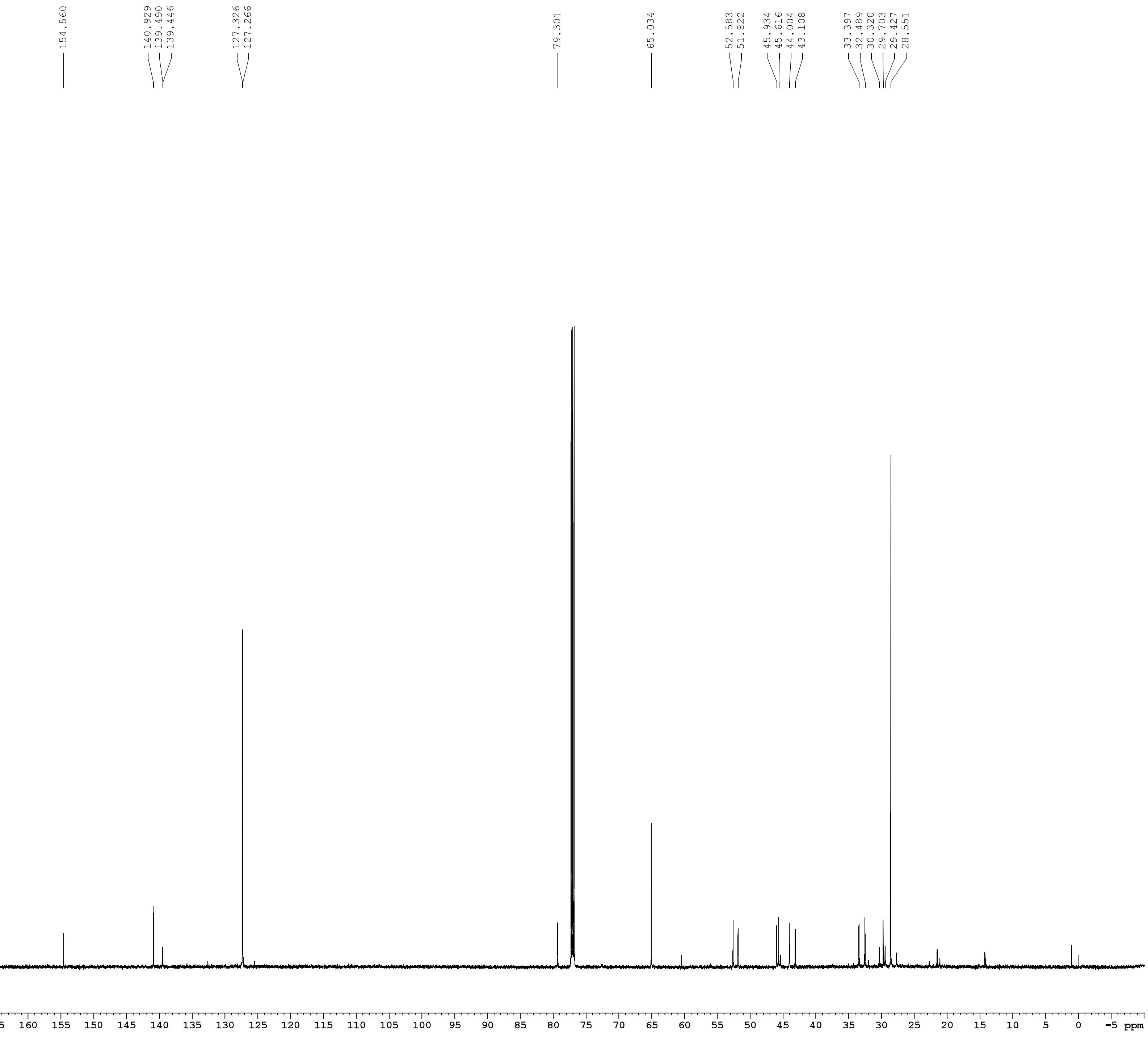


```
Current Data Parameters
NAME      LCB-1-112-600C
EXPNO                  1
PROCNO                 1

F2 - Acquisition Parameters
Date_           20240814
Time               16.28
INSTRUM            av600
PROBHD   5 mm CPBBO BB-
PULPROG          zgdc30
TD                 65536
SOLVENT           CDCl3T
NS                   355
DS                     4
SWH           36231.883 Hz
FIDRES         0.552855 Hz
AQ            0.9043968 sec
RG                  2050
DW                13.800 usec
DE                 19.65 usec
TE                 298.0 K
D1            0.40000001 sec
D11           0.03000000 sec
TD0                    1

======== CHANNEL f1 ========
SFO1        150.9194080 MHz
NUC1                 13C
P1                 10.00 usec
PLW1         68.40000153 W

======== CHANNEL f2 ========
SFO2        600.1330010 MHz
NUC2                  1H
CPDPRG[2         waltz16
PCPD2              80.00 usec
PLW2         30.00000000 W
PLW12         0.39811000 W

F2 - Processing parameters
SI                 65536
SF          150.9028150 MHz
WDW                   EM
SSB                    0
LB                  1.00 Hz
GB                     0
PC                  1.00
```

1H spectrum

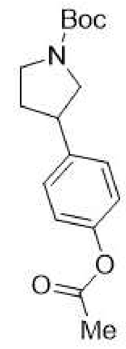

44

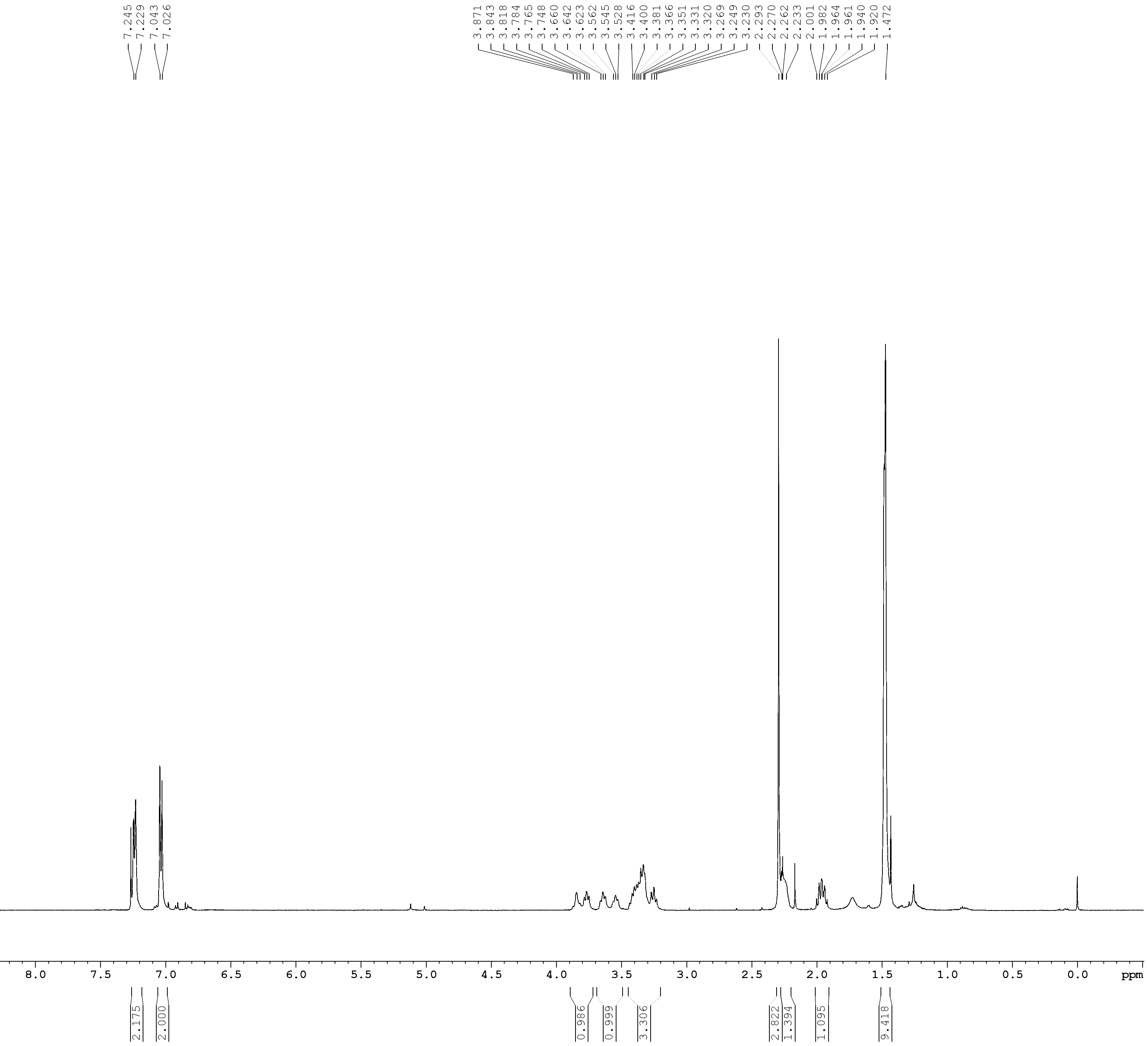


```
Current Data Parameters
NAME       LCB-1-102-ge
EXPNO                 1
PROCNO                1

F2 - Acquisition Parameters
Date_          20240806
Time              12.07
INSTRUM           gn500
PROBHD   5 mm broadband
PULPROG            zg30
TD                81728
SOLVENT          CDCl3T
NS                    8
DS                    2
SWH          8012.820 Hz
FIDRES       0.098043 Hz
AQ          5.0998273 sec
RG                161.3
DW              62.400 usec
DE                6.00 usec
TE               298.0 K
D1          0.10000000 sec
MCREST                0 sec
MCWRK       0.01500000 sec

======== CHANNEL f1 ========
NUC1                 1H
P1               12.00 usec
PL1              -6.00 dB
SFO1        498.4534891 MHz

F2 - Processing parameters
SI                65536
SF          498.4500274 MHz
WDW                  EM
SSB                   0
LB                 0.30 Hz
GB                    0
PC                 1.00
```

13C spectrum with 1H decoupling

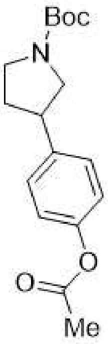

44

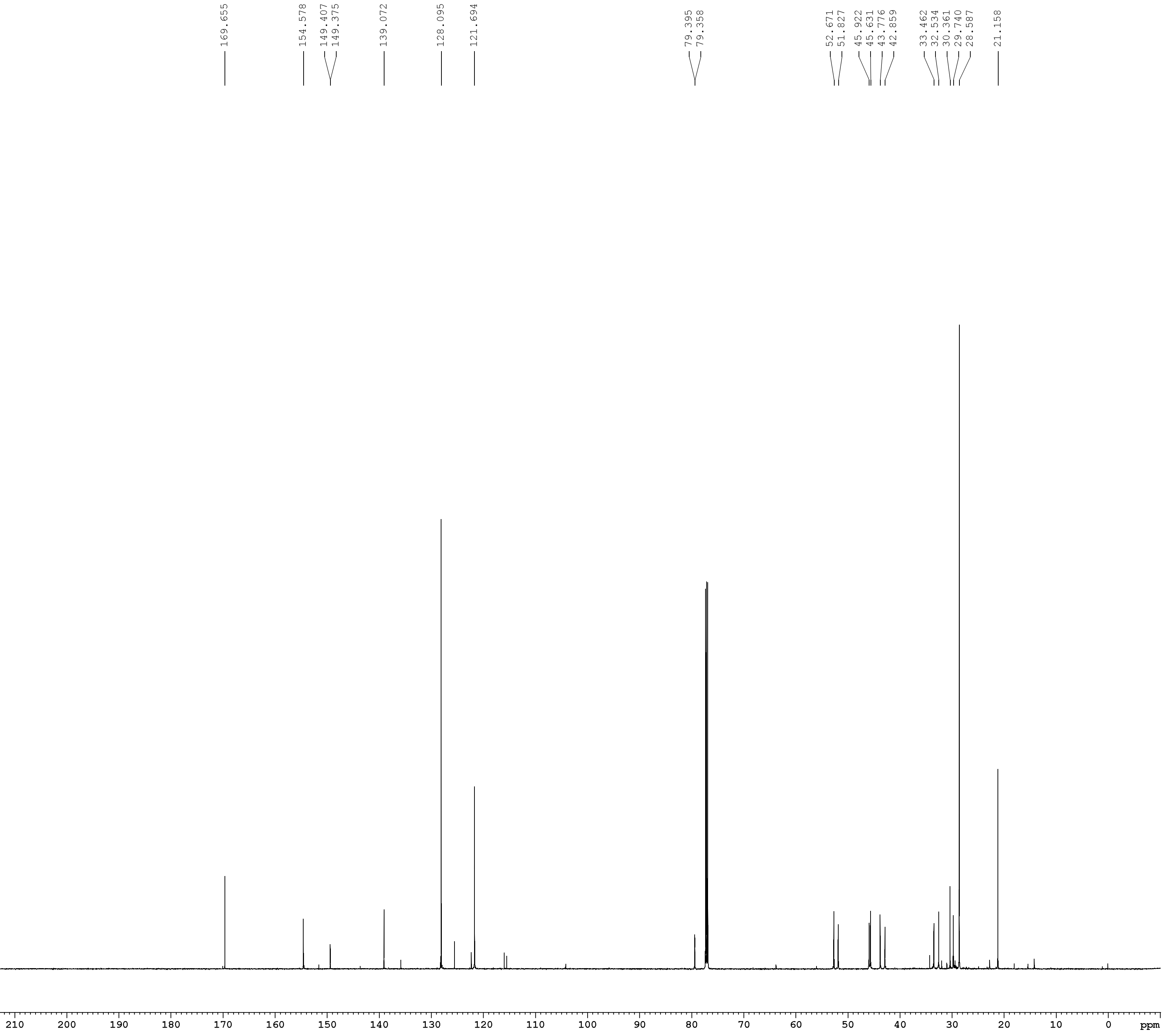


```
Current Data Parameters
NAME     LCB-1-102-600C2
EXPNO              1
PROCNO             1

F2 - Acquisition Parameters
Date_         20240802
Time             13.47
INSTRUM          av600
PROBHD   5 mm CPBBO BB-
PULPROG         zgdc30
TD               65536
SOLVENT         CDCl3T
NS                 849
DS                   4
SWH          36231.883 Hz
FIDRES        0.552855 Hz
AQ           0.9043968 sec
RG                2050
DW              13.800 usec
DE               19.65 usec
TE               297.9 K
D1          0.40000001 sec
D11         0.03000000 sec
TD0                  1

======== CHANNEL f1 ========
SFO1       150.9194080 MHz
NUC1               13C
P1               10.00 usec
PLW1       68.40000153 W

======== CHANNEL f2 ========
SFO2       600.1330010 MHz
NUC2                1H
CPDPRG[2        waltz16
PCPD2            80.00 usec
PLW2       30.00000000 W
PLW12       0.39811000 W

F2 - Processing parameters
SI               65536
SF         150.9028085 MHz
WDW                 EM
SSB                  0
LB                1.00 Hz
GB                   0
PC                1.00
```

1H spectrum

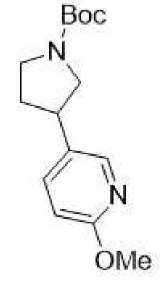

45

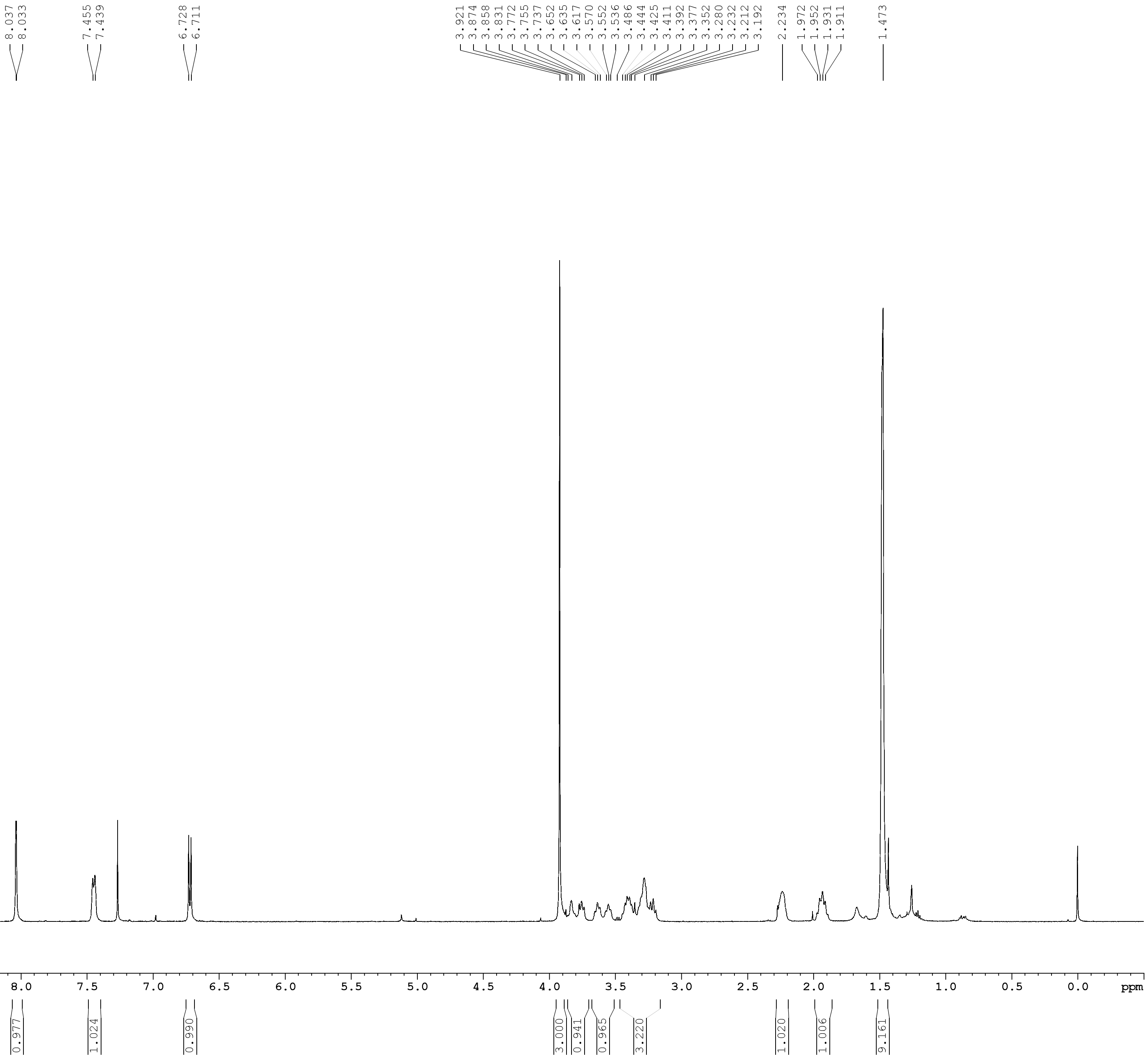


```
Current Data Parameters
NAME       LCB-1-114-ge
EXPNO                 1
PROCNO                1

F2 - Acquisition Parameters
Date_          20240815
Time              15.43
INSTRUM           gn500
PROBHD   5 mm broadband
PULPROG            zg30
TD                81728
SOLVENT          CDCl3T
NS                    8
DS                    2
SWH            8012.820 Hz
FIDRES         0.098043 Hz
AQ            5.0998273 sec
RG                645.1
DW               62.400 usec
DE                 6.00 usec
TE                298.0 K
D1           0.10000000 sec
MCREST                0 sec
MCWRK        0.01500000 sec

======== CHANNEL f1 ========
NUC1                 1H
P1                12.00 usec
PL1               -6.00 dB
SFO1        498.4534891 MHz

F2 - Processing parameters
SI                65536
SF          498.4500266 MHz
WDW                  EM
SSB                   0
LB                 0.30 Hz
GB                    0
PC                 1.00
```

13C spectrum with 1H decoupling

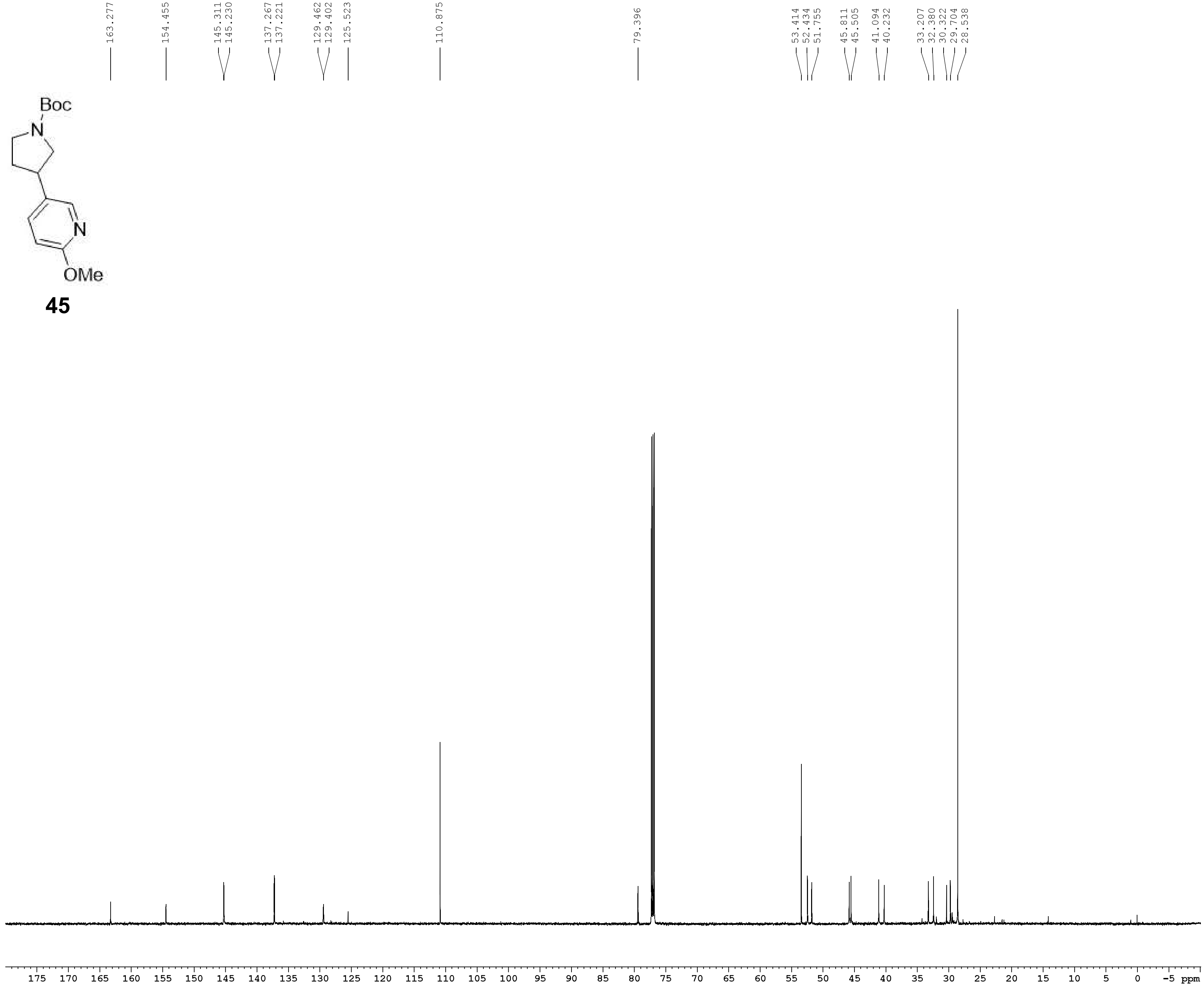

45

Current Data Parameters
NAME LCB-1-114-600C
EXPNO 1
PROCNO 1

F2 - Acquisition Parameters
Date_ 20240814
Time 16.10
INSTRUM av600
PROBHD 5 mm CPBBO BB-
PULPROG zgdc30
TD 65536
SOLVENT CDCl3T
NS 375
DS 4
SWH 36231.883 Hz
FIDRES 0.552855 Hz
AQ 0.9043968 sec
RG 2050
DW 13.800 usec
DE 19.65 usec
TE 298.0 K
D1 0.40000001 sec
D11 0.03000000 sec
TD0 1

======== CHANNEL f1 ========
SFO1 150.9194080 MHz
NUC1 13C
P1 10.00 usec
PLW1 68.40000153 W

======== CHANNEL f2 ========
SFO2 600.1330010 MHz
NUC2 1H
CPDPRG[2 waltz16
PCPD2 80.00 usec
PLW2 30.00000000 W
PLW12 0.39811000 W

F2 - Processing parameters
SI 65536
SF 150.9028134 MHz
WDW EM
SSB 0
LB 1.00 Hz
GB 0
PC 1.00

1H spectrum

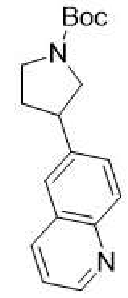

46

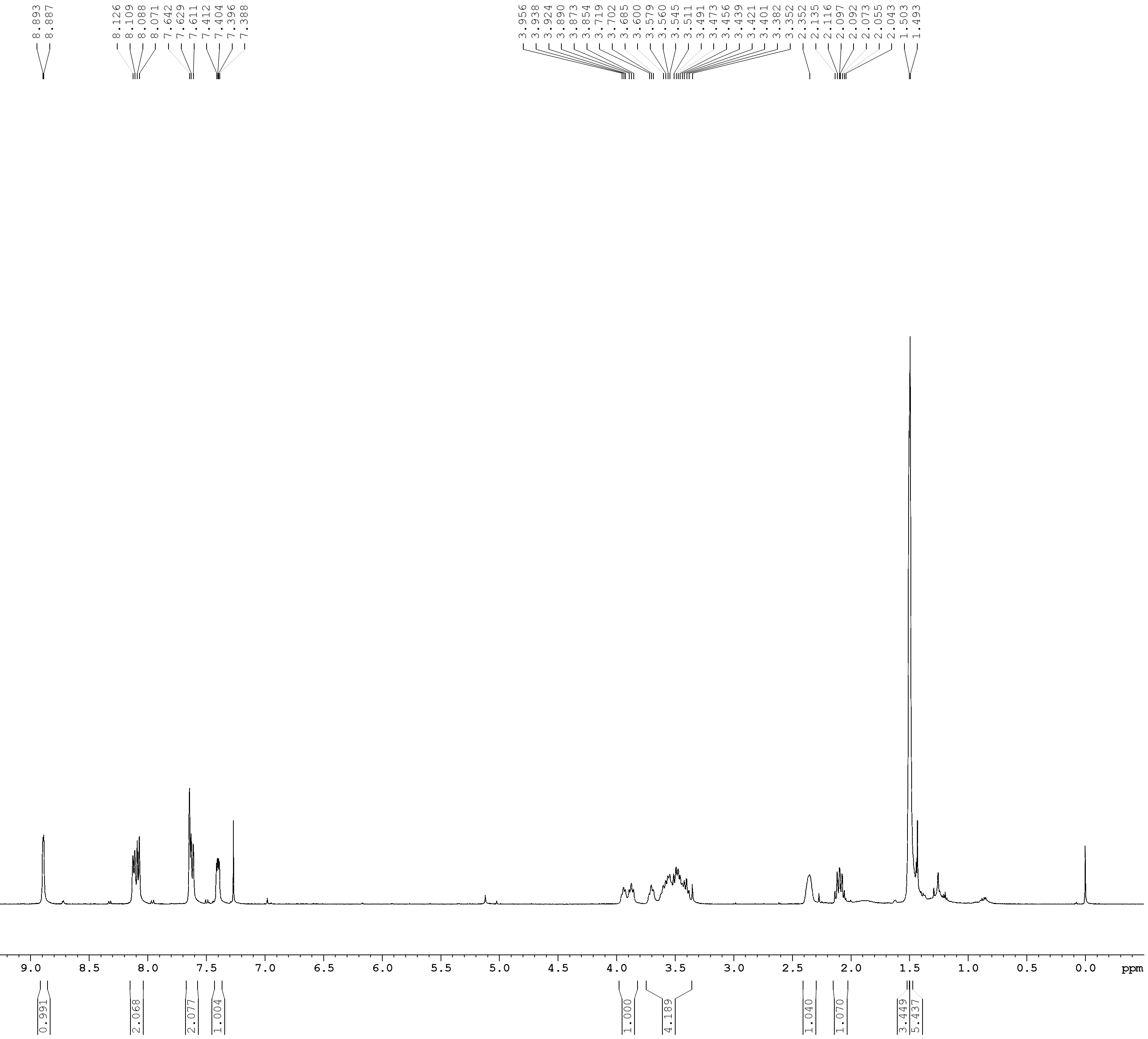


```
Current Data Parameters
NAME        LCB-1-120-ge
EXPNO                  1
PROCNO                 1

F2 - Acquisition Parameters
Date_           20240821
Time                9.48
INSTRUM            gn500
PROBHD   5 mm broadband
PULPROG             zg30
TD                 81728
SOLVENT           CDCl3T
NS                     8
DS                     2
SWH             8012.820 Hz
FIDRES          0.098043 Hz
AQ             5.0998273 sec
RG                   512
DW                62.400 usec
DE                  6.00 usec
TE                 298.0 K
D1            0.10000000 sec
MCREST                 0 sec
MCWRK         0.01500000 sec

======== CHANNEL f1 ========
NUC1                  1H
P1                 12.00 usec
PL1                -6.00 dB
SFO1         498.4534891 MHz

F2 - Processing parameters
SI                 65536
SF           498.4500258 MHz
WDW                   EM
SSB                    0
LB                  0.30 Hz
GB                     0
PC                  1.00
```

13C spectrum with 1H decoupling

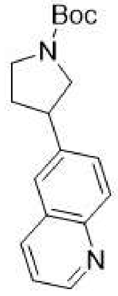

46

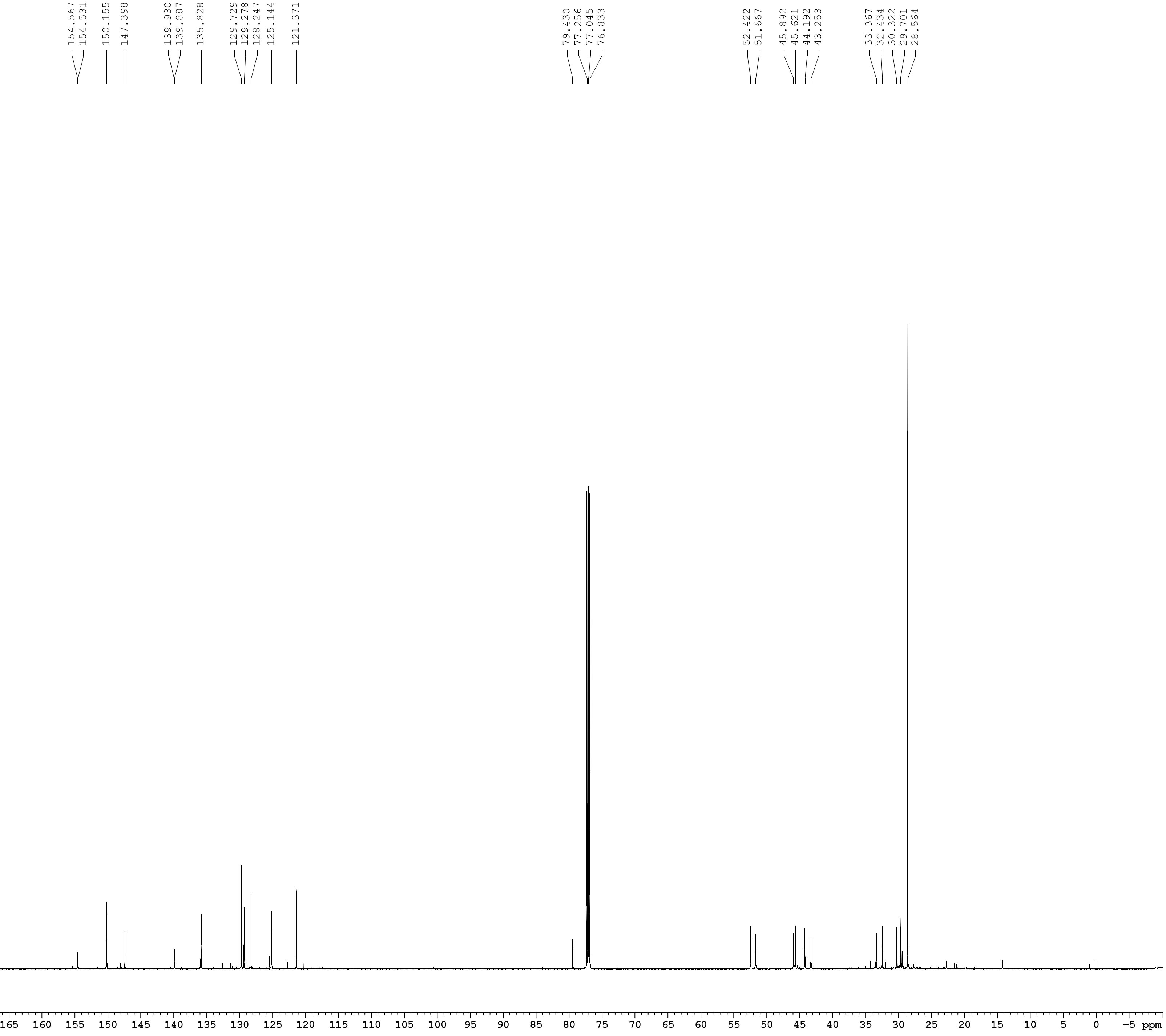


```
Current Data Parameters
NAME     LCB-1-120-600C
EXPNO                 1
PROCNO                1

F2 - Acquisition Parameters
Date_          20240816
Time               9.27
INSTRUM           av600
PROBHD   5 mm CPBBO BB-
PULPROG         zgdc30
TD                65536
SOLVENT          CDCl3T
NS                  822
DS                    4
SWH           36231.883 Hz
FIDRES         0.552855 Hz
AQ            0.9043968 sec
RG                 2050
DW               13.800 usec
DE                19.65 usec
TE                297.9 K
D1           0.40000001 sec
D11          0.03000000 sec
TD0                   1

======== CHANNEL f1 ========
SFO1        150.9194080 MHz
NUC1                13C
P1                10.00 usec
PLW1        68.40000153 W

======== CHANNEL f2 ========
SFO2        600.1330010 MHz
NUC2                 1H
CPDPRG[2         waltz16
PCPD2             80.00 usec
PLW2        30.00000000 W
PLW12        0.39811000 W

F2 - Processing parameters
SI                65536
SF          150.9028141 MHz
WDW                  EM
SSB                   0
LB                 1.00 Hz
GB                    0
PC                 1.00
```

1H spectrum

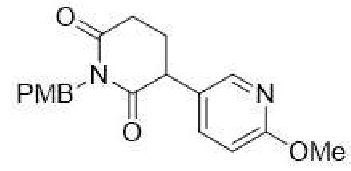

47

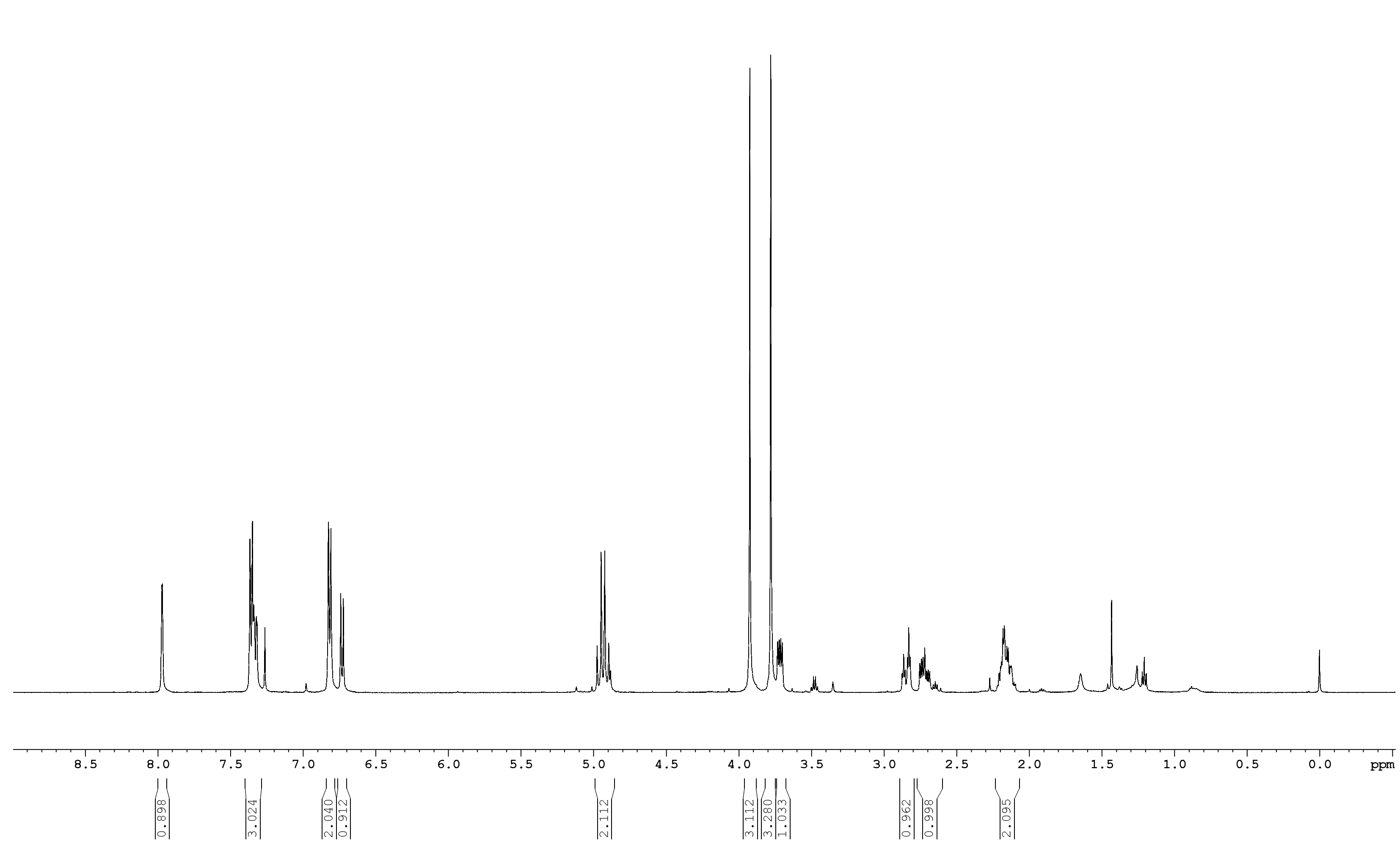

Current Data Parameters
NAME LCB-1-181-fr28-50
EXPNO 1
PROCNO 1

F2 - Acquisition Parameters
Date_ 20241023
Time 9.19
INSTRUM gn500
PROBHD 5 mm broadband
PULPROG zg30
TD 48074
SOLVENT CDCl3T
NS 8
DS 2
SWH 8012.820 Hz
FIDRES 0.166677 Hz
AQ 2.9998176 sec
RG 181
DW 62.400 usec
DE 6.00 usec
TE 298.0 K
D1 0.10000000 sec
MCREST 0 sec
MCWRK 0.01500000 sec

======== CHANNEL f1 ========
NUC1 1H
P1 12.00 usec
PL1 -6.00 dB
SFO1 498.4534891 MHz

F2 - Processing parameters
SI 65536
SF 498.4500287 MHz
WDW EM
SSB 0
LB 0.30 Hz
GB 0
PC 1.00

13C spectrum with 1H decoupling

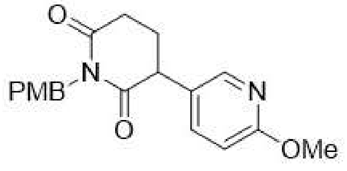

47

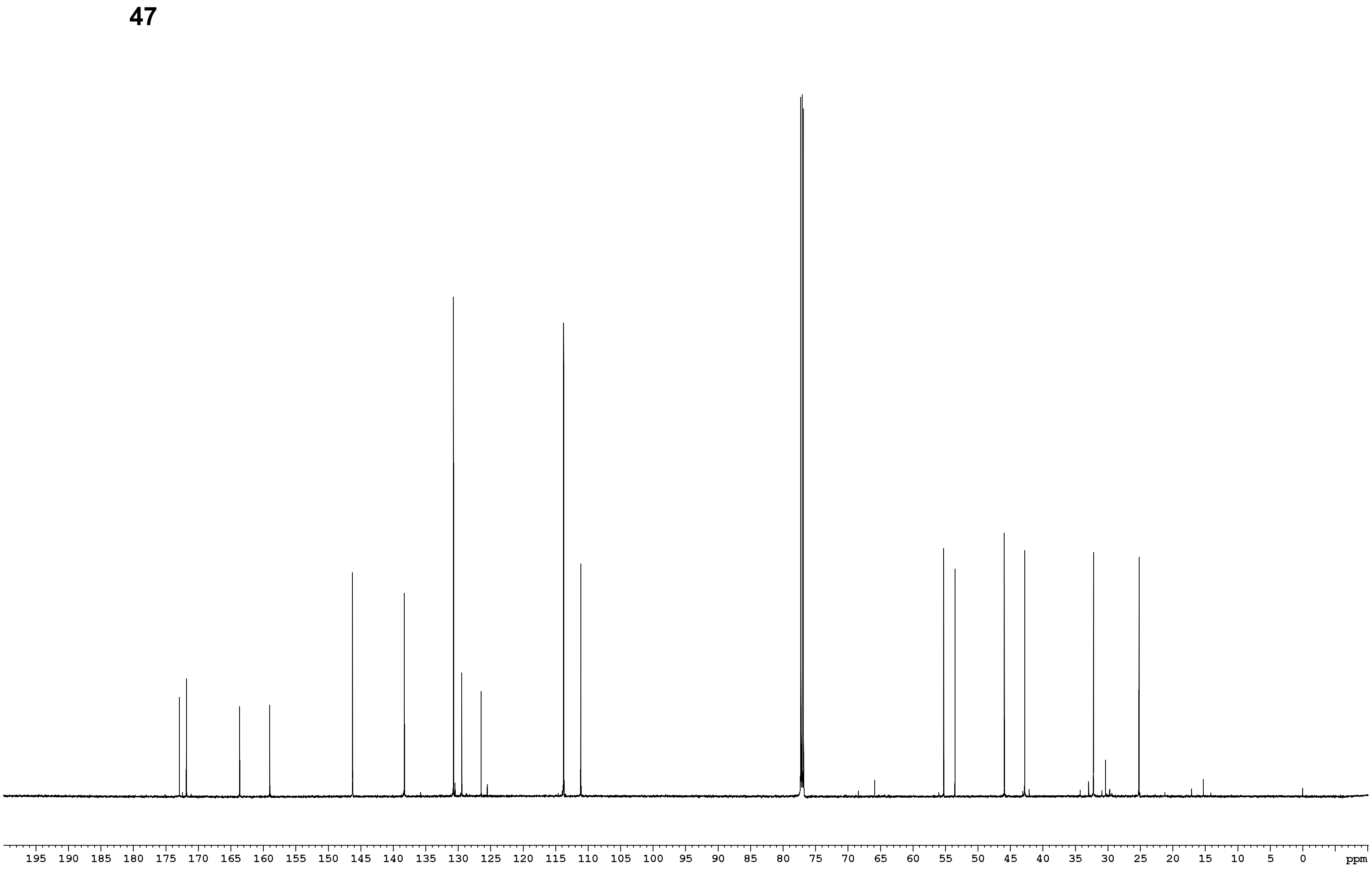


```
Current Data Parameters
NAME     LCB-1-181-600C
EXPNO                 1
PROCNO                1

F2 - Acquisition Parameters
Date_          20241023
Time               9.34
INSTRUM           av600
PROBHD   5 mm CPBBO BB-
PULPROG         zgdc30
TD                65536
SOLVENT          CDCl3T
NS                  502
DS                    4
SWH          36231.883 Hz
FIDRES        0.552855 Hz
AQ           0.9043968 sec
RG                 2050
DW               13.800 usec
DE                19.65 usec
TE                298.2 K
D1           0.40000001 sec
D11          0.03000000 sec
TD0                   1

======== CHANNEL f1 ========
SFO1       150.9194080 MHz
NUC1                13C
P1                10.00 usec
PLW1        68.40000153 W

======== CHANNEL f2 ========
SFO2       600.1330010 MHz
NUC2                 1H
CPDPRG[2          waltz16
PCPD2             80.00 usec
PLW2        30.00000000 W
PLW12        0.39811000 W

F2 - Processing parameters
SI                65536
SF         150.9028154 MHz
WDW                  EM
SSB                   0
LB                 1.00 Hz
GB                    0
PC                 1.00
```

1H spectrum

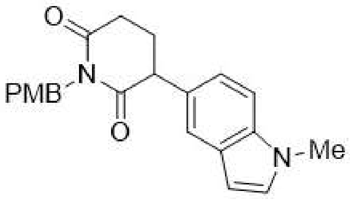

48

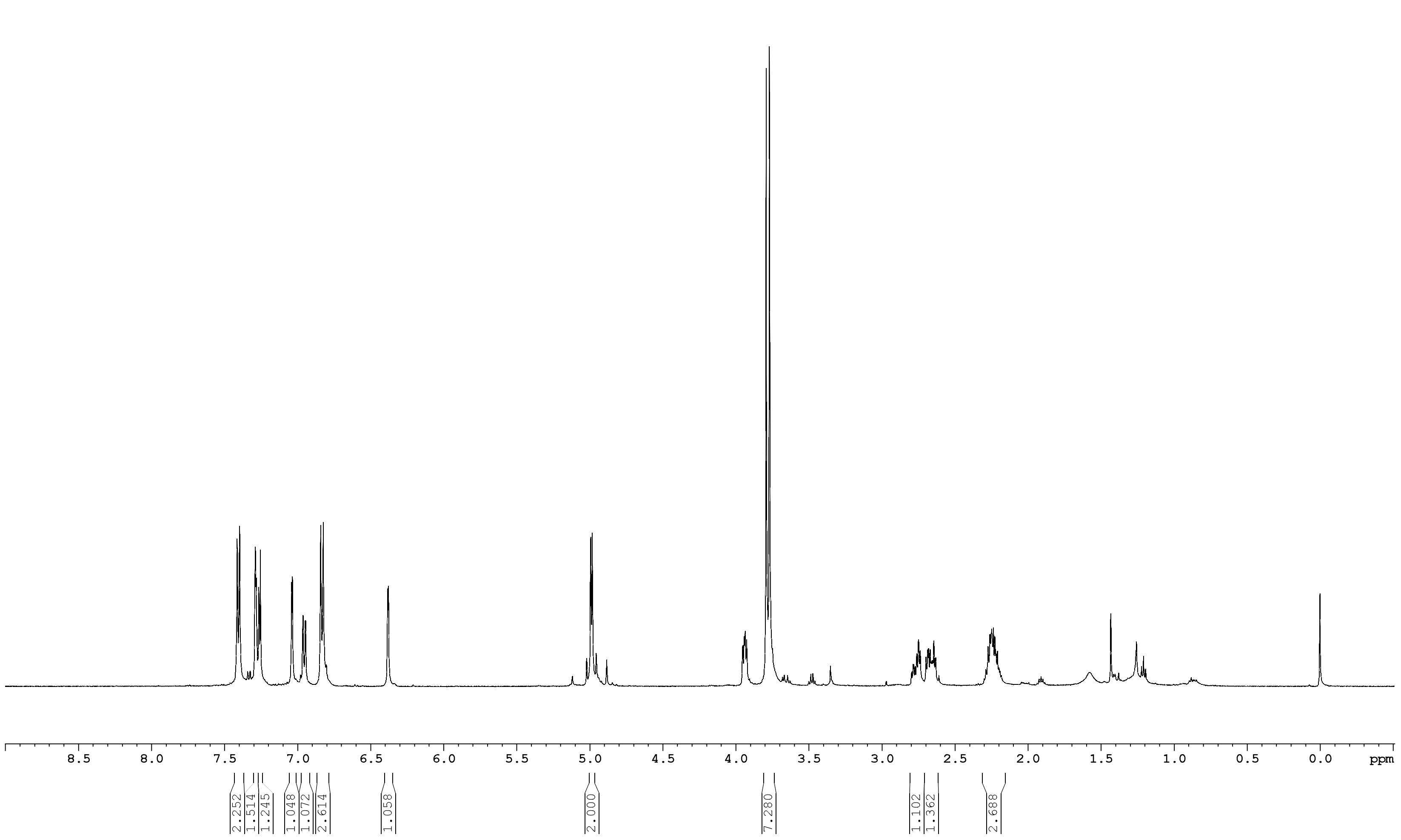


```
Current Data Parameters
NAME        LCB-1-169-ge
EXPNO                  1
PROCNO                 1

F2 - Acquisition Parameters
Date_           20241011
Time               10.17
INSTRUM            gn500
PROBHD   5 mm broadband
PULPROG             zg30
TD                 81728
SOLVENT           CDCl3T
NS                     8
DS                     2
SWH             8012.820 Hz
FIDRES          0.098043 Hz
AQ             5.0998273 sec
RG                 724.1
DW                62.400 usec
DE                  6.00 usec
TE                 298.0 K
D1            0.10000000 sec
MCREST                 0 sec
MCWRK         0.01500000 sec

======== CHANNEL f1 ========
NUC1                  1H
P1                 12.00 usec
PL1                -6.00 dB
SFO1         498.4534891 MHz

F2 - Processing parameters
SI                 65536
SF           498.4500331 MHz
WDW                   EM
SSB                    0
LB                  0.30 Hz
GB                     0
PC                  1.00
```

13C spectrum with 1H decoupling

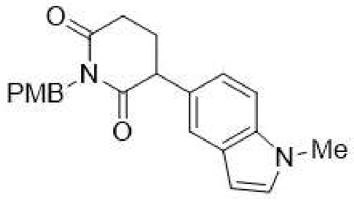

48

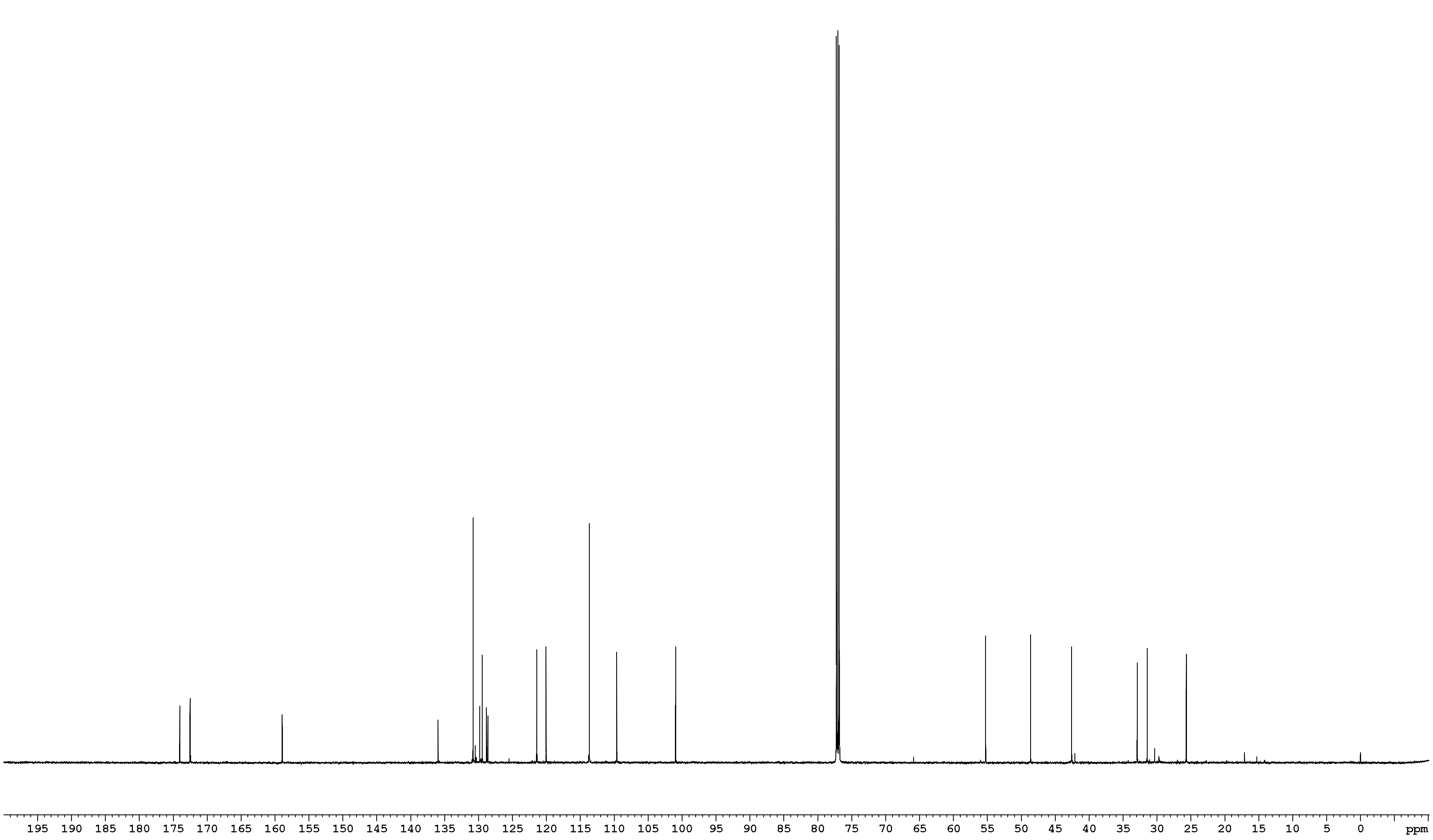


```
Current Data Parameters
NAME     LCB-1-169-600C
EXPNO                 1
PROCNO                1

F2 - Acquisition Parameters
Date_          20241011
Time              11.49
INSTRUM           av600
PROBHD   5 mm CPBBO BB-
PULPROG          zgdc30
TD                65536
SOLVENT          CDCl3T
NS                  596
DS                    4
SWH           36231.883 Hz
FIDRES         0.552855 Hz
AQ            0.9043968 sec
RG                 2050
DW               13.800 usec
DE                19.65 usec
TE                298.0 K
D1           0.40000001 sec
D11          0.03000000 sec
TD0                   1

======== CHANNEL f1 ========
SFO1        150.9194080 MHz
NUC1                13C
P1                10.00 usec
PLW1        68.40000153 W

======== CHANNEL f2 ========
SFO2        600.1330010 MHz
NUC2                 1H
CPDPRG[2        waltz16
PCPD2             80.00 usec
PLW2        30.00000000 W
PLW12        0.39811000 W

F2 - Processing parameters
SI                65536
SF          150.9028185 MHz
WDW                  EM
SSB                   0
LB                 1.00 Hz
GB                    0
PC                 1.00
```

1H spectrum

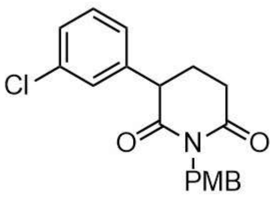

49

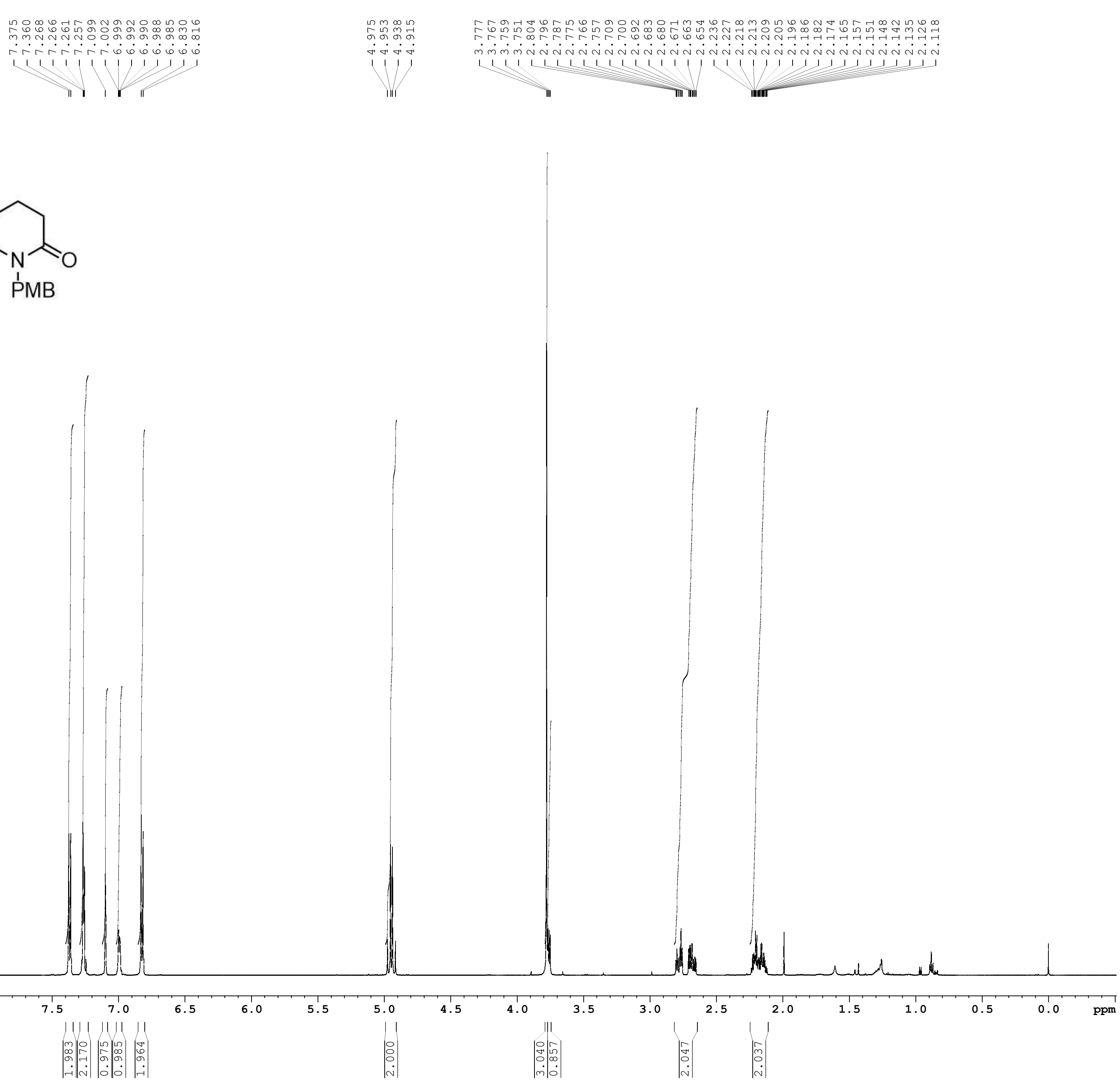

Current Data Parameters
NAME cdw3-256b-p
EXPNO 1
PROCNO 1

F2 - Acquisition Parameters
Date_ 20241109
Time 14.15
INSTRUM av600
PROBHD 5 mm CPBBO BB-
PULPROG zg30
TD 98074
SOLVENT CDCl3
NS 8
DS 2
SWH 9615.385 Hz
FIDRES 0.098042 Hz
AQ 5.0998478 sec
RG 101
DW 52.000 usec
DE 53.12 usec
TE 298.0 K
D1 0.10000000 sec
TD0 1

======== CHANNEL f1 ========
SFO1 600.1342009 MHz
NUC1 1H
P1 10.00 usec
PLW1 30.00000000 W

F2 - Processing parameters
SI 65536
SF 600.1300369 MHz
WDW no
SSB 0
LB 0 Hz
GB 0
PC 1.00

13C spectrum with 1H decoupling

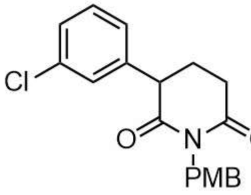

49

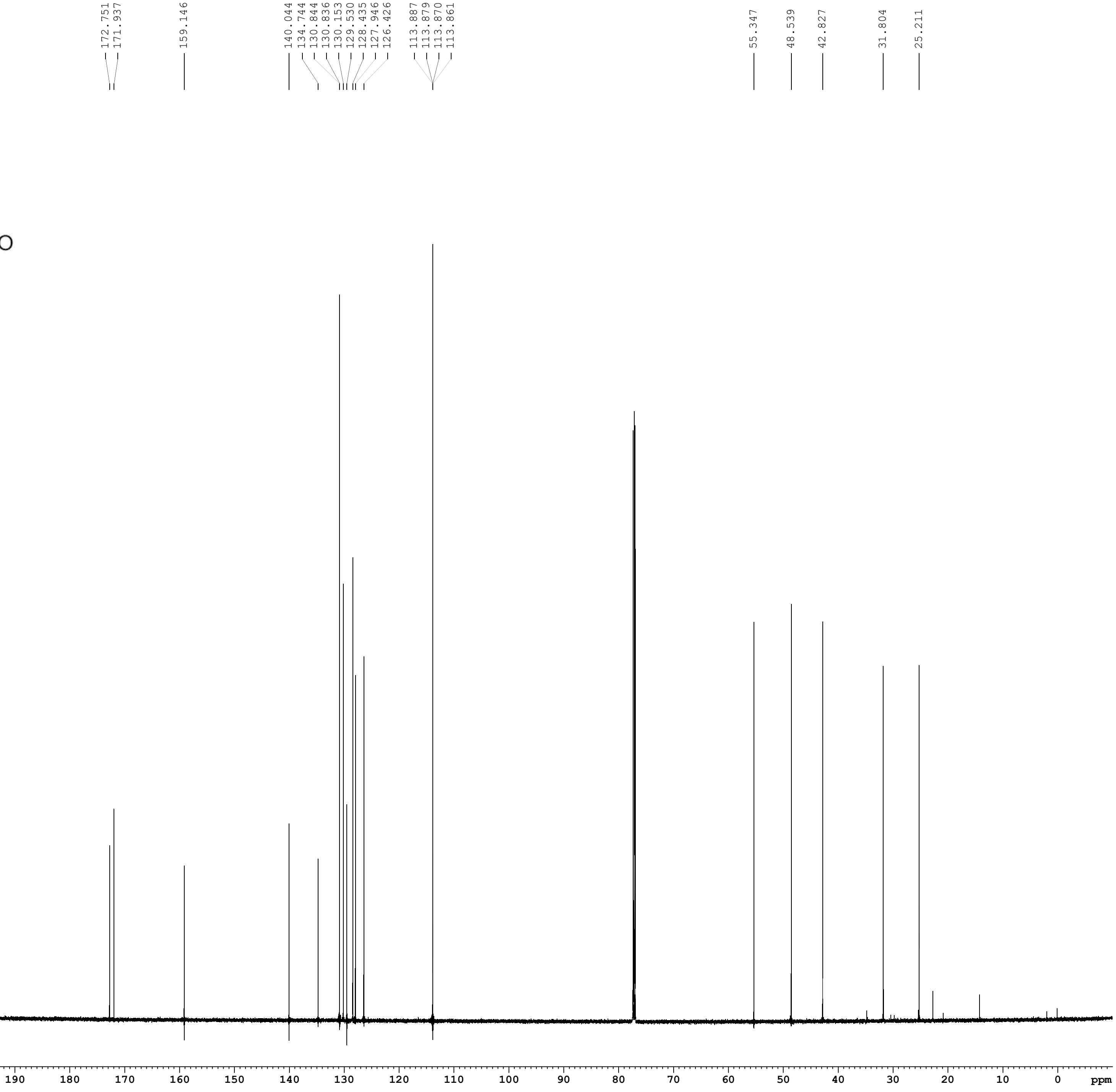

Current Data Parameters
NAME cdw3-256b-c13
EXPNO 1
PROCNO 1

F2 - Acquisition Parameters
Date_ 20241109
Time 14.25
INSTRUM av600
PROBHD 5 mm CPBBO BB-
PULPROG zgdc30
TD 65536
SOLVENT CDCl3
NS 260
DS 4
SWH 36231.883 Hz
FIDRES 0.552855 Hz
AQ 0.9043968 sec
RG 2050
DW 13.800 usec
DE 19.65 usec
TE 298.0 K
D1 0.40000001 sec
D11 0.03000000 sec
TD0 1

======== CHANNEL f1 ========
SFO1 150.9194080 MHz
NUC1 13C
P1 10.00 usec
PLW1 68.40000153 W

======== CHANNEL f2 ========
SFO2 600.1330010 MHz
NUC2 1H
CPDPRG[2 waltz16
PCPD2 80.00 usec
PLW2 30.00000000 W
PLW12 0.39811000 W

F2 - Processing parameters
SI 65536
SF 150.9027996 MHz
WDW no
SSB 0
LB 0 Hz
GB 0
PC 1.00

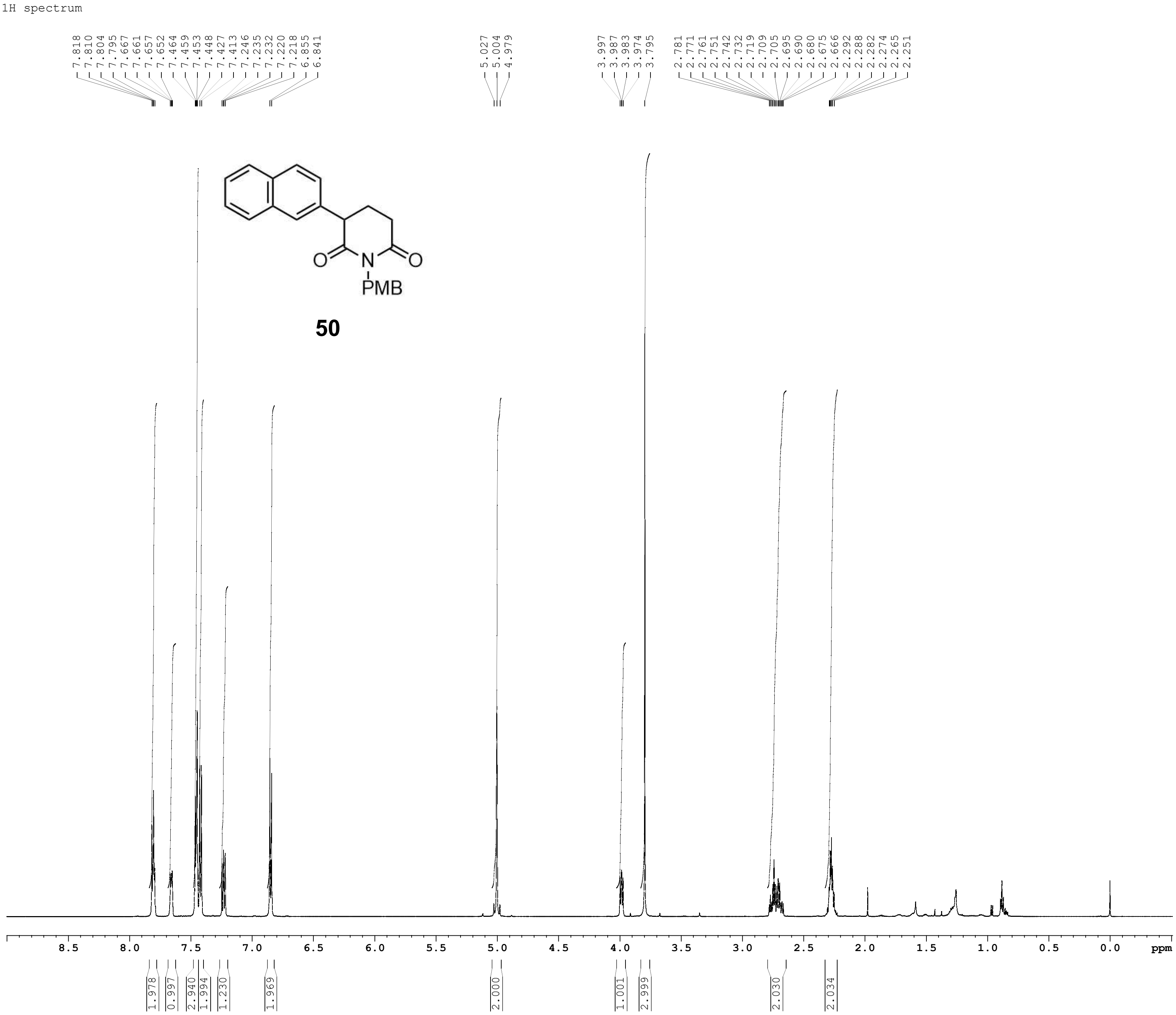


```
Current Data Parameters
NAME        cdw3-256a-p
EXPNO                 1
PROCNO                1

F2 - Acquisition Parameters
Date_          20241109
Time              13.57
INSTRUM           av600
PROBHD   5 mm CPBBO BB-
PULPROG            zg30
TD                98074
SOLVENT           CDCl3
NS                    8
DS                    2
SWH            9615.385 Hz
FIDRES         0.098042 Hz
AQ            5.0998478 sec
RG                  114
DW               52.000 usec
DE                53.12 usec
TE                298.0 K
D1           0.10000000 sec
TD0                   1

======== CHANNEL f1 ========
SFO1        600.1342009 MHz
NUC1                 1H
P1                10.00 usec
PLW1        30.00000000 W

F2 - Processing parameters
SI                65536
SF          600.1300428 MHz
WDW                  no
SSB                   0
LB                    0 Hz
GB                    0
PC                 1.00
```

13C spectrum with 1H decoupling

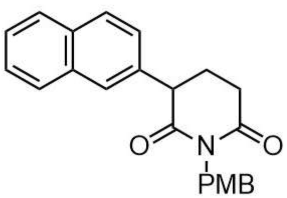

50

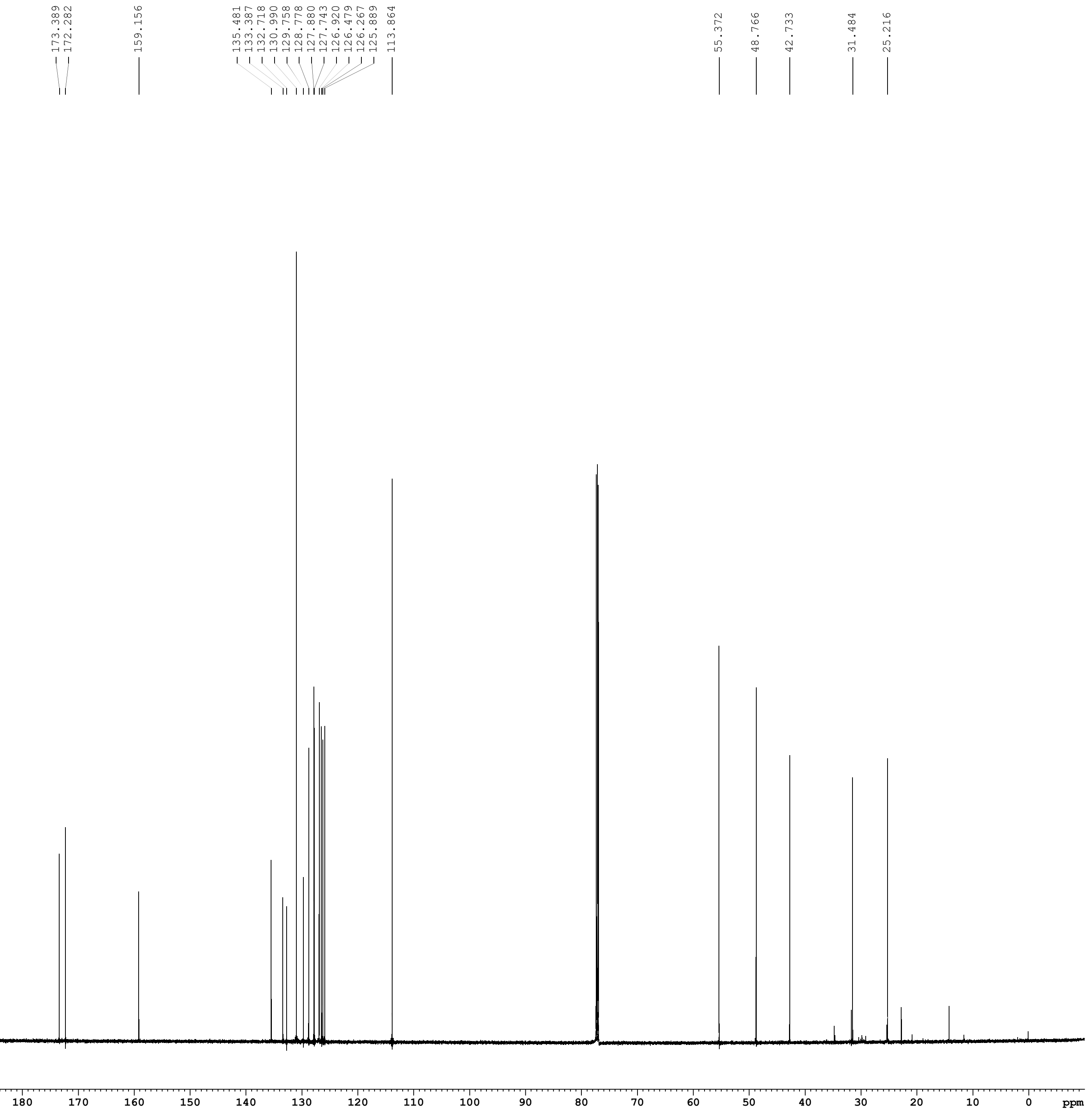


```
Current Data Parameters
NAME        cdw3-256a-c13
EXPNO                   1
PROCNO                  1

F2 - Acquisition Parameters
Date_            20241109
Time                14.09
INSTRUM             av600
PROBHD   5 mm CPBBO BB-
PULPROG            zgdc30
TD                  65536
SOLVENT             CDCl3
NS                    360
DS                      4
SWH             36231.883 Hz
FIDRES           0.552855 Hz
AQ              0.9043968 sec
RG                   2050
DW                 13.800 usec
DE                  19.65 usec
TE                  298.0 K
D1             0.40000001 sec
D11            0.03000000 sec
TD0                     1

======== CHANNEL f1 ========
SFO1         150.9194080 MHz
NUC1                  13C
P1                  10.00 usec
PLW1          68.40000153 W

======== CHANNEL f2 ========
SFO2         600.1330010 MHz
NUC2                   1H
CPDPRG[2            waltz16
PCPD2               80.00 usec
PLW2          30.00000000 W
PLW12          0.39811000 W

F2 - Processing parameters
SI                  65536
SF           150.9028000 MHz
WDW                    no
SSB                     0
LB                      0 Hz
GB                      0
PC                   1.00
```

1H spectrum

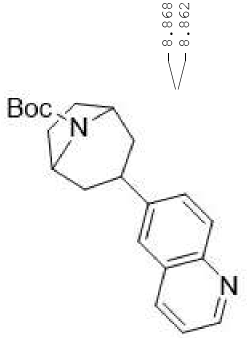

51

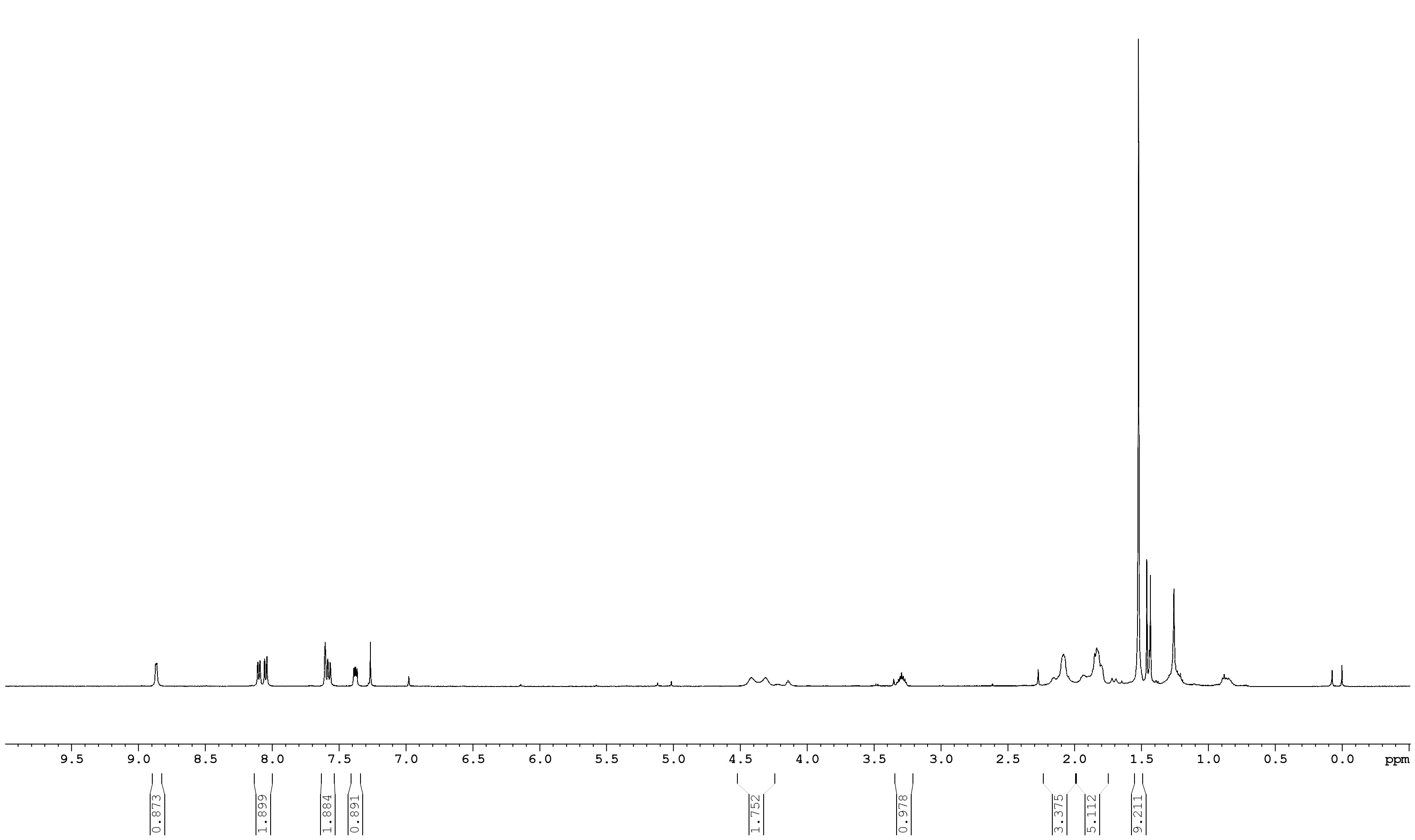

Current Data Parameters
NAME LCB-1-151-fr30-47
EXPNO 1
PROCNO 1

F2 - Acquisition Parameters
Date_ 20240926
Time 10.16
INSTRUM gn500
PROBHD 5 mm broadband
PULPROG zg30
TD 81728
SOLVENT CDCl3T
NS 8
DS 2
SWH 8012.820 Hz
FIDRES 0.098043 Hz
AQ 5.0998273 sec
RG 181
DW 62.400 usec
DE 6.00 usec
TE 298.0 K
D1 0.10000000 sec
MCREST 0 sec
MCWRK 0.01500000 sec

======== CHANNEL f1 ========
NUC1 1H
P1 12.00 usec
PL1 -6.00 dB
SFO1 498.4534891 MHz

F2 - Processing parameters
SI 65536
SF 498.4500276 MHz
WDW EM
SSB 0
LB 0.30 Hz
GB 0
PC 1.00

13C spectrum with 1H decoupling

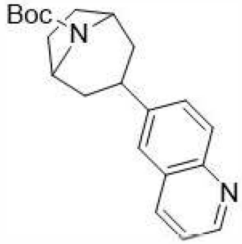

51

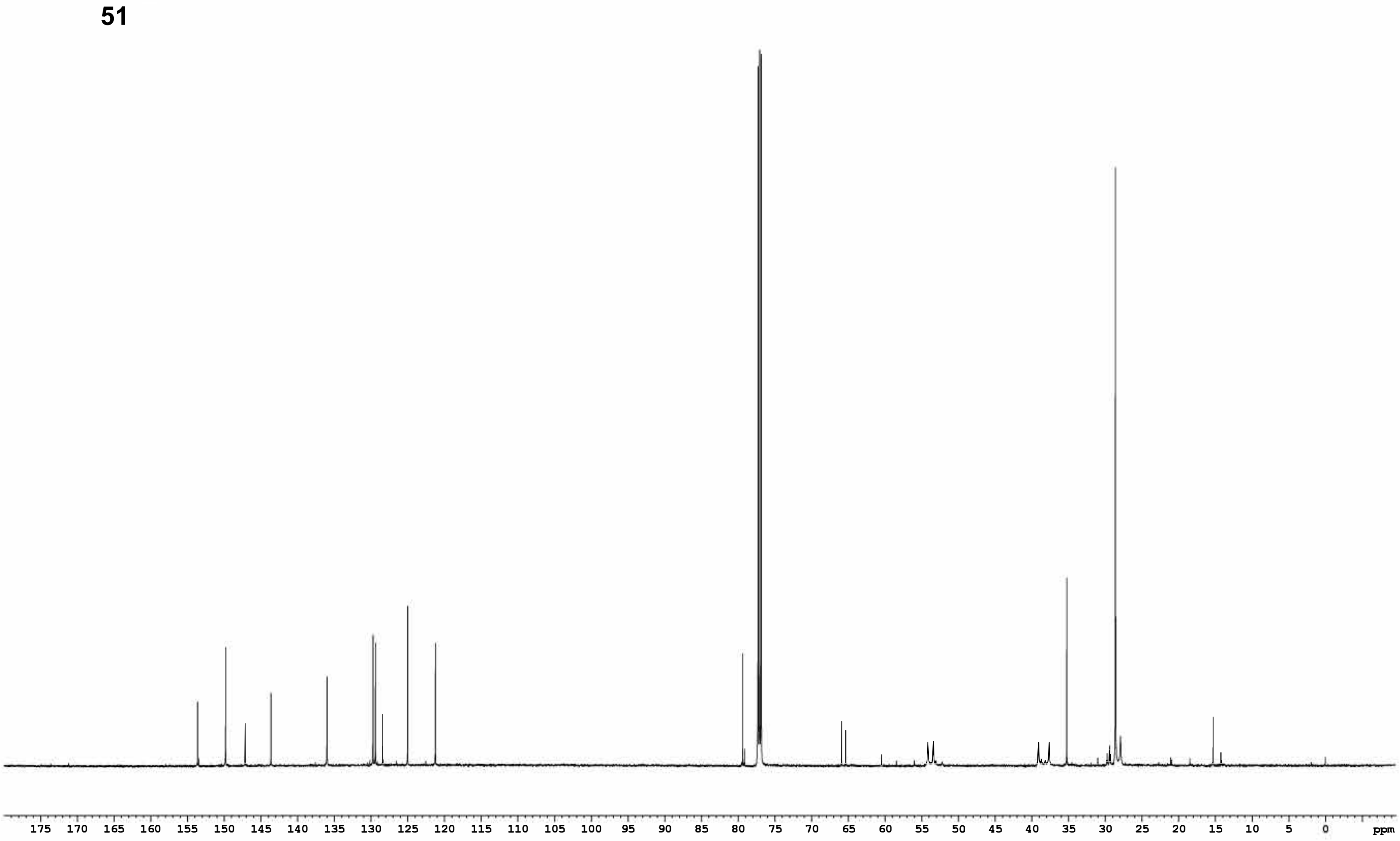


```
Current Data Parameters
NAME     LCB-1-151-600C-2
EXPNO                   1
PROCNO                  1

F2 - Acquisition Parameters
Date_            20241023
Time                 8.05
INSTRUM             av600
PROBHD   5 mm CPBBO BB-
PULPROG           zgdc30
TD                  65536
SOLVENT             CDCl3T
NS                   1024
DS                      4
SWH            36231.883 Hz
FIDRES          0.552855 Hz
AQ             0.9043968 sec
RG                   2050
DW                 13.800 usec
DE                  19.65 usec
TE                  298.2 K
D1             0.40000001 sec
D11            0.03000000 sec
TD0                     1

======== CHANNEL f1 ========
SFO1         150.9194080 MHz
NUC1                  13C
P1                  10.00 usec
PLW1          68.40000153 W

======== CHANNEL f2 ========
SFO2         600.1330010 MHz
NUC2                   1H
CPDPRG[2          waltz16
PCPD2               80.00 usec
PLW2          30.00000000 W
PLW12          0.39811000 W

F2 - Processing parameters
SI                  65536
SF           150.9027369 MHz
WDW                    EM
SSB                     0
LB                   1.00 Hz
GB                      0
PC                   1.00
```

1H spectrum

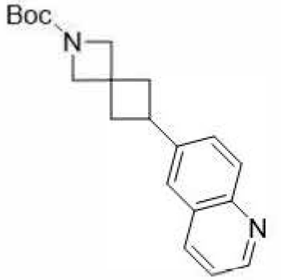

52

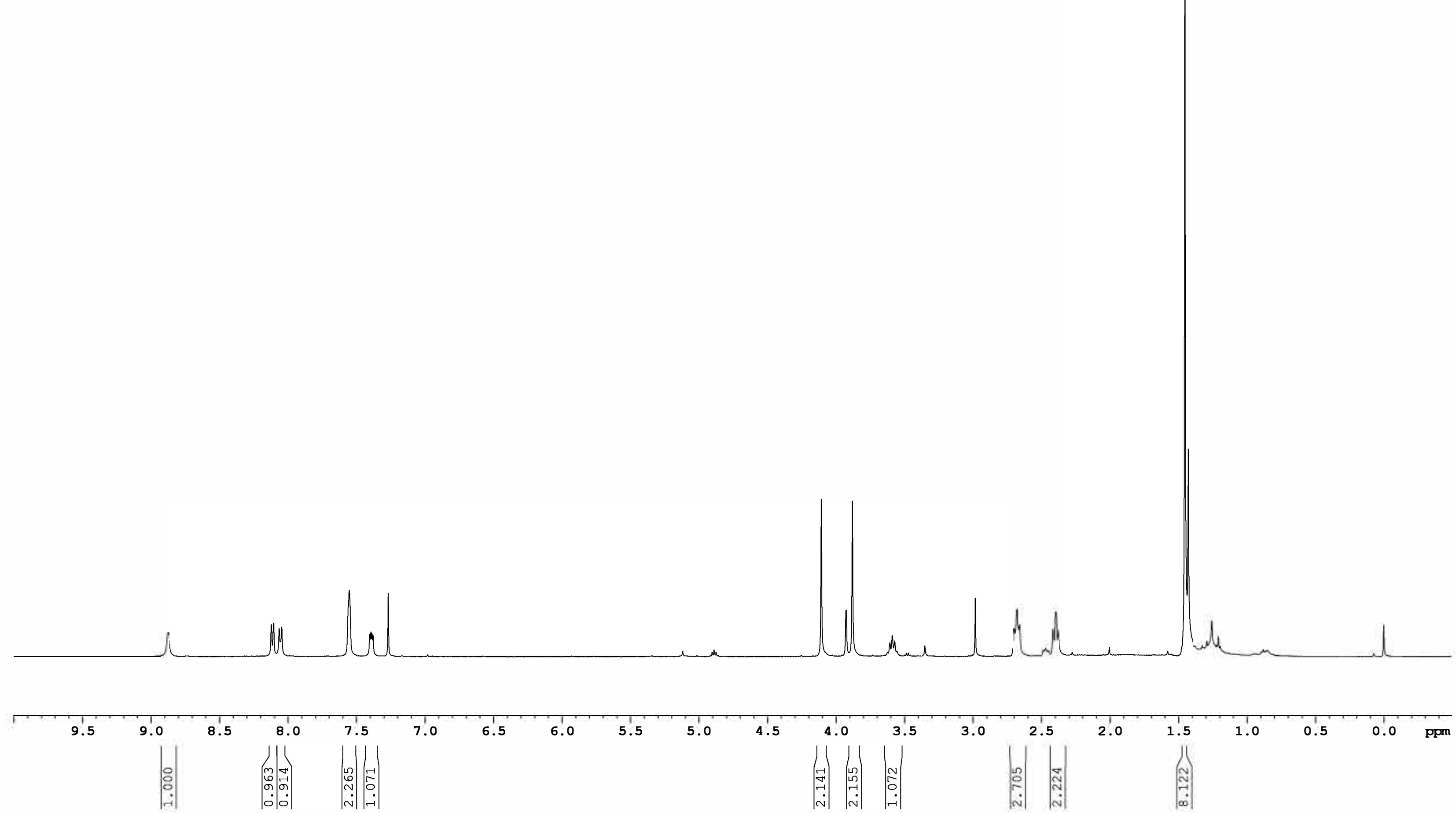

Current Data Parameters
NAME LCB-1-146-ge
EXPNO 1
PROCNO 1

F2 - Acquisition Parameters
Date_ 20240926
Time 10.13
INSTRUM gn500
PROBHD 5 mm broadband
PULPROG zg30
TD 81728
SOLVENT CDCl3T
NS 8
DS 2
SWH 8012.820 Hz
FIDRES 0.098043 Hz
AQ 5.0998273 sec
RG 574.7
DW 62.400 usec
DE 6.00 usec
TE 298.0 K
D1 0.10000000 sec
MCREST 0 sec
MCWRK 0.01500000 sec

======== CHANNEL f1 ========
NUC1 1H
P1 12.00 usec
PL1 -6.00 dB
SFO1 498.4534891 MHz

F2 - Processing parameters
SI 65536
SF 498.4500273 MHz
WDW EM
SSB 0
LB 0.30 Hz
GB 0
PC 1.00

13C spectrum with 1H decoupling

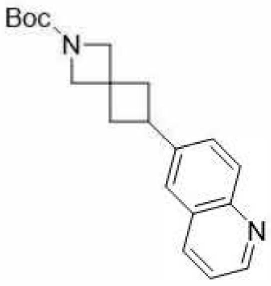

52

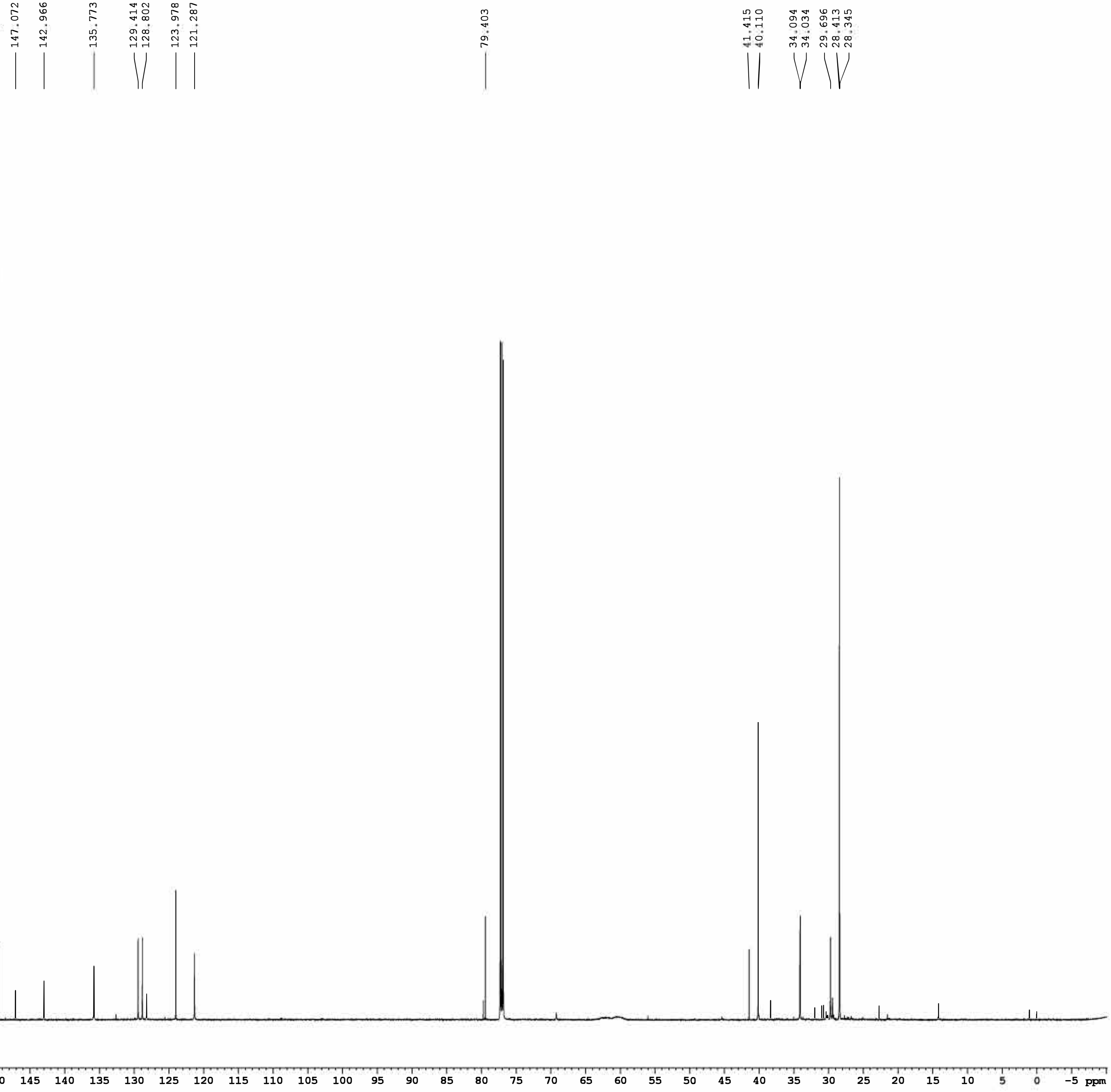


```
Current Data Parameters
NAME      LCB-1-146-600C
EXPNO                  1
PROCNO                 1

F2 - Acquisition Parameters
Date_           20240925
Time               10.08
INSTRUM            av600
PROBHD   5 mm CPBBO BB-
PULPROG          zgdc30
TD                65536
SOLVENT          CDCl3T
NS                  785
DS                    4
SWH           36231.883 Hz
FIDRES         0.552855 Hz
AQ            0.9043968 sec
RG                 2050
DW               13.800 usec
DE                19.65 usec
TE                298.1 K
D1           0.40000001 sec
D11          0.03000000 sec
TD0                   1

======== CHANNEL f1 ========
SFO1        150.9194080 MHz
NUC1                13C
P1                10.00 usec
PLW1        68.40000153 W

======== CHANNEL f2 ========
SFO2        600.1330010 MHz
NUC2                 1H
CPDPRG[2          waltz16
PCPD2             80.00 usec
PLW2        30.00000000 W
PLW12        0.39811000 W

F2 - Processing parameters
SI                65536
SF          150.9028158 MHz
WDW                  EM
SSB                   0
LB                 1.00 Hz
GB                    0
PC                 1.00
```

1H Spectrum

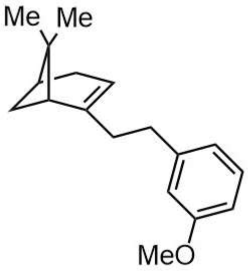

53

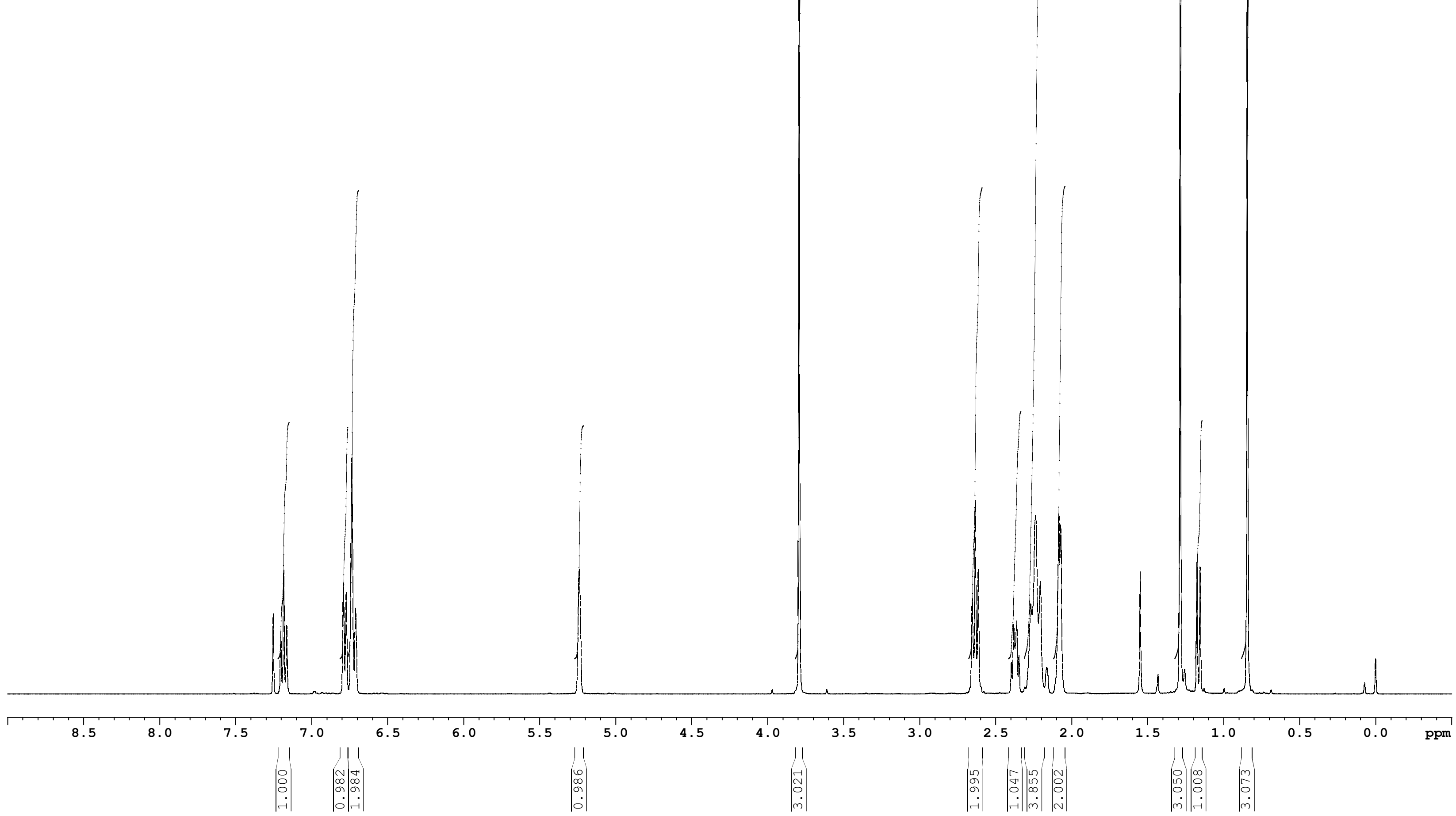


```
Current Data Parameters
NAME       cdw3-258a-p2
EXPNO                 1
PROCNO                1

F2 - Acquisition Parameters
Date_          20241203
Time               8.54 h
INSTRUM           spect
PROBHD   Z149000_0038 (
PULPROG            zg30
TD                65536
SOLVENT           CDCl3
NS                    8
DS                    2
SWH            6402.049 Hz
FIDRES         0.195375 Hz
AQ            5.1183615 sec
RG               118.08
DW               78.100 usec
DE                13.05 usec
TE                298.1 K
D1           0.10000000 sec
TD0                   1
SFO1        400.1328009 MHz
NUC1                 1H
P0                 3.79 usec
P1                11.38 usec
PLW1         7.41450024 W

F2 - Processing parameters
SI                65536
SF          400.1300129 MHz
WDW                  no
SSB                   0
LB                    0 Hz
GB                    0
PC                 1.00
```

1H spectrum

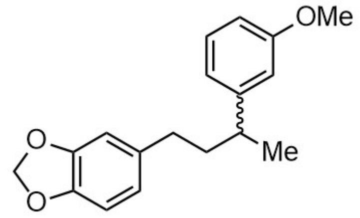

20

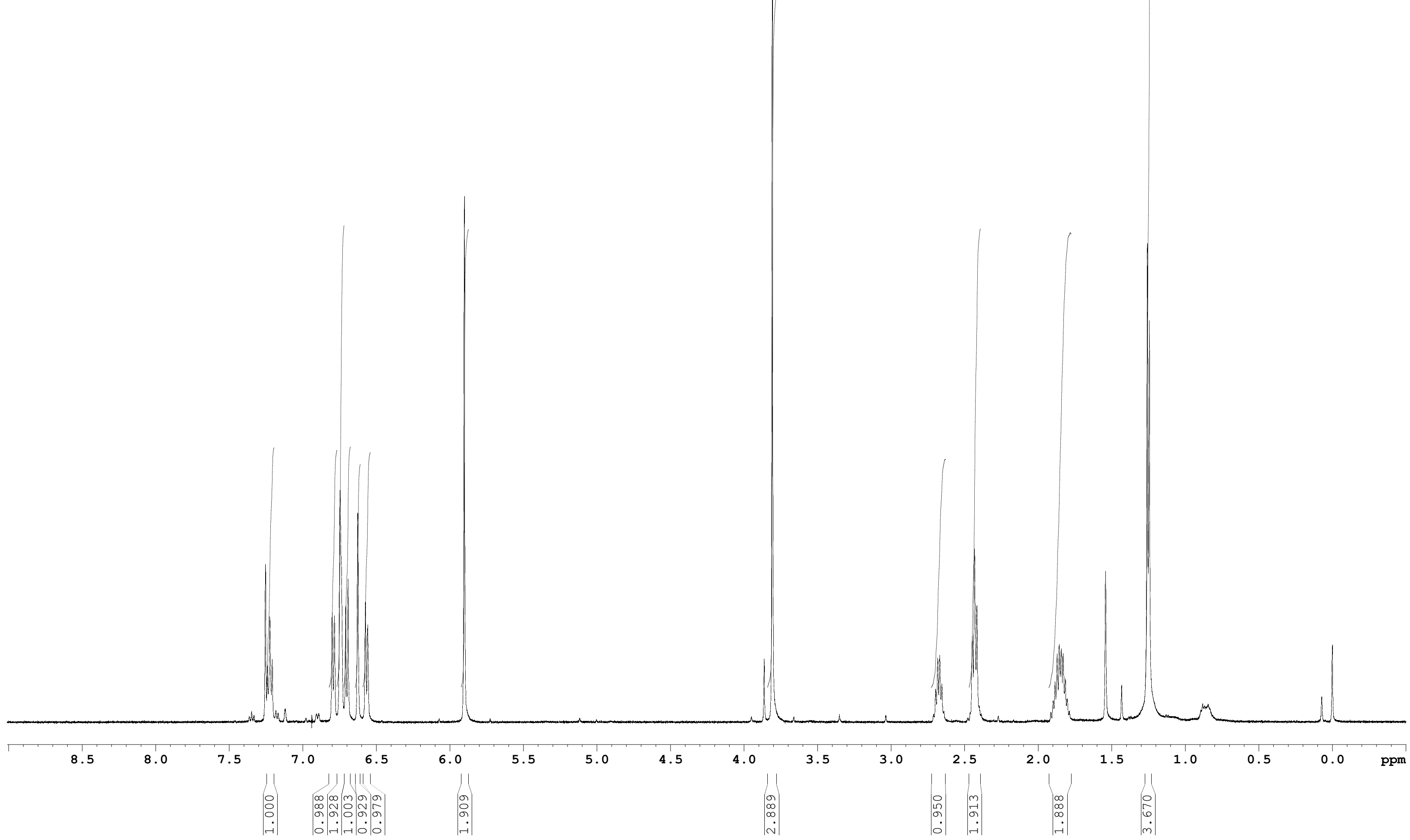

Current Data Parameters
NAME cdw-3-21-test
EXPNO 1
PROCNO 1

F2 - Acquisition Parameters
Date_ 20240402
Time 11.41
INSTRUM gn500
PROBHD 5 mm broadband
PULPROG zg30
TD 81728
SOLVENT CDCl3
NS 8
DS 2
SWH 8012.820 Hz
FIDRES 0.098043 Hz
AQ 5.0998273 sec
RG 812.7
DW 62.400 usec
DE 6.00 usec
TE 298.0 K
D1 0.10000000 sec
MCREST 0 sec
MCWRK 0.01500000 sec

======== CHANNEL f1 ========
NUC1 1H
P1 12.00 usec
PL1 -6.00 dB
SFO1 498.4534891 MHz

F2 - Processing parameters
SI 65536
SF 498.4500311 MHz
WDW no
SSB 0
LB 0 Hz
GB 0
PC 1.00

1H spectrum

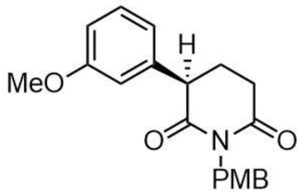

(–)-10

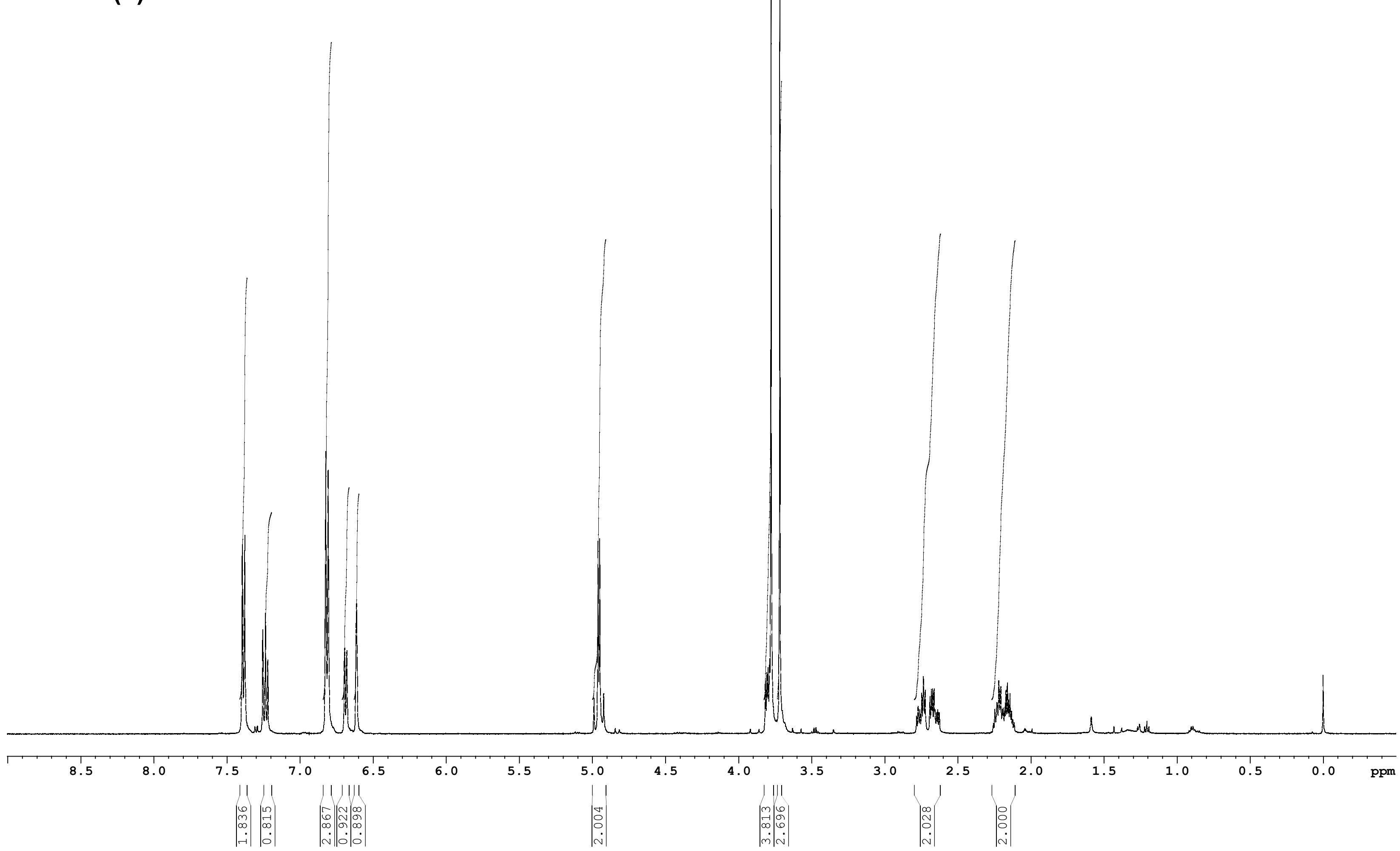

Current Data Parameters
NAME cdw3-253-p
EXPNO 1
PROCNO 1

F2 - Acquisition Parameters
Date_ 20241107
Time 9.46
INSTRUM gn500
PROBHD 5 mm broadband
PULPROG zg30
TD 81728
SOLVENT CDCl3
NS 8
DS 2
SWH 8012.820 Hz
FIDRES 0.098043 Hz
AQ 5.0998273 sec
RG 181
DW 62.400 usec
DE 6.00 usec
TE 298.0 K
D1 0.10000000 sec
MCREST 0 sec
MCWRK 0.01500000 sec

======== CHANNEL f1 ========
NUC1 1H
P1 12.00 usec
PL1 -6.00 dB
SFO1 498.4534891 MHz

F2 - Processing parameters
SI 65536
SF 498.4500317 MHz
WDW no
SSB 0
LB 0 Hz
GB 0
PC 1.00

1H spectrum

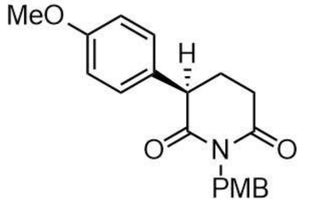

(–)-56

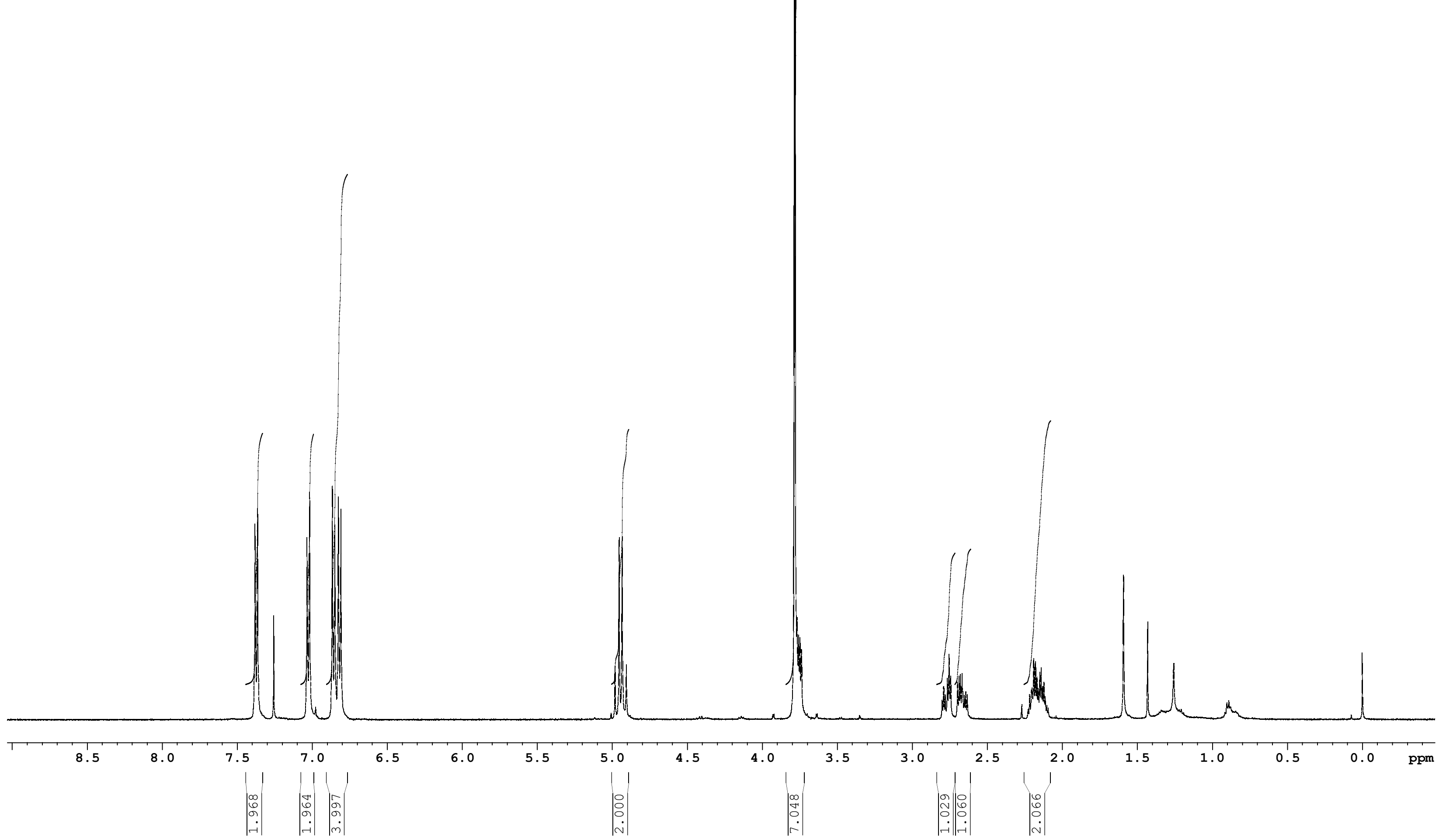

Current Data Parameters
NAME cdw3-226-p
EXPNO 1
PROCNO 1

F2 - Acquisition Parameters
Date_ 20241021
Time 11.03
INSTRUM gn500
PROBHD 5 mm broadband
PULPROG zg30
TD 81728
SOLVENT CDCl3
NS 8
DS 2
SWH 8012.820 Hz
FIDRES 0.098043 Hz
AQ 5.0998273 sec
RG 574.7
DW 62.400 usec
DE 6.00 usec
TE 298.0 K
D1 0.10000000 sec
MCREST 0 sec
MCWRK 0.01500000 sec

======== CHANNEL f1 ========
NUC1 1H
P1 12.00 usec
PL1 -6.00 dB
SFO1 498.4534891 MHz

F2 - Processing parameters
SI 65536
SF 498.4500314 MHz
WDW no
SSB 0
LB 0 Hz
GB 0
PC 1.00

13C spectrum with 1H decoupling

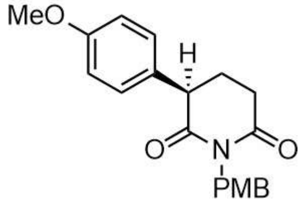

(–)-56

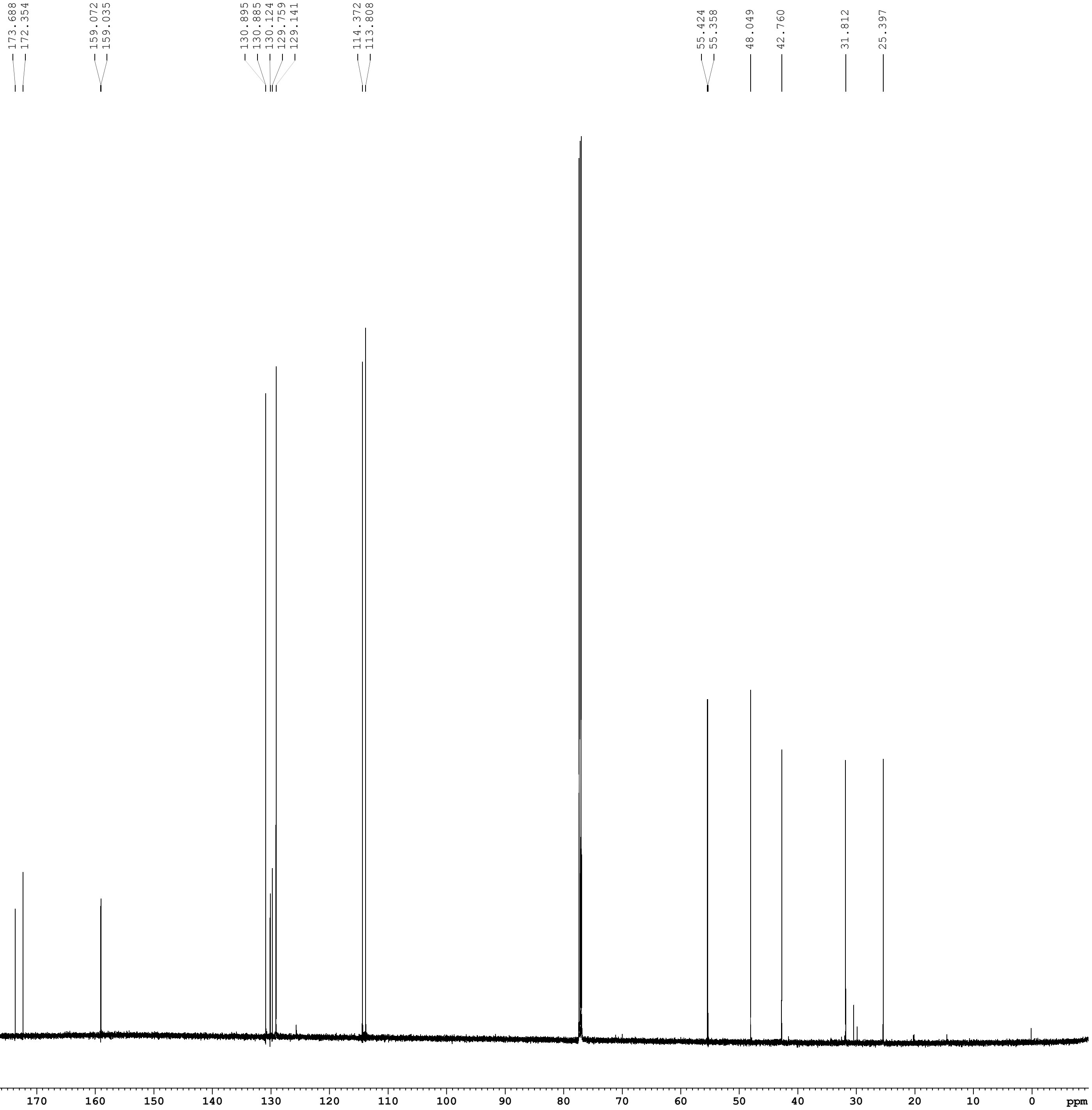


```
Current Data Parameters
NAME       cdw3-226-c13
EXPNO                 1
PROCNO                1

F2 - Acquisition Parameters
Date_          20241021
Time              11.21
INSTRUM           av600
PROBHD   5 mm CPBBO BB-
PULPROG          zgdc30
TD                65536
SOLVENT           CDCl3
NS                  245
DS                    4
SWH          36231.883 Hz
FIDRES        0.552855 Hz
AQ           0.9043968 sec
RG                 2050
DW               13.800 usec
DE                19.65 usec
TE                297.9 K
D1           0.40000001 sec
D11          0.03000000 sec
TD0                   1

======== CHANNEL f1 ========
SFO1        150.9194080 MHz
NUC1                13C
P1                10.00 usec
PLW1        68.40000153 W

======== CHANNEL f2 ========
SFO2        600.1330010 MHz
NUC2                 1H
CPDPRG[2         waltz16
PCPD2             80.00 usec
PLW2        30.00000000 W
PLW12        0.39811000 W

F2 - Processing parameters
SI                65536
SF          150.9027971 MHz
WDW                  no
SSB                   0
LB                    0 Hz
GB                    0
PC                 1.00
```

1H spectrum

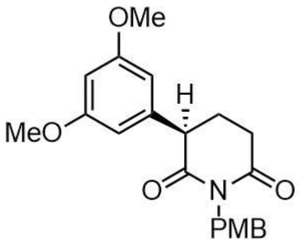

(–)-57

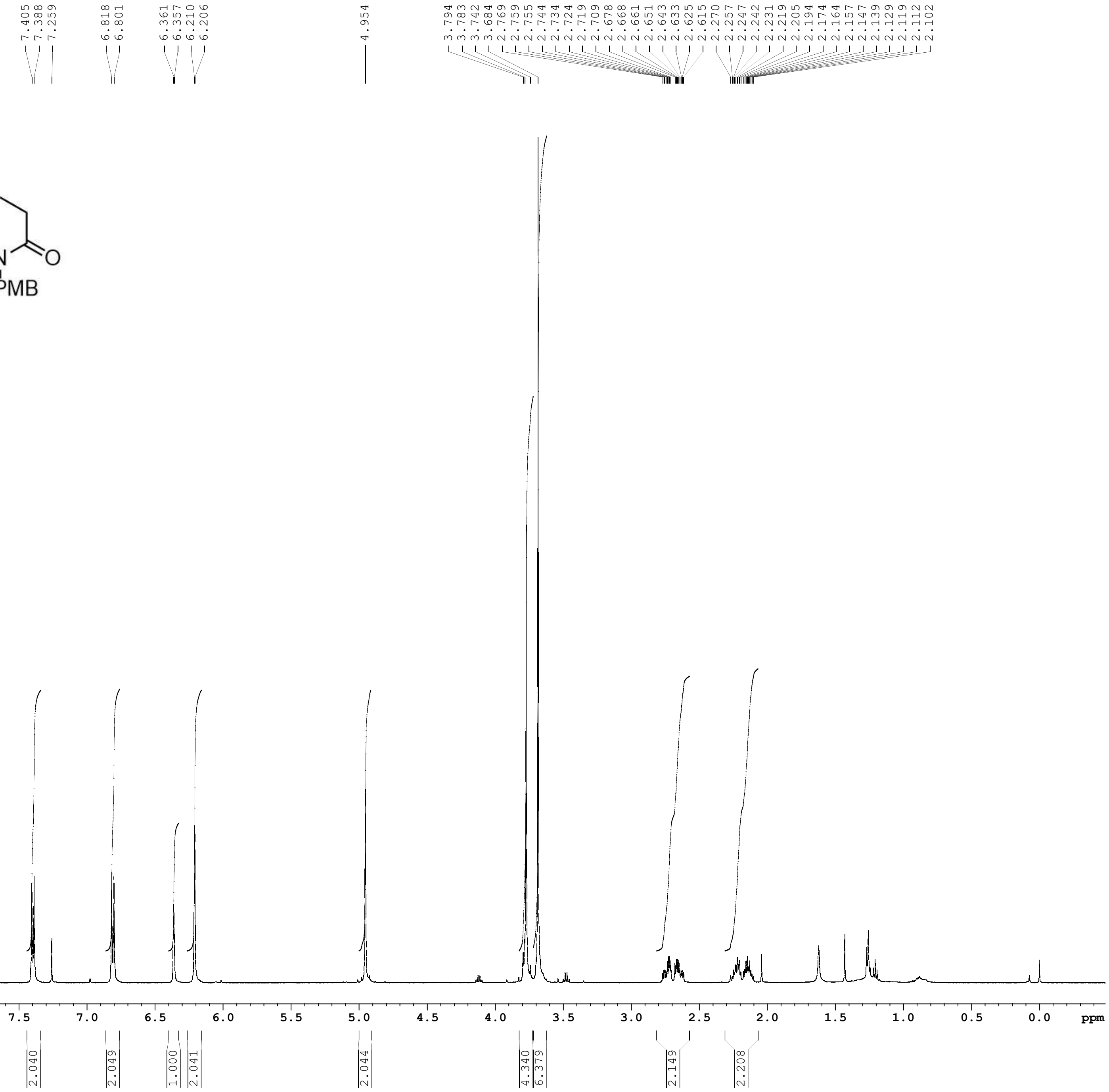


```
Current Data Parameters
NAME          cdw3-236-p
EXPNO                  1
PROCNO                 1

F2 - Acquisition Parameters
Date_           20241024
Time               11.24
INSTRUM            gn500
PROBHD   5 mm broadband
PULPROG             zg30
TD                 81728
SOLVENT            CDCl3
NS                     8
DS                     2
SWH             8012.820 Hz
FIDRES          0.098043 Hz
AQ             5.0998273 sec
RG                   181
DW                62.400 usec
DE                  6.00 usec
TE                 298.0 K
D1            0.10000000 sec
MCREST                 0 sec
MCWRK         0.01500000 sec

======== CHANNEL f1 ========
NUC1                  1H
P1                 12.00 usec
PL1                -6.00 dB
SFO1         498.4534891 MHz

F2 - Processing parameters
SI                 65536
SF           498.4500301 MHz
WDW                   no
SSB                    0
LB                     0 Hz
GB                     0
PC                  1.00
```

13C spectrum with 1H decoupling

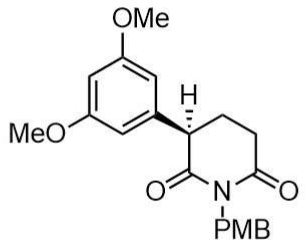

(–)-57

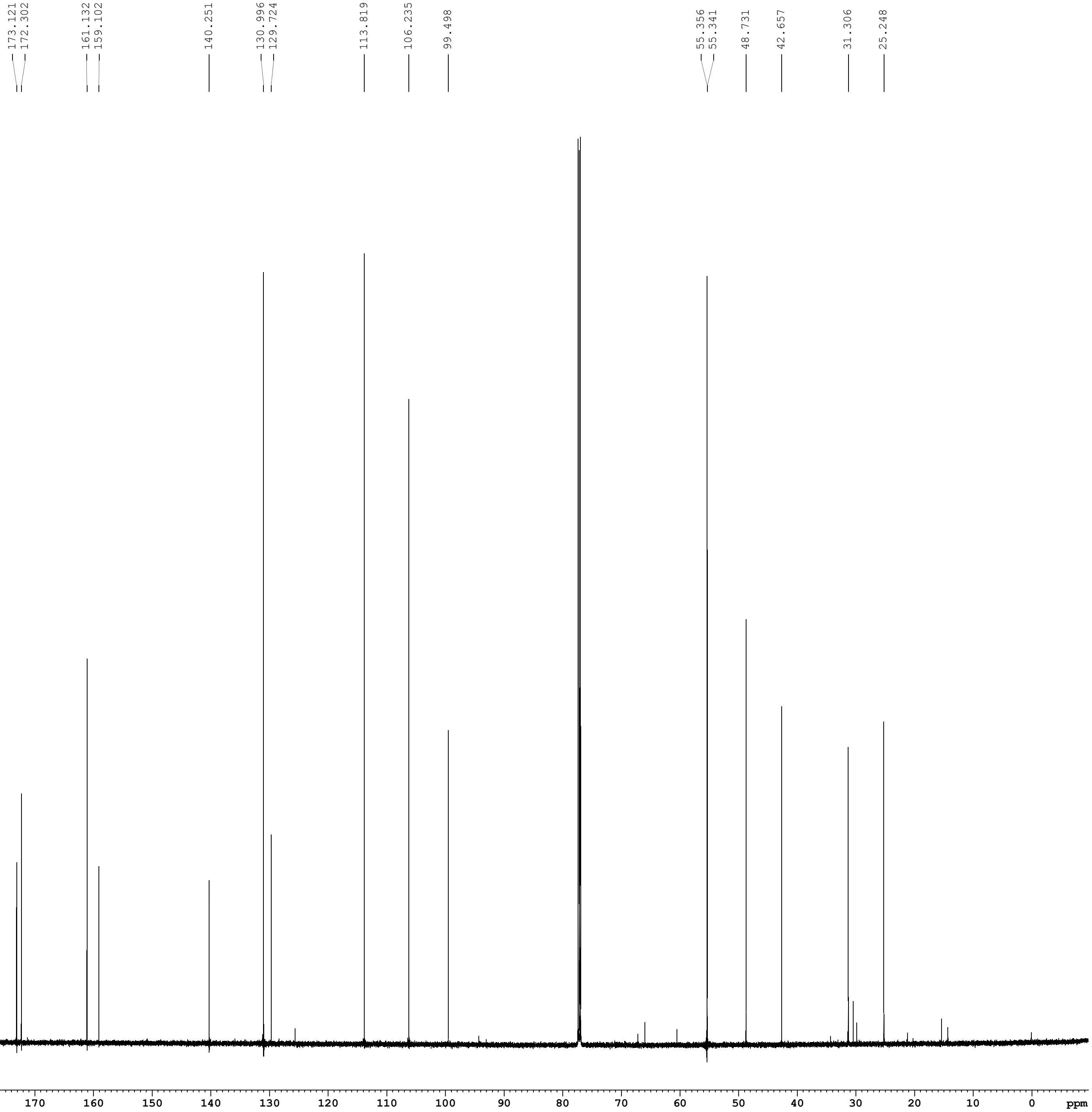


```
Current Data Parameters
NAME       cdw3-236-c13
EXPNO                 1
PROCNO                1

F2 - Acquisition Parameters
Date_          20241024
Time              11.48
INSTRUM           av600
PROBHD   5 mm CPBBO BB-
PULPROG          zgdc30
TD                65536
SOLVENT           CDCl3
NS                  260
DS                    4
SWH          36231.883 Hz
FIDRES        0.552855 Hz
AQ           0.9043968 sec
RG                 2050
DW               13.800 usec
DE                19.65 usec
TE                298.2 K
D1           0.40000001 sec
D11          0.03000000 sec
TD0                   1

======== CHANNEL f1 ========
SFO1        150.9194080 MHz
NUC1                13C
P1                10.00 usec
PLW1        68.40000153 W

======== CHANNEL f2 ========
SFO2        600.1330010 MHz
NUC2                 1H
CPDPRG[2        waltz16
PCPD2             80.00 usec
PLW2        30.00000000 W
PLW12        0.39811000 W

F2 - Processing parameters
SI                65536
SF          150.9027982 MHz
WDW                  no
SSB                   0
LB                    0 Hz
GB                    0
PC                 1.00
```

1H spectrum

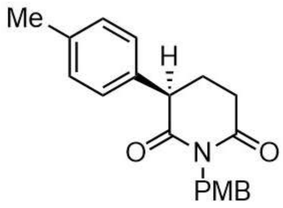

(–)-58

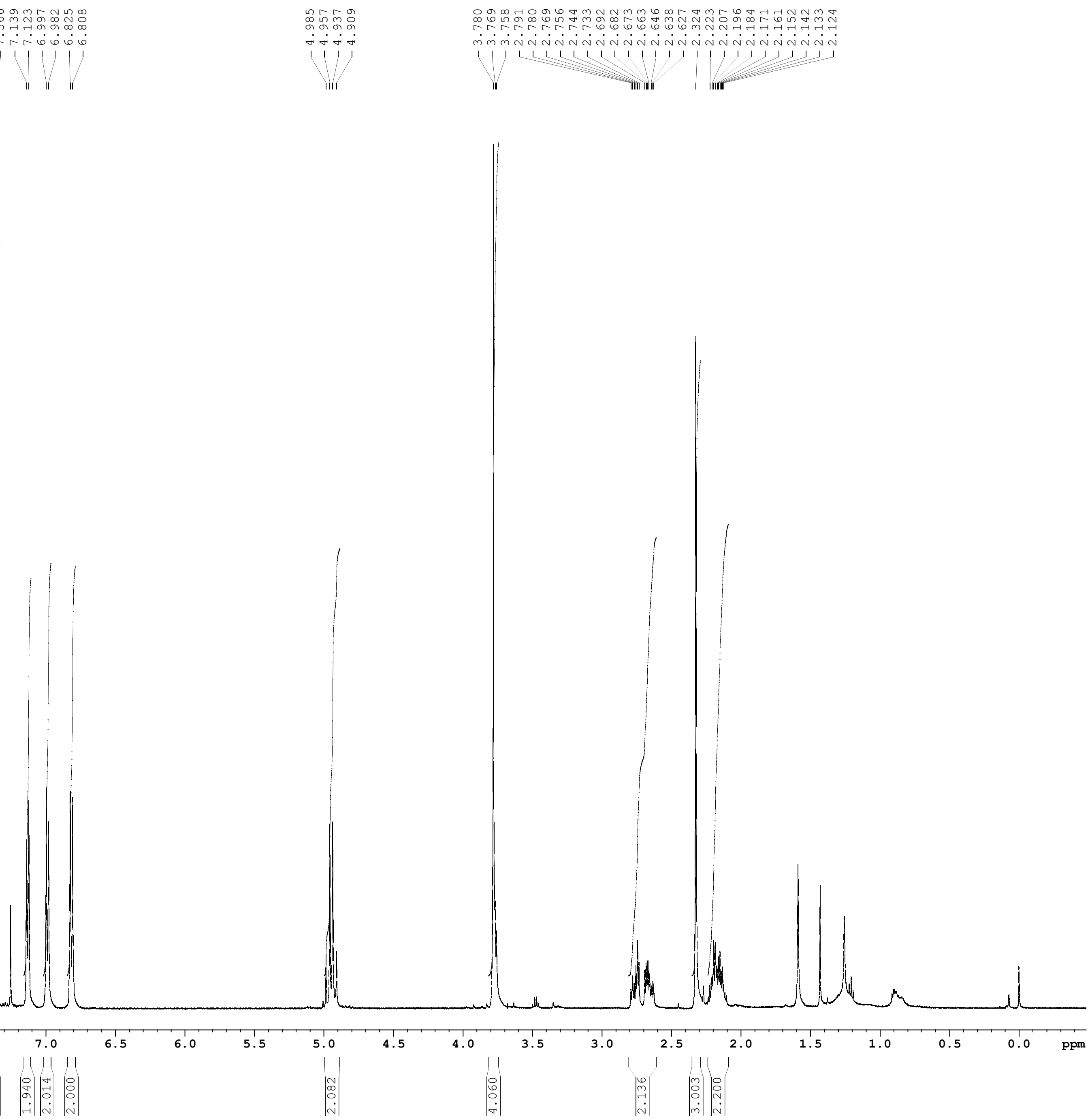

Current Data Parameters
NAME cdw3-239p
EXPNO 1
PROCNO 1

F2 - Acquisition Parameters
Date_ 20241026
Time 11.13
INSTRUM gn500
PROBHD 5 mm broadband
PULPROG zg30
TD 81728
SOLVENT CDCl3
NS 8
DS 2
SWH 8012.820 Hz
FIDRES 0.098043 Hz
AQ 5.0998273 sec
RG 574.7
DW 62.400 usec
DE 6.00 usec
TE 298.0 K
D1 0.10000000 sec
MCREST 0 sec
MCWRK 0.01500000 sec

======== CHANNEL f1 ========
NUC1 1H
P1 12.00 usec
PL1 -6.00 dB
SFO1 498.4534891 MHz

F2 - Processing parameters
SI 65536
SF 498.4500327 MHz
WDW no
SSB 0
LB 0 Hz
GB 0
PC 1.00

13C spectrum with 1H decoupling

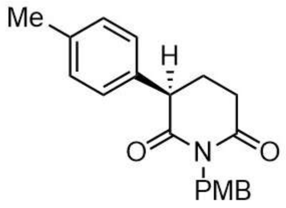

(–)-58

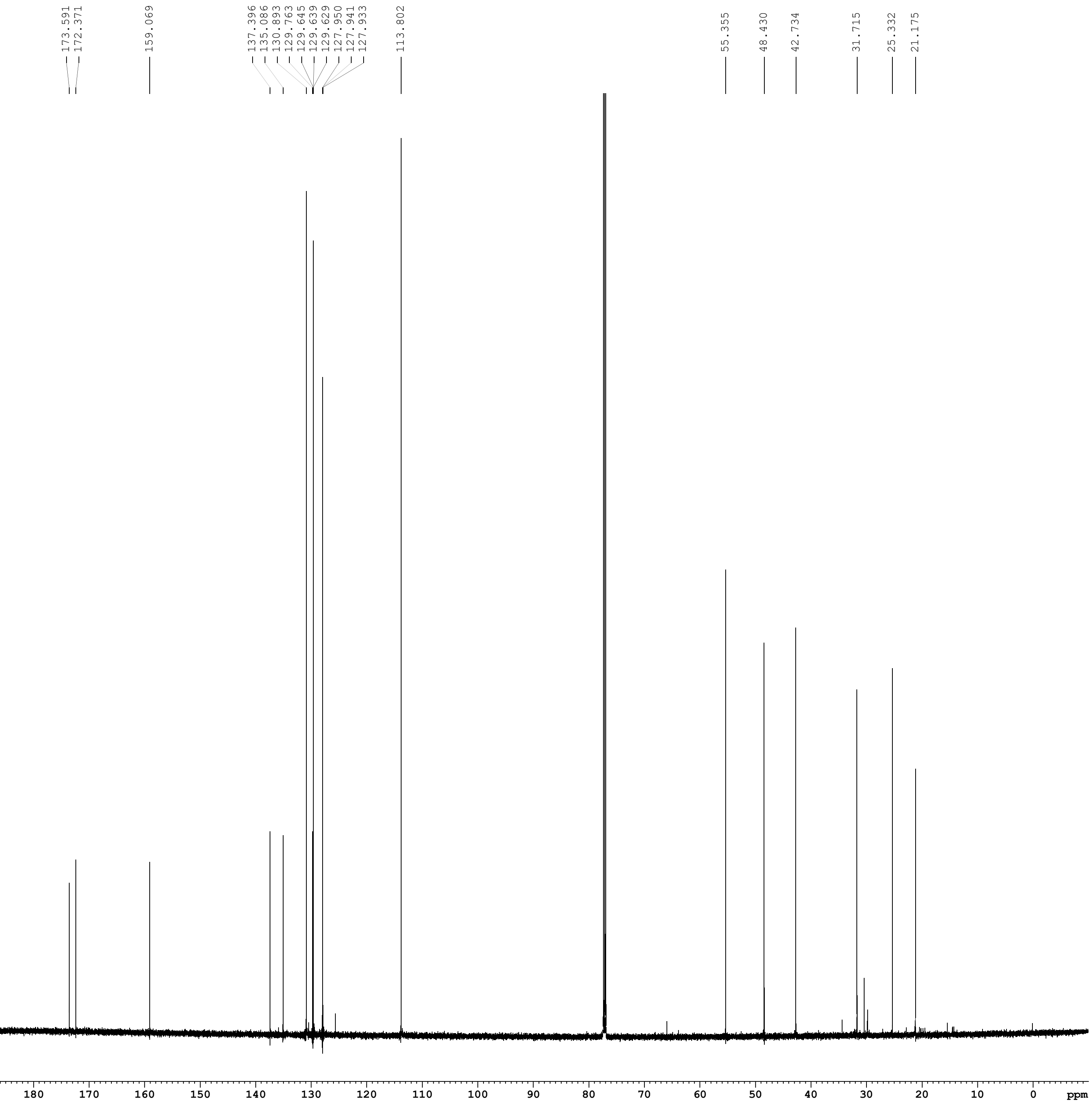


```
Current Data Parameters
NAME        cdw3-239-c13
EXPNO                  1
PROCNO                 1

F2 - Acquisition Parameters
Date_           20241026
Time               11.36
INSTRUM            av600
PROBHD   5 mm CPBBO BB-
PULPROG           zgdc30
TD                 65536
SOLVENT            CDCl3
NS                   400
DS                     4
SWH           36231.883 Hz
FIDRES         0.552855 Hz
AQ            0.9043968 sec
RG                  2050
DW                13.800 usec
DE                 19.65 usec
TE                 298.2 K
D1            0.40000001 sec
D11           0.03000000 sec
TD0                    1

======== CHANNEL f1 ========
SFO1        150.9194080 MHz
NUC1                 13C
P1                 10.00 usec
PLW1         68.40000153 W

======== CHANNEL f2 ========
SFO2        600.1330010 MHz
NUC2                  1H
CPDPRG[2          waltz16
PCPD2              80.00 usec
PLW2         30.00000000 W
PLW12         0.39811000 W

F2 - Processing parameters
SI                 65536
SF          150.9027976 MHz
WDW                   no
SSB                    0
LB                     0 Hz
GB                     0
PC                  1.00
```

1H spectrum

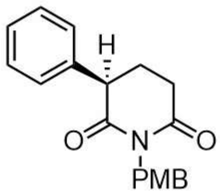

(–)-59

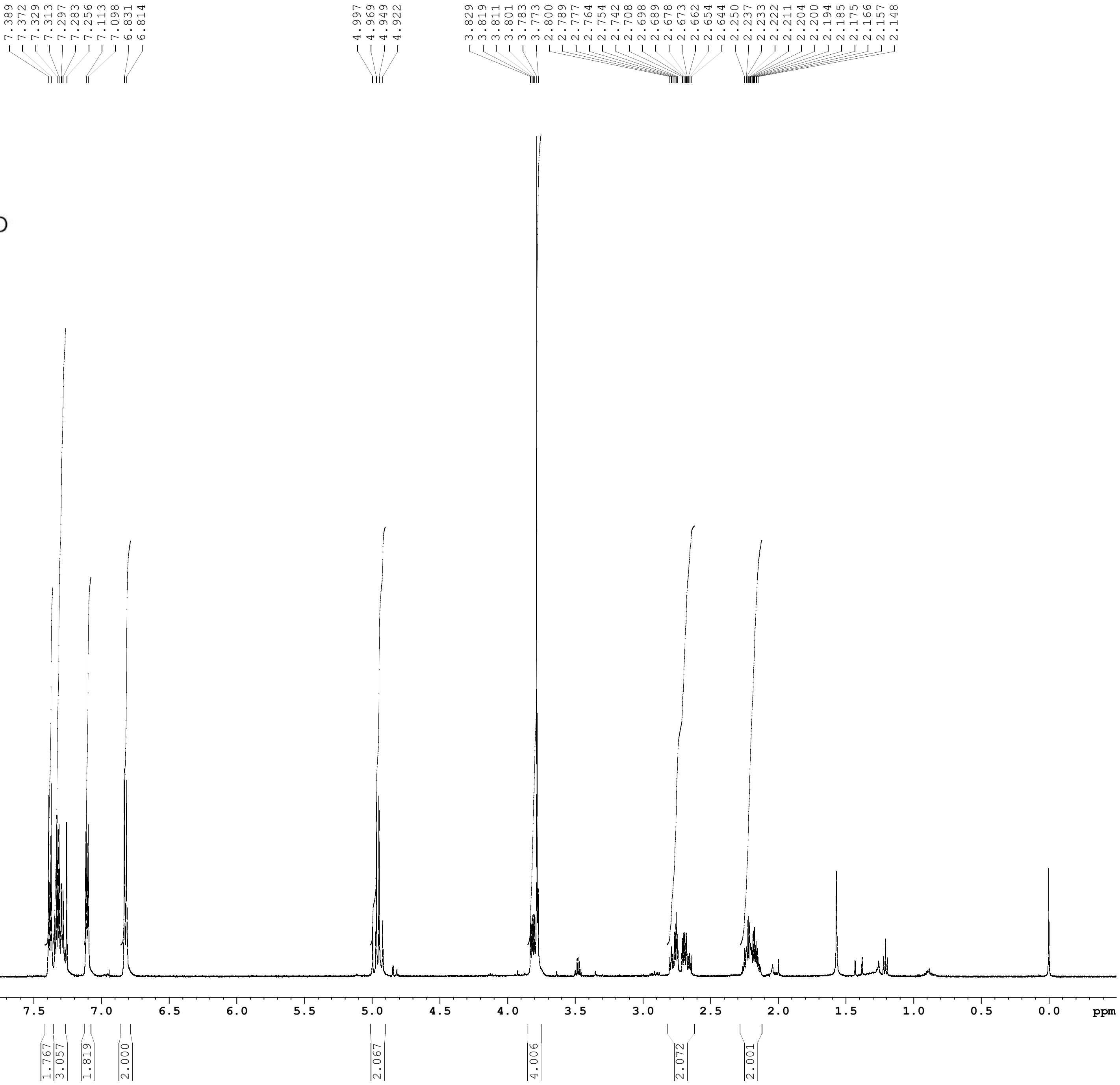


```
Current Data Parameters
NAME        cdw3-240-ge
EXPNO                 1
PROCNO                1

F2 - Acquisition Parameters
Date_          20241030
Time               8.36
INSTRUM           gn500
PROBHD   5 mm broadband
PULPROG            zg30
TD                81728
SOLVENT           CDCl3
NS                    8
DS                    2
SWH            8012.820 Hz
FIDRES         0.098043 Hz
AQ            5.0998273 sec
RG                812.7
DW               62.400 usec
DE                 6.00 usec
TE                298.0 K
D1           0.10000000 sec
MCREST                0 sec
MCWRK        0.01500000 sec

======== CHANNEL f1 ========
NUC1                 1H
P1                12.00 usec
PL1               -6.00 dB
SFO1        498.4534891 MHz

F2 - Processing parameters
SI                65536
SF          498.4500312 MHz
WDW                  no
SSB                   0
LB                    0 Hz
GB                    0
PC                 1.00
```

13C spectrum with 1H decoupling

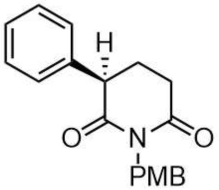

(–)-59

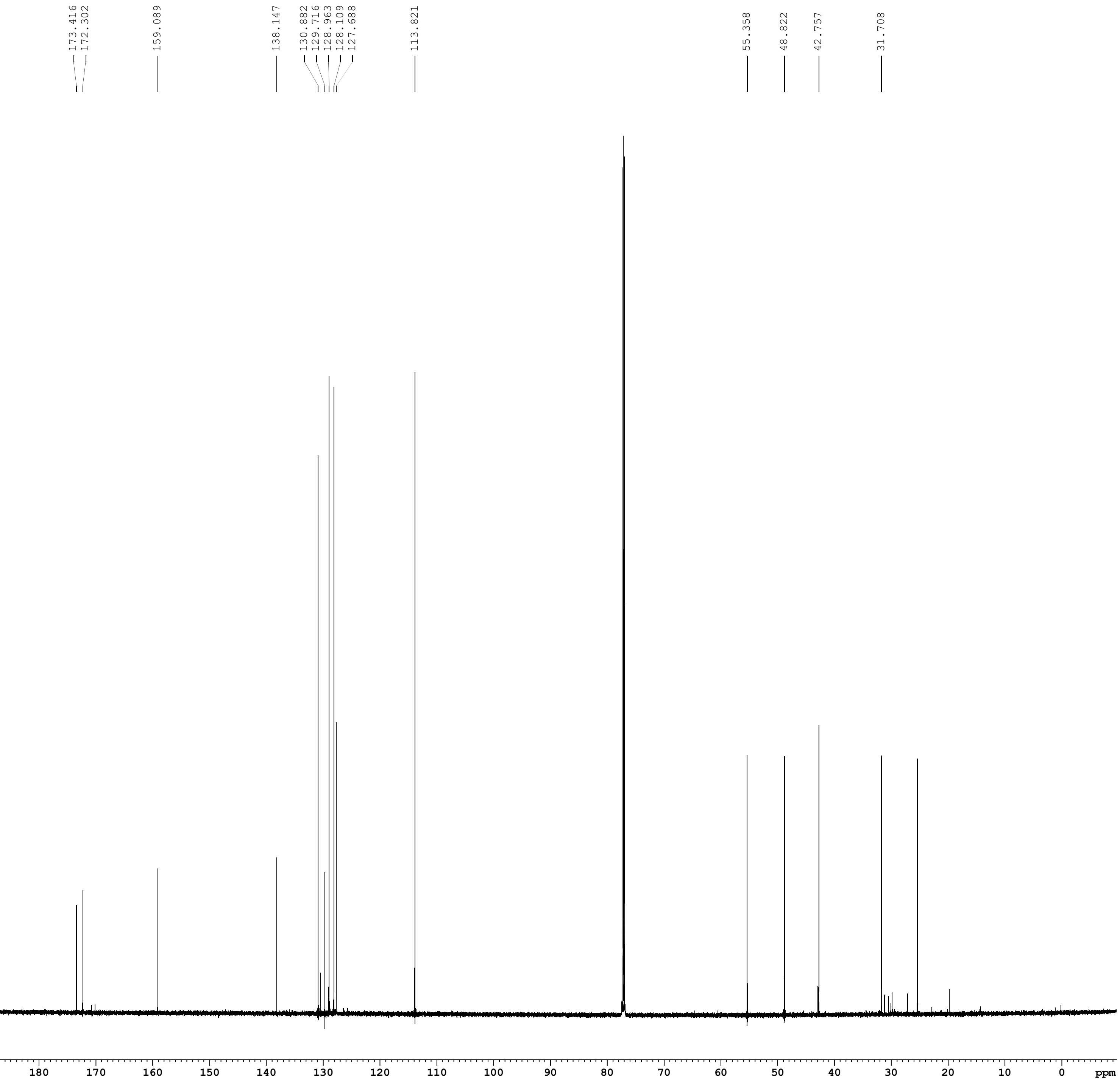


```
Current Data Parameters
NAME       cdw3-240-c13
EXPNO                 1
PROCNO                1

F2 - Acquisition Parameters
Date_          20241029
Time               9.21
INSTRUM           av600
PROBHD   5 mm CPBBO BB-
PULPROG          zgdc30
TD                65536
SOLVENT           CDCl3
NS                  430
DS                    4
SWH           36231.883 Hz
FIDRES         0.552855 Hz
AQ            0.9043968 sec
RG                 2050
DW               13.800 usec
DE                19.65 usec
TE                298.0 K
D1           0.40000001 sec
D11          0.03000000 sec
TD0                   1

======== CHANNEL f1 ========
SFO1        150.9194080 MHz
NUC1                13C
P1                10.00 usec
PLW1        68.40000153 W

======== CHANNEL f2 ========
SFO2        600.1330010 MHz
NUC2                 1H
CPDPRG[2        waltz16
PCPD2             80.00 usec
PLW2        30.00000000 W
PLW12        0.39811000 W

F2 - Processing parameters
SI                65536
SF          150.9027974 MHz
WDW                  no
SSB                   0
LB                    0 Hz
GB                    0
PC                 1.00
```

1H Spectrum

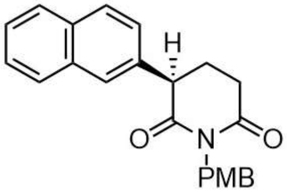

(–)-50

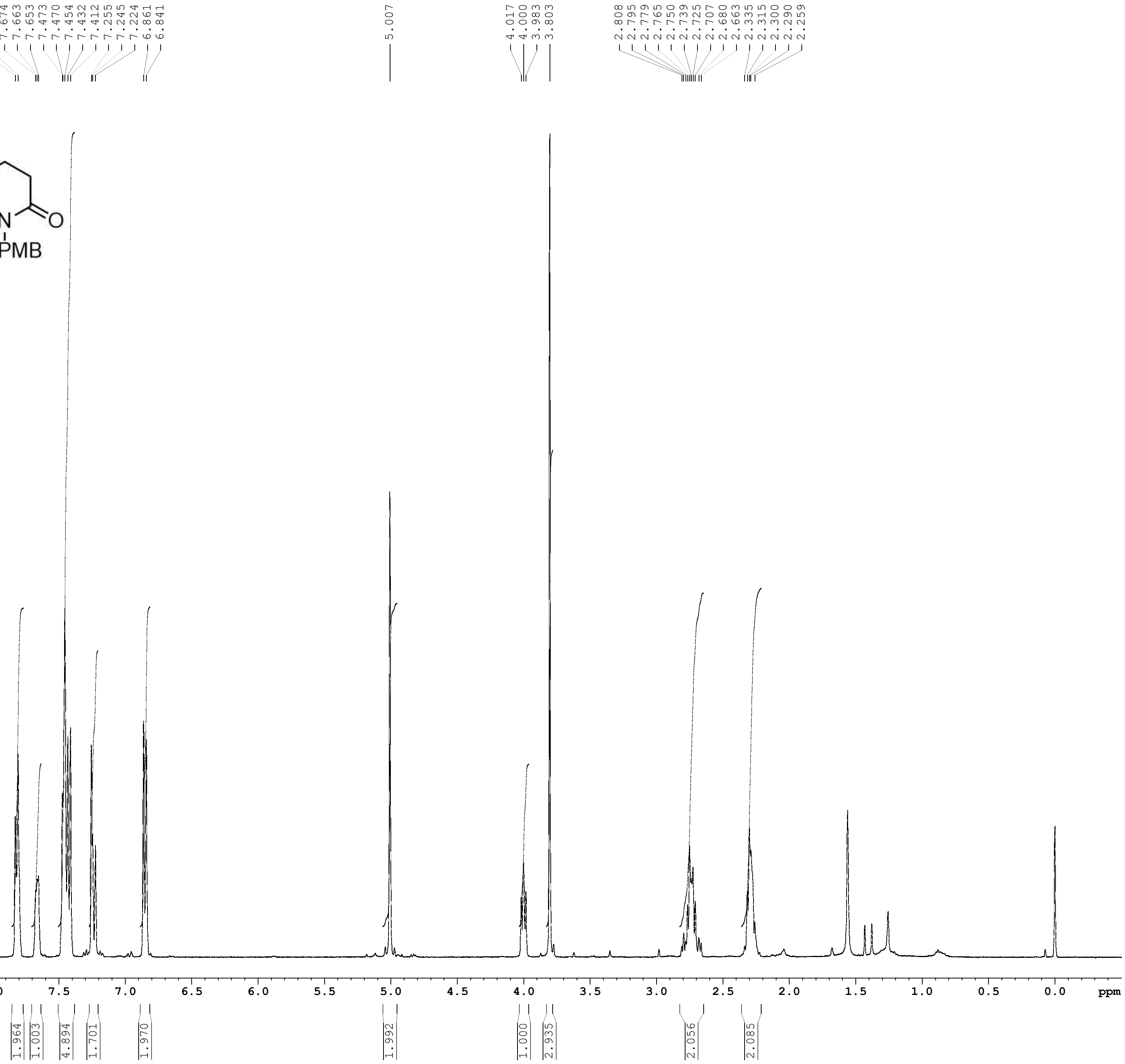

Current Data Parameters
NAME cdw3-254-ge2
EXPNO 1
PROCNO 1

F2 - Acquisition Parameters
Date_ 20241118
Time 9.05 h
INSTRUM spect
PROBHD Z149000_0038 (
PULPROG zg30
TD 65536
SOLVENT CDCl3
NS 8
DS 2
SWH 6402.049 Hz
FIDRES 0.195375 Hz
AQ 5.1183615 sec
RG 207.08
DW 78.100 usec
DE 13.05 usec
TE 298.1 K
D1 0.10000000 sec
TD0 1
SFO1 400.1328009 MHz
NUC1 1H
P0 3.79 usec
P1 11.38 usec
PLW1 7.41450024 W

F2 - Processing parameters
SI 65536
SF 400.1300121 MHz
WDW no
SSB 0
LB 0 Hz
GB 0
PC 1.00

1H spectrum

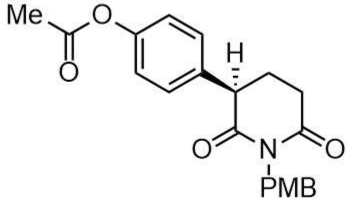

(–)-60

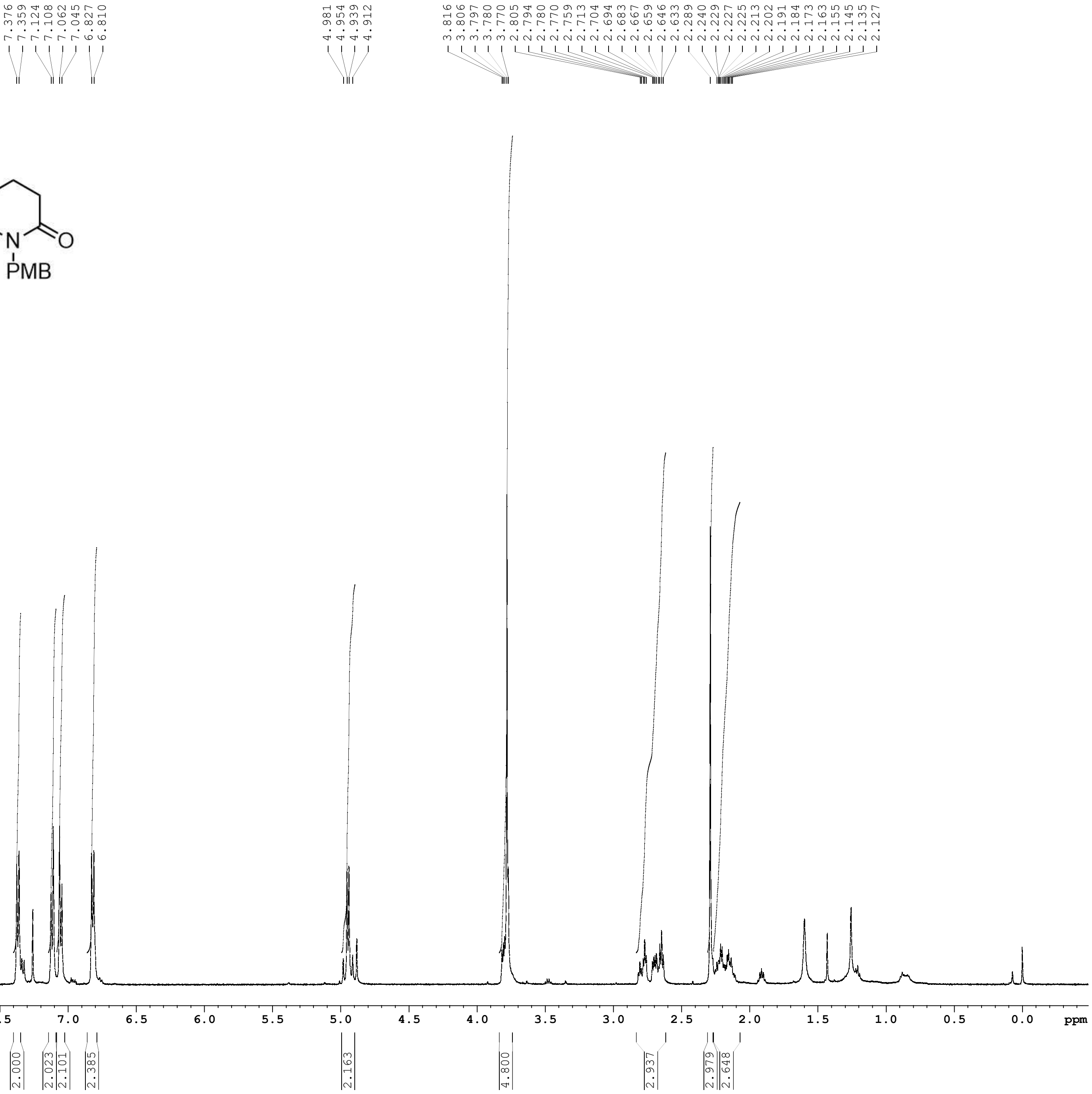


```
Current Data Parameters
NAME          cdw3-241-p
EXPNO                  1
PROCNO                 1

F2 - Acquisition Parameters
Date_           20241025
Time               11.41
INSTRUM            gn500
PROBHD   5 mm broadband
PULPROG             zg30
TD                 81728
SOLVENT            CDCl3
NS                     8
DS                     2
SWH             8012.820 Hz
FIDRES          0.098043 Hz
AQ             5.0998273 sec
RG                 645.1
DW                62.400 usec
DE                  6.00 usec
TE                 298.0 K
D1            0.10000000 sec
MCREST                 0 sec
MCWRK         0.01500000 sec

======== CHANNEL f1 ========
NUC1                  1H
P1                 12.00 usec
PL1                -6.00 dB
SFO1         498.4534891 MHz

F2 - Processing parameters
SI                 65536
SF           498.4500303 MHz
WDW                   no
SSB                    0
LB                     0 Hz
GB                     0
PC                  1.00
```

13C spectrum with 1H decoupling

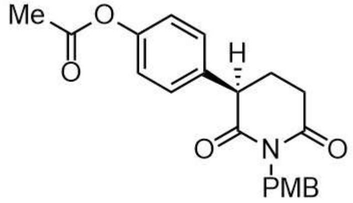

(–)-60

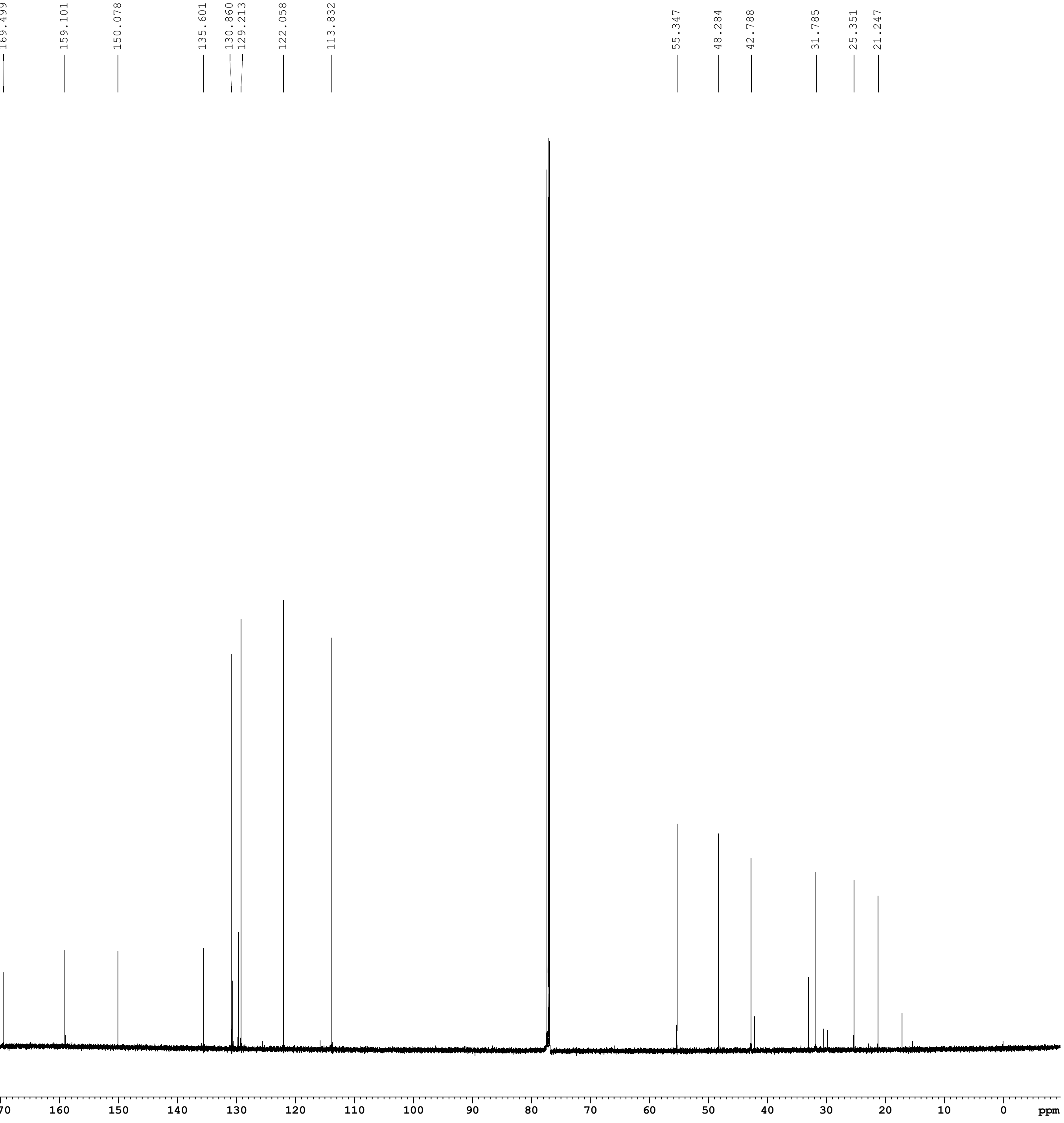


```
Current Data Parameters
NAME        cdw3-241-c13
EXPNO                  1
PROCNO                 1

F2 - Acquisition Parameters
Date_           20241025
Time               12.00
INSTRUM            av600
PROBHD   5 mm CPBBO BB-
PULPROG           zgdc30
TD                 65536
SOLVENT            CDCl3
NS                   250
DS                     4
SWH            36231.883 Hz
FIDRES          0.552855 Hz
AQ             0.9043968 sec
RG                  2050
DW                13.800 usec
DE                 19.65 usec
TE                 298.2 K
D1            0.40000001 sec
D11           0.03000000 sec
TD0                    1

======== CHANNEL f1 ========
SFO1         150.9194080 MHz
NUC1                 13C
P1                 10.00 usec
PLW1         68.40000153 W

======== CHANNEL f2 ========
SFO2         600.1330010 MHz
NUC2                  1H
CPDPRG[2           waltz16
PCPD2              80.00 usec
PLW2         30.00000000 W
PLW12         0.39811000 W

F2 - Processing parameters
SI                 65536
SF           150.9027984 MHz
WDW                   no
SSB                    0
LB                     0 Hz
GB                     0
PC                  1.00
```

1H Spectrum

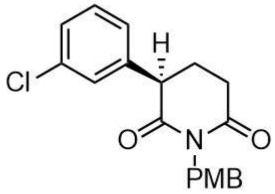

(–)-49

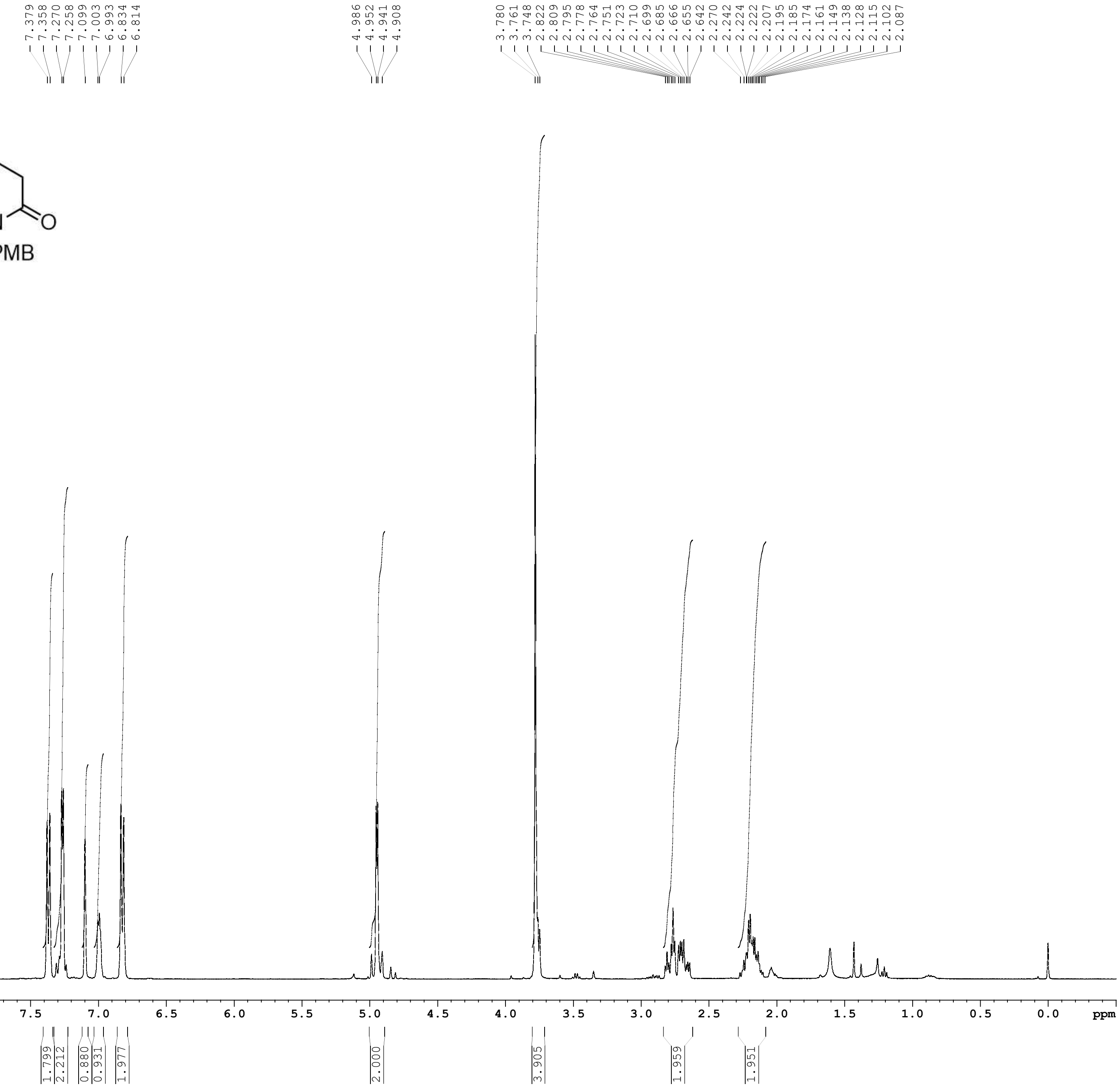

Current Data Parameters
NAME cdw3-255-p
EXPNO 1
PROCNO 1

F2 - Acquisition Parameters
Date_ 20241112
Time 10.15 h
INSTRUM spect
PROBHD Z149000_0038 (
PULPROG zg30
TD 65536
SOLVENT CDCl3
NS 8
DS 2
SWH 6402.049 Hz
FIDRES 0.195375 Hz
AQ 5.1183615 sec
RG 118.08
DW 78.100 usec
DE 13.05 usec
TE 298.1 K
D1 0.10000000 sec
TD0 1
SFO1 400.1328009 MHz
NUC1 1H
P0 3.79 usec
P1 11.38 usec
PLW1 7.41450024 W

F2 - Processing parameters
SI 65536
SF 400.1300107 MHz
WDW no
SSB 0
LB 0 Hz
GB 0
PC 1.00

1H spectrum

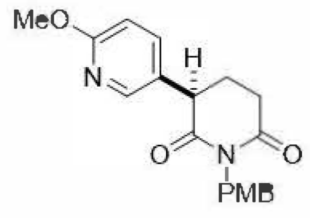

(–)-47

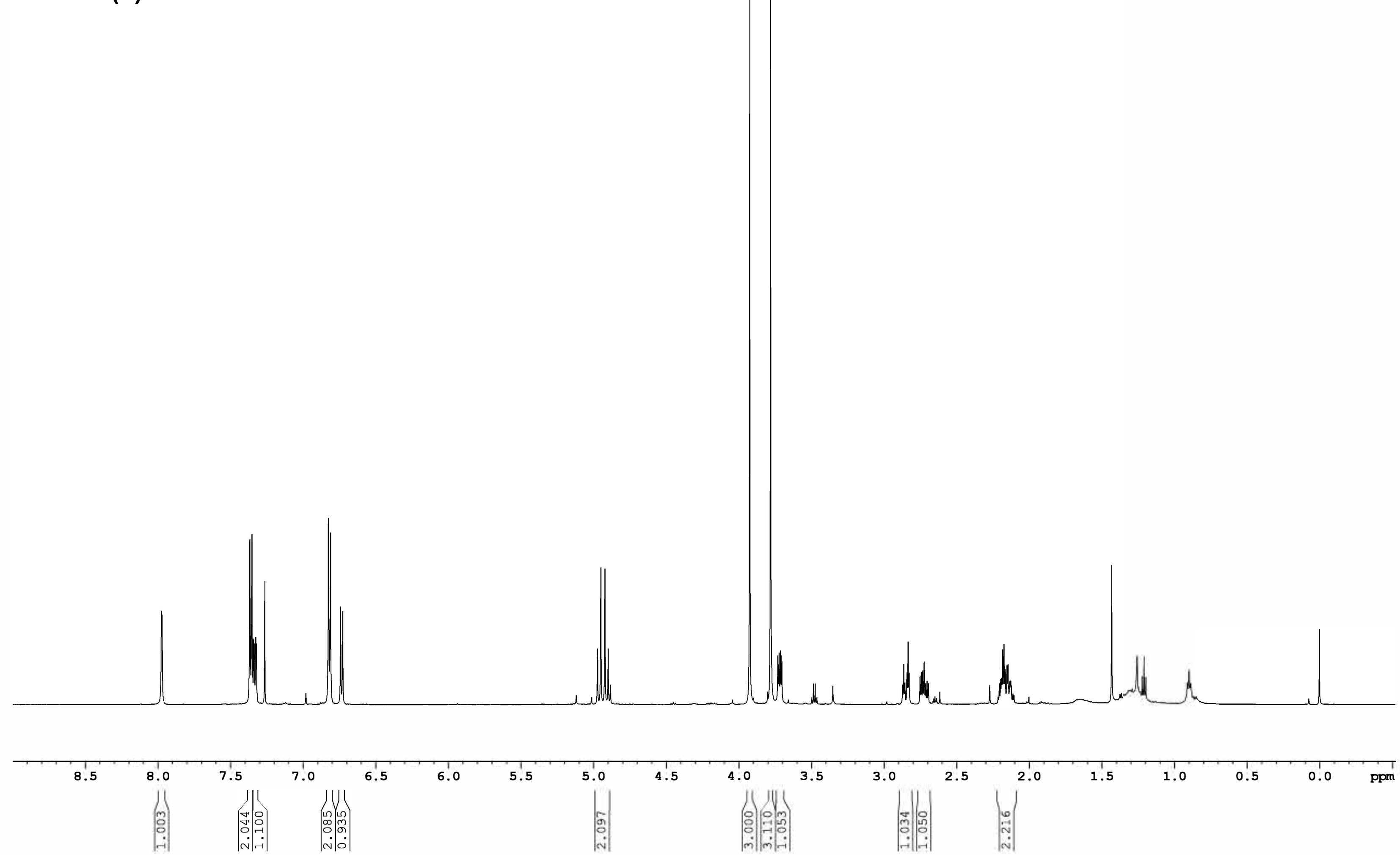


```
Current Data Parameters
NAME      LCB-1-189-600H
EXPNO                  1
PROCNO                 1

F2 - Acquisition Parameters
Date_           20241030
Time                8.43
INSTRUM            av600
PROBHD   5 mm CPBBO BB-
PULPROG             zg30
TD                 98074
SOLVENT           CDCl3T
NS                     8
DS                     2
SWH            9615.385 Hz
FIDRES         0.098042 Hz
AQ            5.0998478 sec
RG                   161
DW               52.000 usec
DE                53.12 usec
TE                297.9 K
D1           0.10000000 sec
TD0                    1

======== CHANNEL f1 ========
SFO1        600.1342009 MHz
NUC1                 1H
P1                10.00 usec
PLW1        30.00000000 W

F2 - Processing parameters
SI                 65536
SF          600.1300338 MHz
WDW                   EM
SSB                    0
LB                  0.30 Hz
GB                     0
PC                  1.00
```

1H spectrum

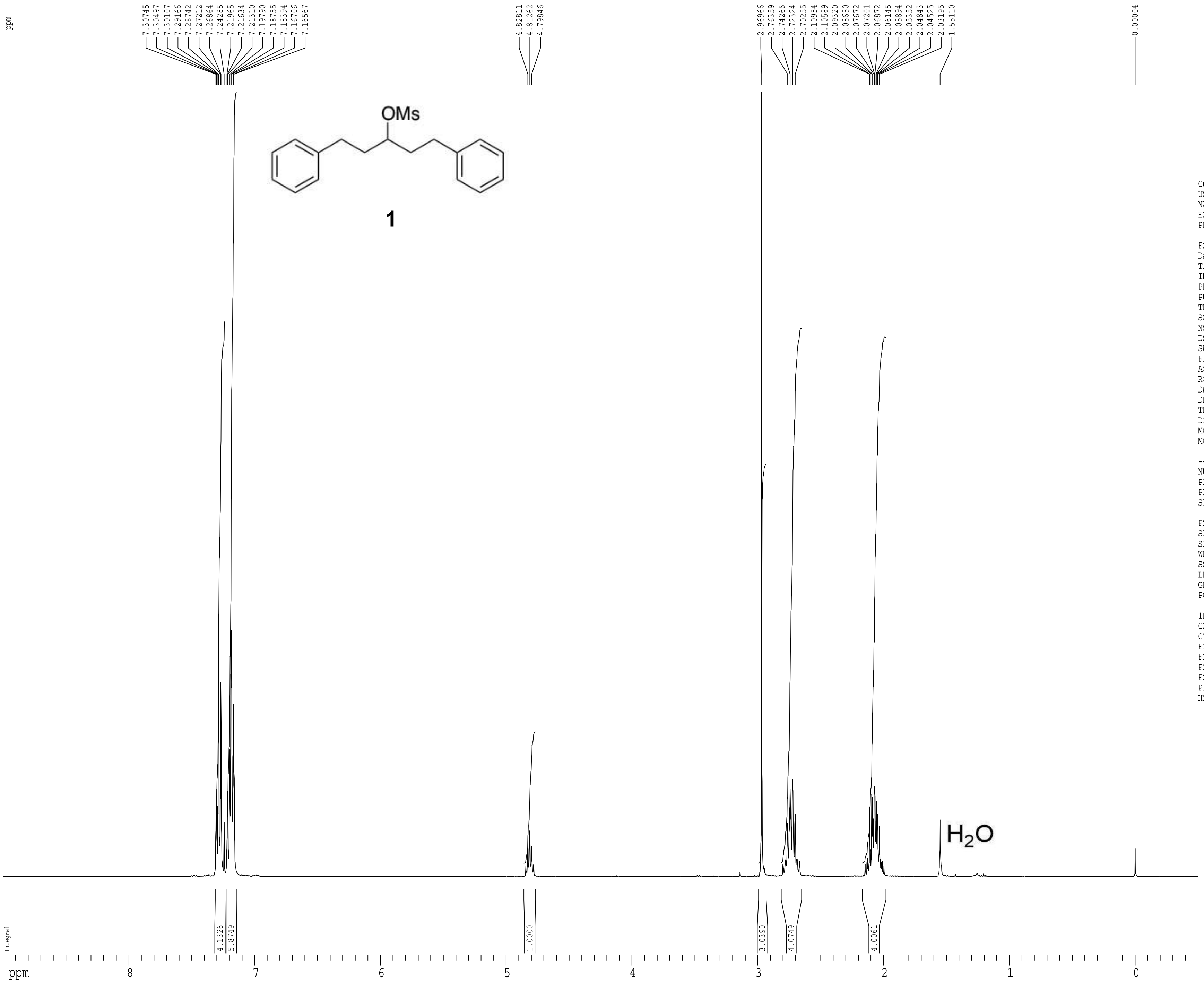

Current Data Parameters
USER chloew10
NAME cdw-2-118-p2
EXPNO 1
PROCNO 1

F2 - Acquisition Parameters
Date_ 20230925
Time 12.38
INSTRUM drx400
PROBHD 5 mm QNP H/F/P
PULPROG zg30
TD 65536
SOLVENT CDCl3
NS 8
DS 2
SWH 6410.256 Hz
FIDRES 0.097813 Hz
AQ 5.1118579 sec
RG 181
DW 78.000 usec
DE 4.50 usec
TE 298.1 K
D1 0.10000000 sec
MCREST 0.00000000 sec
MCWRK 0.01500000 sec

======== CHANNEL f1 ========
NUC1 1H
P1 12.00 usec
PL1 -1.10 dB
SFO1 400.1328009 MHz

F2 - Processing parameters
SI 65536
SF 400.1300278 MHz
WDW no
SSB 0
LB 0.00 Hz
GB 0
PC 2.00

1D NMR plot parameters
CX 22.80 cm
CY 15.00 cm
F1P 9.000 ppm
F1 3601.17 Hz
F2P -0.500 ppm
F2 -200.06 Hz
PPMCM 0.41667 ppm/cm
HZCM 166.72086 Hz/cm

1H spectrum

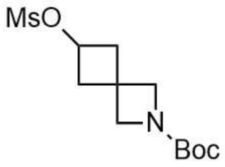

SI-1

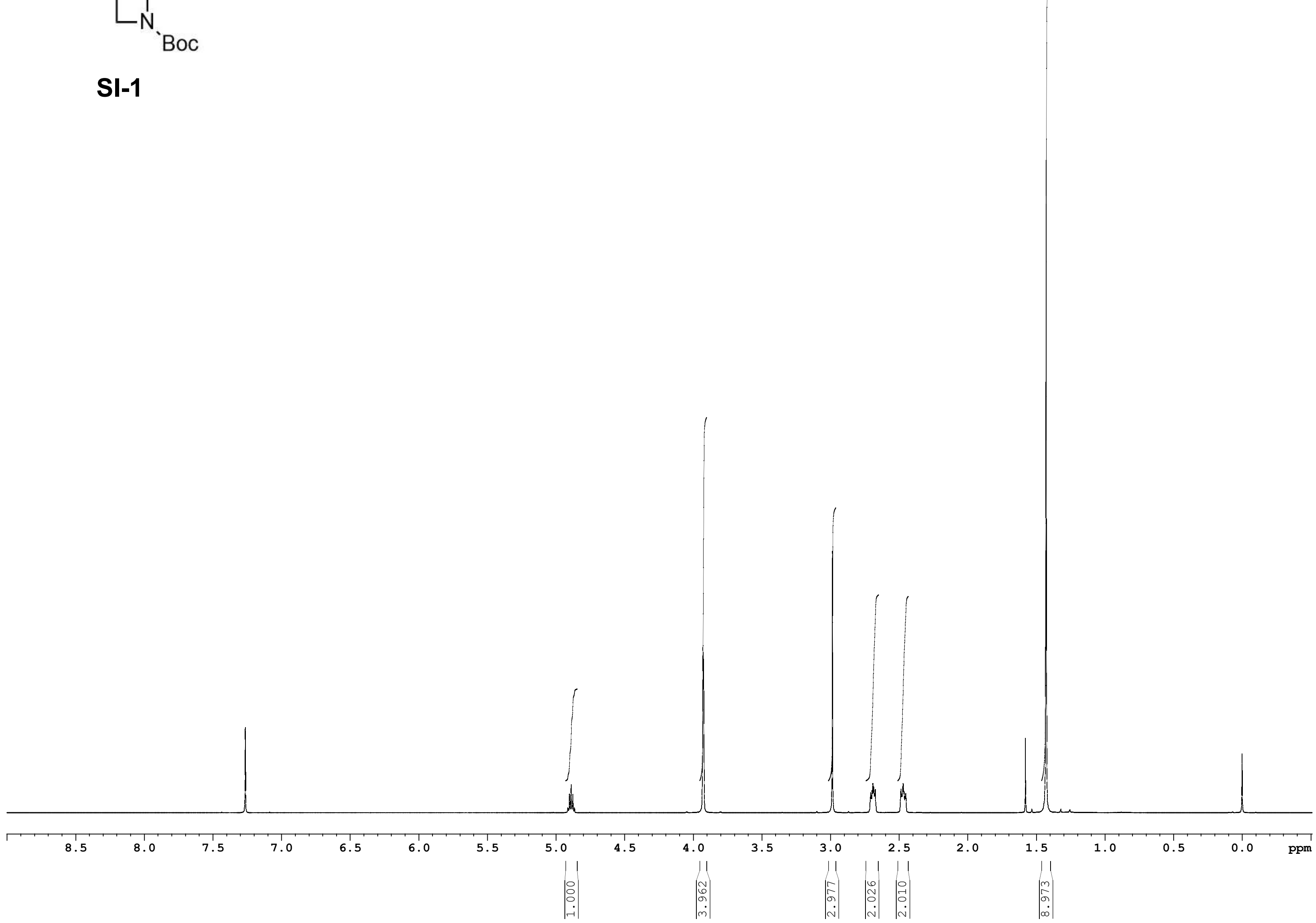


```
Current Data Parameters
NAME          cdw3-159-p
EXPNO                  1
PROCNO                 1

F2 - Acquisition Parameters
Date_           20240727
Time               10.34
INSTRUM            av600
PROBHD   5 mm CPBBO BB-
PULPROG             zg30
TD                 98074
SOLVENT            CDCl3
NS                     8
DS                     2
SWH            9615.385 Hz
FIDRES         0.098042 Hz
AQ            5.0998478 sec
RG                   256
DW                52.000 usec
DE                 53.12 usec
TE                 298.0 K
D1            0.10000000 sec
TD0                    1

======== CHANNEL f1 ========
SFO1        600.1342009 MHz
NUC1                  1H
P1                 10.00 usec
PLW1         30.00000000 W

F2 - Processing parameters
SI                 65536
SF          600.1300327 MHz
WDW                   no
SSB                    0
LB                     0 Hz
GB                     0
PC                  1.00
```

13C spectrum with 1H decoupling

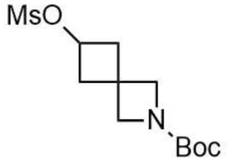

SI-1

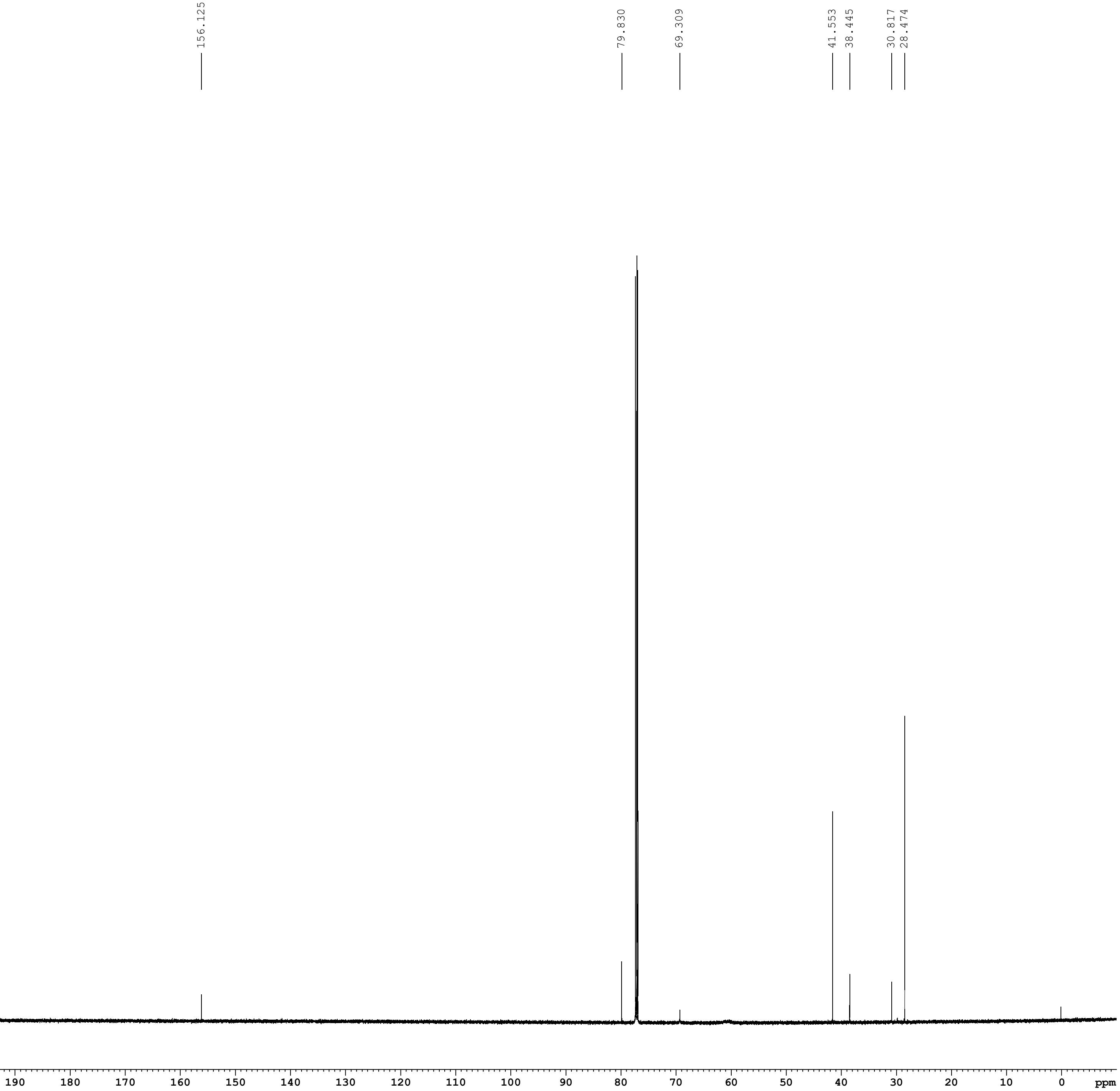


```
Current Data Parameters
NAME        cdw3-159-c13
EXPNO                  1
PROCNO                 1

F2 - Acquisition Parameters
Date_           20240803
Time               11.40
INSTRUM            av600
PROBHD   5 mm CPBBO BB-
PULPROG          zgdc30
TD                 65536
SOLVENT            CDCl3
NS                   650
DS                     4
SWH            36231.883 Hz
FIDRES          0.552855 Hz
AQ             0.9043968 sec
RG                  2050
DW                13.800 usec
DE                 19.65 usec
TE                 297.9 K
D1            0.40000001 sec
D11           0.03000000 sec
TD0                    1

======== CHANNEL f1 ========
SFO1         150.9194080 MHz
NUC1                 13C
P1                 10.00 usec
PLW1         68.40000153 W

======== CHANNEL f2 ========
SFO2         600.1330010 MHz
NUC2                  1H
CPDPRG[2          waltz16
PCPD2              80.00 usec
PLW2         30.00000000 W
PLW12         0.39811000 W

F2 - Processing parameters
SI                 65536
SF           150.9027962 MHz
WDW                   no
SSB                    0
LB                     0 Hz
GB                     0
PC                  1.00
```

1H spectrum

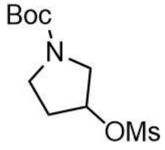

SI-2

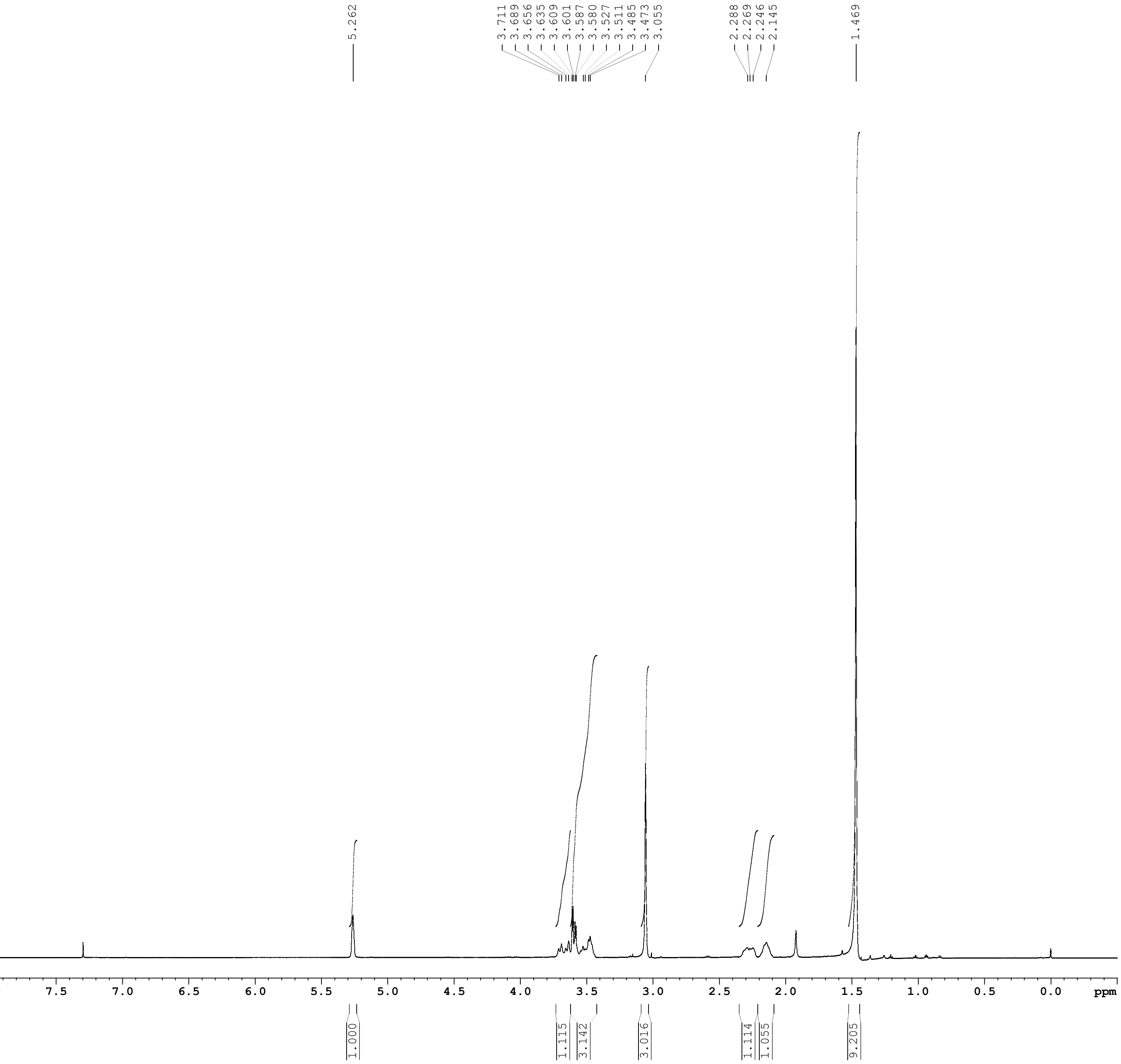


```
Current Data Parameters
NAME      cdw-3-130-1h-good
EXPNO                     1
PROCNO                    1

F2 - Acquisition Parameters
Date_              20240725
Time                   9.34
INSTRUM               av600
PROBHD    5 mm CPBBO BB-
PULPROG                zg30
TD                    98074
SOLVENT               CDCl3
NS                        8
DS                        2
SWH                9615.385 Hz
FIDRES             0.098042 Hz
AQ                5.0998478 sec
RG                     28.5
DW                   52.000 usec
DE                    53.12 usec
TE                    298.0 K
D1               0.10000000 sec
TD0                       1

======== CHANNEL f1 ========
SFO1          600.1342009 MHz
NUC1                     1H
P1                    10.00 usec
PLW1         30.00000000 W

F2 - Processing parameters
SI                    65536
SF            600.1300123 MHz
WDW                      no
SSB                       0
LB                        0 Hz
GB                        0
PC                     1.00
```

13C spectrum with 1H decoupling

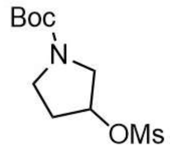

SI-2

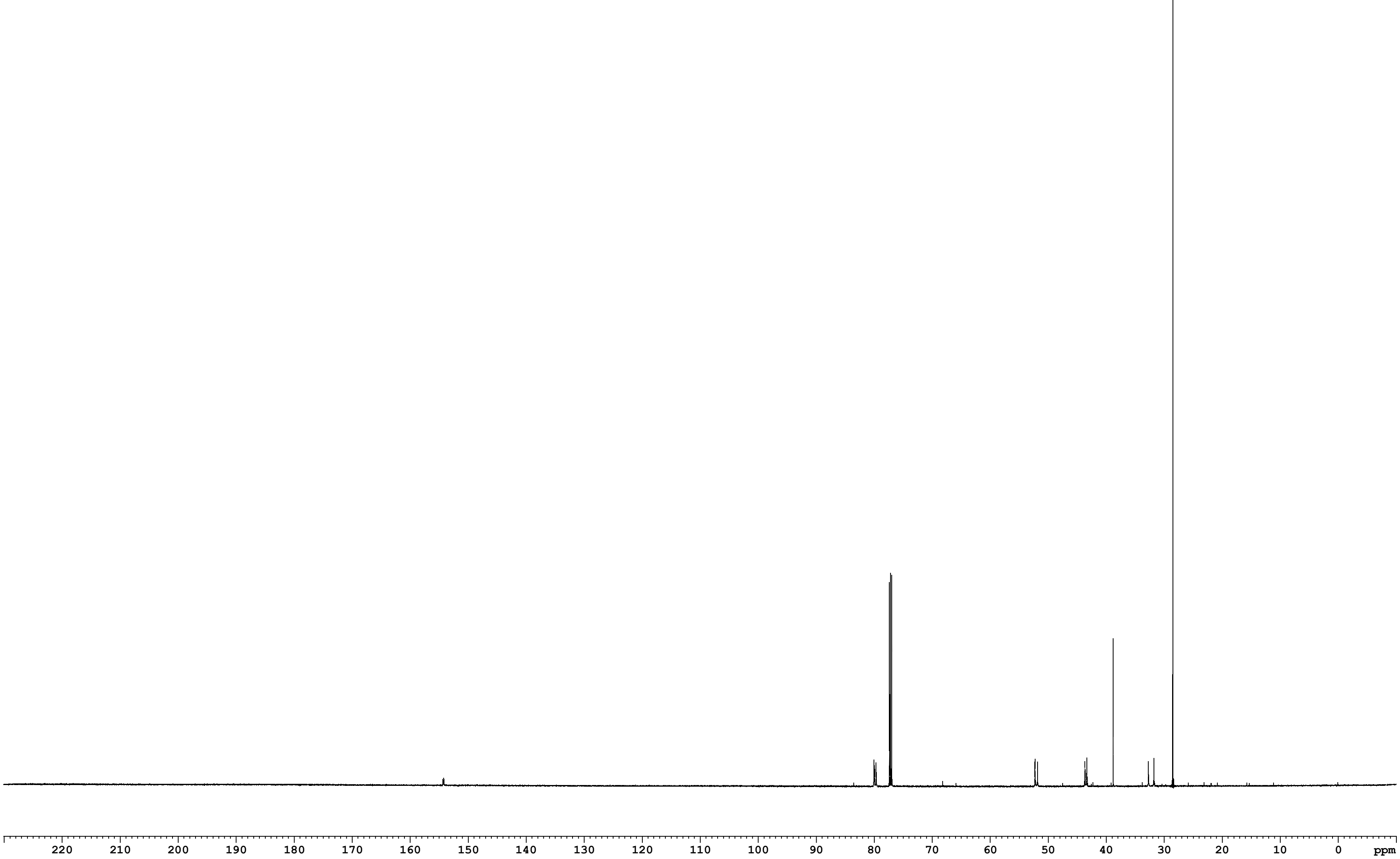

Current Data Parameters
NAME cdw-3-130-c13
EXPNO 1
PROCNO 1

F2 - Acquisition Parameters
Date_ 20240725
Time 9.47
INSTRUM av600
PROBHD 5 mm CPBBO BB-
PULPROG zgdc30
TD 65536
SOLVENT CDCl3
NS 330
DS 4
SWH 36231.883 Hz
FIDRES 0.552855 Hz
AQ 0.9043968 sec
RG 2050
DW 13.800 usec
DE 19.65 usec
TE 297.9 K
D1 0.40000001 sec
D11 0.03000000 sec
TD0 1

======== CHANNEL f1 ========
SFO1 150.9194080 MHz
NUC1 13C
P1 10.00 usec
PLW1 68.40000153 W

======== CHANNEL f2 ========
SFO2 600.1330010 MHz
NUC2 1H
CPDPRG[2 waltz16
PCPD2 80.00 usec
PLW2 30.00000000 W
PLW12 0.39811000 W

F2 - Processing parameters
SI 65536
SF 150.9028037 MHz
WDW no
SSB 0
LB 0 Hz
GB 0
PC 1.00

1H spectrum

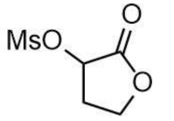

SI-3

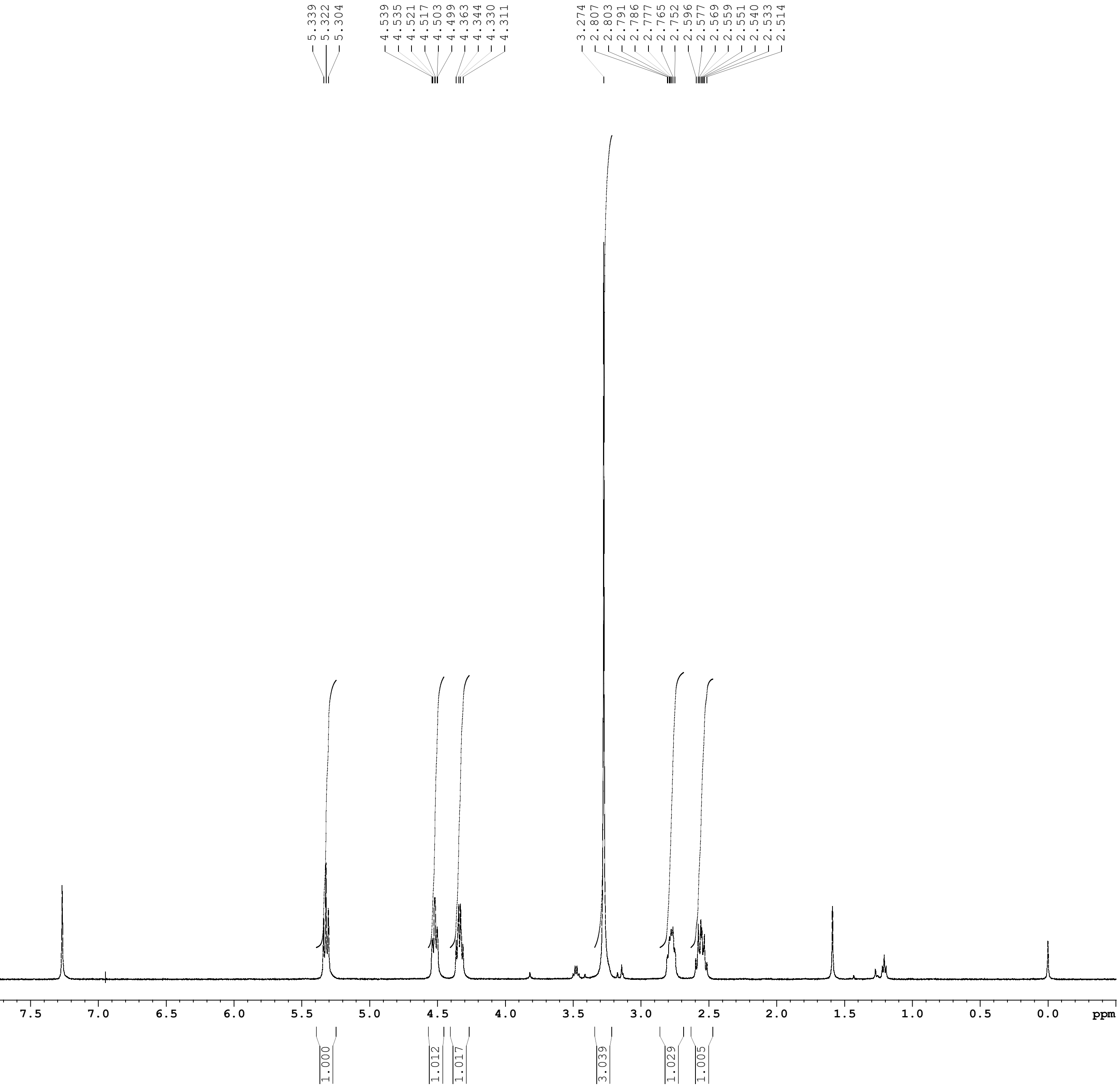

Current Data Parameters
NAME cdw3-173-p
EXPNO 1
PROCNO 1

F2 - Acquisition Parameters
Date_ 20240815
Time 9.44
INSTRUM gn500
PROBHD 5 mm broadband
PULPROG zg30
TD 81728
SOLVENT CDCl3
NS 8
DS 2
SWH 8012.820 Hz
FIDRES 0.098043 Hz
AQ 5.0998273 sec
RG 1024
DW 62.400 usec
DE 6.00 usec
TE 298.0 K
D1 0.10000000 sec
MCREST 0 sec
MCWRK 0.01500000 sec

======== CHANNEL f1 ========
NUC1 1H
P1 12.00 usec
PL1 -6.00 dB
SFO1 498.4534891 MHz

F2 - Processing parameters
SI 65536
SF 498.4500266 MHz
WDW no
SSB 0
LB 0 Hz
GB 0
PC 1.00

13C Spectrum

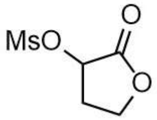

SI-3

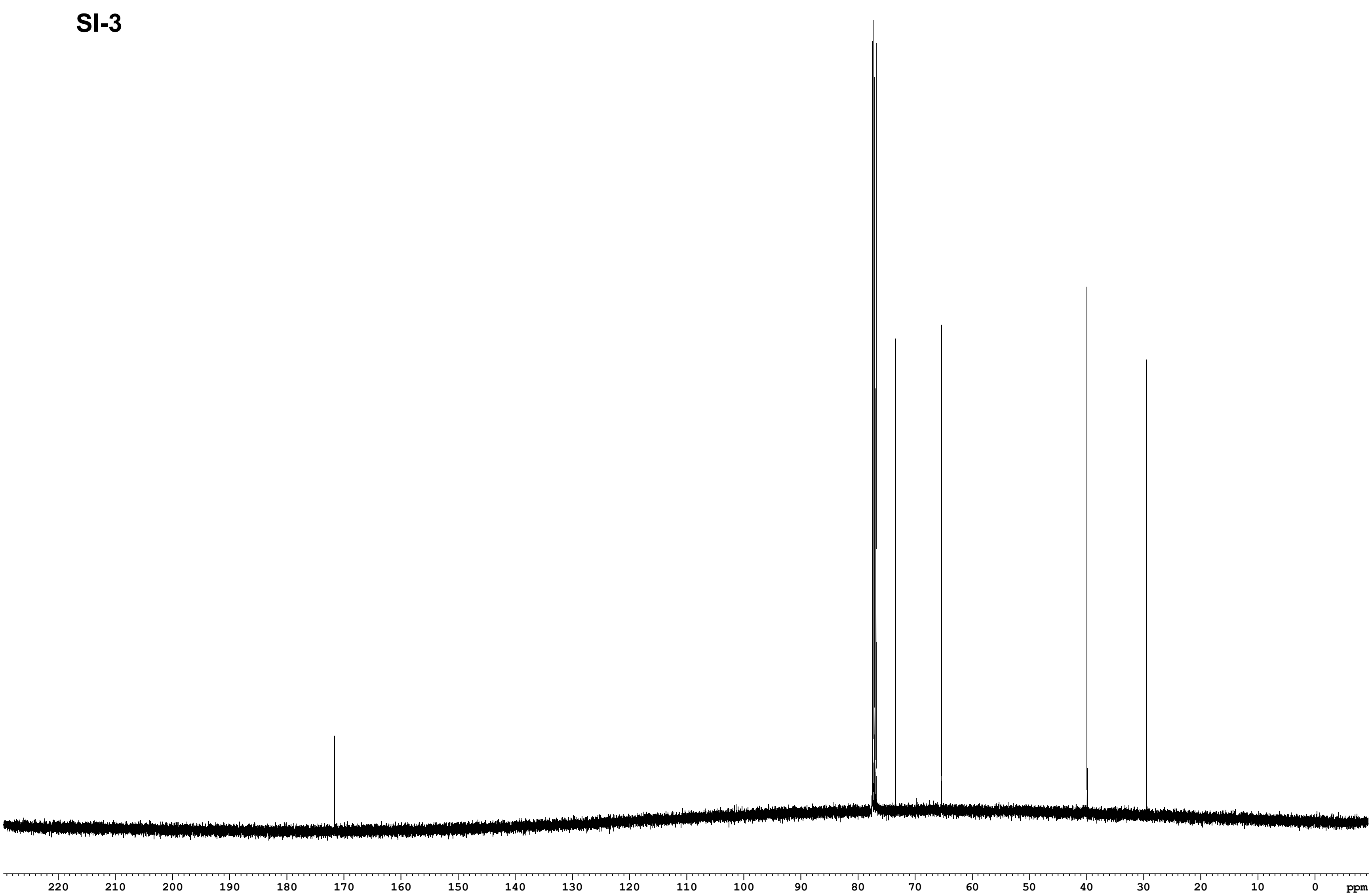


```
Current Data Parameters
NAME        asml-62-c13
EXPNO                 1
PROCNO                1

F2 - Acquisition Parameters
Date_          20241206
Time               9.10 h
INSTRUM           spect
PROBHD   Z149000_0038 (
PULPROG          zgpg30
TD                48074
SOLVENT           CDCl3
NS                  420
DS                    4
SWH           24038.461 Hz
FIDRES         1.000061 Hz
AQ            0.9999392 sec
RG                48.92
DW               20.800 usec
DE                18.00 usec
TE                298.1 K
D1           0.25000000 sec
D11          0.03000000 sec
TD0                   1
SFO1        100.6238359 MHz
NUC1                13C
P0                 3.16 usec
P1                 9.49 usec
PLW1        41.29999924 W
SFO2        400.1316005 MHz
NUC2                 1H
CPDPRG[2        waltz65
PCPD2             80.00 usec
PLW2         7.41450024 W
PLW12        0.15003000 W
PLW13        0.07534500 W

F2 - Processing parameters
SI                65536
SF          100.6127574 MHz
WDW                  no
SSB                   0
LB                    0 Hz
GB                    0
PC                 1.40
```

1H spectrum

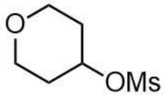

SI-4

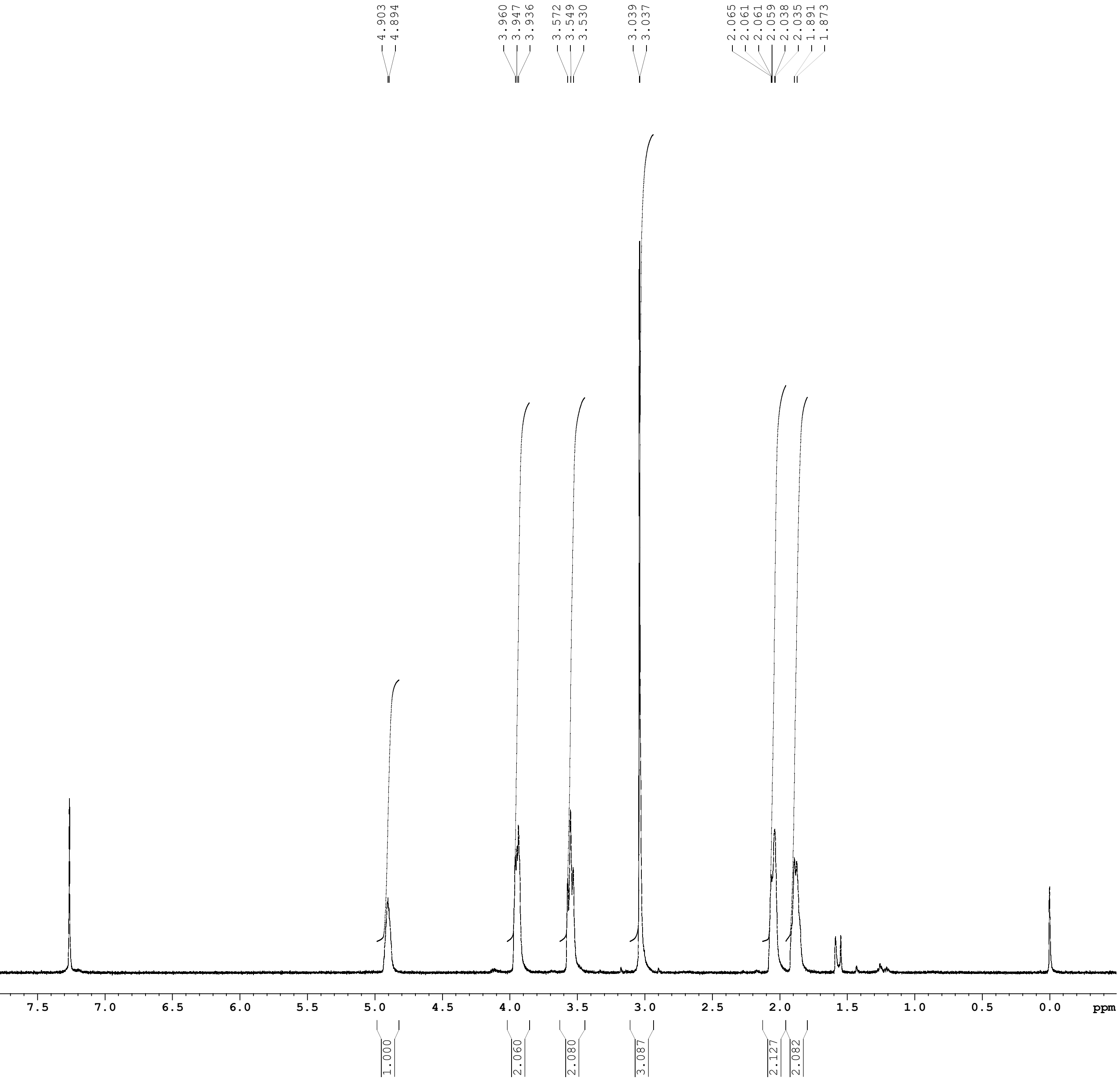


```
Current Data Parameters
NAME          cdw-3-24-p
EXPNO                  1
PROCNO                 1

F2 - Acquisition Parameters
Date_           20240403
Time                8.31
INSTRUM            gn500
PROBHD   5 mm broadband
PULPROG             zg30
TD                 81728
SOLVENT            CDCl3
NS                     8
DS                     2
SWH             8012.820 Hz
FIDRES          0.098043 Hz
AQ             5.0998273 sec
RG                1149.4
DW                62.400 usec
DE                  6.00 usec
TE                 298.0 K
D1            0.10000000 sec
MCREST                 0 sec
MCWRK         0.01500000 sec

======== CHANNEL f1 ========
NUC1                  1H
P1                 12.00 usec
PL1                -6.00 dB
SFO1         498.4534891 MHz

F2 - Processing parameters
SI                 65536
SF           498.4500272 MHz
WDW                   no
SSB                    0
LB                     0 Hz
GB                     0
PC                  1.00
```

1H spectrum

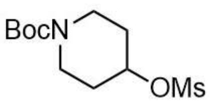

SI-5

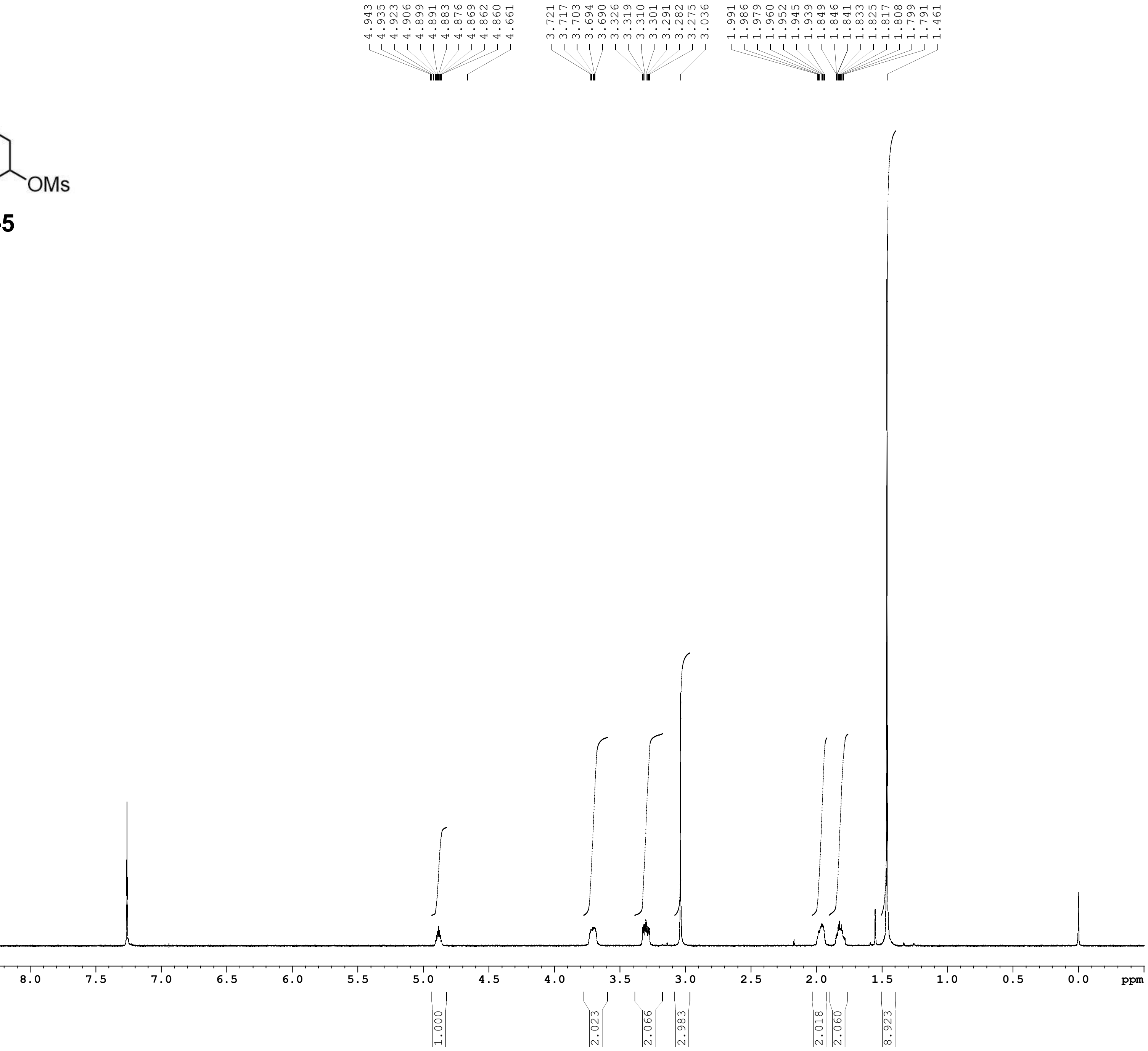


```
Current Data Parameters
NAME          cdw-2-230-p
EXPNO                   1
PROCNO                  1

F2 - Acquisition Parameters
Date_            20240118
Time                 8.47
INSTRUM             gn500
PROBHD   5 mm broadband
PULPROG              zg30
TD                  81728
SOLVENT             CDCl3
NS                      8
DS                      2
SWH              8012.820 Hz
FIDRES           0.098043 Hz
AQ              5.0998273 sec
RG                 1290.2
DW                 62.400 usec
DE                   6.00 usec
TE                  298.4 K
D1             0.10000000 sec
MCREST                  0 sec
MCWRK          0.01500000 sec

======== CHANNEL f1 ========
NUC1                   1H
P1                  12.00 usec
PL1                 -6.00 dB
SFO1          498.4534891 MHz

F2 - Processing parameters
SI                  65536
SF            498.4500294 MHz
WDW                    no
SSB                     0
LB                      0 Hz
GB                      0
PC                   1.00
```

1H spectrum

SI-6

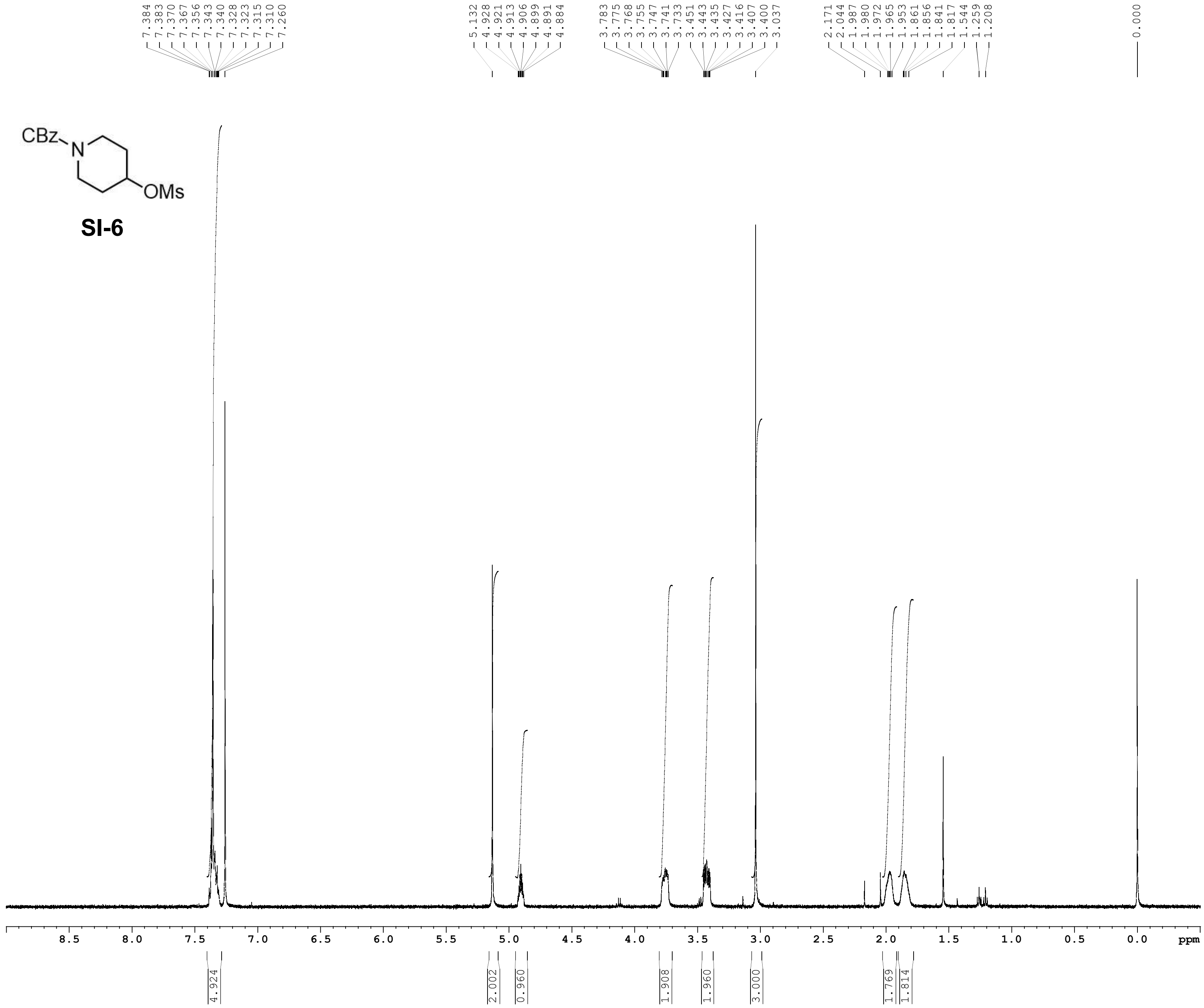


```
Current Data Parameters
NAME        cdw-2-212-p
EXPNO                 1
PROCNO                1

F2 - Acquisition Parameters
Date_          20231222
Time              12.55
INSTRUM           gn500
PROBHD   5 mm broadband
PULPROG            zg30
TD                81728
SOLVENT           CDCl3
NS                    8
DS                    2
SWH            8012.820 Hz
FIDRES         0.098043 Hz
AQ            5.0998273 sec
RG                 2048
DW               62.400 usec
DE                 6.00 usec
TE                298.4 K
D1           0.10000000 sec
MCREST                0 sec
MCWRK        0.01500000 sec

======== CHANNEL f1 ========
NUC1                 1H
P1                12.00 usec
PL1               -6.00 dB
SFO1        498.5534899 MHz

F2 - Processing parameters
SI                65536
SF          498.5500263 MHz
WDW                  no
SSB                   0
LB                    0 Hz
GB                    0
PC                 1.00
```

1H spectrum

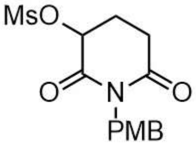

55

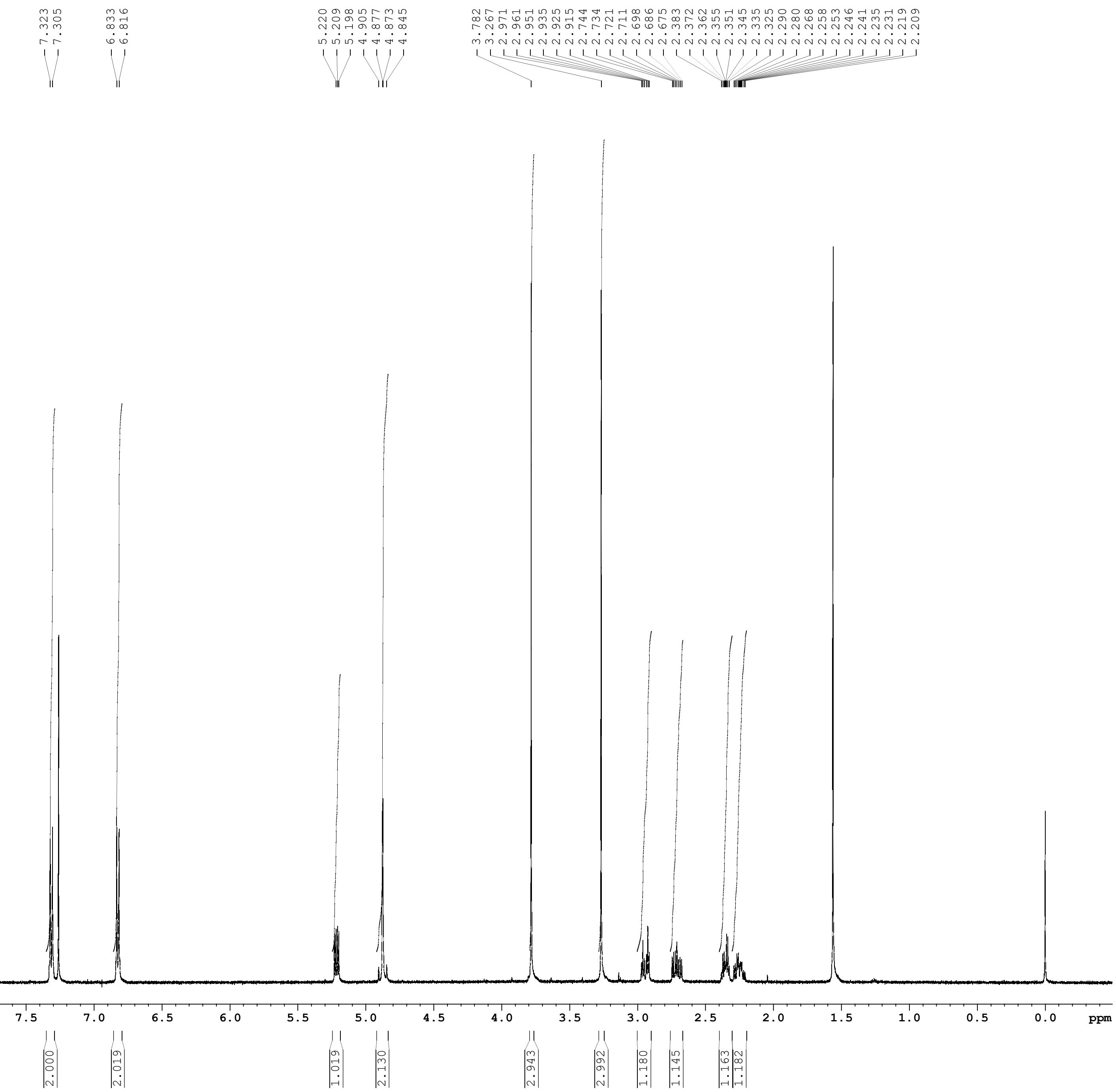


```
Current Data Parameters
NAME          cdw3-207p
EXPNO                 1
PROCNO                1

F2 - Acquisition Parameters
Date_          20240930
Time               9.50
INSTRUM           gn500
PROBHD   5 mm broadband
PULPROG            zg30
TD                81728
SOLVENT           CDCl3
NS                    8
DS                    2
SWH            8012.820 Hz
FIDRES         0.098043 Hz
AQ            5.0998273 sec
RG               1625.5
DW               62.400 usec
DE                 6.00 usec
TE                298.0 K
D1           0.10000000 sec
MCREST                0 sec
MCWRK        0.01500000 sec

======== CHANNEL f1 ========
NUC1                 1H
P1                12.00 usec
PL1               -6.00 dB
SFO1        498.4534891 MHz

F2 - Processing parameters
SI                65536
SF          498.4500293 MHz
WDW                  no
SSB                   0
LB                    0 Hz
GB                    0
PC                 1.00
```

1H spectrum

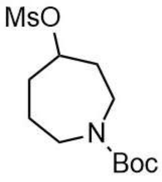

SI-7

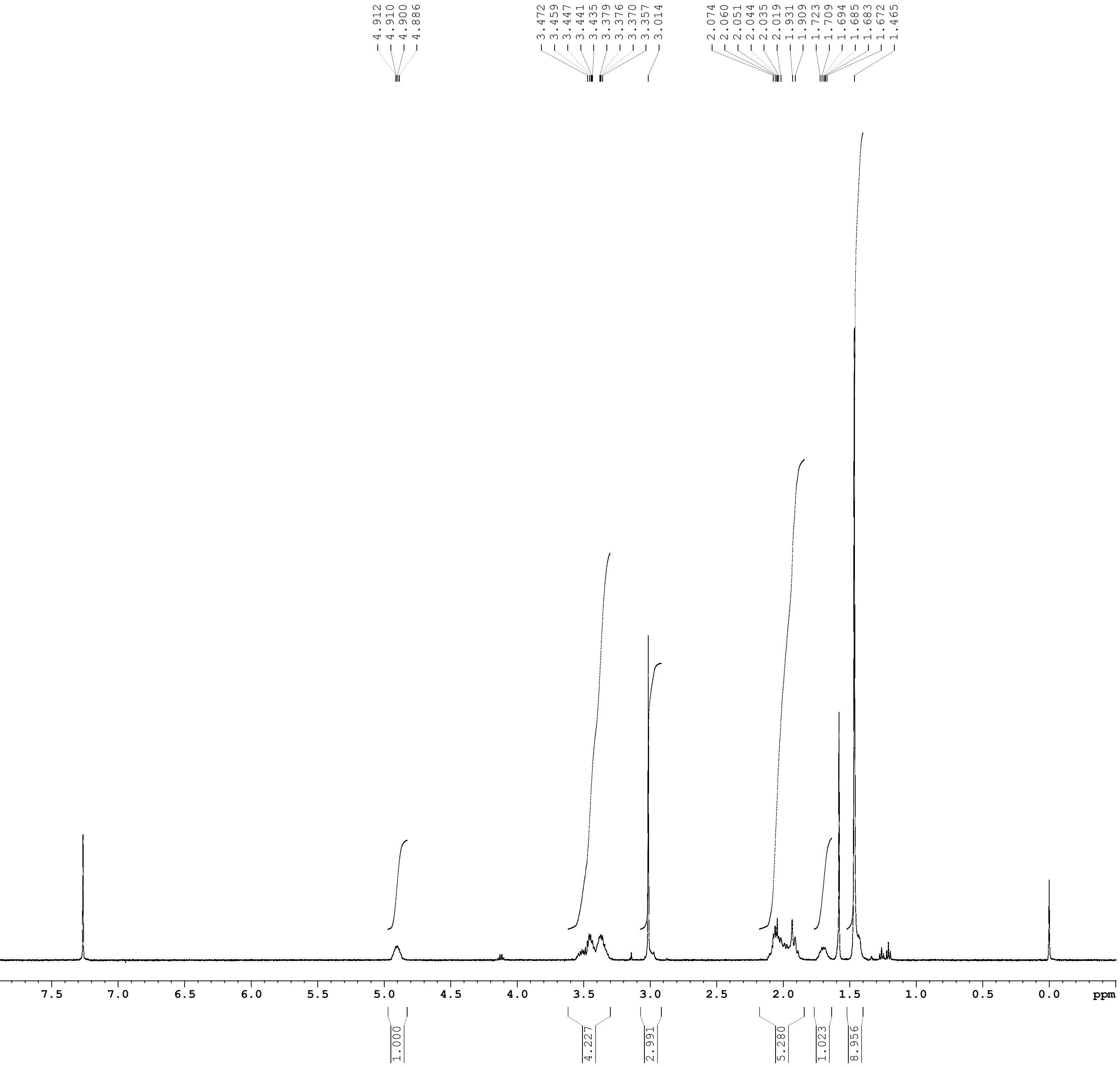


```
Current Data Parameters
NAME        cdw-3-138-p
EXPNO                 1
PROCNO                1

F2 - Acquisition Parameters
Date_          20240627
Time               8.30
INSTRUM           gn500
PROBHD   5 mm broadband
PULPROG            zg30
TD                81728
SOLVENT           CDCl3
NS                    8
DS                    2
SWH            8012.820 Hz
FIDRES         0.098043 Hz
AQ            5.0998273 sec
RG               1290.2
DW               62.400 usec
DE                 6.00 usec
TE                298.0 K
D1           0.10000000 sec
MCREST                0 sec
MCWRK        0.01500000 sec

======== CHANNEL f1 ========
NUC1                 1H
P1                12.00 usec
PL1               -6.00 dB
SFO1        498.4534891 MHz

F2 - Processing parameters
SI                65536
SF          498.4500285 MHz
WDW                  no
SSB                   0
LB                    0 Hz
GB                    0
PC                 1.00
```

13C spectrum with 1H decoupling

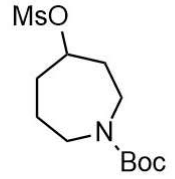

SI-7

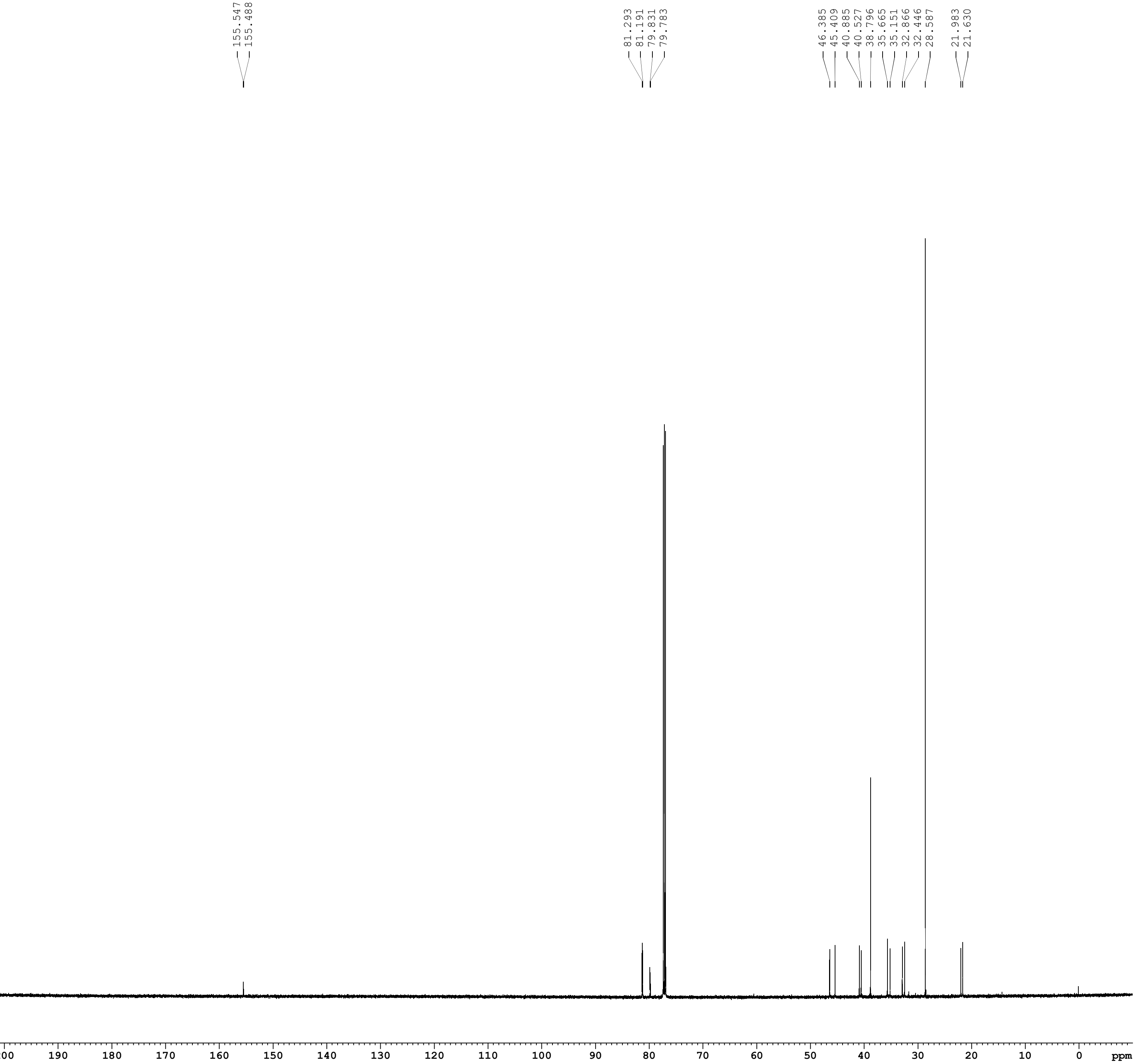


```
Current Data Parameters
NAME     cdw3-138-600-c13
EXPNO                   1
PROCNO                  1

F2 - Acquisition Parameters
Date_            20241206
Time                 9.24
INSTRUM             av600
PROBHD   5 mm CPBBO BB-
PULPROG            zgdc30
TD                  65536
SOLVENT             CDCl3
NS                    520
DS                      4
SWH             36231.883 Hz
FIDRES           0.552855 Hz
AQ              0.9043968 sec
RG                   2050
DW                 13.800 usec
DE                  19.65 usec
TE                  297.9 K
D1             0.40000001 sec
D11            0.03000000 sec
TD0                     1

======== CHANNEL f1 ========
SFO1         150.9194080 MHz
NUC1                  13C
P1                  10.00 usec
PLW1         68.40000153 W

======== CHANNEL f2 ========
SFO2         600.1330010 MHz
NUC2                   1H
CPDPRG[2           waltz16
PCPD2               80.00 usec
PLW2         30.00000000 W
PLW12         0.39811000 W

F2 - Processing parameters
SI                  65536
SF           150.9027961 MHz
WDW                    no
SSB                     0
LB                      0 Hz
GB                      0
PC                   1.00
```

1H spectrum

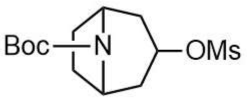

SI-8

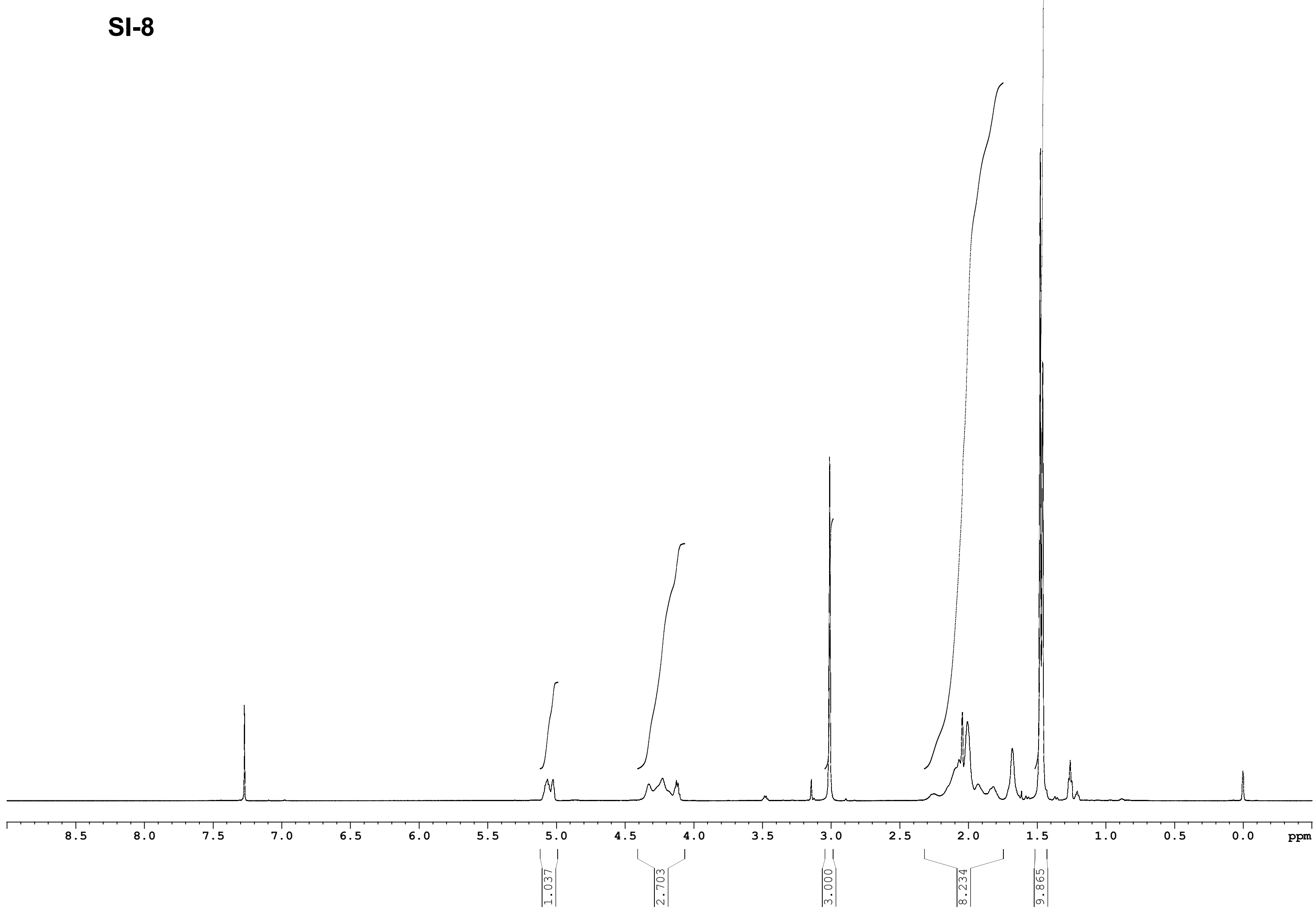


```
Current Data Parameters
NAME          cdw3-158-p
EXPNO                  1
PROCNO                 1

F2 - Acquisition Parameters
Date_           20240727
Time               10.27
INSTRUM            av600
PROBHD   5 mm CPBBO BB-
PULPROG             zg30
TD                 98074
SOLVENT            CDCl3
NS                     8
DS                     2
SWH             9615.385 Hz
FIDRES          0.098042 Hz
AQ             5.0998478 sec
RG                  90.5
DW                52.000 usec
DE                 53.12 usec
TE                 297.9 K
D1            0.10000000 sec
TD0                    1

======== CHANNEL f1 ========
SFO1         600.1342009 MHz
NUC1                  1H
P1                 10.00 usec
PLW1         30.00000000 W

F2 - Processing parameters
SI                 65536
SF           600.1300284 MHz
WDW                   no
SSB                    0
LB                     0 Hz
GB                     0
PC                  1.00
```

13C spectrum with 1H decoupling

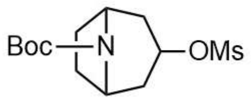

SI-8

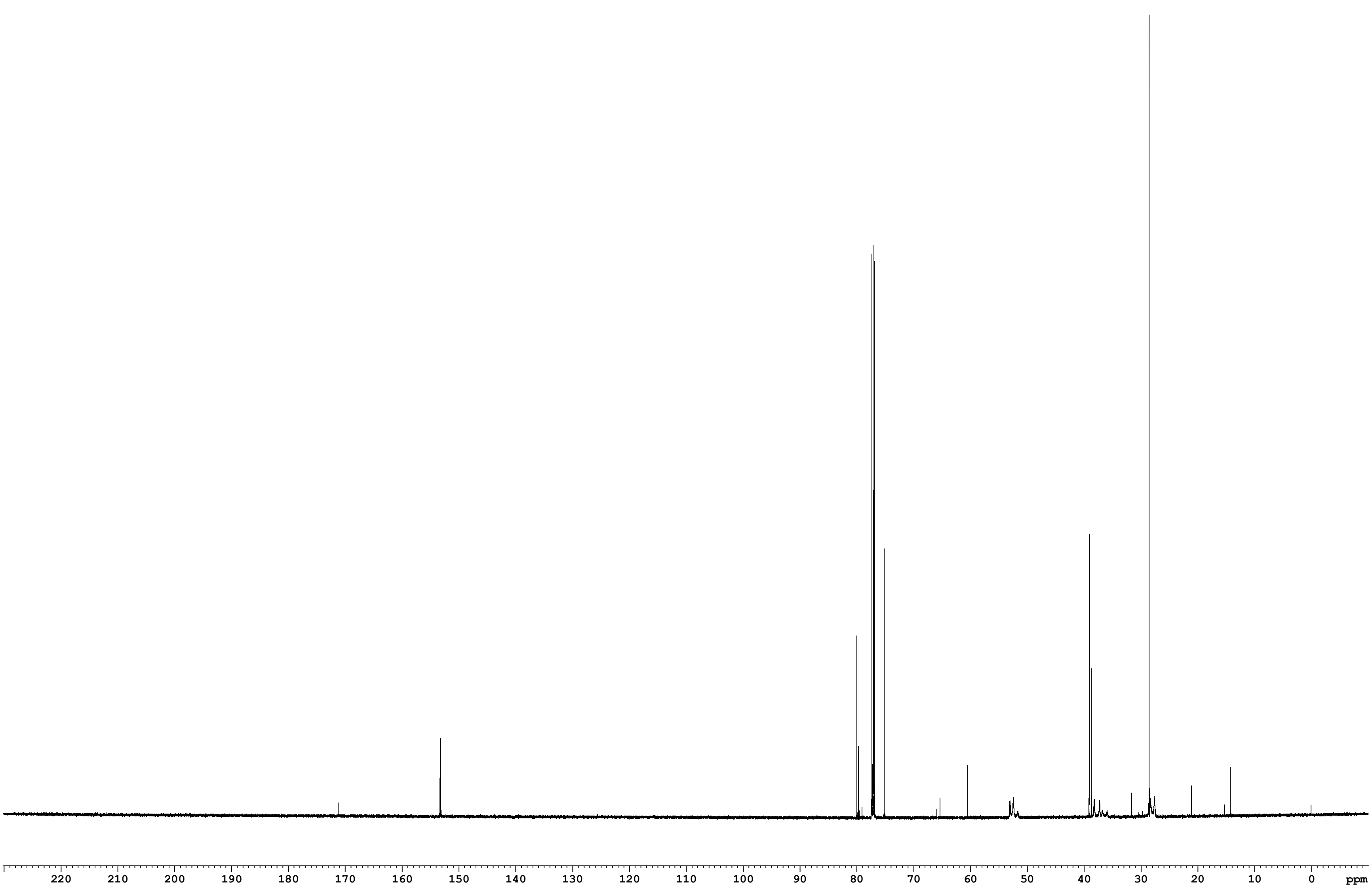


```
Current Data Parameters
NAME       cdw3-158-c13
EXPNO                 1
PROCNO                1

F2 - Acquisition Parameters
Date_          20240803
Time              11.22
INSTRUM           av600
PROBHD   5 mm CPBBO BB-
PULPROG          zgdc30
TD                65536
SOLVENT           CDCl3
NS                  550
DS                    4
SWH           36231.883 Hz
FIDRES         0.552855 Hz
AQ            0.9043968 sec
RG                 2050
DW               13.800 usec
DE                19.65 usec
TE                298.0 K
D1           0.40000001 sec
D11          0.03000000 sec
TD0                   1

======== CHANNEL f1 ========
SFO1        150.9194080 MHz
NUC1                13C
P1                10.00 usec
PLW1        68.40000153 W

======== CHANNEL f2 ========
SFO2        600.1330010 MHz
NUC2                 1H
CPDPRG[2         waltz16
PCPD2             80.00 usec
PLW2        30.00000000 W
PLW12        0.39811000 W

F2 - Processing parameters
SI                65536
SF          150.9028011 MHz
WDW                  no
SSB                   0
LB                    0 Hz
GB                    0
PC                 1.00
```

1H spectrum

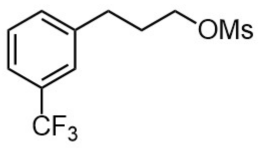

SI-9

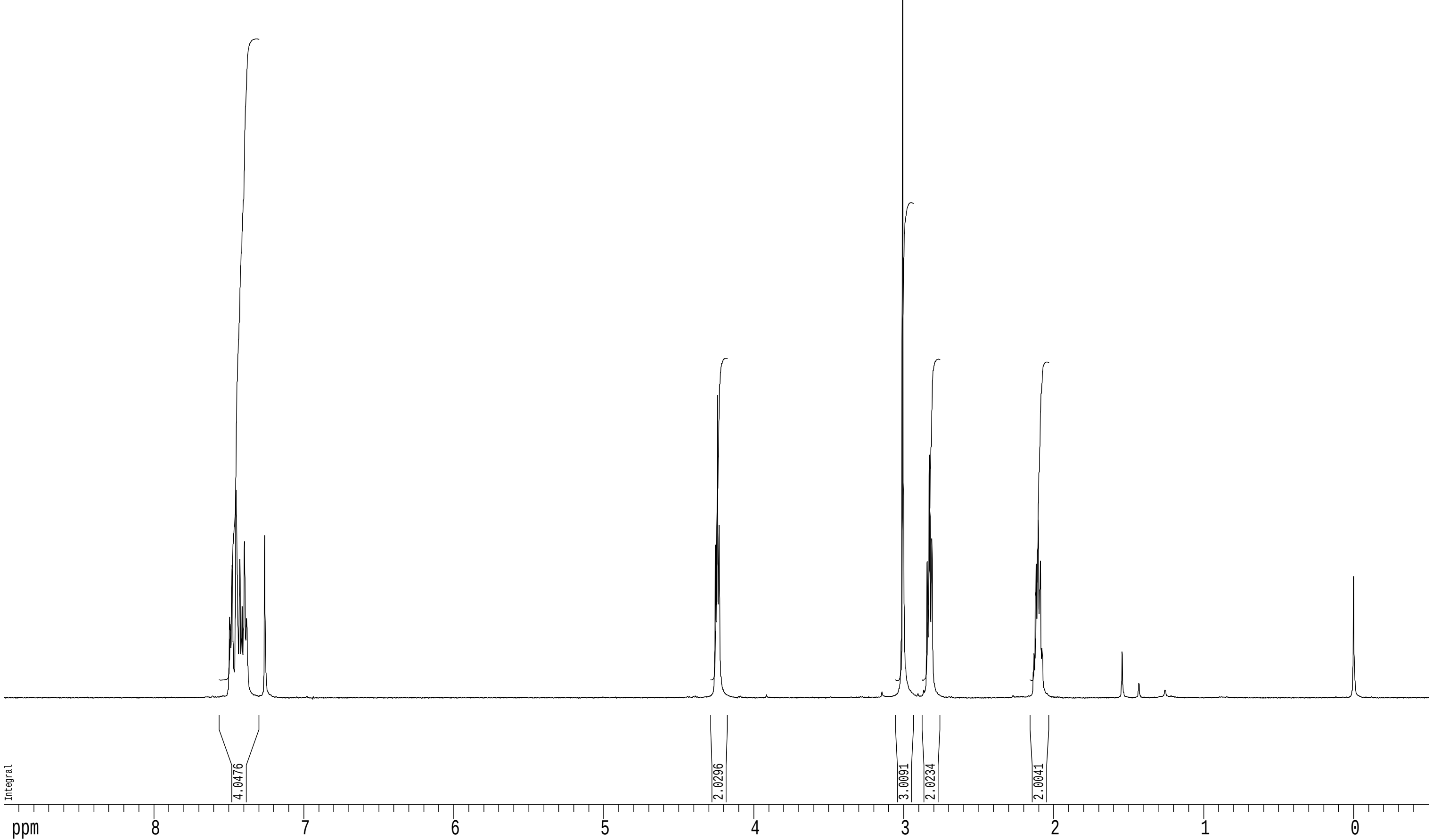

Current Data Parameters
USER nhirbawi
NAME NH-4-187-column
EXPNO 1
PROCNO 1

F2 - Acquisition Parameters
Date_ 20240403
Time 19.33
INSTRUM gn500
PROBHD 5 mm broadband
PULPROG zg30
TD 48074
SOLVENT CDCl3T
NS 8
DS 2
SWH 8012.820 Hz
FIDRES 0.166677 Hz
AQ 2.9998677 sec
RG 1290.2
DW 62.400 usec
DE 6.00 usec
TE 298.0 K
D1 0.10000000 sec
MCREST 0.00000000 sec
MCWRK 0.01500000 sec

======== CHANNEL f1 ========
NUC1 1H
P1 12.00 usec
PL1 -6.00 dB
SFO1 498.4534891 MHz

F2 - Processing parameters
SI 65536
SF 498.4500302 MHz
WDW EM
SSB 0
LB 0.30 Hz
GB 0
PC 1.00

1D NMR plot parameters
CX 20.00 cm
CY 12.50 cm
F1P 9.000 ppm
F1 4486.05 Hz
F2P -0.500 ppm
F2 -249.23 Hz
PPMCM 0.47500 ppm/cm
HZCM 236.76376 Hz/cm

1H spectrum

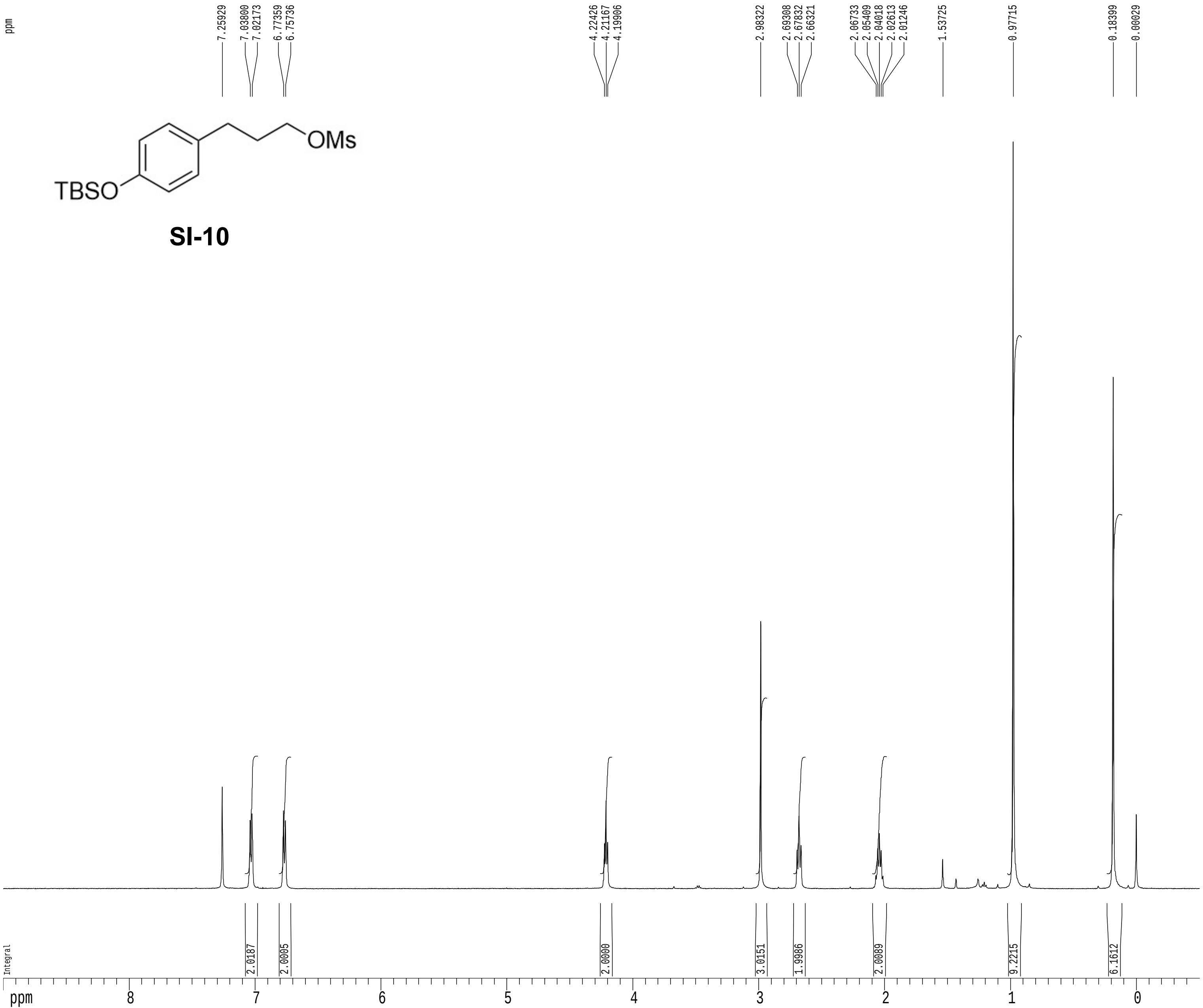

SI-10

Current Data Parameters
USER nhirbawi
NAME NH-4-188-column
EXPNO 1
PROCNO 1

F2 - Acquisition Parameters
Date_ 20240403
Time 19.37
INSTRUM gn500
PROBHD 5 mm broadband
PULPROG zg30
TD 81728
SOLVENT CDCl3T
NS 8
DS 2
SWH 8012.820 Hz
FIDRES 0.098043 Hz
AQ 5.0998774 sec
RG 1149.4
DW 62.400 usec
DE 6.00 usec
TE 298.0 K
D1 0.10000000 sec
MCREST 0.00000000 sec
MCWRK 0.01500000 sec

======== CHANNEL f1 ========
NUC1 1H
P1 12.00 usec
PL1 -6.00 dB
SFO1 498.4534891 MHz

F2 - Processing parameters
SI 65536
SF 498.4500304 MHz
WDW EM
SSB 0
LB 0.30 Hz
GB 0
PC 1.00

1D NMR plot parameters
CX 20.00 cm
CY 12.50 cm
F1P 9.000 ppm
F1 4486.05 Hz
F2P -0.500 ppm
F2 -249.23 Hz
PPMCM 0.47500 ppm/cm
HZCM 236.76376 Hz/cm

1H spectrum

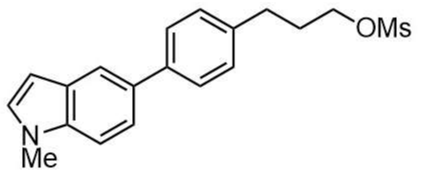

SI-11

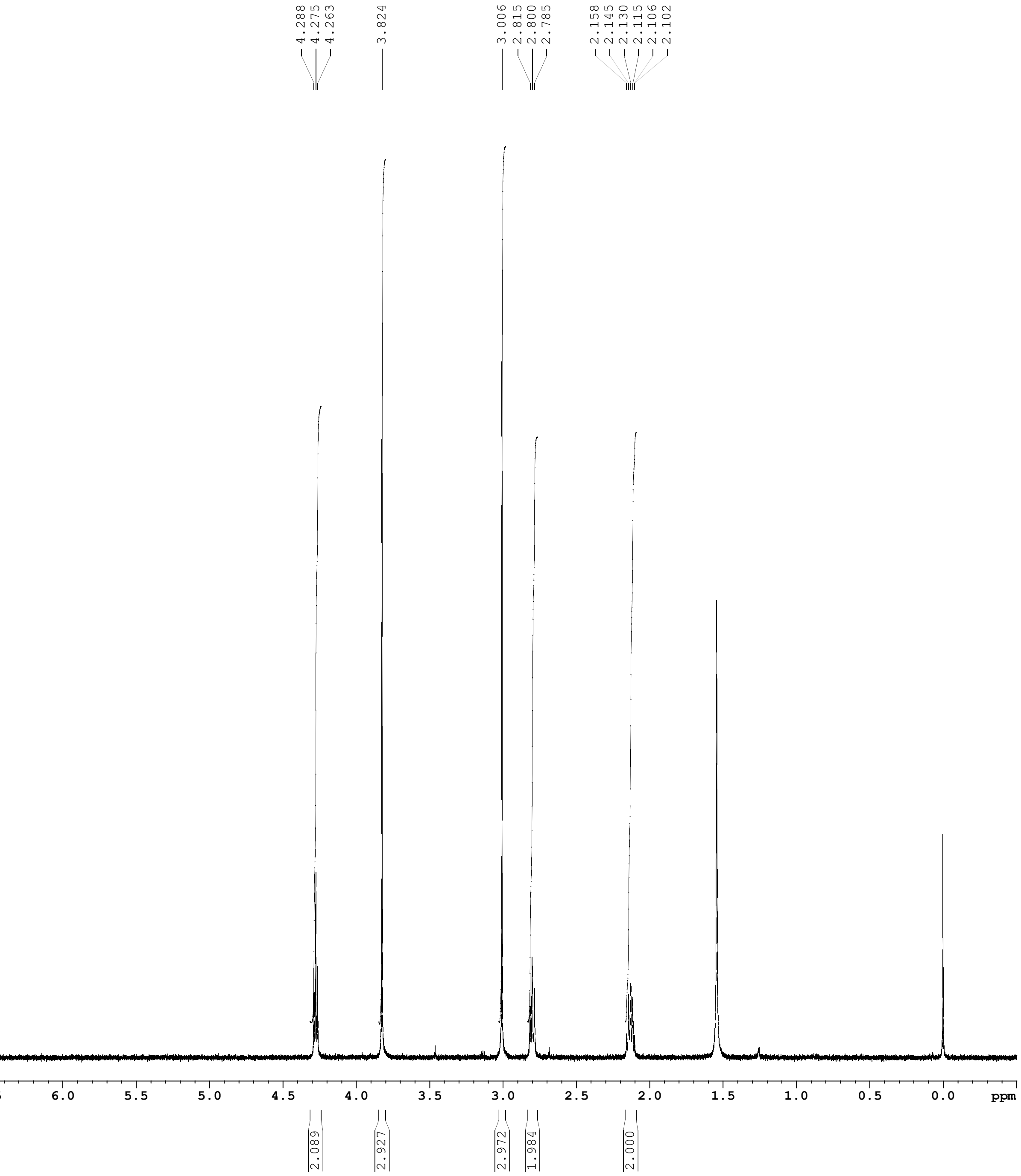


```
Current Data Parameters
NAME        cdw-2-176-p
EXPNO                 1
PROCNO                1

F2 - Acquisition Parameters
Date_          20231129
Time               8.42
INSTRUM           gn500
PROBHD   5 mm broadband
PULPROG            zg30
TD                81728
SOLVENT           CDCl3
NS                    8
DS                    2
SWH            8012.820 Hz
FIDRES         0.098043 Hz
AQ            5.0998273 sec
RG               1824.6
DW               62.400 usec
DE                 6.00 usec
TE                298.4 K
D1           0.10000000 sec
MCREST                0 sec
MCWRK        0.01500000 sec

======== CHANNEL f1 ========
NUC1                 1H
P1                12.00 usec
PL1               -6.00 dB
SFO1        498.5534899 MHz

F2 - Processing parameters
SI                65536
SF          498.5500257 MHz
WDW                  no
SSB                   0
LB                    0 Hz
GB                    0
PC                 1.00
```

13C Spectrum

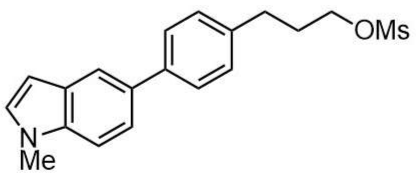

SI-11

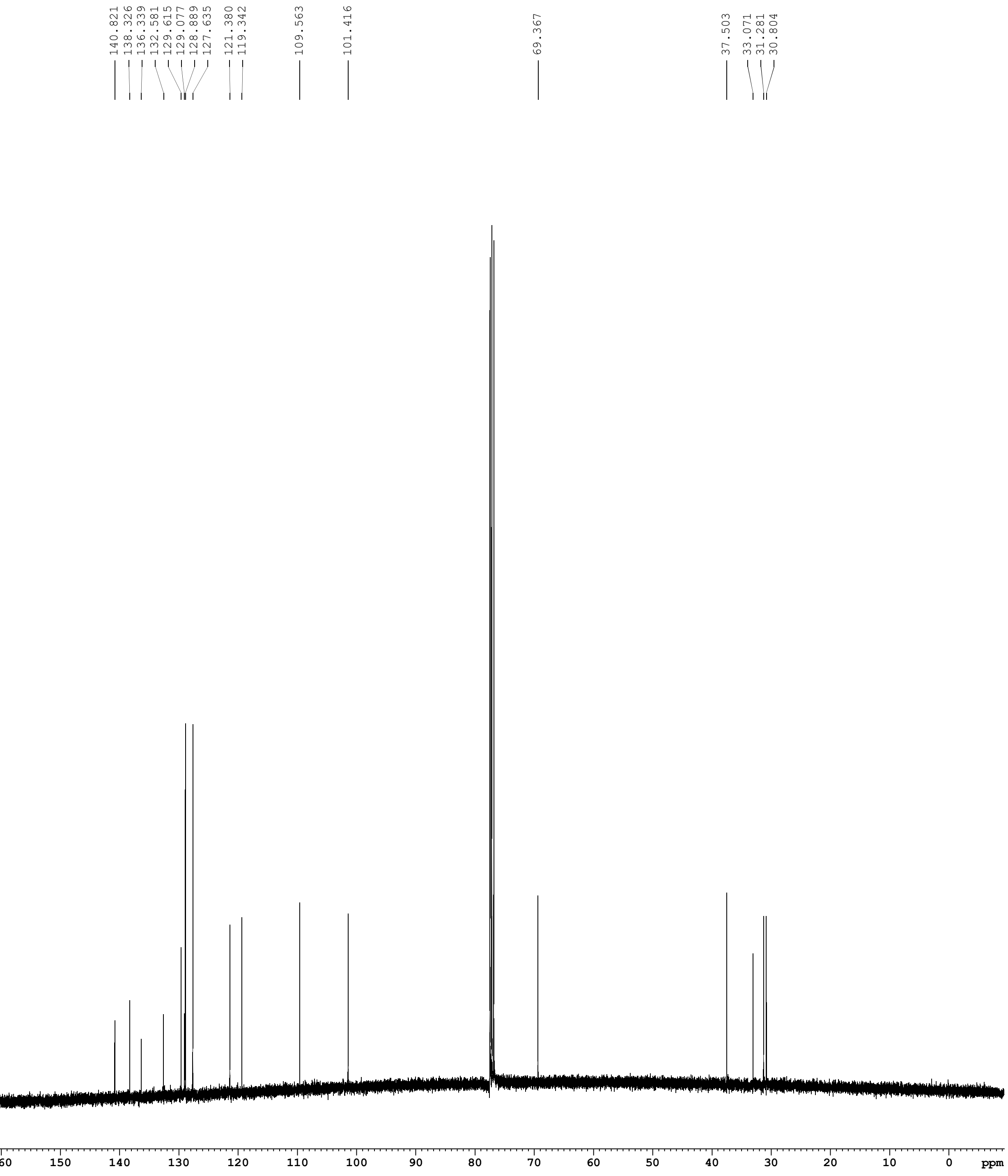

Current Data Parameters
NAME cdw2-176-c13-400
EXPNO 1
PROCNO 1

F2 - Acquisition Parameters
Date_ 20241206
Time 8.44 h
INSTRUM spect
PROBHD Z149000_0038 (
PULPROG zgpg30
TD 48074
SOLVENT CDCl3
NS 500
DS 4
SWH 24038.461 Hz
FIDRES 1.000061 Hz
AQ 0.9999392 sec
RG 48.92
DW 20.800 usec
DE 18.00 usec
TE 298.1 K
D1 0.25000000 sec
D11 0.03000000 sec
TD0 1
SFO1 100.6238359 MHz
NUC1 13C
P0 3.16 usec
P1 9.49 usec
PLW1 41.29999924 W
SFO2 400.1316005 MHz
NUC2 1H
CPDPRG[2 waltz65
PCPD2 80.00 usec
PLW2 7.41450024 W
PLW12 0.15003000 W
PLW13 0.07534500 W

F2 - Processing parameters
SI 65536
SF 100.6127573 MHz
WDW no
SSB 0
LB 0 Hz
GB 0
PC 1.40

1H spectrum

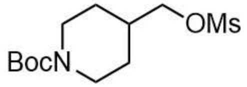

SI-12

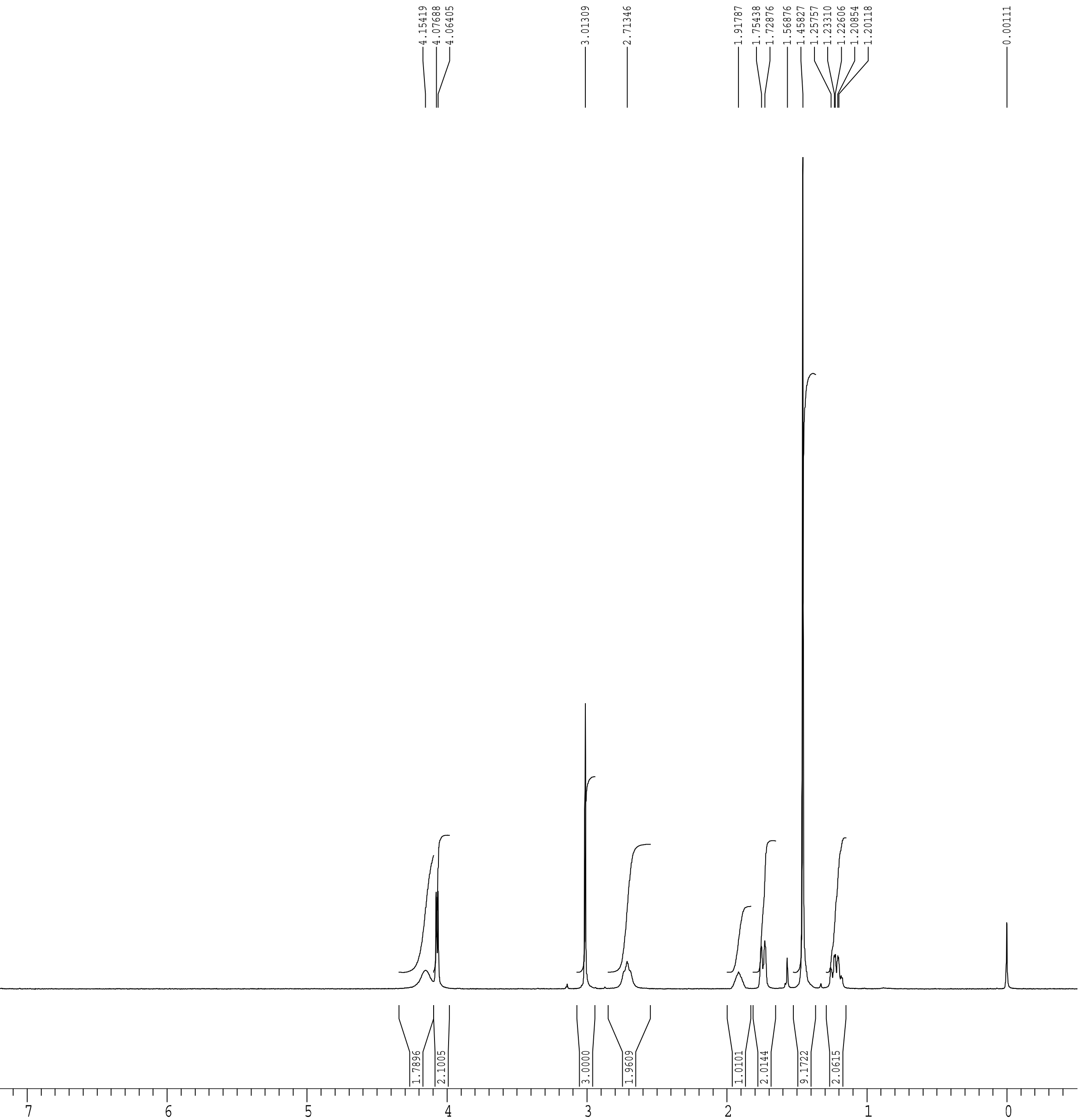

Current Data Parameters
USER nhirbawi
NAME NH-4-192-column
EXPNO 1
PROCNO 1

F2 - Acquisition Parameters
Date_ 20240411
Time 11.48
INSTRUM gn500
PROBHD 5 mm broadband
PULPROG zg30
TD 48074
SOLVENT CDCl3T
NS 8
DS 2
SWH 8012.820 Hz
FIDRES 0.166677 Hz
AQ 2.9998677 sec
RG 1290.2
DW 62.400 usec
DE 6.00 usec
TE 298.0 K
D1 0.10000000 sec
MCREST 0.00000000 sec
MCWRK 0.01500000 sec

======== CHANNEL f1 ========
NUC1 1H
P1 12.00 usec
PL1 -6.00 dB
SFO1 498.4534891 MHz

F2 - Processing parameters
SI 65536
SF 498.4500283 MHz
WDW EM
SSB 0
LB 0.30 Hz
GB 0
PC 1.00

1D NMR plot parameters
CX 20.00 cm
CY 12.50 cm
F1P 9.000 ppm
F1 4486.05 Hz
F2P -0.500 ppm
F2 -249.23 Hz
PPMCM 0.47500 ppm/cm
HZCM 236.76576 Hz/cm

1H spectrum

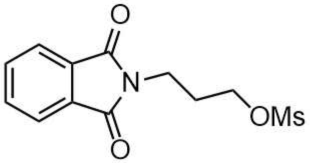

SI-13

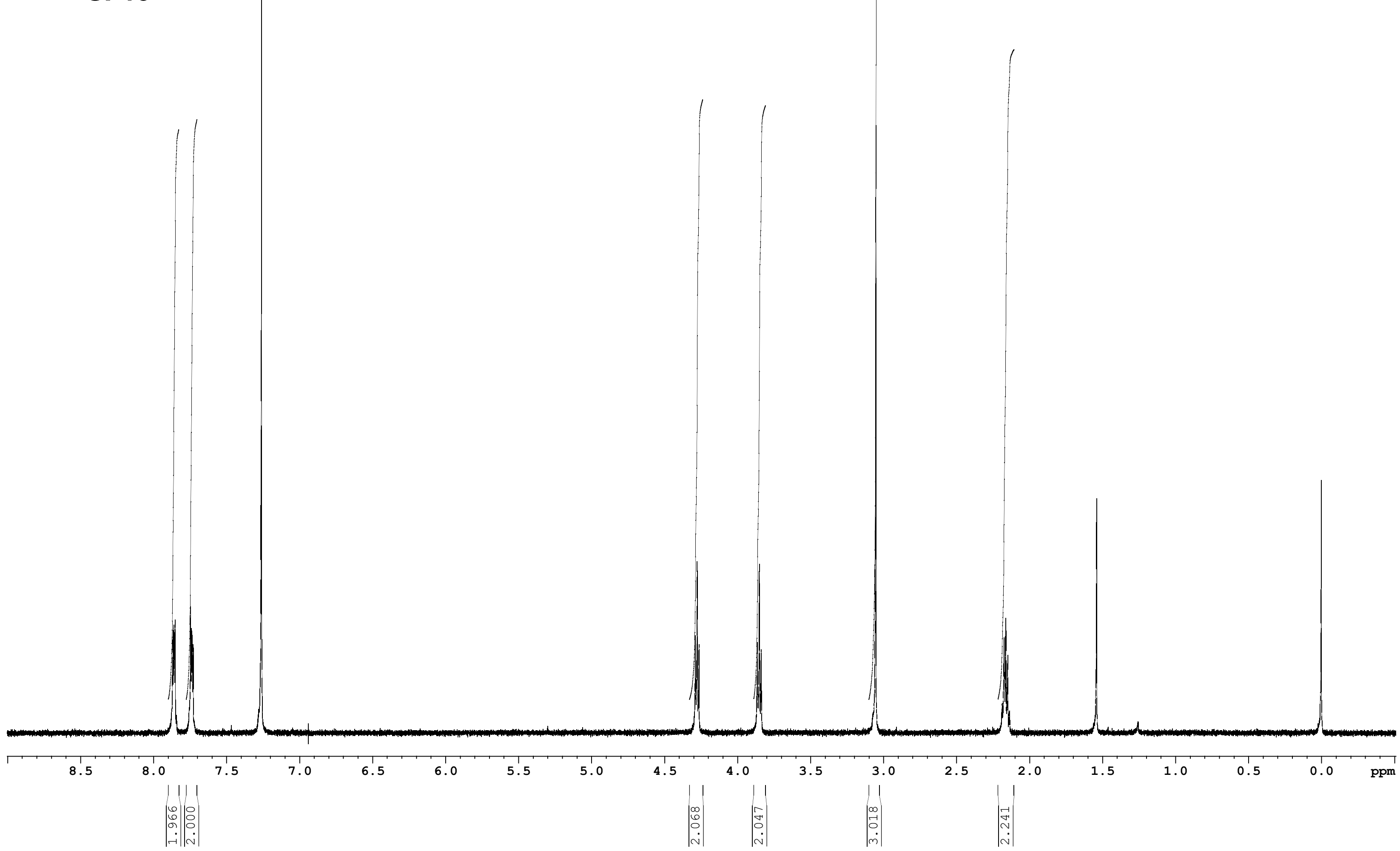


```
Current Data Parameters
NAME        cdw-2-241-p
EXPNO                 1
PROCNO                1

F2 - Acquisition Parameters
Date_          20240120
Time              12.06
INSTRUM           gn500
PROBHD   5 mm broadband
PULPROG            zg30
TD                81728
SOLVENT           CDCl3
NS                    8
DS                    2
SWH            8012.820 Hz
FIDRES         0.098043 Hz
AQ            5.0998273 sec
RG                 2048
DW               62.400 usec
DE                 6.00 usec
TE                298.2 K
D1           0.10000000 sec
MCREST                0 sec
MCWRK        0.01500000 sec

======== CHANNEL f1 ========
NUC1                 1H
P1                12.00 usec
PL1               -6.00 dB
SFO1        498.4534891 MHz

F2 - Processing parameters
SI                65536
SF          498.4500294 MHz
WDW                  no
SSB                   0
LB                    0 Hz
GB                    0
PC                 1.00
```

1H spectrum

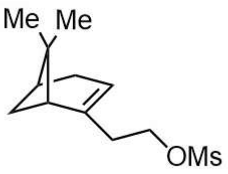

SI-14

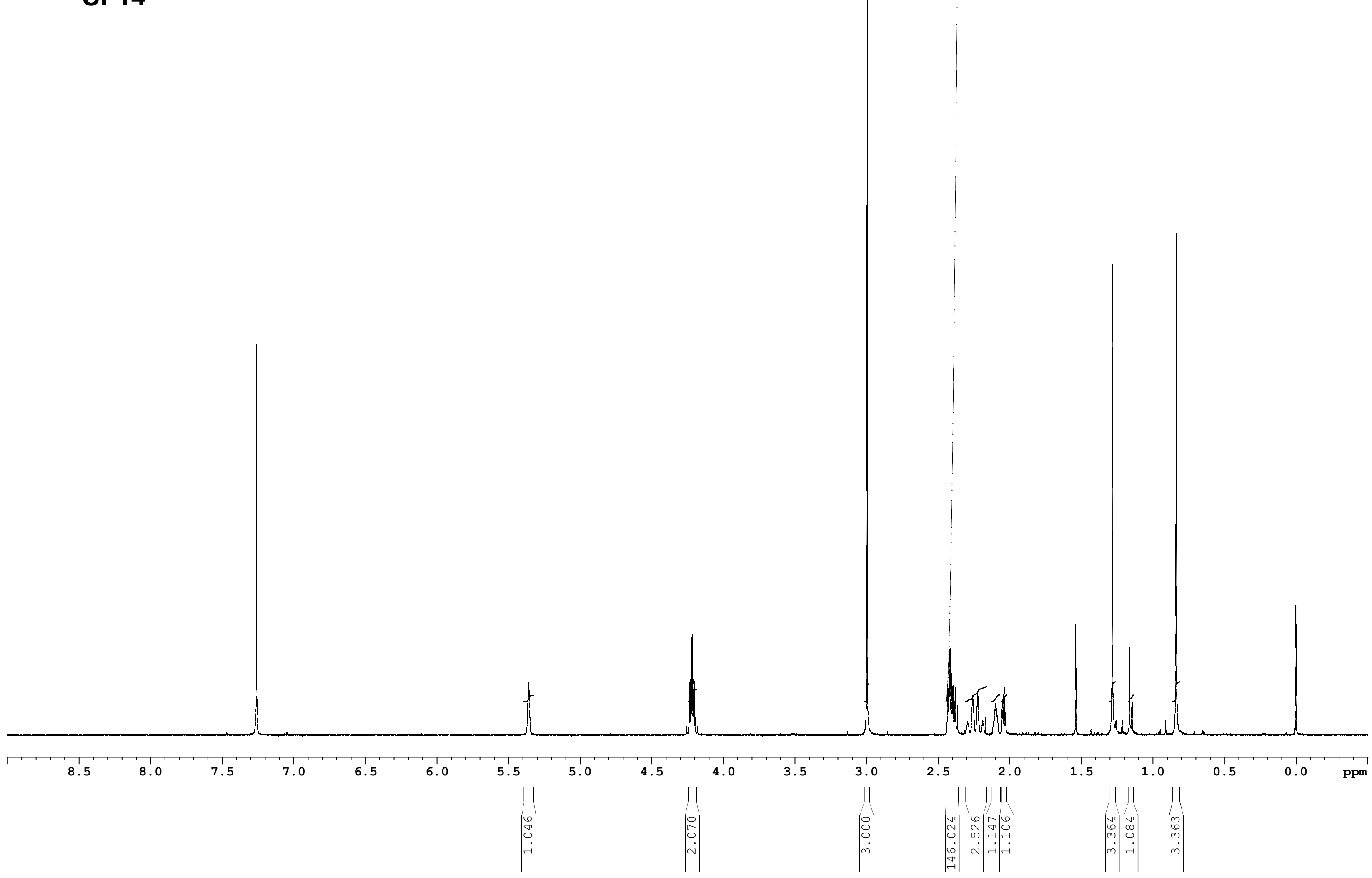


```
Current Data Parameters
NAME        cdw-2-240-p
EXPNO                 1
PROCNO                1

F2 - Acquisition Parameters
Date_          20240120
Time              12.02
INSTRUM           gn500
PROBHD   5 mm broadband
PULPROG            zg30
TD                81728
SOLVENT           CDCl3
NS                    8
DS                    2
SWH            8012.820 Hz
FIDRES         0.098043 Hz
AQ            5.0998273 sec
RG               1290.2
DW               62.400 usec
DE                 6.00 usec
TE                298.4 K
D1           0.10000000 sec
MCREST                0 sec
MCWRK        0.01500000 sec

======== CHANNEL f1 ========
NUC1                 1H
P1                12.00 usec
PL1               -6.00 dB
SFO1        498.4534891 MHz

F2 - Processing parameters
SI                65536
SF          498.4500301 MHz
WDW                  no
SSB                   0
LB                    0 Hz
GB                    0
PC                 1.00
```

1H spectrum

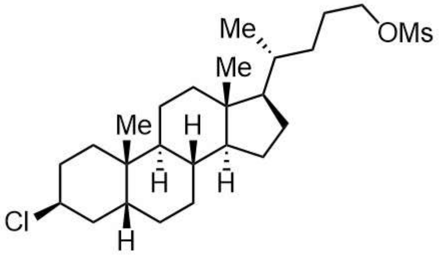

SI-15

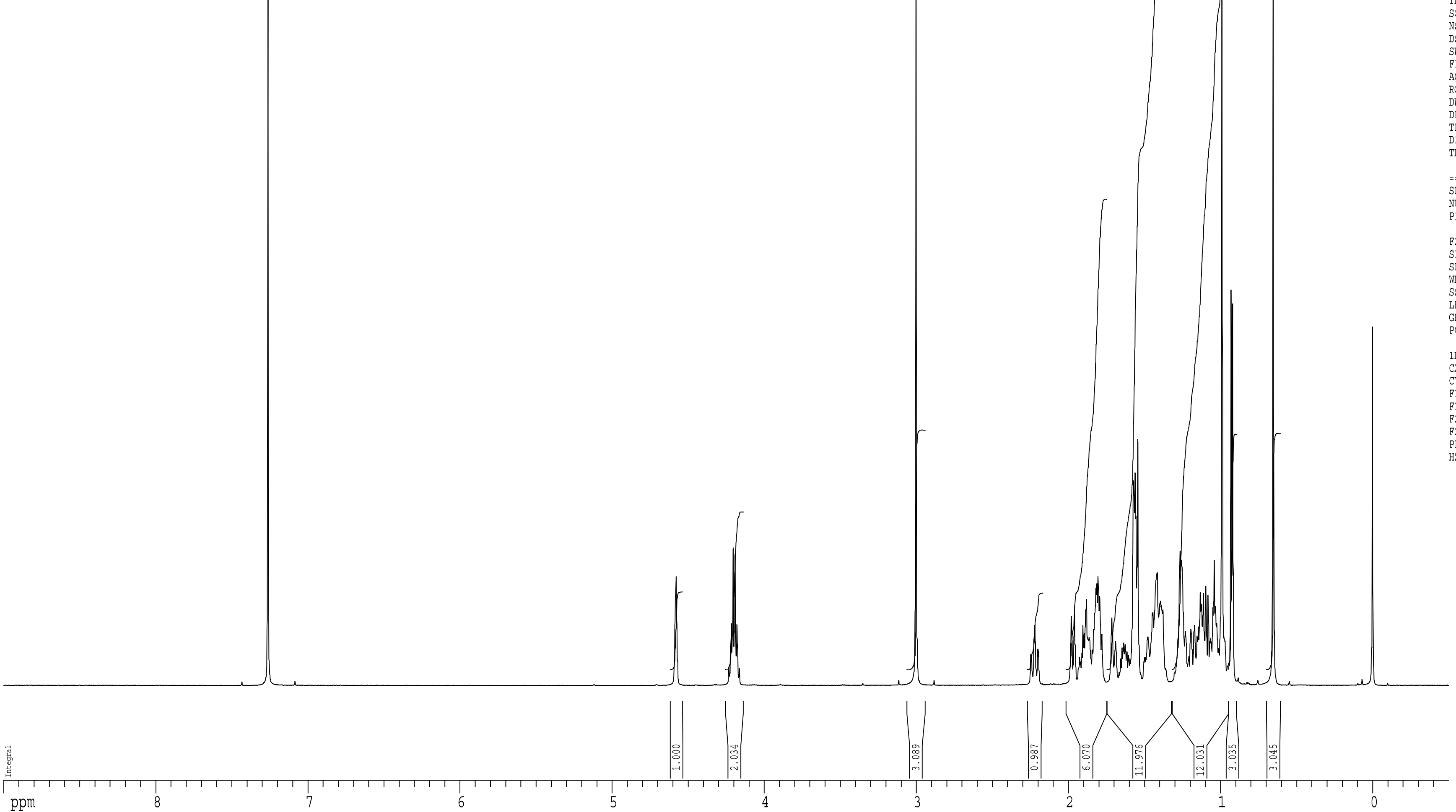

Current Data Parameters
USER nhirbawi
NAME NH-4-193
EXPNO 2
PROCNO 1

F2 - Acquisition Parameters
Date_ 20240528
Time 18.34
INSTRUM av600
PROBHD 5 mm CPBBO BB-
PULPROG zg30
TD 98074
SOLVENT CDCl3T
NS 8
DS 2
SWH 9615.385 Hz
FIDRES 0.098042 Hz
AQ 5.0998979 sec
RG 32
DW 52.000 usec
DE 14.12 usec
TE 298.0 K
D1 0.10000000 sec
TD0 1

======== CHANNEL f1 ========
SFO1 600.1342009 MHz
NUC1 1H
P1 10.00 usec

F2 - Processing parameters
SI 65536
SF 600.1300341 MHz
WDW EM
SSB 0
LB 0.30 Hz
GB 0
PC 1.00

1D NMR plot parameters
CX 22.80 cm
CY 15.00 cm
F1P 9.000 ppm
F1 5401.17 Hz
F2P -0.500 ppm
F2 -300.06 Hz
PPMCM 0.41667 ppm/cm
HZCM 250.05418 Hz/cm

13C spectrum with 1H decoupling

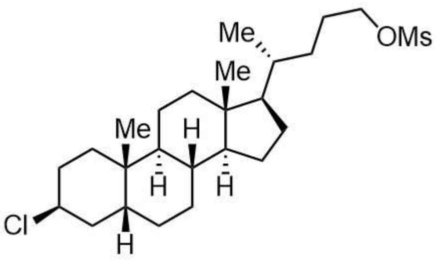

SI-15

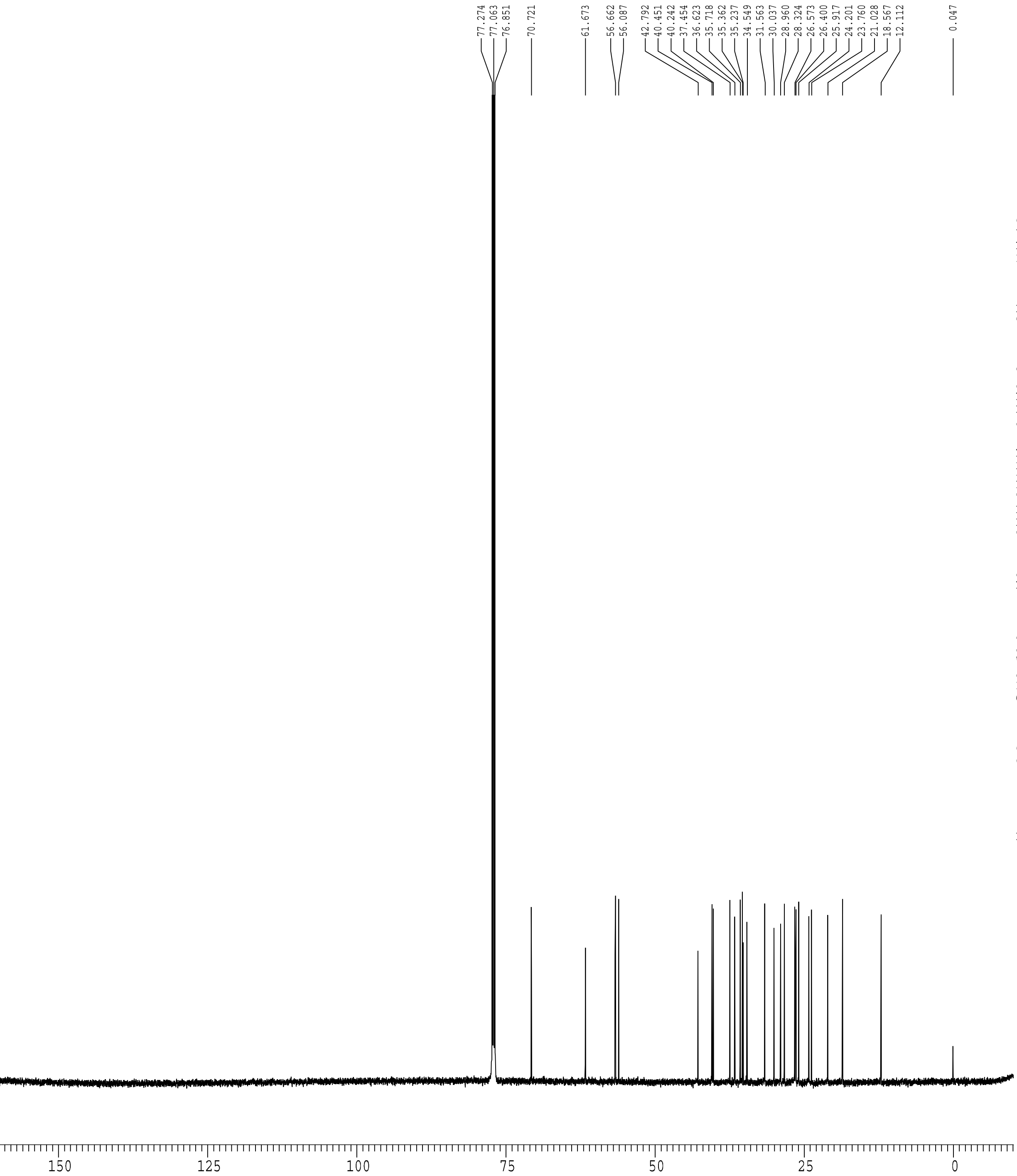

Current Data Parameters
USER nhirbawi
NAME NH-4-193
EXPNO 1
PROCNO 1

F2 - Acquisition Parameters
Date_ 20240528
Time 18.26
INSTRUM av600
PROBHD 5 mm CPBBO BB-
PULPROG zgdc30
TD 65536
SOLVENT CDCl3T
NS 483
DS 4
SWH 36231.883 Hz
FIDRES 0.552855 Hz
AQ 0.9044468 sec
RG 2050
DW 13.800 usec
DE 19.65 usec
TE 297.9 K
D1 0.40000001 sec
D11 0.03000000 sec
TD0 1

======== CHANNEL f1 ========
SFO1 150.9194080 MHz
NUC1 13C
P1 10.00 usec

F2 - Processing parameters
SI 65536
SF 150.9028085 MHz
WDW EM
SSB 0
LB 1.00 Hz
GB 0
PC 1.00

1D NMR plot parameters
CX 22.80 cm
CY 40.00 cm
F1P 229.520 ppm
F1 34635.16 Hz
F2P -10.507 ppm
F2 -1585.47 Hz
PPMCM 10.52747 ppm/cm
HZCM 1588.62451 Hz/cm

1H spectrum

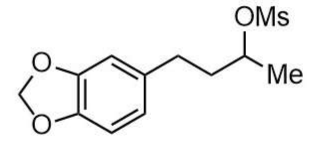

(±)-54

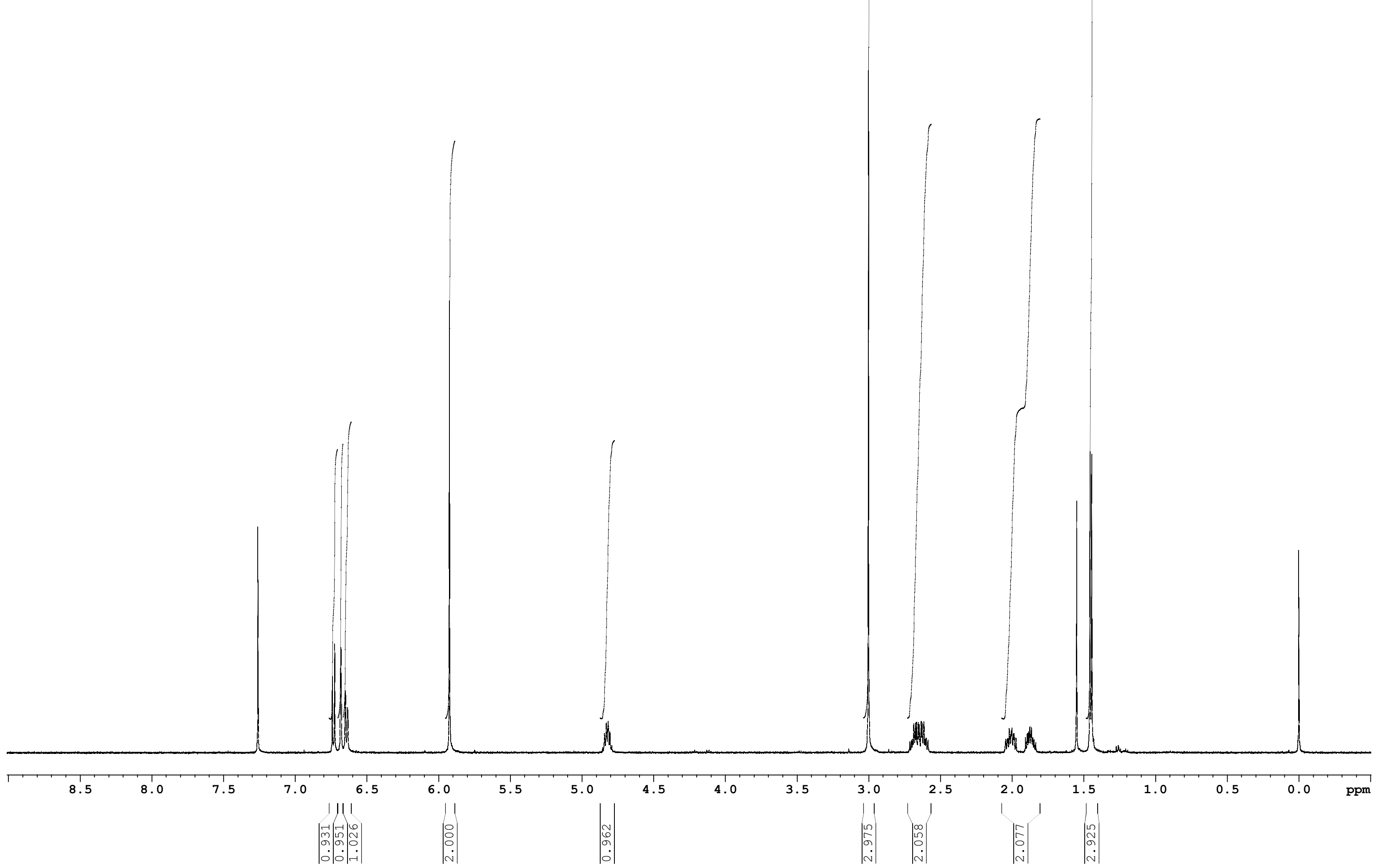

Current Data Parameters
NAME cdw-2-276-p
EXPNO 1
PROCNO 1

F2 - Acquisition Parameters
Date_ 20240216
Time 8.42
INSTRUM gn500
PROBHD 5 mm broadband
PULPROG zg30
TD 81728
SOLVENT CDCl3
NS 8
DS 2
SWH 8012.820 Hz
FIDRES 0.098043 Hz
AQ 5.0998273 sec
RG 1448.2
DW 62.400 usec
DE 6.00 usec
TE 298.0 K
D1 0.10000000 sec
MCREST 0 sec
MCWRK 0.01500000 sec

======== CHANNEL f1 ========
NUC1 1H
P1 12.00 usec
PL1 -6.00 dB
SFO1 498.4534891 MHz

F2 - Processing parameters
SI 65536
SF 498.4500310 MHz
WDW no
SSB 0
LB 0 Hz
GB 0
PC 1.00

1H spectrum

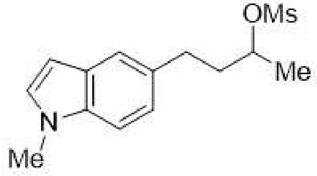

SI-16

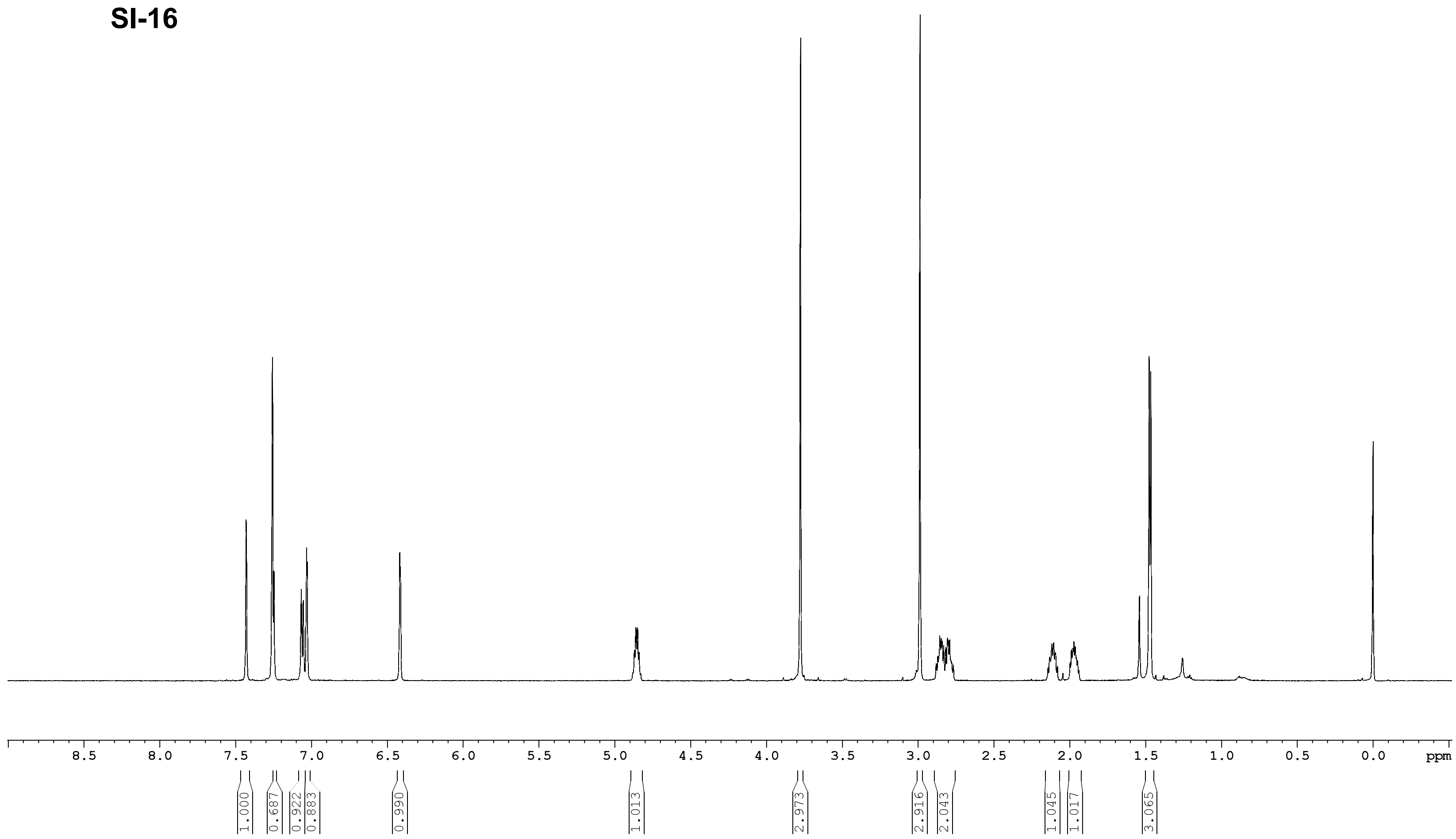

Current Data Parameters
NAME LCB-1-25-col-proton
EXPNO 1
PROCNO 1

F2 - Acquisition Parameters
Date_ 20240405
Time 13.57
INSTRUM av600
PROBHD 5 mm CPBBO BB-
PULPROG zg30
TD 98074
SOLVENT CDCl3T
NS 8
DS 2
SWH 9615.385 Hz
FIDRES 0.098042 Hz
AQ 5.0998478 sec
RG 20.2
DW 52.000 usec
DE 14.12 usec
TE 298.1 K
D1 0.10000000 sec
TD0 1

======== CHANNEL f1 ========
SFO1 600.1342009 MHz
NUC1 1H
P1 10.00 usec
PLW1 30.00000000 W

F2 - Processing parameters
SI 65536
SF 600.1300367 MHz
WDW EM
SSB 0
LB 0.30 Hz
GB 0
PC 1.00

13C spectrum with 1H decoupling

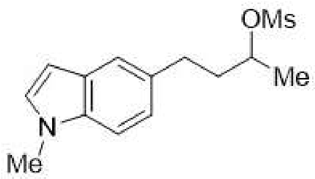

SI-16

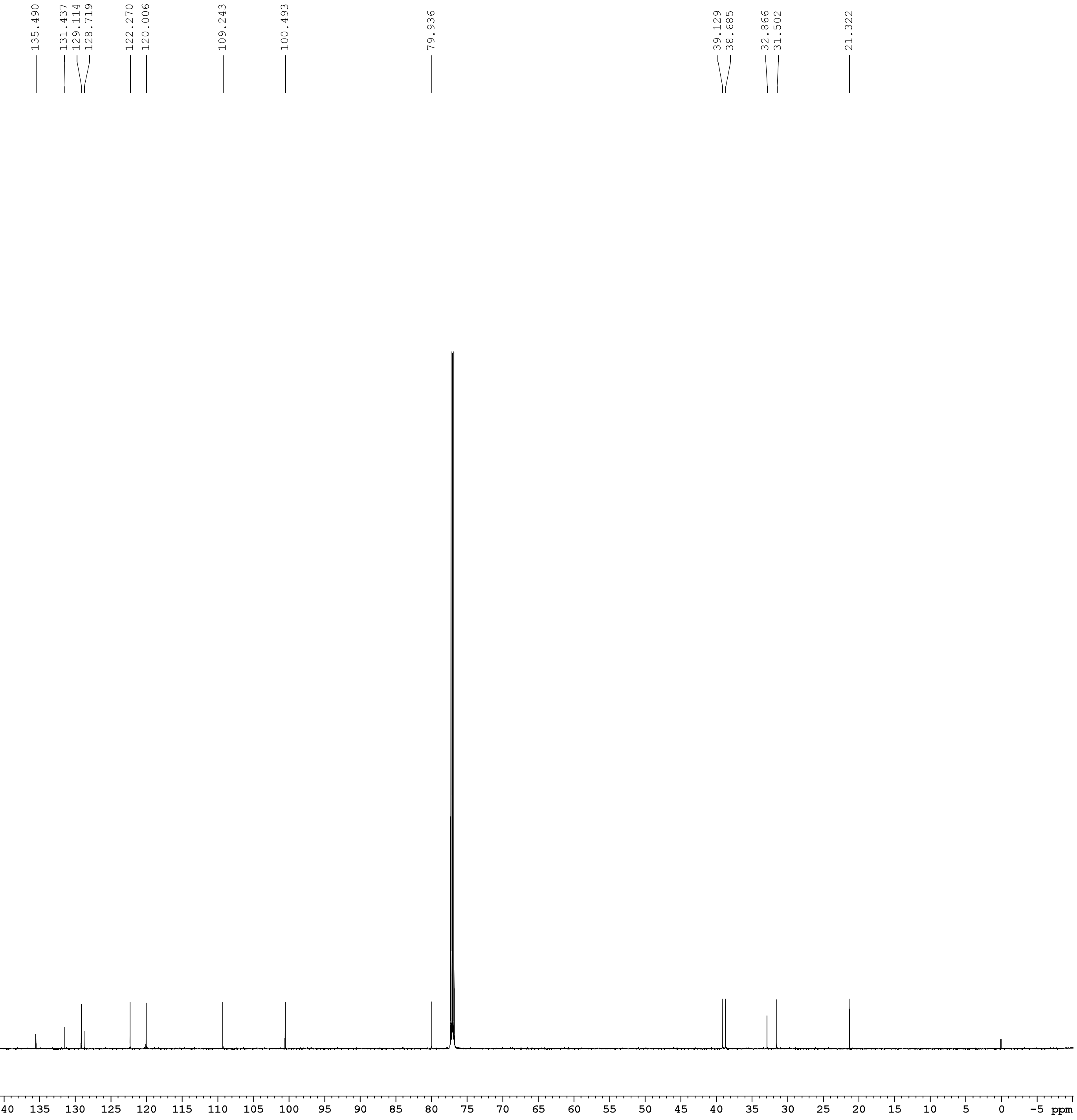


```
Current Data Parameters
NAME       LCB-1-25-col
EXPNO                 1
PROCNO                1

F2 - Acquisition Parameters
Date_          20240405
Time              13.45
INSTRUM           av600
PROBHD   5 mm CPBBO BB-
PULPROG         zgdc30
TD                65536
SOLVENT          CDCl3T
NS                  630
DS                    4
SWH           36231.883 Hz
FIDRES         0.552855 Hz
AQ            0.9043968 sec
RG                 2050
DW               13.800 usec
DE                19.65 usec
TE                298.1 K
D1           0.40000001 sec
D11          0.03000000 sec
TD0                   1

======== CHANNEL f1 ========
SFO1        150.9194080 MHz
NUC1                13C
P1                10.00 usec
PLW1        68.40000153 W

======== CHANNEL f2 ========
SFO2        600.1330010 MHz
NUC2                 1H
CPDPRG[2        waltz16
PCPD2             80.00 usec
PLW2        30.00000000 W
PLW12        0.39811000 W

F2 - Processing parameters
SI                65536
SF          150.9028160 MHz
WDW                  EM
SSB                   0
LB                 1.00 Hz
GB                    0
PC                 1.00
```

1H spectrum

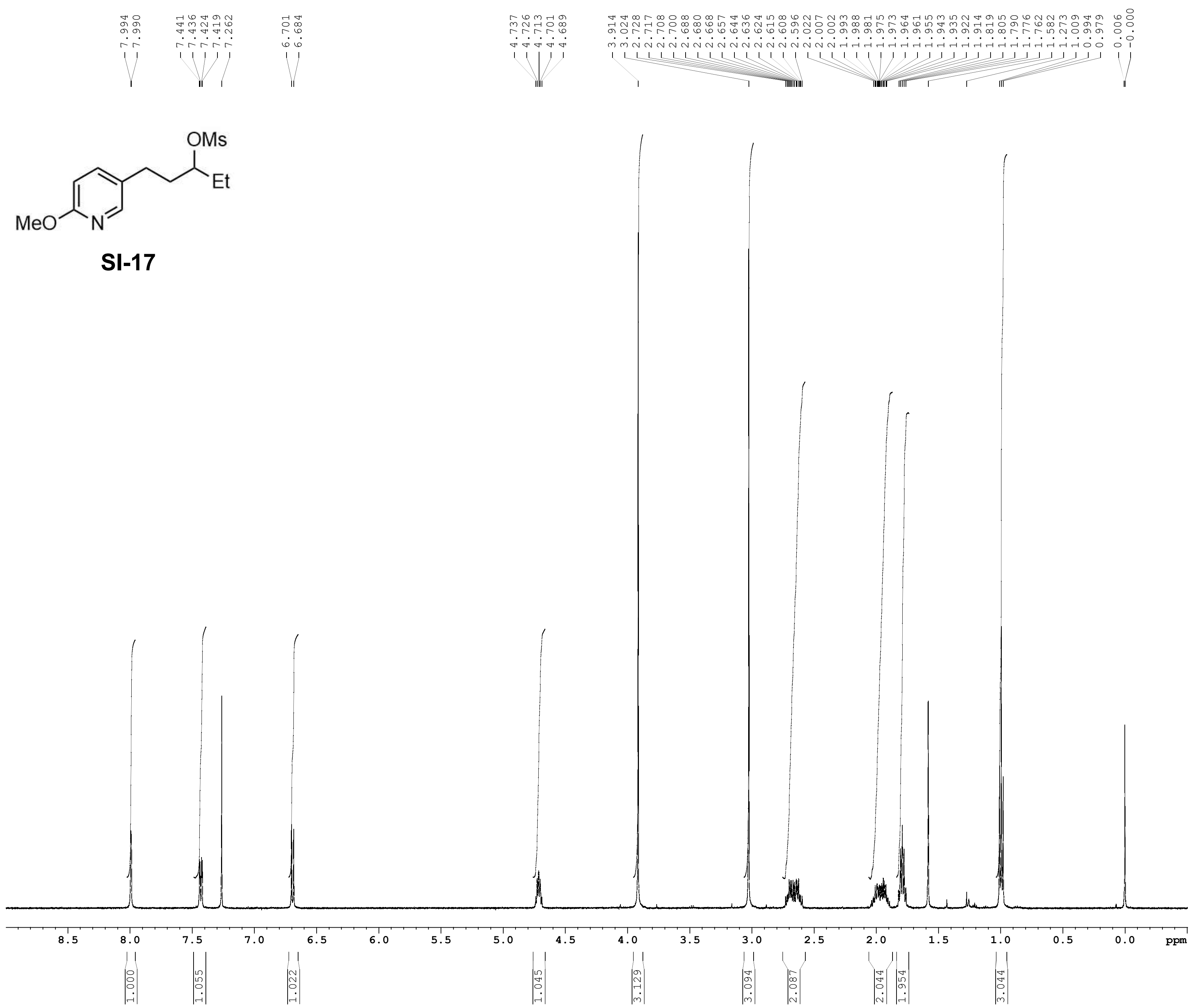

Current Data Parameters
NAME cdw-2-277-p
EXPNO 1
PROCNO 1

F2 - Acquisition Parameters
Date_ 20240216
Time 8.46
INSTRUM gn500
PROBHD 5 mm broadband
PULPROG zg30
TD 81728
SOLVENT CDCl3
NS 8
DS 2
SWH 8012.820 Hz
FIDRES 0.098043 Hz
AQ 5.0998273 sec
RG 1448.2
DW 62.400 usec
DE 6.00 usec
TE 298.0 K
D1 0.10000000 sec
MCREST 0 sec
MCWRK 0.01500000 sec

======== CHANNEL f1 ========
NUC1 1H
P1 12.00 usec
PL1 -6.00 dB
SFO1 498.4534891 MHz

F2 - Processing parameters
SI 65536
SF 498.4500299 MHz
WDW no
SSB 0
LB 0 Hz
GB 0
PC 1.00

1H spectrum

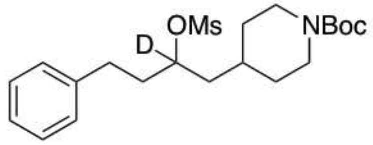

D-SI-18

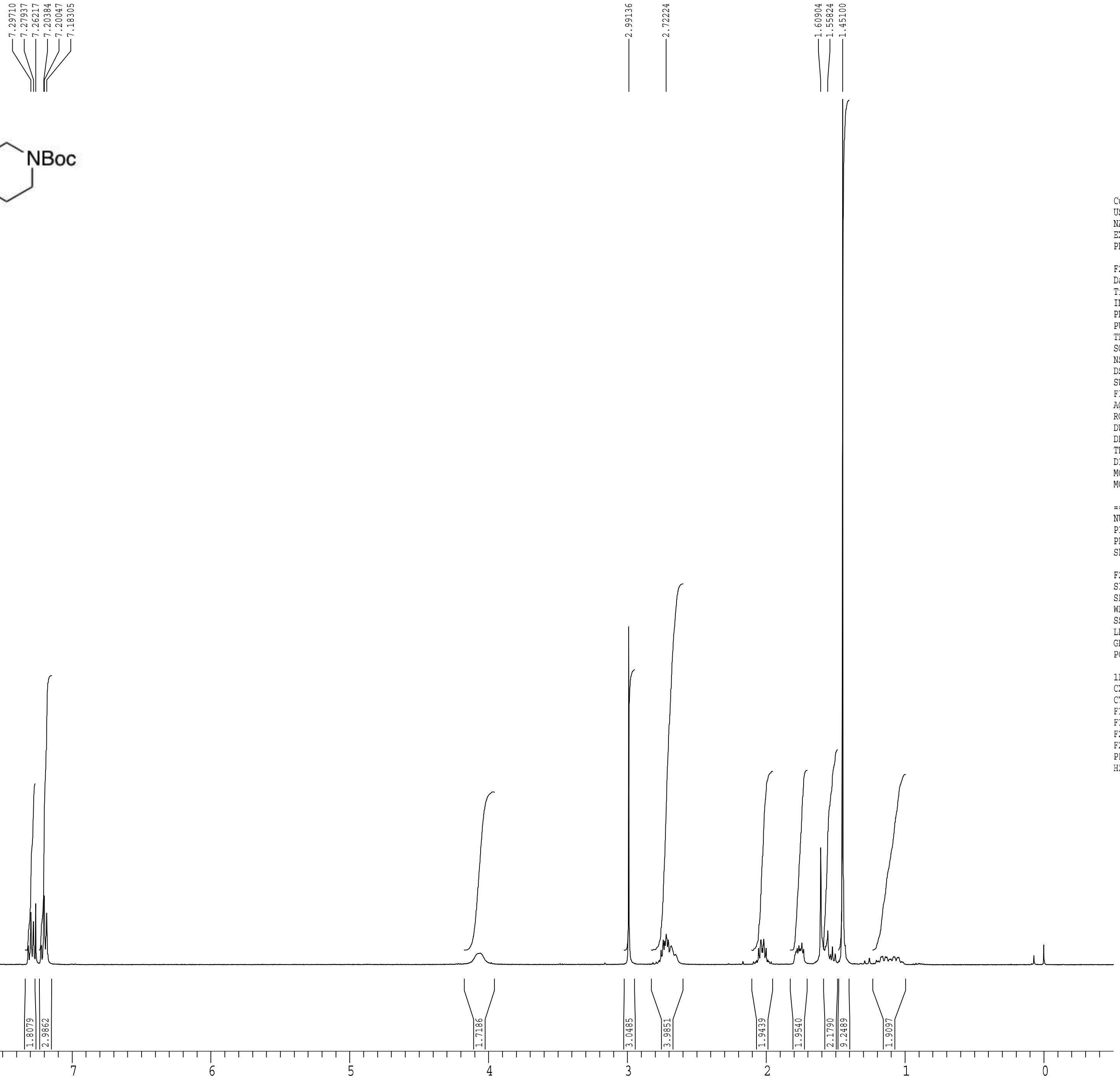

Current Data Parameters
USER chloew10
NAME cdw-2-101-1h
EXPNO 1
PROCNO 1

F2 - Acquisition Parameters
Date_ 20230831
Time 8.24
INSTRUM drx400
PROBHD 5 mm QNP H/F/P
PULPROG zg30
TD 65536
SOLVENT CDCl3
NS 8
DS 2
SWH 6410.256 Hz
FIDRES 0.097813 Hz
AQ 5.1118579 sec
RG 256
DW 78.000 usec
DE 4.50 usec
TE 298.0 K
D1 0.10000000 sec
MCREST 0.00000000 sec
MCWRK 0.01500000 sec

======== CHANNEL f1 ========
NUC1 1H
P1 12.00 usec
PL1 -1.10 dB
SFO1 400.1328009 MHz

F2 - Processing parameters
SI 65536
SF 400.1300207 MHz
WDW no
SSB 0
LB 0.00 Hz
GB 0
PC 2.00

1D NMR plot parameters
CX 22.80 cm
CY 15.00 cm
F1P 9.000 ppm
F1 3601.17 Hz
F2P -0.500 ppm
F2 -200.06 Hz
PPMCM 0.41667 ppm/cm
HZCM 166.72086 Hz/cm

1H spectrum

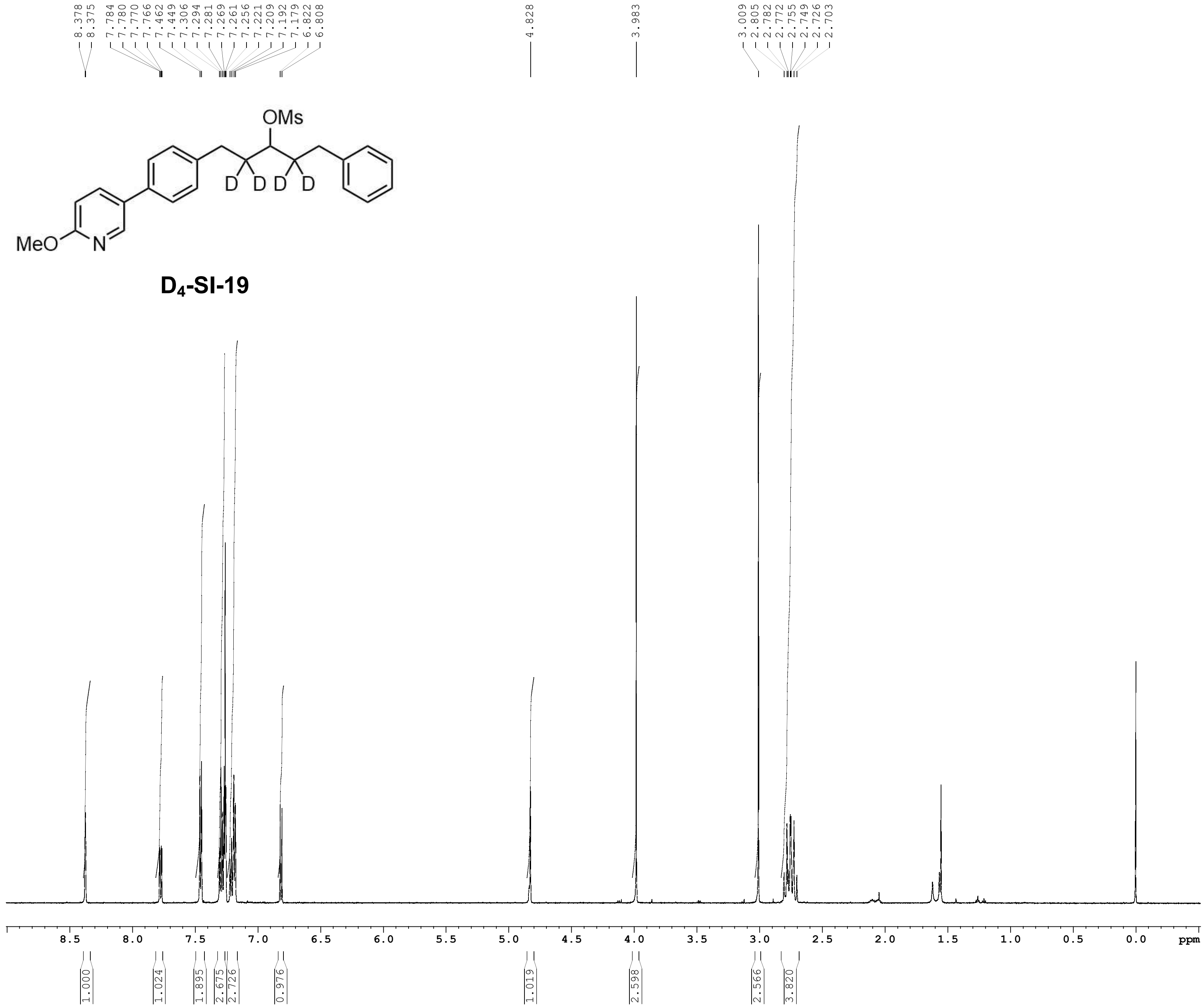

D_4-SI-19

Current Data Parameters
NAME cdw-2-135-1h
EXPNO 1
PROCNO 1

F2 - Acquisition Parameters
Date_ 20231014
Time 11.53
INSTRUM av600
PROBHD 5 mm CPBBO BB-
PULPROG zg30
TD 98074
SOLVENT CDCl3
NS 8
DS 2
SWH 9615.385 Hz
FIDRES 0.098042 Hz
AQ 5.0998478 sec
RG 10
DW 52.000 usec
DE 14.12 usec
TE 298.0 K
D1 0.10000000 sec
TD0 1

======== CHANNEL f1 ========
SFO1 600.1342009 MHz
NUC1 1H
P1 10.00 usec
PLW1 30.00000000 W

F2 - Processing parameters
SI 65536
SF 600.1300345 MHz
WDW no
SSB 0
LB 0 Hz
GB 0
PC 1.00

13C spectrum with 1H decoupling

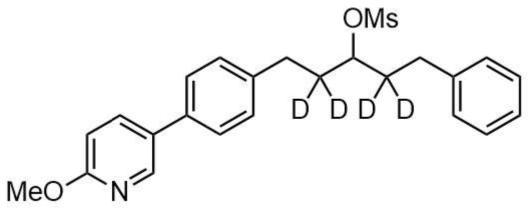

D_4-SI-19

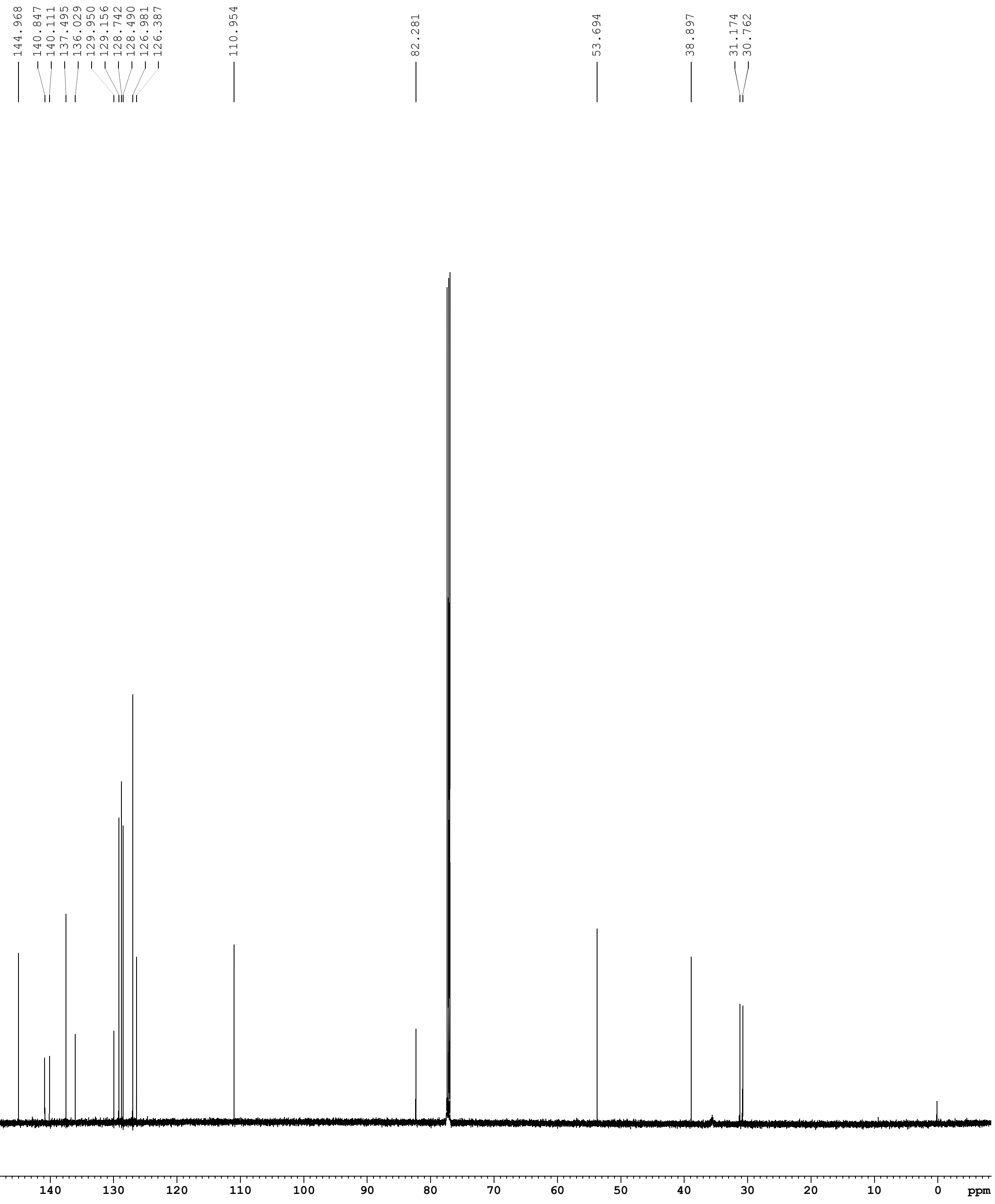


```
Current Data Parameters
NAME       cdw-2-135-c13
EXPNO                  1
PROCNO                 1

F2 - Acquisition Parameters
Date_           20231014
Time               11.59
INSTRUM            av600
PROBHD   5 mm CPBBO BB-
PULPROG           zgdc30
TD                 65536
SOLVENT            CDCl3
NS                   500
DS                     4
SWH            36231.883 Hz
FIDRES          0.552855 Hz
AQ             0.9043968 sec
RG                  2050
DW                13.800 usec
DE                 19.65 usec
TE                 297.9 K
D1            0.40000001 sec
D11           0.03000000 sec
TD0                    1

======== CHANNEL f1 ========
SFO1         150.9194080 MHz
NUC1                 13C
P1                 10.00 usec
PLW1         68.40000153 W

======== CHANNEL f2 ========
SFO2         600.1330010 MHz
NUC2                  1H
CPDPRG[2          waltz16
PCPD2              80.00 usec
PLW2         30.00000000 W
PLW12         0.39811000 W

F2 - Processing parameters
SI                 65536
SF           150.9027936 MHz
WDW                   no
SSB                    0
LB                     0 Hz
GB                     0
PC                  1.00
```

1H spectrum

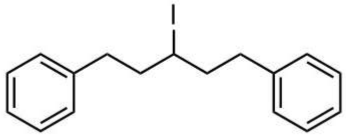

3

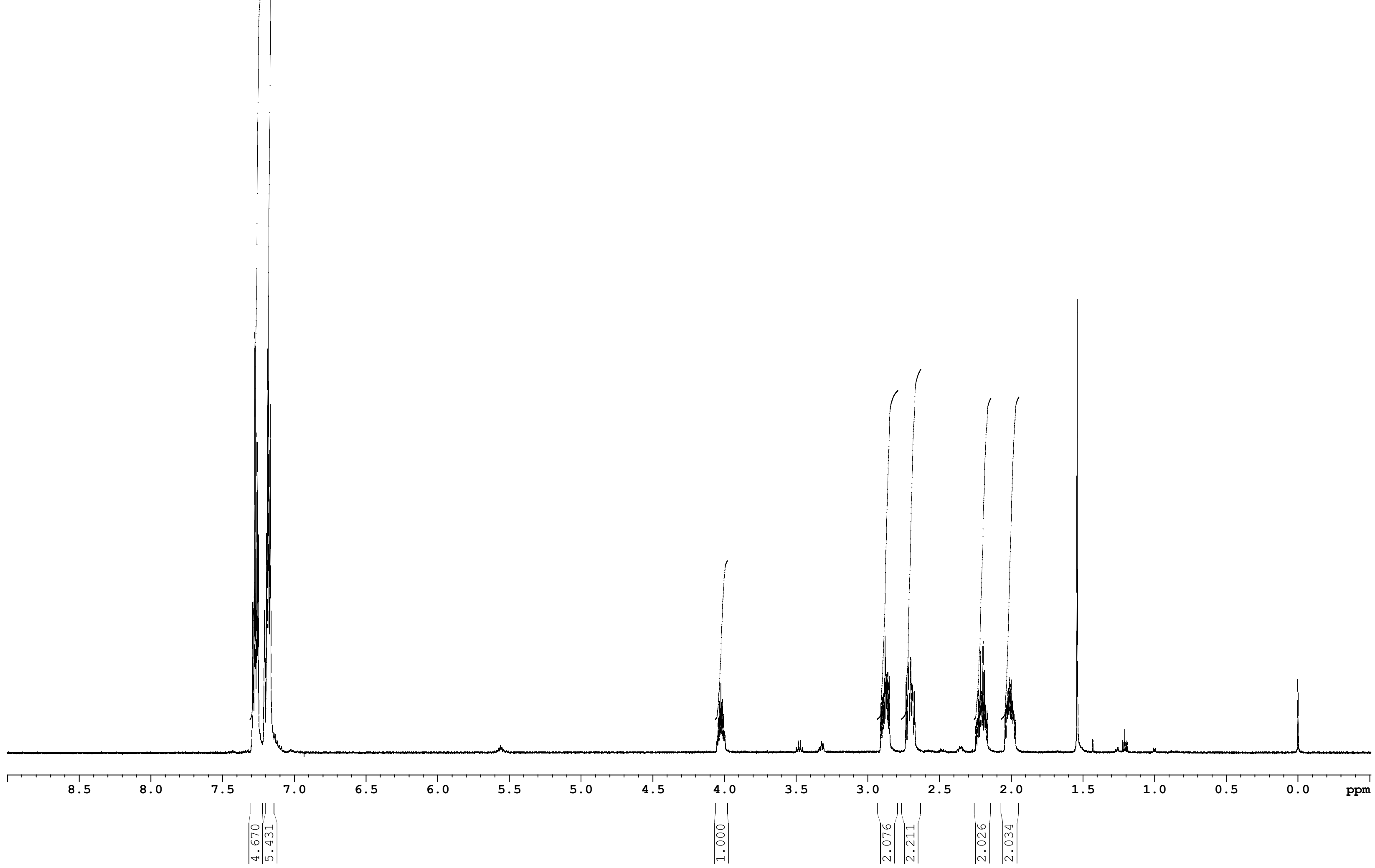

Current Data Parameters
NAME cdw3-243-p
EXPNO 1
PROCNO 1

F2 - Acquisition Parameters
Date_ 20241029
Time 8.52
INSTRUM gn500
PROBHD 5 mm broadband
PULPROG zg30
TD 81728
SOLVENT CDCl3
NS 8
DS 2
SWH 8012.820 Hz
FIDRES 0.098043 Hz
AQ 5.0998273 sec
RG 912.3
DW 62.400 usec
DE 6.00 usec
TE 298.0 K
D1 0.10000000 sec
MCREST 0 sec
MCWRK 0.01500000 sec

======== CHANNEL f1 ========
NUC1 1H
P1 12.00 usec
PL1 -6.00 dB
SFO1 498.4534891 MHz

F2 - Processing parameters
SI 65536
SF 498.4500344 MHz
WDW no
SSB 0
LB 0 Hz
GB 0
PC 1.00

1H spectrum

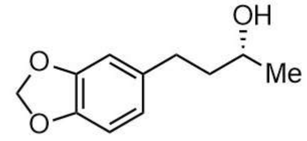

SI-(*R*)-20

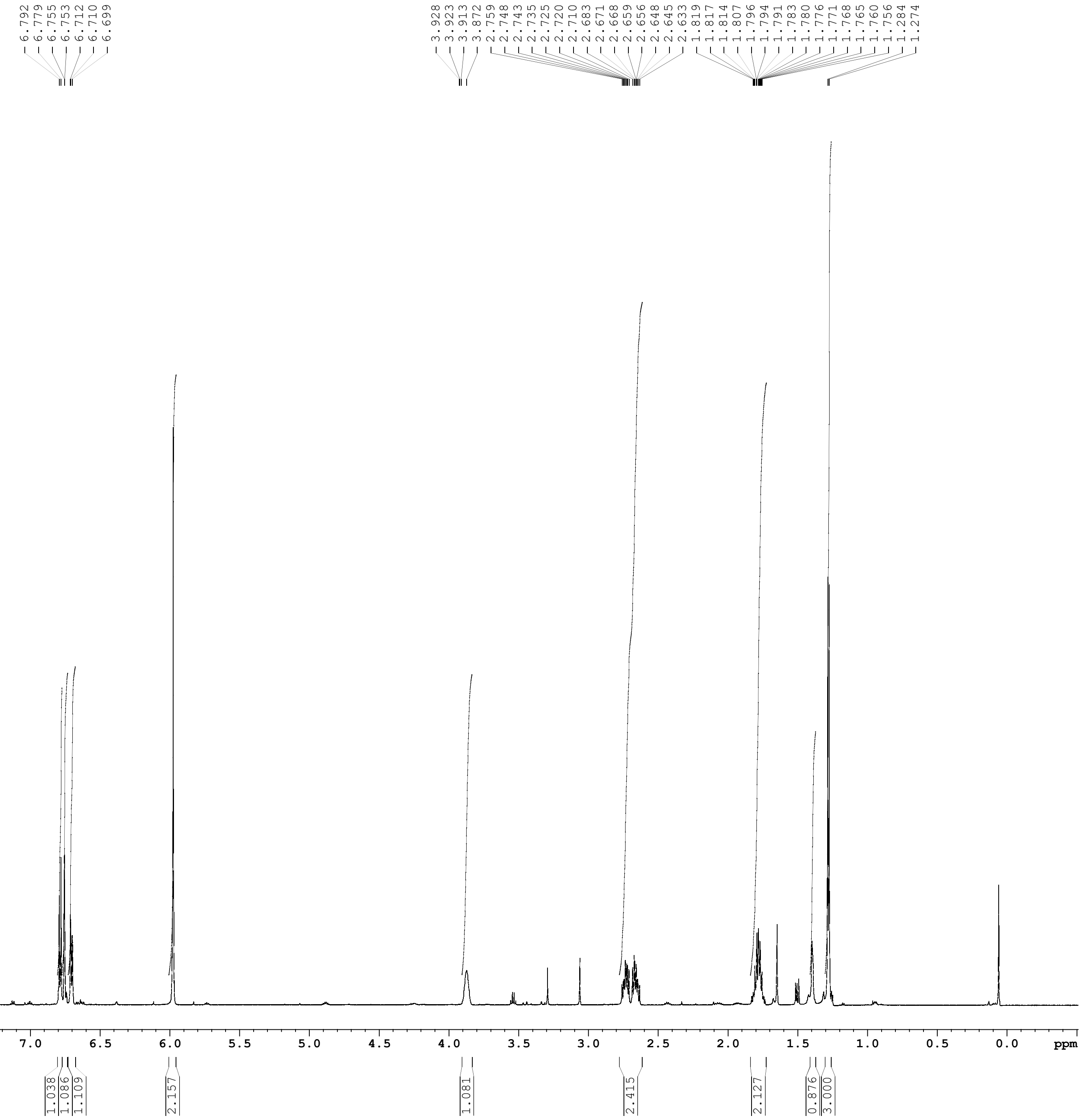


```
Current Data Parameters
NAME          cdw-2-287-2
EXPNO                   1
PROCNO                  1

F2 - Acquisition Parameters
Date_            20240321
Time                 9.47
INSTRUM             av600
PROBHD   5 mm CPBBO BB-
PULPROG              zg30
TD                  98074
SOLVENT             CDCl3
NS                      8
DS                      2
SWH             9615.385 Hz
FIDRES          0.098042 Hz
AQ             5.0998478 sec
RG                     10
DW                 52.000 usec
DE                  14.12 usec
TE                  298.1 K
D1             0.10000000 sec
TD0                     1

======== CHANNEL f1 ========
SFO1         600.1342009 MHz
NUC1                   1H
P1                  10.00 usec
PLW1          30.00000000 W

F2 - Processing parameters
SI                  65536
SF           600.1300000 MHz
WDW                    no
SSB                     0
LB                      0 Hz
GB                      0
PC                   1.00
```

1H spectrum

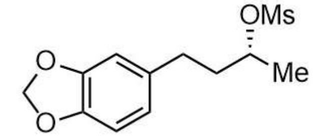

(*R*)-54

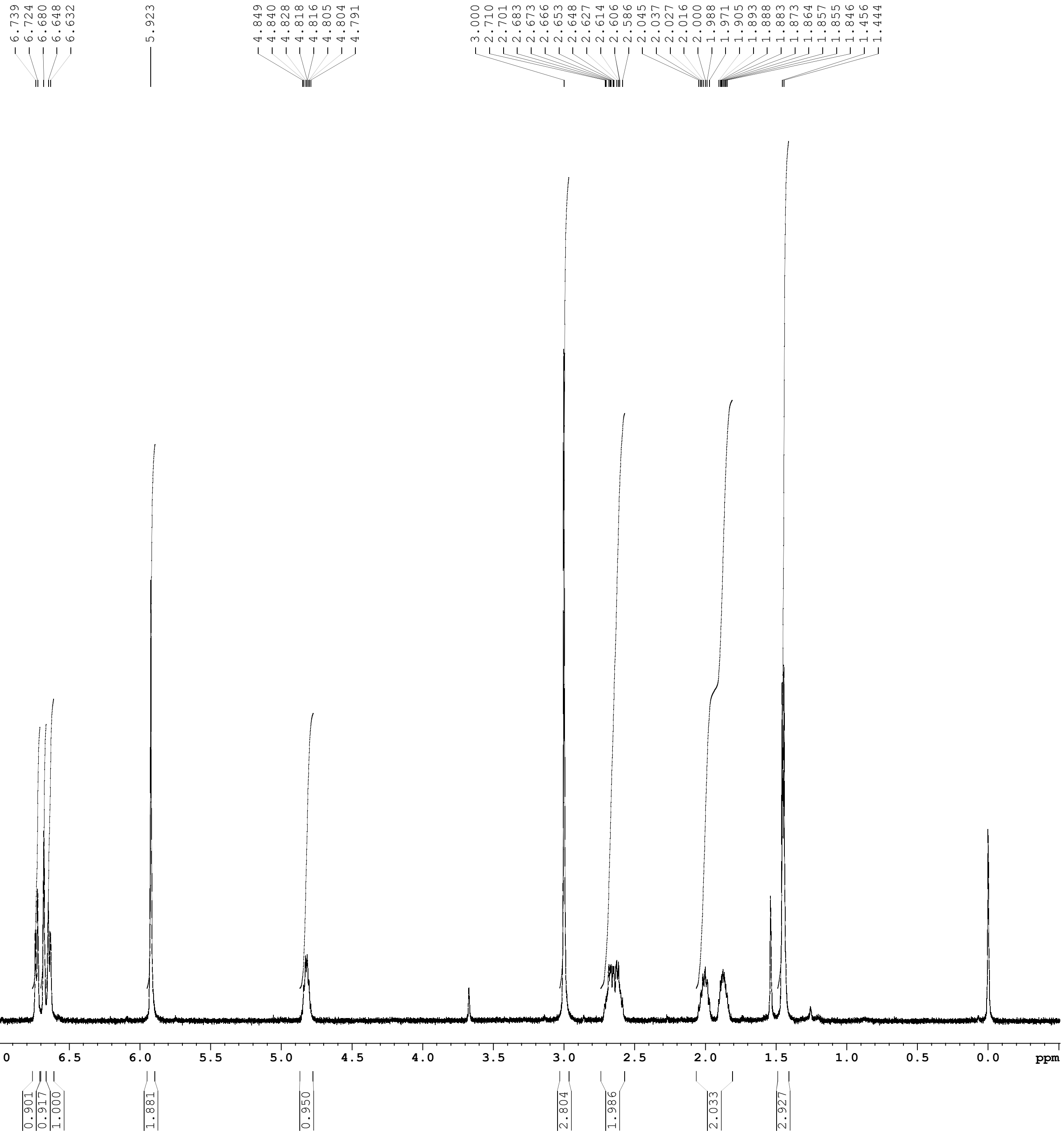

Current Data Parameters
NAME cdw-2-298-p
EXPNO 1
PROCNO 1

F2 - Acquisition Parameters
Date_ 20240327
Time 7.36
INSTRUM gn500
PROBHD 5 mm broadband
PULPROG zg30
TD 81728
SOLVENT CDCl3
NS 8
DS 2
SWH 8012.820 Hz
FIDRES 0.098043 Hz
AQ 5.0998273 sec
RG 1625.5
DW 62.400 usec
DE 6.00 usec
TE 298.0 K
D1 0.10000000 sec
MCREST 0 sec
MCWRK 0.01500000 sec

======== CHANNEL f1 ========
NUC1 1H
P1 12.00 usec
PL1 -6.00 dB
SFO1 498.4534891 MHz

F2 - Processing parameters
SI 65536
SF 498.4500317 MHz
WDW no
SSB 0
LB 0 Hz
GB 0
PC 1.00
